# Clonal relatedness between lobular carcinoma *in situ *and synchronous malignant lesions

**DOI:** 10.1186/bcr3222

**Published:** 2012-07-09

**Authors:** Victor P Andrade, Irina Ostrovnaya, Venkatraman E Seshan, Mary Morrogh, Dilip Giri, Narciso Olvera, Marina De Brot, Monica Morrow, Colin B Begg, Tari A King

**Affiliations:** 1 Department of Surgery, Hospital AC Camargo Anatomia Patológica, Predio Hilda Jacob Subsolo 2, Rua Prof. Antonio Prudente 210, Liberdade, Sao Paulo, SP 01509-010, Brazil; 2 Department of Epidemiology and Biostatistics, Memorial Sloan-Kettering Cancer Center, 1275 York Avenue, New York, NY 10065, USA; 3 Breast Service, Department of Surgery, Memorial Sloan-Kettering Cancer Center, 1275 York Avenue, New York, NY 10065, USA; 4 Department of Pathology, Memorial Sloan-Kettering Cancer Center, 1275 York Avenue, New York, NY 10065, USA; 5 Sloan-Kettering Institute, Department of Surgery, Memorial Sloan-Kettering Cancer Center, 1275 York Avenue, New York, NY 10065, USA; 6 Breast Research Laboratory, Department of Surgery, Memorial Sloan-Kettering Cancer Center, 1275 York Avenue, New York, NY 10065, USA

## Abstract

**Introduction:**

Lobular carcinoma *in situ *(LCIS) has been accepted as a marker of risk for the development of invasive breast cancer, yet modern models of breast carcinogenesis include LCIS as a precursor of low-grade carcinomas. We provide evidence favoring a clonal origin for LCIS and synchronous estrogen receptor-positive malignant lesions of the ductal and lobular phenotype.

**Methods:**

Patients with prior LCIS undergoing mastectomy were identified preoperatively from 2003 to 2008. Specimens were widely sampled, and frozen blocks were screened for LCIS and co-existing malignant lesions, and were subject to microdissection. Samples from 65 patients were hybridized to the Affymetrix SNP 6.0 array platform. Cases with both an LCIS sample and an associated ductal carcinoma *in situ *(DCIS) or invasive tumor sample were evaluated for patterns of somatic copy number changes to assess evidence of clonal relatedness.

**Results:**

LCIS was identified in 44 of the cases, and among these a DCIS and/or invasive lesion was also identified in 21 cases. A total of 17 tumor pairs had adequate DNA/array data for analysis, including nine pairs of LCIS/invasive lobular cancer, four pairs of LCIS/DCIS, and four pairs of LCIS/invasive ductal cancer. Overall, seven pairs (41%) were judged to be clonally related; in five (29%) evidence suggested clonality but was equivocal, and five (29%) were considered independent. Clonal pairs were observed with all matched lesion types and low and high histological grades. We also show anecdotal evidence of clonality between a patient-matched triplet of LCIS, DCIS, and invasive ductal cancer.

**Conclusion:**

Our results support the role of LCIS as a precursor in the development of both high-grade and low-grade ductal and lobular cancers.

## Introduction

Since the original description of lobular carcinoma *in situ *(LCIS) by Foote and Stewart in 1941 [1], confusion has existed about its management. Historical data suggest that LCIS is not an obligate precursor to invasive disease, and LCIS has until relatively recently been accepted as a risk factor for the development of invasive breast carcinoma (lifetime risk, 20 to 25%) in both the affected breast and the nonaffected breast [2-5]. Emerging laboratory findings supporting a precursor role for LCIS in the development of invasive lobular carcinoma (ILC) include the presence of shared molecular alterations in LCIS and co-existing ILC in a small number of archival specimens [6-8]. Specifically, comparative genomic hybridization studies have demonstrated losses on chromosomes 16q and 17p in both LCIS and ILC [9-11], truncating mutations in the E-cadherin gene and loss of heterozygosity (LOH) of the wild-type E-cadherin allele have been found in LCIS and adjacent ILCs [12], and studies have suggested a clonal relationship in a small number of co-existing LCIS and ILC tumors [6-8]. More recently, shared patterns of LOH were identified in a small study of LCIS and adjacent ductal lesions contributing to an emerging molecular concept that LCIS may be one of several early identifiable lesions in the pathogenesis of low-grade carcinomas [13].

Although provocative, current molecular data are limited in number and nature, largely due to the use of archival paraffin-embedded tissues. The objective of this study was to elucidate the clonal relationship between LCIS and related malignant lesions of the breast using purified cell populations from fresh frozen tissue specimens in a study of systematically collected specimens from patients undergoing mastectomy.

## Materials and methods

Patients with a documented history of LCIS undergoing risk-reducing mastectomy or therapeutic mastectomy for a new diagnosis of breast cancer at Memorial Sloan-Kettering Cancer Center, New York, USA were identified preoperatively, informed consent was obtained, and patients were prospectively enrolled on a Memorial Sloan-Kettering Cancer Center Institutional Review Board-approved protocol from 2003 to 2008. The ethics committee that approved the protocol was the Memorial Sloan-Kettering Cancer Center Institutional Review Board. Microarray data were generated in the Genomics Core Lab at Memorial Sloan-Kettering Cancer Center.

Following routine clinical sampling, up to 10 frozen blocks from each quadrant of the breast(s) were collected from the mastectomy specimens for the purposes of this study. H & E-stained sections 5 μm thick from the frozen blocks were systematically screened for LCIS, invasive carcinoma, and the presence of other high-risk lesions, including ductal carcinoma *in situ *(DCIS). Selected frozen blocks were used to prepare 10 to 15 μm thick sections for laser capture microdissection (P.A.L.M. HAL 100; Carl Zeiss Microimaging, Inc., Thornwood, NY, USA) as previously described by our group [14]. DNA was extracted (Qiagen DNAtissue Kit; Qiagen Benelux B.V., Venlo, the Netherlands) from 140 laser-capture microdissected samples representing 65 patients who consented to the prospective study, and samples were submitted to the Affymetrix SNP 6.0 microarray platform. For this analysis, we restricted attention to patients who had both an LCIS sample and an associated DCIS or invasive tumor sample.

Clinical formalin-fixed paraffin-embedded (FFPE) slides were also obtained for all cases. The histologic subtype was defined as described by the College of American Pathologists Consensus Statement [15]. Briefly, classic lobular carcinomas demonstrated low neoplastic cell density, monotonous grade 1 nuclei, low mitotic activity, and cellular dishesion, with or without targetoid infiltration, in at least 90% of the neoplastic cell population. The diagnosis of infiltrating mammary carcinoma with mixed ductal and lobular features was made in cases that had a component showing ductal differentiation (in the form of tubule formation) and areas with a lobular growth pattern (Indian file arrangement of tumor cells). These tumors typically had low nuclear grade. Invasive tumors were graded using the Scarf-Bloom and Richardson system modified by Elston and Ellis [16], and DCIS was graded according to the Van Nuys classification [17]. LCIS was classified as pleomorphic LCIS only when moderate or marked nuclear atypia was present, with or without necrosis or apocrine features [18].

Immunohistochemistry (IHC) slides were reviewed to assess the estrogen receptor (ER), progesterone receptor (PR), and HER2 status of invasive cancers (and *in situ *lesions when present in the same slide). E-cadherin slides were also reviewed when available. Fresh FFPE sections were prepared for ER, PR, and HER2 for those cases in which the *in situ *lesions (LCIS or DCIS) were not present on the original IHC slides. Additional E-cadherin staining was not performed. Care was taken to ensure that the lesions assessed by IHC were taken from the same quadrant of the breast as the lesions that were subject to the Affymetrix SNP 6.0 microarray platform. Any nuclear staining was considered positive for ER and PR. HER2 was graded according to the American Society of Clinical Oncology/College of American Pathologists guidelines [19]. The characteristics of the antibodies and protocols used in IHC are shown in Additional File 1.

The SNP arrays were processed using the aroma.affymetrix package in R to estimate raw copy numbers [20]. Briefly, the steps included normalizing the arrays to account for allelic crosstalk, base position, and fragment-length effects. Multiple probesets per SNP were then summarized for SNP-level intensity. The average intensity of all the arrays from normal tissues was used as the reference to estimate the relative copy numbers of individual arrays. The total number of probes on the array was ~1.8 million; approximately 316,000 probes that target known germline copy number variants were excluded. By averaging blocks of 100 adjacent log-ratios, we reduced the resolution of the arrays to ~15,000 markers in order to limit the noise levels. The closeness of identified paired copy number changes was examined to gauge the evidence for and against a clonal origin of the tumor pairs. Briefly, the method involves first segmenting the arrays to identify the locations of at most one copy number gain or loss on a chromosome arm [21]. Segments < 2.3 MB in length that overlapped known copy number variations from the database of genomic variants [22] were excluded. Correlation in the patterns of gains and losses between the tumors is then evaluated using the chromosome arm as the unit of analysis, which is classified based on the central or most outstanding segment if there are two or one breakpoints, respectively. Individual concordant gains or losses are then examined more carefully to assess the evidence that each concordant change could have originated from a clonal (that is, identical) somatic event. The results are aggregated, and a measure characterizing the strength of evidence favoring clonality is calculated. This measure is then benchmarked against the distribution of the measure in pairs of tumors from different patients to obtain a *P *value. Tumor pairs are considered clonal if the observed similarity measure lies outside the reference distribution of independent pairs, and is considered suggestive for clonality (equivocal) if the *P *value is within the range of the reference distribution but more extreme than the 5th percentile (that is, *P *< 0.05). Further details of the method are described in Ostrovnaya and colleagues [21], and software is available in the Bioconductor package [23].

The source data used in this study are available in Dryad Repository [doi:10.5061/dryad.6354b].

## Results

LCIS was identified in 44/65 (68%) patients subject to the array; among these, 21 patients had a paired DCIS and/or a paired invasive lesion for comparison. Seven patients were excluded due to poor-quality DNA/array data, leaving 14 patients with 17 paired samples for analysis (three patients each had three samples for comparison). The characteristics of each of the LCIS and paired samples are described in Table 1.

**Table 1:** Characteristics of lobular carcinoma *in situ *and paired lesions

			LCIS^b^				Associated lesion							
					
			IHC profile			E-cadherin	Histology	Grade^c^	IHC profile			E-cadherin		
										
Pair	Case^a^	Mastectomy	ER	PR	HER2				ER	PR	HER2		*P *value	Diagnosis^d^
LCIS and ILC	#32	Bilateral	+	+	-	NA	ILC classic	I	+	+	-	NA	0.07	I
	#43	Left	+	+	-	NA	ILC classic	I	+	+	-	NA	0.99	I
	#93	Left	+	+	-	-	ILC solid variant	II	+	+	-	-	0.004	E
	#107	Bilateral	+	+	-	-	ILC classic	I	+	+	-	-	0.013	E
	#114a	Bilateral	+	+	-	NA	ILC classic	I	+	+	-	NA	< 0.001	C
	#114b	Bilateral	+	+	-	NA	ILC classic	I	+	+	-	NA	< 0.001	C
	#121	Left	+	+	-	NA	ILC variant	II	+	+	-	NA	0.30	I
	#122	Bilateral	+	+	-	-	ILC pleomorphic	III	+	+	-	-	< 0.001	C
	#126	Right	+	+	-	NA	ILC classic	I	+	+	-	NA	0.009	E
LCIS and DCIS	#84	Bilateral	+	+	-	-	DCIS cribiform	Group 1	+	+	-	+	< 0.001	C
	#95	Left	+	+	-	NA	DCIS micropapillary	Group 3	+	+	-	NA	< 0.001	C
	#110	Bilateral	+	+	-	-	DCIS cribiform	Group 1	+	+	-	+	0.031	E
	#120	Bilateral	+	+	-	-	DCIS papillary	Group 3	-	-	-	+	0.96	I
LCIS and IDC-LF	#76	Bilateral	+	+	-	-	ILC and IDC NST	II	+	+	-	ILC-negative, IDC-positive	< 0.001	C
	#83	Left	+	+	-	-	ILC and IDC NST	I	+	+	-	-	0.12	I
LCIS and IDC	#95	Left	+	+	-	NA	IDC NST	III	+	+	-	NA	< 0.001	C
	#110	Bilateral	+	+	-	-	IDC NST and micropapillary	II	+	+	-	+	0.004	E

^a^Seventeen distinct comparisons of tumor pairs from 14 distinct patients. Case #114 involves samples from two distinct LCIS tumors (one in the upper outer quadrant and one in the lower outer quadrant) and one invasive lobular tumor. Case #76 involves samples from LCIS and the invasive lobular component of the mixed lesion. ^b^Any nuclear staining was considered positive for estrogen receptor (ER) and progesterone receptor (PR). HER2 was graded according to the American Society of Clinical Oncology/College of American Pathologists guidelines [19]. All LCIS lesions were classic LCIS, except Case #122 that was classified as pleomorphic LCIS [18]. ^c^Invasive cancers were graded according to the Scarff-Bloom and Richardson system modified by Elston and Ellis [16], and DCIS was graded according to the Van Nuys classification [17]. ^d^C, clonal (*P *< 0.001); E, equivocal (0.001 <*P *< 0.05); I, independent (*P *> 0.05). DCIS, ductal carcinoma *in situ*; IDC, invasive ductal carcinoma; IDC-LF, invasive mammary carcinoma with mixed ductal and lobular features; IHC, immunohistochemistry; ILC, invasive lobular carcinoma; LCIS, lobular carcinoma *in situ*; NA, not available; NST, no special type.

**Figure 1:**
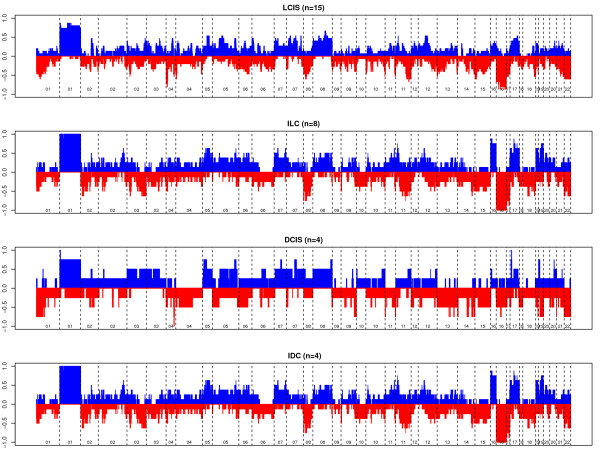
**Genome-wide histograms of allelic gains and losses**. The proportions of samples with copy number gains (blue) or losses (red) are displayed at each genetic location. DCIS, ductal carcinoma *in situ*; IDC, invasive ductal carcinoma; ILC, invasive lobular carcinoma; LCIS, lobular carcinoma *in situ*.

As a group, lesions varied in terms of the complexity of copy number changes. Among lesions of lobular origin, the most frequent changes observed were 1q gain and 16q loss. The genome-wide averages of all gains and losses in LCIS, DCIS, and invasive cancers, including partial-arm changes, are displayed in Figure 1. Overall, although the frequencies of allelic changes are slightly less in the *in situ *lesions, the general pattern of broad genetic instability is similar. The most frequently observed whole-arm gains and losses for LCIS, DCIS, and invasive lesions are ranked in Additional File 2.

**Figure 2:**
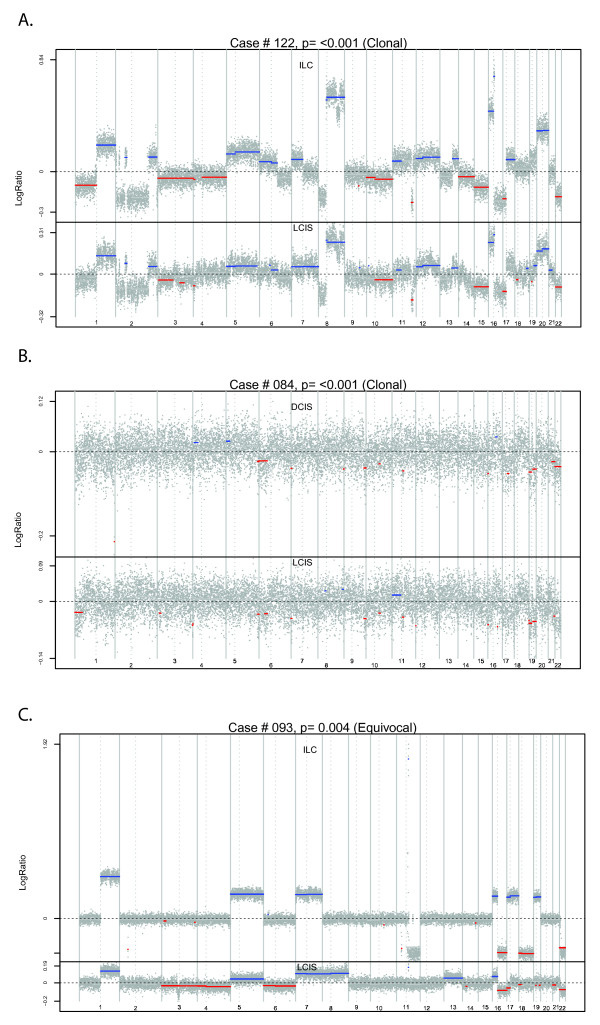
**Examples of genome-wide segmentation patterns of tumor pairs**. **(A) **Case #122. **(B) **Case #84. **(C) **Case #93. Each dot represents a log ratio; that is, allelic copy number relative to the reference normal value on log scale. In the absence of gains or losses, this should be zero. Known or suspected germline copy number variations were filtered out prior to constructing these plots. Regions of significant gain used in clonality comparison are highlighted in blue, while regions of loss are highlighted in red. DCIS, ductal carcinoma *in situ*; ILC, invasive lobular carcinoma; LCIS, lobular carcinoma *in situ*.

Of the 17 pairs evaluated, seven (41%) were classified as clonal, five (29%) as equivocal, and five (29%) as independent (Table 1). Clonal pairs were observed with all matched lesion types; that is, both ductal and lobular, and low and high histological grades. Selected examples of individual segmented arrays from three cases (Case #122, Case #84, and Case #93) are provided in Figure 2. Similar plots for all cases are provided in Additional File 3. Case #122 is an example of LCIS and paired ILC (both of pleomorphic morphology) with convincing evidence of clonality. The broad patterns of segmented changes are very similar, and there are several within-chromosome changes that are very closely matched; for example, on 2p, 8p, 11q, and 16q.

**Figure 3:**
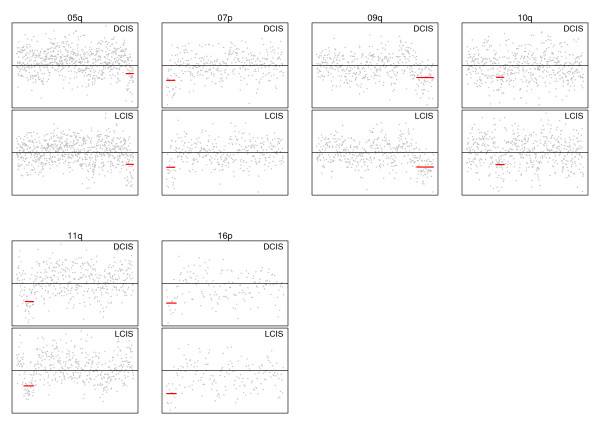
**Detailed plots of examples of matching allelic gains and losses in Case #84**. Copy number plots specific to the chromosome arm are provided for examples of probable clonal allelic changes. Details of all arms for all comparisons are provided in Additional File 4. DCIS, ductal carcinoma *in situ*; LCIS, lobular carcinoma *in situ*.

Case #84 is an example of paired LCIS and cribriform low-grade DCIS where the immediate visible evidence is much less clear cut in that allelic changes are not easily visible due to the noise in the arrays. However, this is an example where the statistical algorithm is especially useful. The within-chromosome-arm segmentation method detects closely matching changes on several chromosome arms; that is, 5q, 7p, 9q, 10q, 11q, and 16p. These matches can be observed more clearly by examining individual chromosome arms. Figure 3 displays these changes, clearly showing the closeness of the matching gains and losses. Chromosome-specific plots are provided for all cases in Additional File 4.

It is harder to find convincing evidence that tumor pairs are independent, since we have to be confident that we have not failed to detect true clonal changes. Case #93 involving LCIS and an ILC solid variant has the ubiquitous matching whole-arm gains on 1q and losses on 16q, and a few other matching whole-arm changes (Figure 2), but there are many nonmatching changes and no concordant gains or losses within an individual chromosome arm. Consequently, in this case there is no strong evidence that the tumors are clonal. For all five cases classified as independent (Case #32, Case #43, Case #121, Case #120, and Case #83), an examination of the detailed arm-by-arm changes in Additional File 4 shows that where evidence of allelic changes is detected they are rarely overlapping for the tumors being compared. Representative photomicrographs of Case #122 (paired pleomorphic LCIS and ILC determined to be of clonal origin) and Case #93 (paired classic LCIS and ILC determined to be of equivocal origin) are provided in Additional File 5.

**Figure 4:**
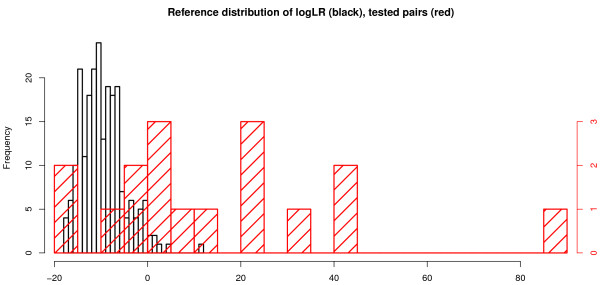
**Histogram of measures of similarity**. Plot of the distribution of our measure of clonality for the individual comparisons of tumor pairs (red histogram). The black reference histogram is obtained by comparing all pairs of tumors from different patients. Since these pairs are definitively nonclonal they provide the reference distribution, which is used to permit calculation of the *P *values of the real pairings. The scale of the horizontal axis is the log-likelihood ratio (logLR), our measure of evidence in favor of clonality. The vertical axes represent the frequencies of pairs in the samples: left axis, black (reference) histogram; right axis, red (sample) histogram.

The measures representing the strength of evidence for clonality from the individual comparisons are plotted in the red histogram in Figure 4. The black histogram represents a reference distribution of measures obtained from analyzing pairs of tumors from different patients. The seven definitively clonal cases comprise the entries in the red histogram that lie clearly to the right of the entire black histogram, and these are identified by their case numbers below the plot.

**Figure 5:**
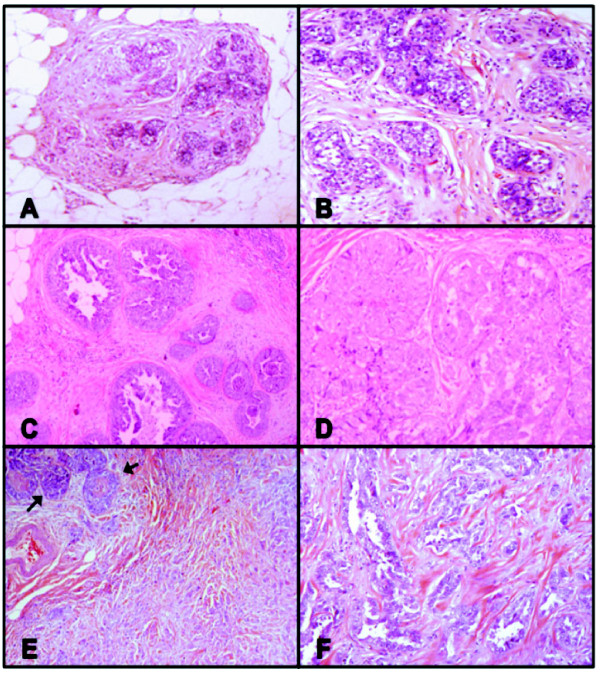
**Representative photomicrographs: Case #95, lobular carcinoma *in situ*-ductal carcinoma *in situ*-invasive cancer triplet**. **(A), (B) **Terminal duct lobular units involved by classic lobular carcinoma *in situ *(LCIS): (A) H & E, 40×; (B) H & E, 100×. **(C), (D) **High-grade ductal carcinoma *in situ *(DCIS) with solid and micropapillary patterns: (C) H & E, 40×; (D) H & E, 100×. **(E), (F) **Focus of high-grade DCIS (arrows) and associated invasive ductal carcinoma of no special type of moderate tubule formation and intermediate nuclear grade - histologic grade II/III: (E) H & E, 40×; (F) H & E, 100×.

**Figure 6:**
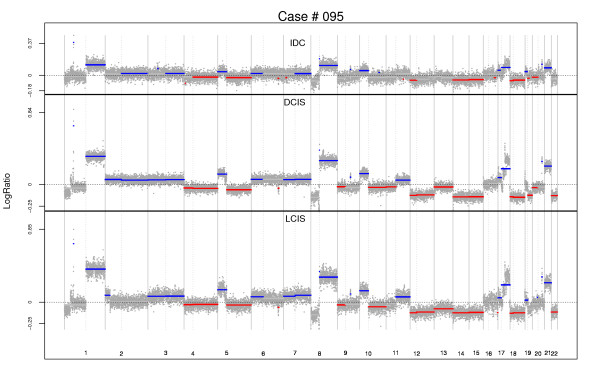
**Genome-wide segmentation: Case #95, lobular carcinoma *in situ*-ductal carcinoma *in situ*-invasive cancer triplet**. Regions of significant gain used in clonality comparison are highlighted in blue, while regions of loss are highlighted in red. DCIS, ductal carcinoma *in situ*; IDC, invasive ductal carcinoma; LCIS, lobular carcinoma *in situ*.

As expected, all LCIS, classic ILCs, and low-grade invasive ductal carcinoma (IDC) were ER-positive and HER2-negative. All but one of the high-grade lesions (*in situ *or invasive) included in this study were also ER-positive and HER2-negative. The only exception was a solid high-grade DCIS with a triple-negative profile (Case #120). Interestingly, the triple-negative case was considered to be of independent origin from the adjacent ER-positive LCIS; while two ER-positive DCIS cases and two ER-positive IDCs, both low-grade and high-grade histologies, were considered to be of clonal origin with the adjacent LCIS. In addition, Case #95 involved three tumor samples (LCIS, DCIS, and IDC), all of which displayed the same IHC profile and all of which were determined to be clonal. The photomicrographs of the individual lesions for Case #95 and the respective genome-wide plots are displayed in Figures 5 and 6, respectively. In these tumors, the broad similarity of profiles is evident from the plots, although the matching changes are mostly whole-arm changes.

**Figure 7:**
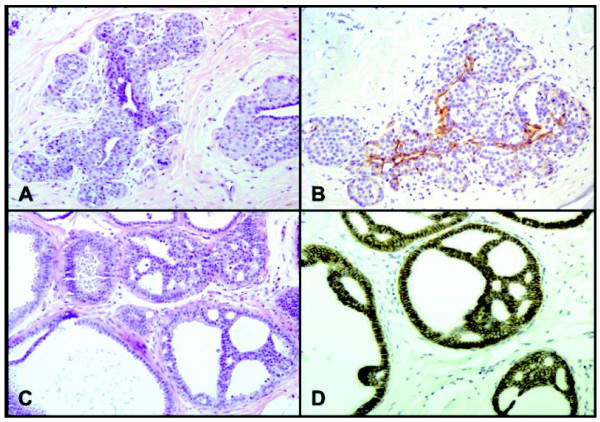
**Case #84: classic lobular carcinoma *in situ *and associated low-grade ductal carcinoma *in situ***. **(A) **Terminal duct lobular units expanded by classic lobular carcinoma *in situ *(LCIS; H & E, 200×). **(B) **Lack of membranous E-cadherin immunoreactivity on LCIS cells. The residual luminal cells show strong membrane staining (E-cadherin, 100×). **(C) **Low-grade ductal carcinoma *in situ *(DCIS) showing a prominent cribriform pattern (H & E, 100×). **(D) **Strong, uniform membrane staining with E-cadherin on DCIS cells (E-cadherin, 100×).

**Figure 8:**
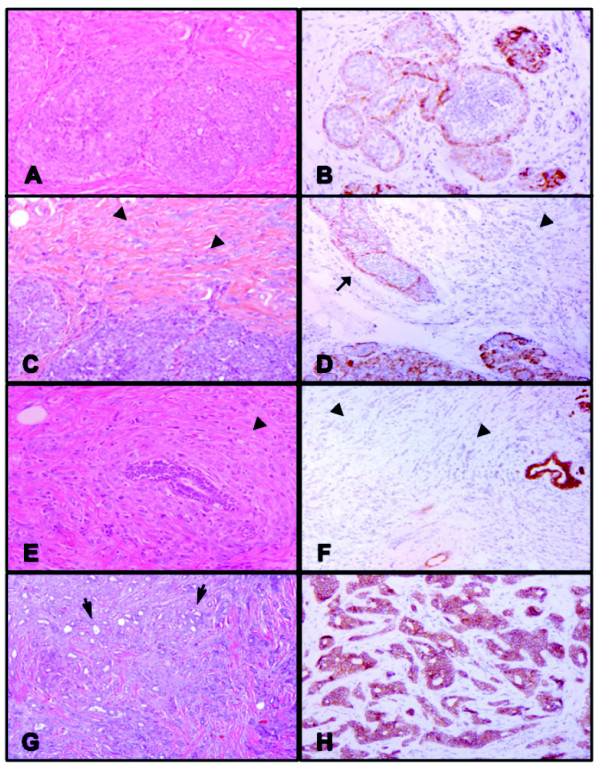
**Case #76: lobular carcinoma *in situ *and associated invasive mammary carcinoma with mixed components**. **(A), (B) **Classic lobular carcinoma *in situ *(LCIS) with no membranous reactivity for E-cadherin. Residual luminal cells show membrane staining: (A) H & E, 100×; (B) E-cadherin, 100×. **(C), (D) **Focus of LCIS (arrows) and lobular component of the invasive mammary carcinoma (arrowheads) with no E-cadherin immunoreactivity: (C) H & E, 100×; (D) E-cadherin, 100×. **(E), (F) **E-cadherin-negative invasive mammary carcinoma cells (arrowheads) surrounding a normal duct concentrically in a targetoid pattern: (E) H & E, 100×; (F) E-cadherin, 40×. **(G), (H) **Ductal component of the invasive mammary carcinoma showing tubule formation (arrows) and strong, uniform membrane staining with E-cadherin: (G) H & E, 40×; (H) E-cadherin, 100×.

Although we did not perform E-cadherin immunochemistry systematically in these cases, for the purposes of this study clinical slides were available for 9/17 (53%) pairs (Table 1). In all available cases, E-cadherin staining was characteristically absent in lobular lesions and present in ductal lesions - including Case #84, in which the LCIS and adjacent DCIS were considered to be of clonal origin as described above in Figure 2. Representative photomicrographs of Case #84 are shown in Figure 7. Case #76, a comparison of LCIS and the invasive lobular component of the mixed invasive lesion (IDC-LF), also considered to be of clonal origin, similarly demonstrated absent E-cadherin staining in both LCIS and the lobular component of the invasive lesion, while E-cadherin staining was present in the ductal component (Figure 8).

Finally, data are publically available from an important previous study by Hwang and colleagues that examined 24 pairs of synchronous LCIS and ipsilateral ILCs microdissected from FFPE tissue using a bacterial artificial chromosome array with roughly 1,900 clones covering the whole genome [7]. We re-analyzed these 24 tumor pairs using the same statistical methodology as in our analyses. We classified six of the 24 tumor pairs (25%) as clonal, seven (29%) as equivocal, and 11 (46%) as independent - results broadly consistent with the interpretations of Hwang and colleagues.

## Discussion

Once a woman is diagnosed with LCIS, she faces an eightfold to 10-fold increased risk for the subsequent development of breast cancer, the pathogenesis of which is poorly understood [24,25]. Emerging reports of shared molecular alterations between LCIS and adjacent invasive lesions in parallel with genomic evidence that LCIS may be one of several early identifiable lesions in the pathogenesis of low-grade cancer have re-opened the debate regarding the true significance of LCIS and its precursor potential. Using fresh frozen breast samples subject to laser-capture microdissection for isolation of pure cell populations, and using specialized statistical methods, we report evidence that LCIS is clonally related to a substantial proportion of the adjacent malignancies studied in this series, including cases of low-grade and high-grade DCIS and invasive mammary carcinoma with mixed ductal and lobular features. In addition, the immunohistochemical profiles of all cases considered clonal or equivocal were consistent with the recent theory of breast carcinogenesis whereby LCIS and ER-positive DCIS are grouped as precursors of ER-positive invasive cancer, and ER-negative DCIS is a precursor of ER-negative invasive cancer, regardless of histologic grade [26].

As LCIS is usually a small, incidentally detected lesion, most prior reports assessing clonality between LCIS and adjacent malignancies were based on FFPE samples. These studies also suggest clonality between LCIS and adjacent breast carcinomas, despite the fact that they differed in terms of methodology [6,7,27,28]. Our re-analysis of the publically available data from the study by Hwang and colleagues [7] using our own statistical methods provided classification results broadly consistent with our own results. The use of fresh frozen tissue samples and SNP arrays in our study allows for a substantially increased resolution for assessing clonal relatedness.

Wagner and colleagues analyzed LOH in 10 cases of co-existent ipsilateral DCIS, LCIS, and invasive carcinoma [27]. LOH was investigated in 13 commonly informative and deleted markers on chromosome 16 (six markers), chromosome 17 (two markers), and chromosomes 1, 8, 9, 11, and 13 (one marker each). In five cases, the authors observed phenotype concordance among all three samples (LCIS, DCIS, and IDC) in at least one marker. In two additional cases, concordant LOH was found between LCIS and DCIS, but not between LCIS and the invasive lesion. This study was novel in suggesting evidence of clonality between LCIS and lesions with ductal phenotype, and our own results provide substantive support for this conclusion. We found definitive or probable clonality in three out of four LCIS-DCIS pairs and among all three lesions in two LCIS-DCIS-invasive cancer case triplets (Case #95 and Case #110). All clonal pairs displayed concordant ER-positivity between paired lesions, yet the subsequent lesions were not restricted to low-grade disease. This finding is consistent with our previous work demonstrating a prominent role for ER status over grade in breast cancer progression in a historical cohort of women with *in situ *and subsequent invasive lesions [29], and adds to the growing body of literature that suggests low-grade and high-grade ER-positive tumors are more similar to each other than to their ER-negative counterparts [26].

Our results also suggest that LCIS and DCIS can have the same cell of origin and therefore may be part of a morphological spectrum of the same precursor lesion. Although pathologists often use E-cadherin staining to distinguish between these two lesions, IHC has many well-described pitfalls [30-35] and many pathologists prefer to differentiate these lesions based on morphology alone. If newly proposed models of breast carcinogenesis are validated, whereby both ER-positive LCIS and DCIS behave as precursor lesions to low-grade ER-positive breast cancer, this distinction may become less clinically relevant.

While results to date provide broad support for clonality among synchronous lobular lesions, the data favoring clonality among LCIS and subsequent (metachronous) invasive cancers are less clear. Aulmann and colleagues studied nine patients with LCIS who developed subsequent invasive breast carcinoma (five ILC cases and four IDC cases) between 2 and 10 years after the index biopsy [6]. All cases (LCIS and invasive) showed an ER-positive/HER2-negative profile. This study was based on comparisons of mitochondrial DNA heteroplasmy by PCR, direct sequencing, and phylogenetic tree clustering, and used microdissected samples from FFPE tissue. They observed identical patterns of heteroplasmy in two out of five pairs of LCIS and ILC. In one case the changes were more complex in ILC than in LCIS, and in two pairs of LCIS and ILC the changes favored unrelatedness. Similarly, in all four pairs of LCIS and subsequent IDC, the changes favored unrelatedness. More work examining metachronous cancers is needed to better understand the clonal relationship between primary cancers and subsequent recurrences.

Despite the extensive data available to us from copy number profiling, the classification of cases as clonal versus independent is far from a litmus test. Ideally, the histogram plot of our likelihood ratio measure (Figure 4, red histogram) would separate clearly into two distinct groups: one group overlapping the reference (black histogram) distribution, representing the independent pairs; and one group clearly separated, representing the clonal pairs. We do see a clear separation for some pairs that can be confidently classified as clonal. However, the histogram also includes several tumor pairs in an intermediate grey zone at the upper tail of the reference distribution (*P *< 0.05) but within the range of values obtained by pairing tumors from different patients. This phenomenon could have various causes. Tumor evolution following clonal divergence would tend to lead to a mixed pattern of matching and nonmatching allelic changes. This concept has been hypothesized to account for the wide range of histologic and molecular diversity seen within many ductal *in situ *lesions and may also explain the proposed evolution from low-grade to high-grade disease among ER-positive lesions [36,37]. Alternatively, contamination of the tumor samples with normal cells or technical artifacts will tend to obscure true signals, making it harder for our statistical algorithm to detect them clearly. The algorithm is especially useful in assessing the evidence for and against clonal relatedness in these difficult cases.

Others have attempted to determine clonality based on the presence of concordant mutations, yet this methodology can be limited by the fact that sporadic mutations happen recurrently at the same point in some tumors. For example, the BRAF point mutation (T1799A) occurs in 45% of papillary thyroid carcinomas and is associated with poor clinical outcome, but its high frequency limits its usefulness to address clonality [38]. In contrast, inactivating mutations of the E-cadherin gene that occur at dozens of different locations within the gene are highly frequent in infiltrating lobular breast carcinomas and in diffuse gastric carcinomas. The specificity of the mutations makes this gene much more informative regarding clonal relatedness. These mutations can be small insertions or deletions and are frequently combined with LOH of the wild-type allele [39-42]. Mutations in E-cadherin have been found at very early non-invasive stages of these diseases, leading to an association between E-cadherin mutations and loss of growth control, and to the classification of E-cadherin as a candidate tumor suppressor. Data regarding the presence of coincident mutations in E-cadherin among LCIS and adjacent invasive cancers have been mixed. In an early report, Vos and colleagues presented two LCIS-ILC pairs with matching point mutations [12]. Rieger-Christ and colleagues, however, found no matches in a series of eight patients in which mutations were detected in LCIS-invasive pairs [42]. Germline mutations of the E-cadherin gene have been described in families with hereditary diffuse gastric carcinomas, and family members are also at increased risk for invasive lobular cancers; however, germline mutations have not been identified among women with LCIS outside these kindreds [39,43-45].

Our statistical method compares all areas of gains and losses for a pair of samples, examines the concordance of the starting and ending points within chromosomes, and compares the degree of similarity of changes of an individual pair to a reference distribution created with samples paired from different patients. This provides a stronger argument for clonality than any single concordant point mutation. The use of an empirical reference distribution created using pairs of tumors from different patients is especially important since we observed some similar patterns of copy number variation between different patients. For example, the presence of 1q gain and 16q loss, consistently reported in low-grade lesions of ductal and lobular morphology, was present in 73% and 53% of our LCIS cases, respectively, and therefore these changes are not very meaningful in an analysis of clonal relatedness.

We recruited all women in a defined period presenting for risk-reducing or therapeutic mastectomy, but we only aimed to harvest fresh frozen LCIS for DNA extraction in a proportion of them; as a result, this group may not be representative of all women harboring LCIS. While this small prospective study confirms to us that LCIS is likely to be associated with ER-positive and low-grade disease, the lack of a larger group with ER-negative and more high-grade histology prevents us from drawing broad conclusions on the characteristics of clonally related lesions.

## Conclusions

In summary, high-resolution genome mapping using fresh frozen microdissected samples suggests clonality between classic LCIS and a substantial proportion of adjacent malignant synchronous lesions of lobular and ductal phenotype. Both low-grade and high-grade DCIS and invasive lesions showed high degrees of similarity with patient-matched LCIS. The ER status was concordant in all lesions considered to be clonal or equivocal. These data support the recent theory of breast carcinogenesis where LCIS and ER-positive DCIS are grouped as precursors of ER-positive invasive cancer, and where ER-negative DCIS is a precursor of ER-negative invasive cancer, regardless of histologic grade.

## Abbreviations

DCIS: ductal carcinoma *in situ*; ER: estrogen receptor; FFPE: formalin-fixed paraffin-embedded; H & E: hematoxylin and eosin; HER2: human epidermal growth factor receptor 2; IDC: invasive ductal carcinoma; IHC: immunohistochemistry; ILC: invasive lobular carcinoma; LCIS: lobular carcinoma *in situ*; LOH: loss of heterozygosity; PCR: polymerase chain reaction; PR: progesterone receptor; SNP: single nucleotide polymorphism.

## Competing interests

The authors declare that they have no competing interests.

## Authors' contributions

The authors confirm that they all made substantial contributions to conception and design, acquisition of data, or analysis and interpretation of data, they have all been involved in drafting the manuscript or revising it critically for important intellectual content, and they have all given final approval of the version to be published. VPA, MMorrogh, DG, NO, and TK participated in the acquisition of fresh frozen tissue samples, laser capture microdissection, and DNA extraction. VPA, IO, VES, CBB, DG, MDB, and TK drafted the manuscript. MDB, VPA, and DG participated in the review of clinical slides and figure preparation. VPA, IO, VES, MMorrogh, MDB, MMorrow, CBB, and TK participated in the design and coordination of the study. IO, VES, and CBB performed the statistical analysis. VPA, IO, VES, MMorrogh, CBB, and TK conceived of the study. All authors read and approved the final version submitted for publication.

## Supplementary Material

Additional file 1 **Supplemental Table 1 presenting characteristics of antibodies and protocols used in immunohistochemistry**. Click here for file

Additional file 2 **Supplemental Table 2 presenting frequencies of whole-arm gains/losses**. Table shows the percentages of tumors for which the segmentation algorithm identified a whole-arm gain or loss. Click here for file

Additional file 3 **Genome-wide plots, analogous to Figure **2. Click here for file

Additional file 4 **Magnified version of genome-wide plots with detailed marker plots and segmentation on a chromosome-arm-specific basis**. Click here for file

Additional file 5 **Representative photomicrographs of Case #122 and Case #93**. Case #122: LCIS and paired invasive lobular carcinoma of pleomorphic morphology. (A), (B) LCIS of pleomorphic morphology with no E-cadherin immunoreactivity. Residual luminal cells show membrane staining: (A) H & E, 40×; (B) E-cadherin, 100×. (C), (D) Focus of LCIS (arrows) and associated invasive lobular carcinoma (ILC, arrowheads) both of pleomorphic morphology showing lack of membranous reactivity for E-cadherin: (C) H & E, 40×; (D) E-cadherin, 100×. (E), (F) ILC pleomorphic variant with no positivity with E-cadherin staining as opposed to a normal duct. The inset image at 400× magnification illustrates tumor cells with abundant eosinophilic cytoplasm and high nuclear grade in a single-file infiltrating pattern: (E) H & E, 40×; (F) E-cadherin, 40×. Case #93: LCIS and associated invasive lobular carcinoma with no immunoreactivity for E-cadherin. (A) Classic LCIS and invasive lobular carcinoma (ILC) surrounding a normal duct (H & E, 40×). (B) E-cadherin staining shows lack of membranous reactivity in LCIS and ILC as opposed to the adjacent normal terminal duct (E-cadherin, 40×). Click here for file
