# Supplementary material for: Clonal relatedness between lobular carcinoma in situ and synchronous malignant lesions
Source: Breast Cancer Res. 2012 Jul 9;14(4):R103. doi: 10.1186/bcr3222 (PMC3680923; doi:10.1186/bcr3222)
Supplement: Additional file 1 — Supplemental Table 1 presenting characteristics of antibodies and protocols used in immunohistochemistry. [file bcr3222-S1.DOC]

Additional File No. 1.

Supplemental Table 1. Characteristics of antibodies and protocols used in immunohistochemistry.

| **Antibody** | **Origin** | **Clone** | **Dilution** | **Incubation** | **Retrieval** | **Detection** | **Staining**  **location** | **Scoring** |
| --- | --- | --- | --- | --- | --- | --- | --- | --- |
| **ER** | Dako | 1D5 | 1:100 | 30mn | EDTA pH9 | Horse radish peroxidase polymer | Nuclear | Any positivity |
| **PR** | Dako | PgR636 | 1:100 | 30mn | Citrate pH6.0 | Horse radish peroxidase polymer | Nuclear | Any positivity |
| **HER2** | Dako | Rabbit | as per kit | 30mn | EDTA pH9 | Horse radish peroxidase polymer | Membranous | Herceptest |
| **Ecad** | Dako | NCH-38 | 1:50 | 30mn | Citrate pH6.0 | Horse radish peroxidase polymer | Membranous | Any positivity |
