# Supplementary material for: Clonal relatedness between lobular carcinoma in situ and synchronous malignant lesions
Source: Breast Cancer Res. 2012 Jul 9;14(4):R103. doi: 10.1186/bcr3222 (PMC3680923; doi:10.1186/bcr3222)
Supplement: Additional file 2 — Supplemental Table 2 presenting frequencies of whole-arm gains/losses. Table shows the percentages of tumors for which the segmentation algorithm identified a whole-arm gain or loss. [file bcr3222-S2.DOCX]

Supplemental Table 2. Frequencies of whole-arm gains/losses^1^.

|  | LCIS  (n = 15) | ILC  (n = 8) | DCIS  (n = 4) | IDC  (n = 4) |
| --- | --- | --- | --- | --- |
| 01q Gain | 73% | 100% | 75% | 100% |
| 16q Loss | 53% | 88% | 50% | 50% |
| 22q Loss | 40% | 38% | 75% | 50% |
| 18p Loss | 20% | 25% | 50% | 75% |
| 08q Gain | 40% | 25% | 50% | 50% |
| 17p Loss | 40% | 63% | 25% | 25% |
| 16p Gain | 27% | 63% | 0% | 50% |
| 06p Loss | 20% | 13% | 25% | 50% |
| 08p Loss | 20% | 13% | 25% | 50% |
| 15q Loss | 20% | 38% | 25% | 25% |
| 20p Gain | 7% | 25% | 25% | 50% |
| 07q Gain | 13% | 13% | 25% | 50% |
| 19p Loss | 20% | 25% | 25% | 25% |
| 07p Gain | 13% | 25% | 25% | 25% |
| 17q Gain | 13% | 50% | 0% | 25% |

^1^ The table shows the percentages of tumors for which the segmentation algorithm identified a whole-arm gain or loss.
