# Supplementary material for: Clonal relatedness between lobular carcinoma in situ and synchronous malignant lesions
Source: Breast Cancer Res. 2012 Jul 9;14(4):R103. doi: 10.1186/bcr3222 (PMC3680923; doi:10.1186/bcr3222)
Supplement: Additional file 3 — Genome-wide plots, analogous to Figure 2. [file bcr3222-S3.ZIP › genomewideplots revision.pdf]

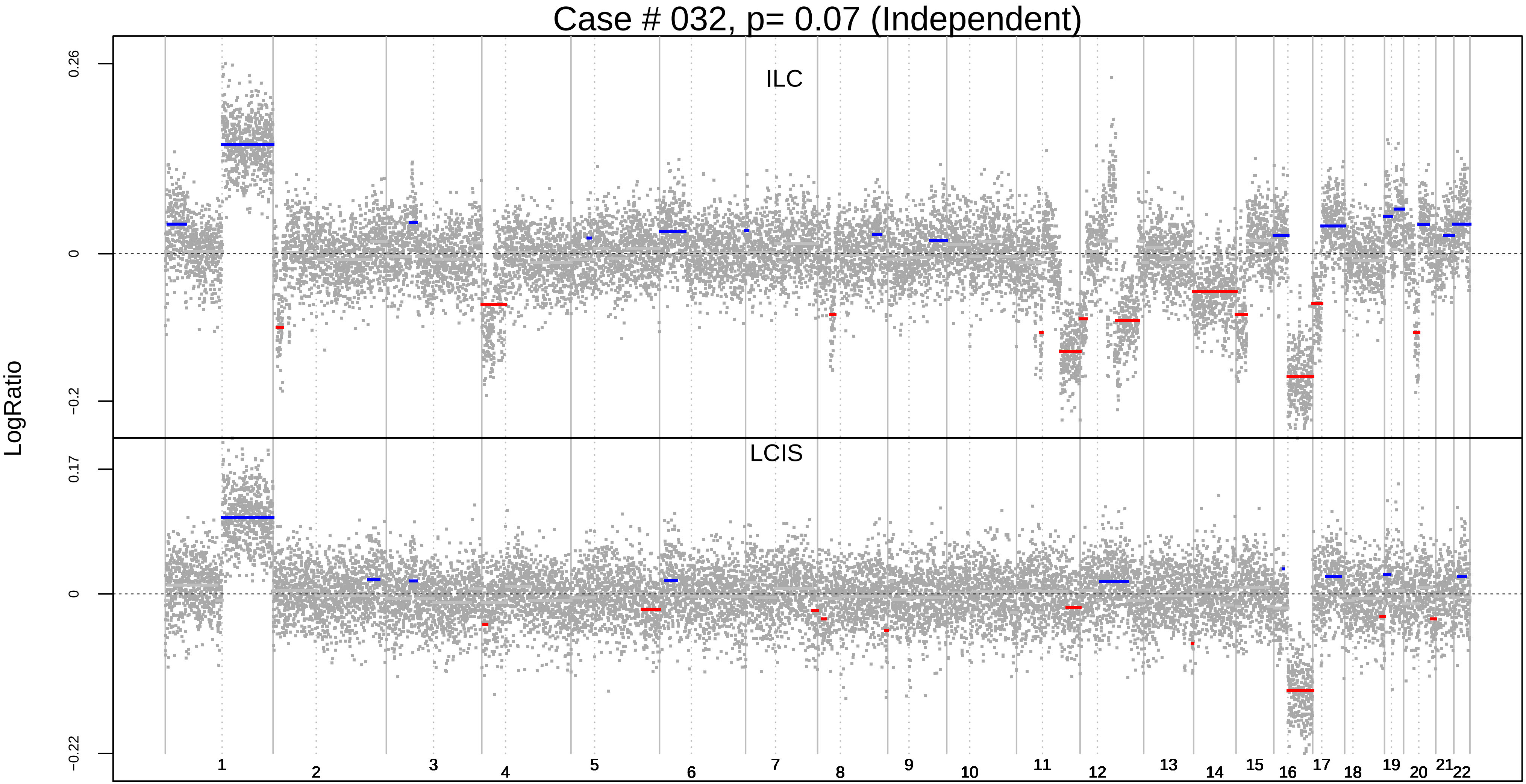

Case # 032, p= 0.07 (Independent)
ILC
LCIS
LogRatio
0.26
0
−0.2
0.17
0
−0.22
1
2
3
4
5
6
7
8
9
10
11
12
13
14
15
16
17
18
19
20
21
22

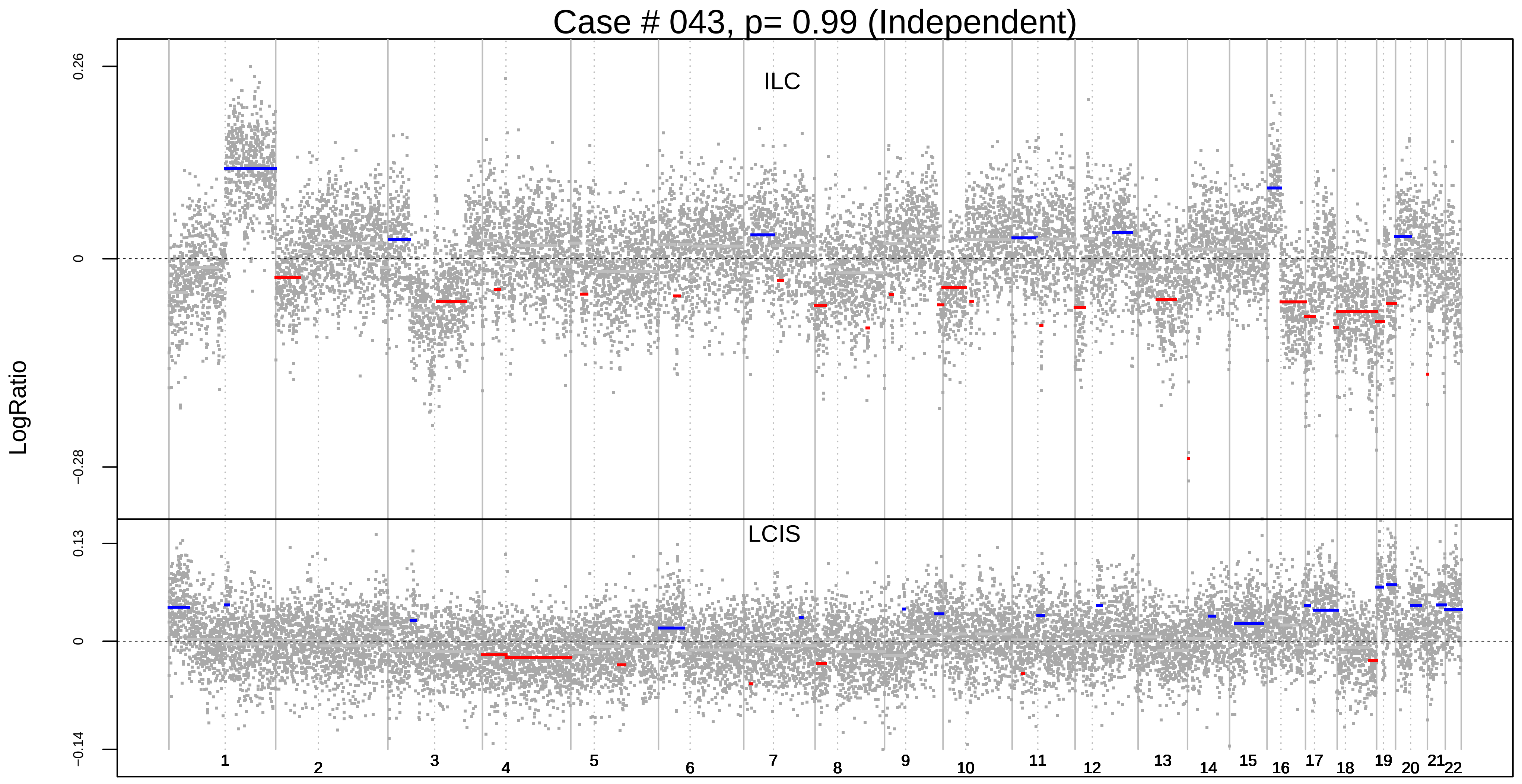

Case # 043, p= 0.99 (Independent)
ILC
LCIS
LogRatio
0.26
0
−0.28
0.13
0
−0.14
1
2
3
4
5
6
7
8
9
10
11
12
13
14
15
16
17
18
19
20
21
22

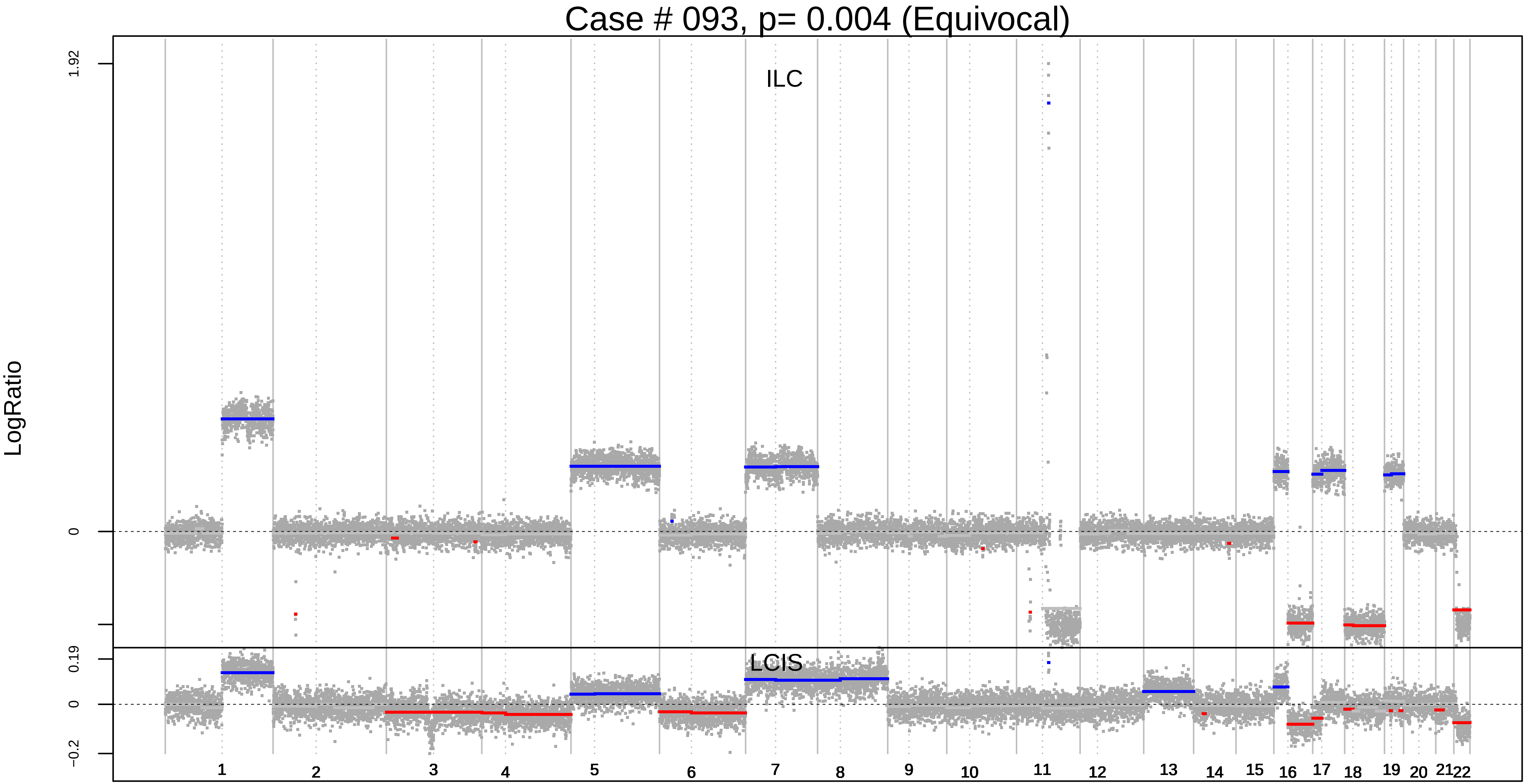

Case # 093, p= 0.004 (Equivocal)
LogRatio
1.92
0
0.19
0
−0.2
ILC
LCIS
1
2
3
4
5
6
7
8
9
10
11
12
13
14
15
16
17
18
19
20
21
22

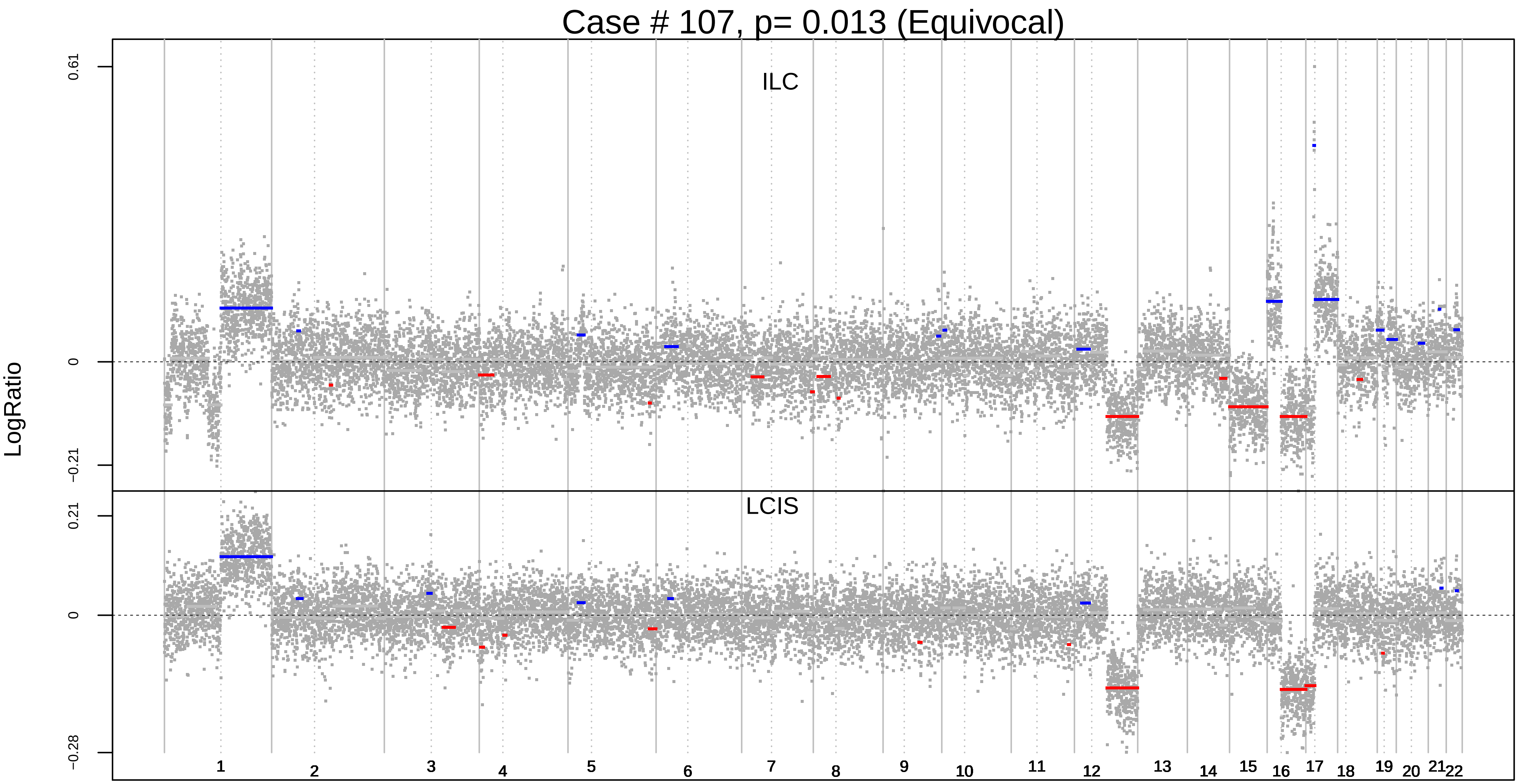
Case # 107, p= 0.013 (Equivocal)
ILC
LCIS
LogRatio
0.61
0
−0.21
0.21
0
−0.28
1
2
3
4
5
6
7
8
9
10
11
12
13
14
15
16
17
18
19
20
21
22

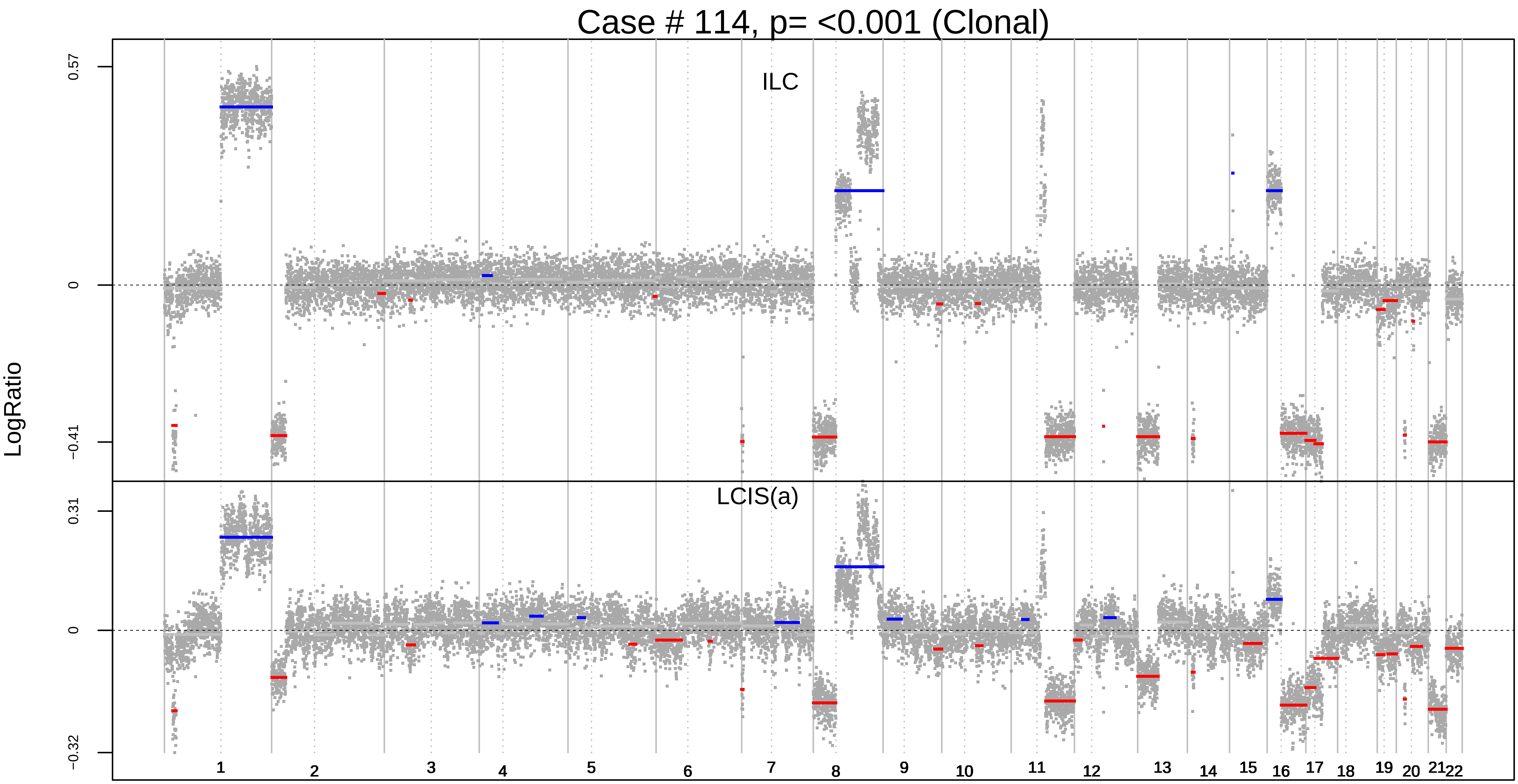

Case # 114, p= <0.001 (Clonal)
LogRatio
ILC
LCIS(a)
0.57
0
−0.41
0.31
0
−0.32
1
2
3
4
5
6
7
8
9
10
11
12
13
14
15
16
17
18
19
20
21
22

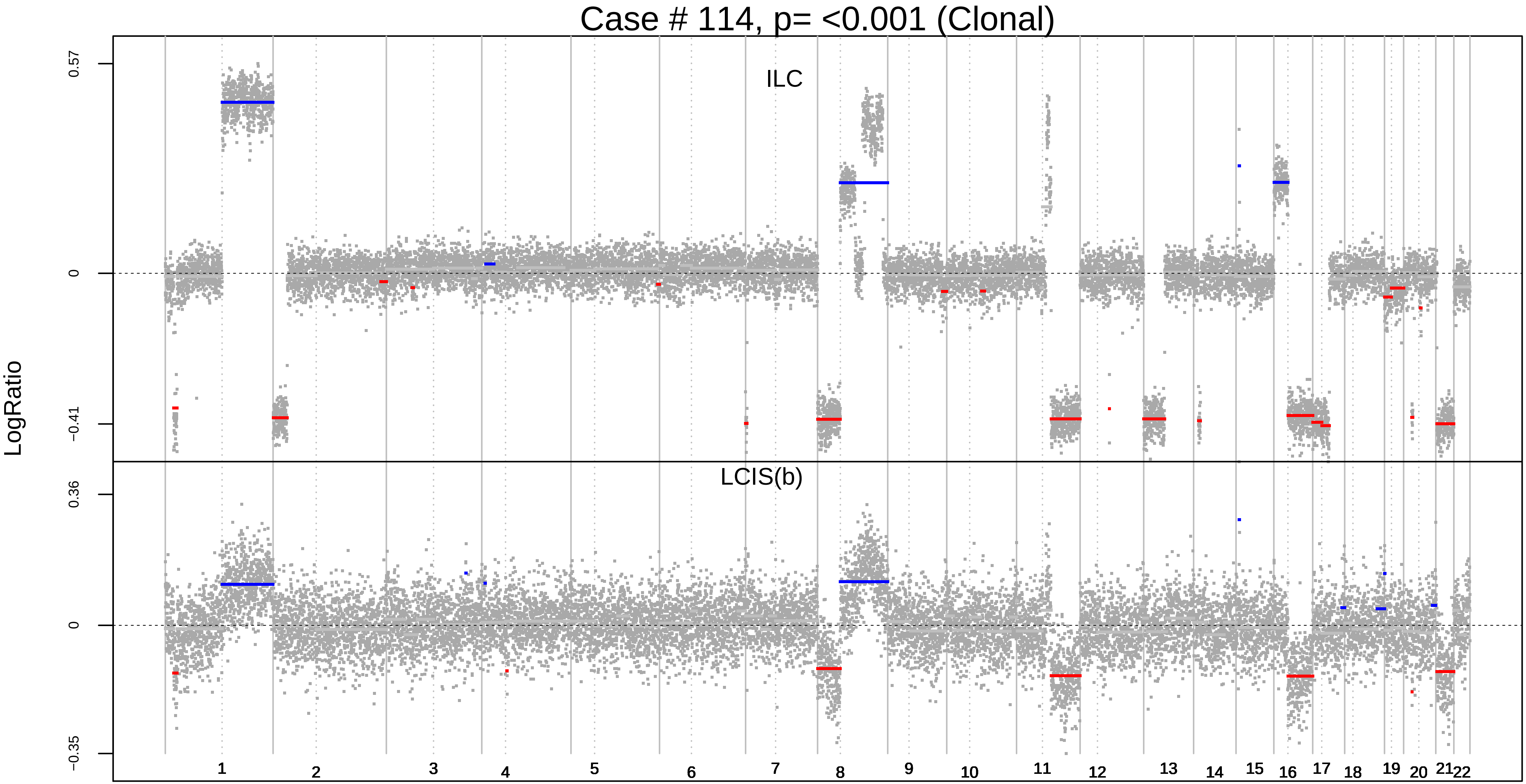

Case # 114, p= <0.001 (Clonal)
ILC
LCIS(b)
LogRatio
0.57
0
−0.41
0.36
0
−0.35
1
2
3
4
5
6
7
8
9
10
11
12
13
14
15
16
17
18
19
20
21
22

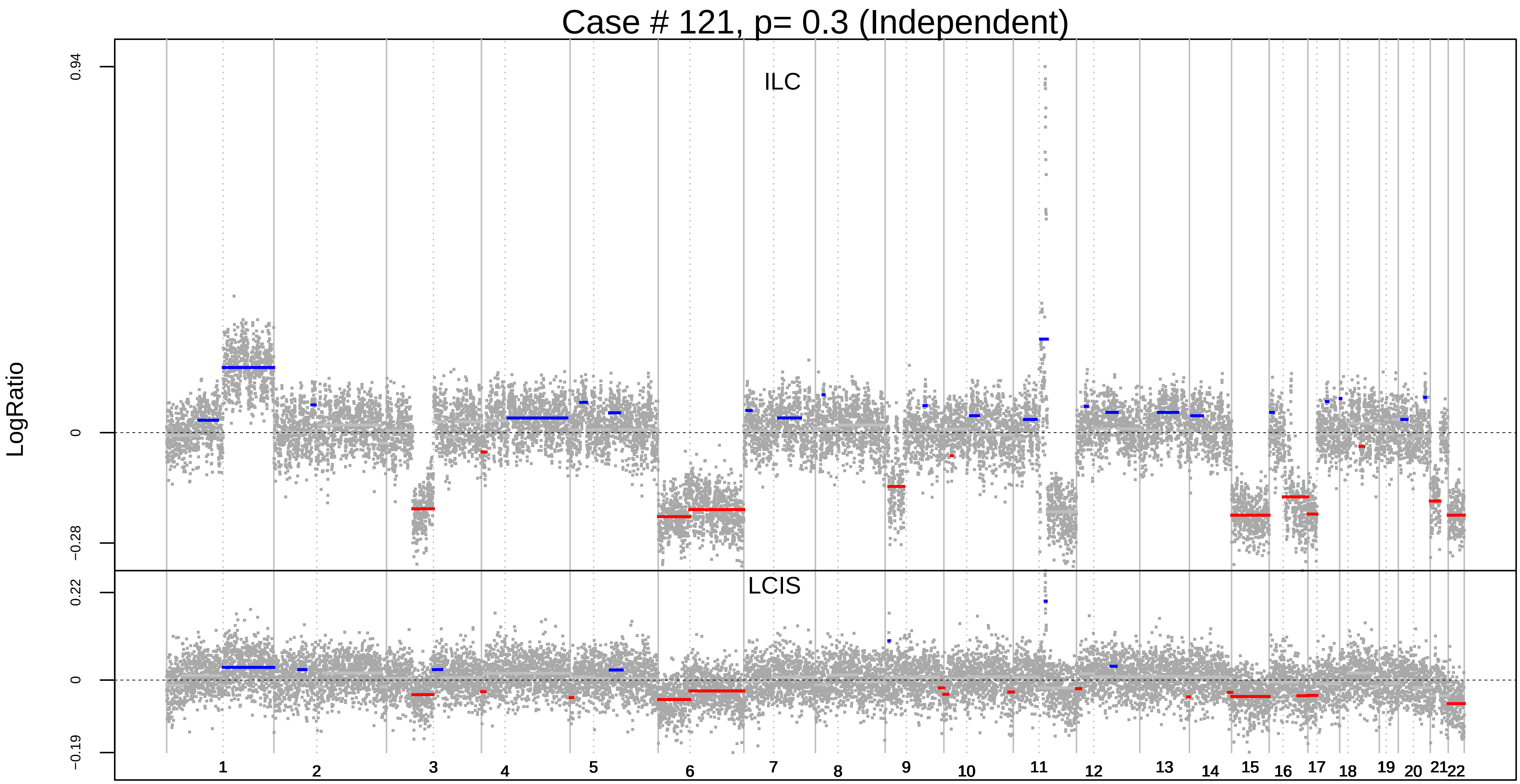
Case # 121, p= 0.3 (Independent)
ILC
LCIS
LogRatio
0.94
0
−0.28
0.22
0
−0.19
1
2
3
4
5
6
7
8
9
10
11
12
13
14
15
16
17
18
19
20
21
22

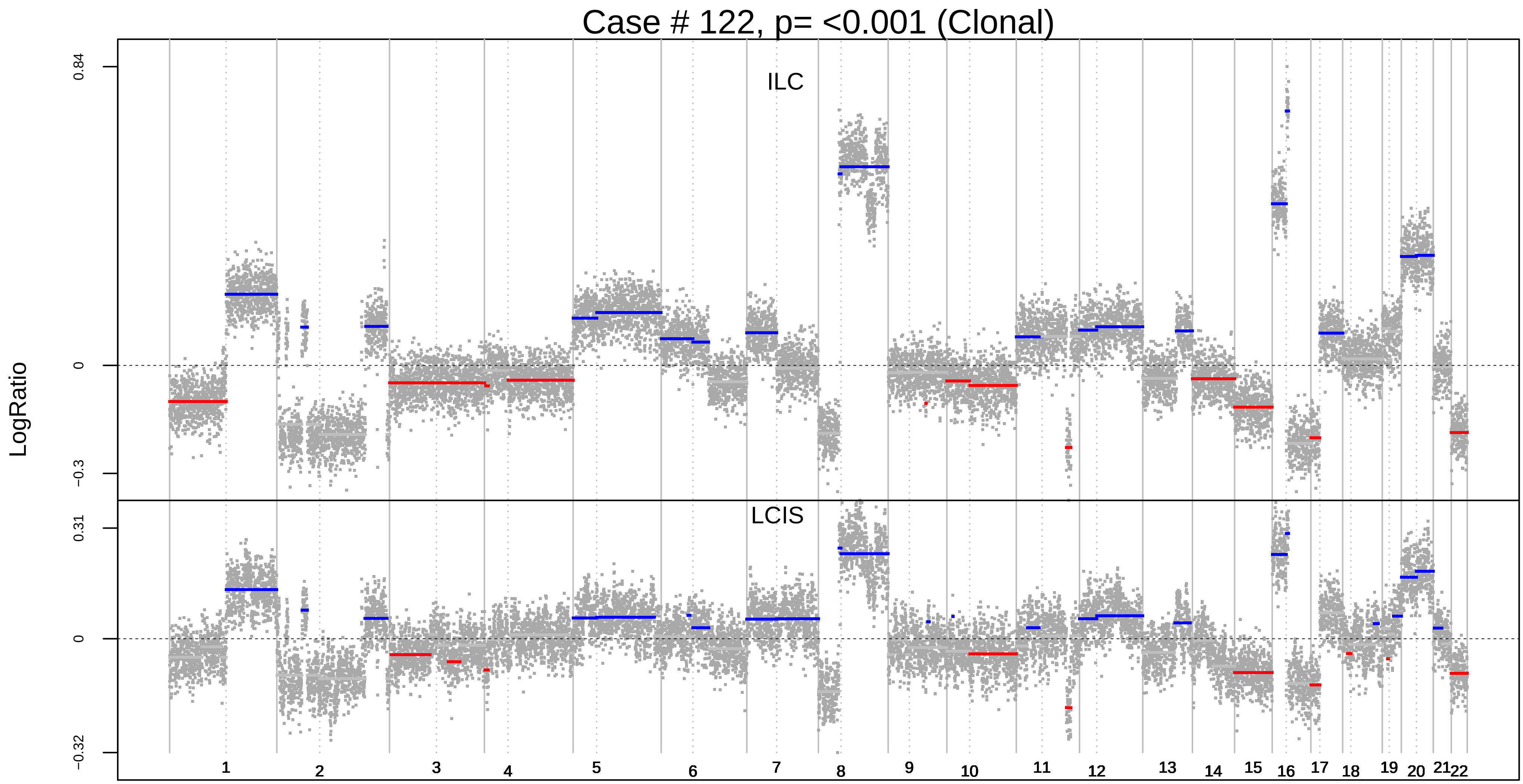
Case # 122, p= <0.001 (Clonal)
ILC
LCIS
LogRatio
0.84
0
−0.3
0.31
0
−0.32
1
2
3
4
5
6
7
8
9
10
11
12
13
14
15
16
17
18
19
20
21
22

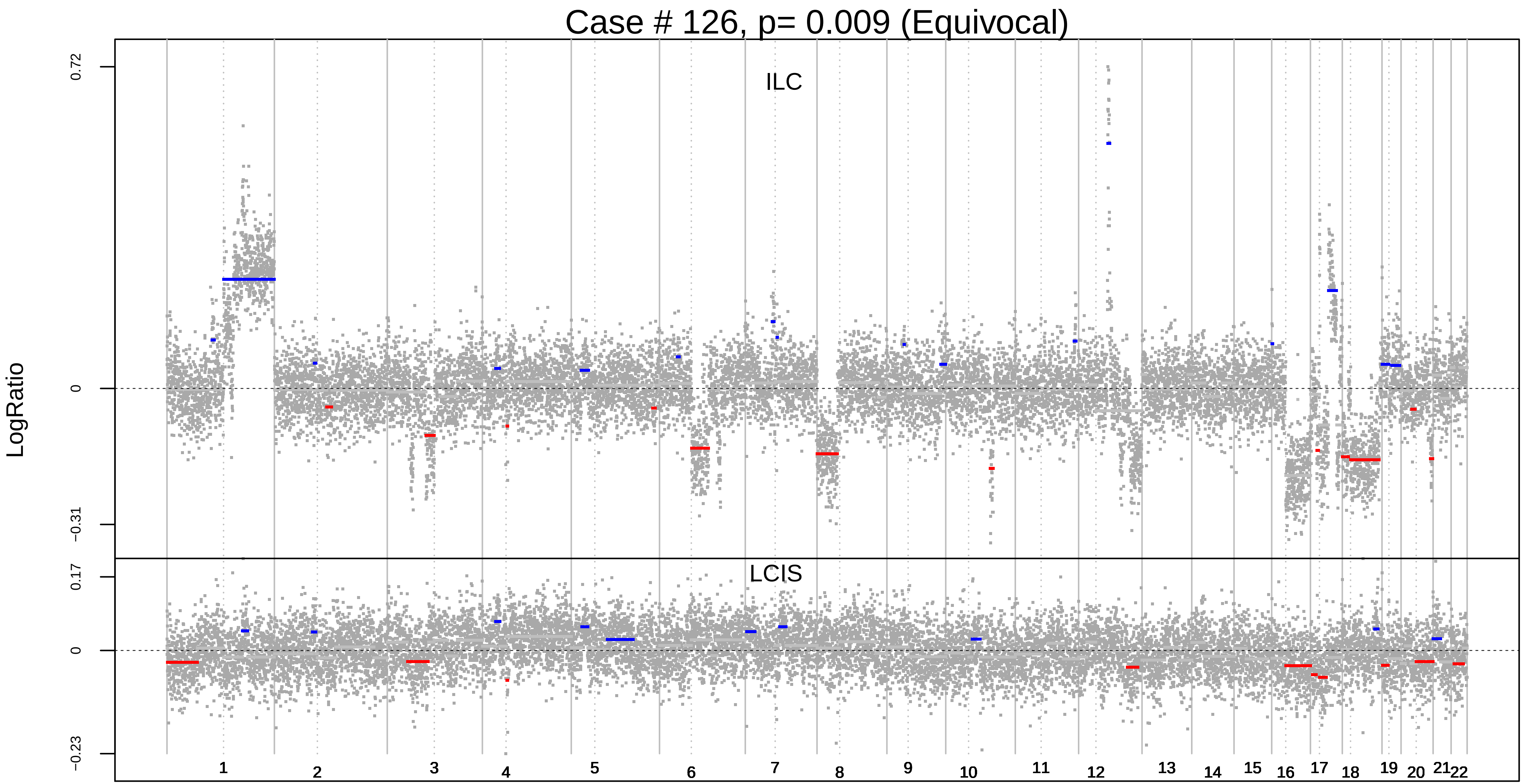

Case # 126, p= 0.009 (Equivocal)
ILC
LCIS
LogRatio
0.72
0
−0.31
0.17
0
−0.23
1
2
3
4
5
6
7
8
9
10
11
12
13
14
15
16
17
18
19
20
21
22

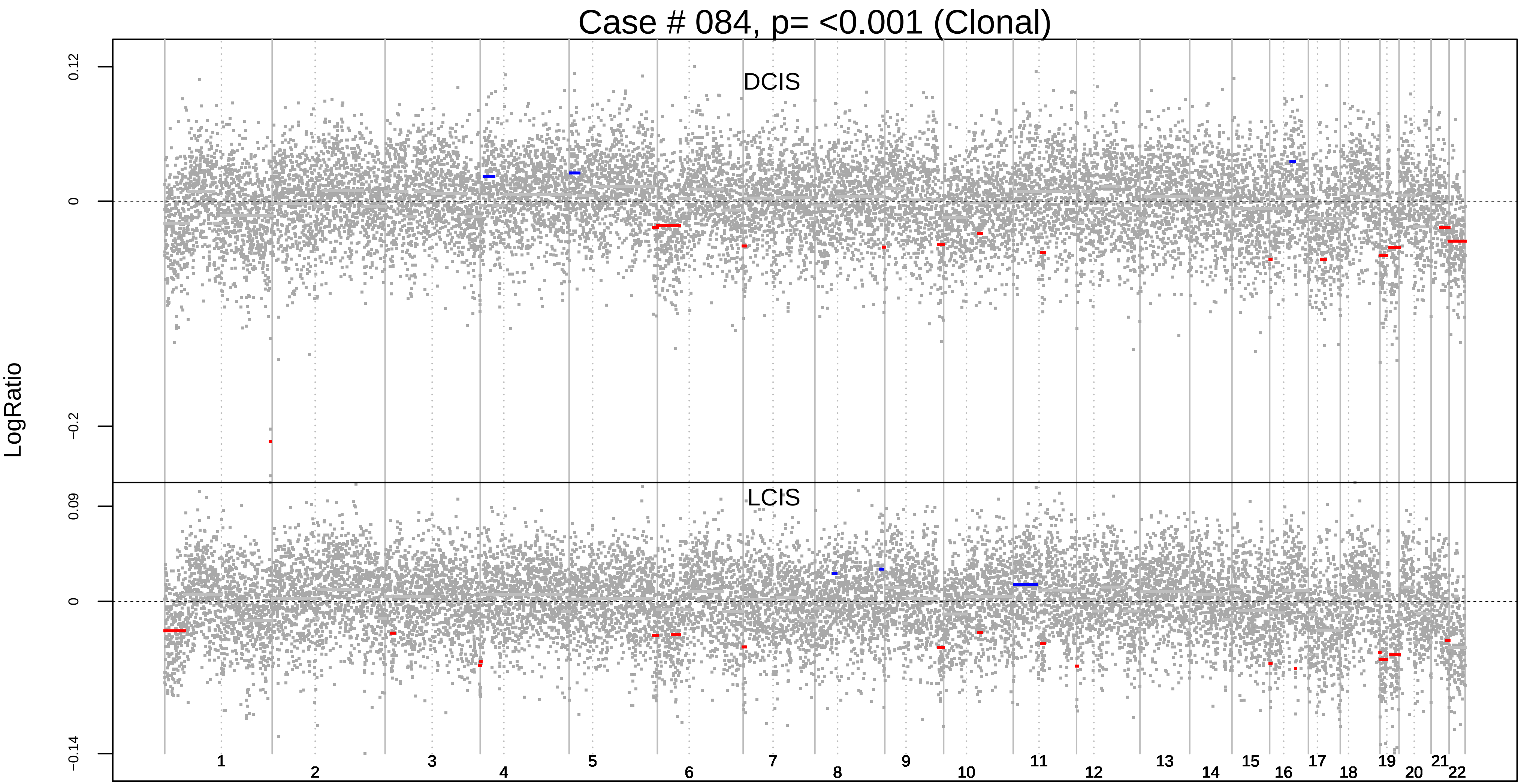

Case # 084, p= <0.001 (Clonal)
DCIS
LCIS
LogRatio
0.12
0
-0.2
0.09
0
-0.14
1
2
3
4
5
6
7
8
9
10
11
12
13
14
15
16
17
18
19
20
21
22

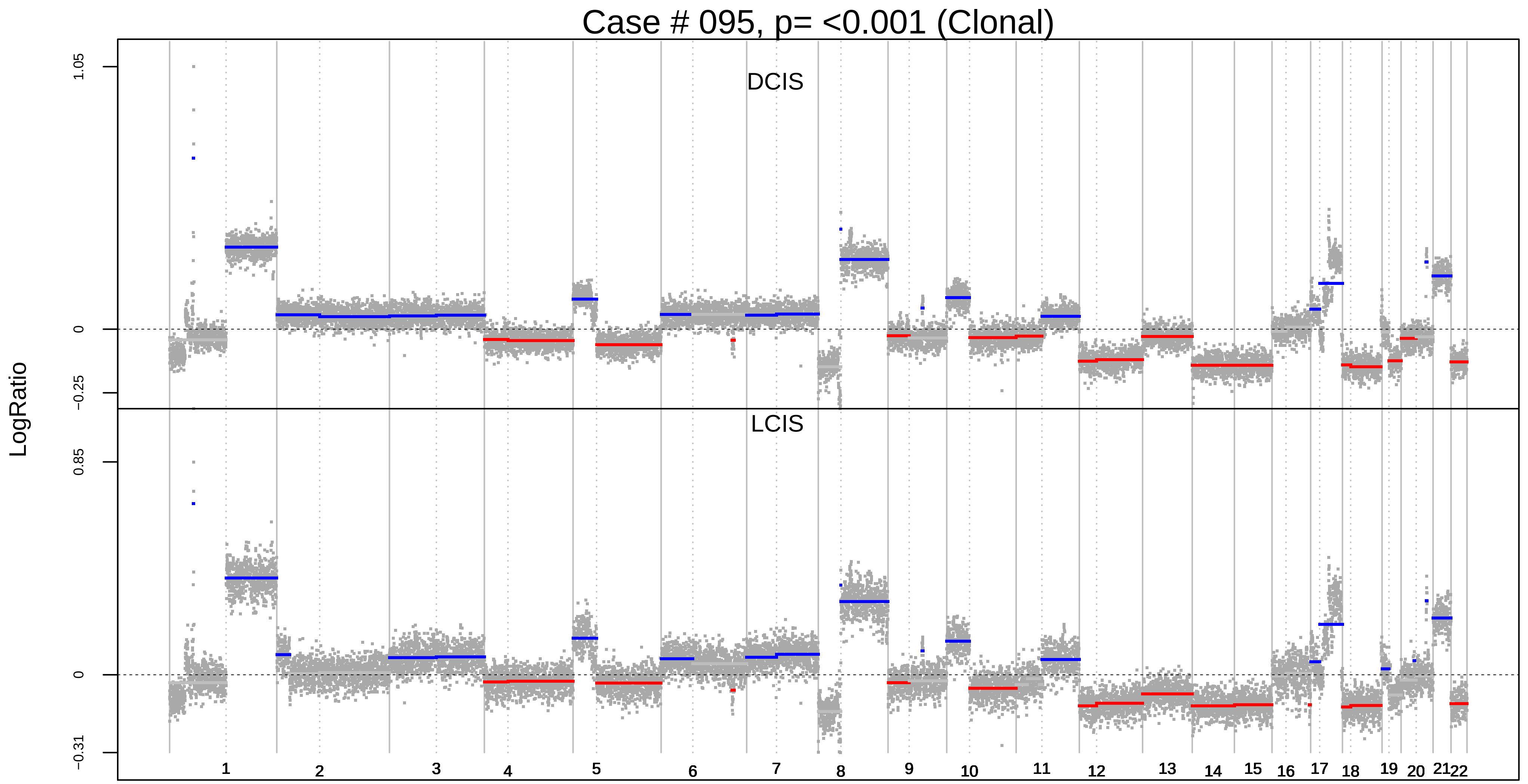
Case # 095, p= <0.001 (Clonal)
DCIS
LCIS
LogRatio
1.05
0
−0.25
0.85
0
−0.31
1
2
3
4
5
6
7
8
9
10
11
12
13
14
15
16
17
18
19
20
21
22

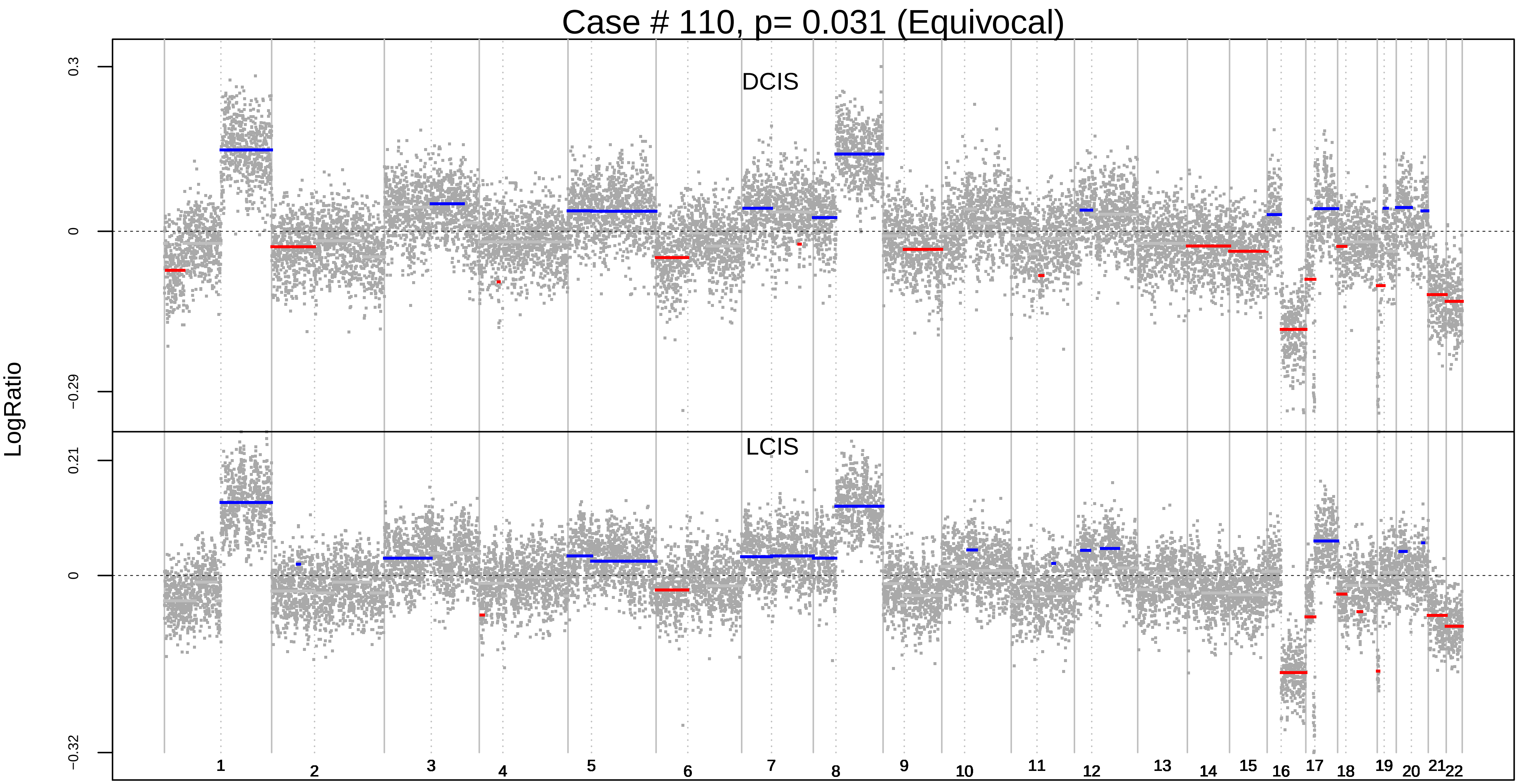
Case # 110, p= 0.031 (Equivocal)
DCIS
LCIS
LogRatio
0.3
0
−0.29
0.21
0
−0.32
1
2
3
4
5
6
7
8
9
10
11
12
13
14
15
16
17
18
19
20
21
22

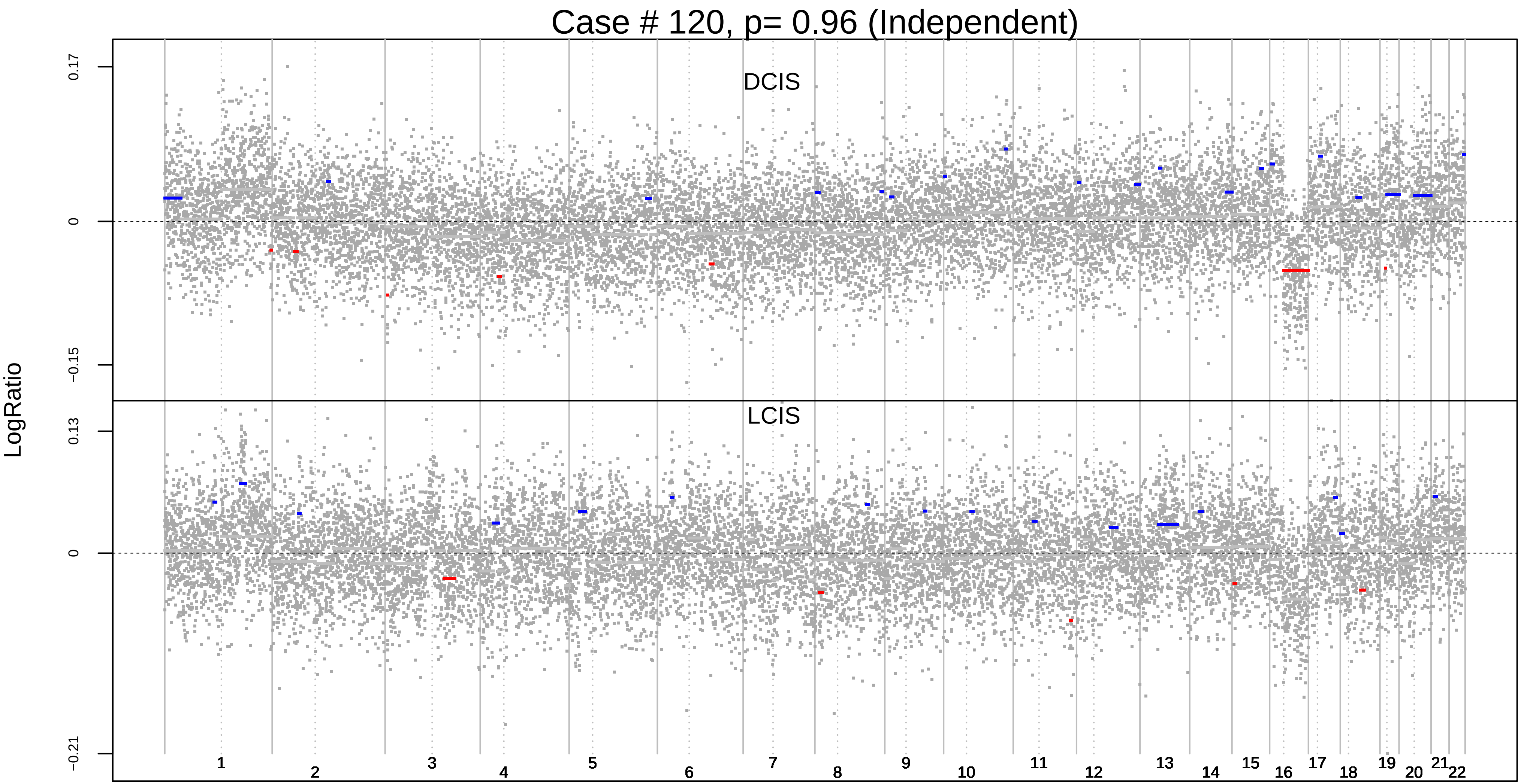
Case # 120, p= 0.96 (Independent)
DCIS
LCIS
LogRatio
0.17
0
−0.15
0.13
0
−0.21
1
2
3
4
5
6
7
8
9
10
11
12
13
14
15
16
17
18
19
20
21
22

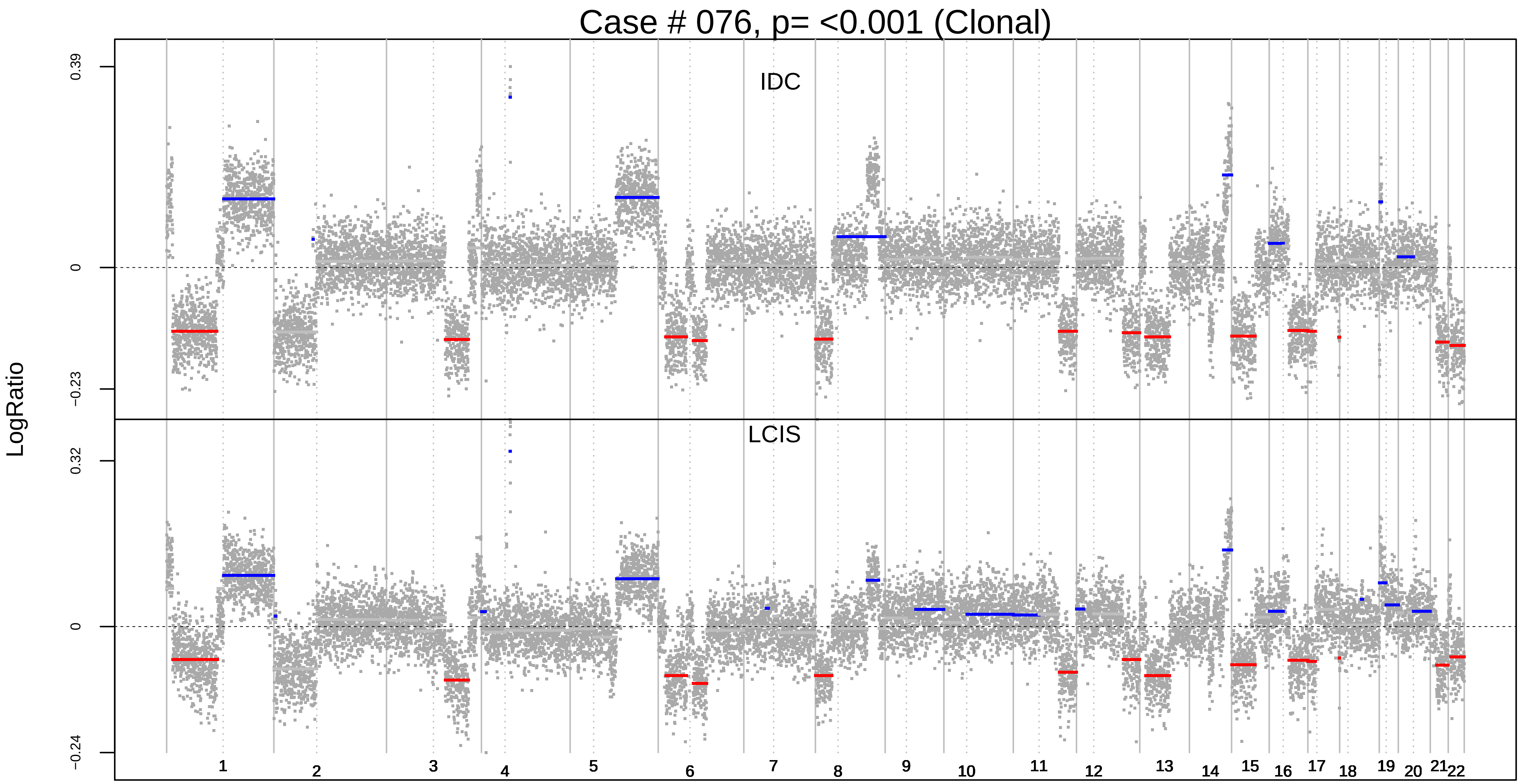

Case # 076, p= <0.001 (Clonal)
IDC
LCIS
LogRatio
0.39
0
−0.23
0.32
0
−0.24
1
2
3
4
5
6
7
8
9
10
11
12
13
14
15
16
17
18
19
20
21
22

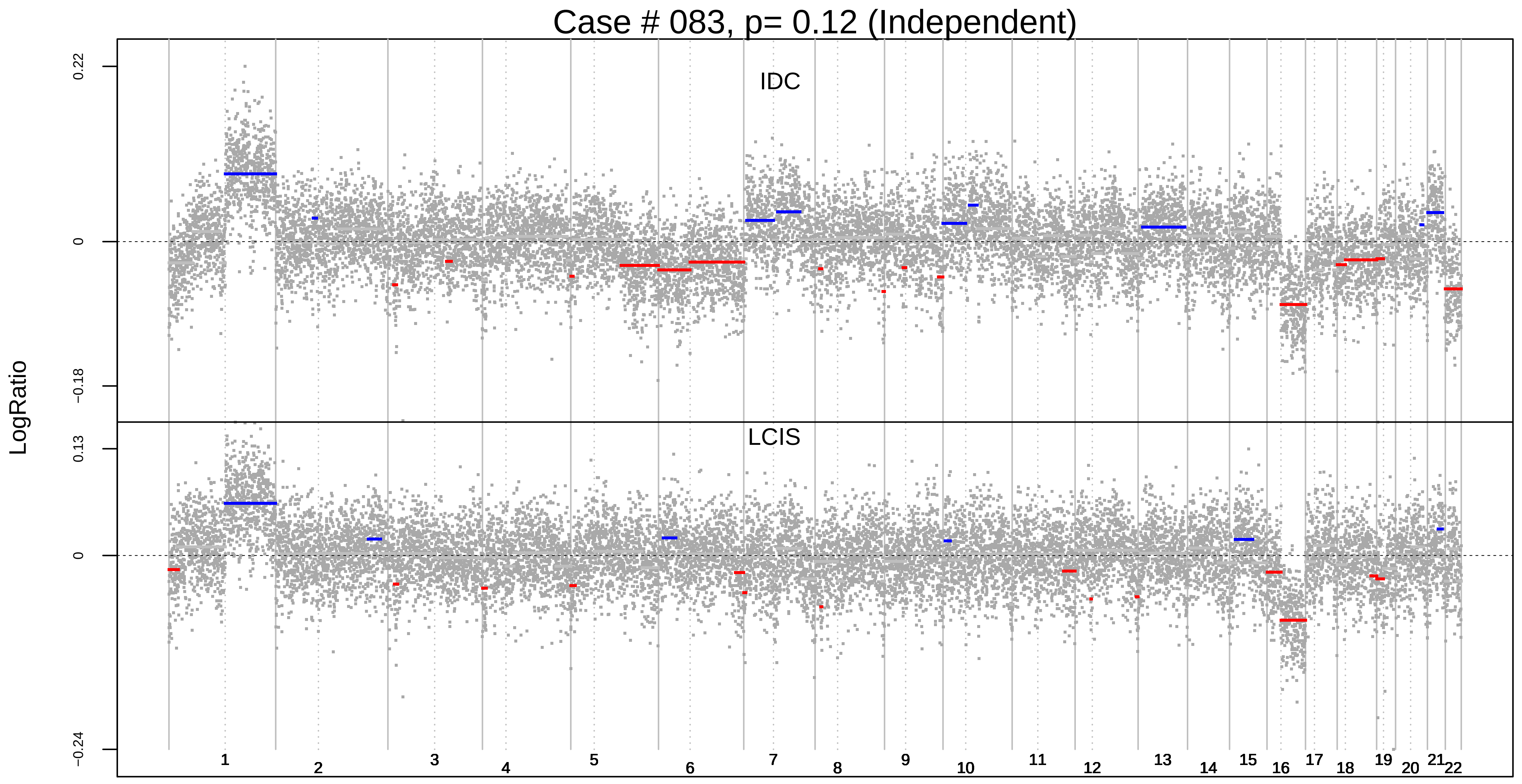

Case # 083, p= 0.12 (Independent)
IDC
LCIS
LogRatio
0.22
0
−0.18
0.13
0
−0.24
1
2
3
4
5
6
7
8
9
10
11
12
13
14
15
16
17
18
19
20
21
22

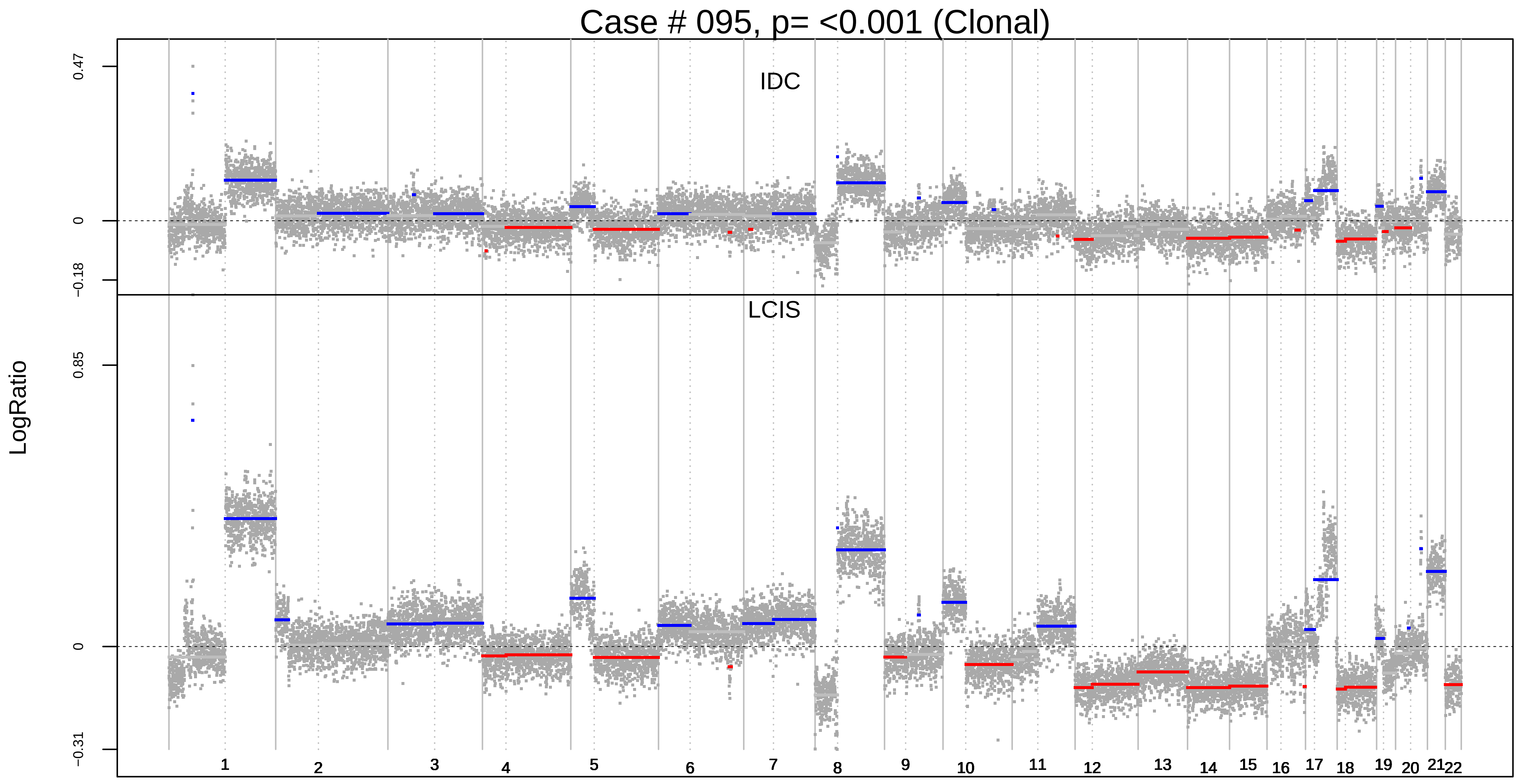

Case # 095, p= <0.001 (Clonal)
IDC
LCIS
LogRatio
0.47
0
−0.18
0.85
0
−0.31
1
2
3
4
5
6
7
8
9
10
11
12
13
14
15
16
17
18
19
20
21
22

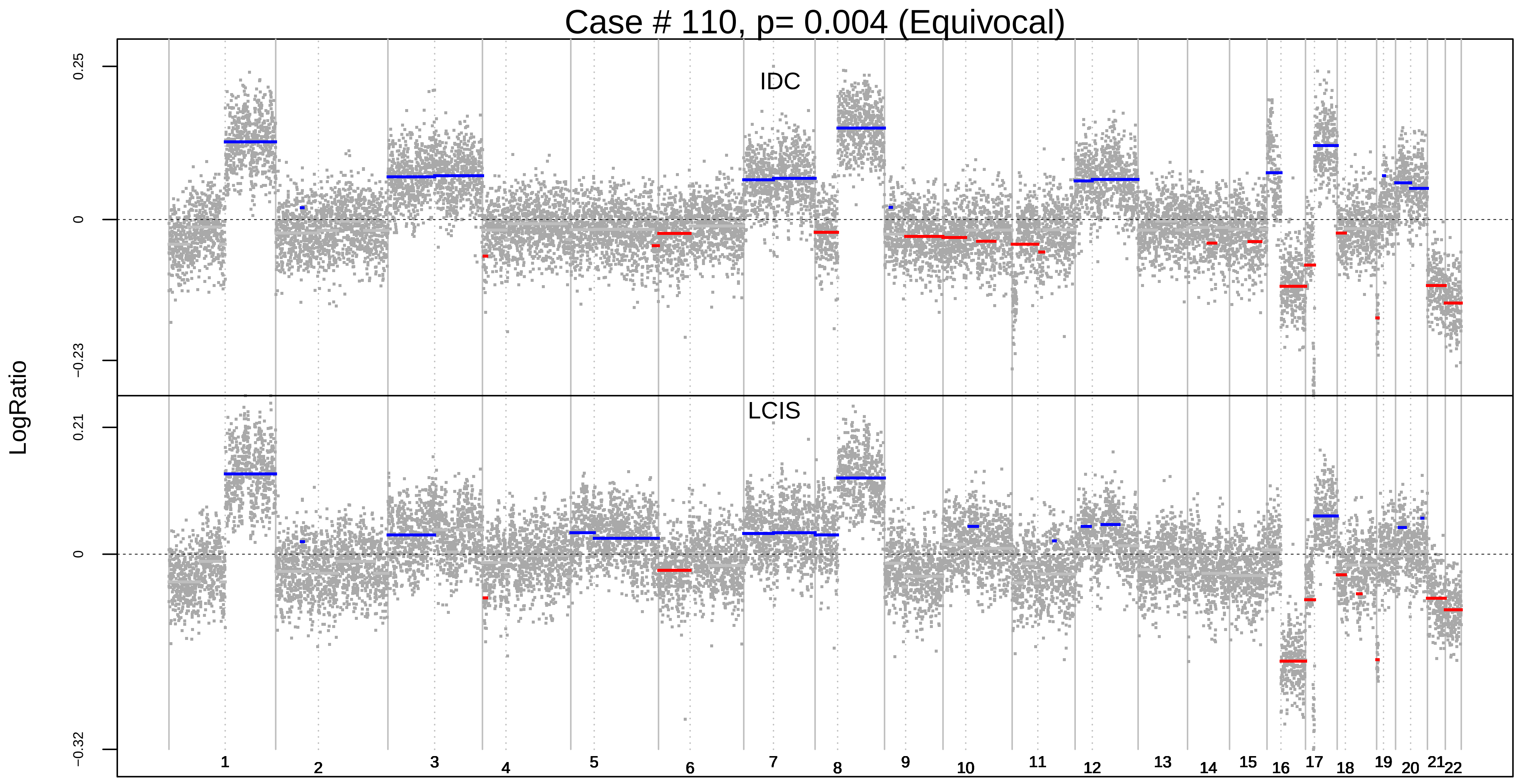

Case # 110, p= 0.004 (Equivocal)
IDC
LCIS
LogRatio
0.25
0
−0.23
0.21
0
−0.32
1
2
3
4
5
6
7
8
9
10
11
12
13
14
15
16
17
18
19
20
21
22
