# Supplementary material for: Clonal relatedness between lobular carcinoma in situ and synchronous malignant lesions
Source: Breast Cancer Res. 2012 Jul 9;14(4):R103. doi: 10.1186/bcr3222 (PMC3680923; doi:10.1186/bcr3222)
Supplement: Additional file 4 — Magnified version of genome-wide plots with detailed marker plots and segmentation on a chromosome-arm-specific basis. [file bcr3222-S4.ZIP › Case 114 a.pdf]

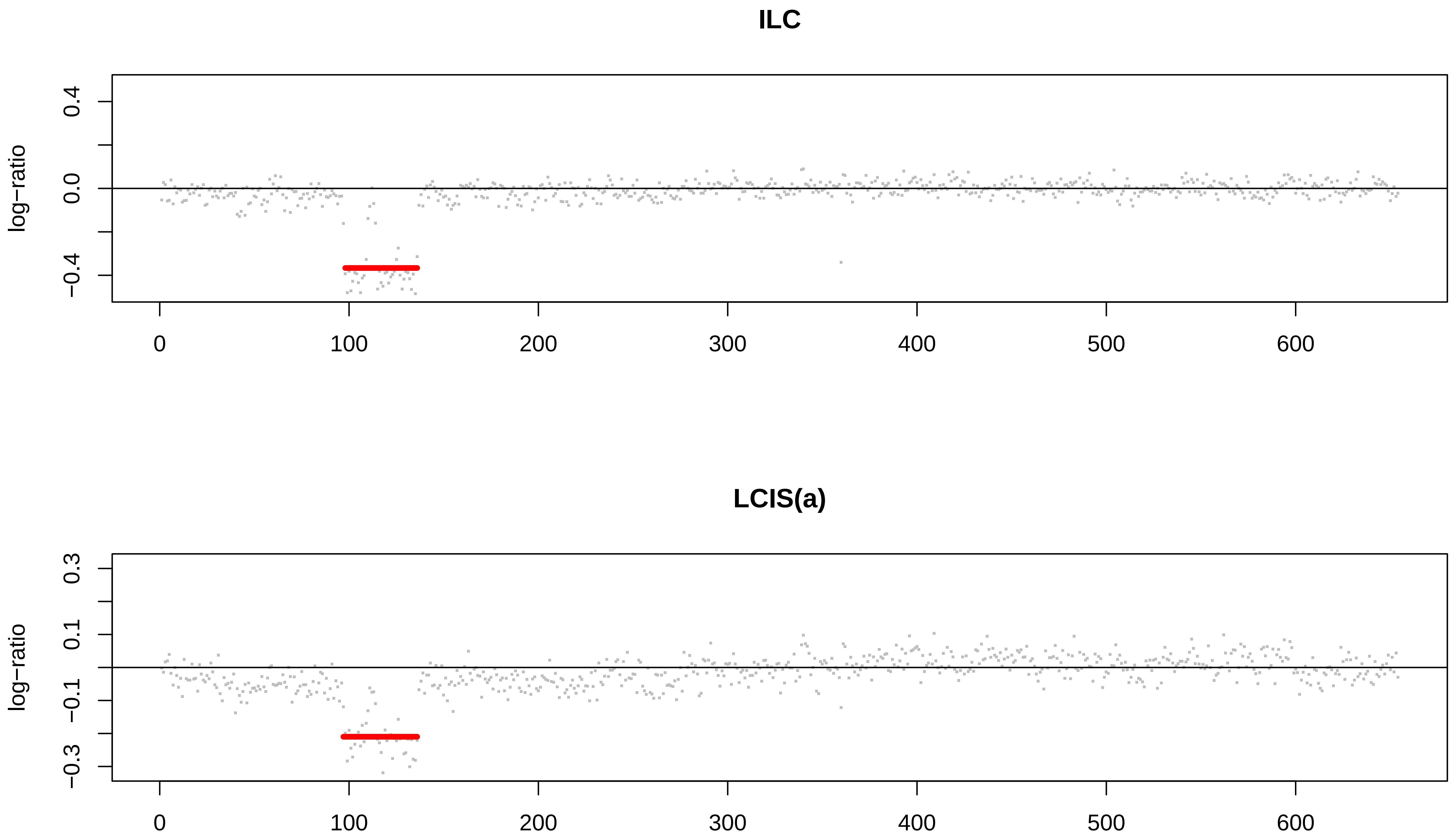

Case # 114, Chromosome 01p
Odds in favor of clonality = 6.2e+02

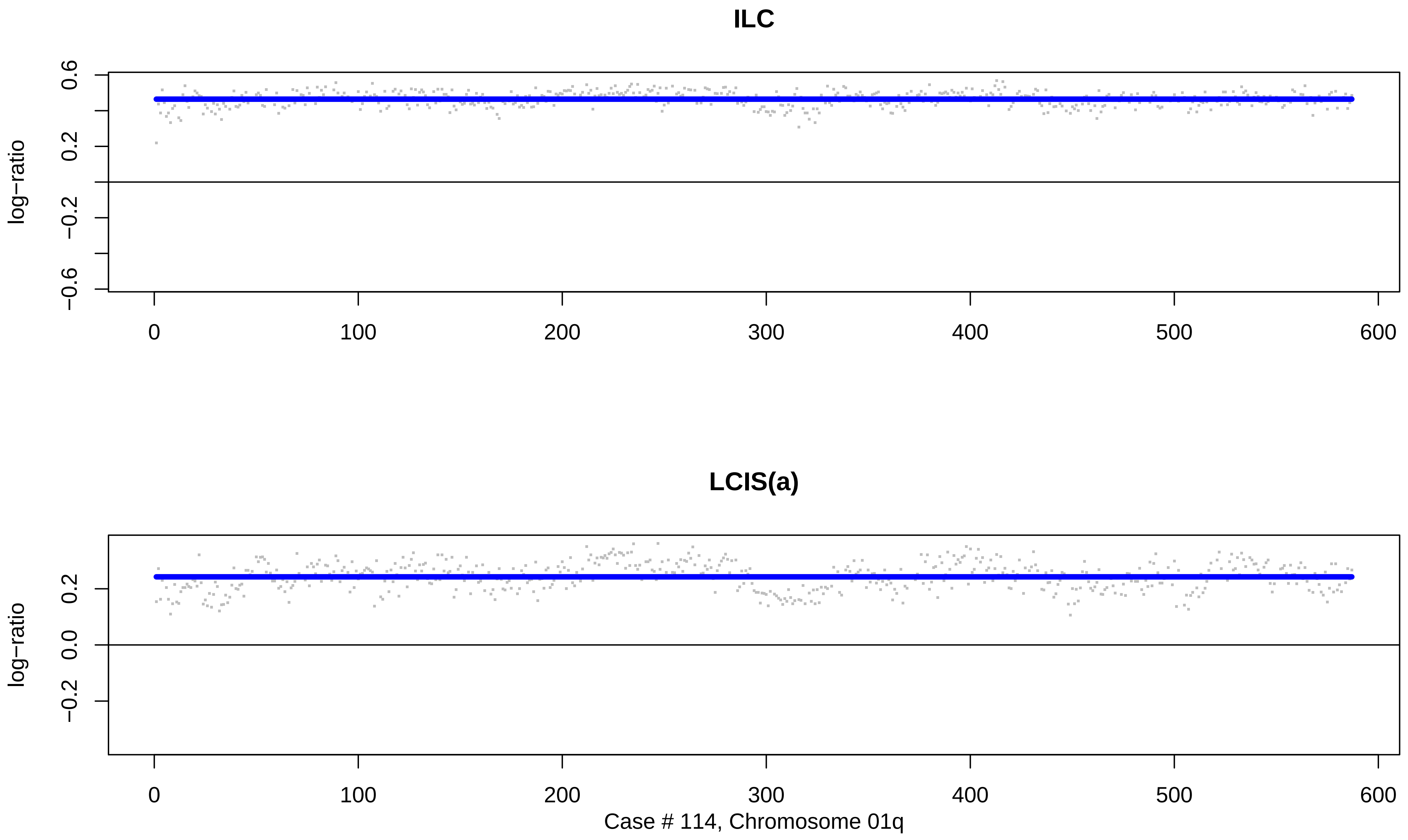
ILC
log−ratio
0.6
0.2
−0.2
−0.6
0
100
200
300
400
500
600
LCIS(a)
log−ratio
0.2
0.0
−0.2
0
100
200
300
400
500
600
Case # 114, Chromosome 01q

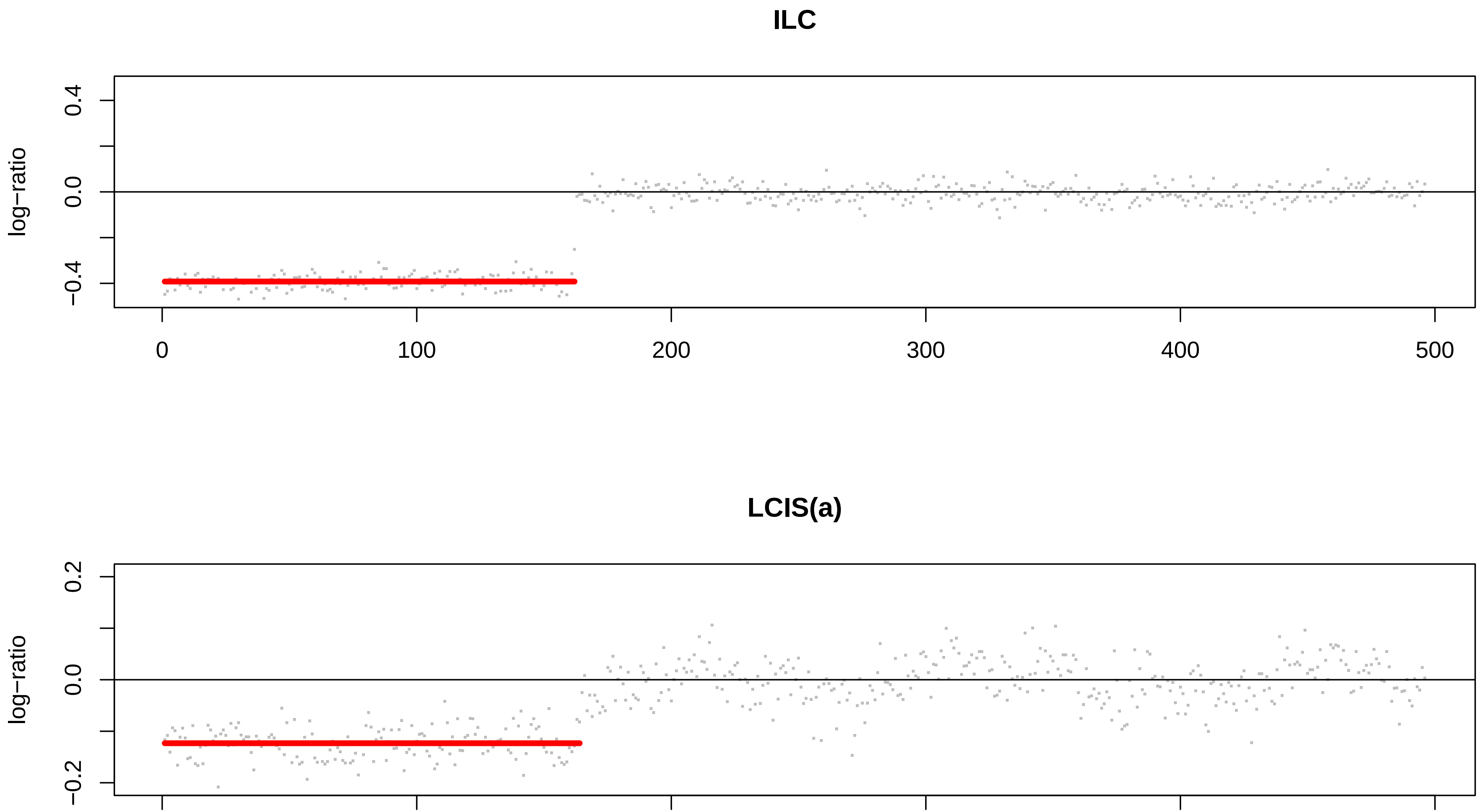

Case # 114, Chromosome 02p
Odds in favor of clonality = 2.5

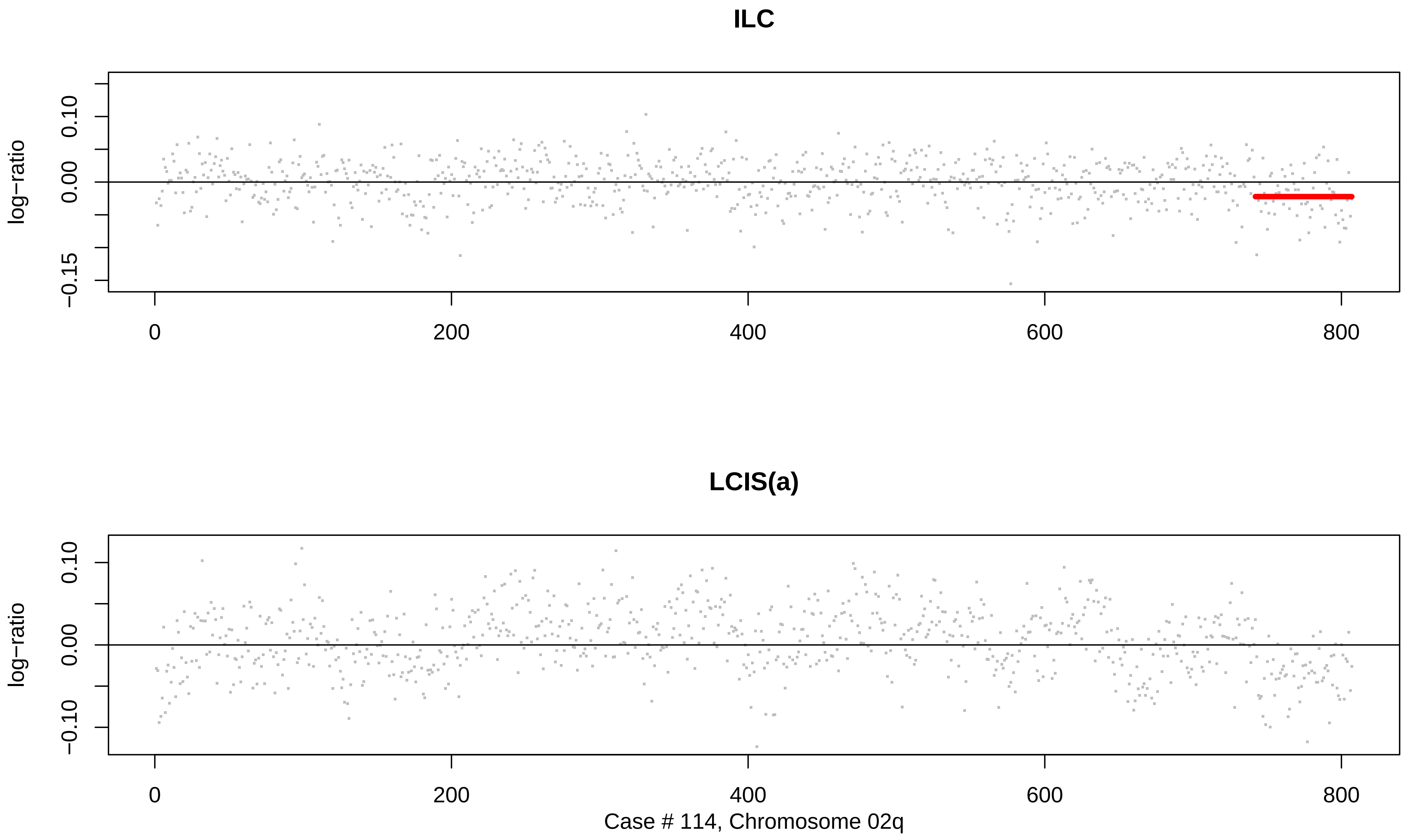
ILC
log−ratio
0.10
0.00
−0.15
0
200
400
600
800
LCIS(a)
log−ratio
0.10
0.00
−0.10
0
200
400
600
800
Case # 114, Chromosome 02q

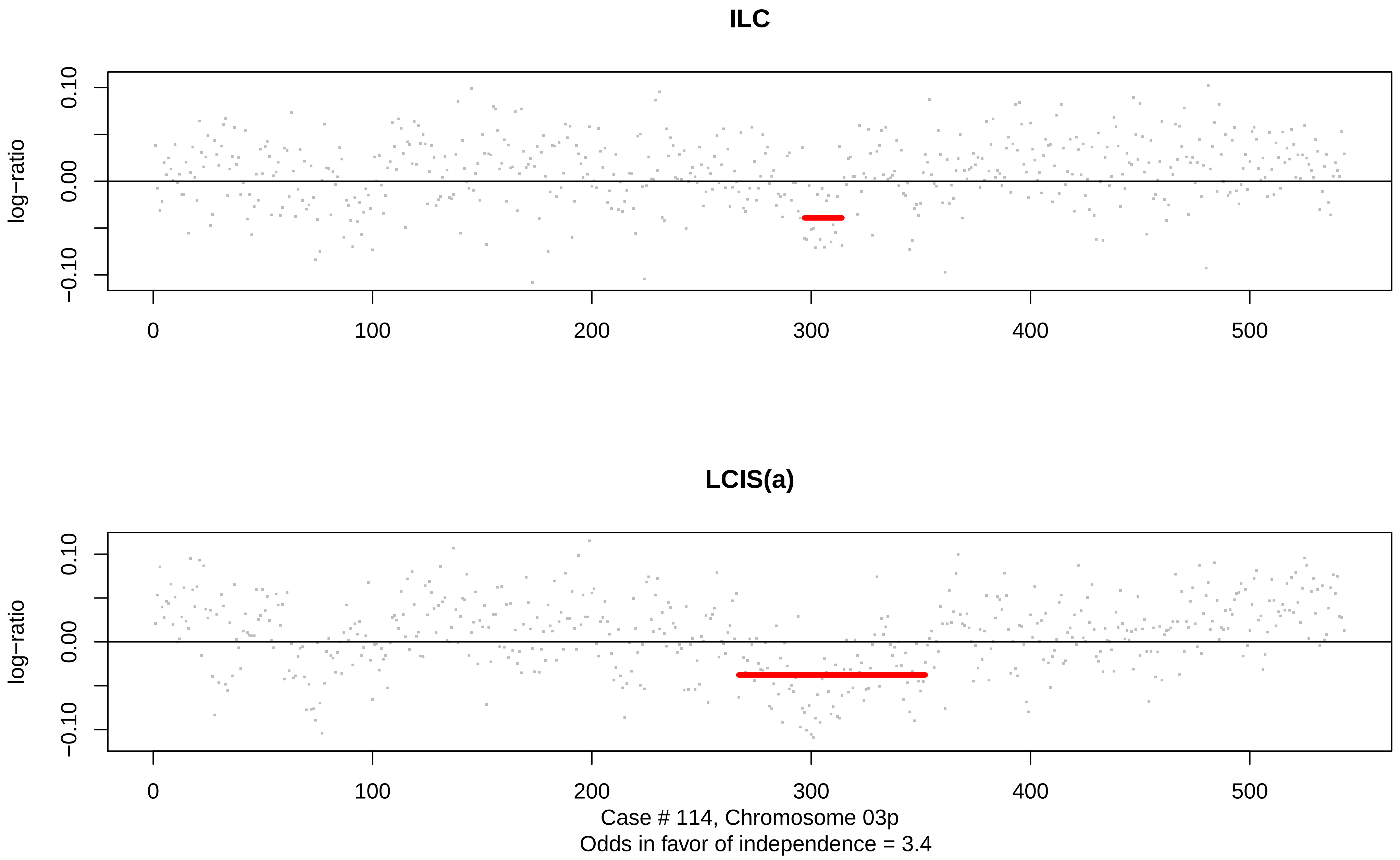
ILC
log−ratio
0.10
0.00
−0.10
0
100
200
300
400
500
LCIS(a)
log−ratio
0.10
0.00
−0.10
0
100
200
300
400
500
Case # 114, Chromosome 03p
Odds in favor of independence = 3.4

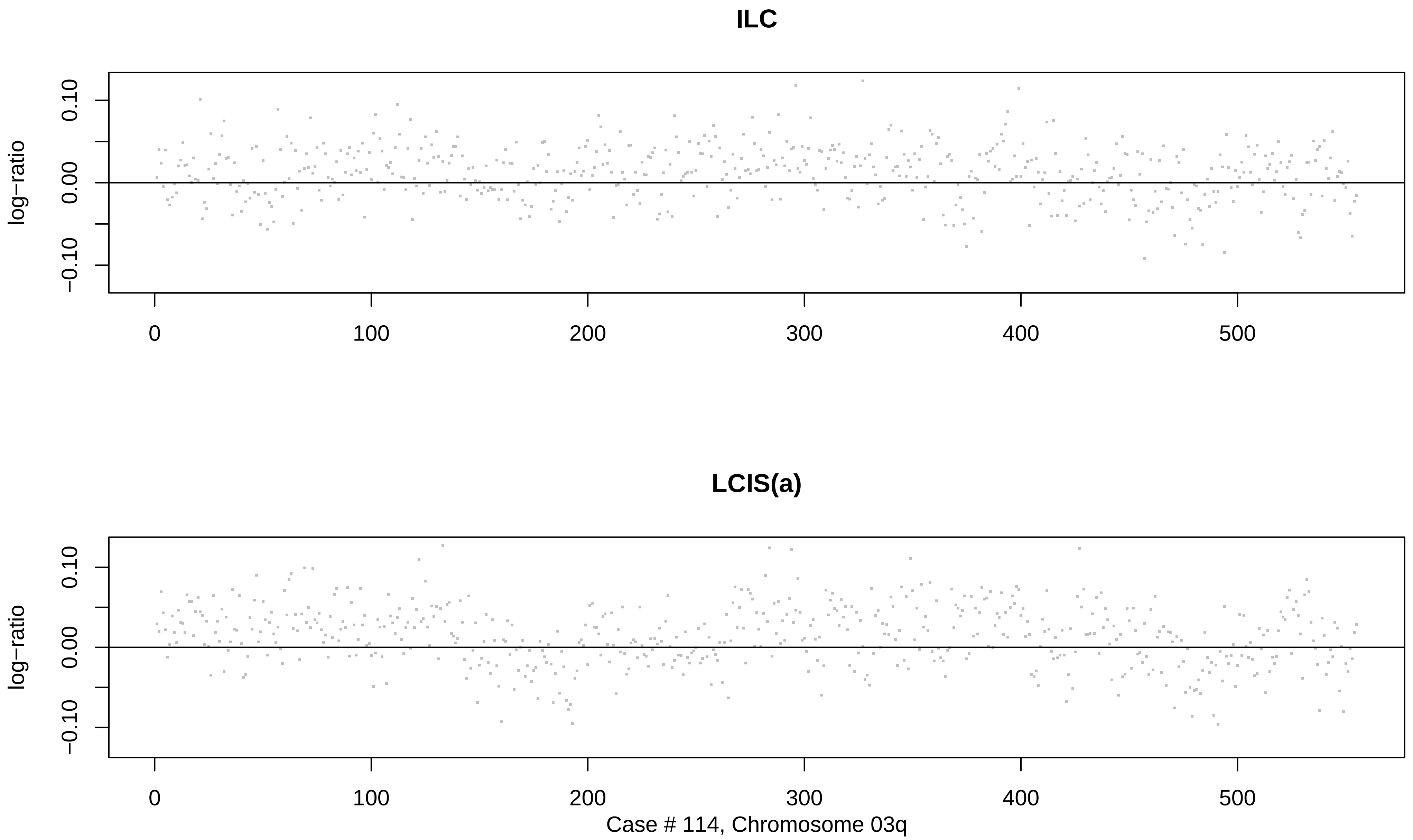
ILC
log−ratio
0.10
0.00
−0.10
0
100
200
300
400
500
LCIS(a)
log−ratio
0.10
0.00
−0.10
0
100
200
300
400
500
Case # 114, Chromosome 03q

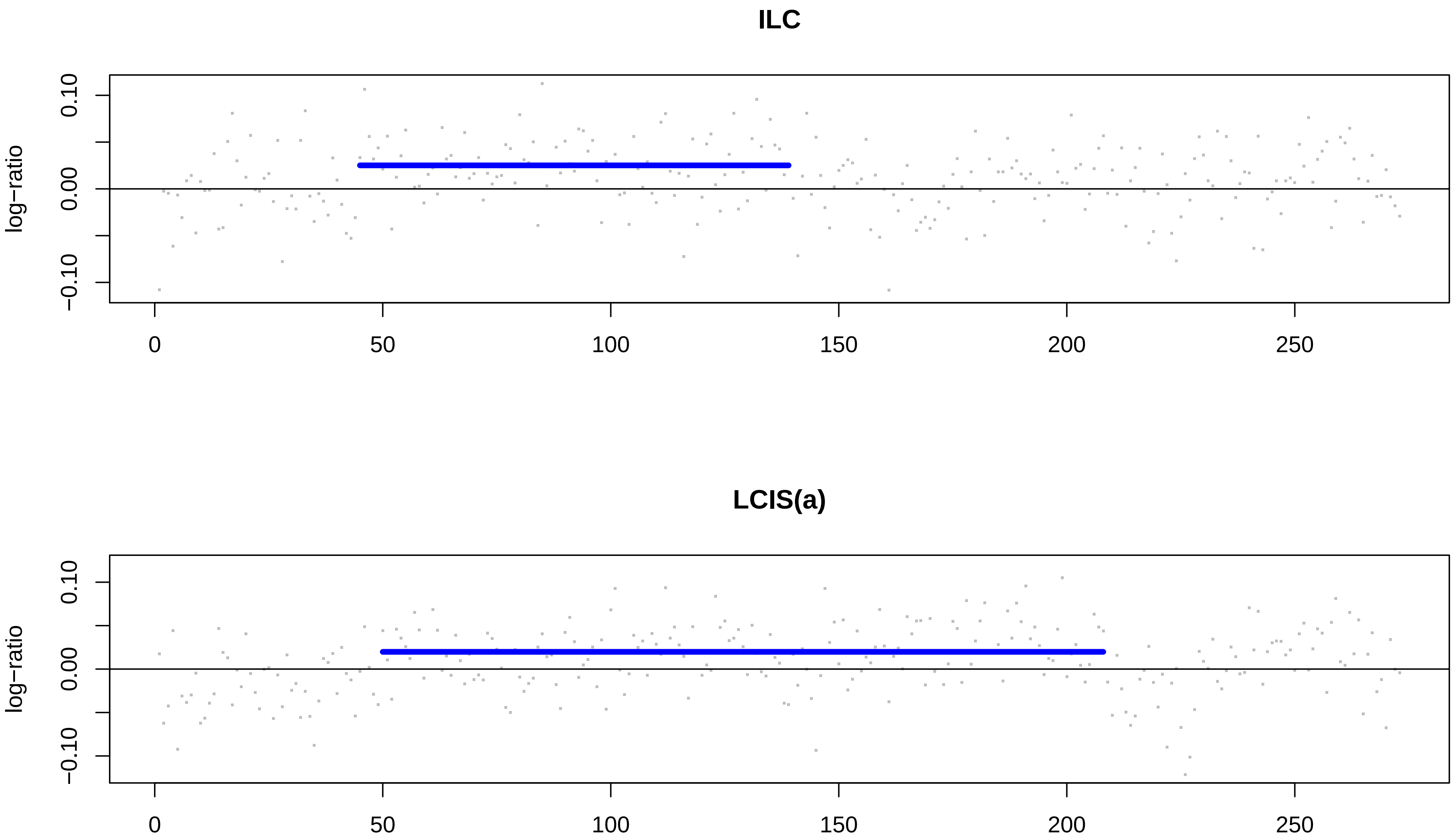

Case # 114, Chromosome 04p
Odds in favor of independence = 2.7

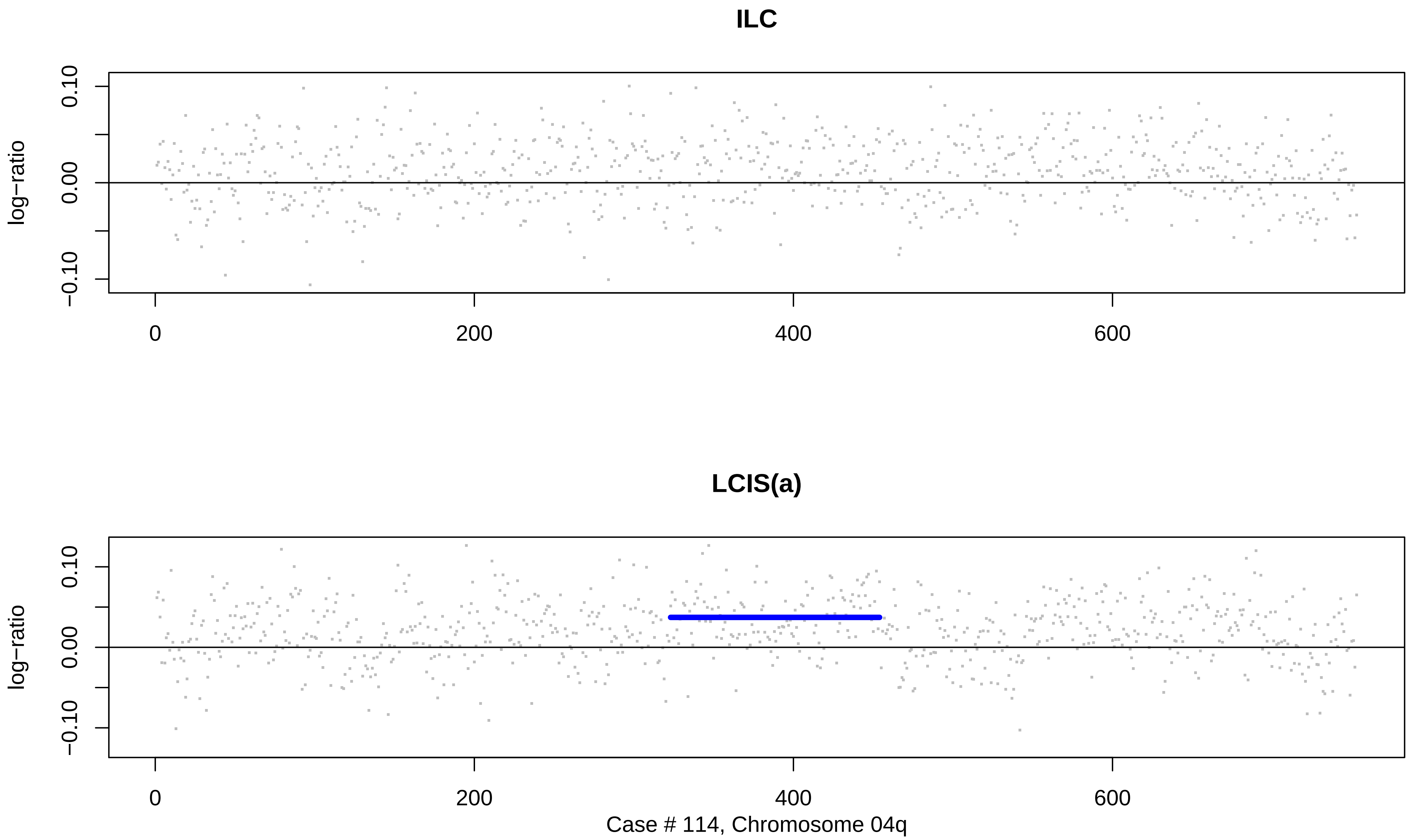
ILC
log−ratio
0.10
0.00
−0.10
0
200
400
600
LCIS(a)
log−ratio
0.10
0.00
−0.10
0
200
400
600
Case # 114, Chromosome 04q

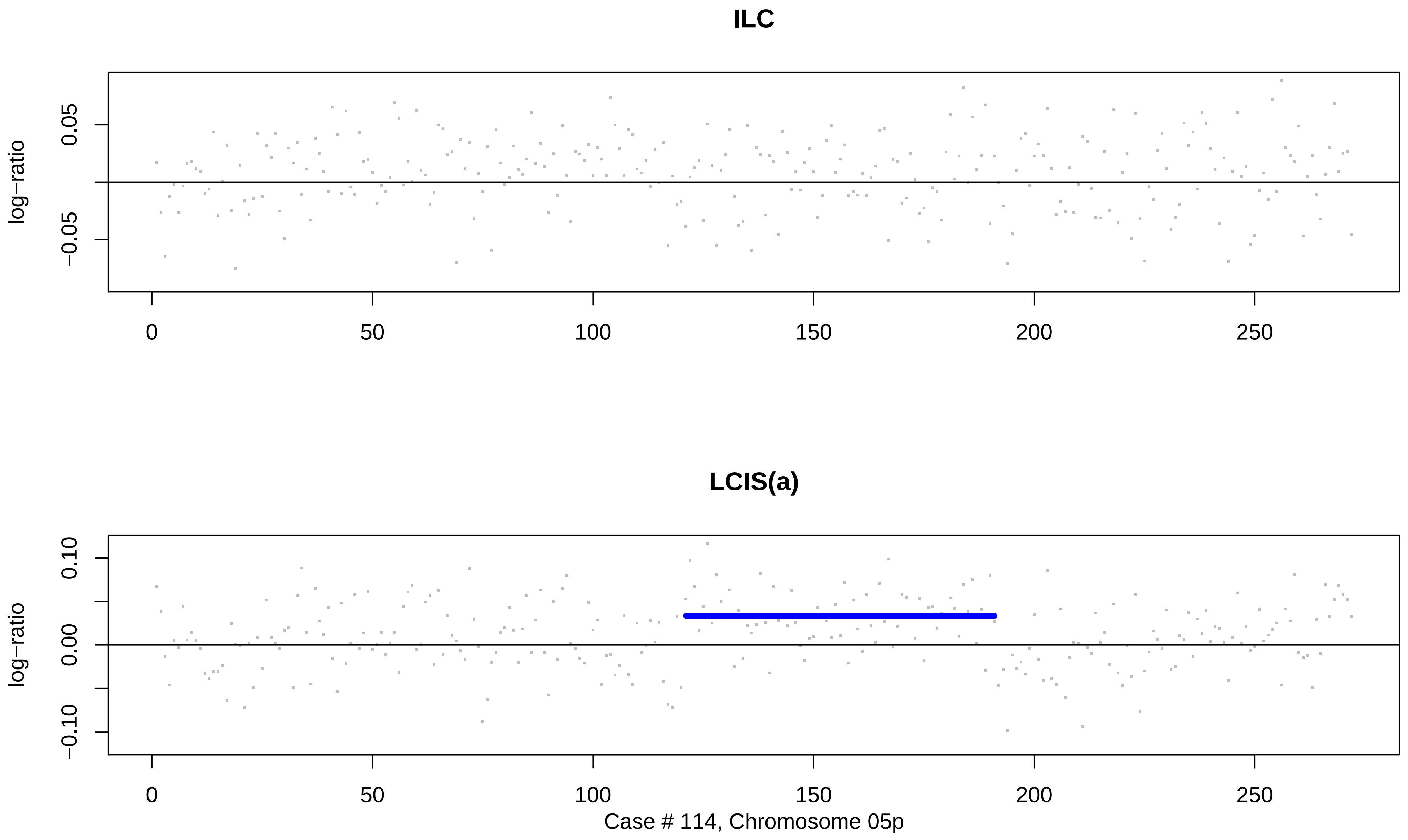

ILC
log−ratio
0.05
−0.05
0
50
100
150
200
250
LCIS(a)
log−ratio
0.10
0.00
−0.10
0
50
100
150
200
250
Case # 114, Chromosome 05p

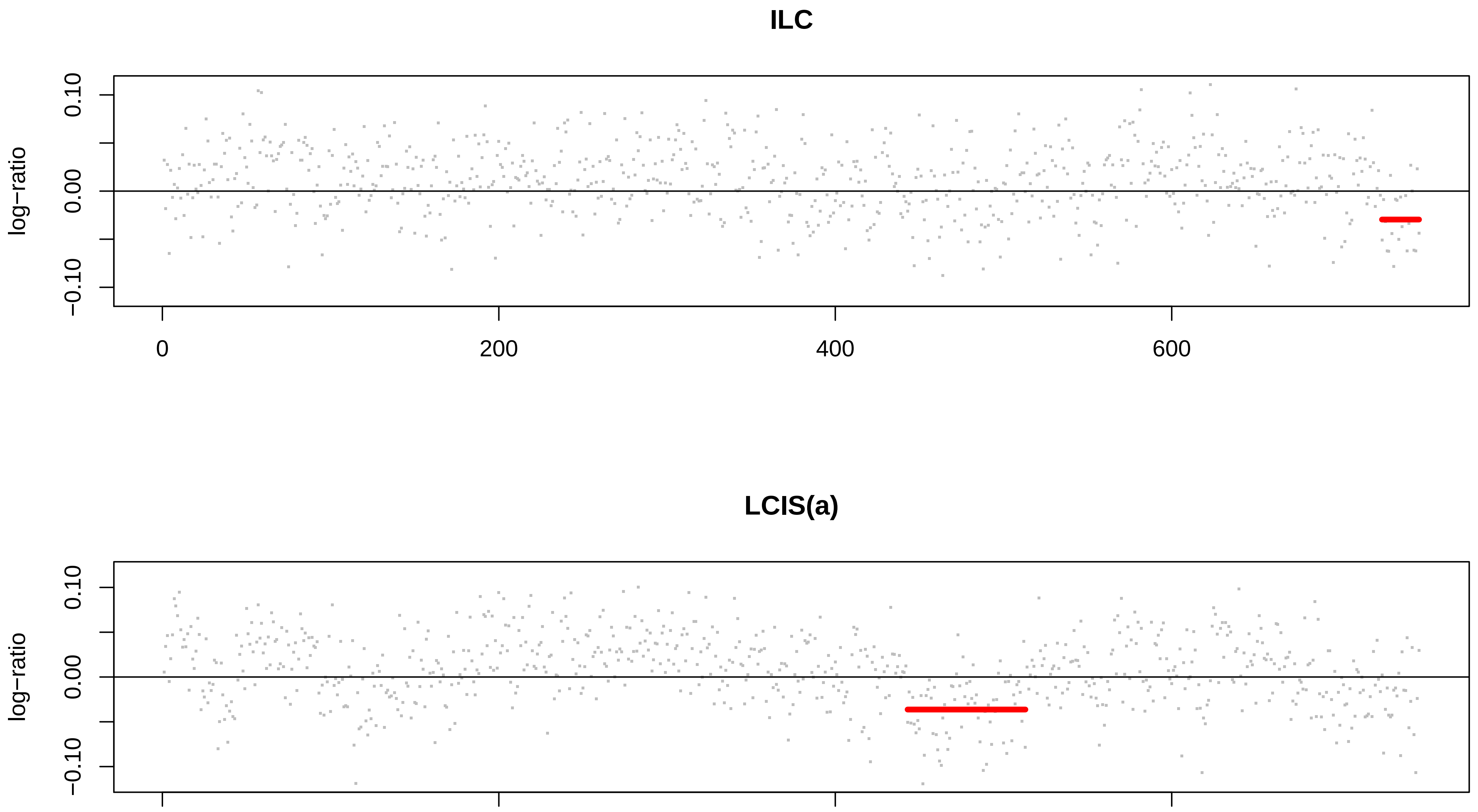

Case # 114, Chromosome 05q
Odds in favor of independence = 3.2

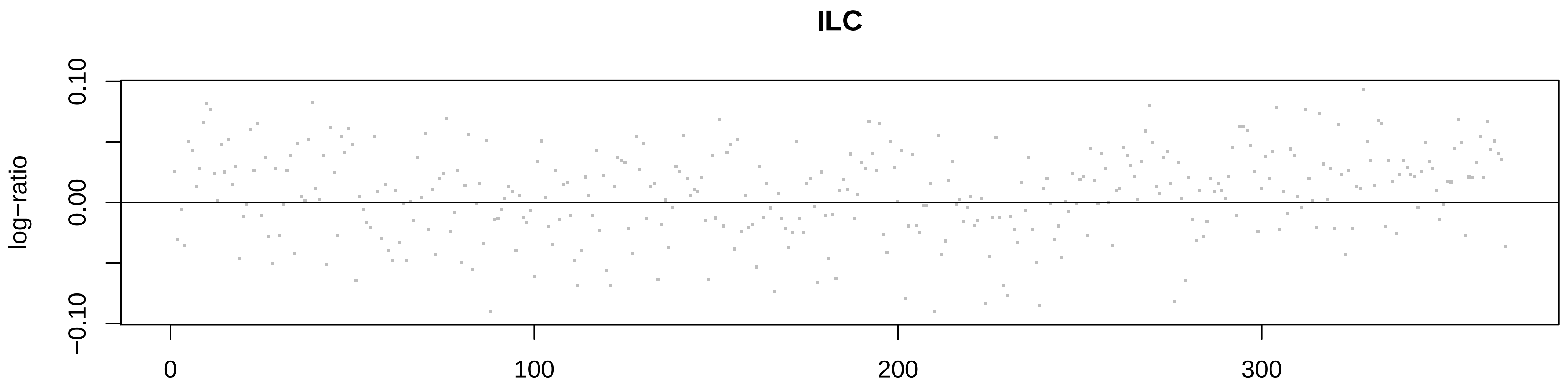

ILC
log−ratio
0.10
0.00
−0.10
0
100
200
300

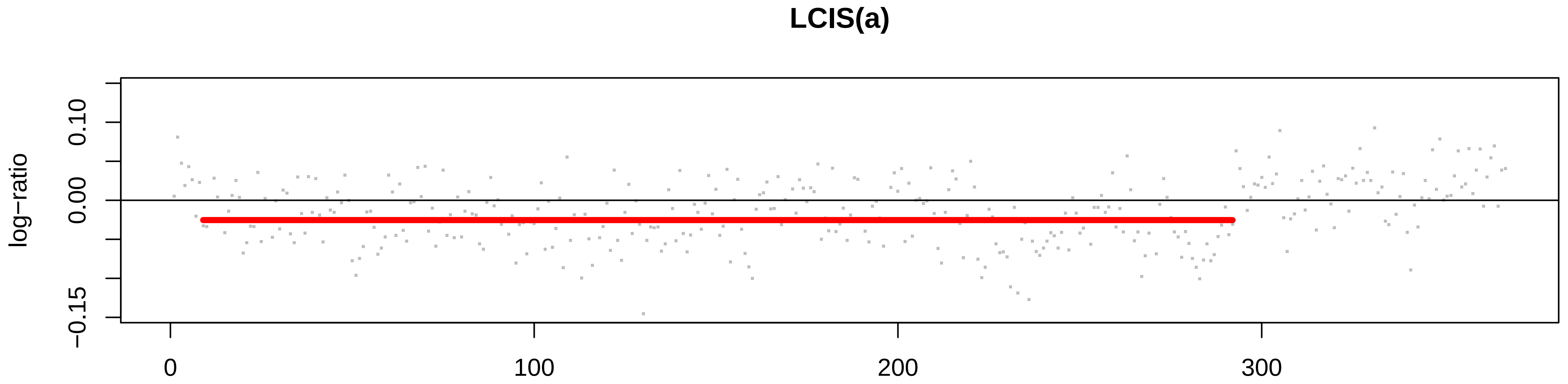

LCIS(a)
log−ratio
0.10
0.00
−0.15
0
100
200
300
Case # 114, Chromosome 06p

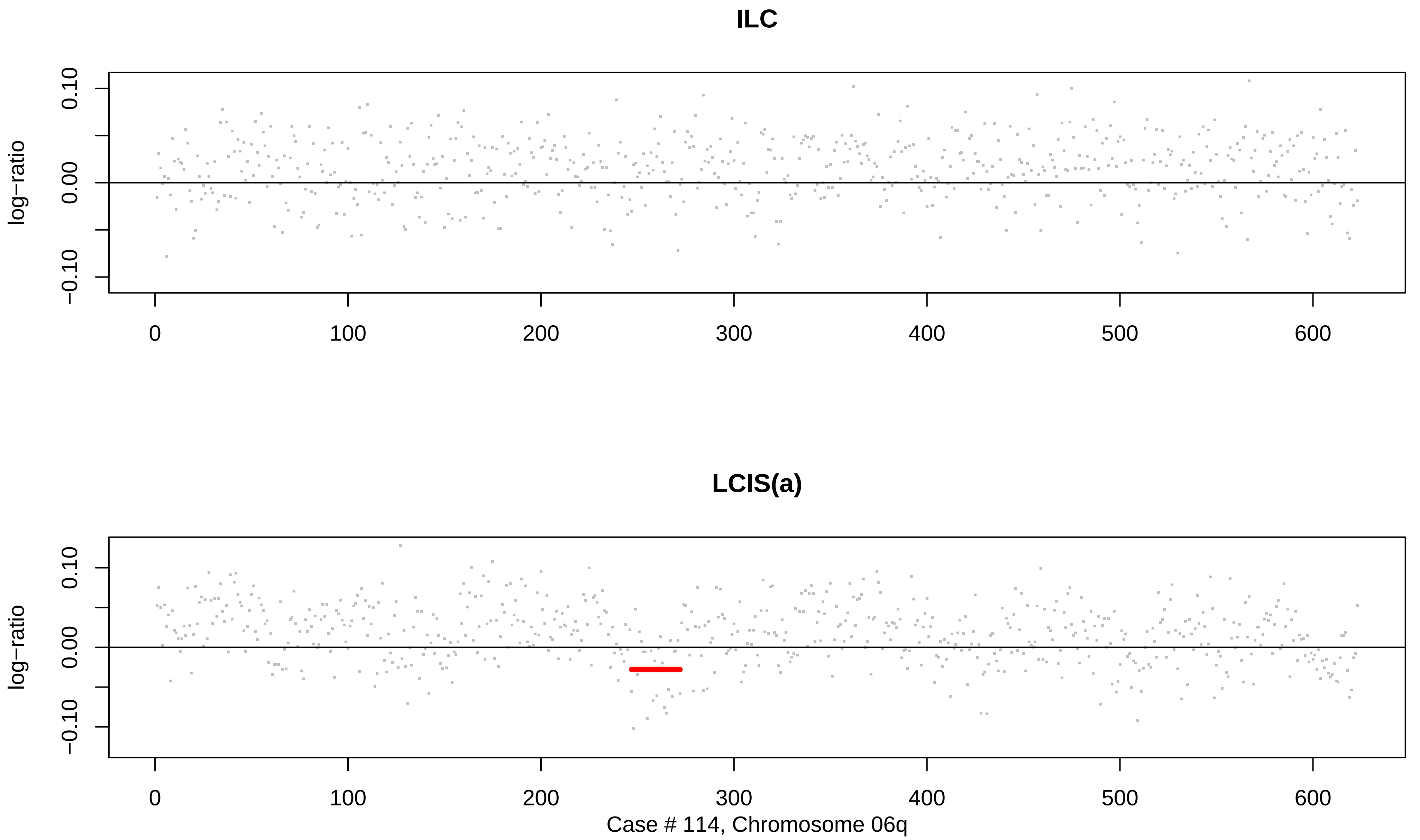
ILC
log−ratio
0.10
0.00
−0.10
0
100
200
300
400
500
600
LCIS(a)
log−ratio
0.10
0.00
−0.10
0
100
200
300
400
500
600
Case # 114, Chromosome 06q

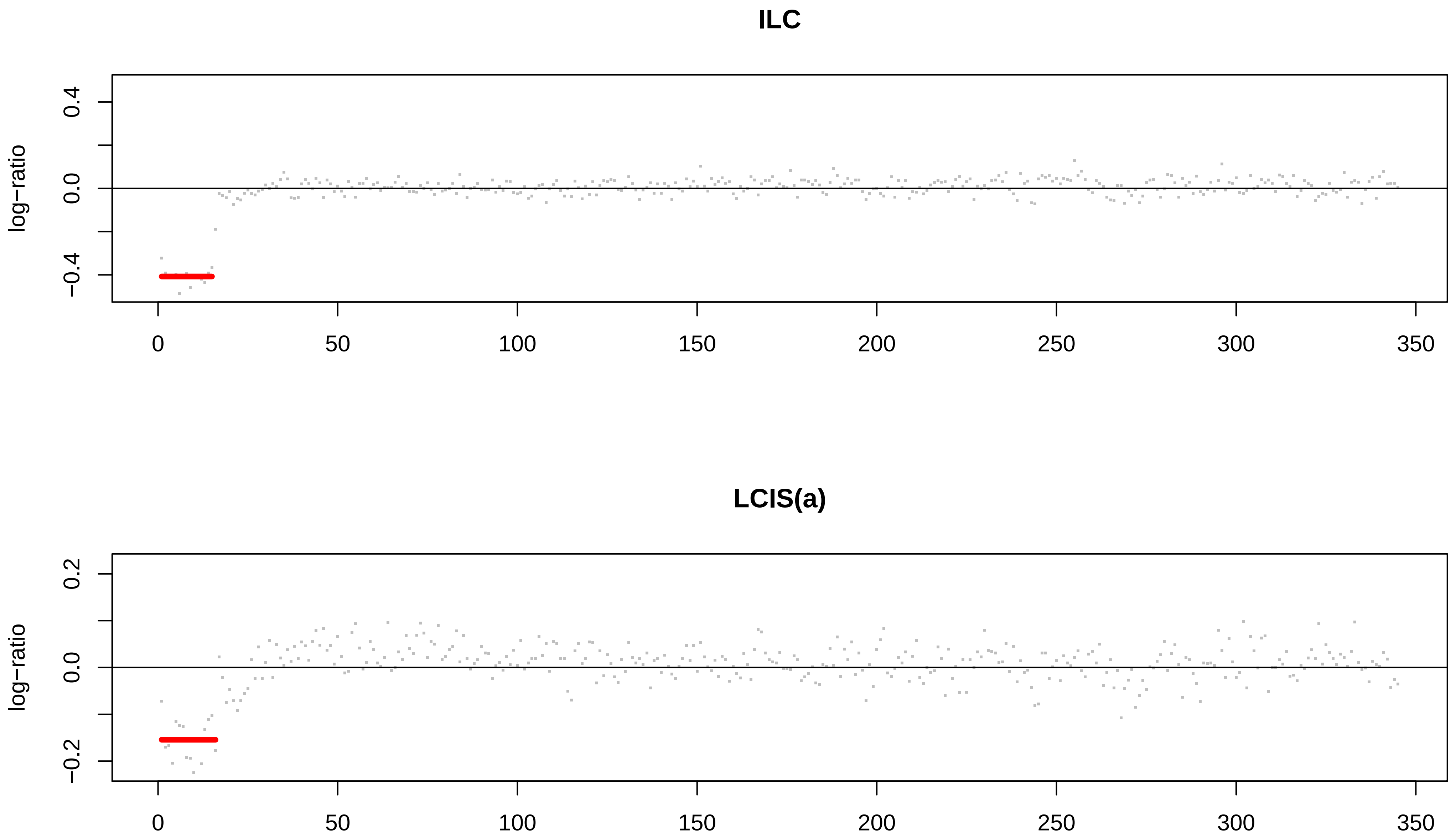

Case # 114, Chromosome 07p
Odds in favor of clonality = 65.8

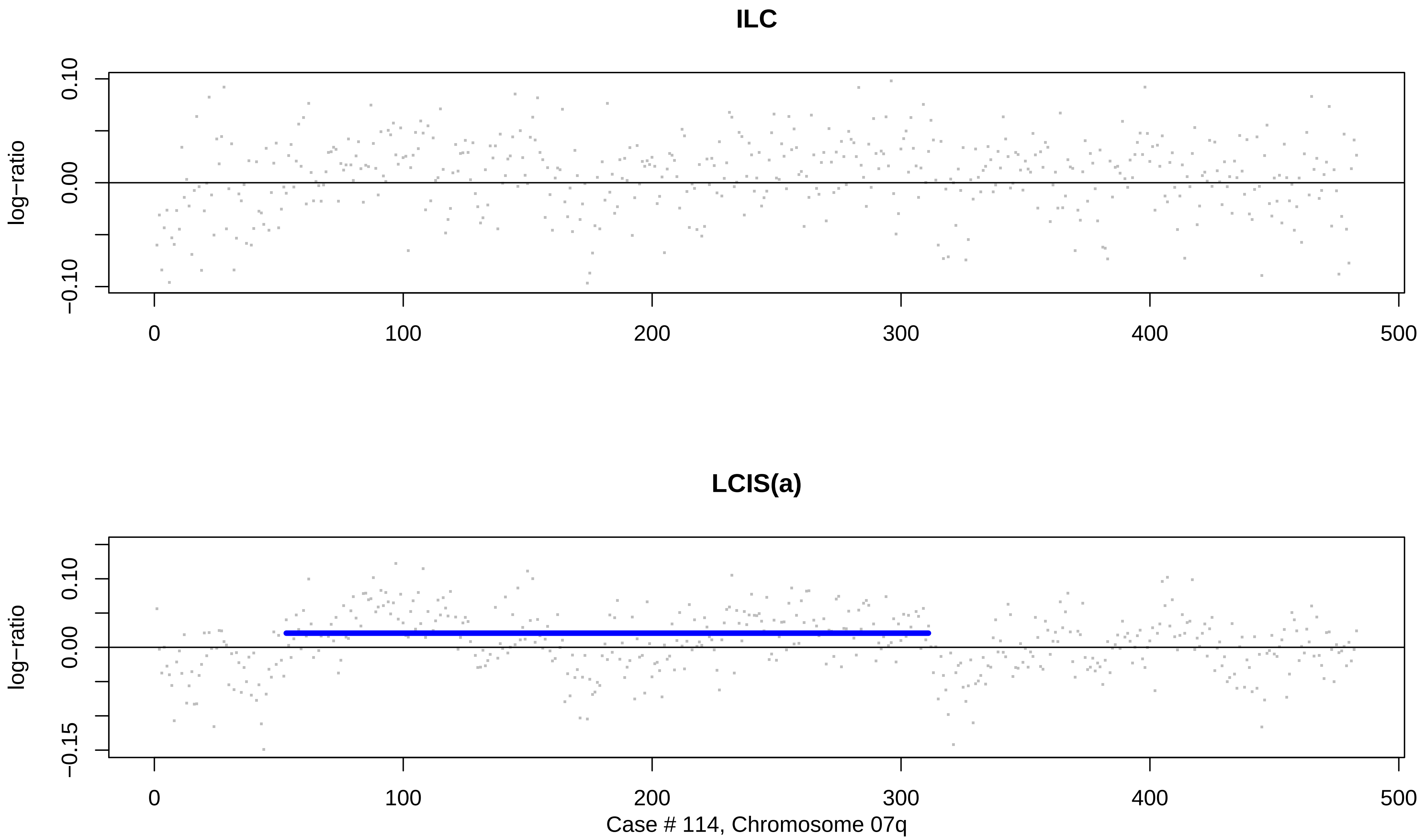
ILC
log−ratio
0.10
0.00
−0.10
0
100
200
300
400
500
LCIS(a)
log−ratio
0.10
0.00
−0.15
0
100
200
300
400
500
Case # 114, Chromosome 07q

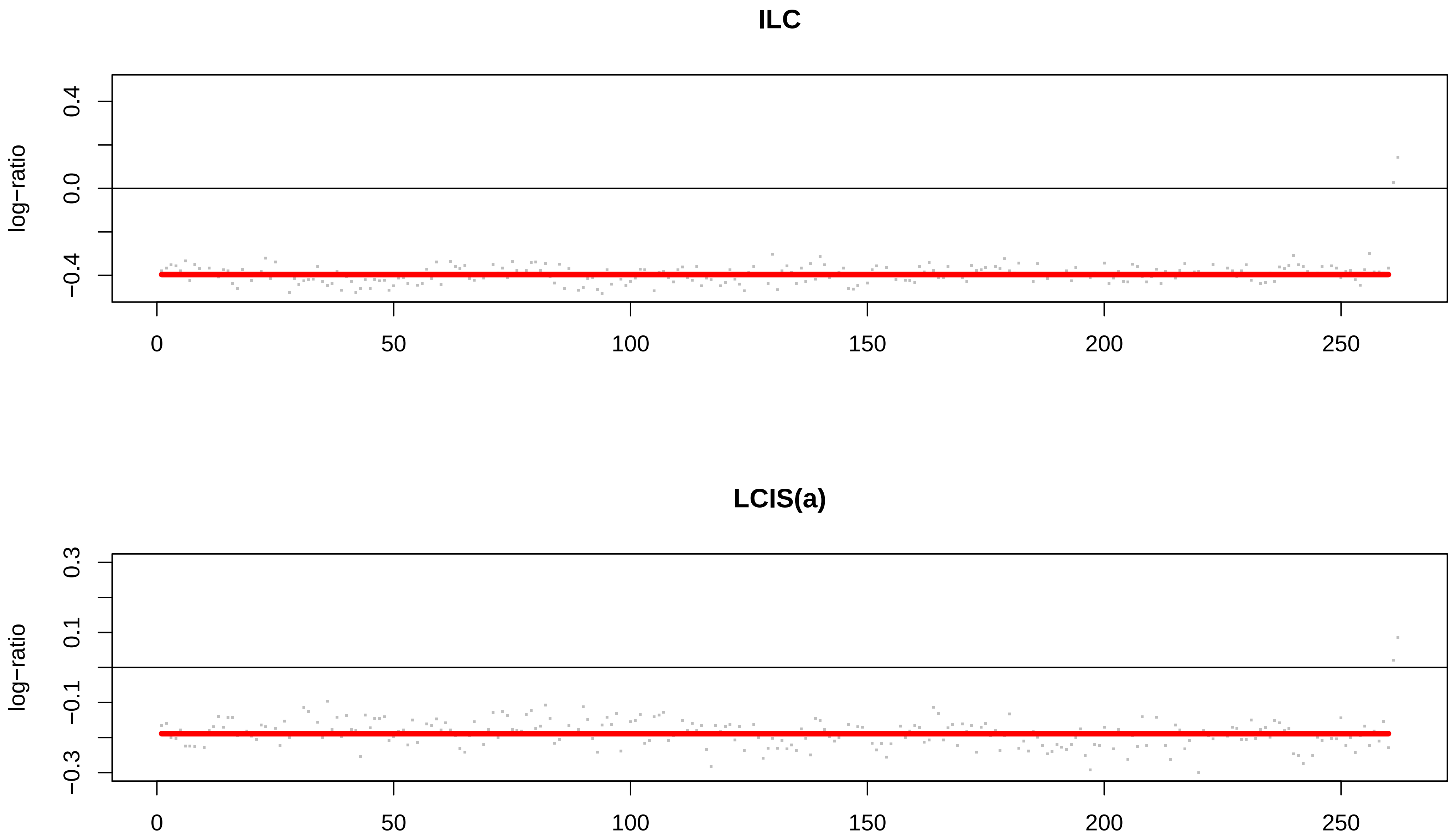

Case # 114, Chromosome 08p
Odds in favor of clonality = 71.4

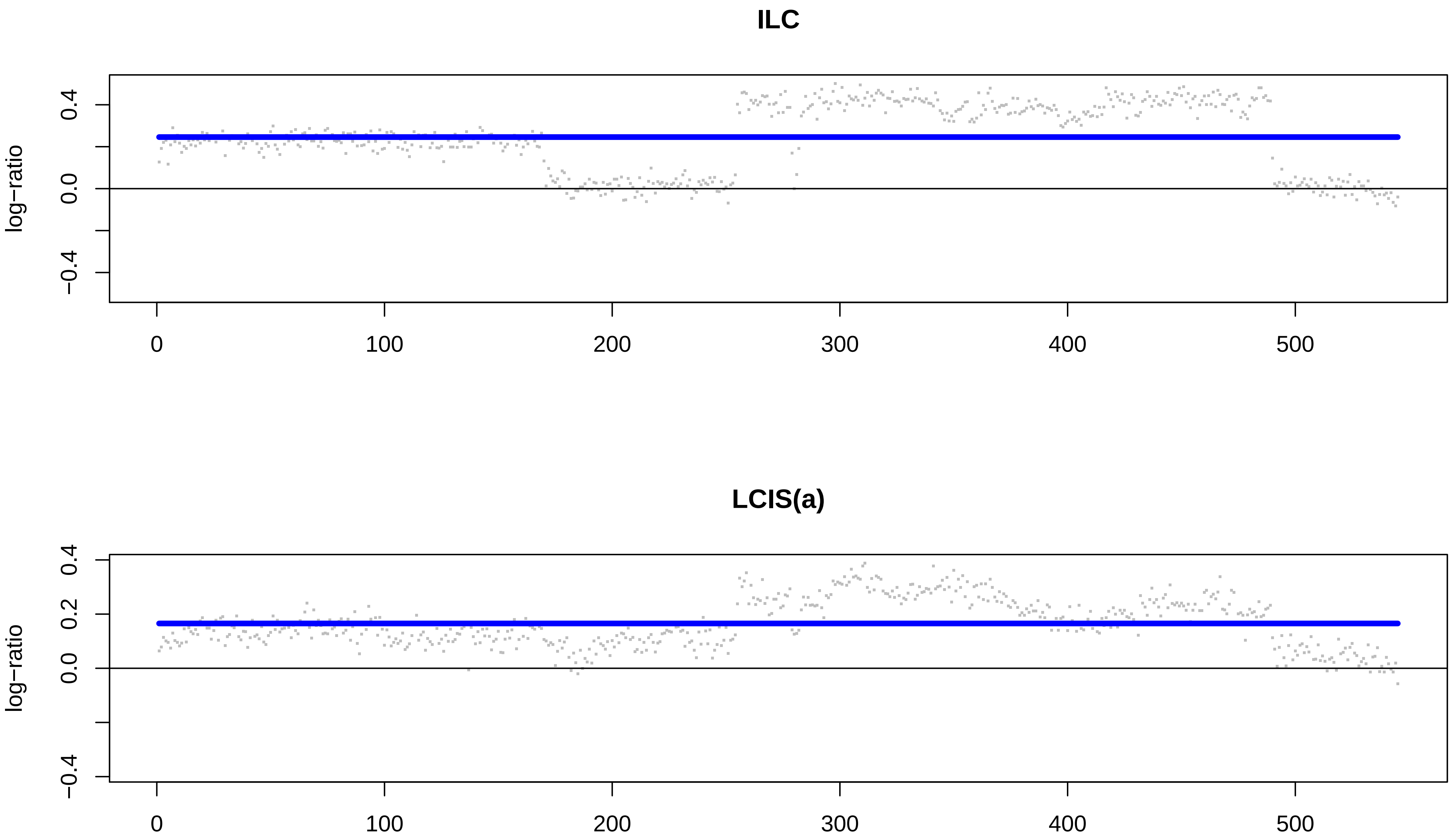

Case # 114, Chromosome 08q

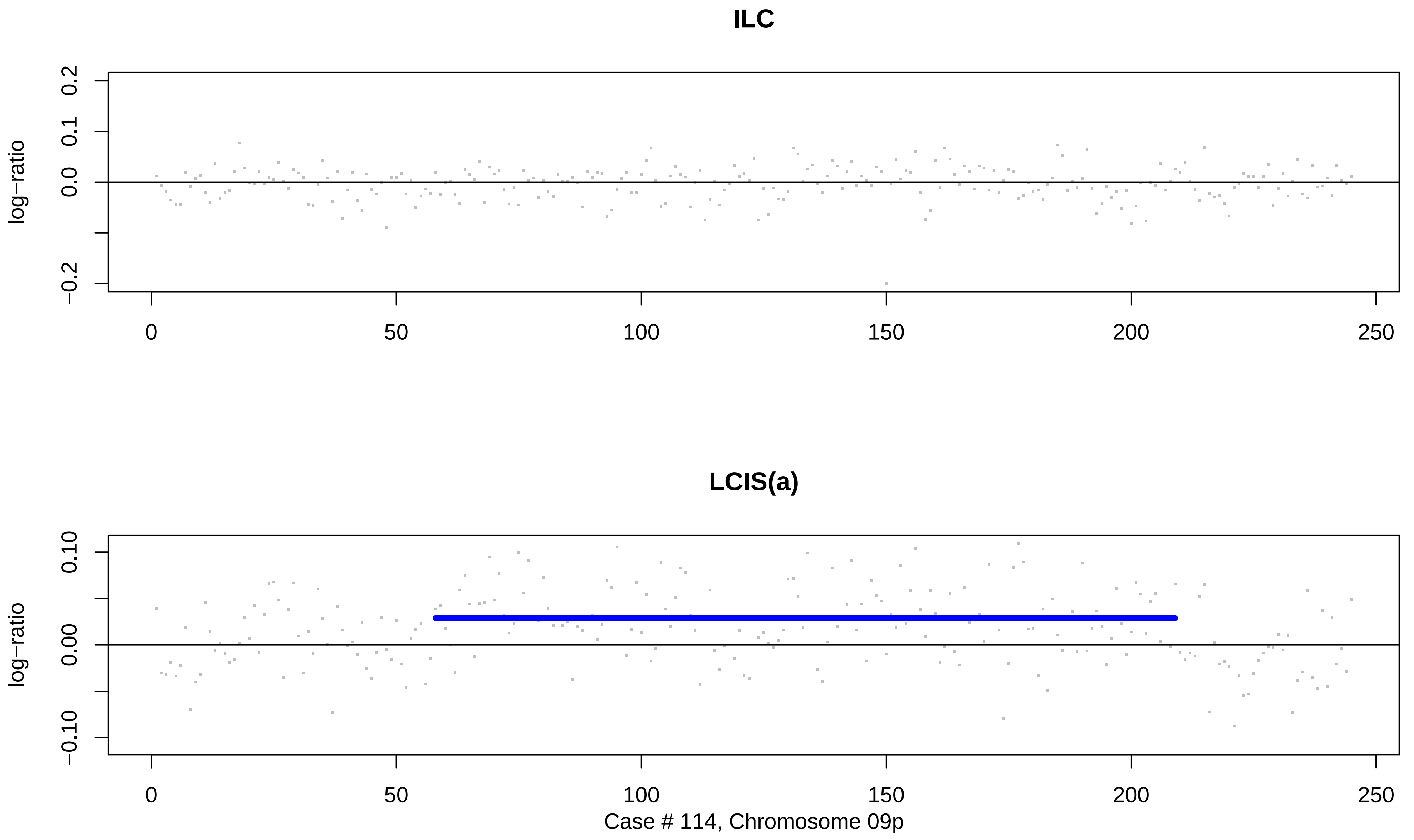

ILC
log−ratio
0.2
0.1
0.0
−0.2
0 50 100 150 200 250
LCIS(a)
log−ratio
0.10
0.00
−0.10
0 50 100 150 200 250
Case # 114, Chromosome 09p

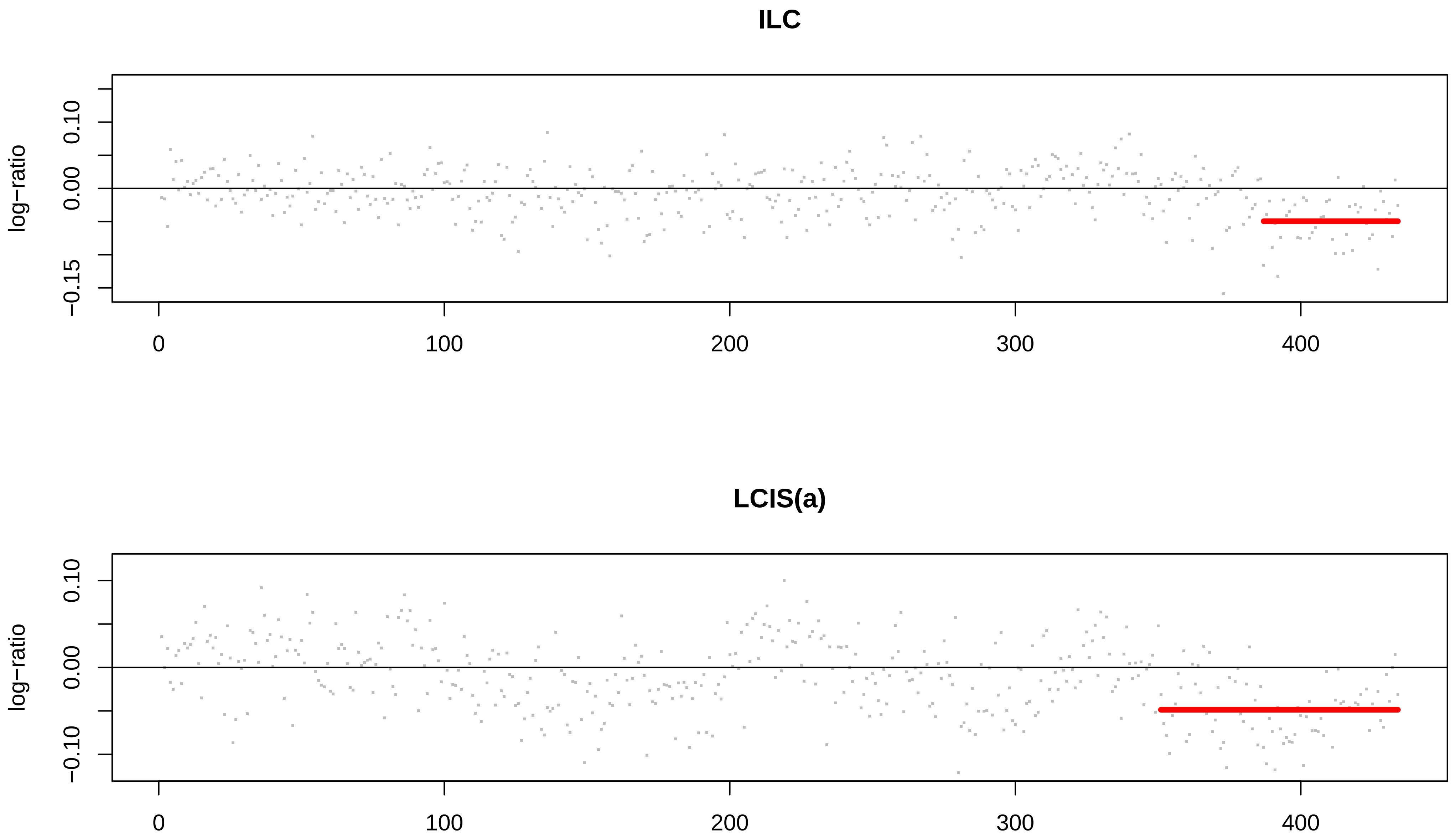

Case # 114, Chromosome 09q
Odds in favor of independence = 1.8

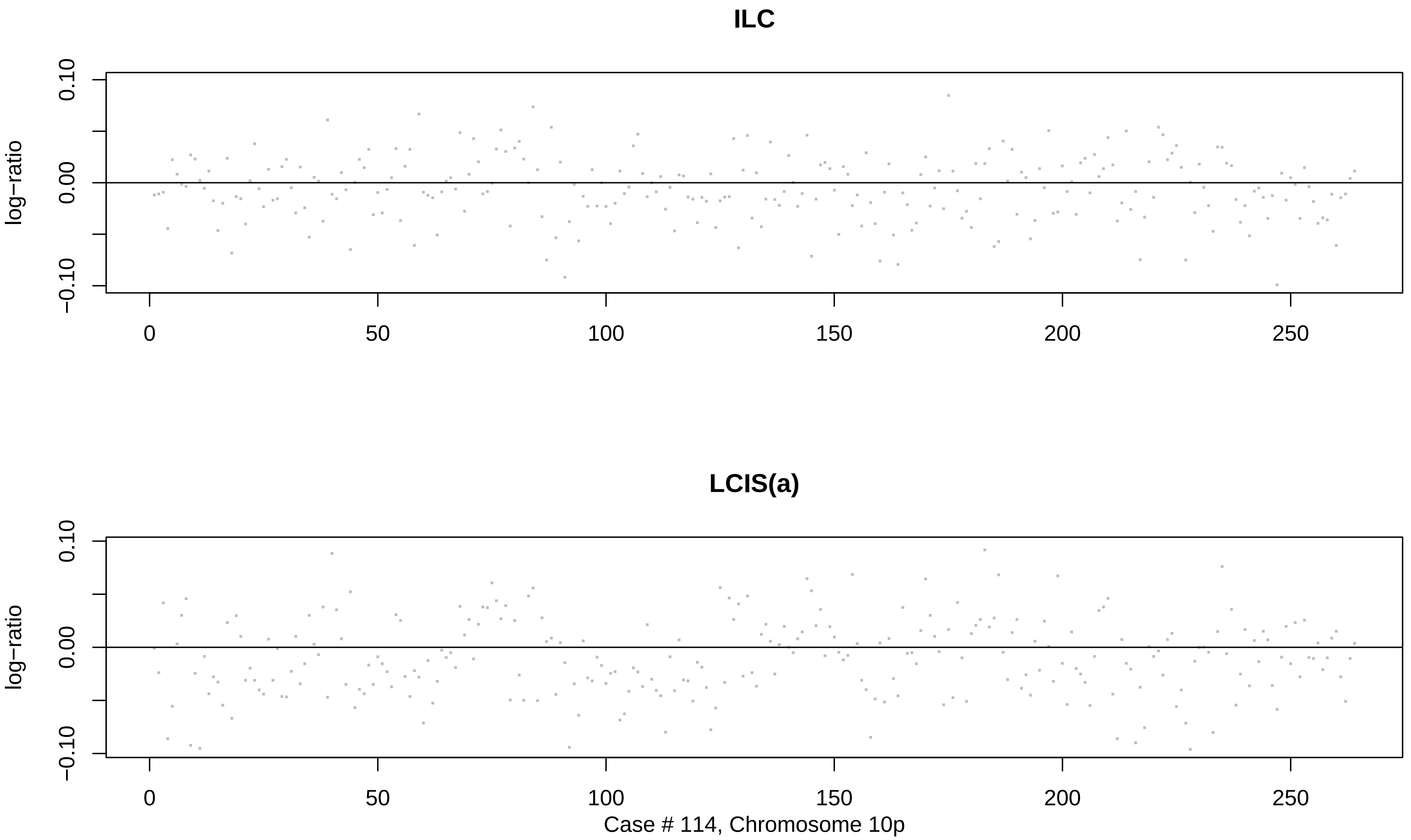

ILC
log−ratio
0.10
0.00
−0.10
0
50
100
150
200
250
LCIS(a)
log−ratio
0.10
0.00
−0.10
0
50
100
150
200
250
Case # 114, Chromosome 10p

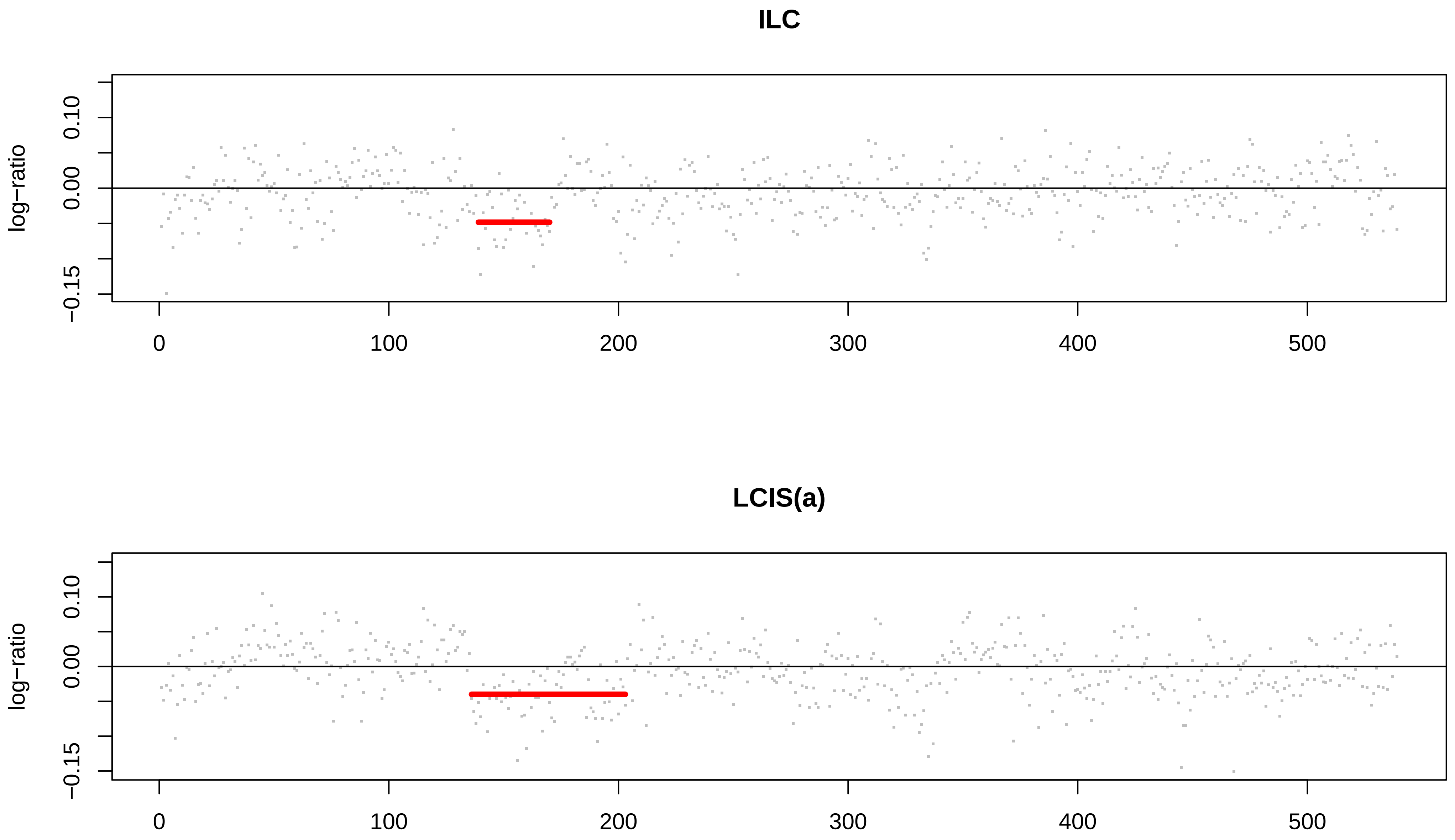

Case # 114, Chromosome 10q
Odds in favor of independence = 1.2

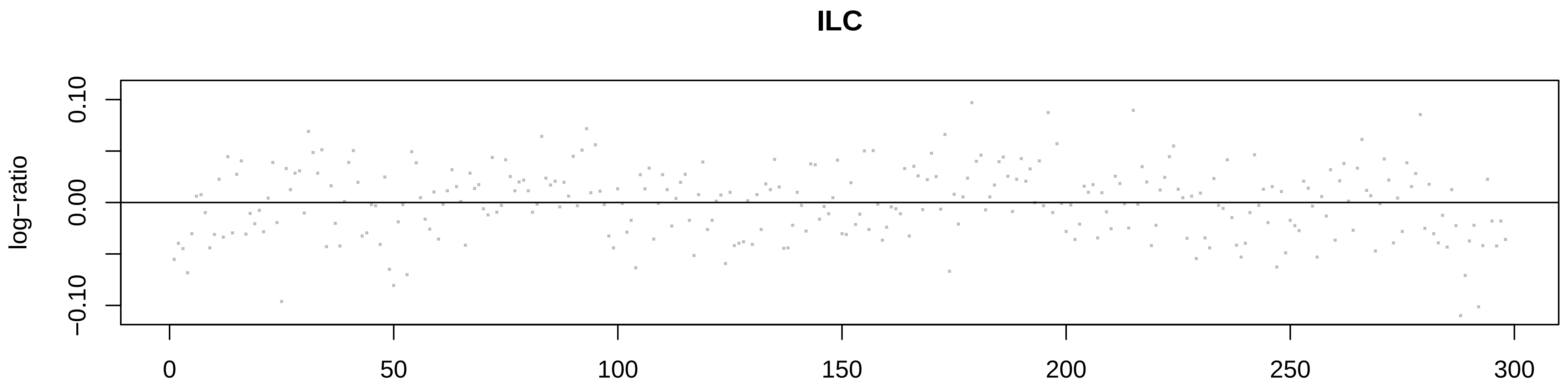
ILC
log−ratio
0.10
0.00
−0.10
0
50
100
150
200
250
300

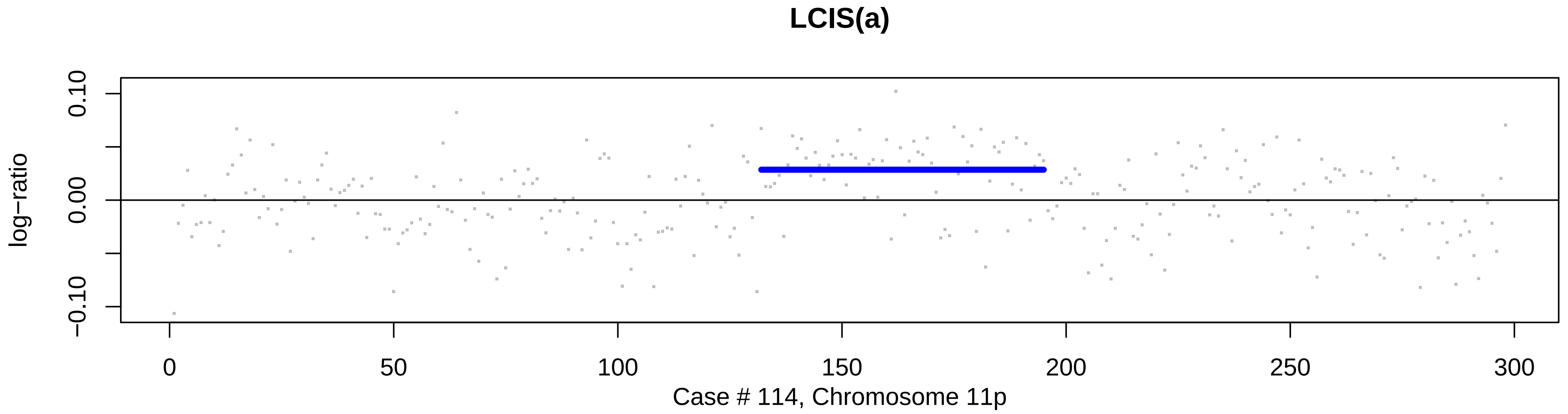
LCIS(a)
log−ratio
0.10
0.00
−0.10
0
50
100
150
200
250
300
Case # 114, Chromosome 11p

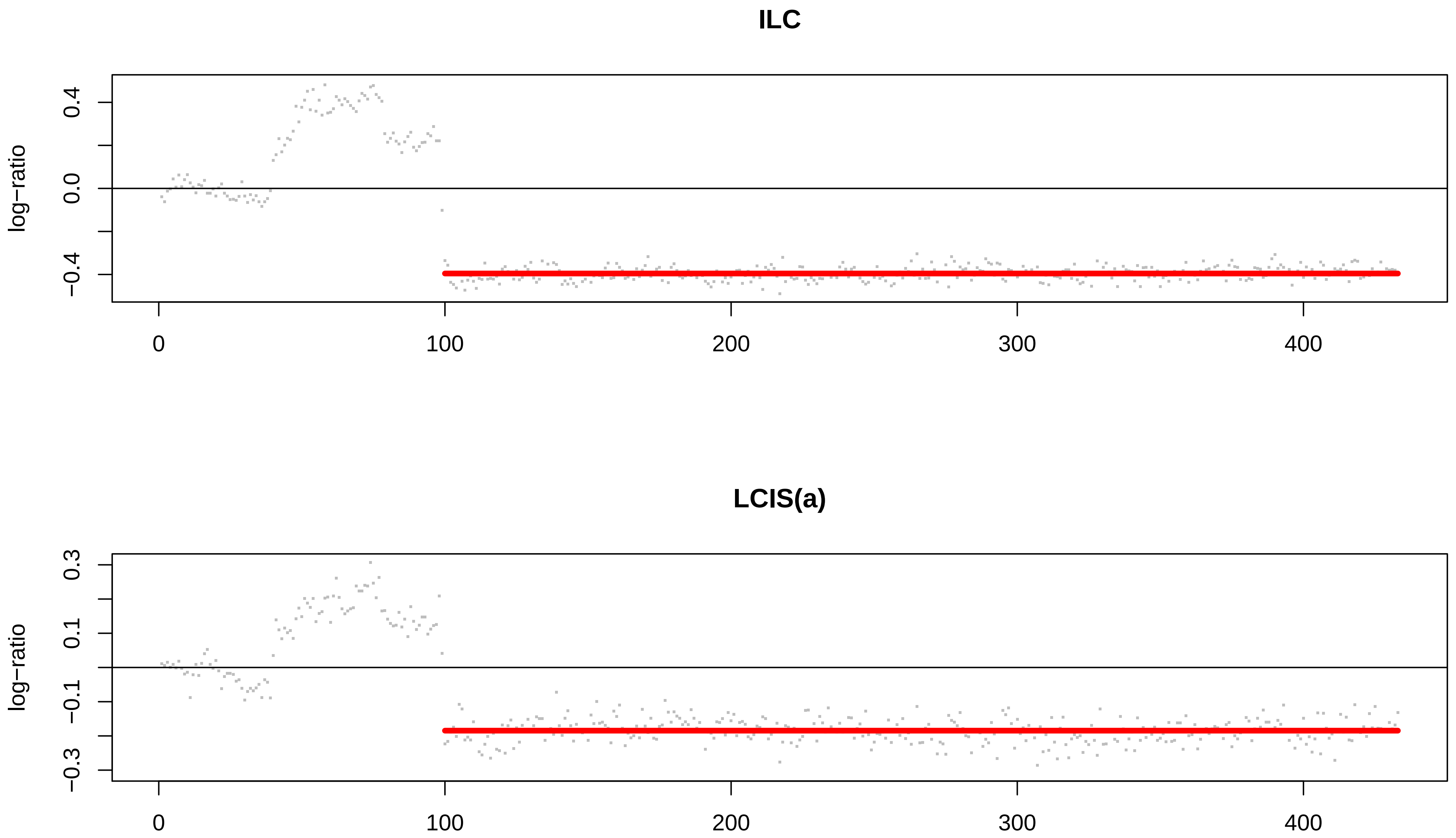

Case # 114, Chromosome 11q
Odds in favor of clonality = 1.1e+03

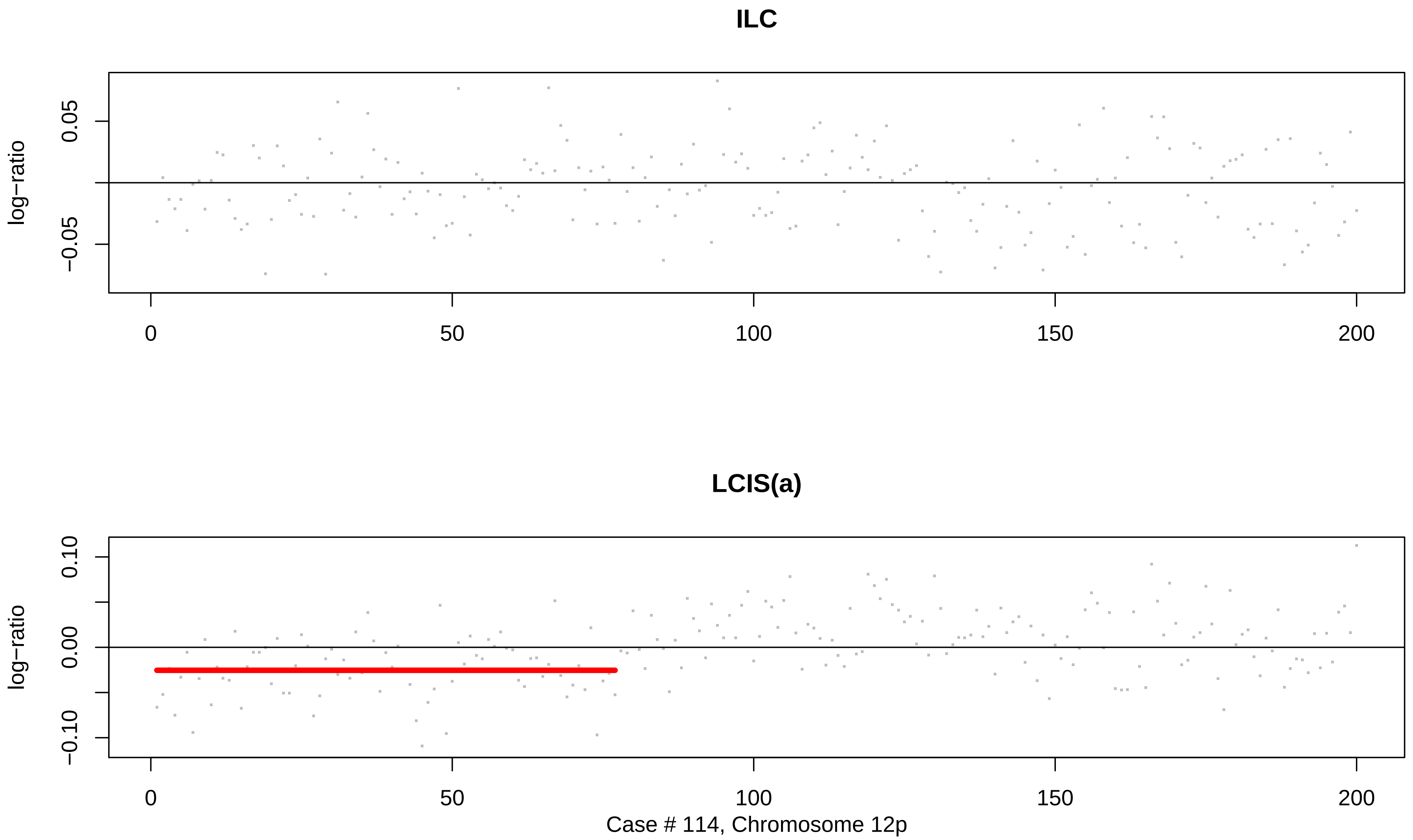
ILC
log−ratio
0.05
−0.05
0
50
100
150
200
LCIS(a)
log−ratio
0.10
0.00
−0.10
0
50
100
150
200
Case # 114, Chromosome 12p

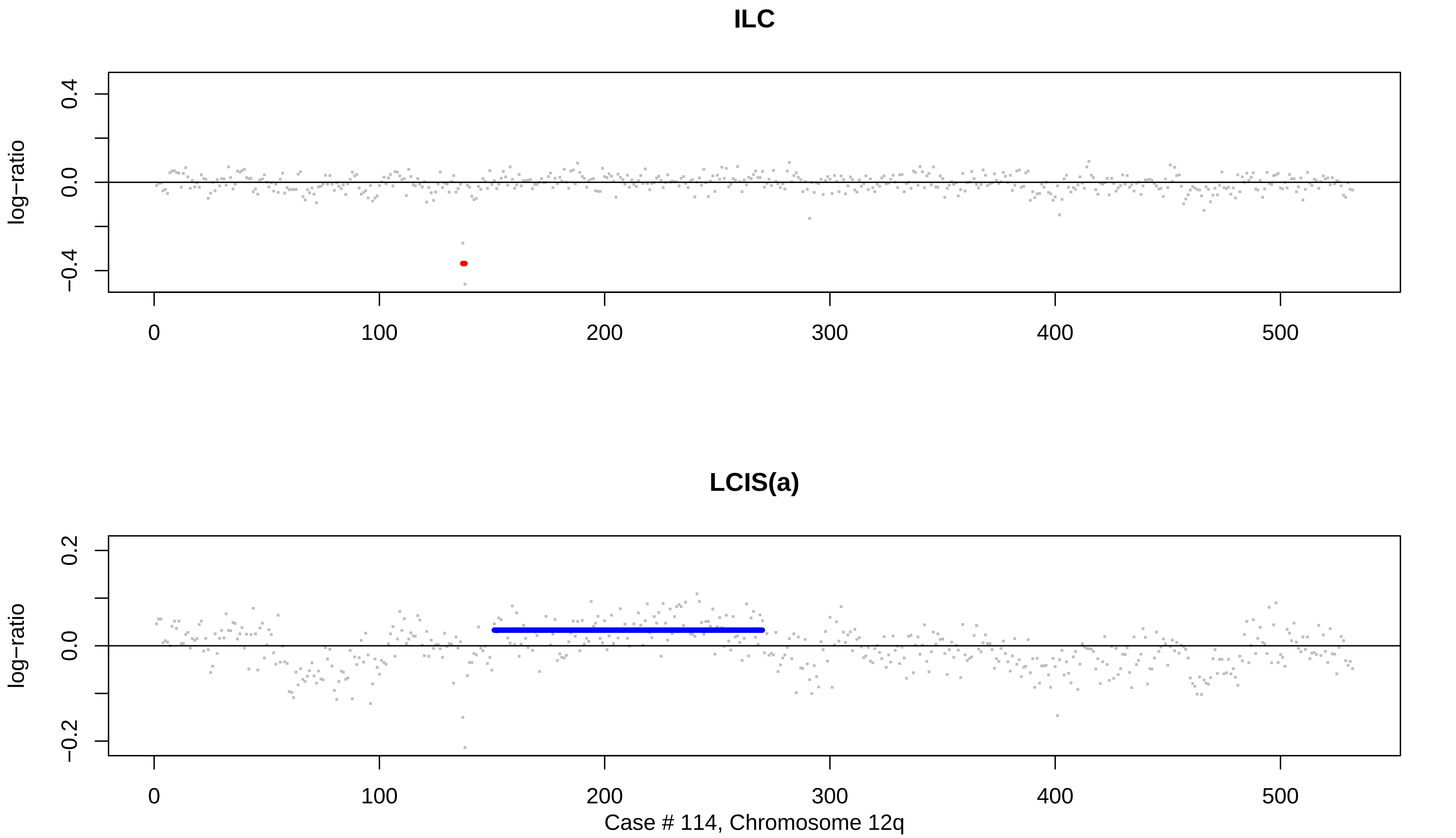
ILC
log−ratio
0.4
0.0
−0.4
0
100
200
300
400
500
LCIS(a)
log−ratio
0.2
0.0
−0.2
0
100
200
300
400
500
Case # 114, Chromosome 12q

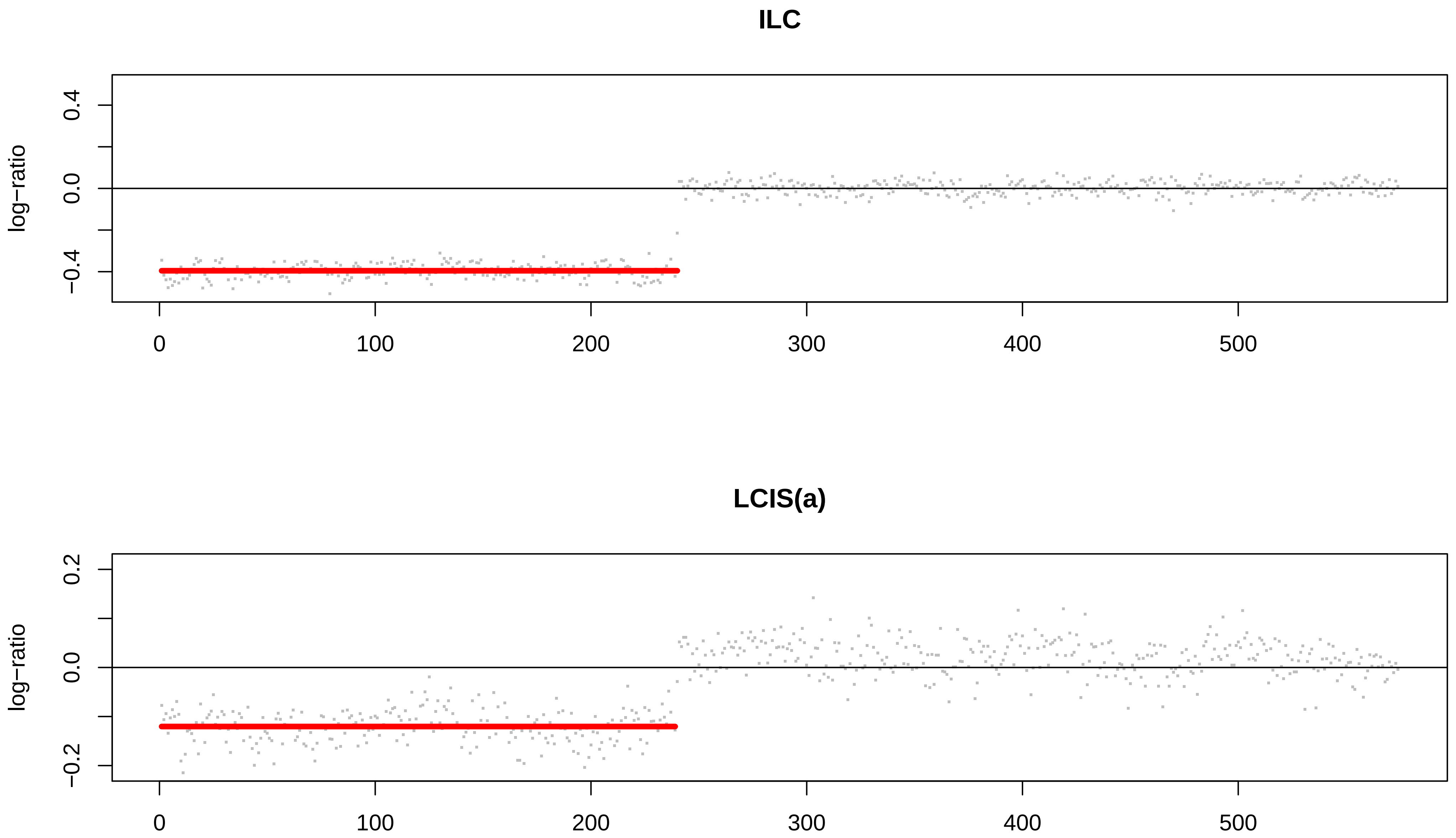

Case # 114, Chromosome 13q
Odds in favor of clonality = 67.4

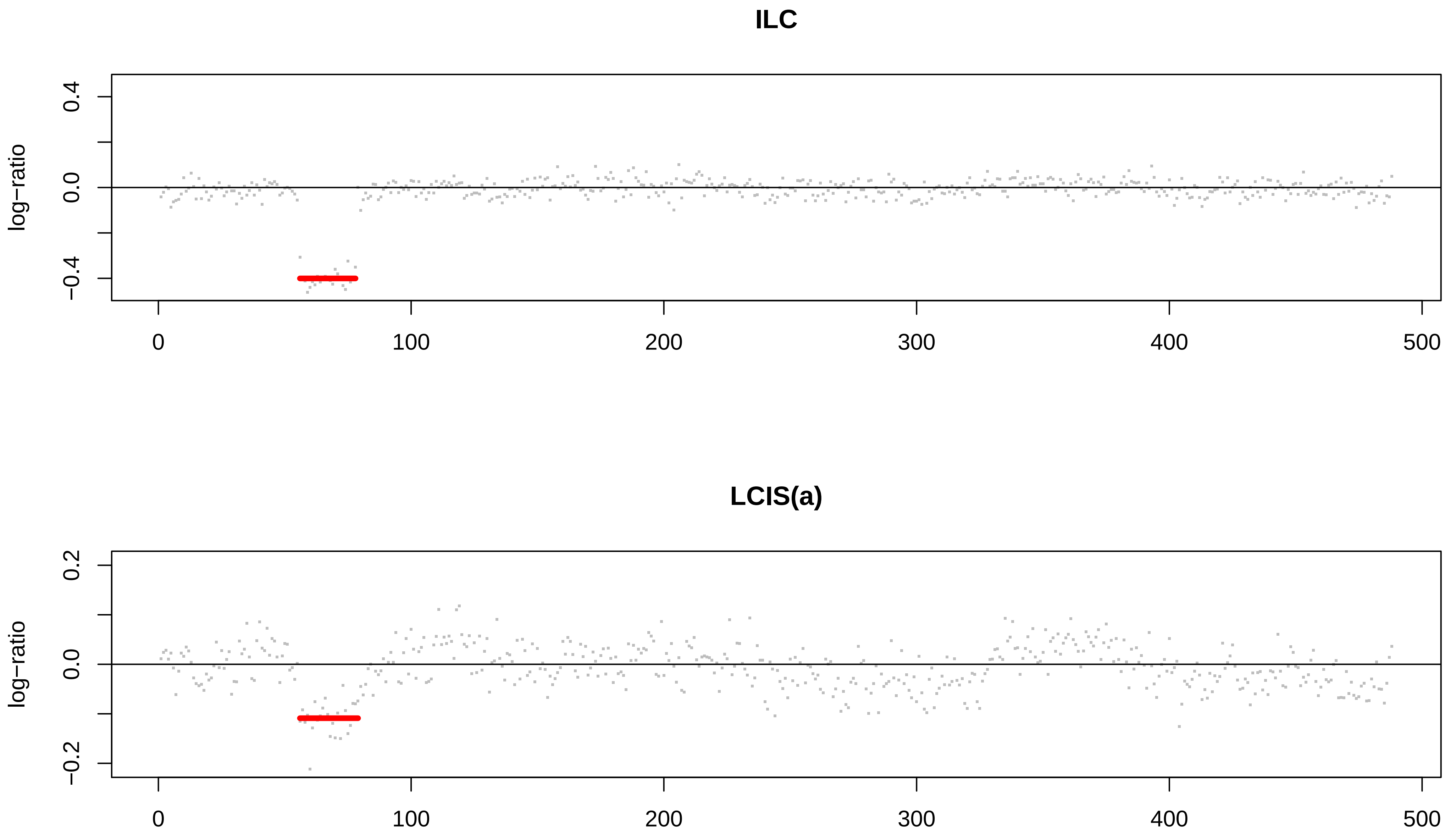

Case # 114, Chromosome 14q
Odds in favor of clonality = 1.4e+02

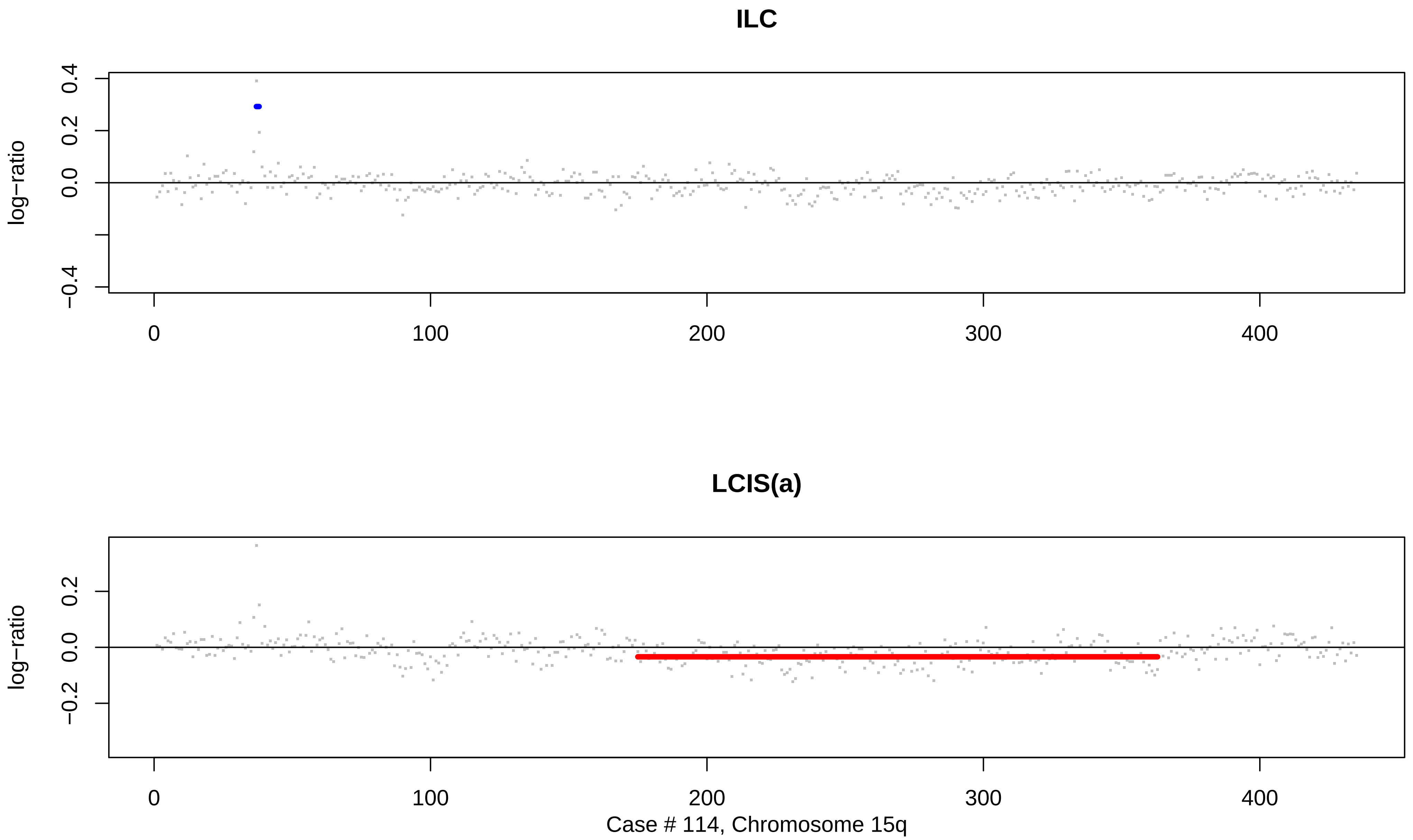
ILC
log−ratio
0.4
0.2
0.0
−0.4
0
100
200
300
400
LCIS(a)
log−ratio
0.2
0.0
−0.2
0
100
200
300
400
Case # 114, Chromosome 15q

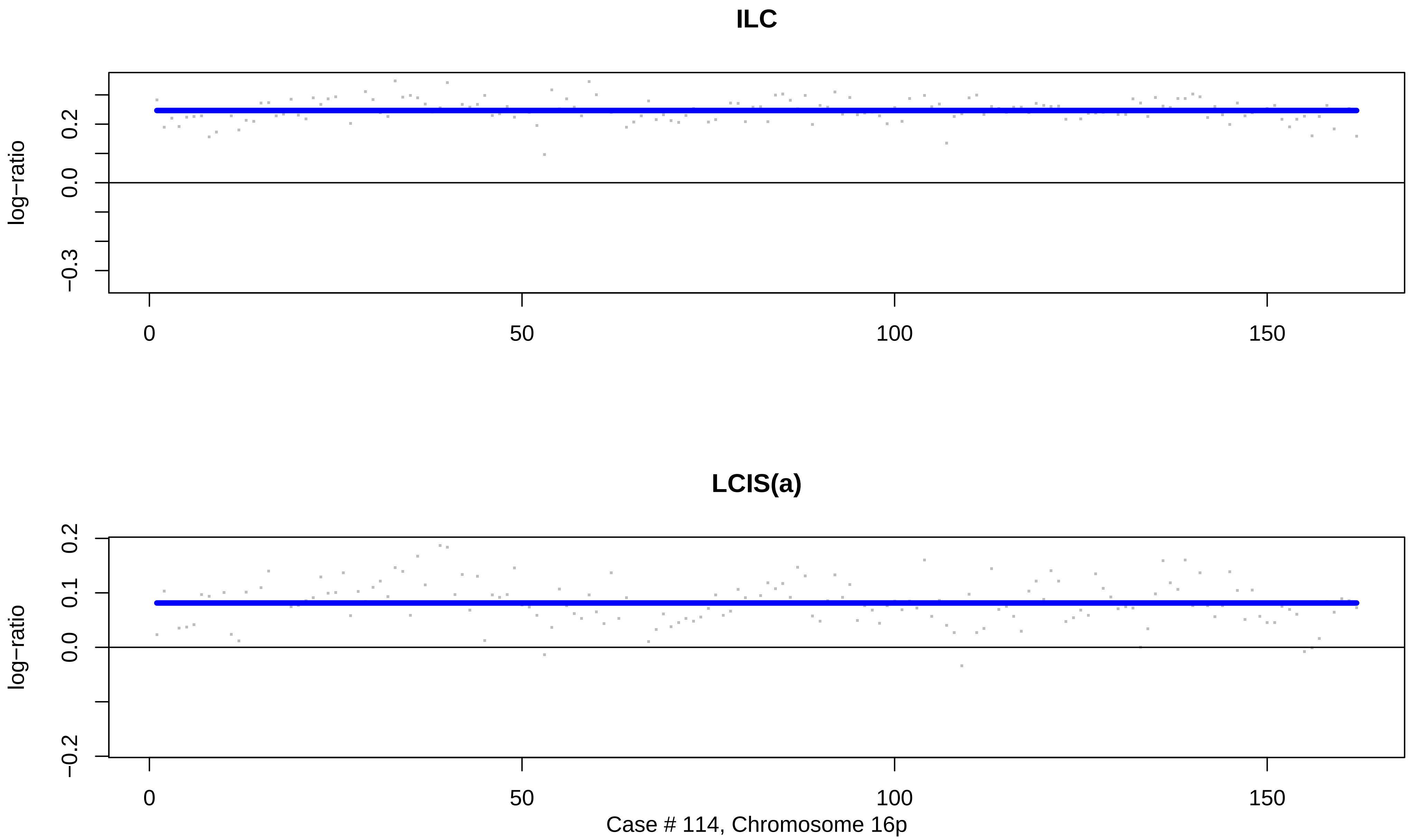
ILC
log−ratio
0.2
0.0
−0.3
0
50
100
150
LCIS(a)
log−ratio
0.2
0.1
0.0
−0.2
0
50
100
150
Case # 114, Chromosome 16p

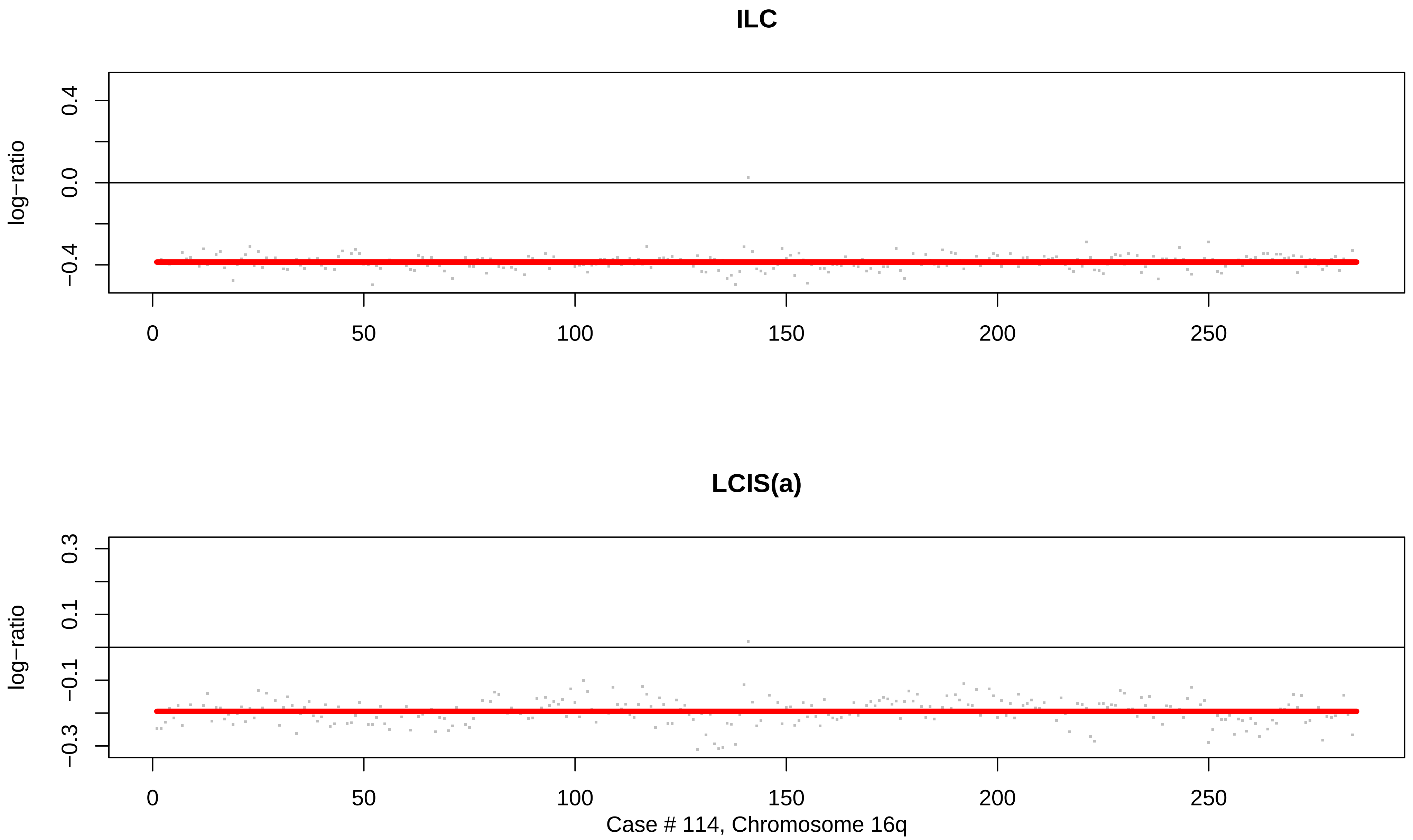
ILC
log−ratio
0.4
0.0
−0.4
0
50
100
150
200
250
LCIS(a)
log−ratio
0.3
0.1
−0.1
−0.3
0
50
100
150
200
250
Case # 114, Chromosome 16q

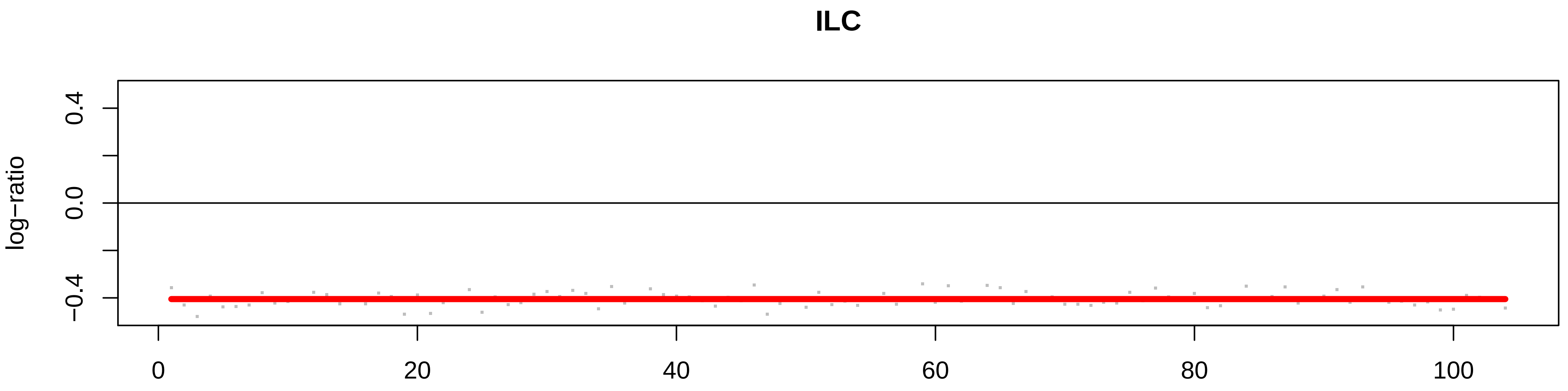
ILC
log−ratio
0.4
0.0
−0.4
0
20
40
60
80
100

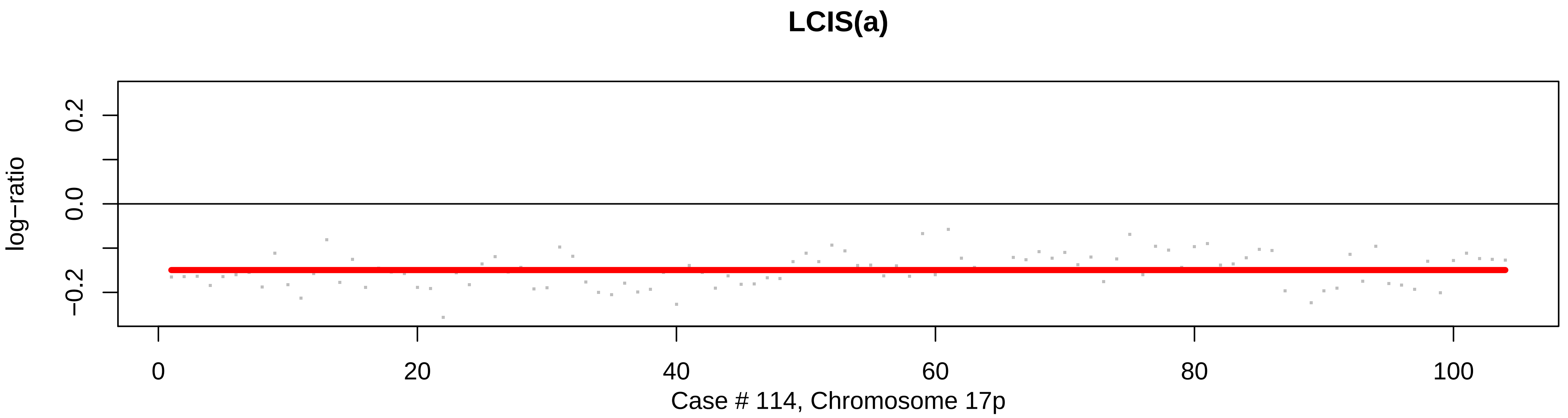
LCIS(a)
log−ratio
0.2
0.0
−0.2
0
20
40
60
80
100
Case # 114, Chromosome 17p

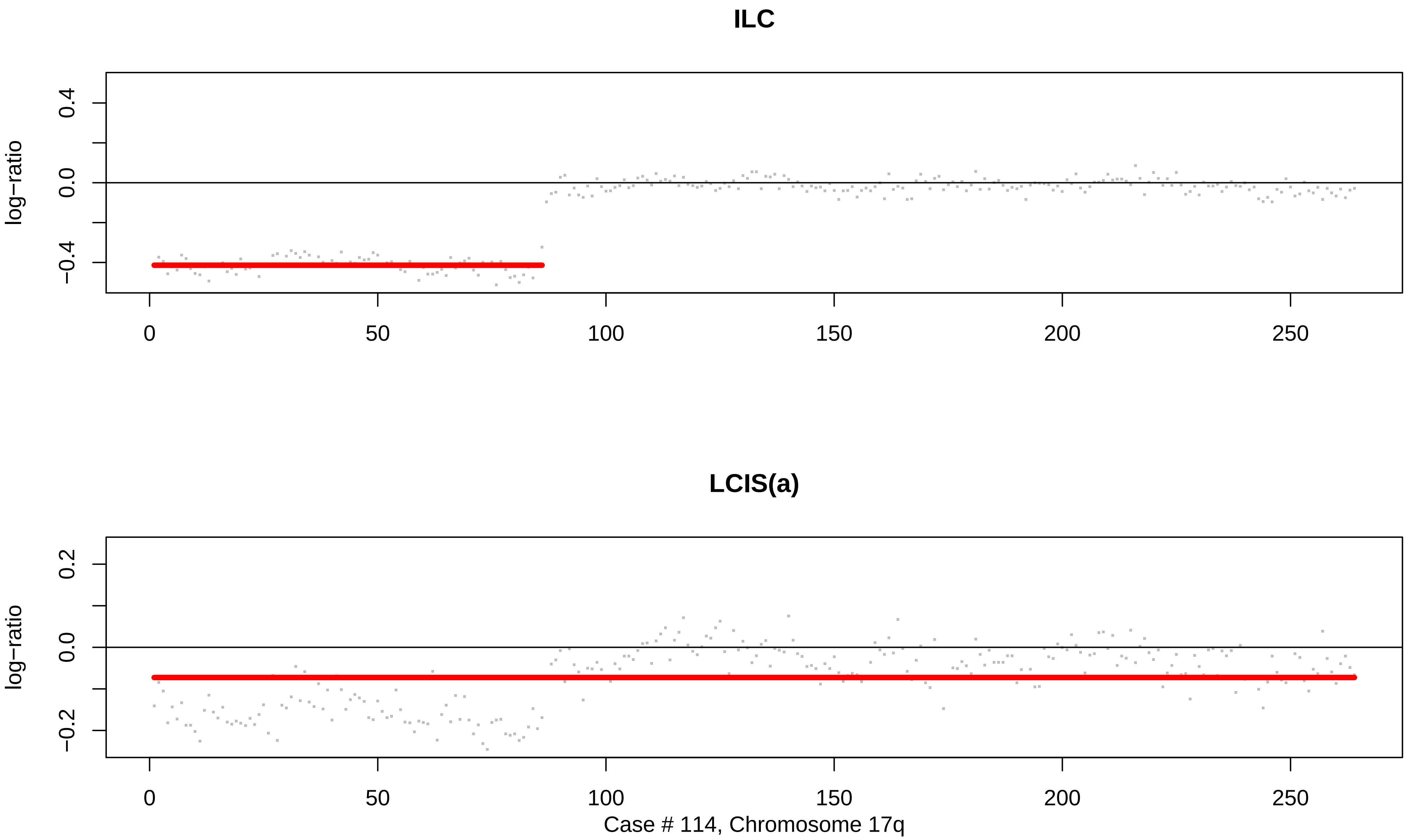
ILC
log−ratio
0.4
0.0
−0.4
0
50
100
150
200
250
LCIS(a)
log−ratio
0.2
0.0
−0.2
0
50
100
150
200
250
Case # 114, Chromosome 17q

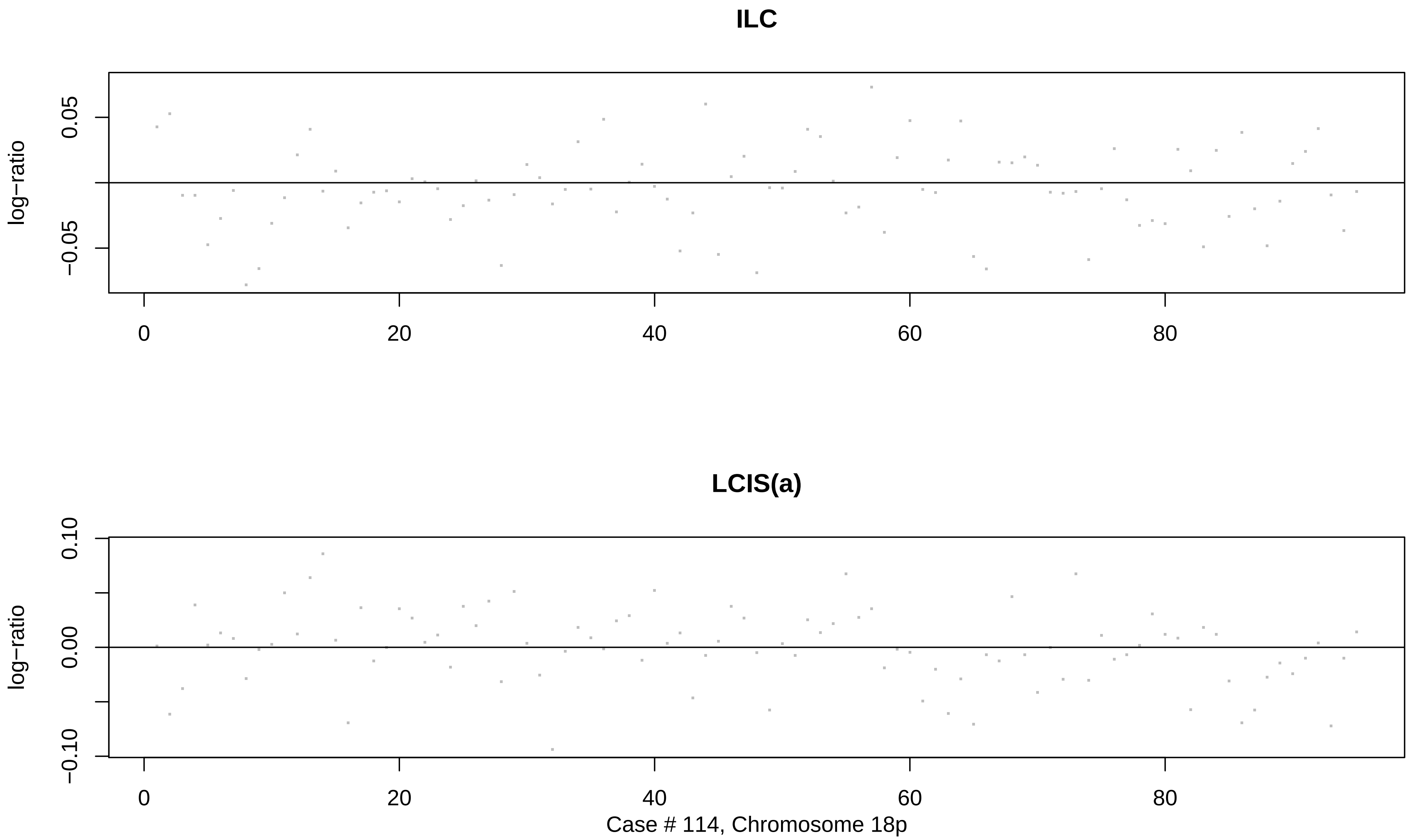

ILC
log−ratio
0.05
−0.05
0
20
40
60
80
LCIS(a)
log−ratio
0.10
0.00
−0.10
0
20
40
60
80
Case # 114, Chromosome 18p

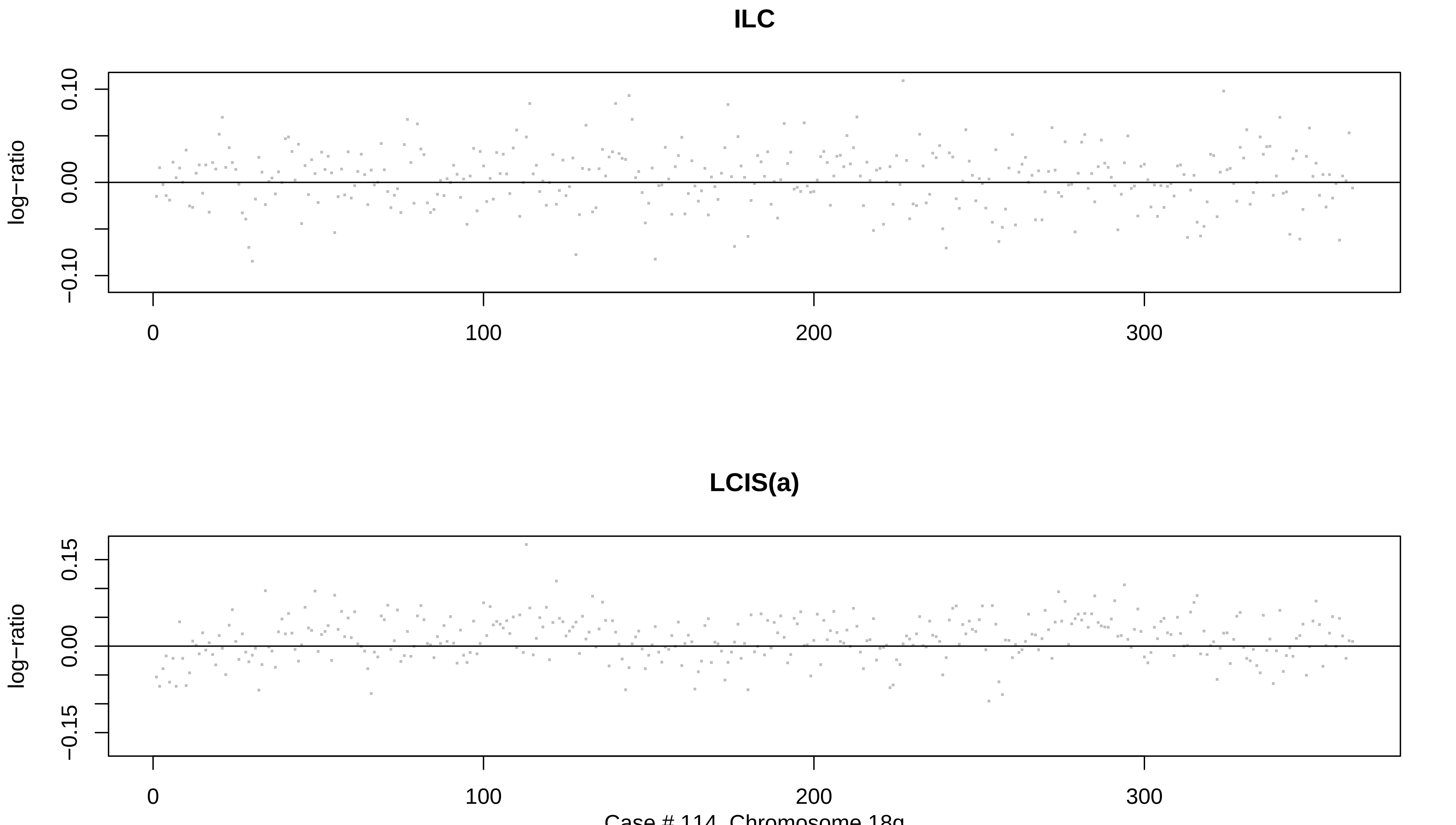
ILC
log−ratio
0.10
0.00
−0.10
0
100
200
300
LCIS(a)
log−ratio
0.15
0.00
−0.15
0
100
200
300
Case # 114, Chromosome 18q

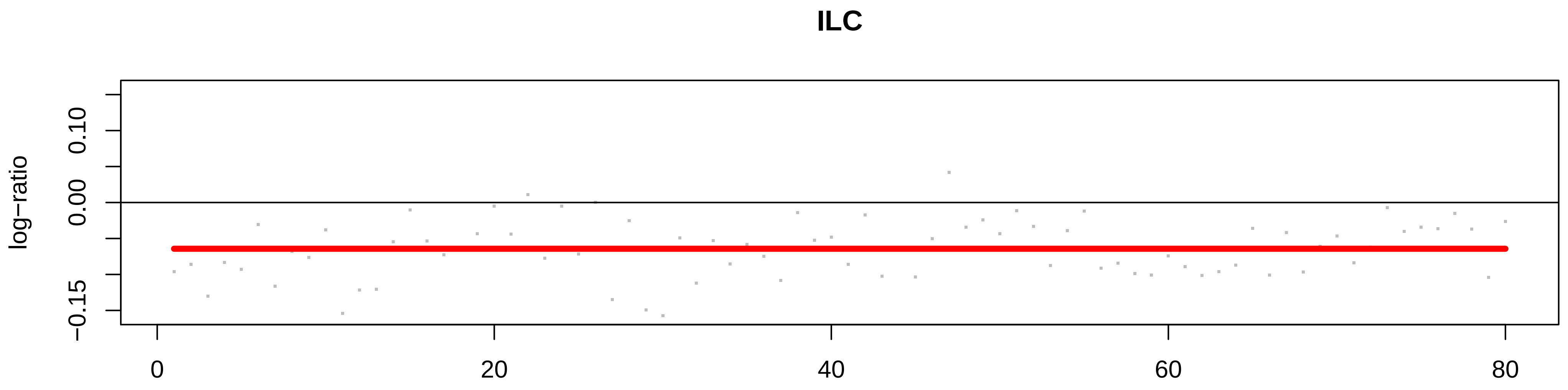
ILC
log−ratio
0.10
0.00
−0.15
0
20
40
60
80

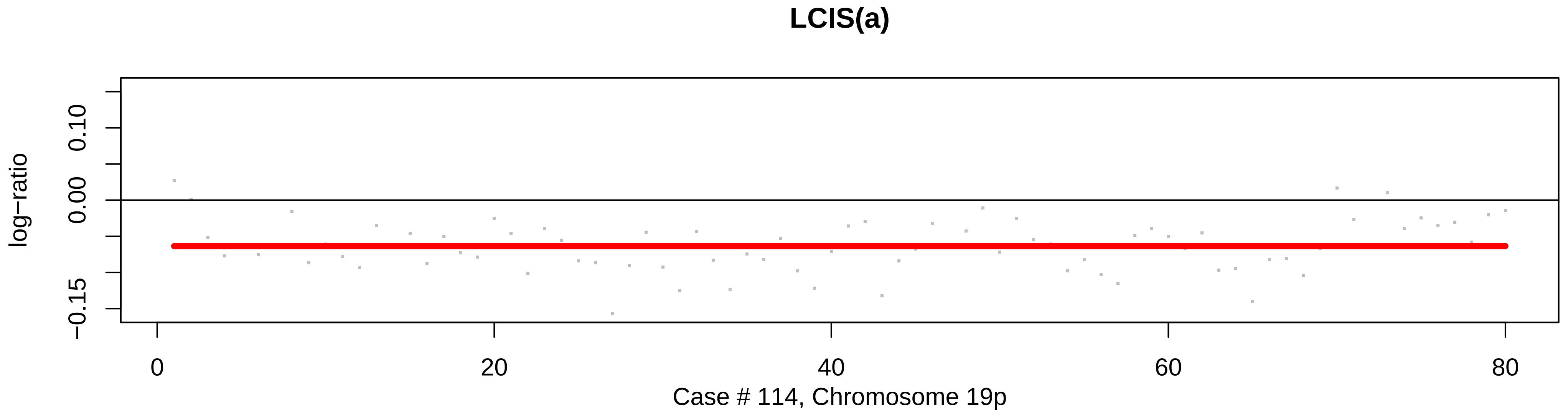
LCIS(a)
log−ratio
0.10
0.00
−0.15
0
20
40
60
80
Case # 114, Chromosome 19p

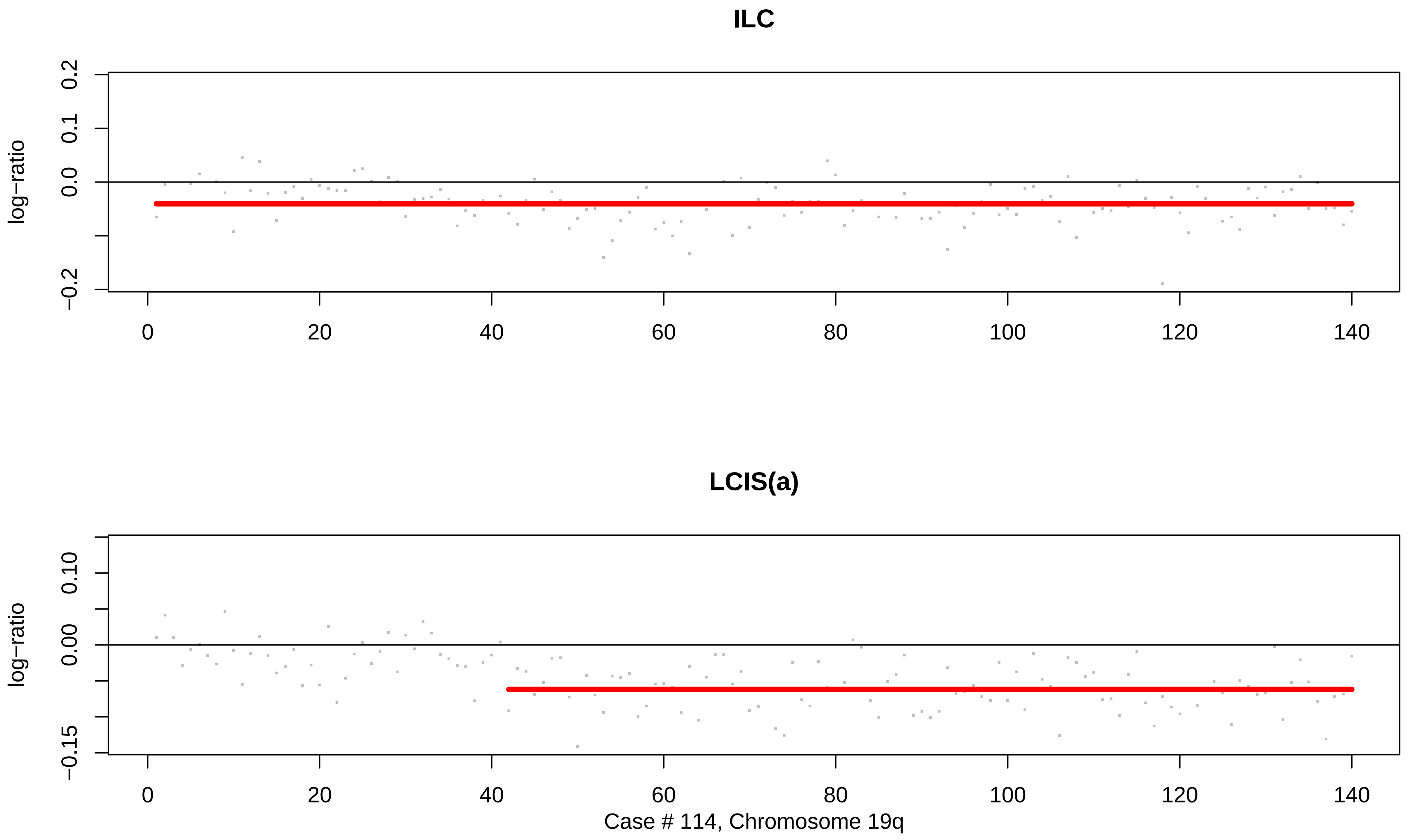
ILC
log−ratio
0.2
0.1
0.0
−0.2
0
20
40
60
80
100
120
140
LCIS(a)
log−ratio
0.10
0.00
−0.15
0
20
40
60
80
100
120
140
Case # 114, Chromosome 19q

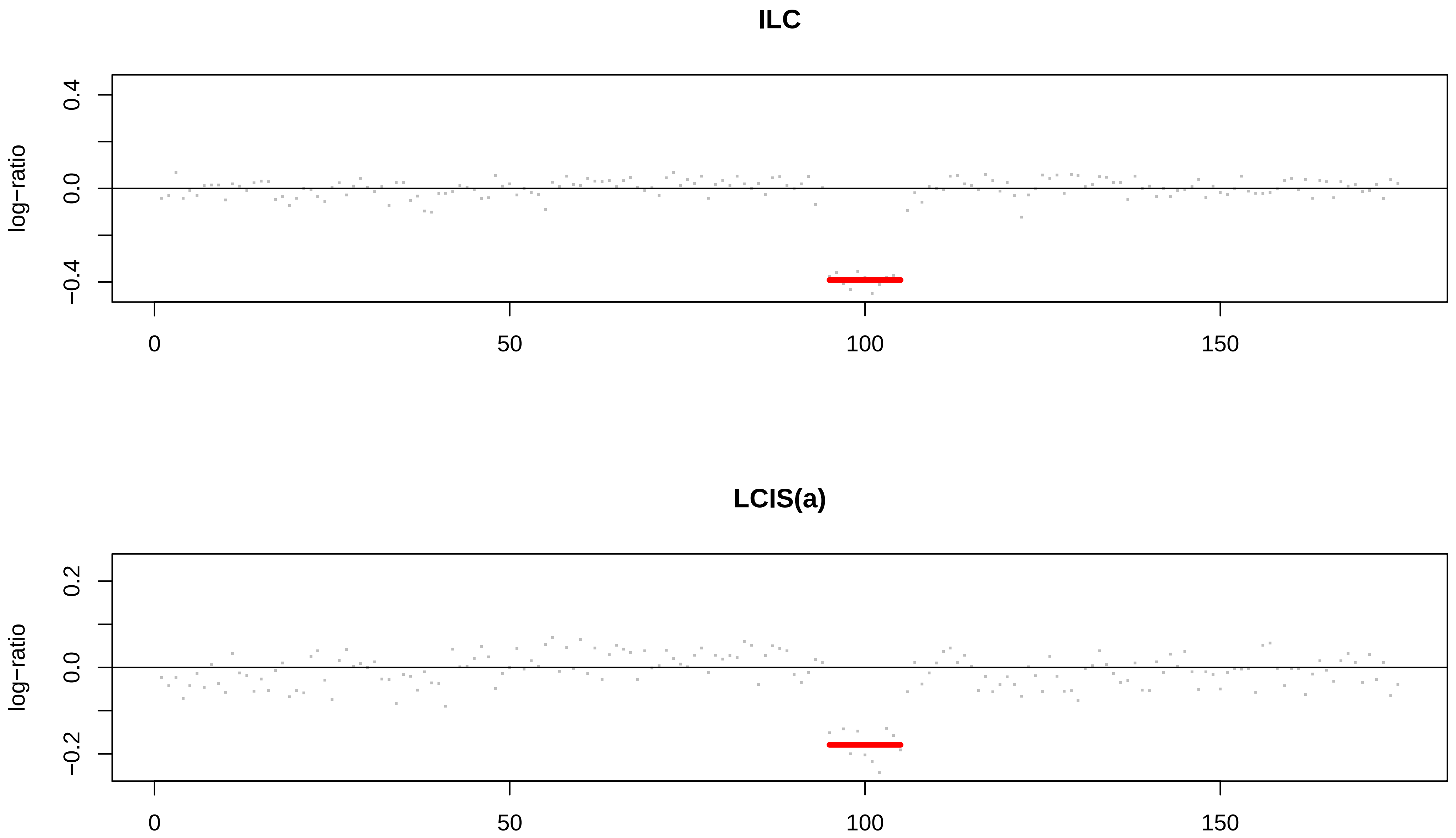

Case # 114, Chromosome 20p
Odds in favor of clonality = 3e+02

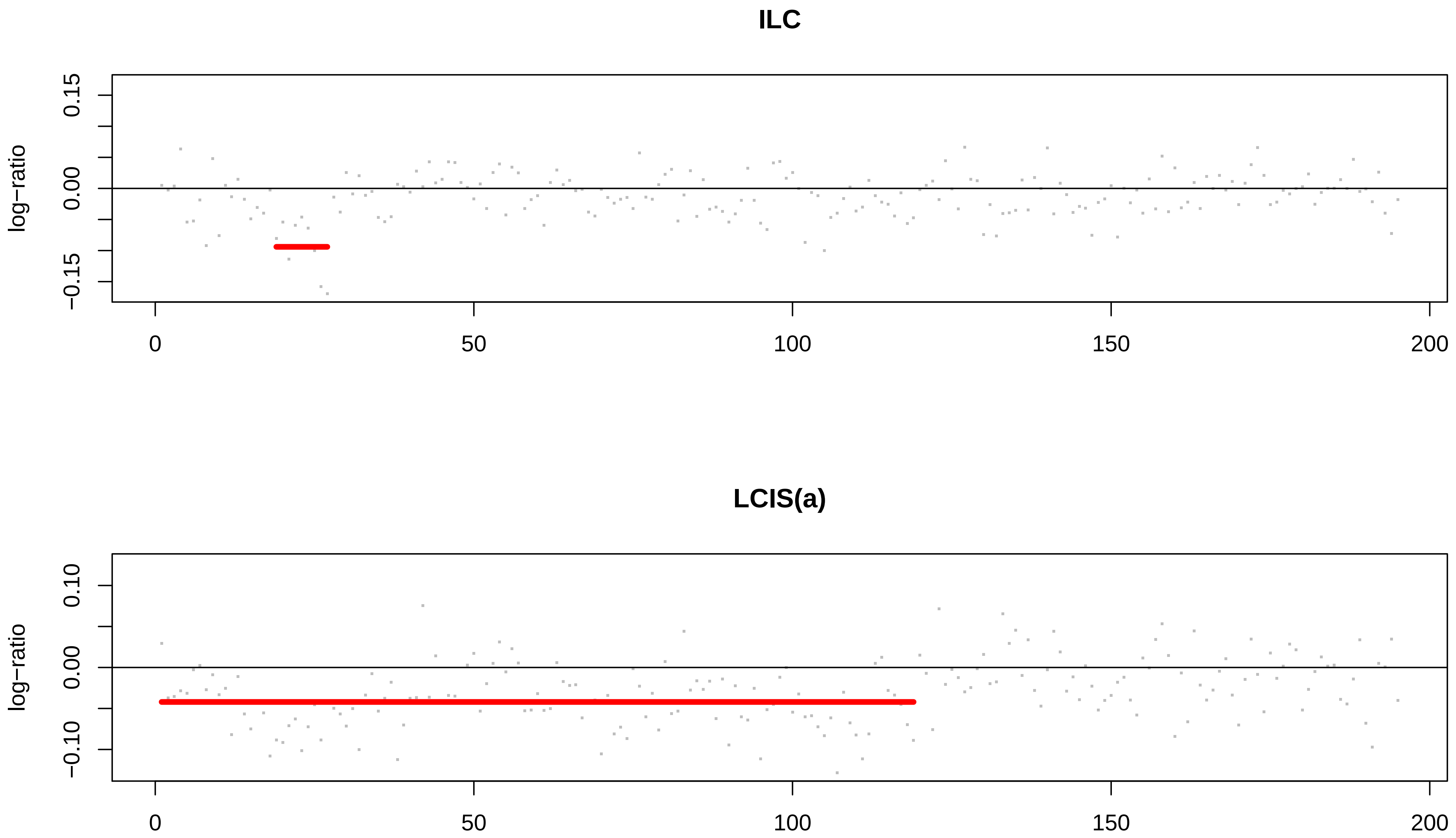

Case # 114, Chromosome 20q
Odds in favor of independence = 3

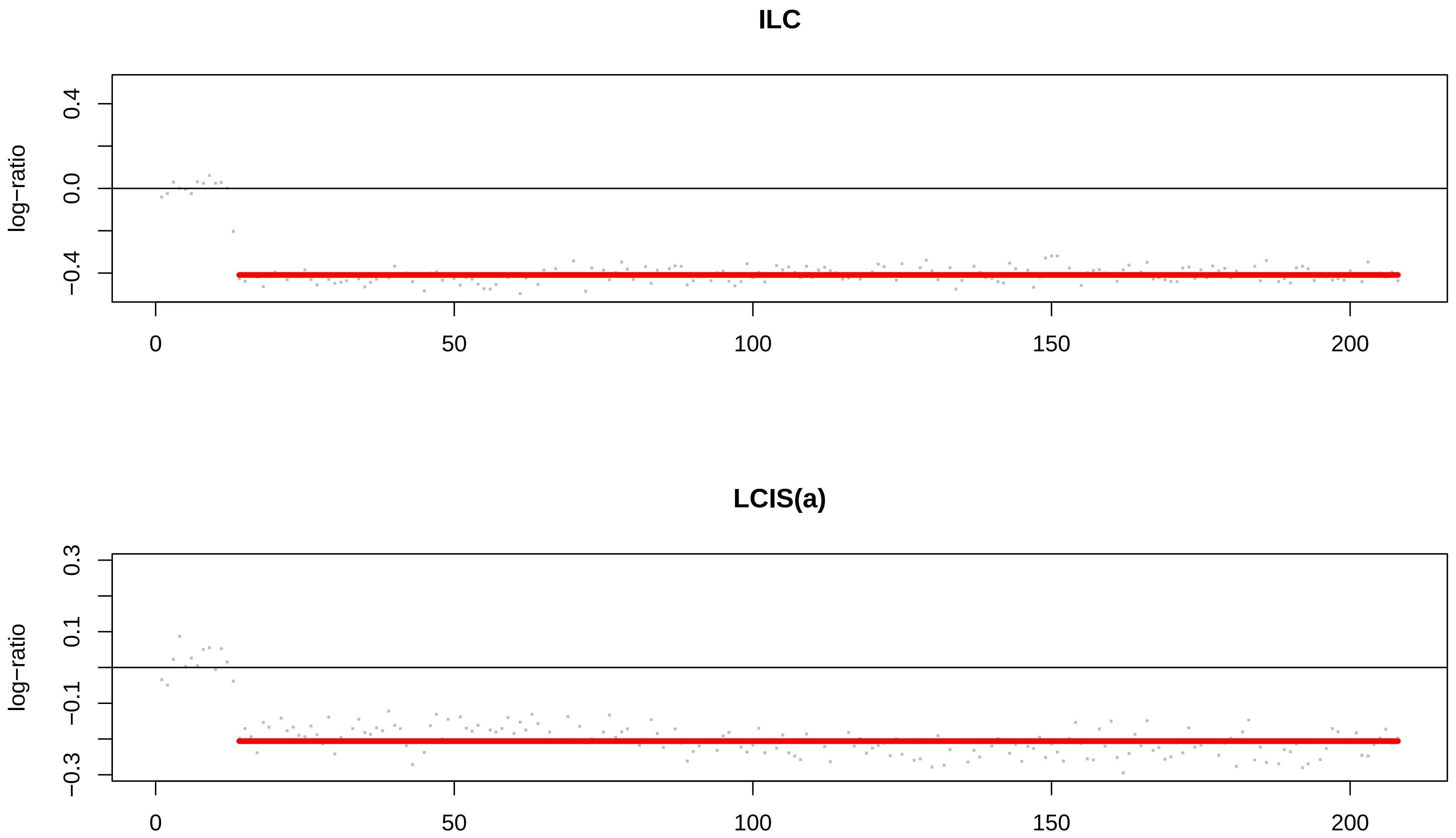

Case # 114, Chromosome 21q
Odds in favor of clonality = 4e+02

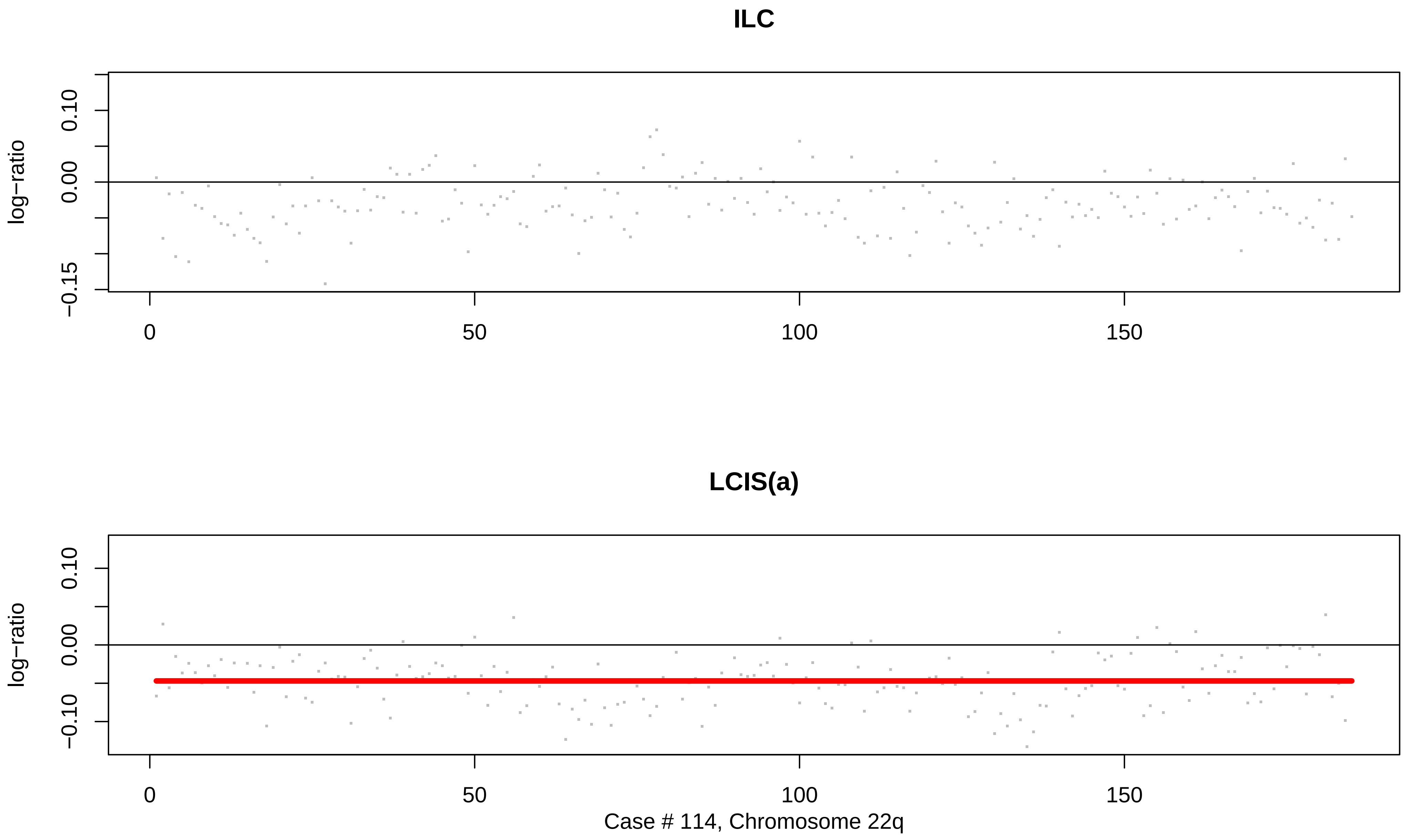

ILC
log−ratio
0.10
0.00
−0.15
0
50
100
150
LCIS(a)
log−ratio
0.10
0.00
−0.10
0
50
100
150
Case # 114, Chromosome 22q
