# Supplementary material for: Clonal relatedness between lobular carcinoma in situ and synchronous malignant lesions
Source: Breast Cancer Res. 2012 Jul 9;14(4):R103. doi: 10.1186/bcr3222 (PMC3680923; doi:10.1186/bcr3222)
Supplement: Additional file 4 — Magnified version of genome-wide plots with detailed marker plots and segmentation on a chromosome-arm-specific basis. [file bcr3222-S4.ZIP › Case 110 ID.pdf]

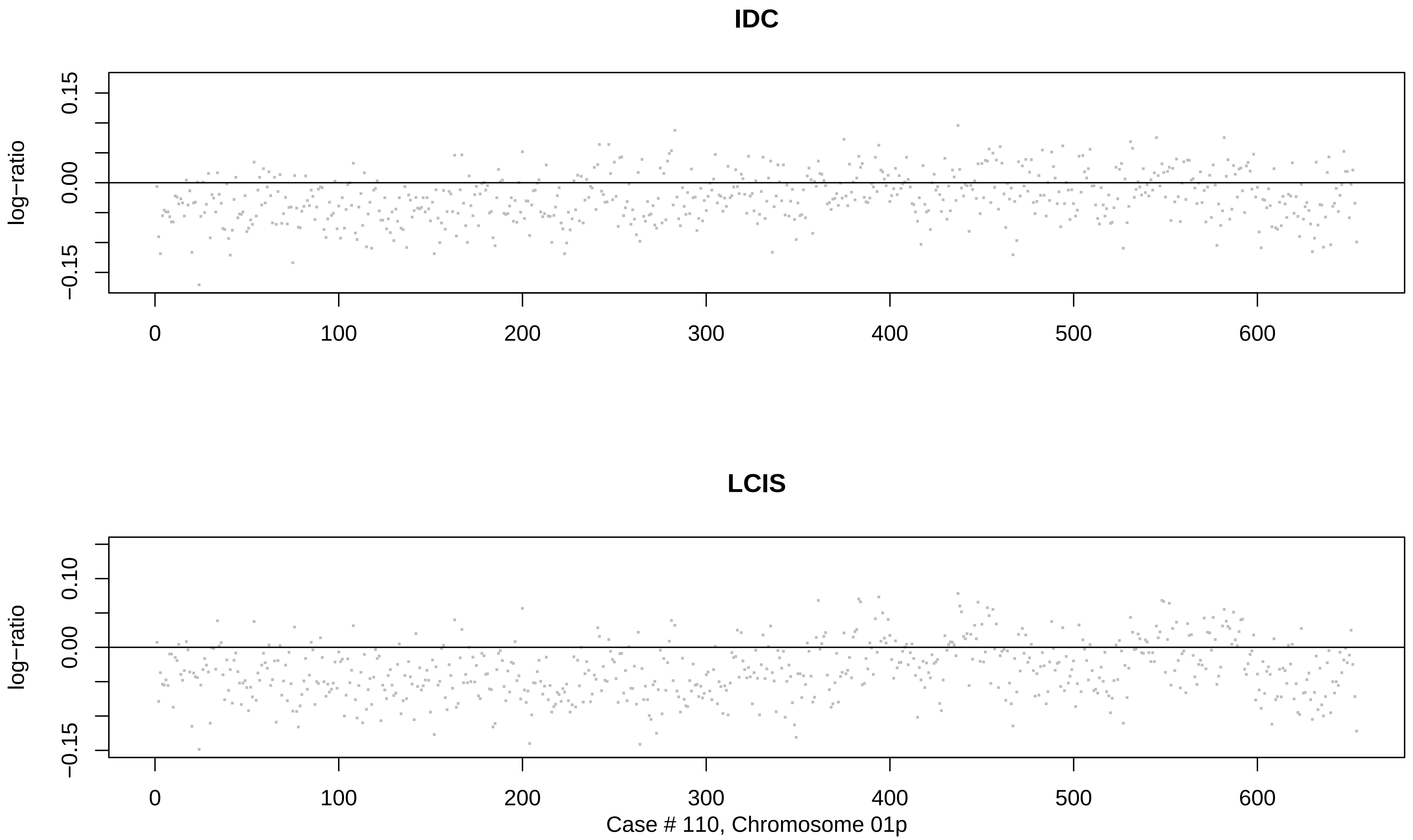
IDC
log−ratio
0.15
0.00
−0.15
0
100
200
300
400
500
600
LCIS
log−ratio
0.10
0.00
−0.15
0
100
200
300
400
500
600
Case # 110, Chromosome 01p

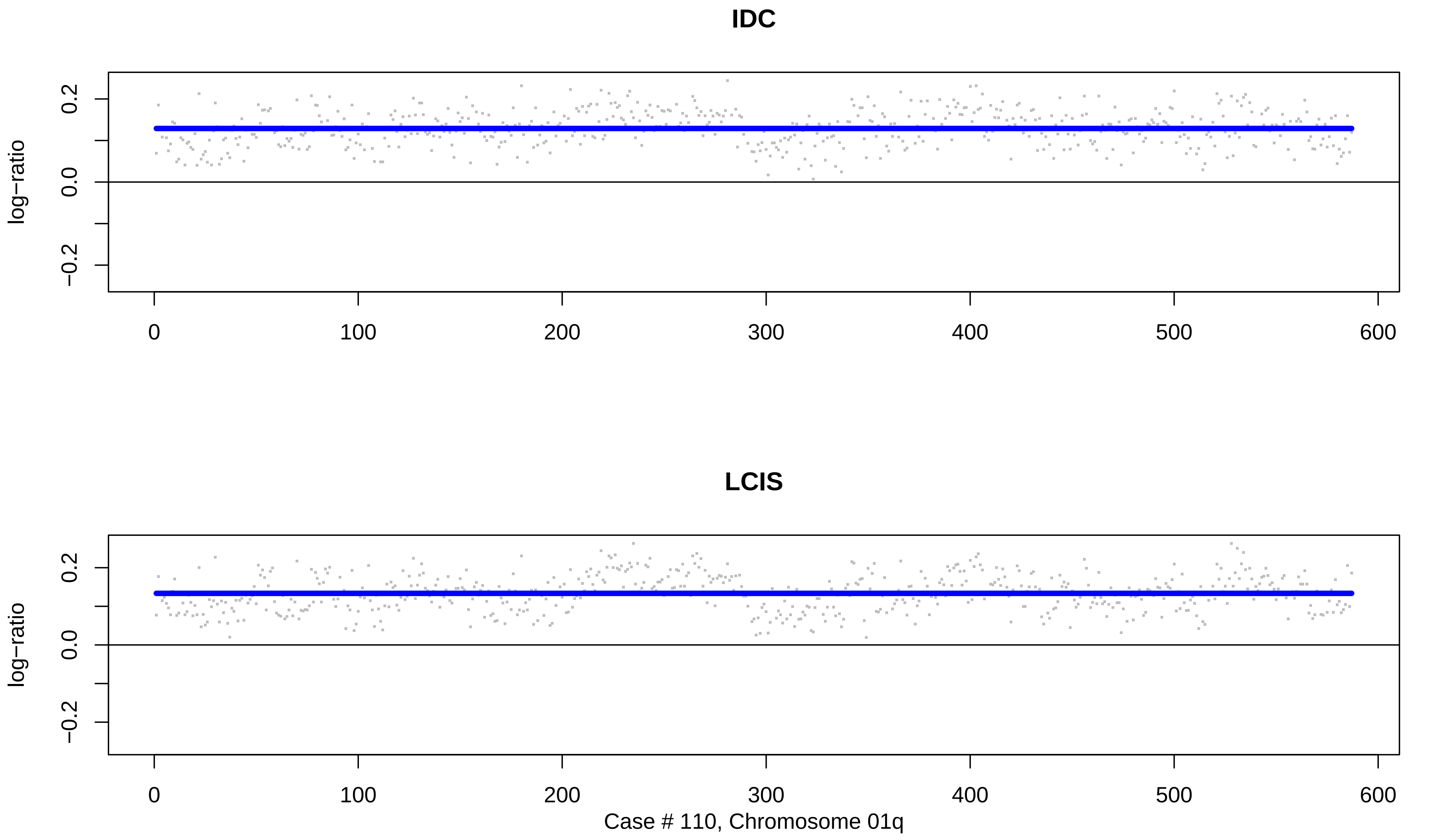
IDC
log−ratio
−0.2
0.0
0.2
0
100
200
300
400
500
600
LCIS
log−ratio
−0.2
0.0
0.2
0
100
200
300
400
500
600
Case # 110, Chromosome 01q

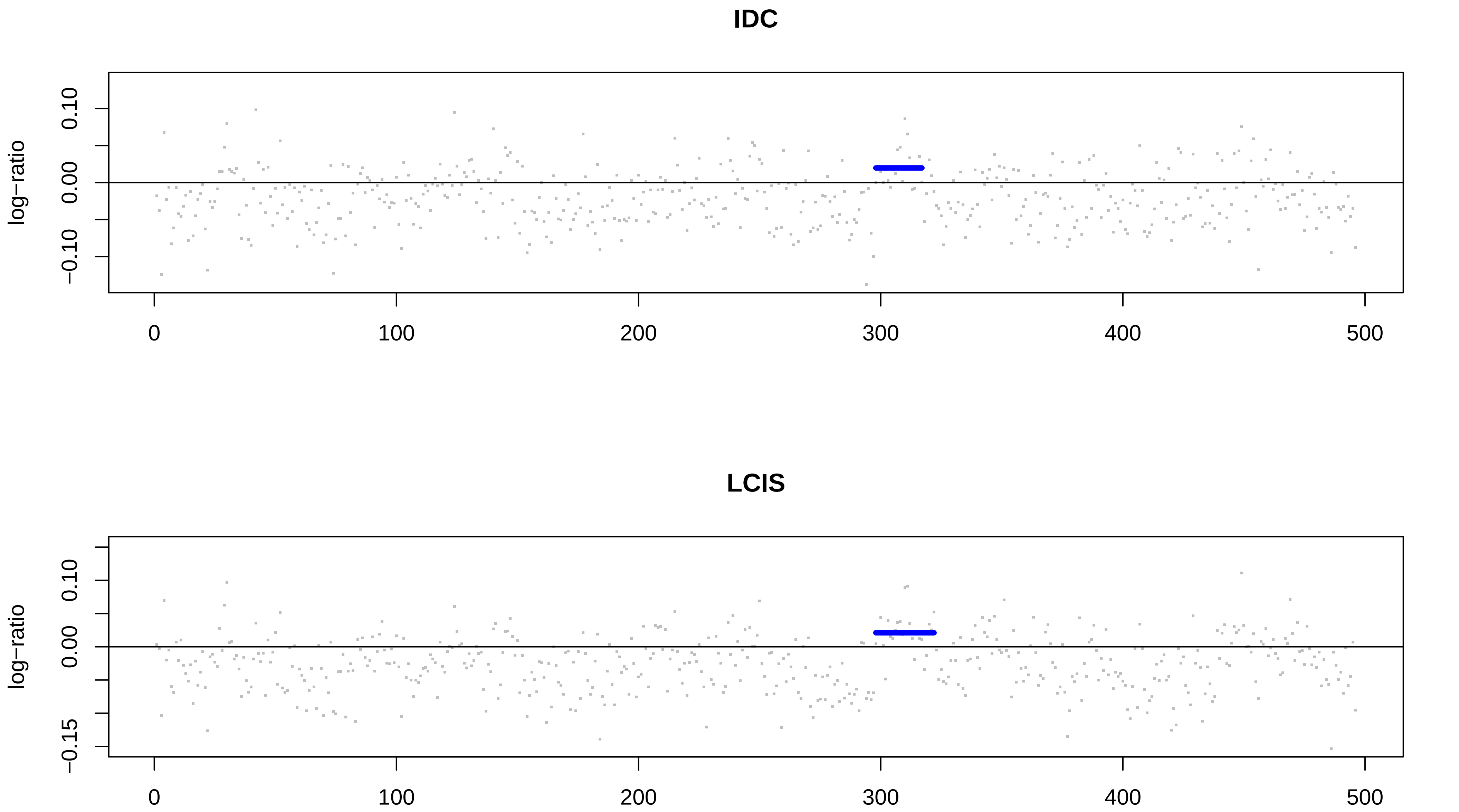

Case # 110, Chromosome 02p
Odds in favor of clonality = 1.2e+02

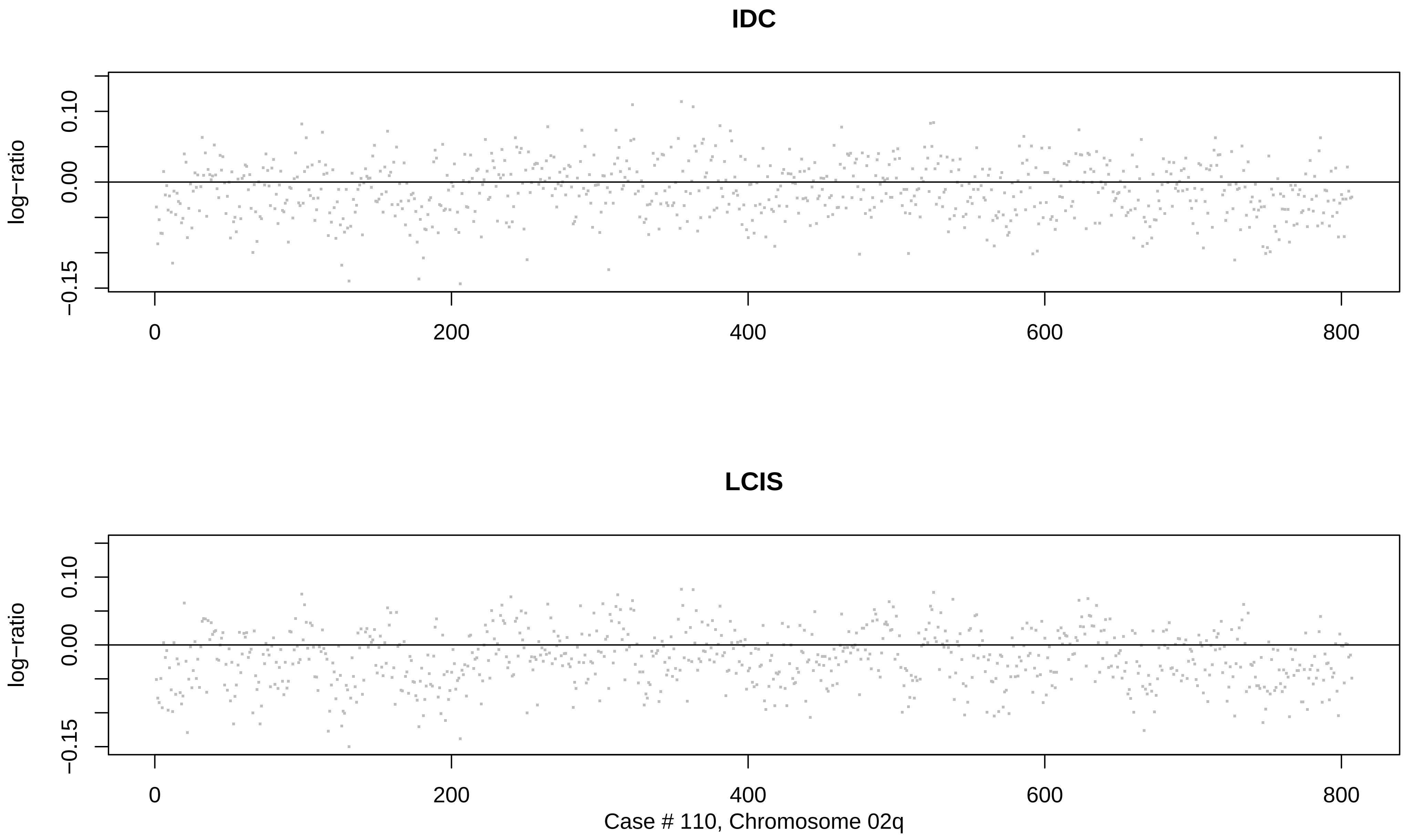

IDC
log−ratio
0.10
0.00
−0.15
0
200
400
600
800
LCIS
log−ratio
0.10
0.00
−0.15
0
200
400
600
800
Case # 110, Chromosome 02q

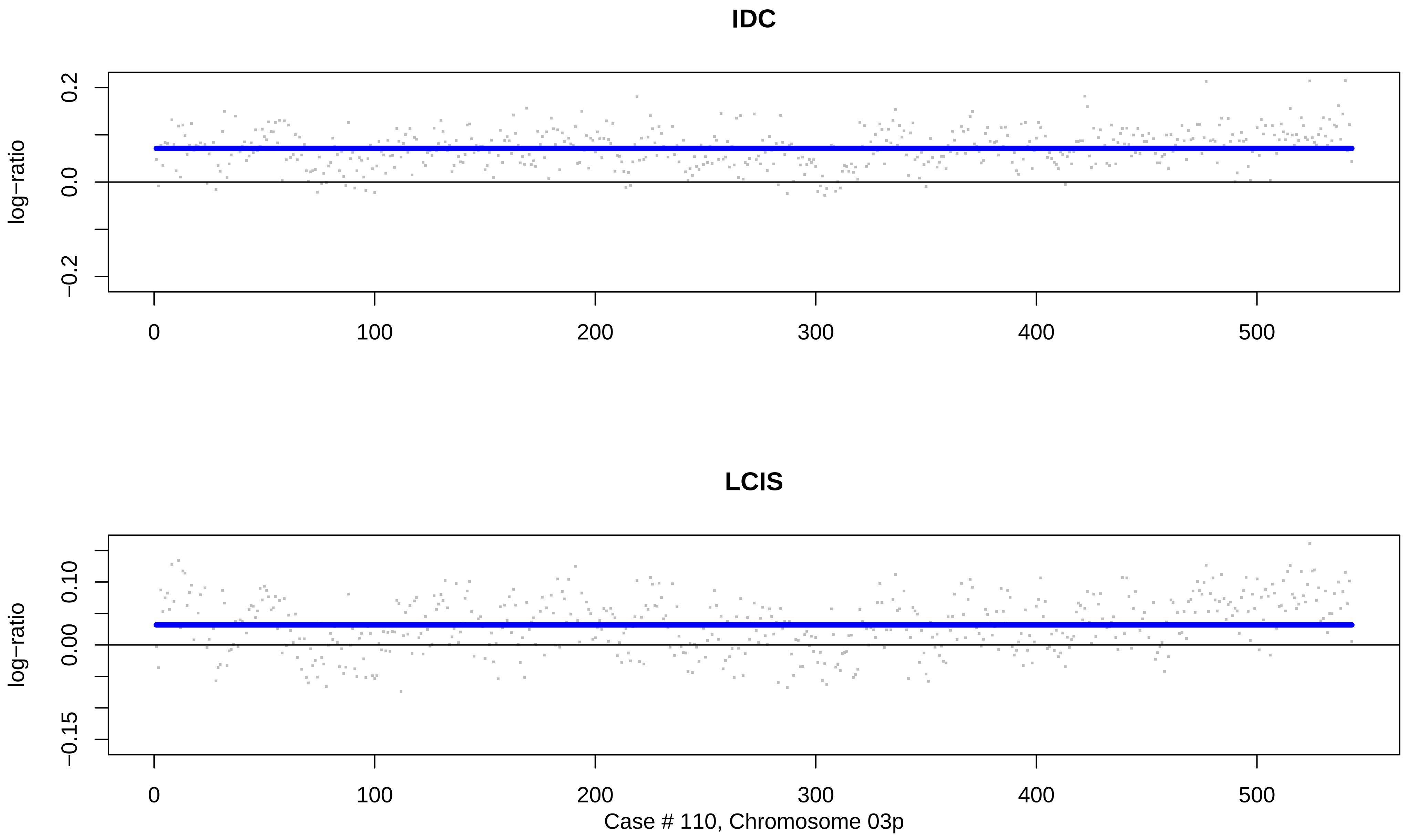
IDC
log−ratio
0.2
0.0
−0.2
0
100
200
300
400
500
LCIS
log−ratio
0.10
0.00
−0.15
0
100
200
300
400
500
Case # 110, Chromosome 03p

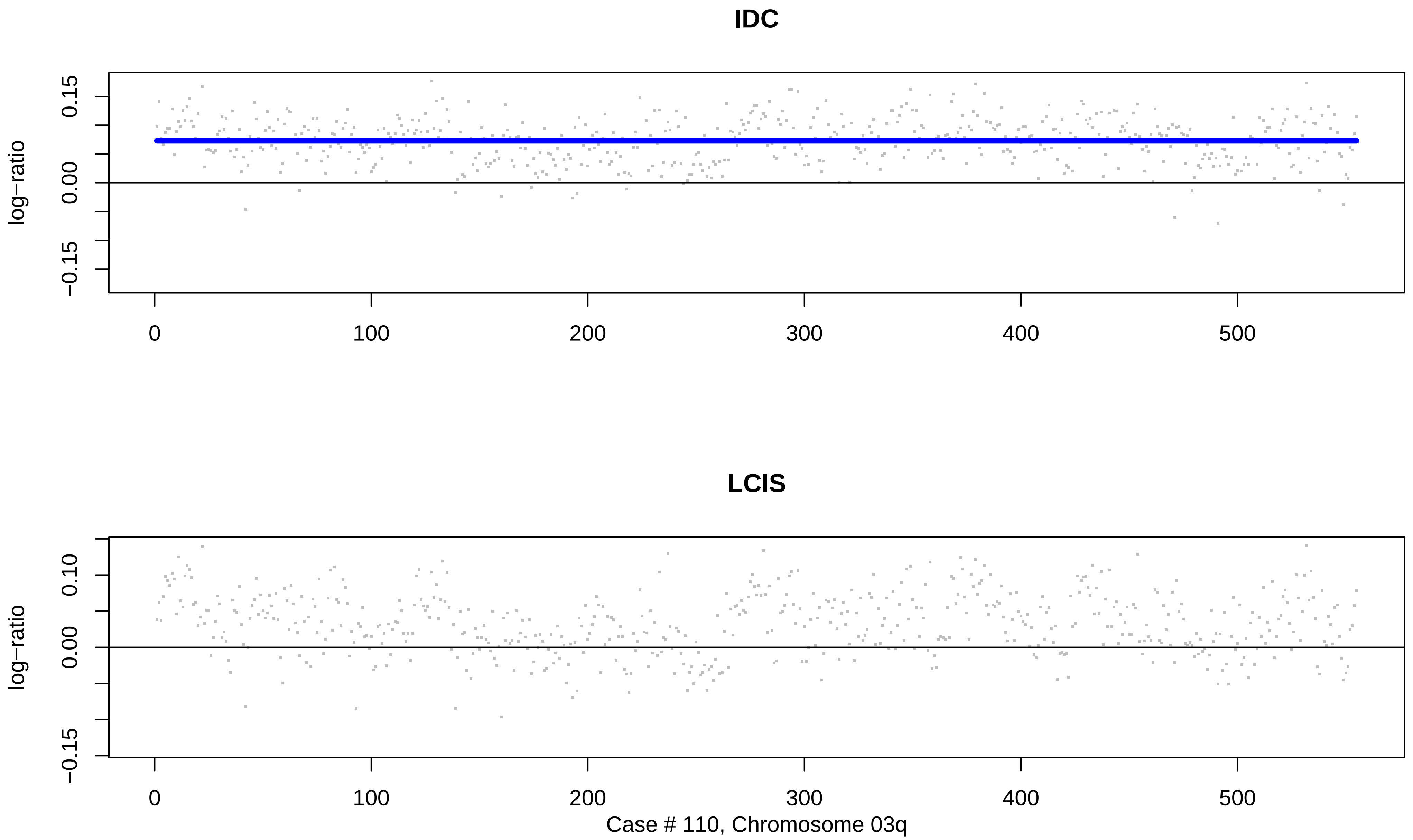
IDC
log−ratio
−0.15
0.00
0.15
0
100
200
300
400
500
LCIS
log−ratio
−0.15
0.00
0.10
0
100
200
300
400
500
Case # 110, Chromosome 03q

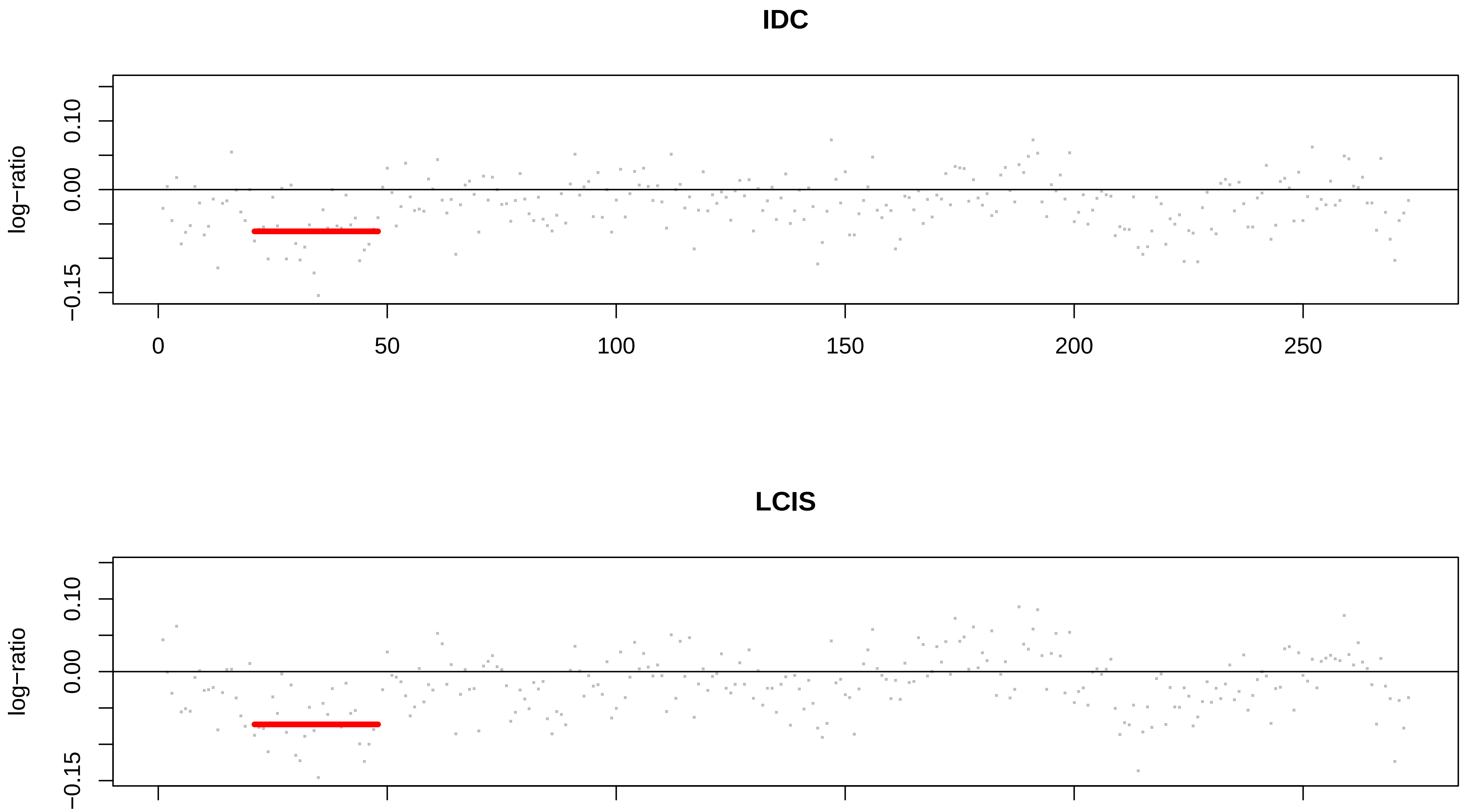

Case # 110, Chromosome 04p
Odds in favor of clonality = 17.4

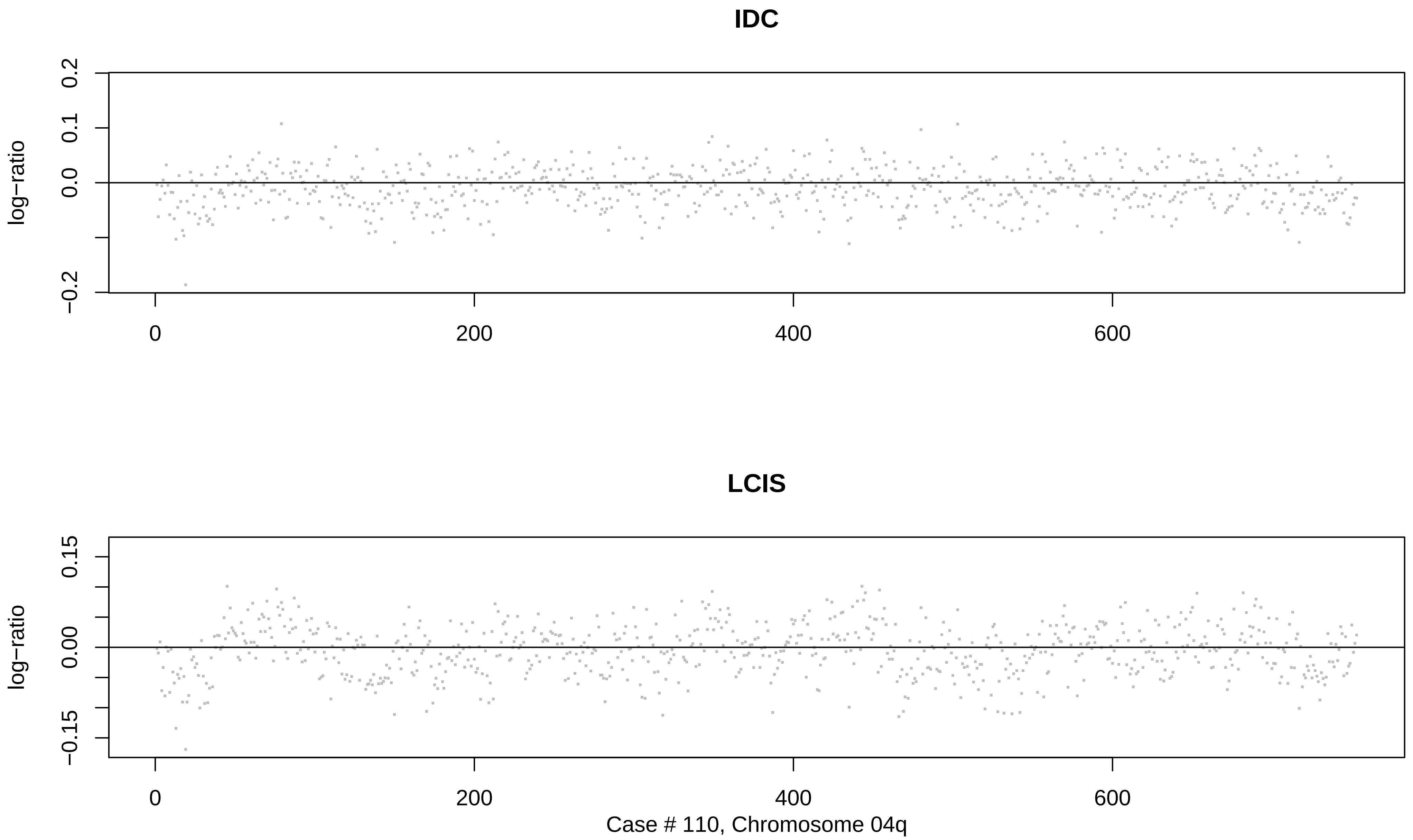
IDC
log−ratio
0.2
0.1
0.0
−0.2
0
200
400
600
LCIS
log−ratio
0.15
0.00
−0.15
0
200
400
600
Case # 110, Chromosome 04q

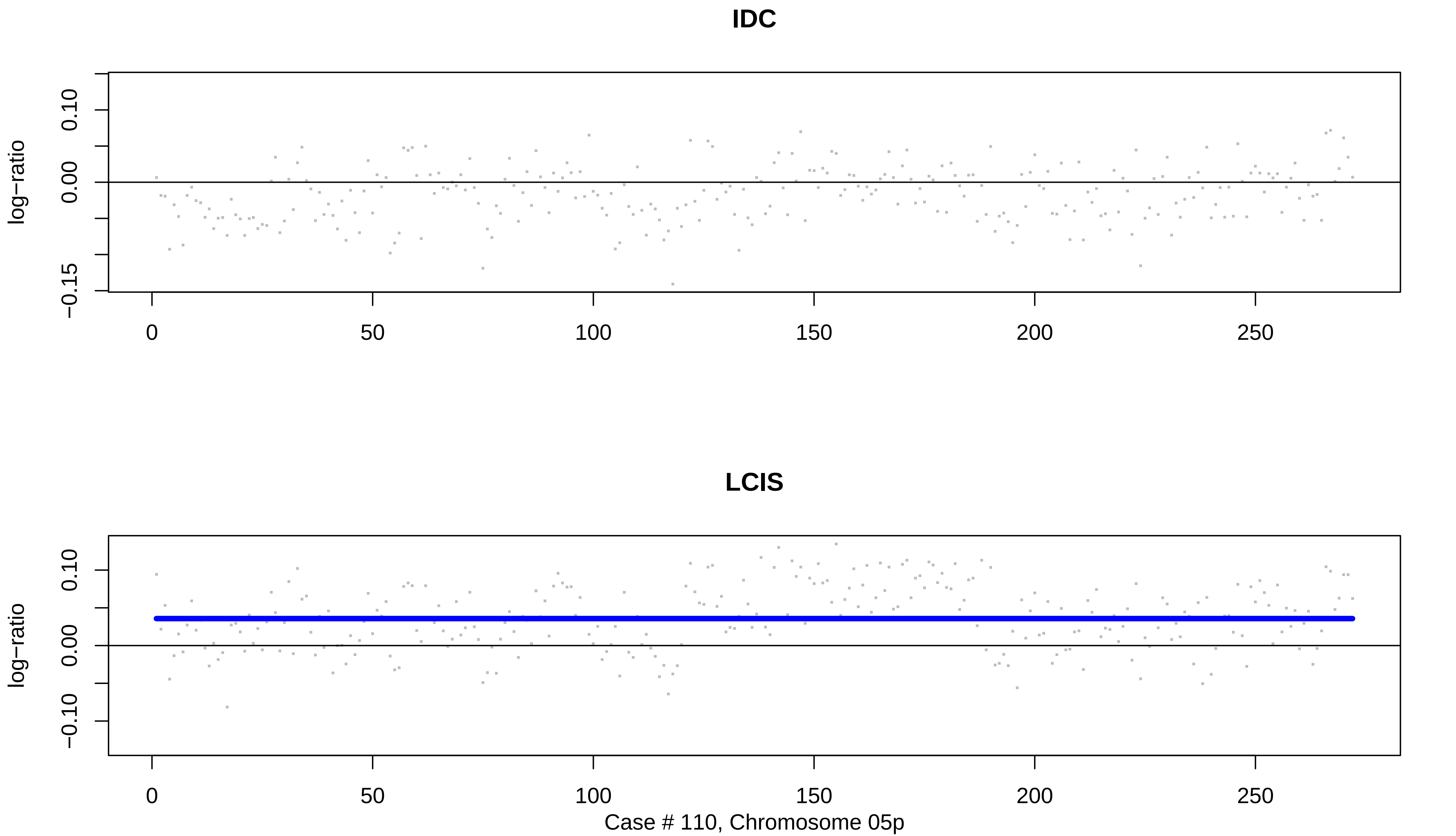
IDC
log−ratio
0.10
0.00
−0.15
0
50
100
150
200
250
LCIS
log−ratio
0.10
0.00
−0.10
0
50
100
150
200
250
Case # 110, Chromosome 05p

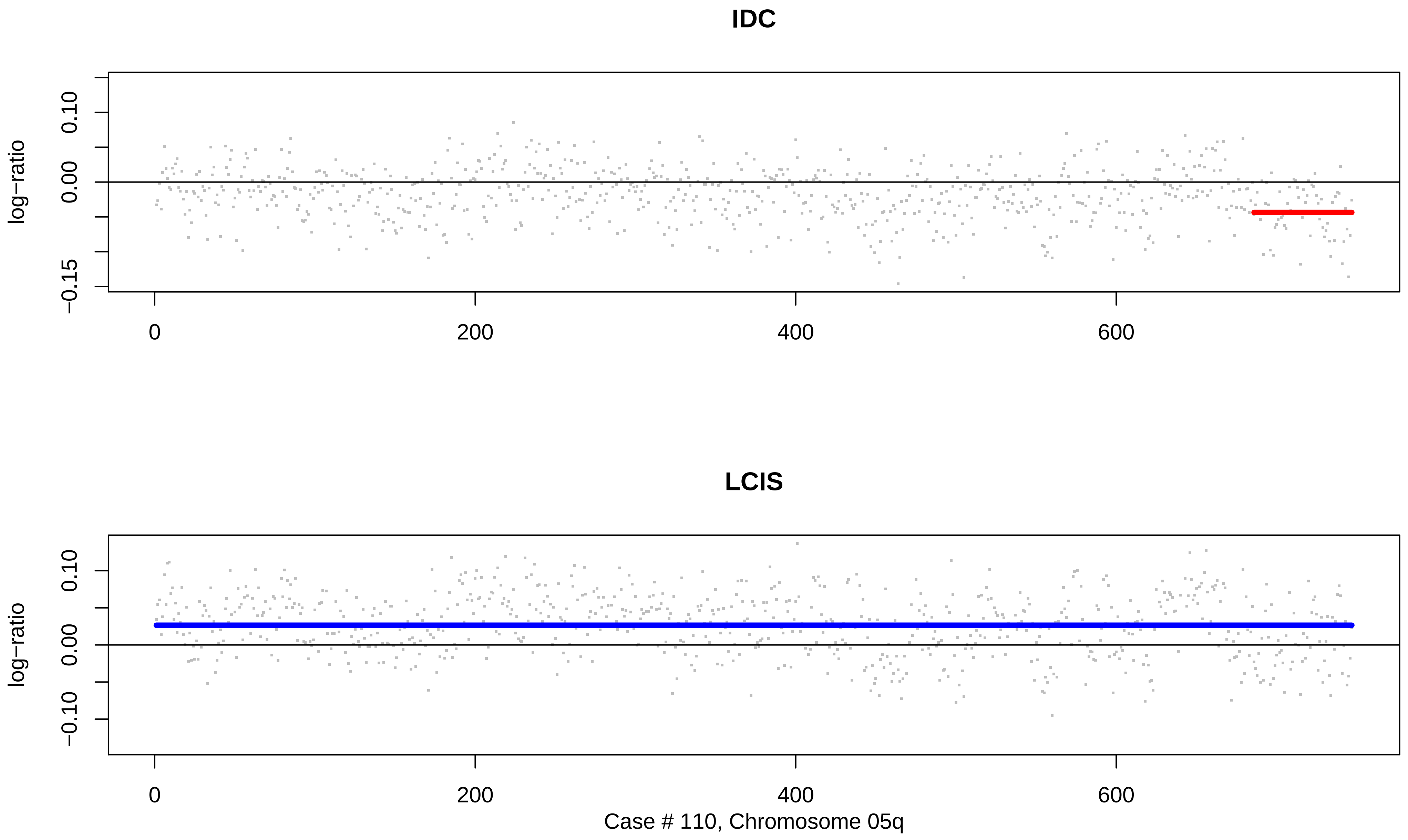
IDC
log−ratio
0.10
0.00
−0.15
0
200
400
600
LCIS
log−ratio
0.10
0.00
−0.10
0
200
400
600
Case # 110, Chromosome 05q

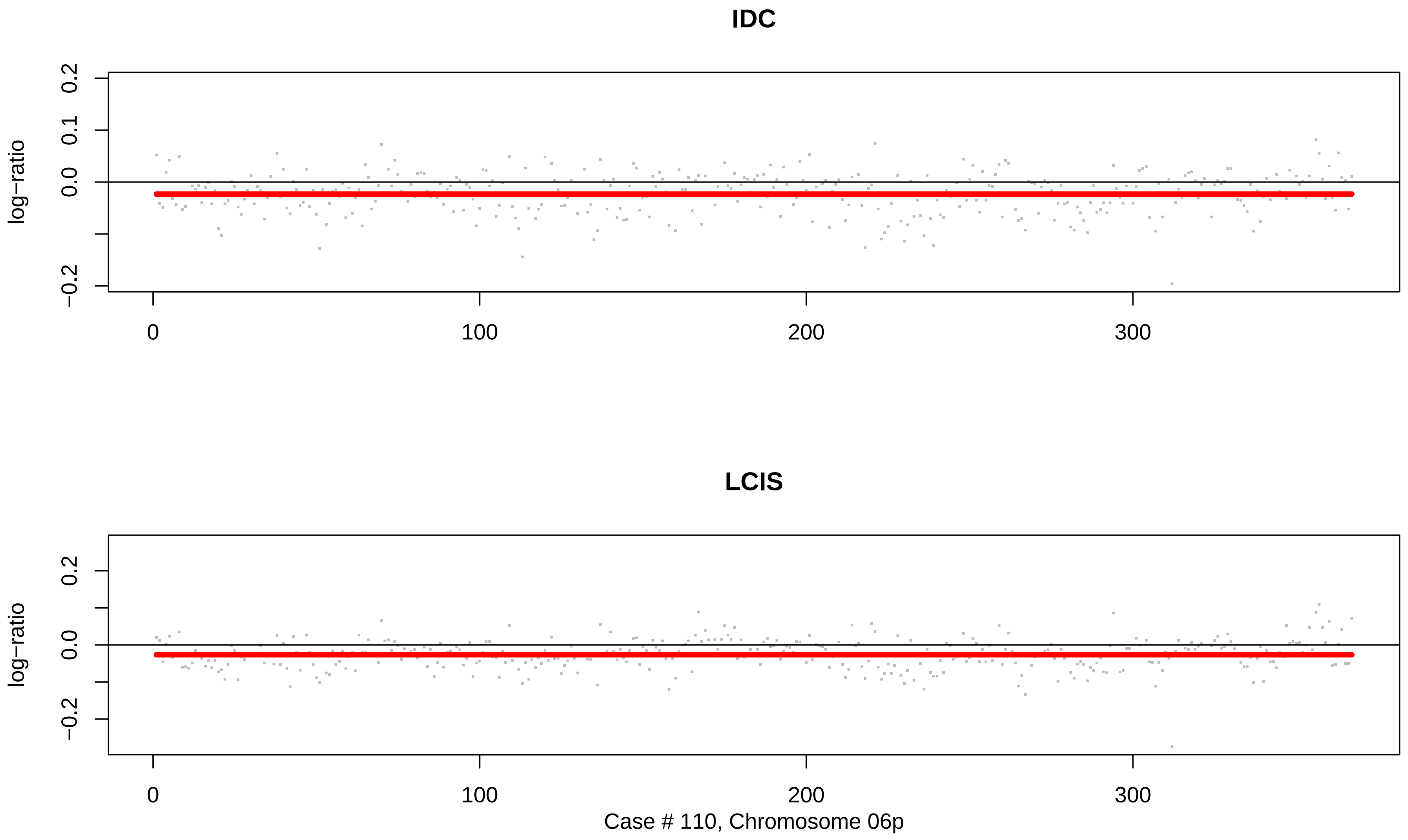

IDC
log−ratio
0.2
0.1
0.0
−0.2
0
100
200
300
LCIS
log−ratio
0.2
0.0
−0.2
0
100
200
300
Case # 110, Chromosome 06p

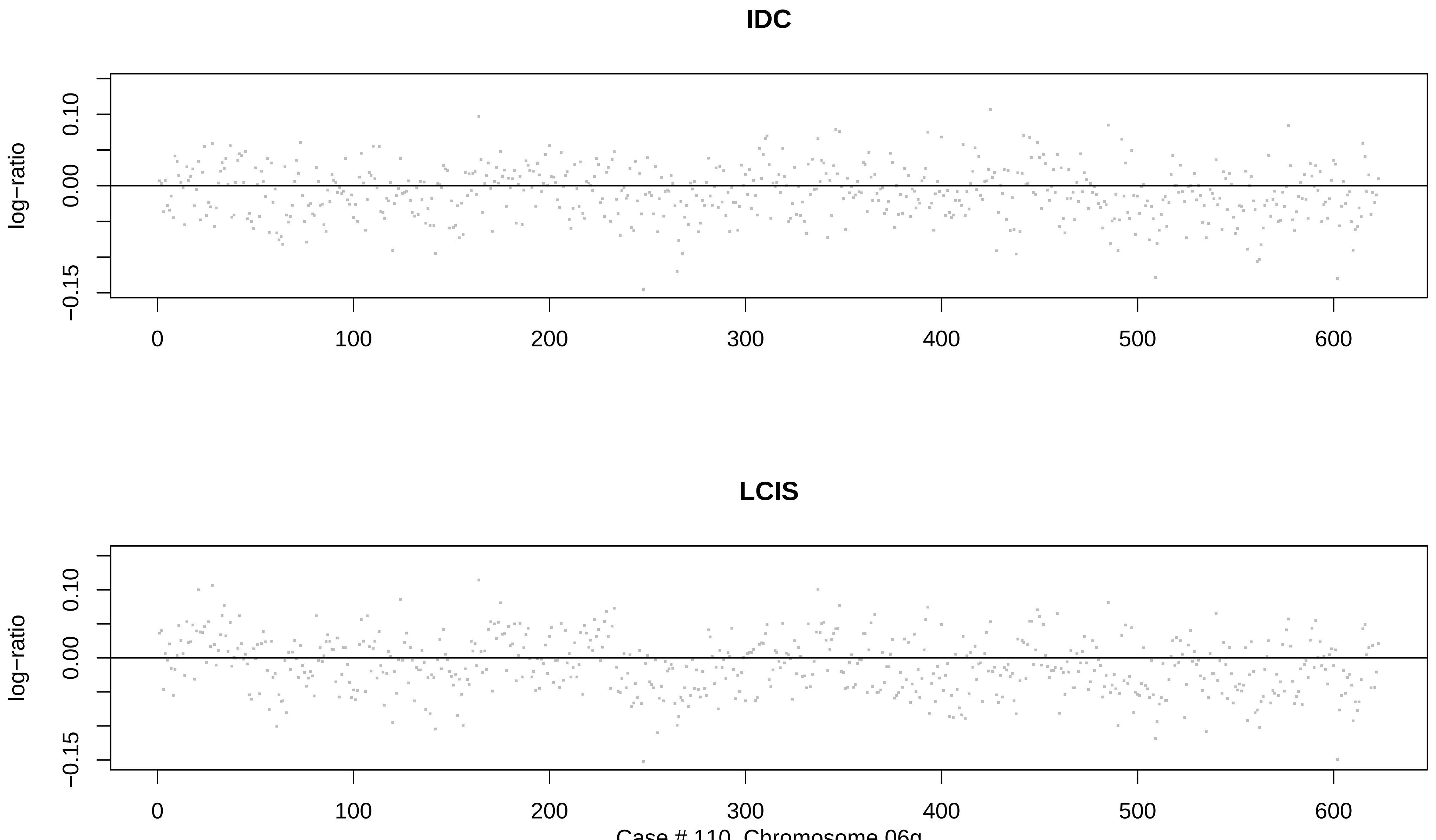
IDC
log−ratio
0.10
0.00
−0.15
0
100
200
300
400
500
600
LCIS
log−ratio
0.10
0.00
−0.15
0
100
200
300
400
500
600
Case # 110, Chromosome 06q

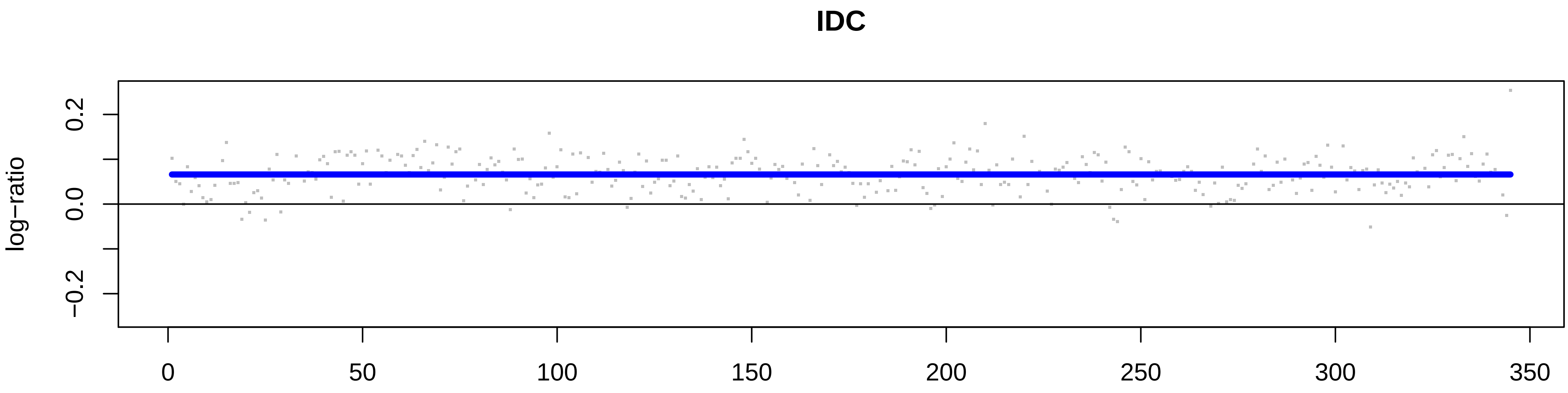
IDC
log−ratio
0.2
0.0
−0.2
0
50
100
150
200
250
300
350

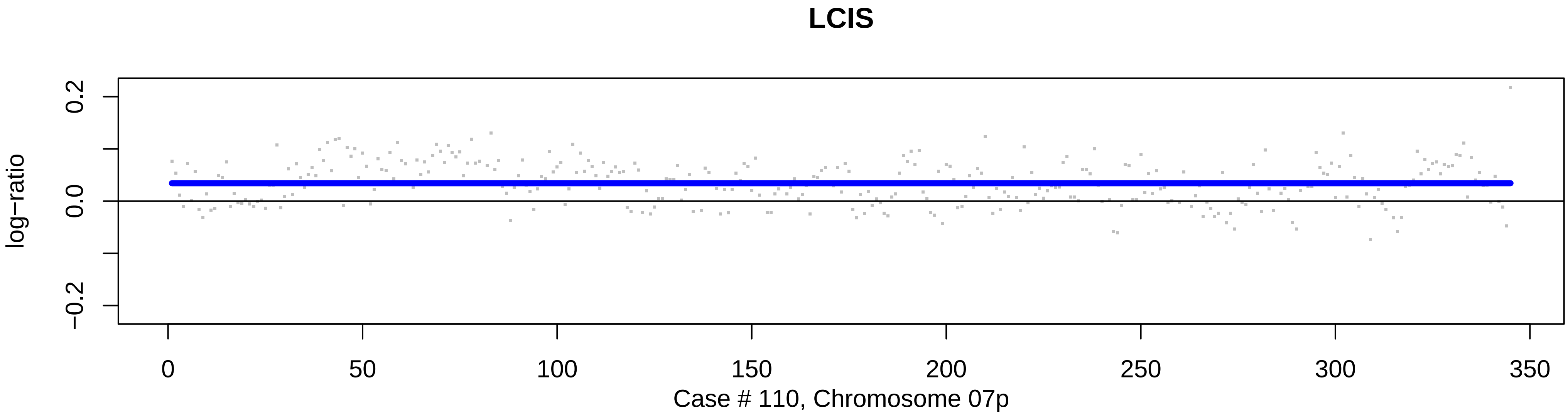
LCIS
log−ratio
0.2
0.0
−0.2
0
50
100
150
200
250
300
350
Case # 110, Chromosome 07p

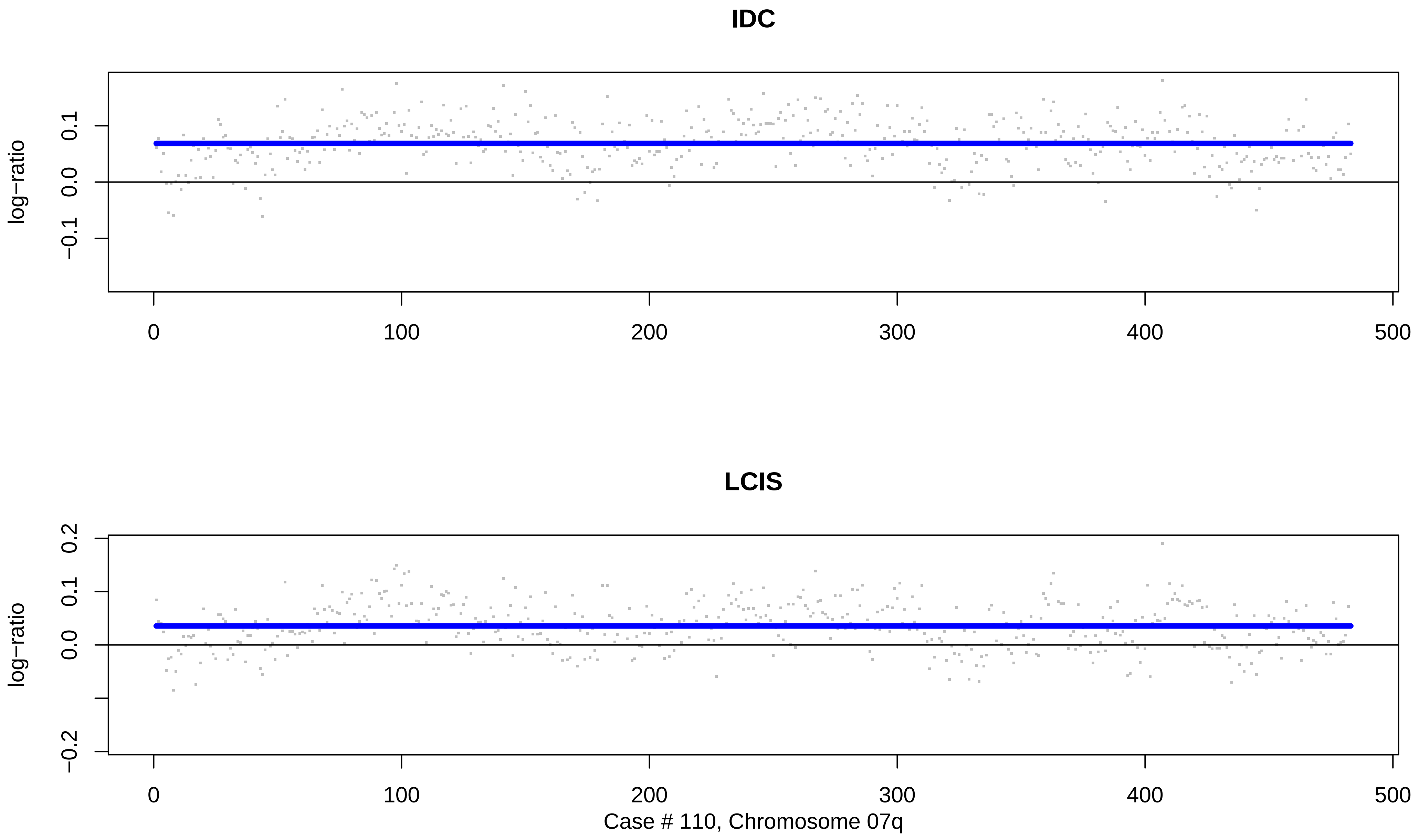
IDC
log−ratio
−0.1
0.0
0.1
0
100
200
300
400
500
LCIS
log−ratio
−0.2
0.0
0.1
0.2
0
100
200
300
400
500
Case # 110, Chromosome 07q

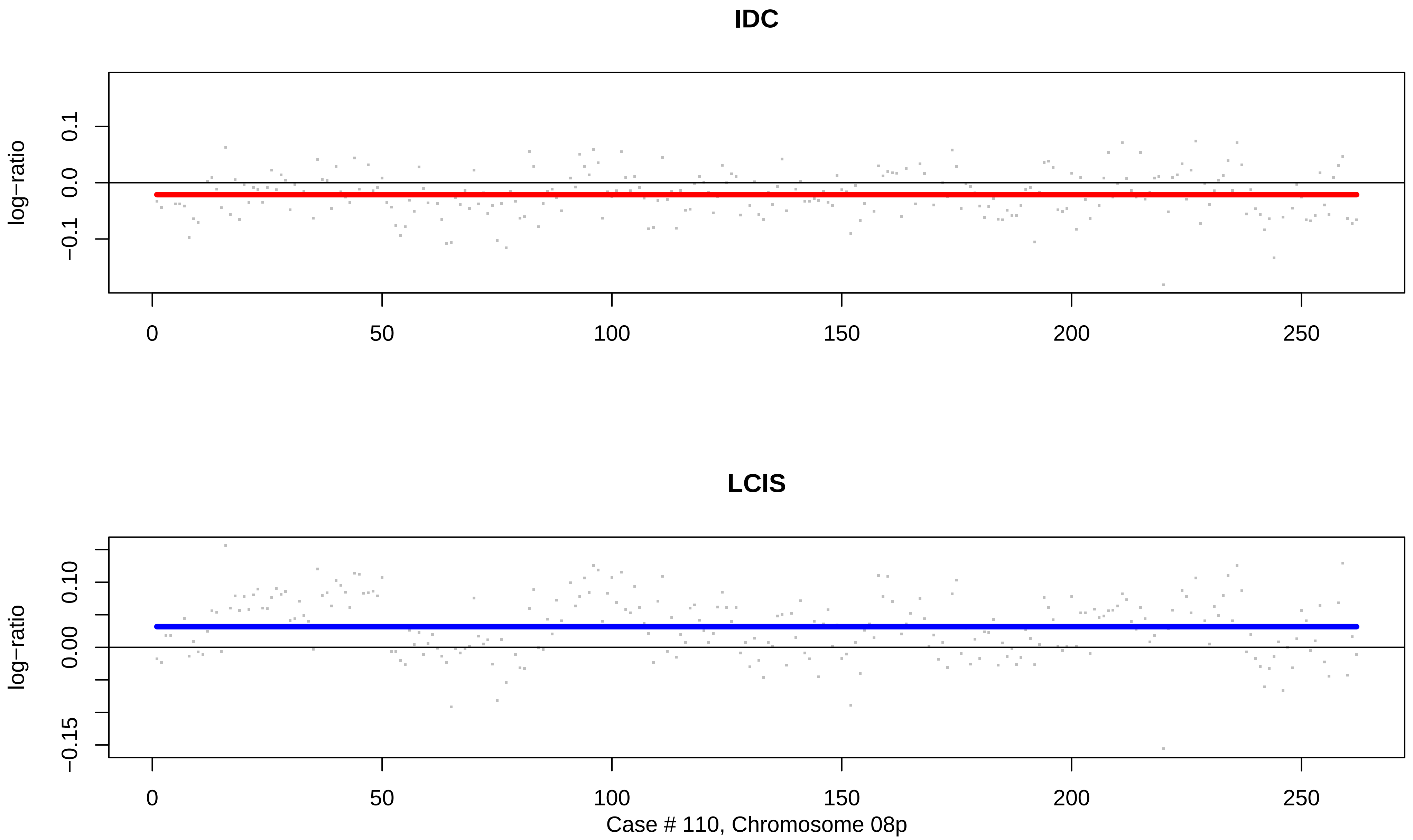
IDC
log−ratio
0.1
0.0
−0.1
0
50
100
150
200
250
LCIS
log−ratio
0.10
0.00
−0.15
0
50
100
150
200
250
Case # 110, Chromosome 08p

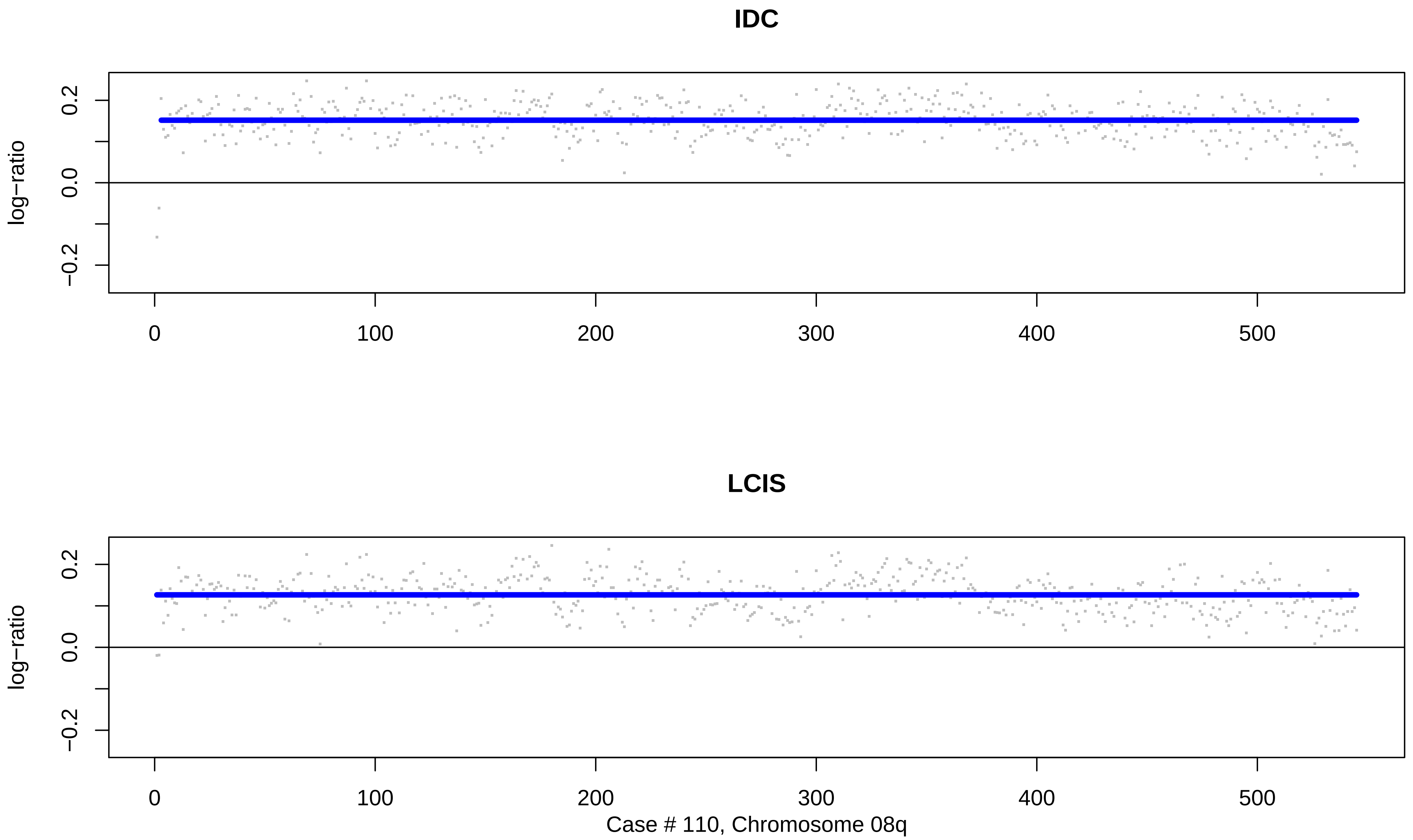
IDC
log−ratio
0.2
0.0
−0.2
0
100
200
300
400
500
LCIS
log−ratio
0.2
0.0
−0.2
0
100
200
300
400
500
Case # 110, Chromosome 08q

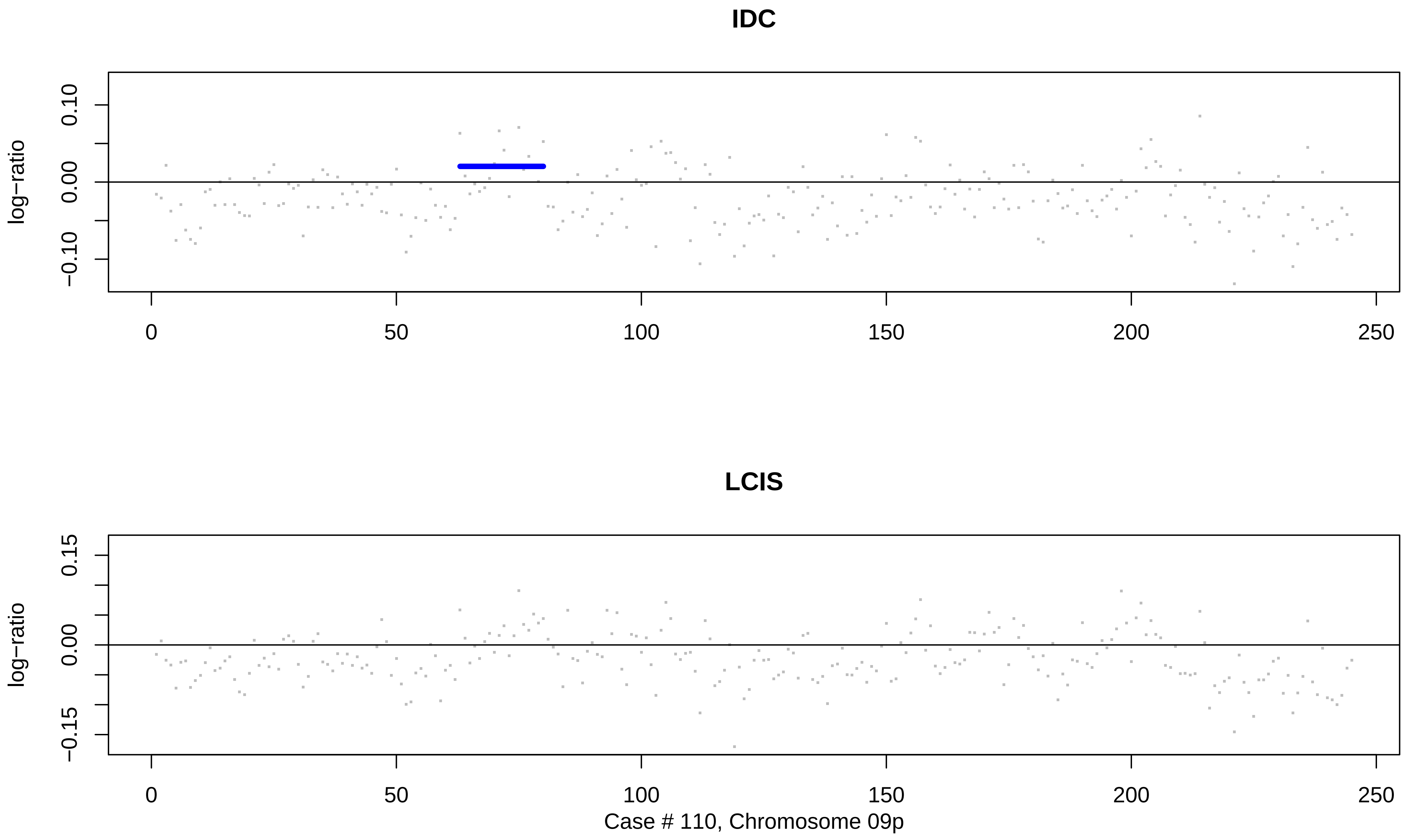

IDC
log−ratio
−0.10
0.00
0.10
0
50
100
150
200
250
LCIS
log−ratio
−0.15
0.00
0.15
0
50
100
150
200
250
Case # 110, Chromosome 09p

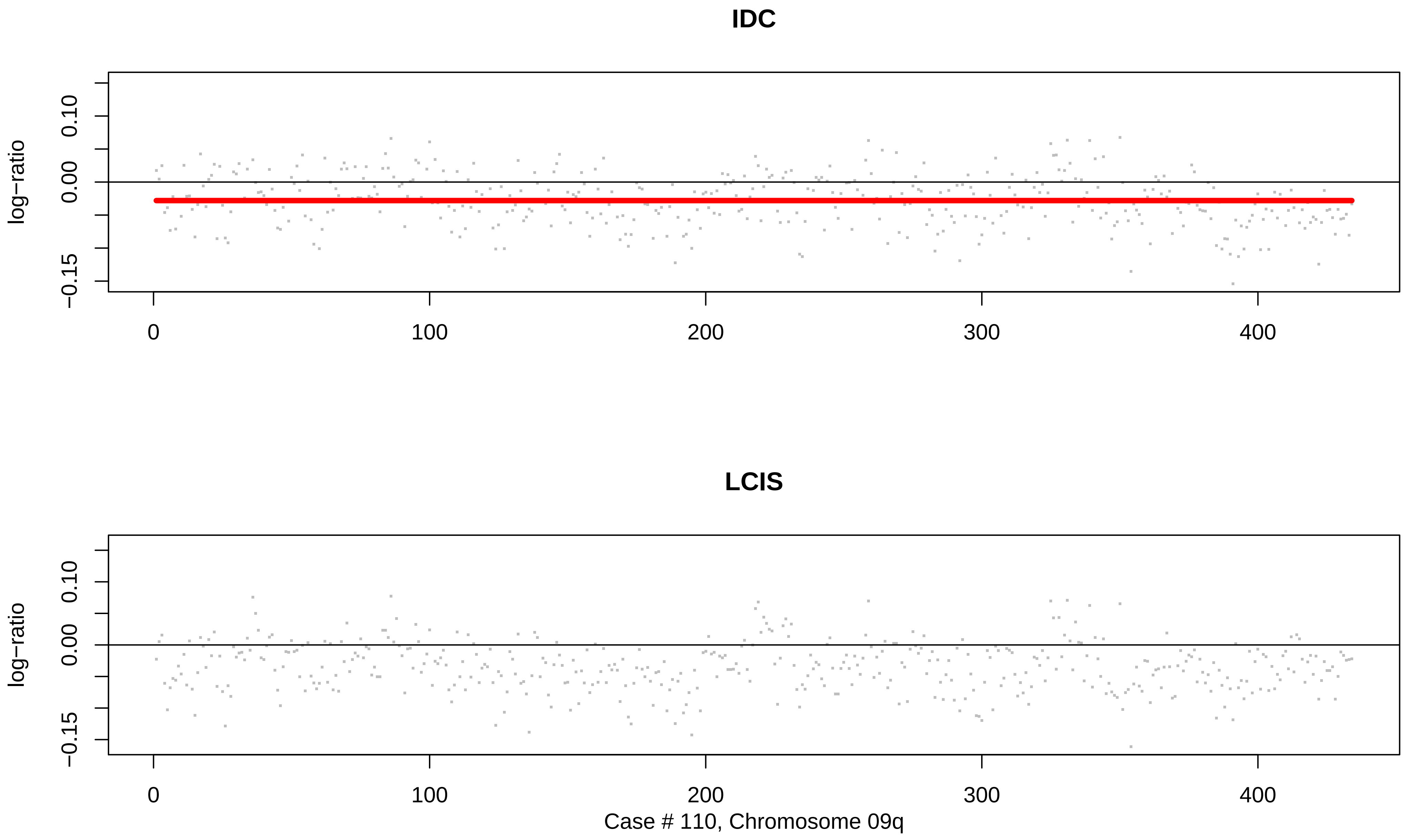
IDC
log−ratio
−0.15
0.00
0.10
0
100
200
300
400
LCIS
log−ratio
−0.15
0.00
0.10
0
100
200
300
400
Case # 110, Chromosome 09q

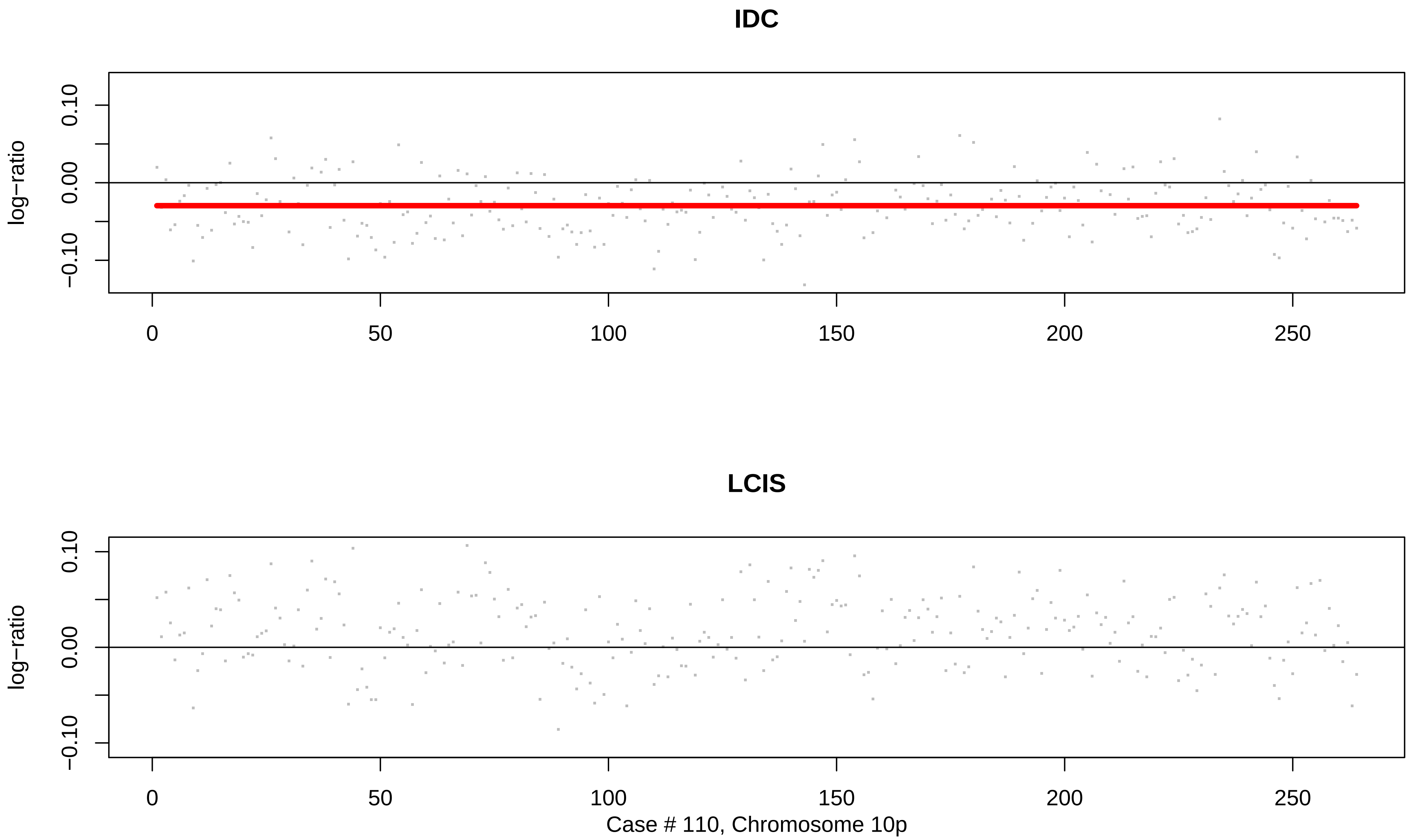

IDC
log−ratio
0.10
0.00
−0.10
0
50
100
150
200
250
LCIS
log−ratio
0.10
0.00
−0.10
0
50
100
150
200
250
Case # 110, Chromosome 10p

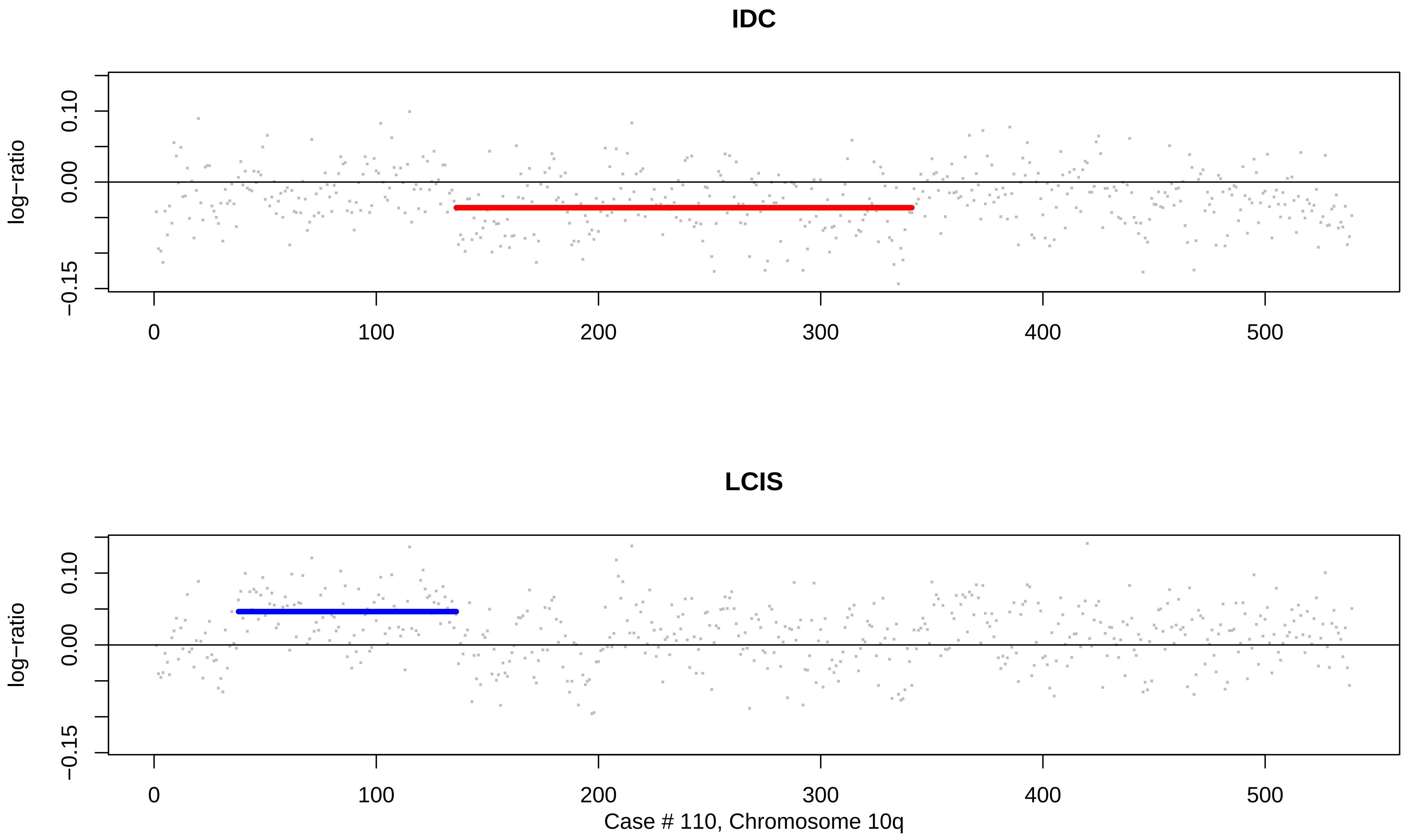
IDC
log−ratio
0.10
0.00
−0.15
0
100
200
300
400
500
LCIS
log−ratio
0.10
0.00
−0.15
0
100
200
300
400
500
Case # 110, Chromosome 10q

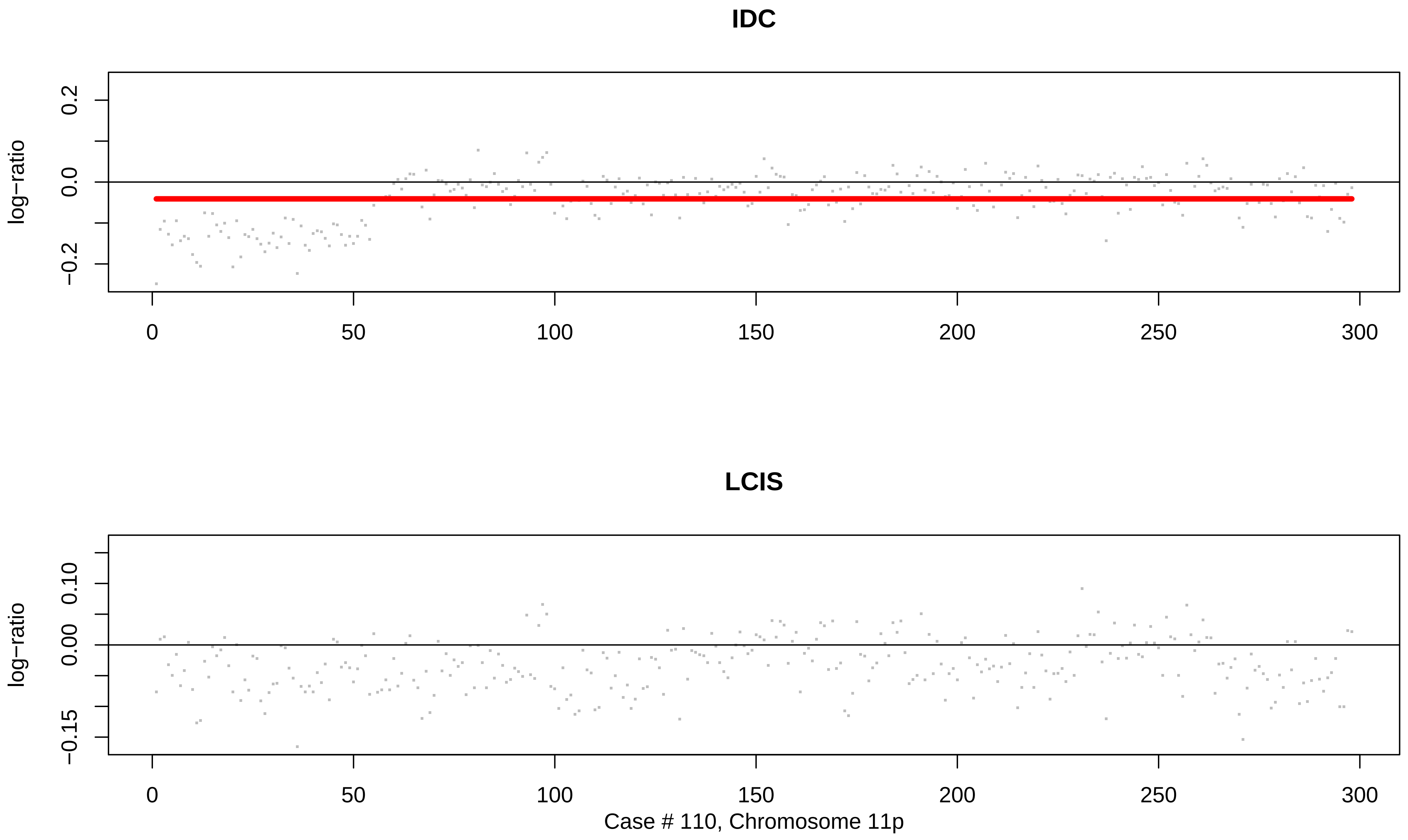

IDC
log−ratio
0.2
0.0
−0.2
0
50
100
150
200
250
300
LCIS
log−ratio
0.10
0.00
−0.15
0
50
100
150
200
250
300
Case # 110, Chromosome 11p

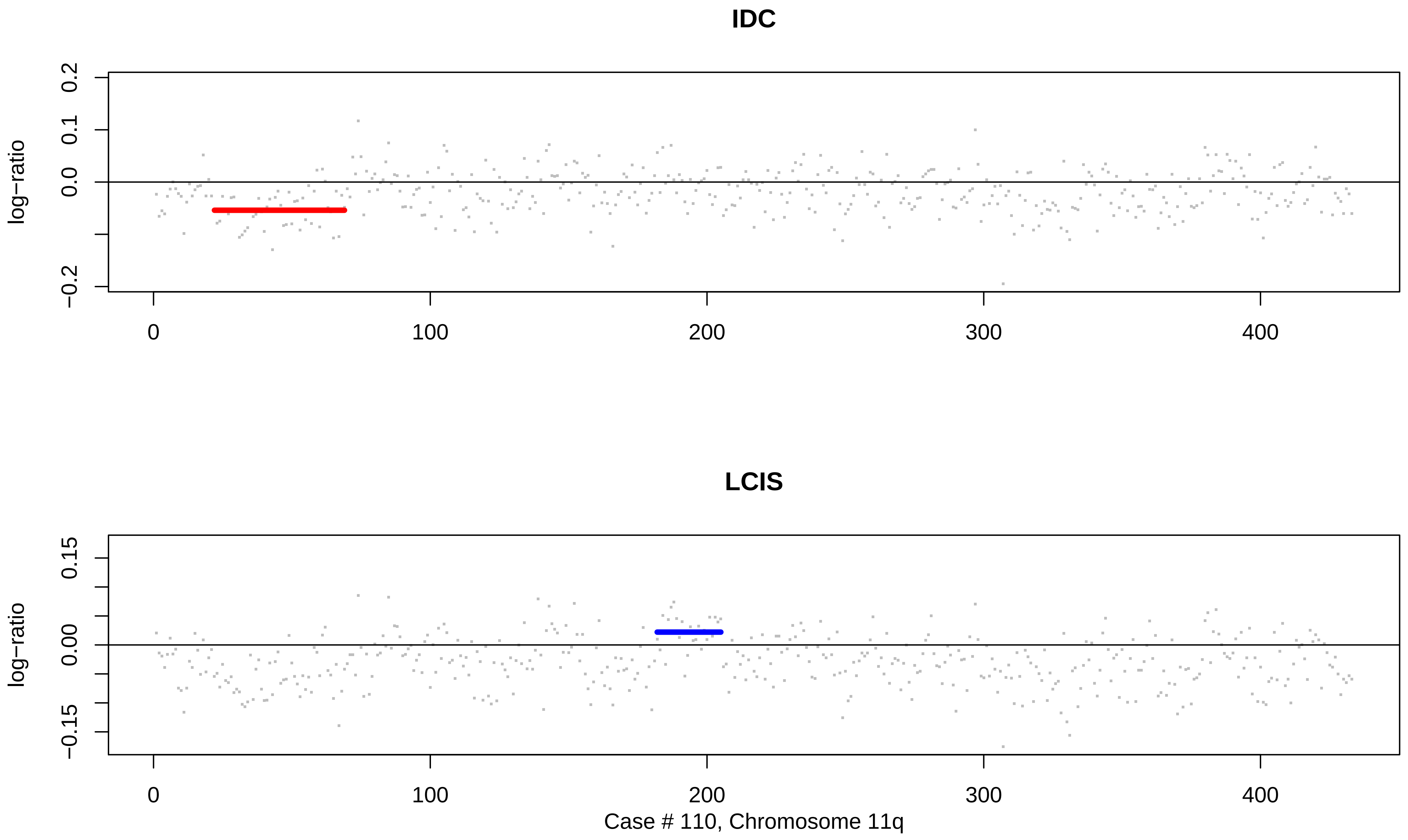
IDC
log−ratio
0.2
0.1
0.0
−0.2
0
100
200
300
400
LCIS
log−ratio
0.15
0.00
−0.15
0
100
200
300
400
Case # 110, Chromosome 11q

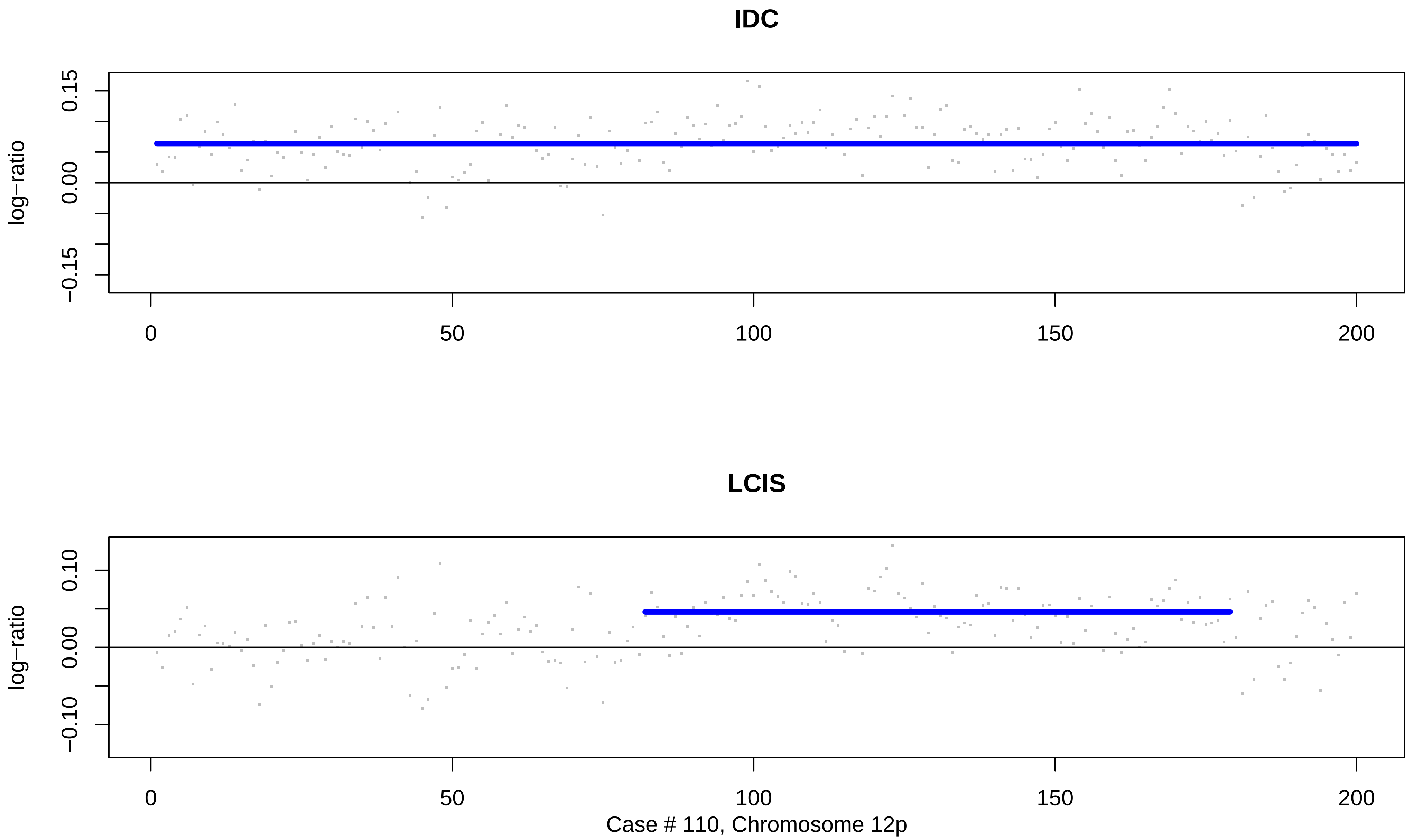

IDC
log−ratio
0.15
0.00
−0.15
0
50
100
150
200
LCIS
log−ratio
0.10
0.00
−0.10
0
50
100
150
200
Case # 110, Chromosome 12p

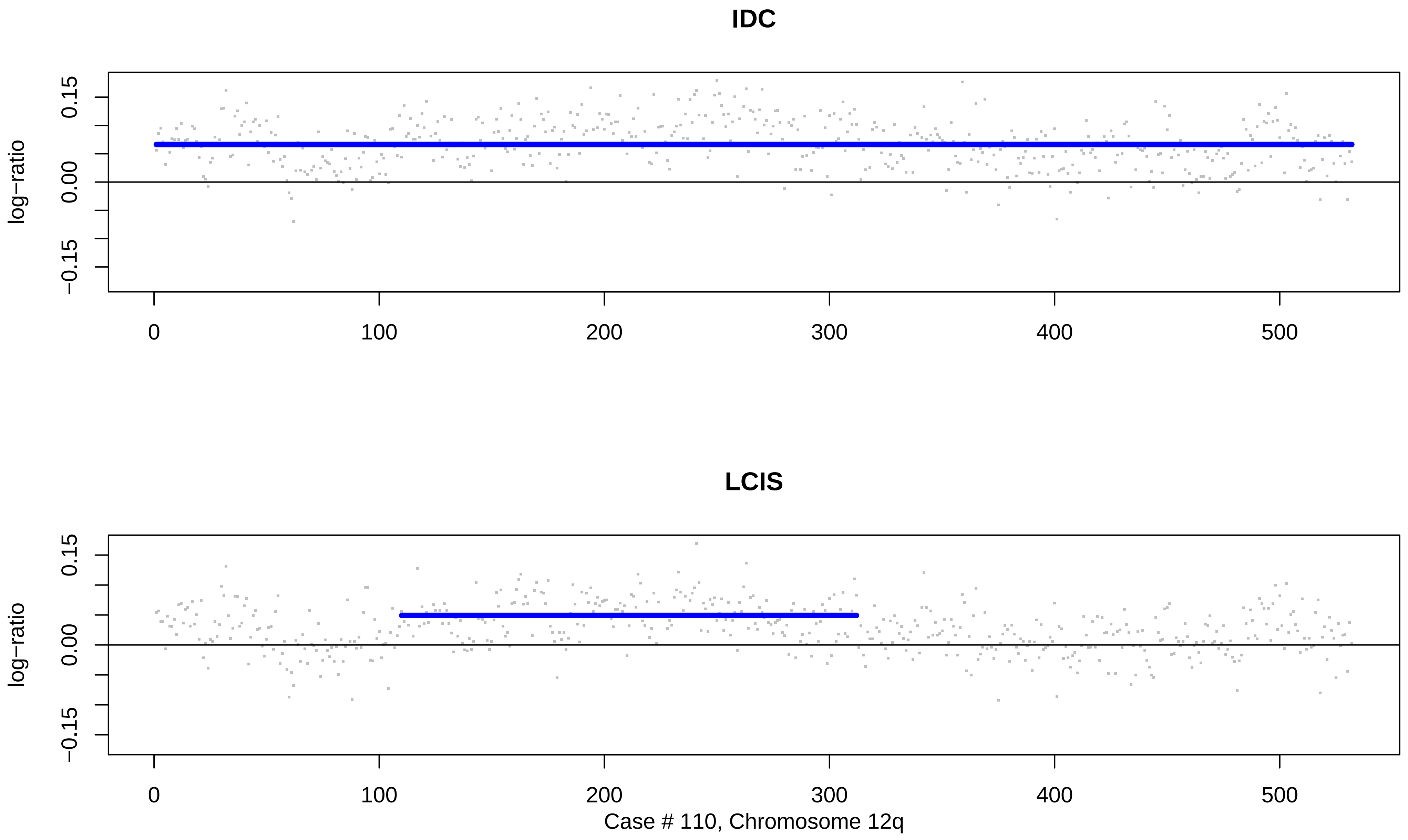
IDC
log−ratio
0.15
0.00
−0.15
0
100
200
300
400
500
LCIS
log−ratio
0.15
0.00
−0.15
0
100
200
300
400
500
Case # 110, Chromosome 12q

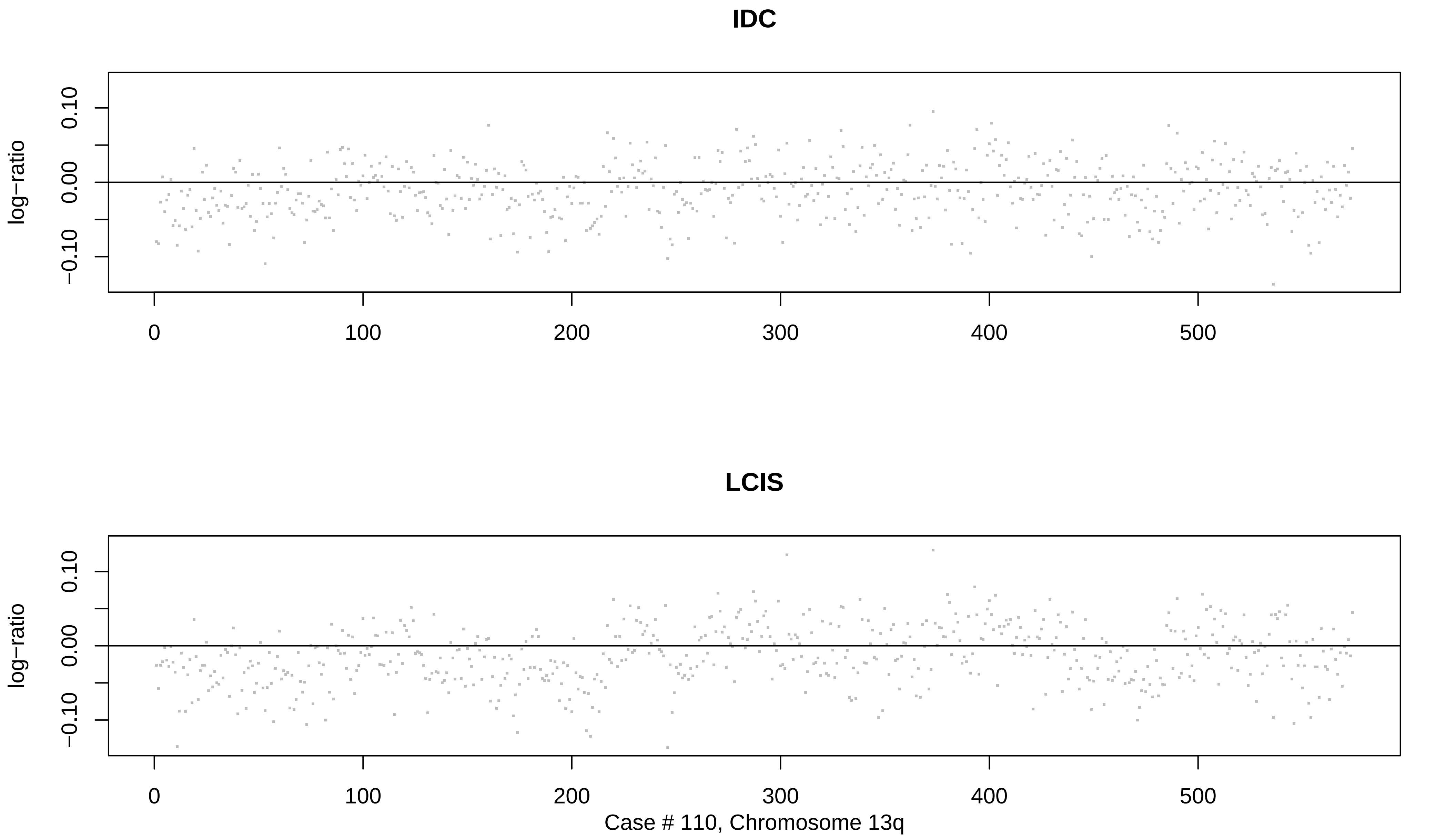

IDC
log−ratio
0.10
0.00
−0.10
0
100
200
300
400
500
LCIS
log−ratio
0.10
0.00
−0.10
0
100
200
300
400
500
Case # 110, Chromosome 13q

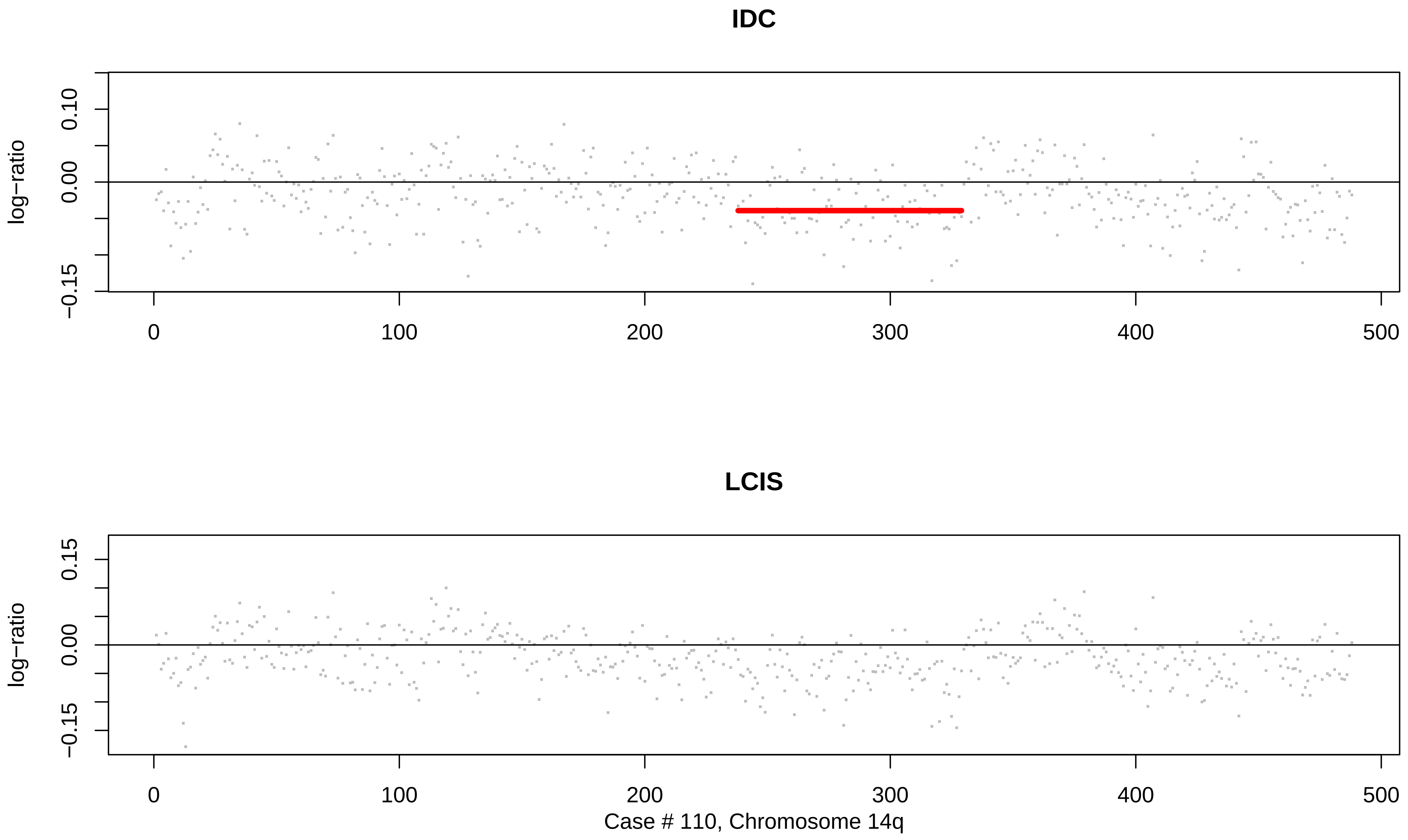
IDC
log−ratio
−0.15
0.00
0.10
0
100
200
300
400
500
LCIS
log−ratio
−0.15
0.00
0.15
0
100
200
300
400
500
Case # 110, Chromosome 14q

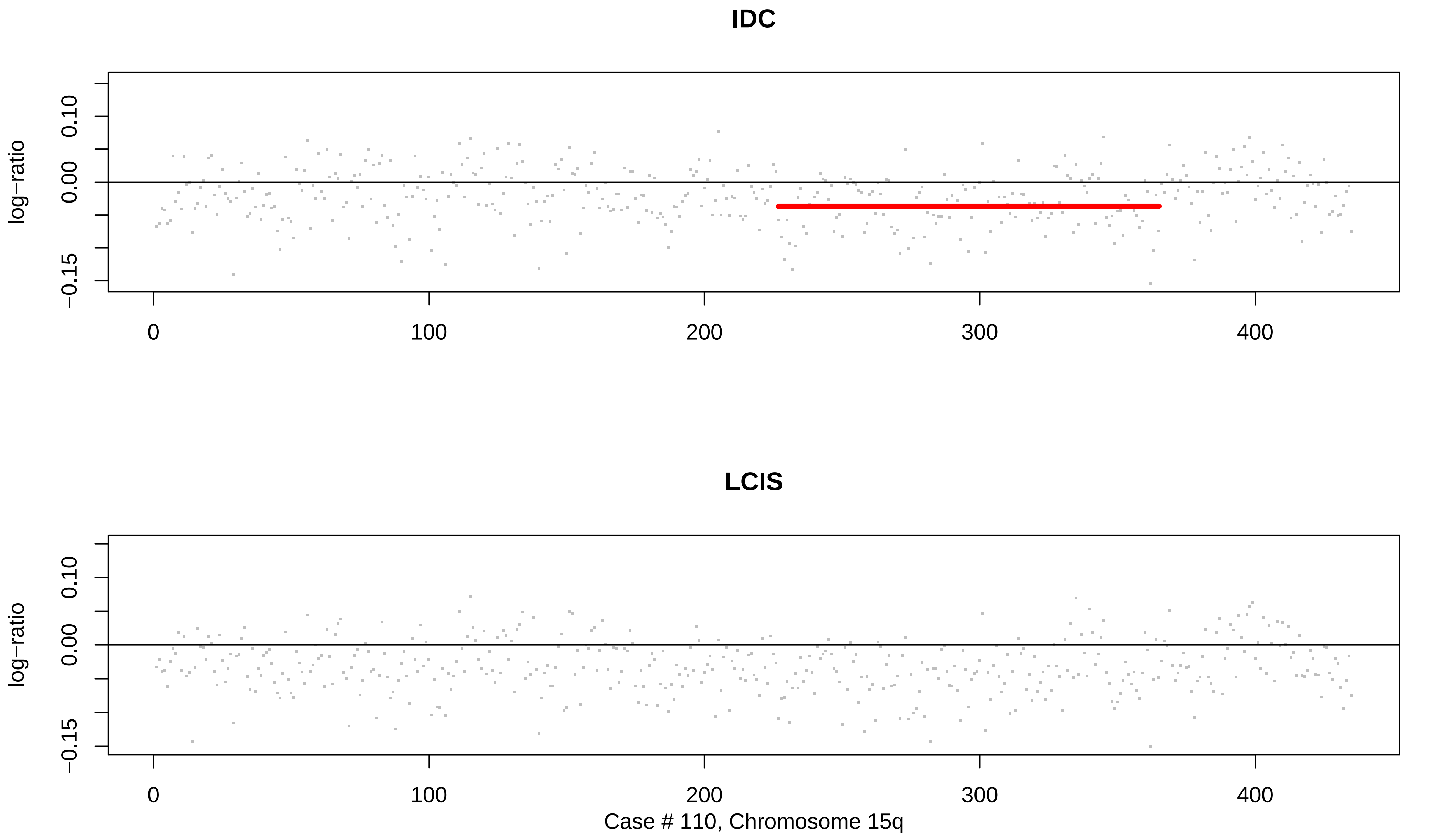
IDC
log−ratio
0.10
0.00
−0.15
0
100
200
300
400
LCIS
log−ratio
0.10
0.00
−0.15
0
100
200
300
400
Case # 110, Chromosome 15q

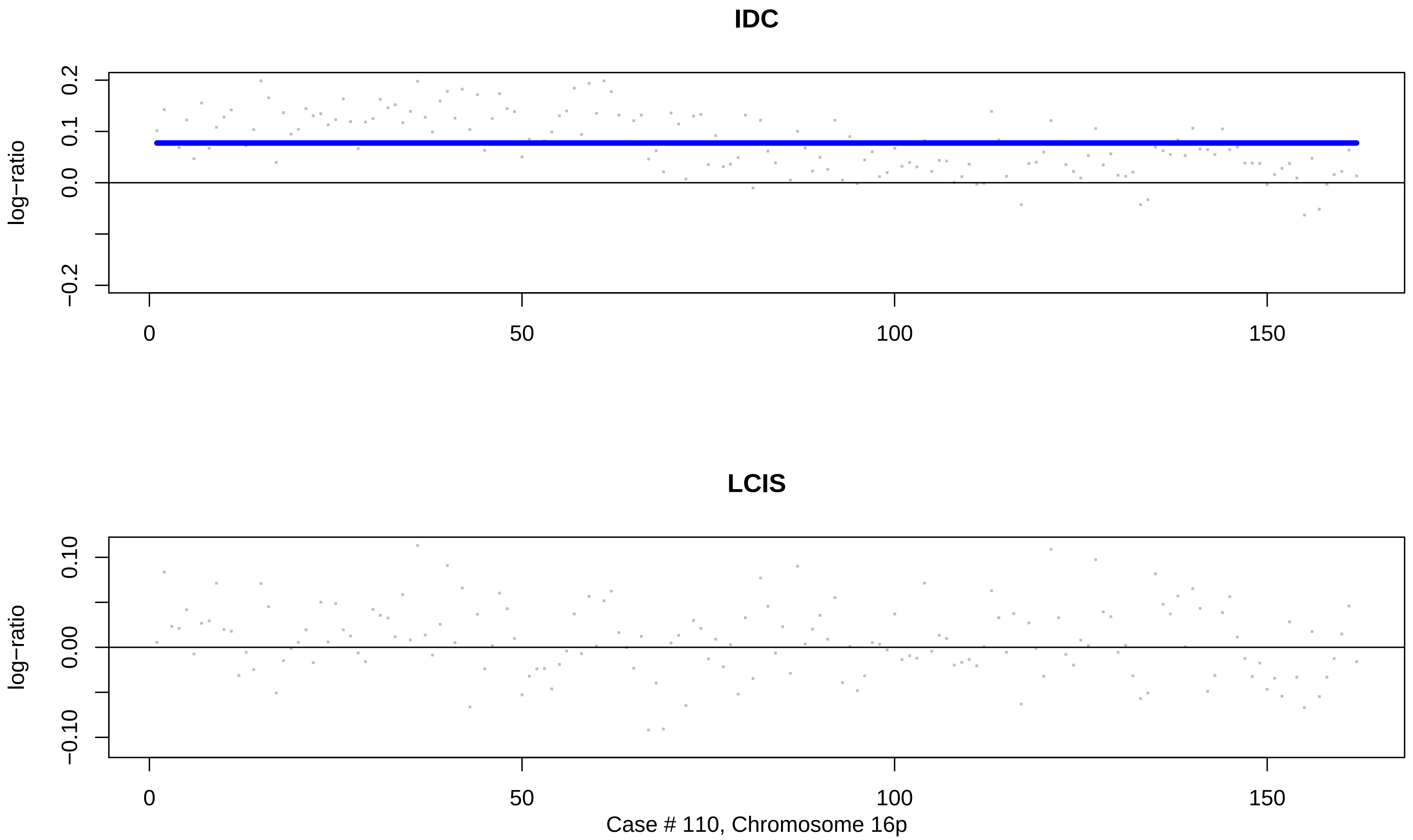
IDC
log−ratio
0.2
0.1
0.0
−0.2
0
50
100
150
LCIS
log−ratio
0.10
0.00
−0.10
0
50
100
150
Case # 110, Chromosome 16p

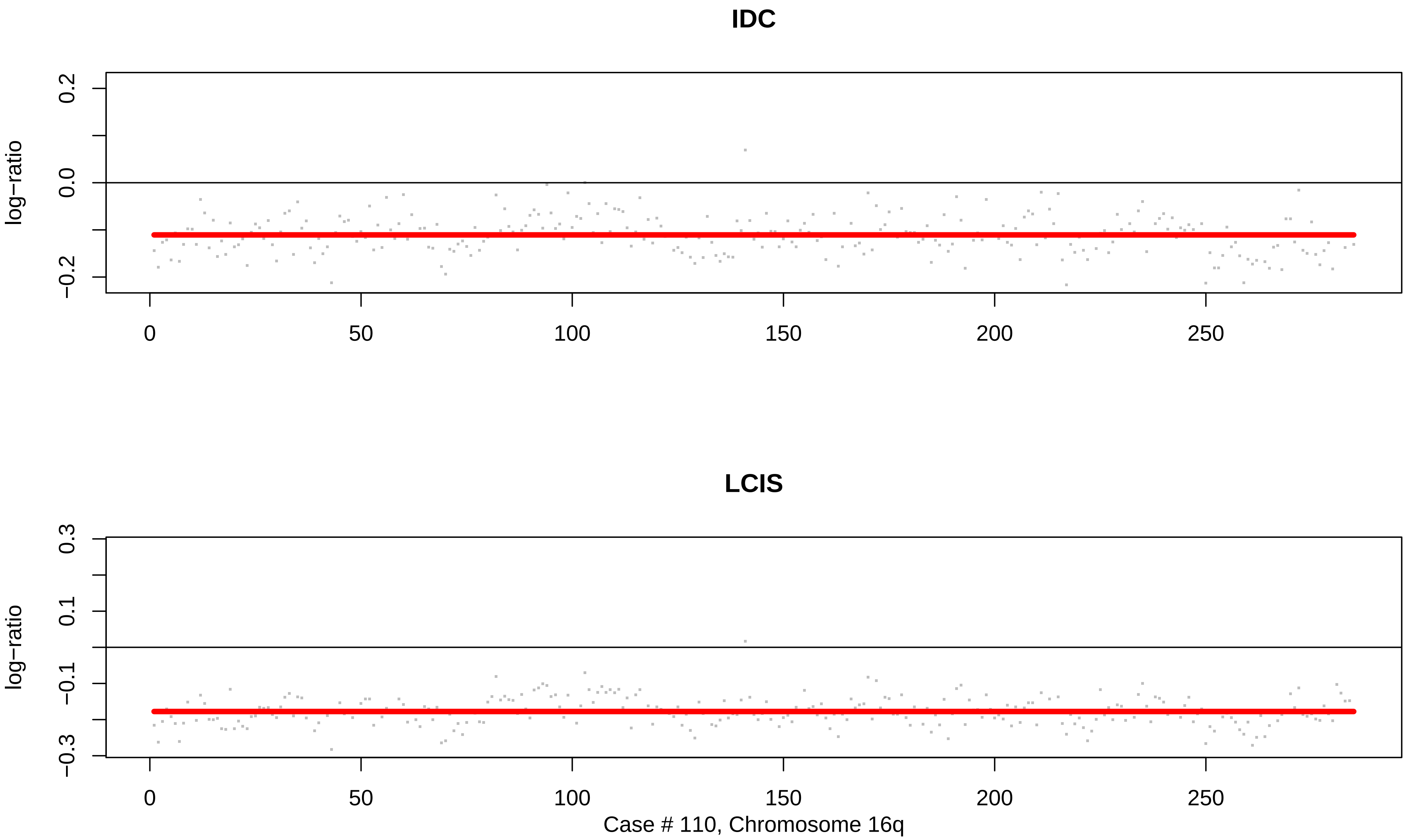
IDC
log−ratio
0.2
0.0
−0.2
0
50
100
150
200
250
LCIS
log−ratio
0.3
0.1
−0.1
−0.3
0
50
100
150
200
250
Case # 110, Chromosome 16q

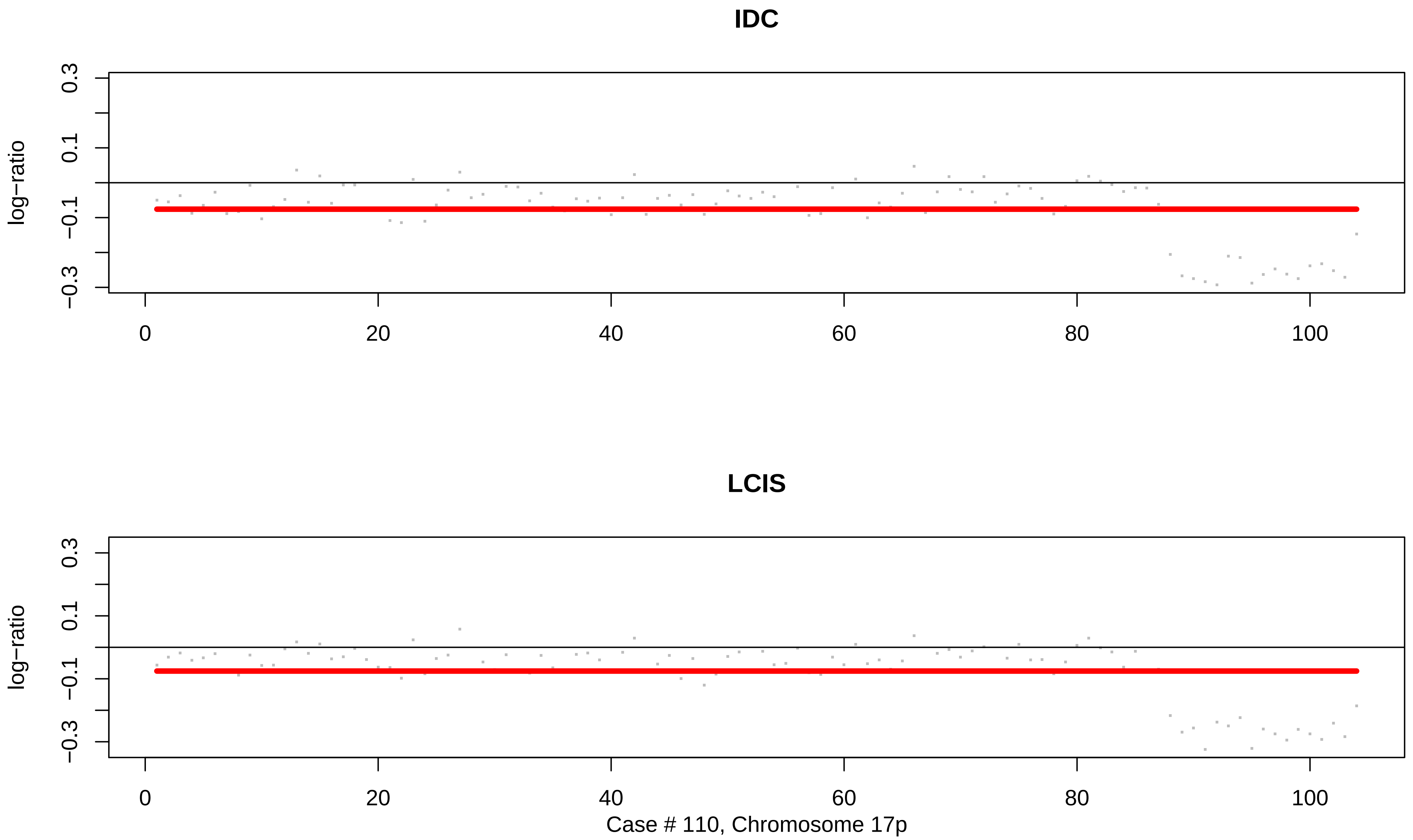

IDC
log−ratio
0.3
0.1
−0.1
−0.3
0
20
40
60
80
100
LCIS
log−ratio
0.3
0.1
−0.1
−0.3
0
20
40
60
80
100
Case # 110, Chromosome 17p

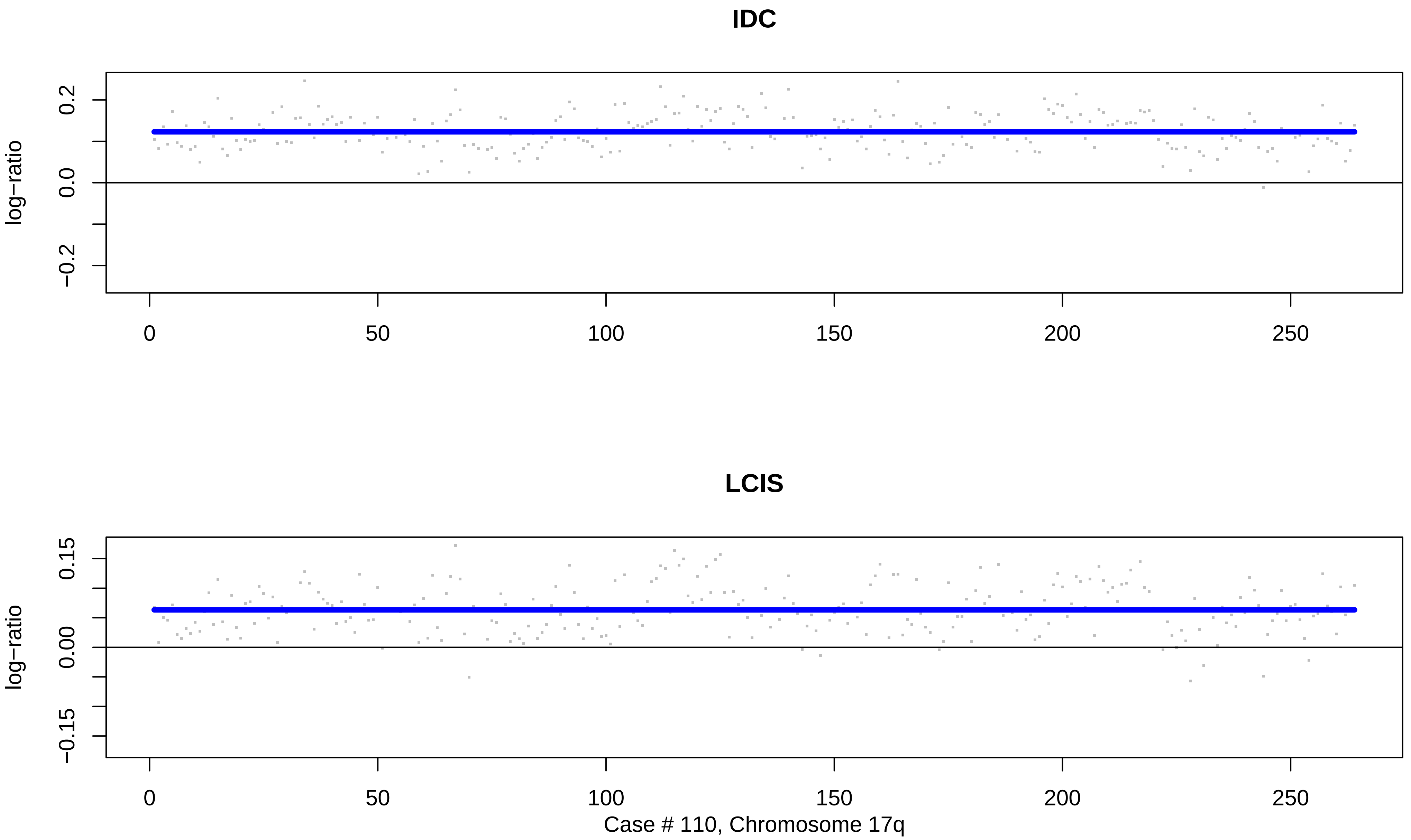
IDC
log−ratio
0.2
0.0
−0.2
0
50
100
150
200
250
LCIS
log−ratio
0.15
0.00
−0.15
0
50
100
150
200
250
Case # 110, Chromosome 17q

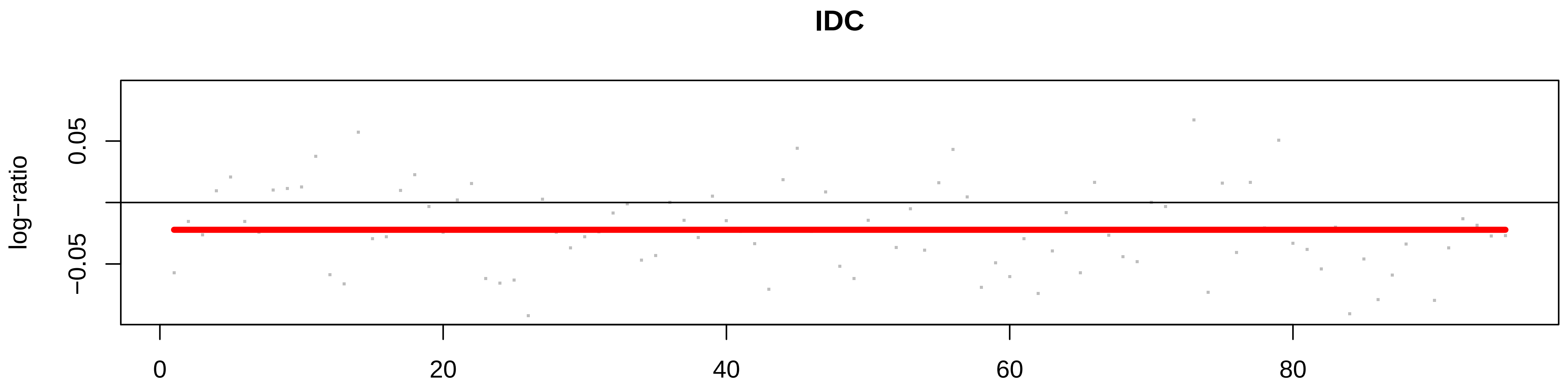
IDC
log−ratio
0.05
−0.05
0
20
40
60
80

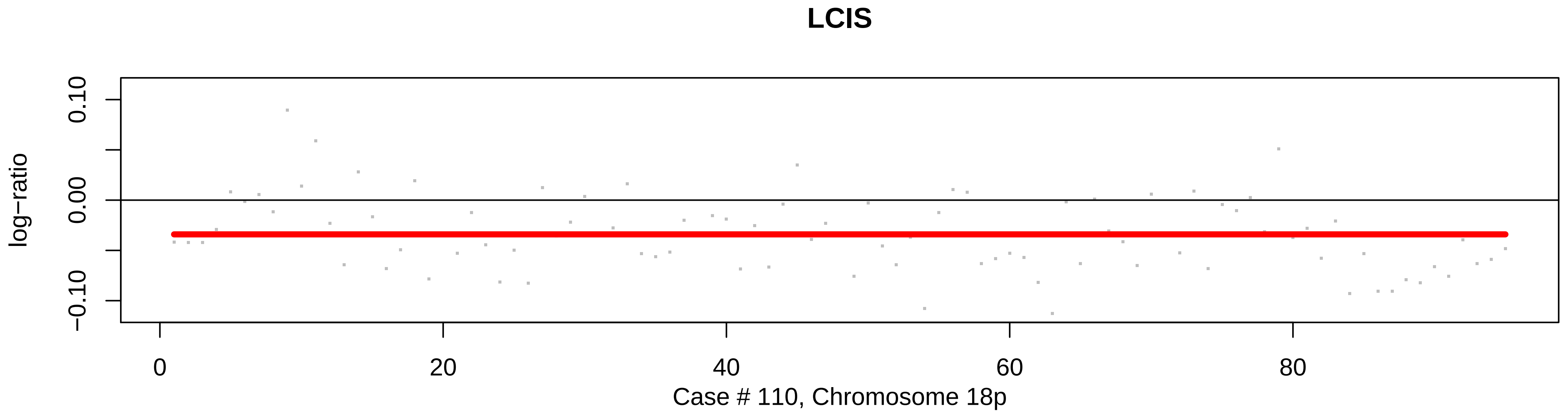
LCIS
log−ratio
0.10
0.00
−0.10
0
20
40
60
80
Case # 110, Chromosome 18p

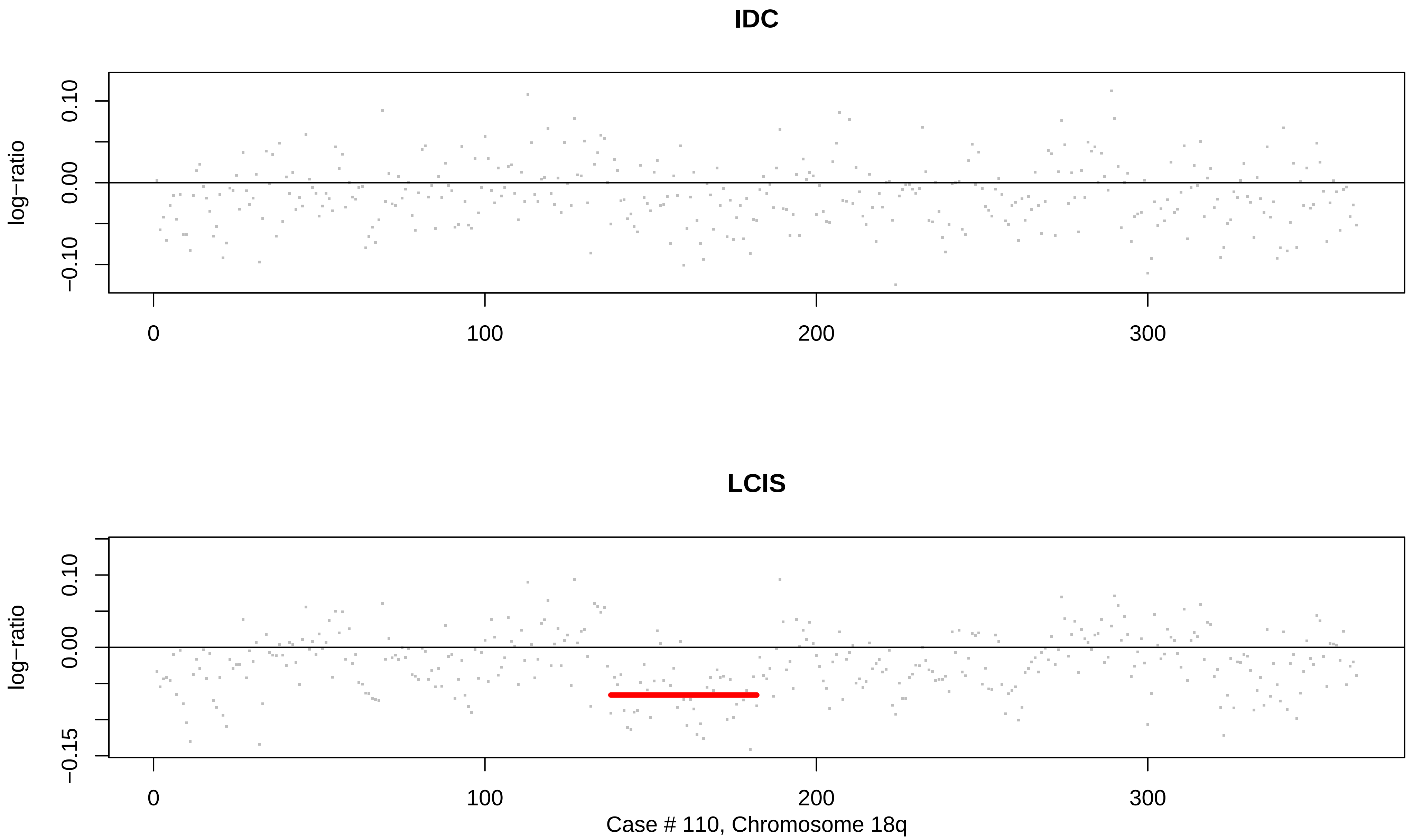
IDC
log−ratio
0.10
0.00
−0.10
0
100
200
300
LCIS
log−ratio
0.10
0.00
−0.15
0
100
200
300
Case # 110, Chromosome 18q

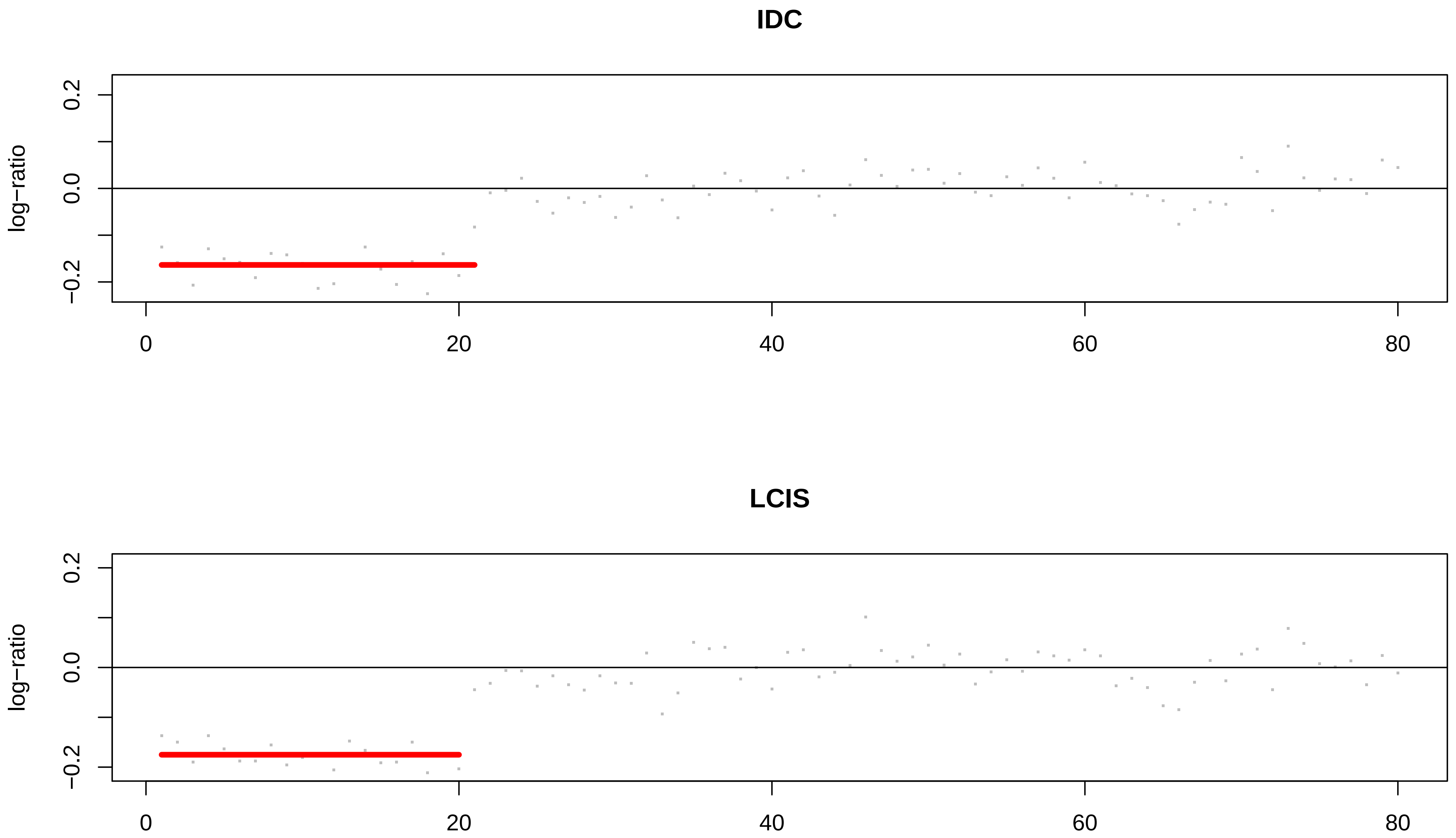

Case # 110, Chromosome 19p
Odds in favor of clonality = 3.6

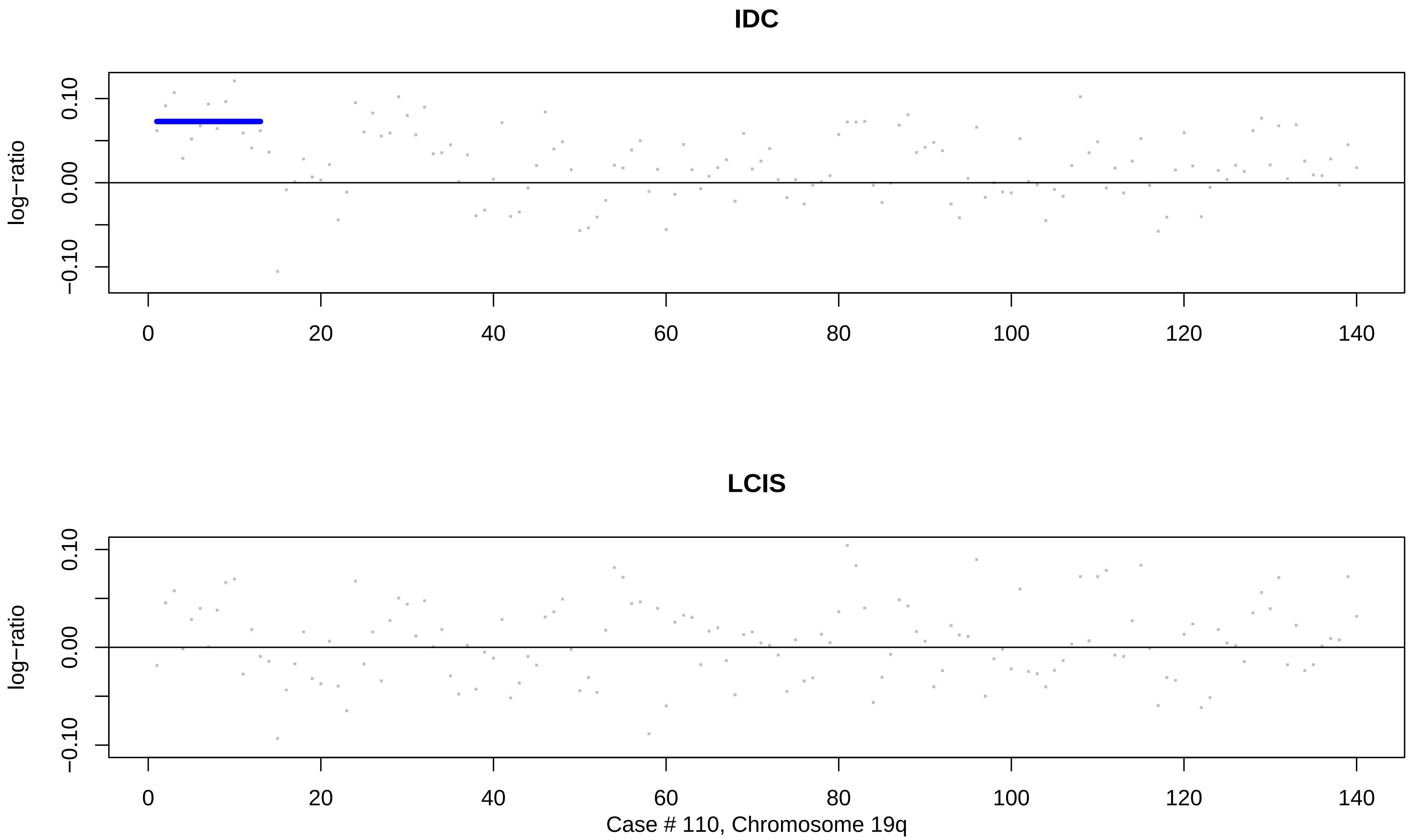
IDC
log−ratio
0.10
0.00
−0.10
0
20
40
60
80
100
120
140
LCIS
log−ratio
0.10
0.00
−0.10
0
20
40
60
80
100
120
140
Case # 110, Chromosome 19q

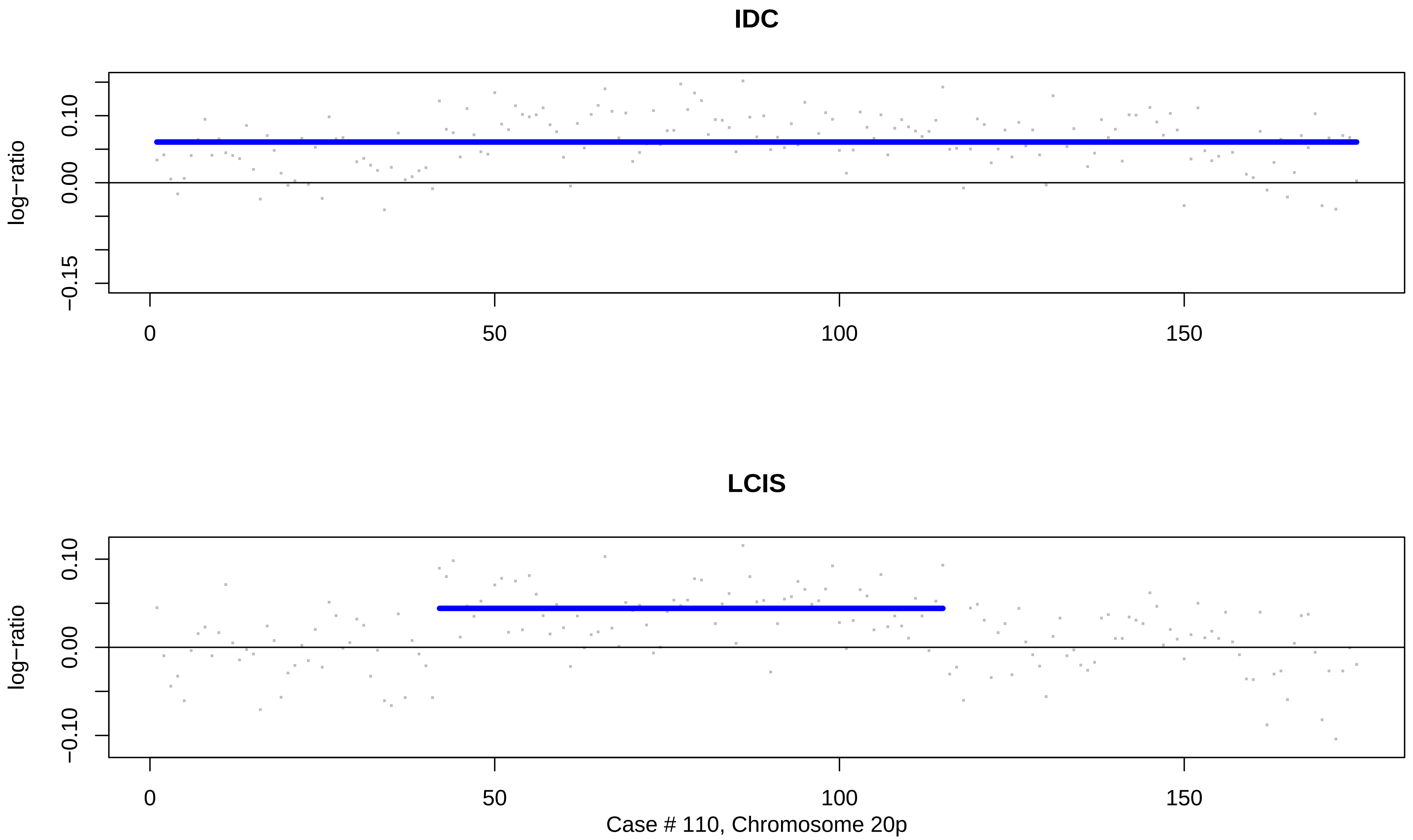
IDC
log−ratio
−0.15
0.00
0.10
0
50
100
150
LCIS
log−ratio
−0.10
0.00
0.10
0
50
100
150
Case # 110, Chromosome 20p

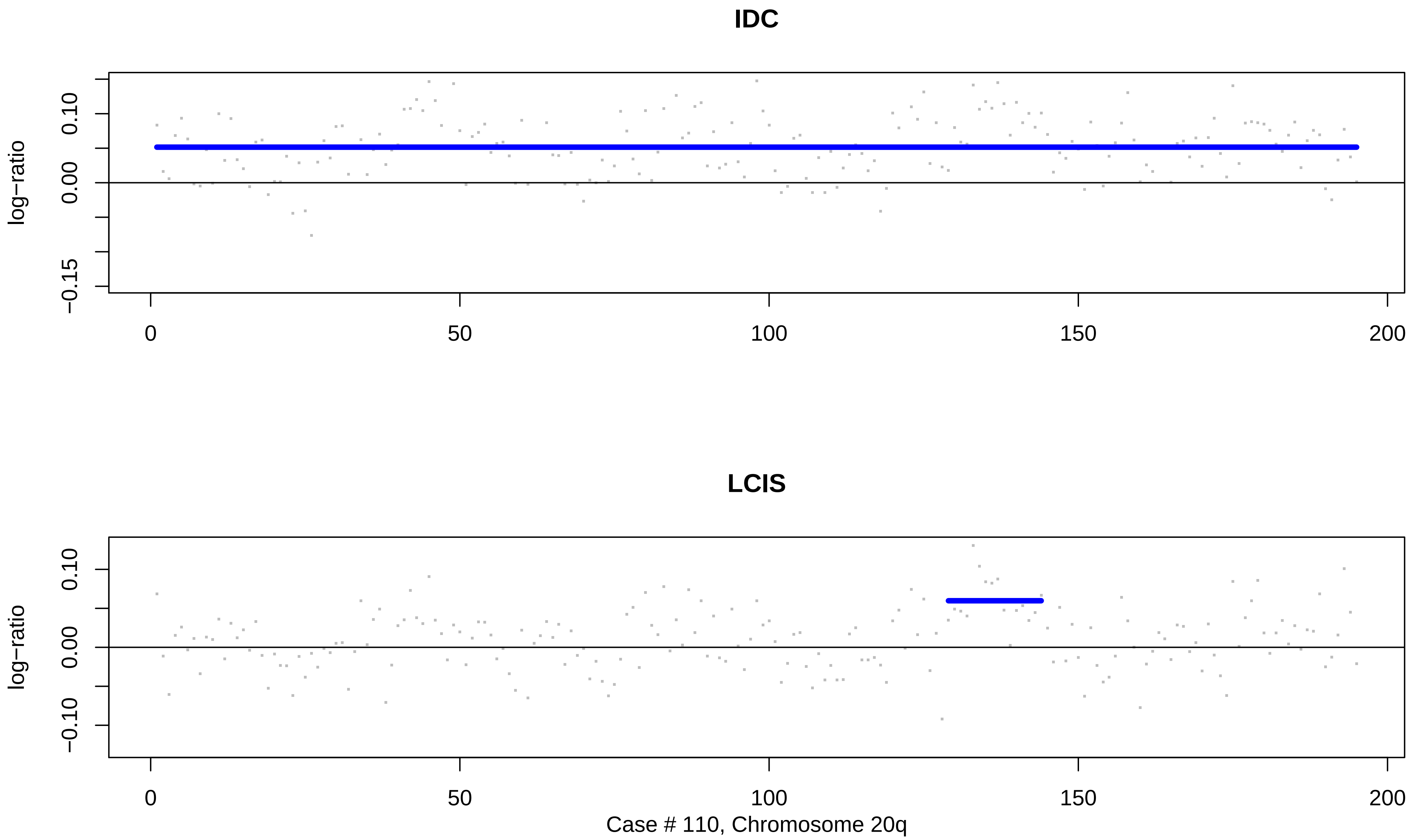
IDC
log−ratio
−0.15
0.00
0.10
0
50
100
150
200
LCIS
log−ratio
−0.10
0.00
0.10
0
50
100
150
200
Case # 110, Chromosome 20q

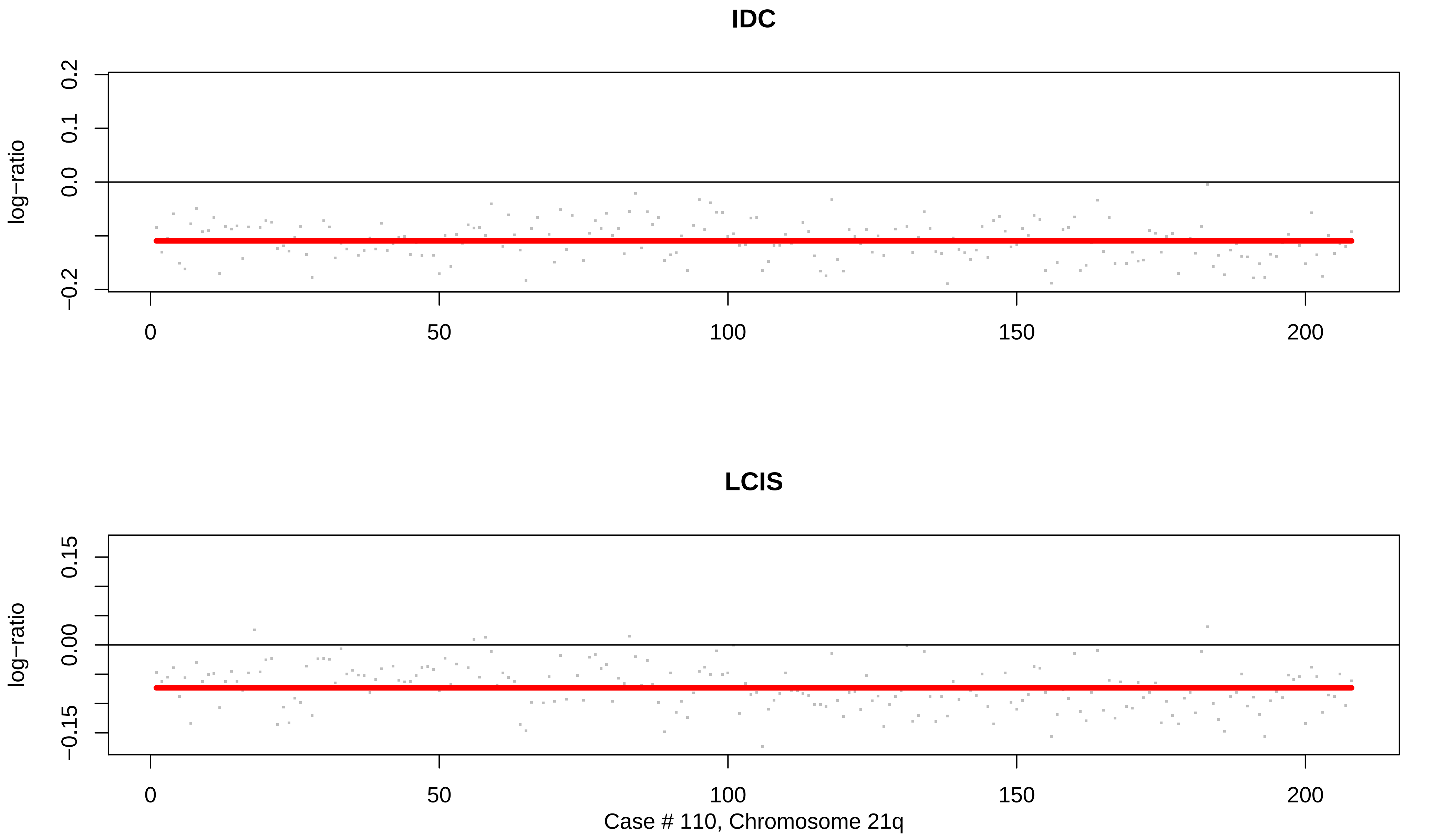
IDC
log−ratio
0.2
0.1
0.0
−0.2
0
50
100
150
200
LCIS
log−ratio
0.15
0.00
−0.15
0
50
100
150
200
Case # 110, Chromosome 21q

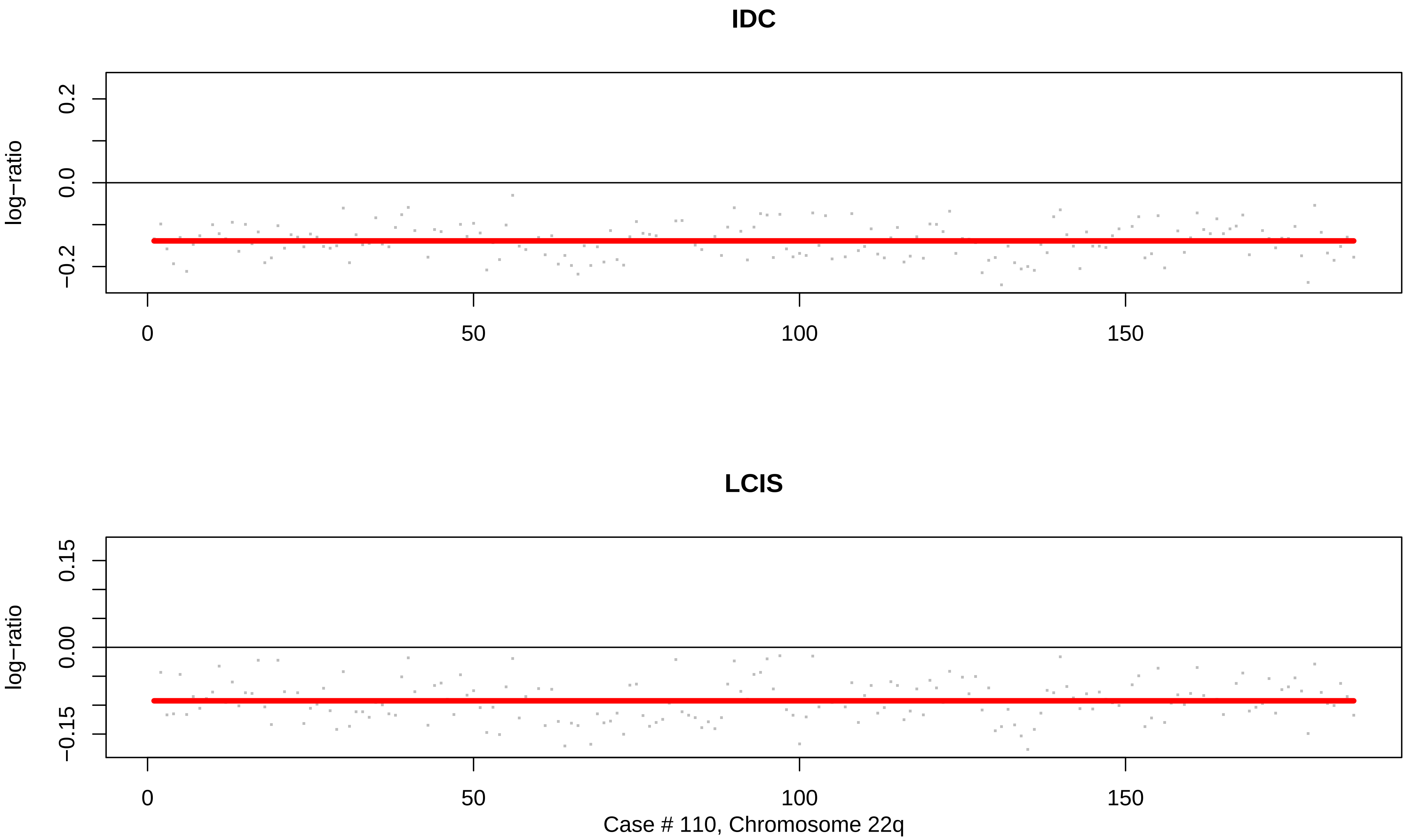
IDC
log−ratio
0.2
0.0
−0.2
0
50
100
150
LCIS
log−ratio
0.15
0.00
−0.15
0
50
100
150
Case # 110, Chromosome 22q
