# Supplementary material for: Clonal relatedness between lobular carcinoma in situ and synchronous malignant lesions
Source: Breast Cancer Res. 2012 Jul 9;14(4):R103. doi: 10.1186/bcr3222 (PMC3680923; doi:10.1186/bcr3222)
Supplement: Additional file 4 — Magnified version of genome-wide plots with detailed marker plots and segmentation on a chromosome-arm-specific basis. [file bcr3222-S4.ZIP › Case 110 DS.pdf]

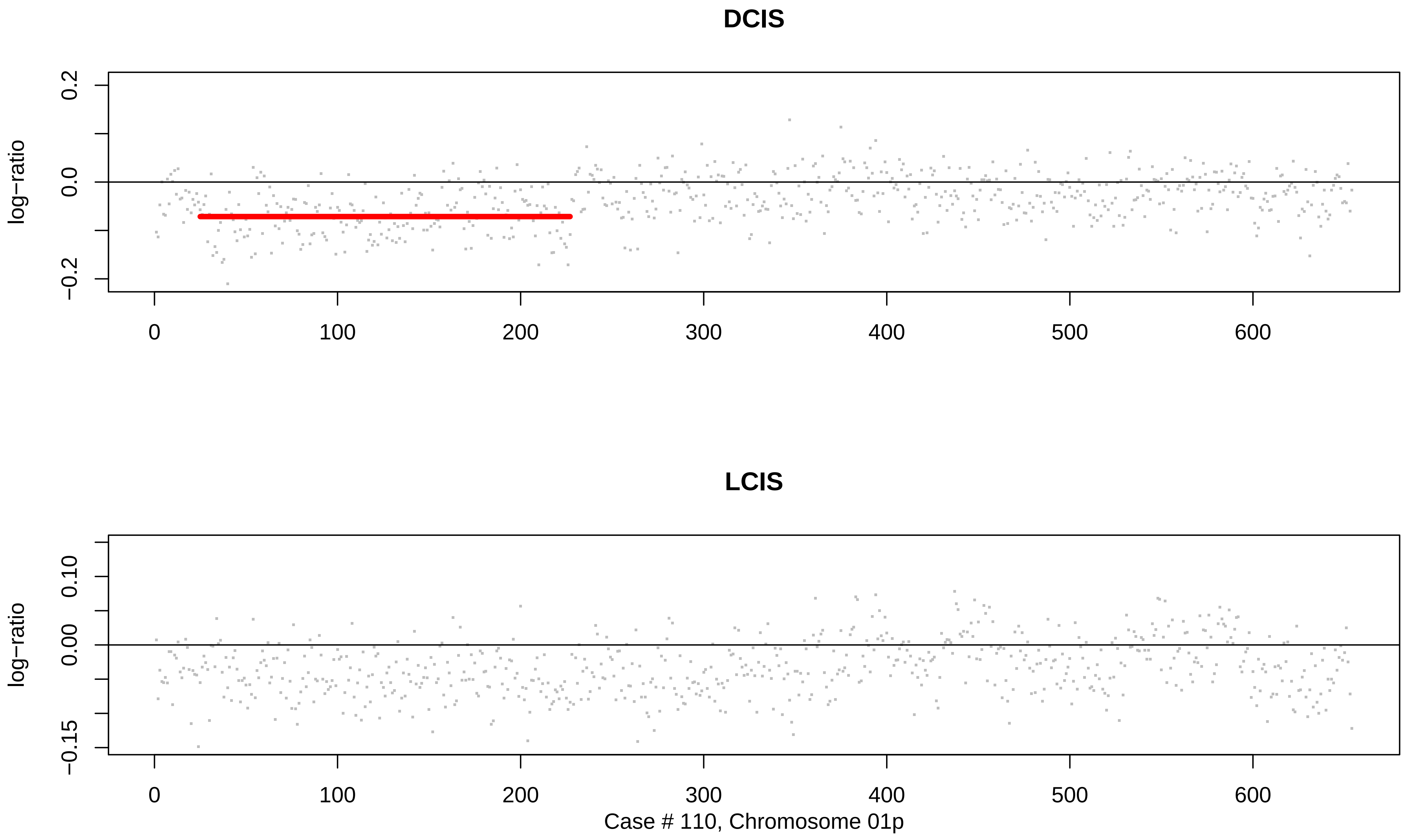
DCIS
log−ratio
0.2
0.0
−0.2
0
100
200
300
400
500
600
LCIS
log−ratio
0.10
0.00
−0.15
0
100
200
300
400
500
600
Case # 110, Chromosome 01p

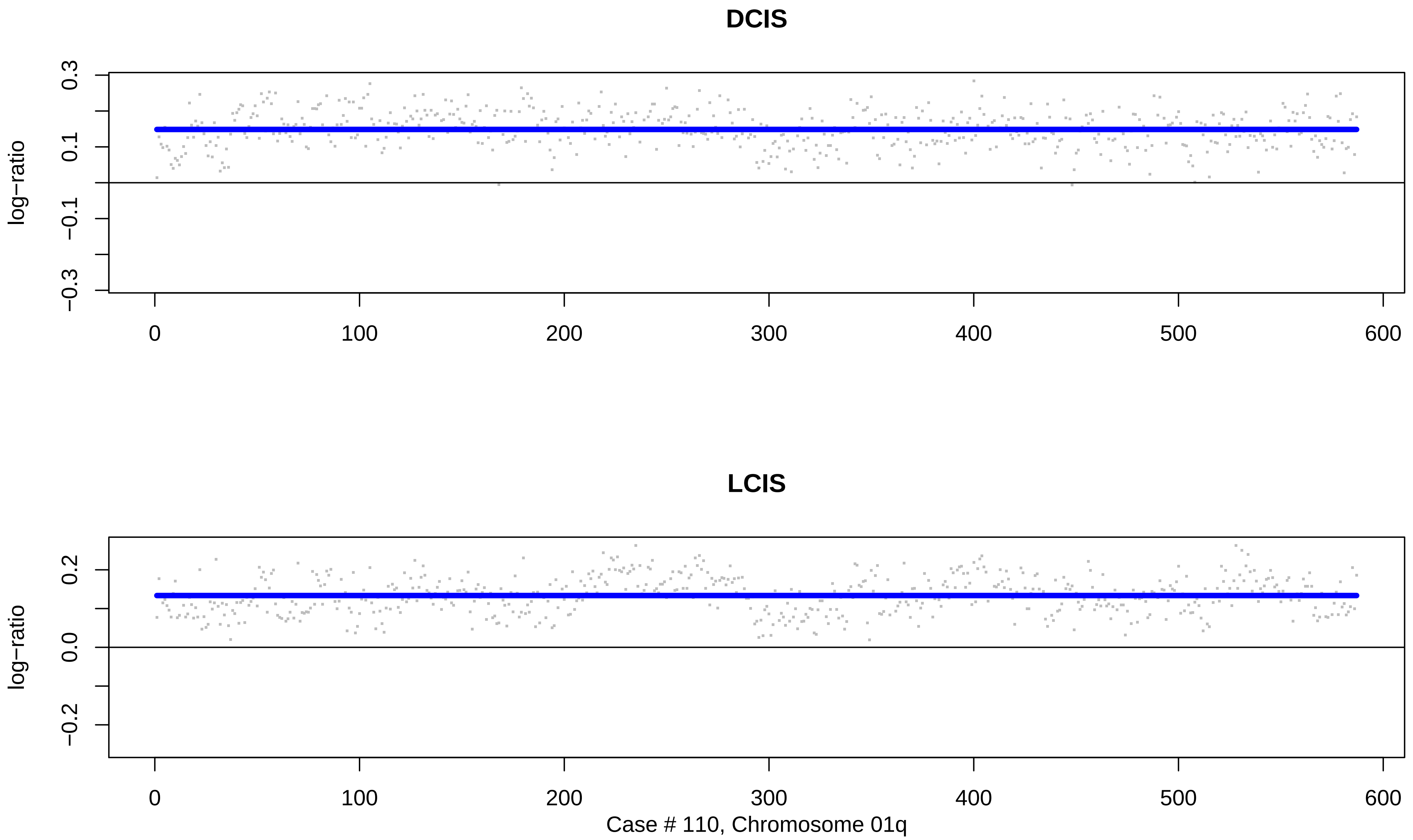
DCIS
log−ratio
0.3
0.1
−0.1
−0.3
0
100
200
300
400
500
600
LCIS
log−ratio
0.2
0.0
−0.2
0
100
200
300
400
500
600
Case # 110, Chromosome 01q

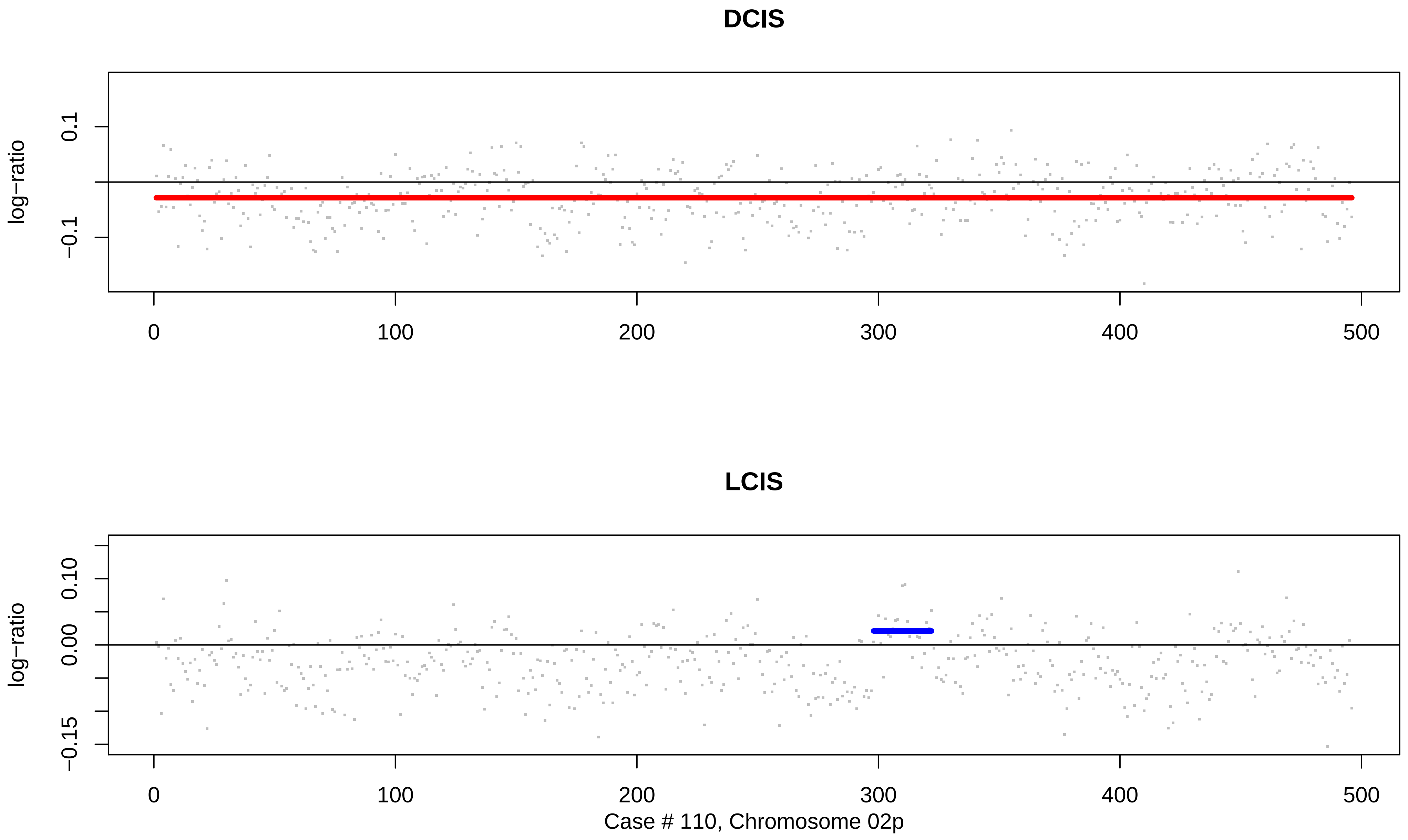
DCIS
log−ratio
0.1
−0.1
0
100
200
300
400
500
LCIS
log−ratio
0.10
0.00
−0.15
0
100
200
300
400
500
Case # 110, Chromosome 02p

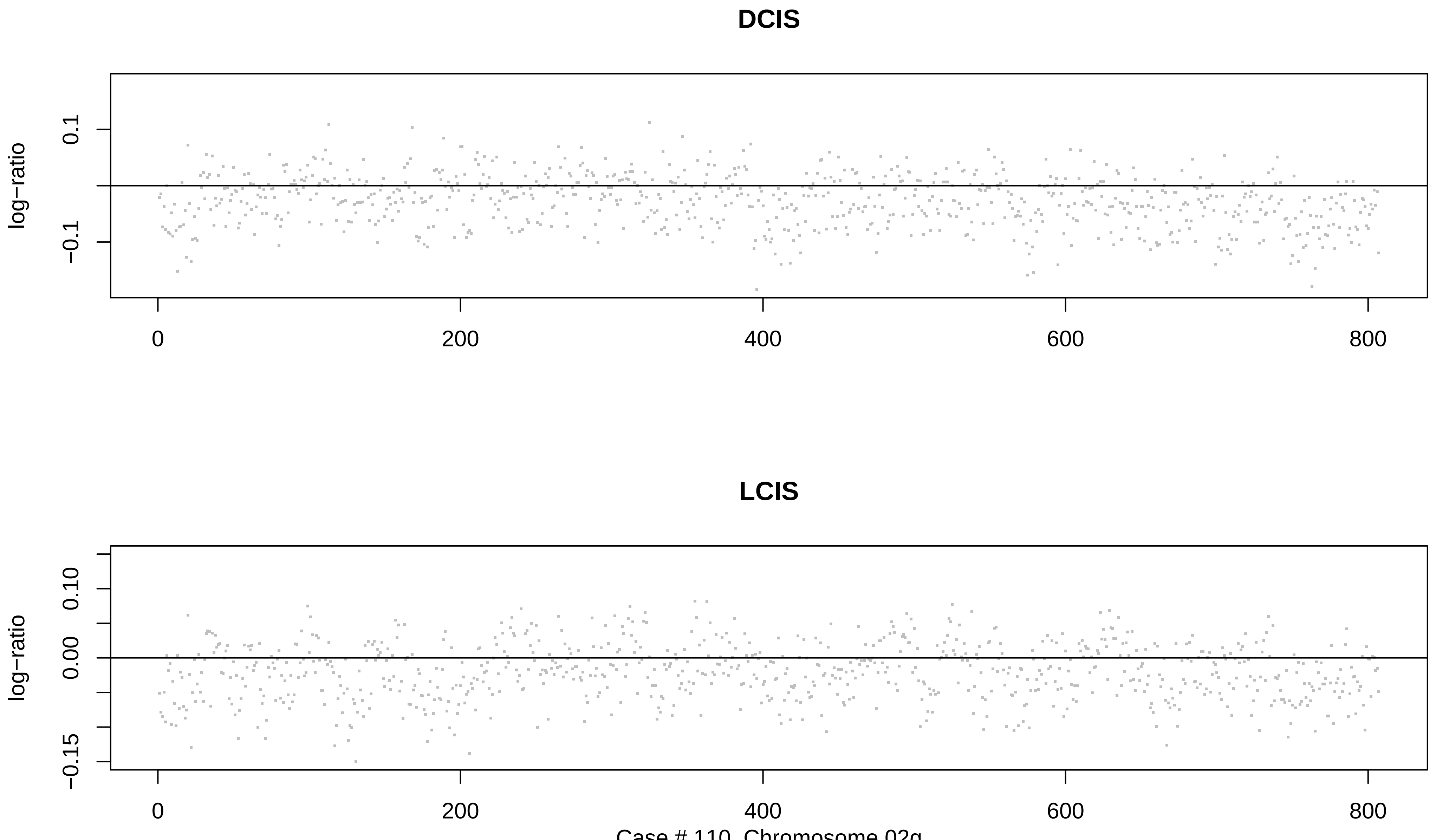
DCIS
log−ratio
0.1
−0.1
0
200
400
600
800
LCIS
log−ratio
0.10
0.00
−0.15
0
200
400
600
800
Case # 110, Chromosome 02q

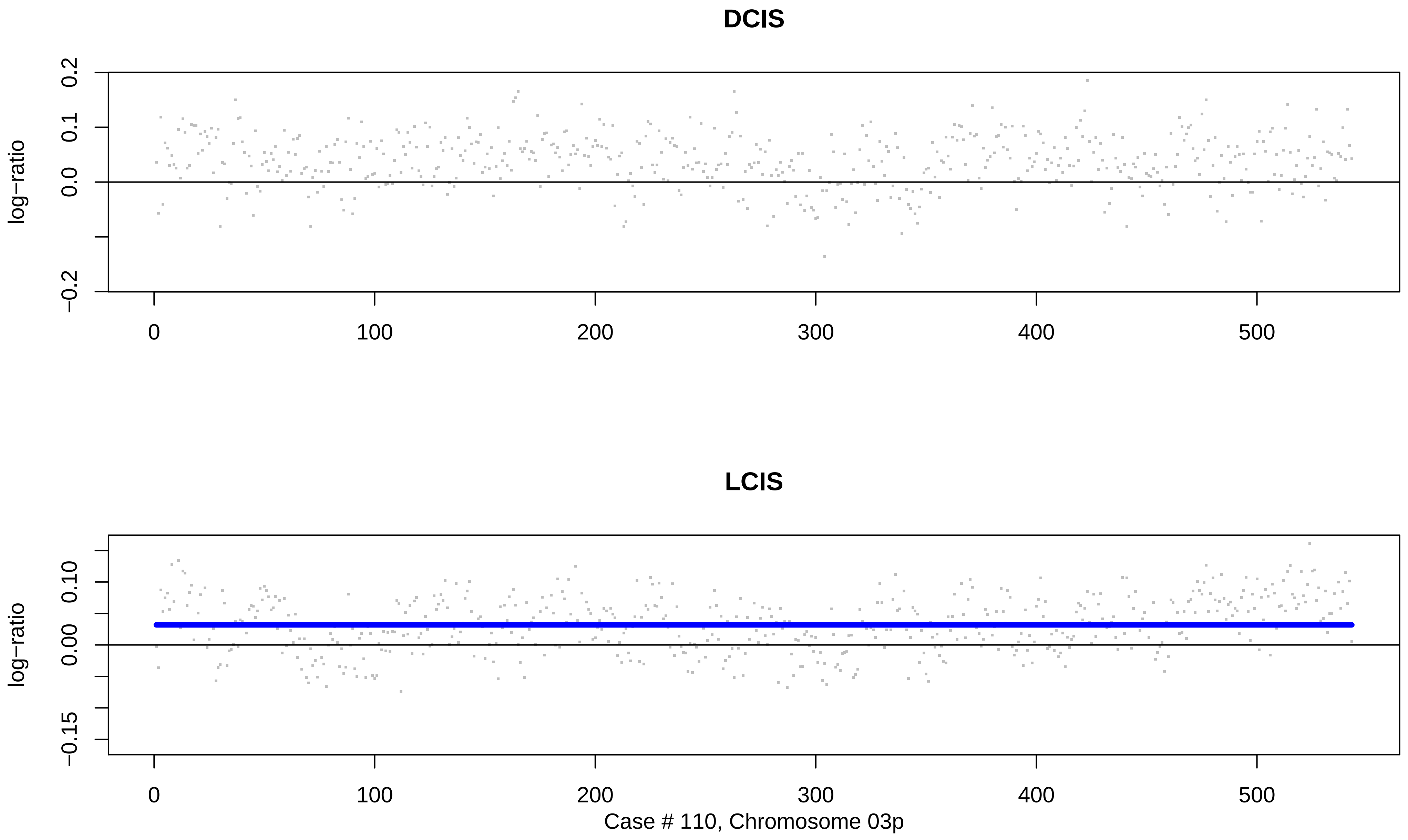

DCIS
log−ratio
0.2
0.1
0.0
−0.2
0
100
200
300
400
500
LCIS
log−ratio
0.10
0.00
−0.15
0
100
200
300
400
500
Case # 110, Chromosome 03p

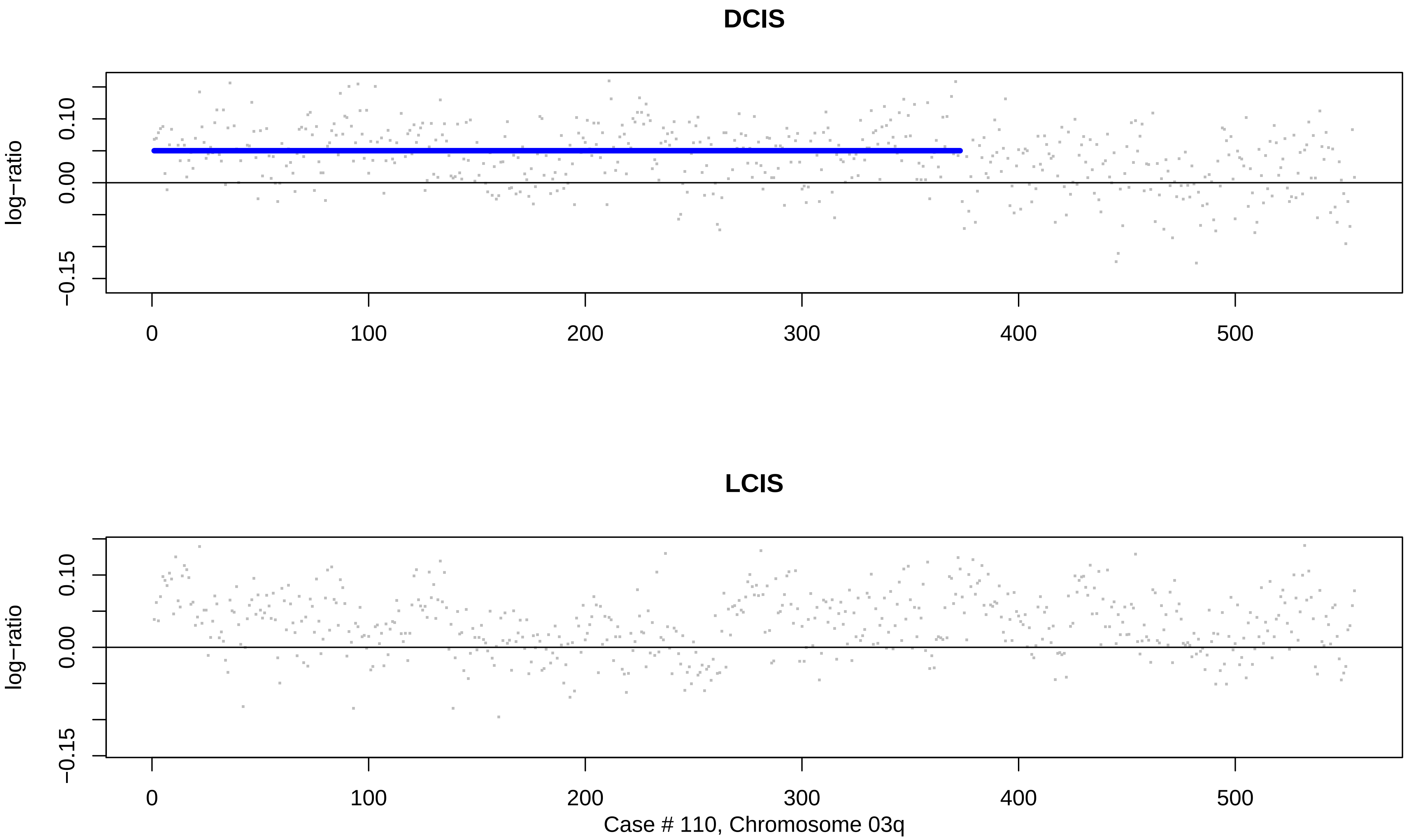
DCIS
log−ratio
−0.15
0.00
0.10
0
100
200
300
400
500
LCIS
log−ratio
−0.15
0.00
0.10
0
100
200
300
400
500
Case # 110, Chromosome 03q

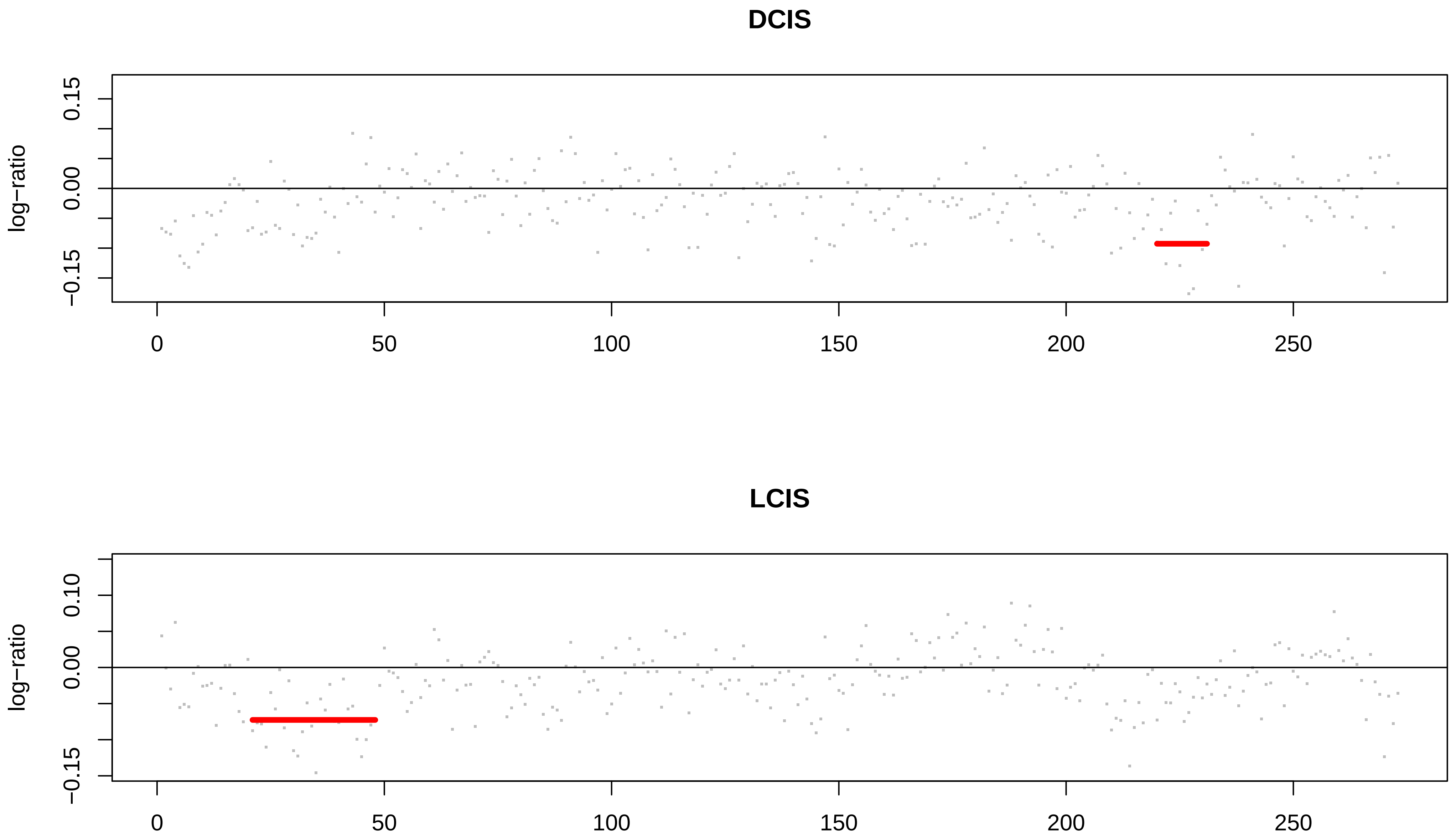

Case # 110, Chromosome 04p
Odds in favor of independence = 2.6

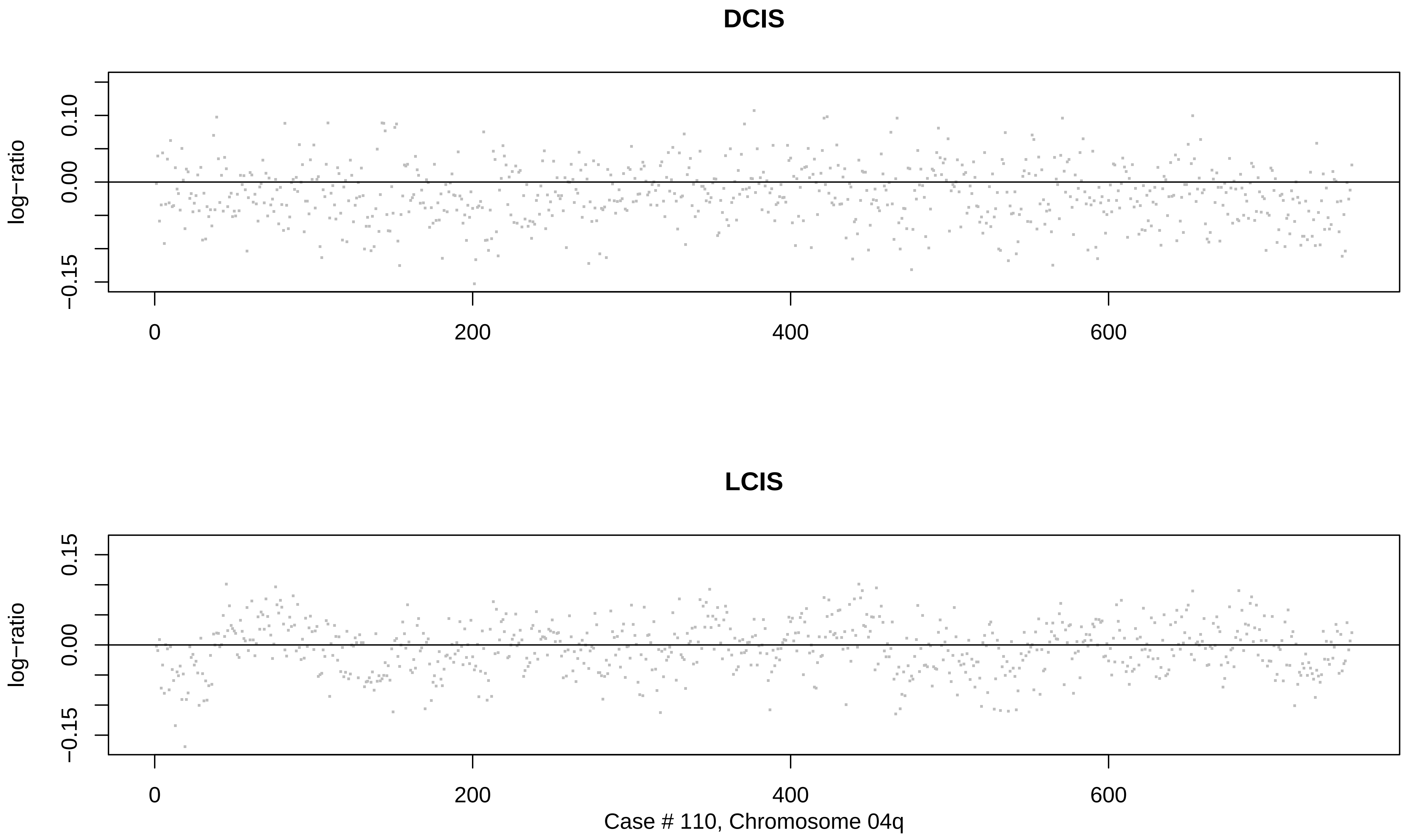
DCIS
log−ratio
0.10
0.00
−0.15
0
200
400
600
LCIS
log−ratio
0.15
0.00
−0.15
0
200
400
600
Case # 110, Chromosome 04q

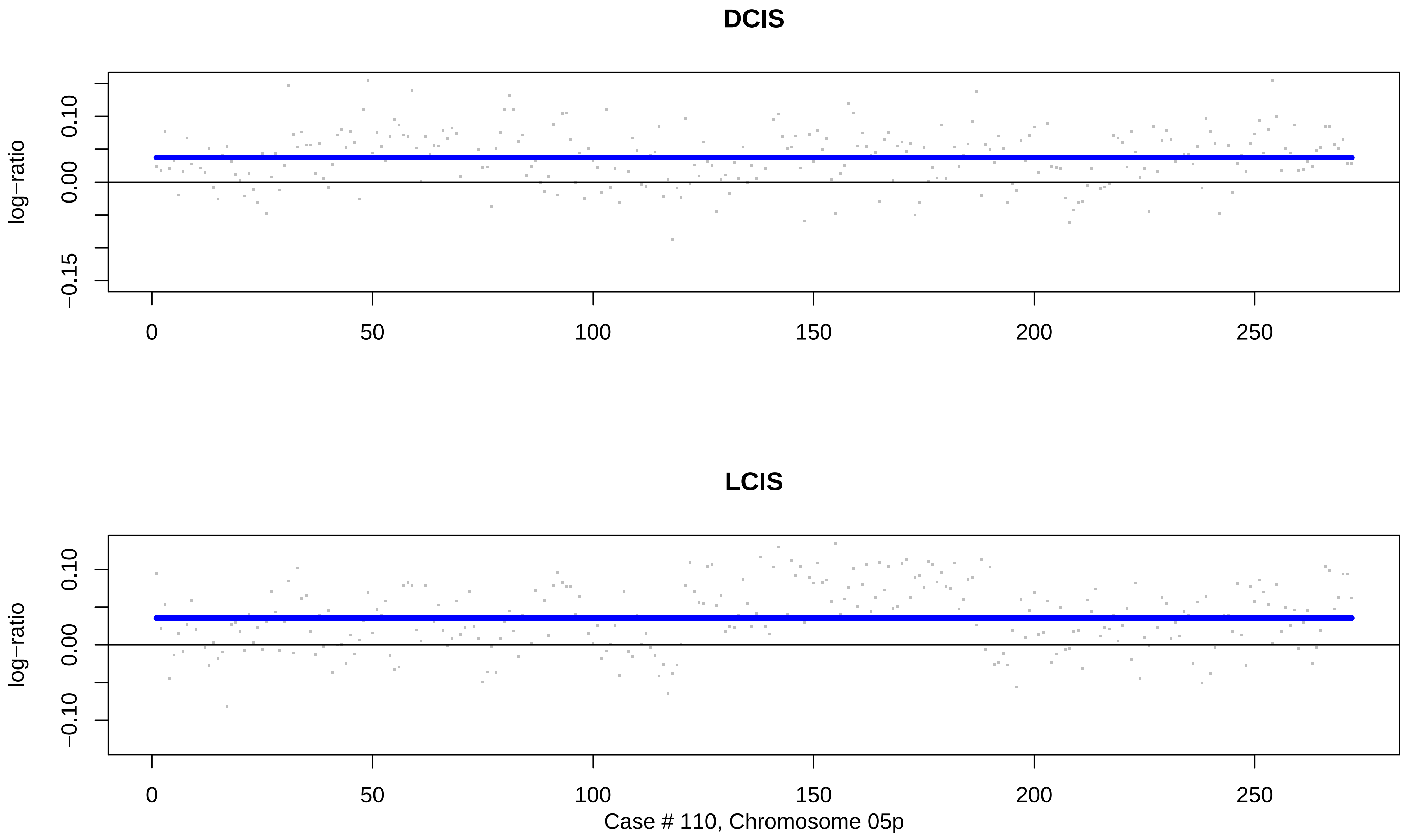
DCIS
log−ratio
−0.15
0.00
0.10
0
50
100
150
200
250
LCIS
log−ratio
−0.10
0.00
0.10
0
50
100
150
200
250
Case # 110, Chromosome 05p

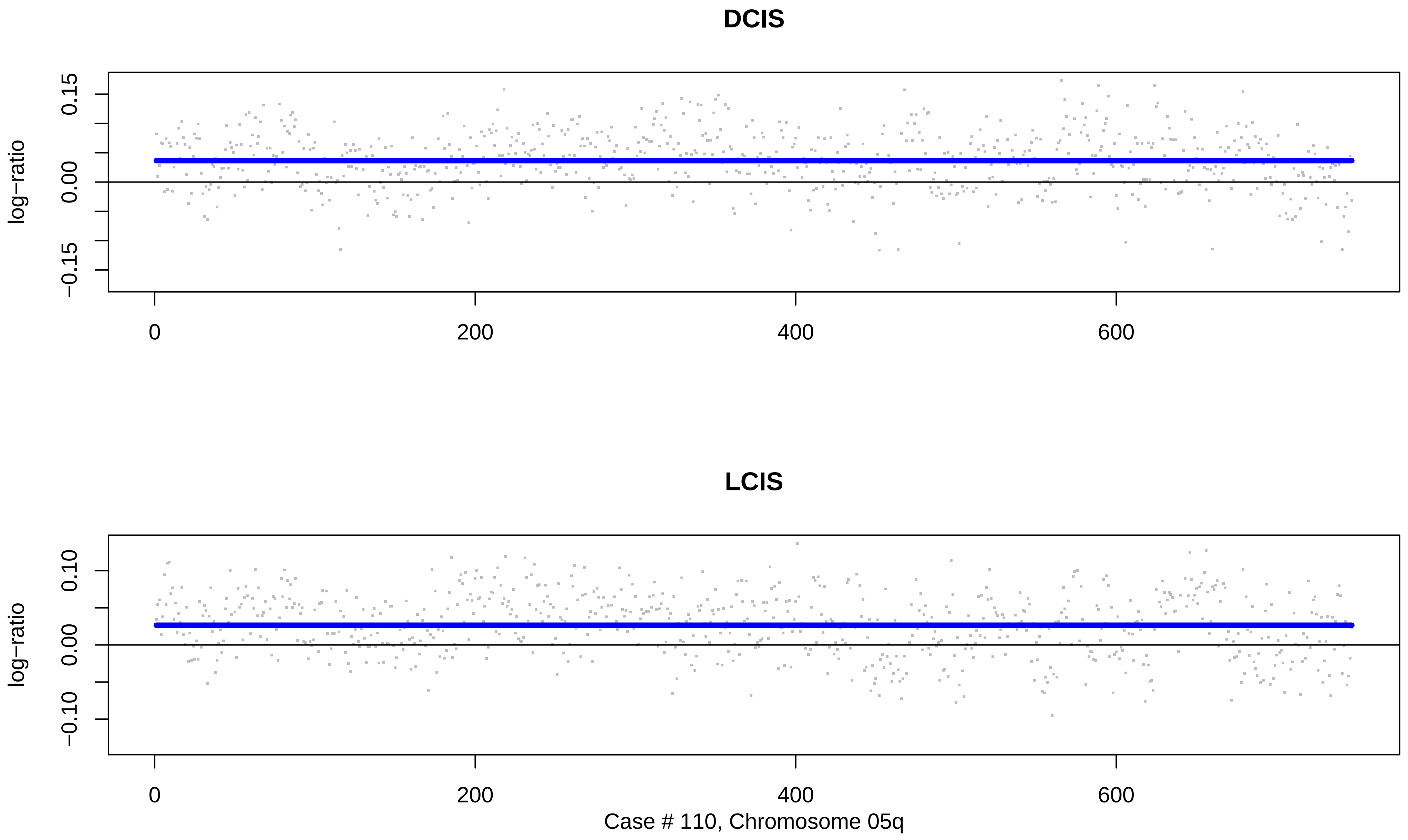
DCIS
log−ratio
0.15
0.00
−0.15
0
200
400
600
LCIS
log−ratio
0.10
0.00
−0.10
0
200
400
600
Case # 110, Chromosome 05q

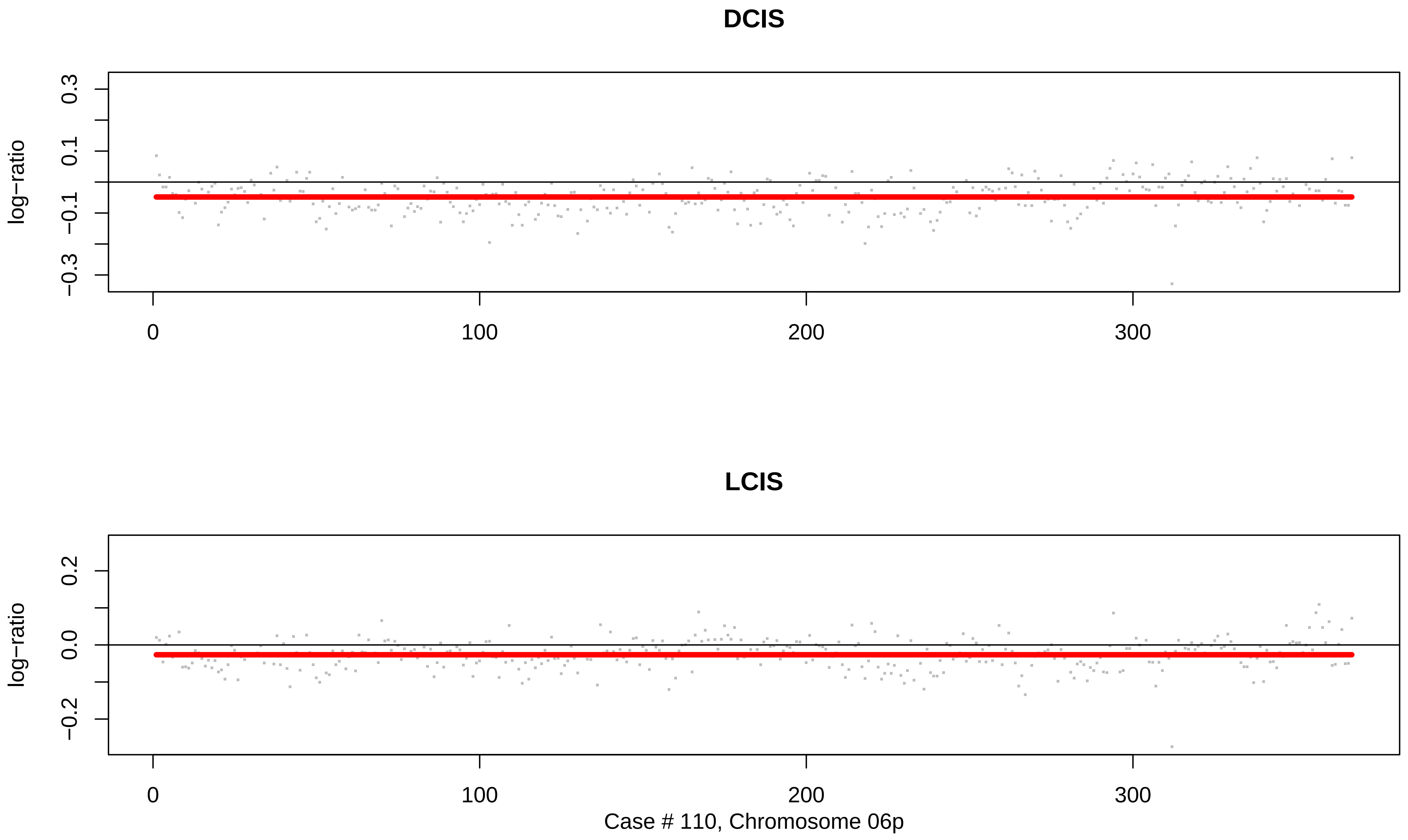

DCIS
log−ratio
0.3
0.1
−0.1
−0.3
0
100
200
300
LCIS
log−ratio
0.2
0.0
−0.2
0
100
200
300
Case # 110, Chromosome 06p

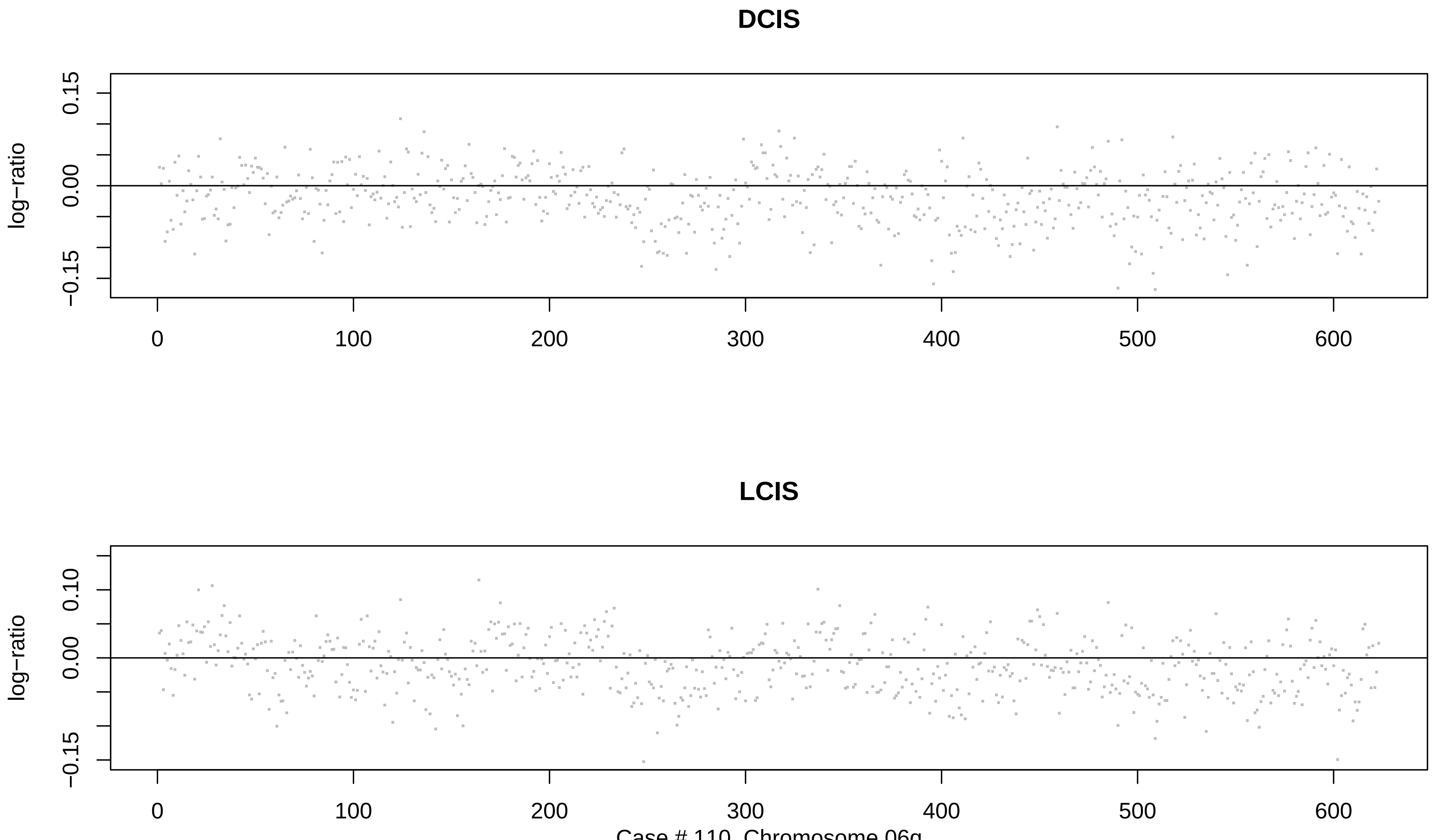
DCIS
log−ratio
0.15
0.00
−0.15
0
100
200
300
400
500
600
LCIS
log−ratio
0.10
0.00
−0.15
0
100
200
300
400
500
600
Case # 110, Chromosome 06q

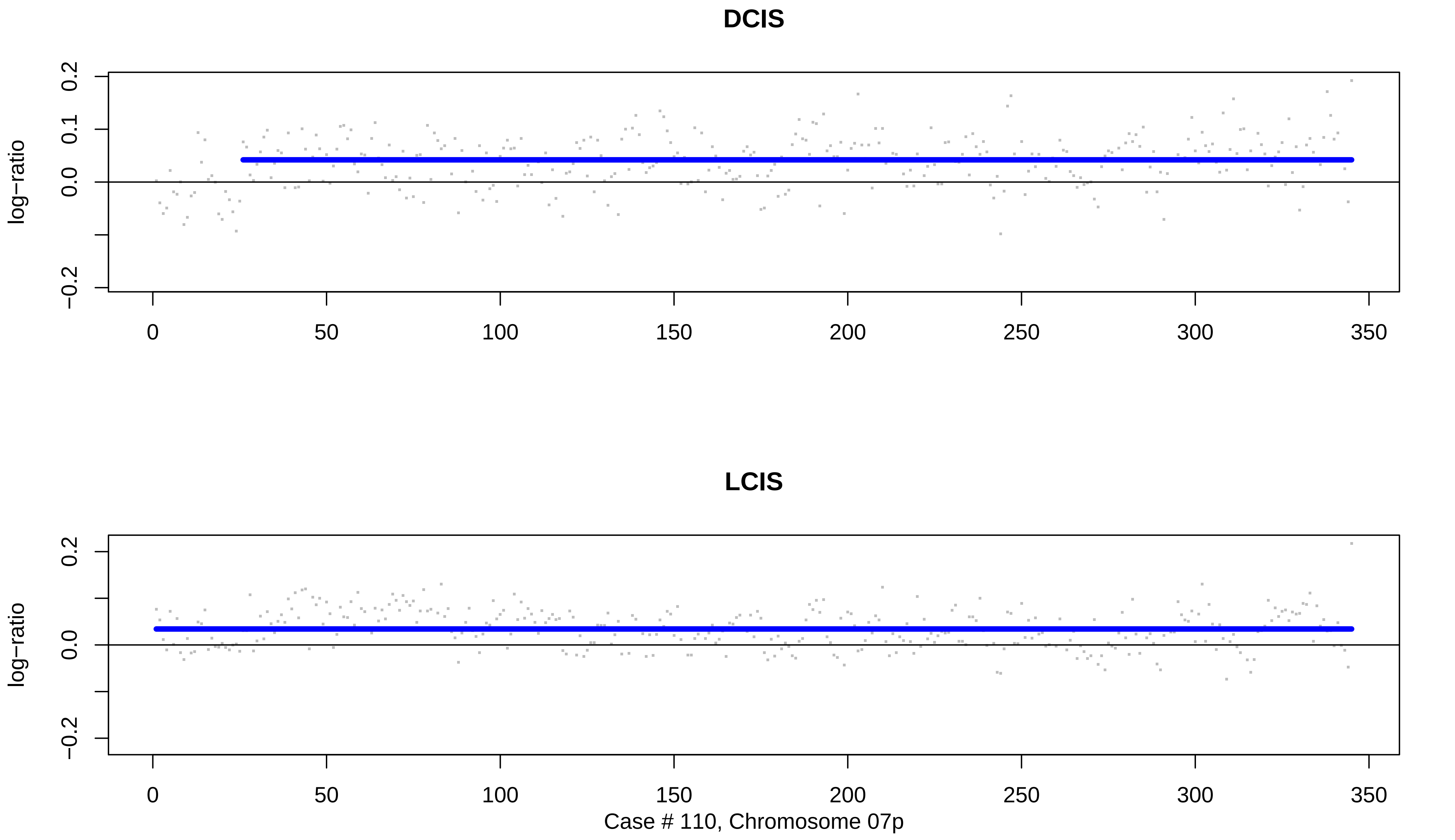
DCIS
log−ratio
0.2
0.1
0.0
−0.2
0
50
100
150
200
250
300
350
LCIS
log−ratio
0.2
0.0
−0.2
0
50
100
150
200
250
300
350
Case # 110, Chromosome 07p

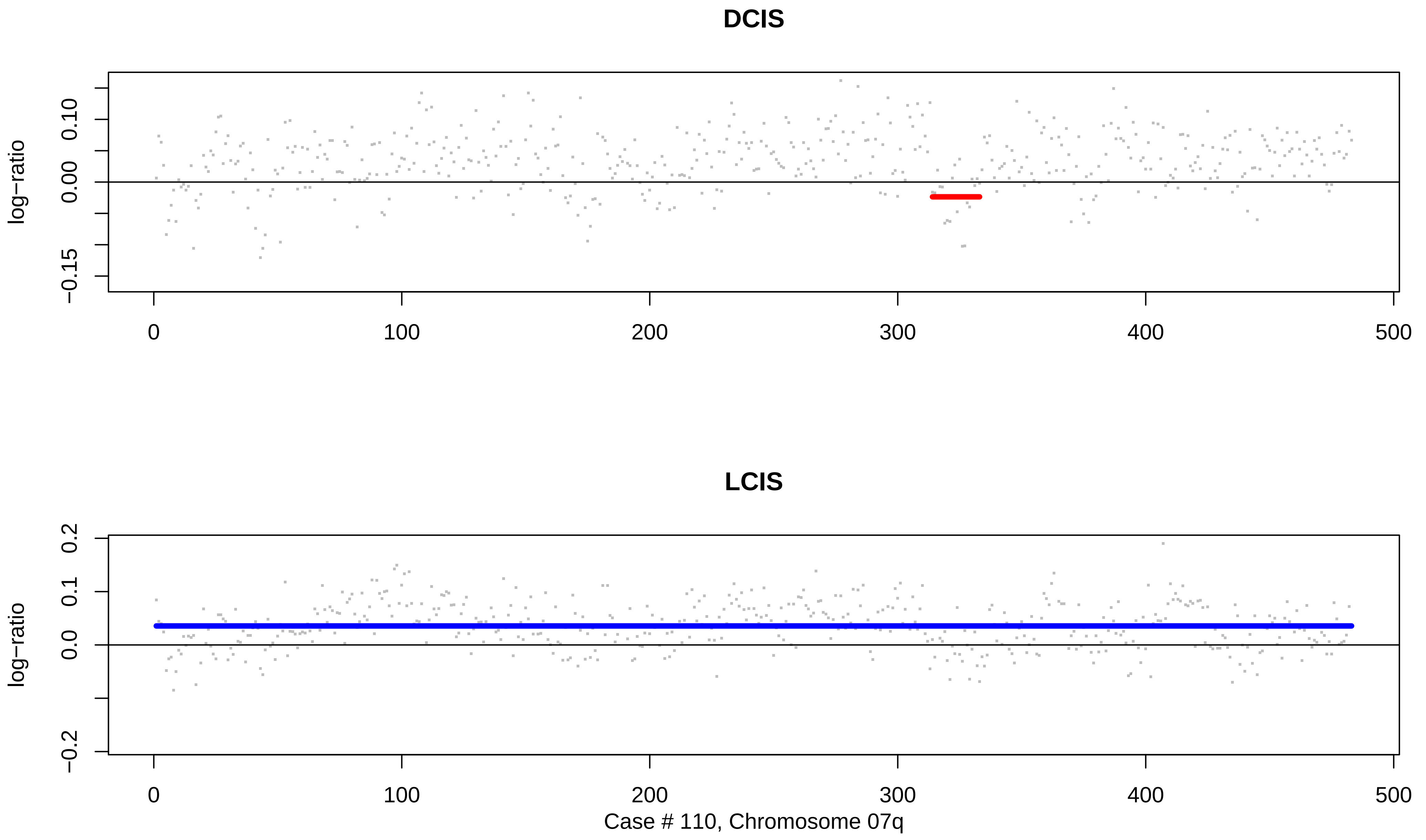

DCIS
log−ratio
−0.15
0.00
0.10
0
100
200
300
400
500
LCIS
log−ratio
−0.2
0.0
0.1
0.2
0
100
200
300
400
500
Case # 110, Chromosome 07q

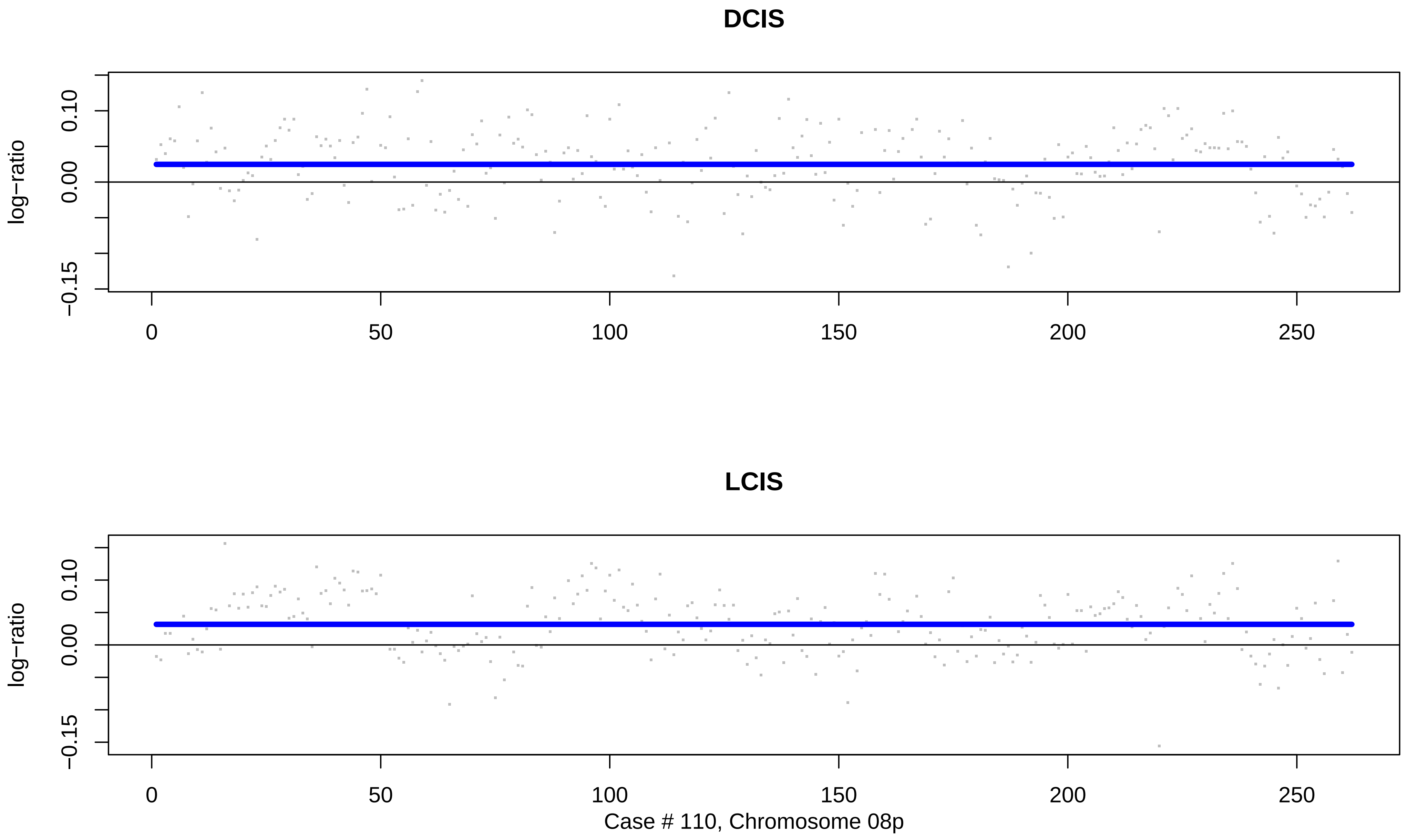
DCIS
log−ratio
−0.15
0.00
0.10
0
50
100
150
200
250
LCIS
log−ratio
−0.15
0.00
0.10
0
50
100
150
200
250
Case # 110, Chromosome 08p

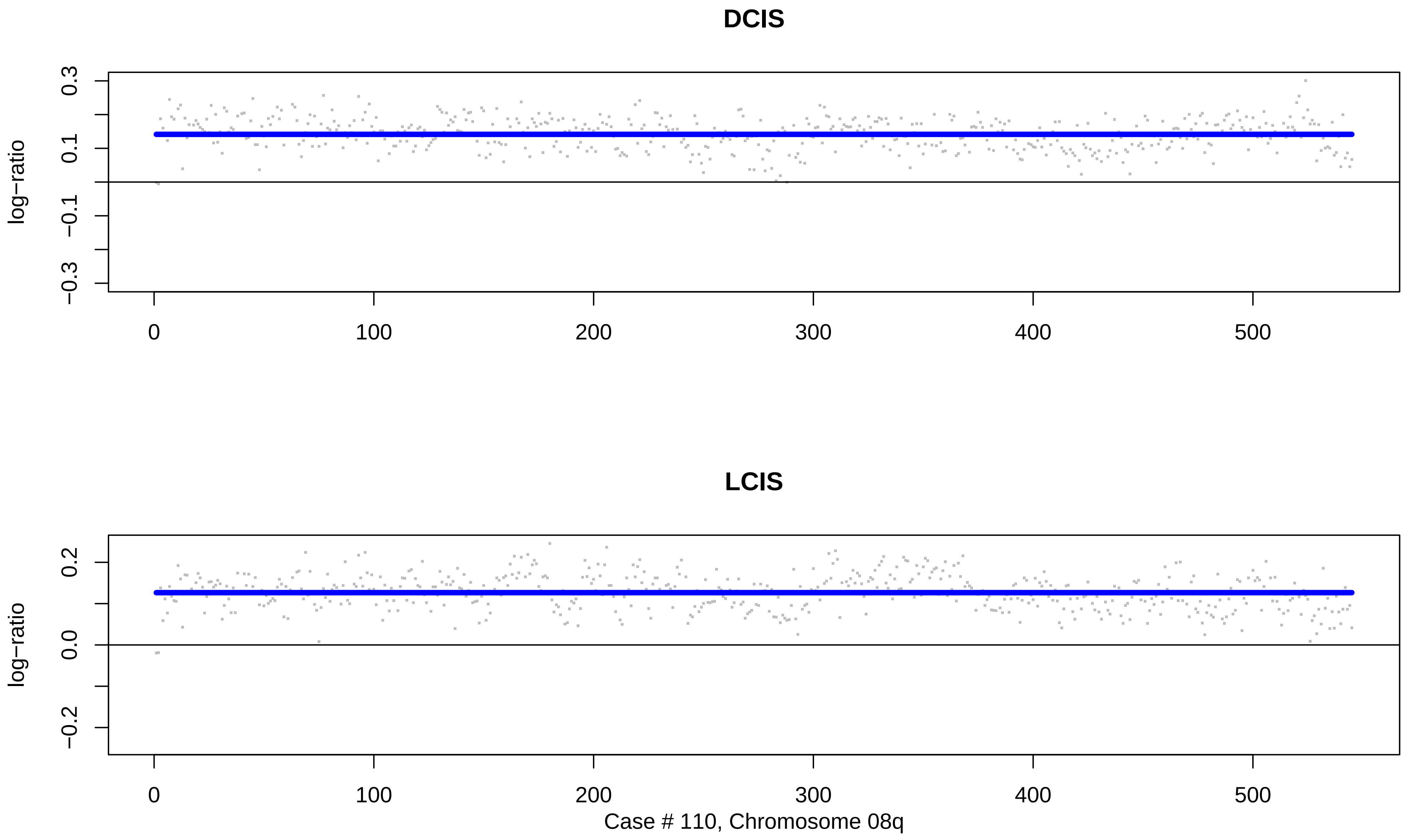
DCIS
log−ratio
0.3
0.1
−0.1
−0.3
0
100
200
300
400
500
LCIS
log−ratio
0.2
0.0
−0.2
0
100
200
300
400
500
Case # 110, Chromosome 08q

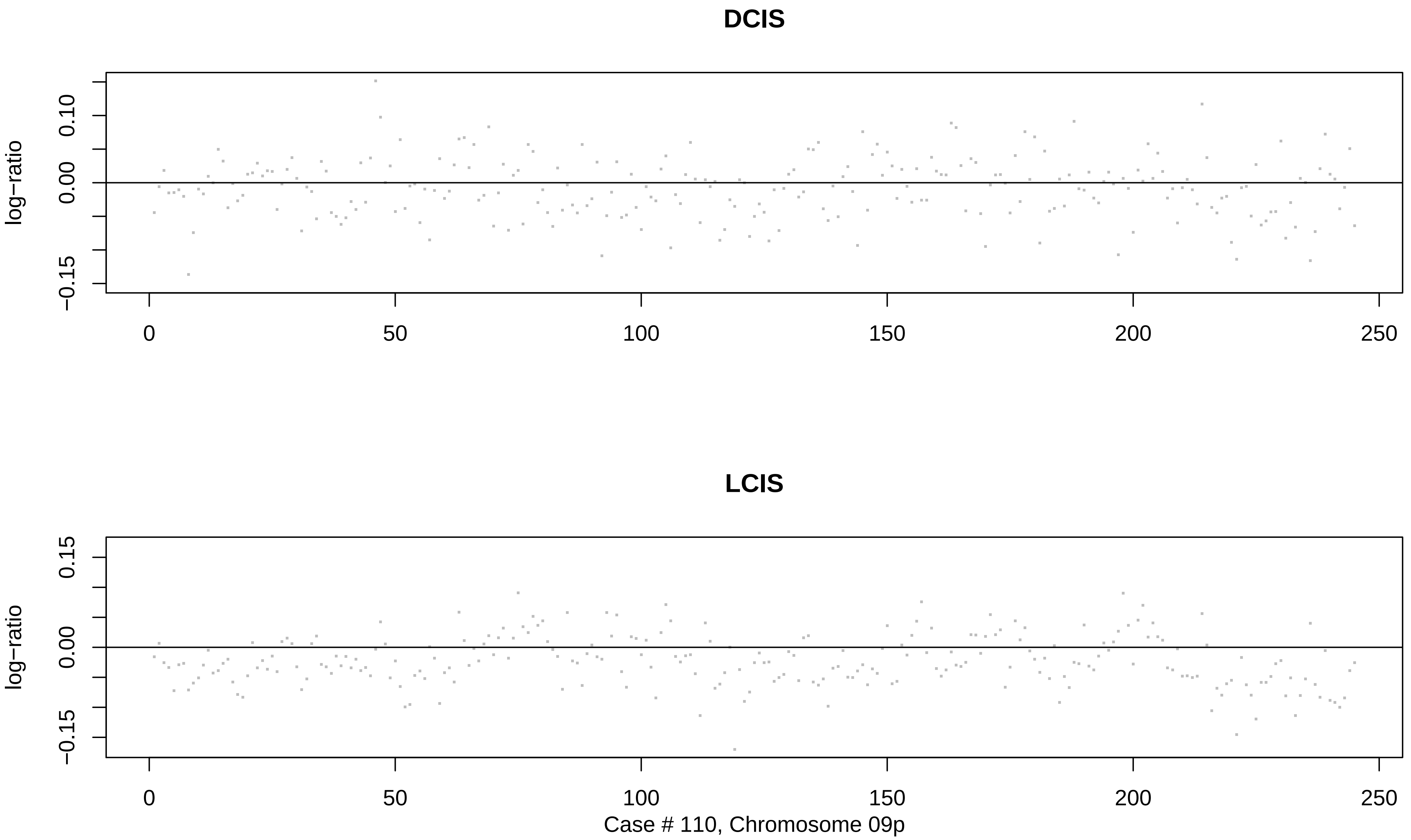
DCIS
log−ratio
0.10
0.00
−0.15
0
50
100
150
200
250
LCIS
log−ratio
0.15
0.00
−0.15
0
50
100
150
200
250
Case # 110, Chromosome 09p

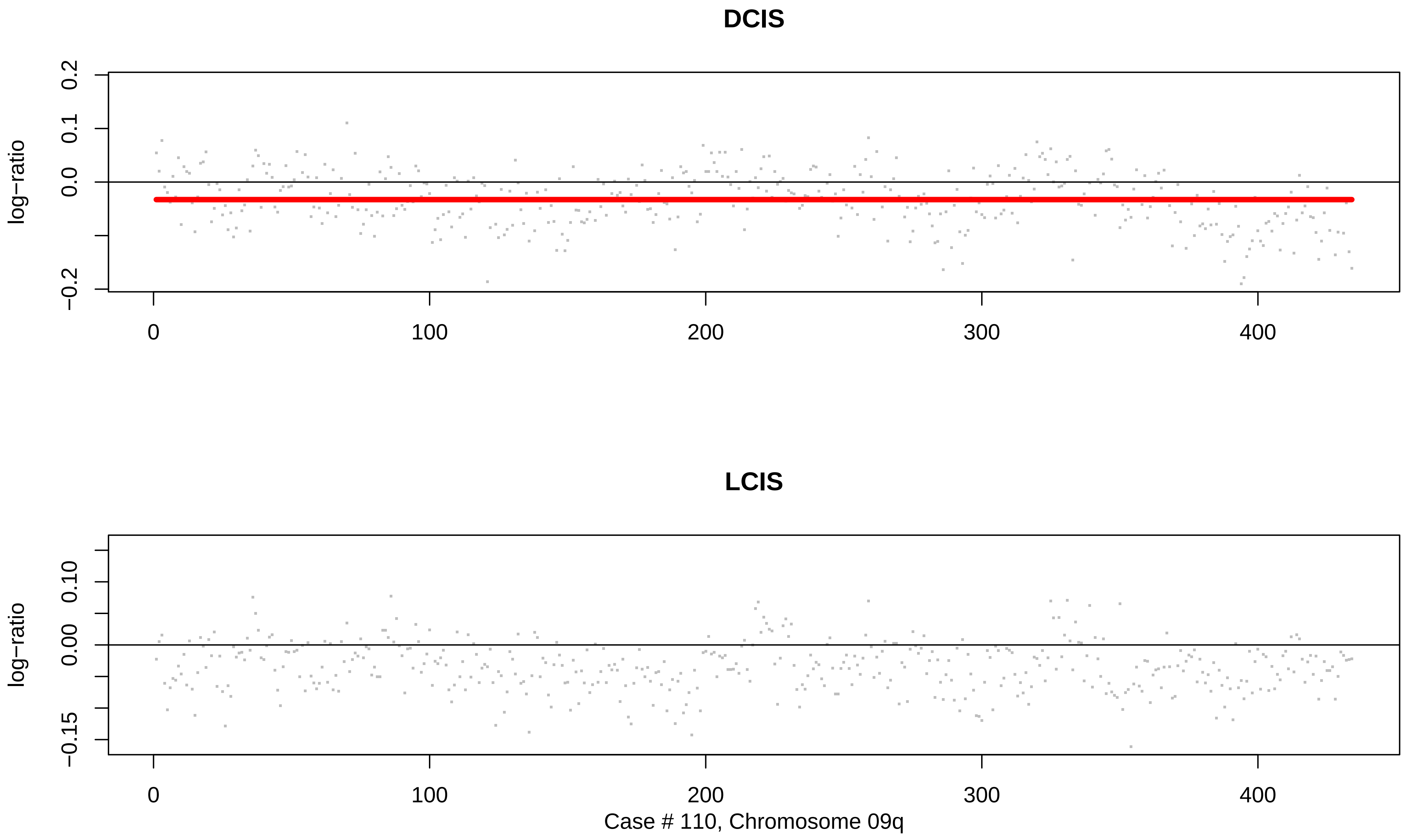
DCIS
log−ratio
0.2
0.1
0.0
−0.2
0
100
200
300
400
LCIS
log−ratio
0.10
0.00
−0.15
0
100
200
300
400
Case # 110, Chromosome 09q

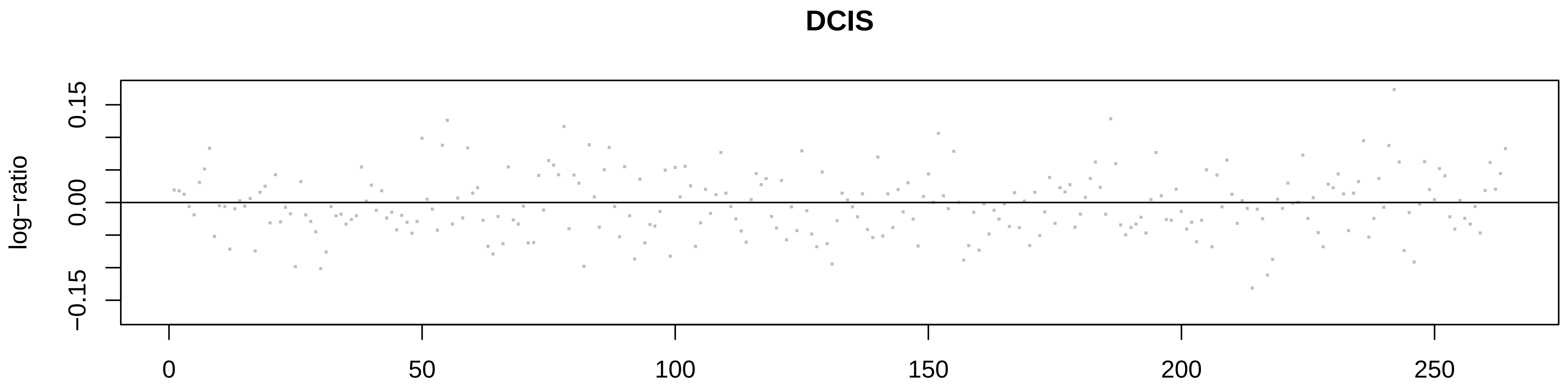
DCIS
log−ratio
0.15
0.00
−0.15
0
50
100
150
200
250

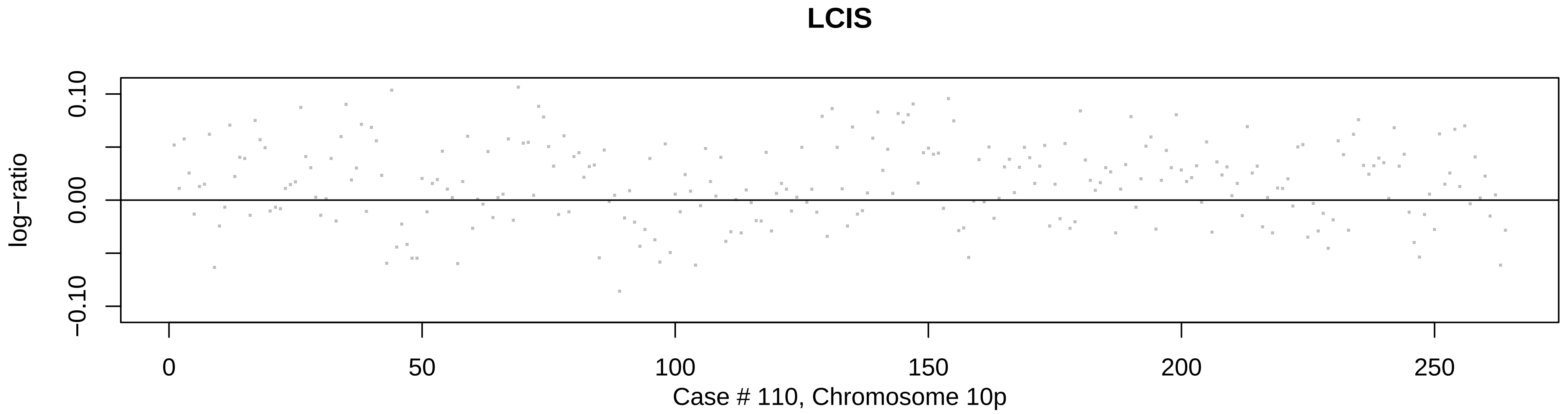
LCIS
log−ratio
0.10
0.00
−0.10
0
50
100
150
200
250
Case # 110, Chromosome 10p

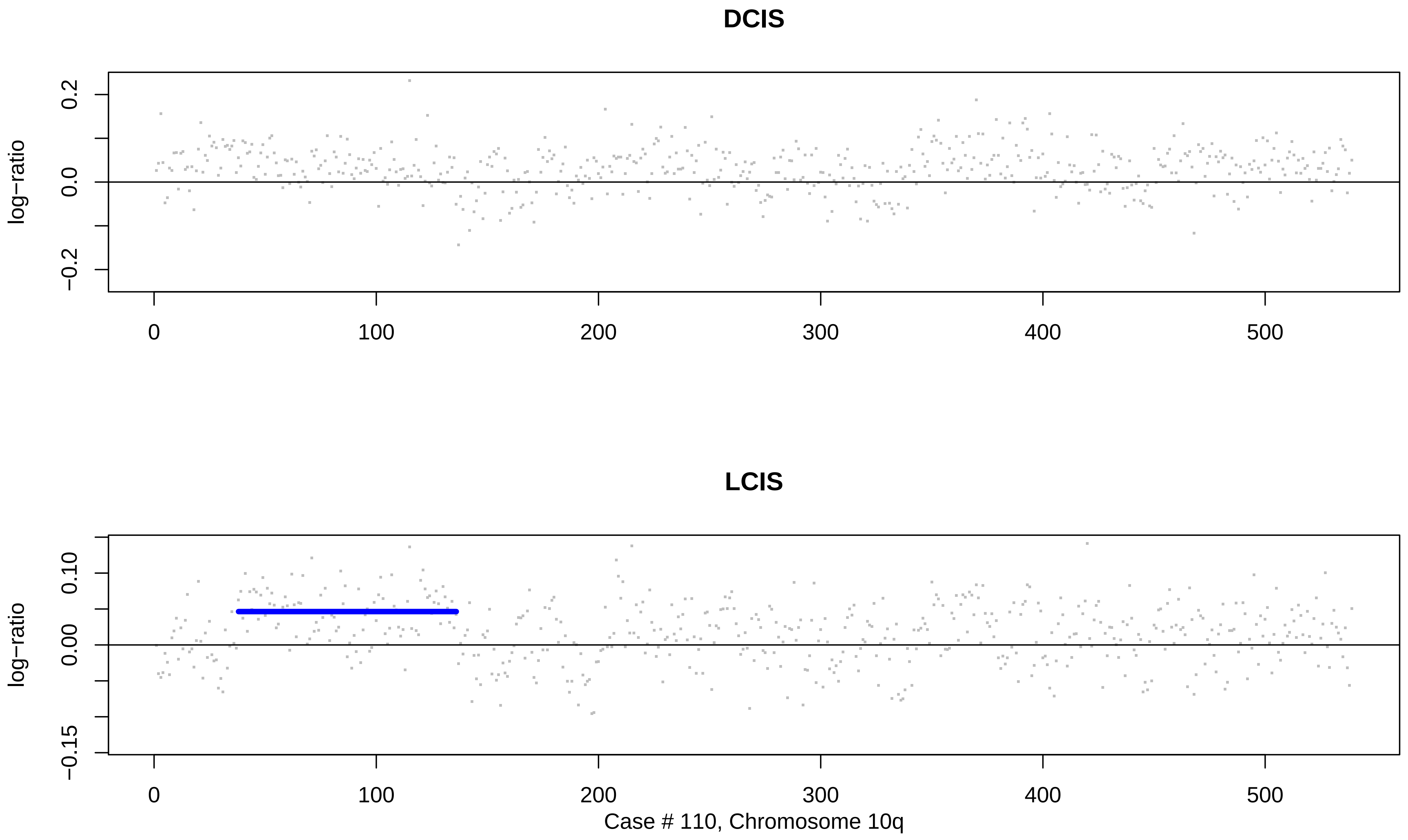
DCIS
log−ratio
0.2
0.0
−0.2
0
100
200
300
400
500
LCIS
log−ratio
0.10
0.00
−0.15
0
100
200
300
400
500
Case # 110, Chromosome 10q

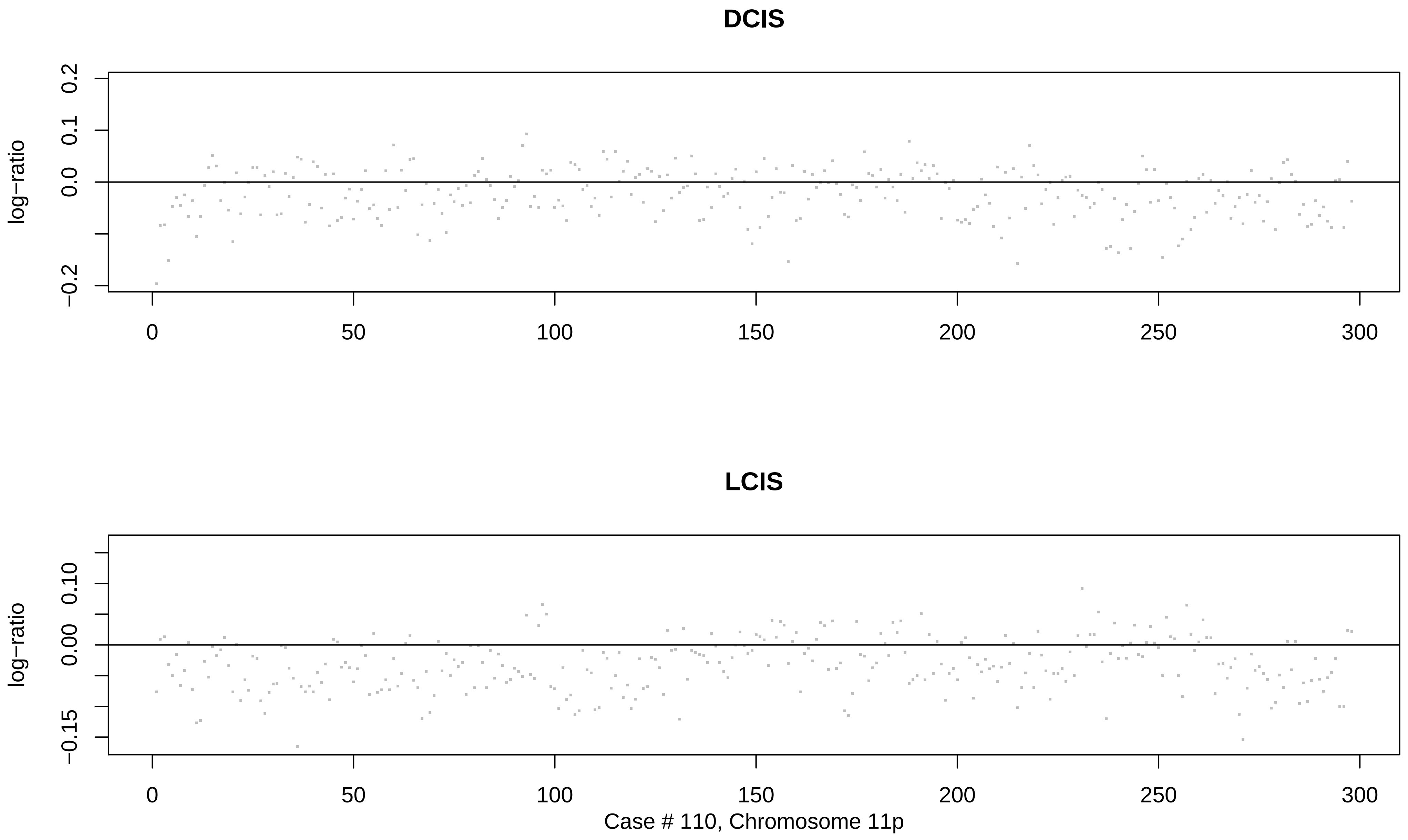
DCIS
log−ratio
0.2
0.1
0.0
−0.2
0
50
100
150
200
250
300
LCIS
log−ratio
0.10
0.00
−0.15
0
50
100
150
200
250
300
Case # 110, Chromosome 11p

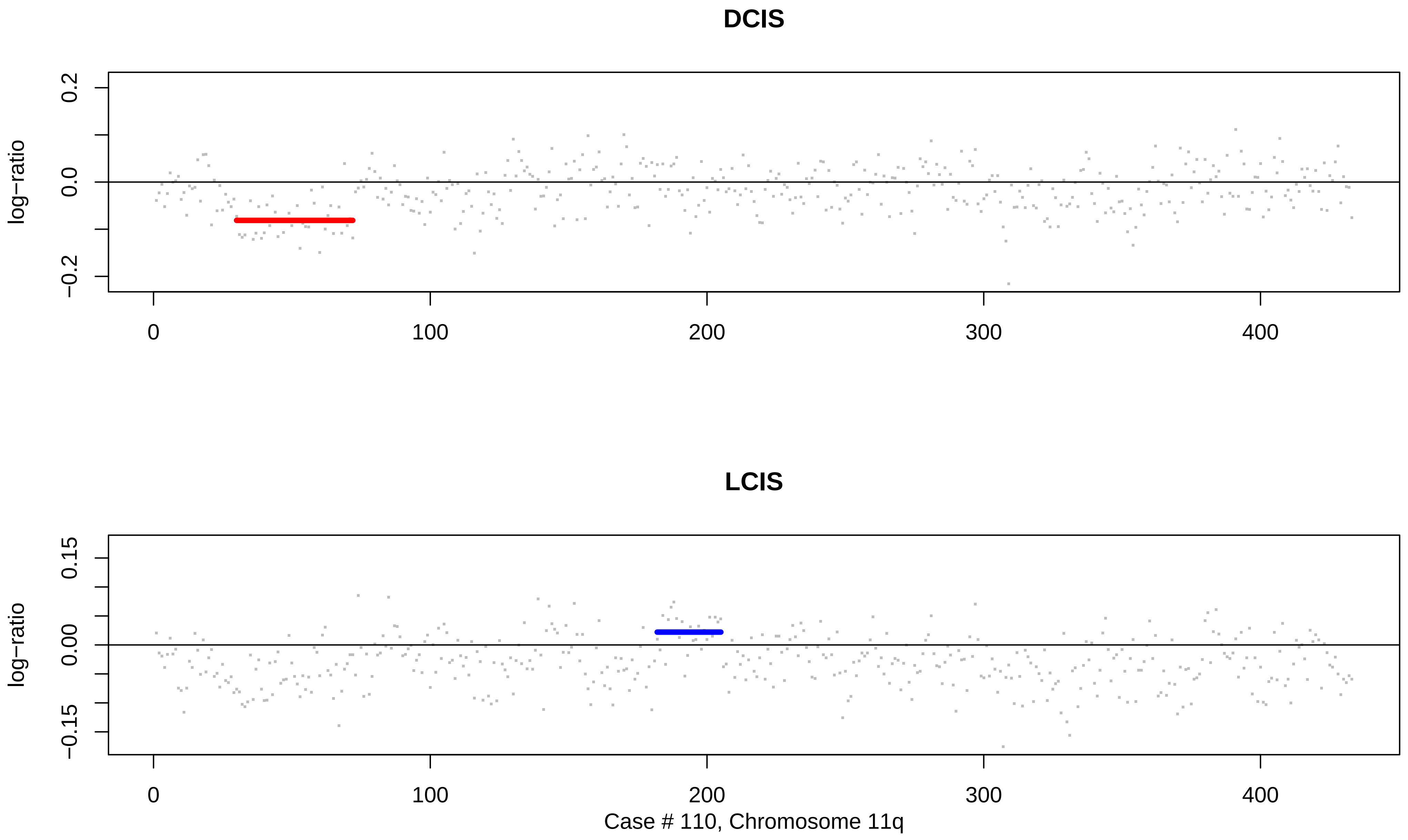

DCIS
log−ratio
0.2
0.0
−0.2
0
100
200
300
400
LCIS
log−ratio
0.15
0.00
−0.15
0
100
200
300
400
Case # 110, Chromosome 11q

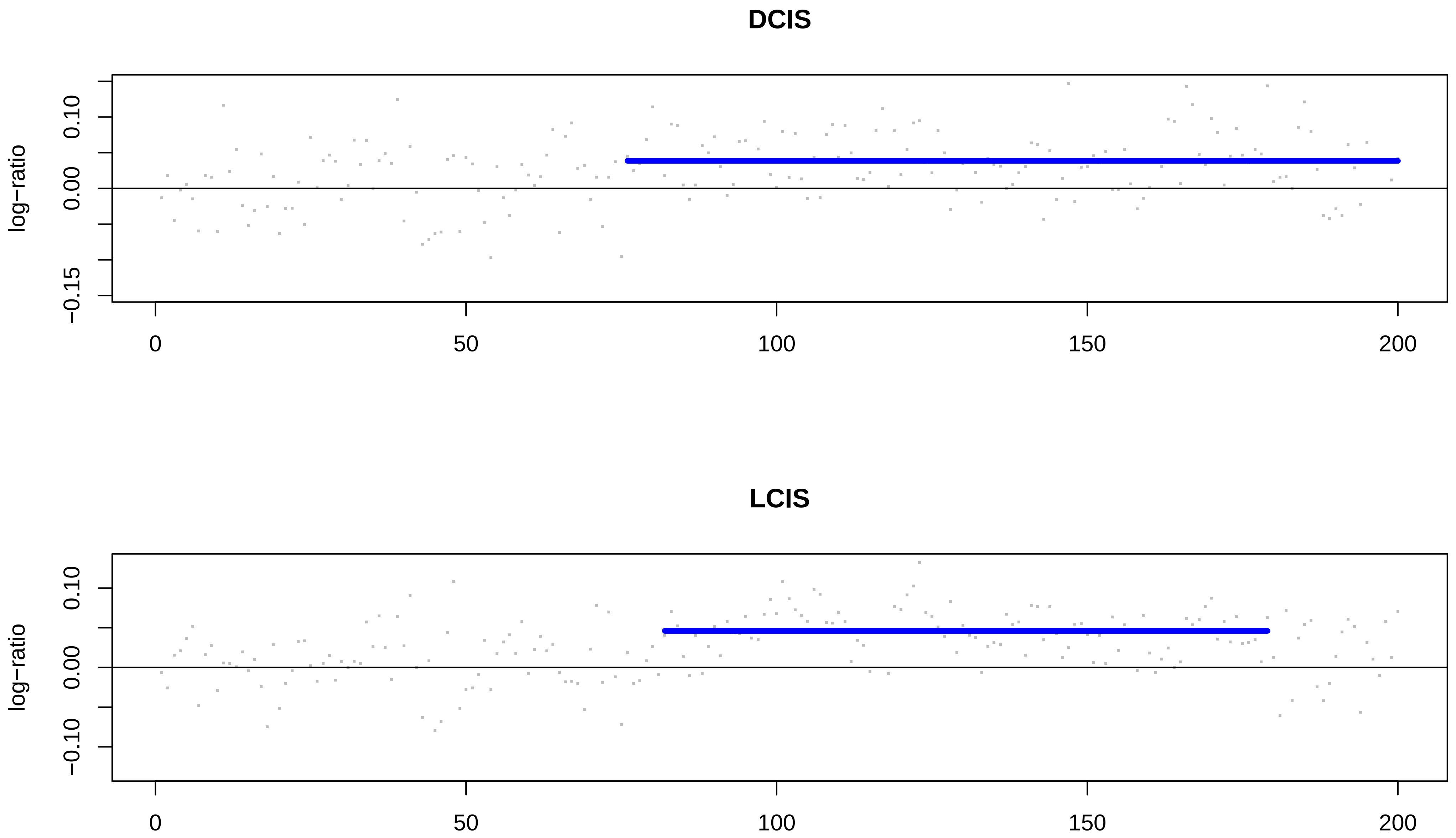

Case # 110, Chromosome 12p
Odds in favor of independence = 1.7

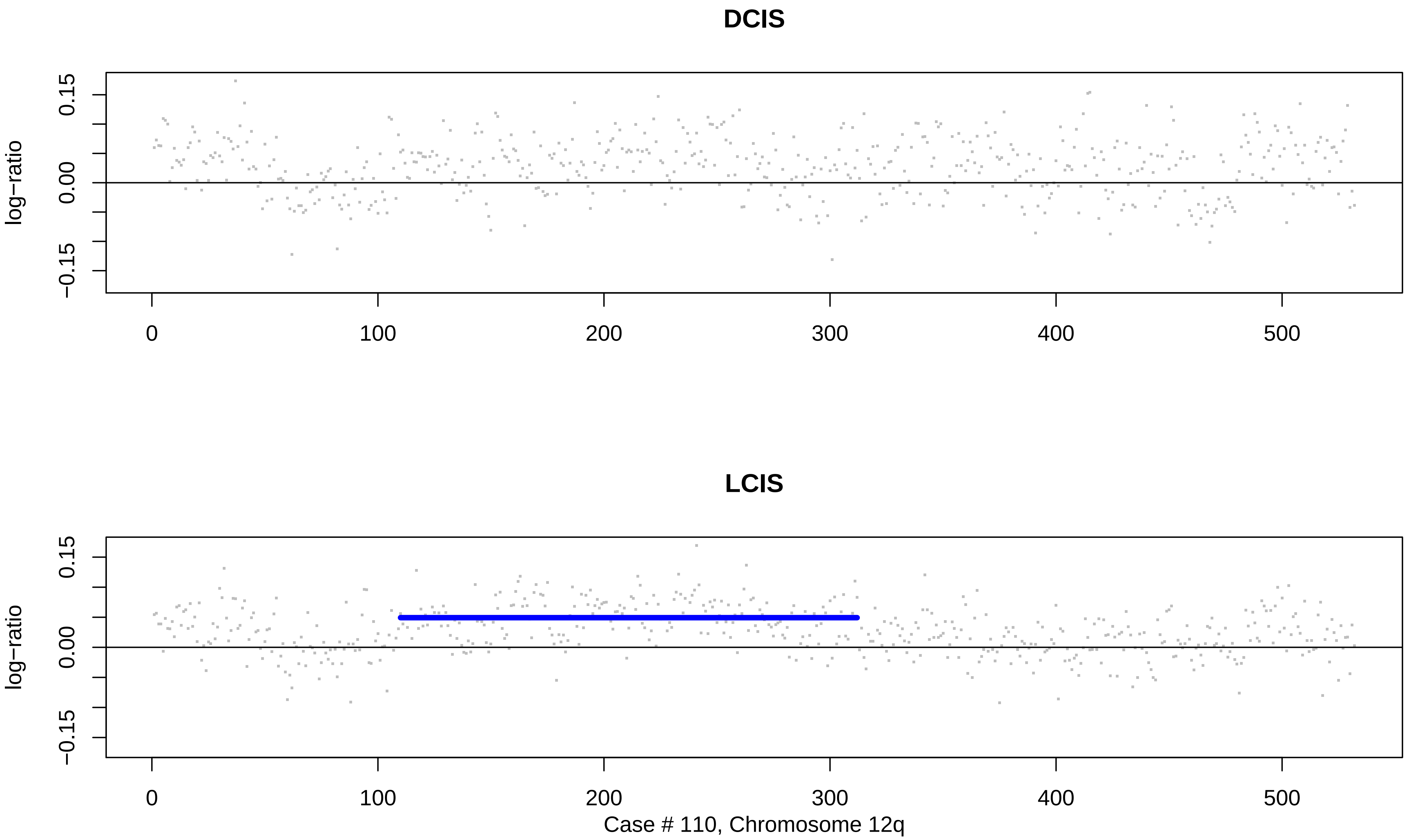
DCIS
log−ratio
0.15
0.00
−0.15
0
100
200
300
400
500
LCIS
log−ratio
0.15
0.00
−0.15
0
100
200
300
400
500
Case # 110, Chromosome 12q

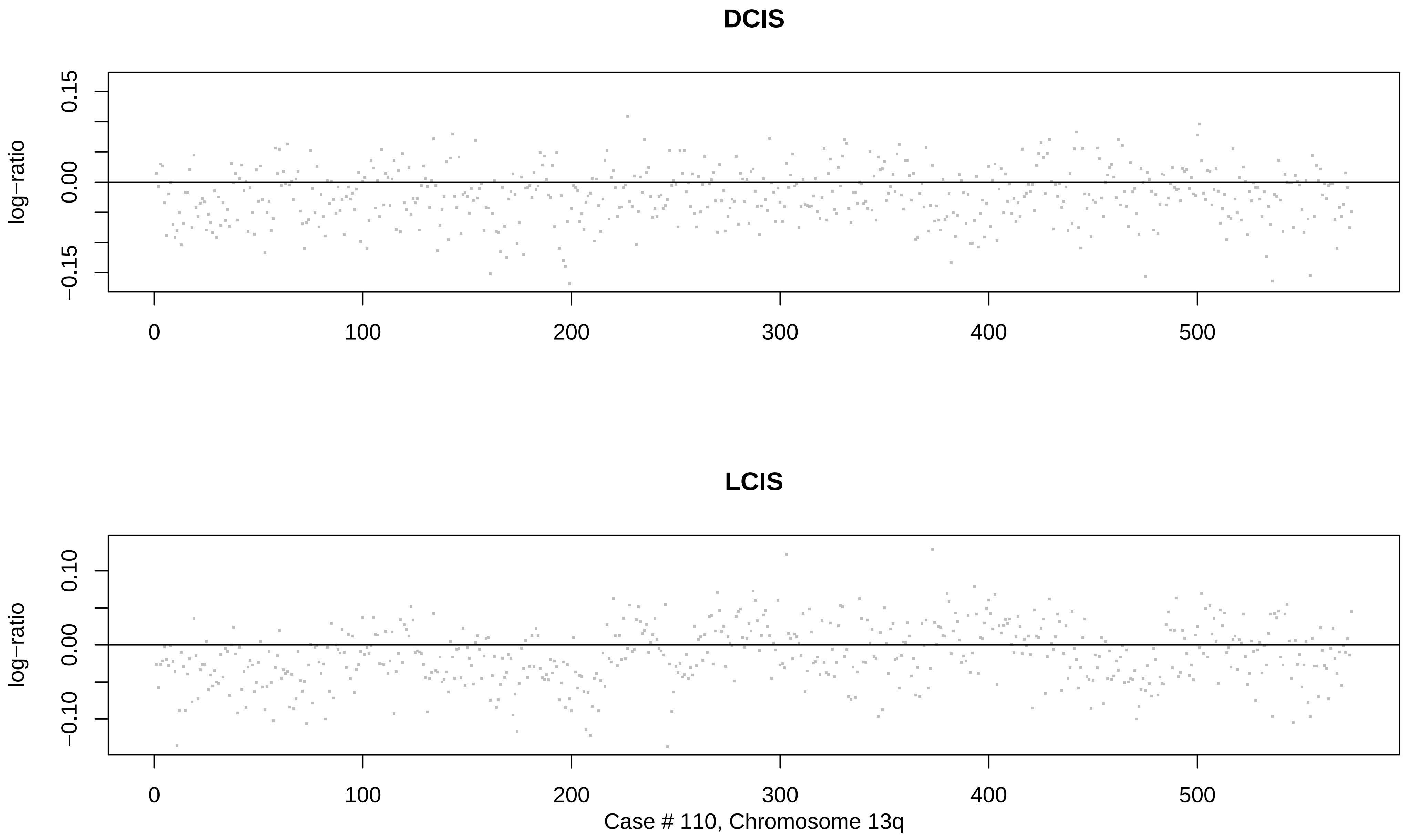

DCIS
log−ratio
0.15
0.00
−0.15
0
100
200
300
400
500
LCIS
log−ratio
0.10
0.00
−0.10
0
100
200
300
400
500
Case # 110, Chromosome 13q

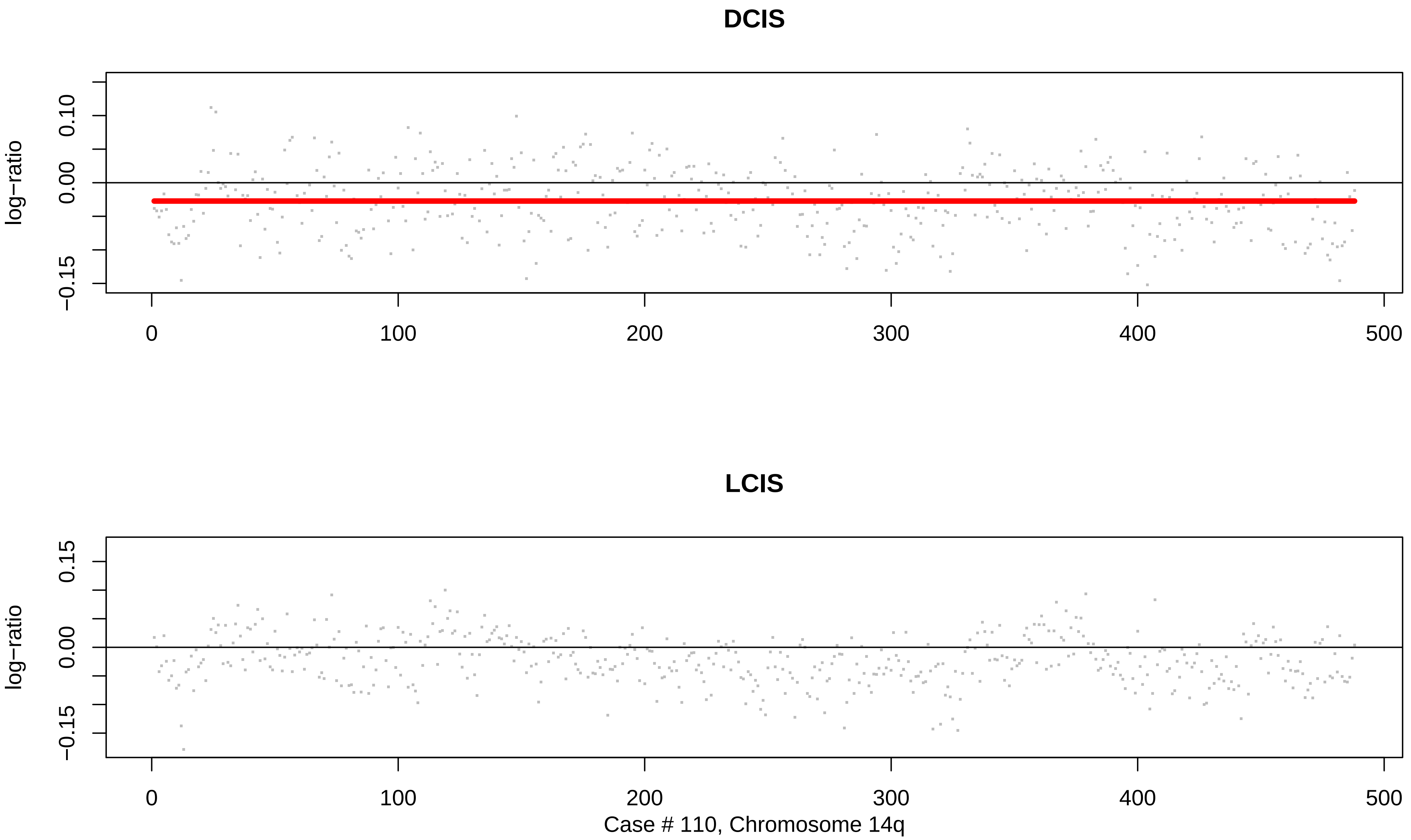
DCIS
log−ratio
−0.15
0.00
0.10
0
100
200
300
400
500
LCIS
log−ratio
−0.15
0.00
0.15
0
100
200
300
400
500
Case # 110, Chromosome 14q

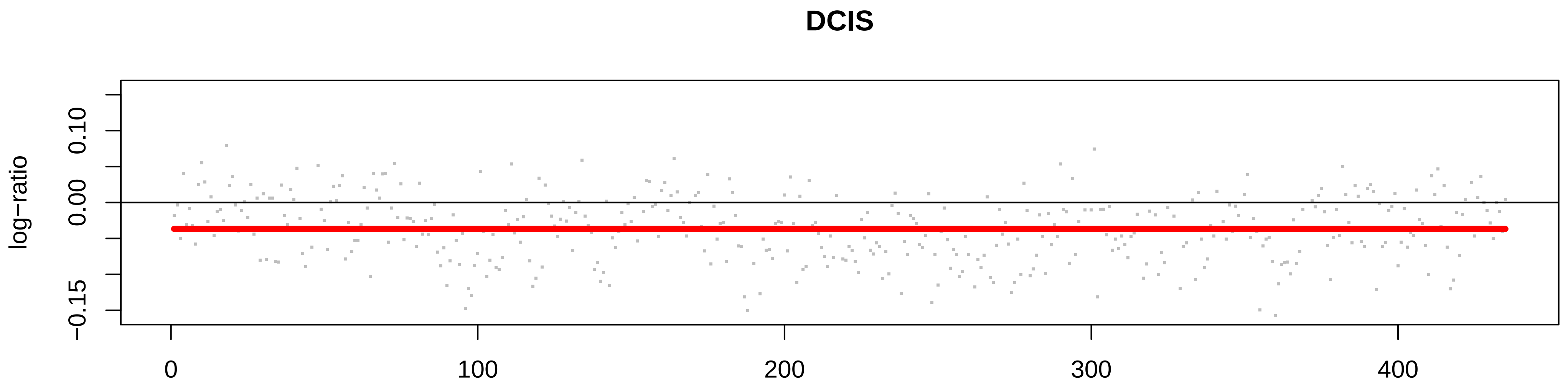
DCIS
log−ratio
0.10
0.00
−0.15
0
100
200
300
400

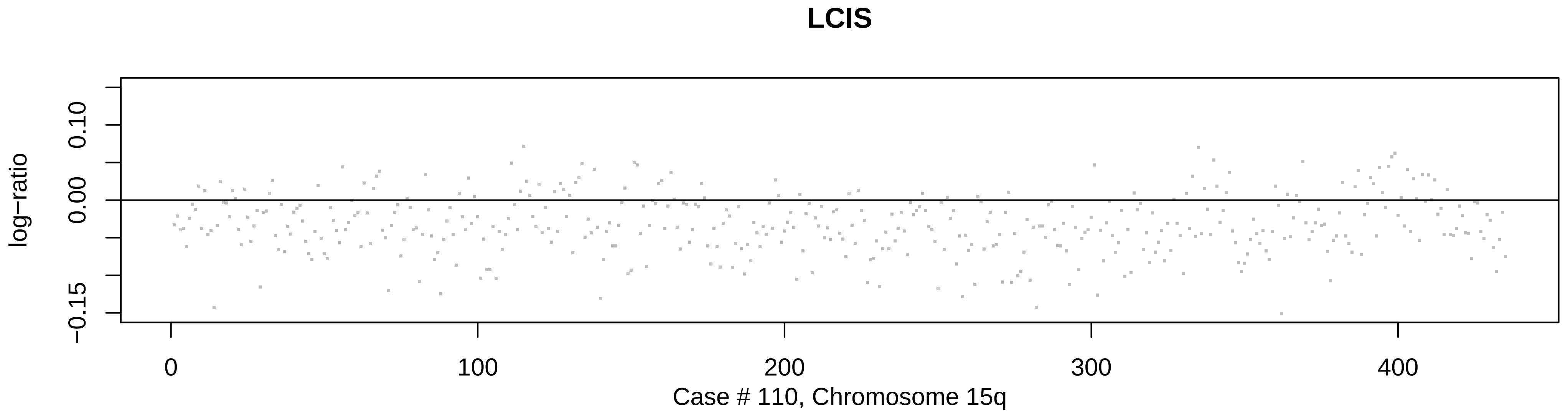
LCIS
log−ratio
0.10
0.00
−0.15
0
100
200
300
400
Case # 110, Chromosome 15q

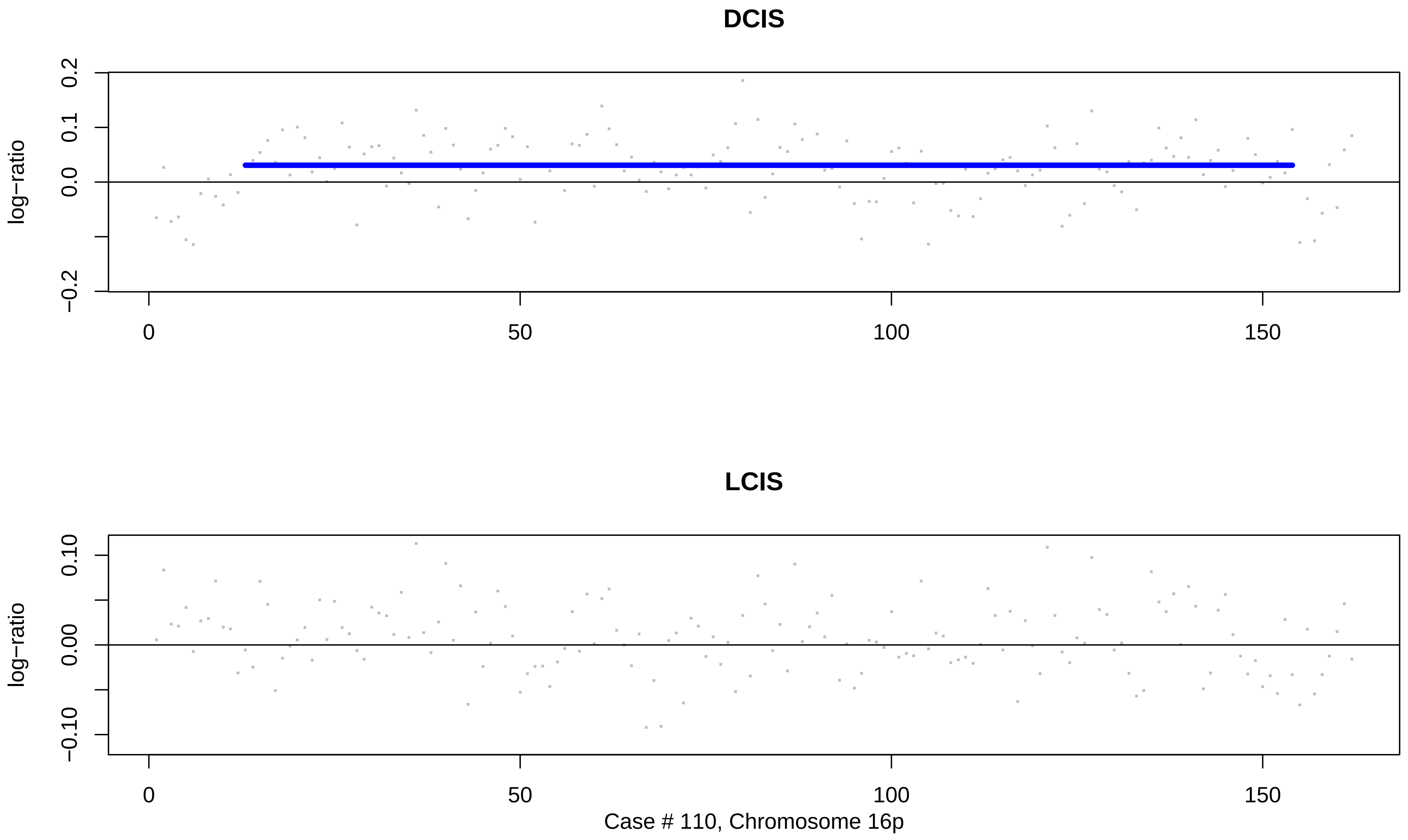
DCIS
log−ratio
0.2
0.1
0.0
−0.2
0
50
100
150
LCIS
log−ratio
0.10
0.00
−0.10
0
50
100
150
Case # 110, Chromosome 16p

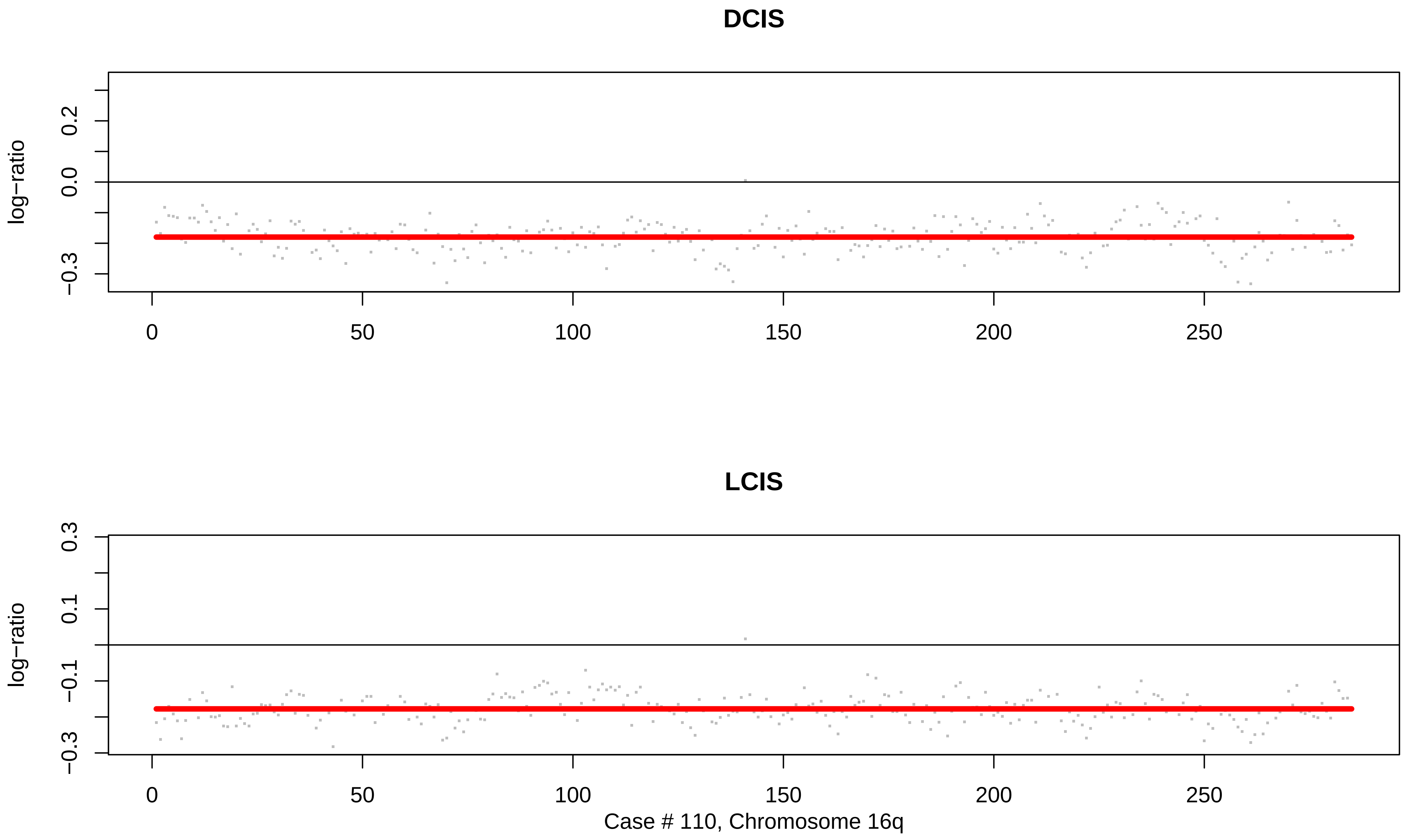
DCIS
log−ratio
0.2
0.0
−0.3
0
50
100
150
200
250
LCIS
log−ratio
0.3
0.1
−0.1
−0.3
0
50
100
150
200
250
Case # 110, Chromosome 16q

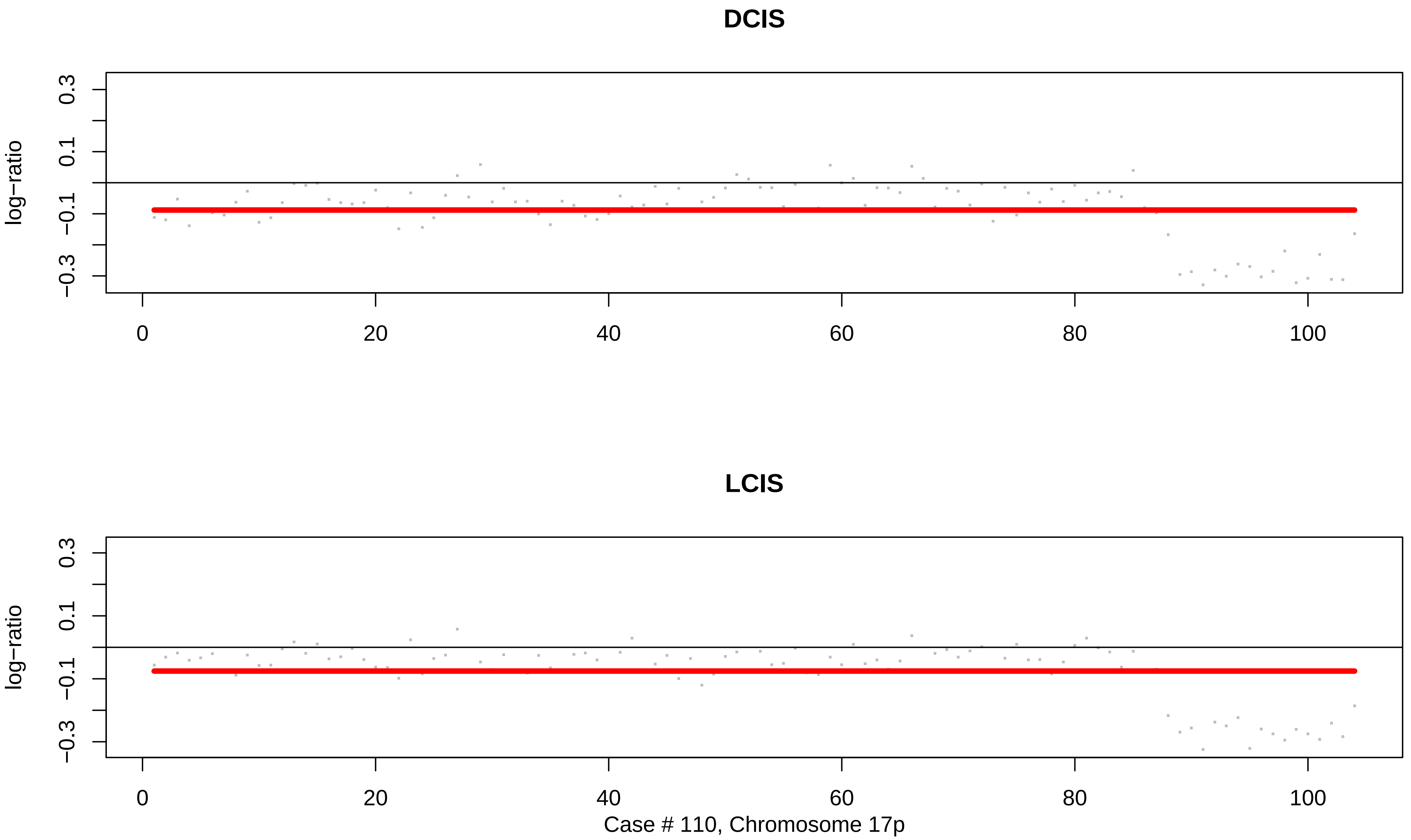
DCIS
log−ratio
0.3
0.1
−0.1
−0.3
0
20
40
60
80
100
LCIS
log−ratio
0.3
0.1
−0.1
−0.3
0
20
40
60
80
100
Case # 110, Chromosome 17p

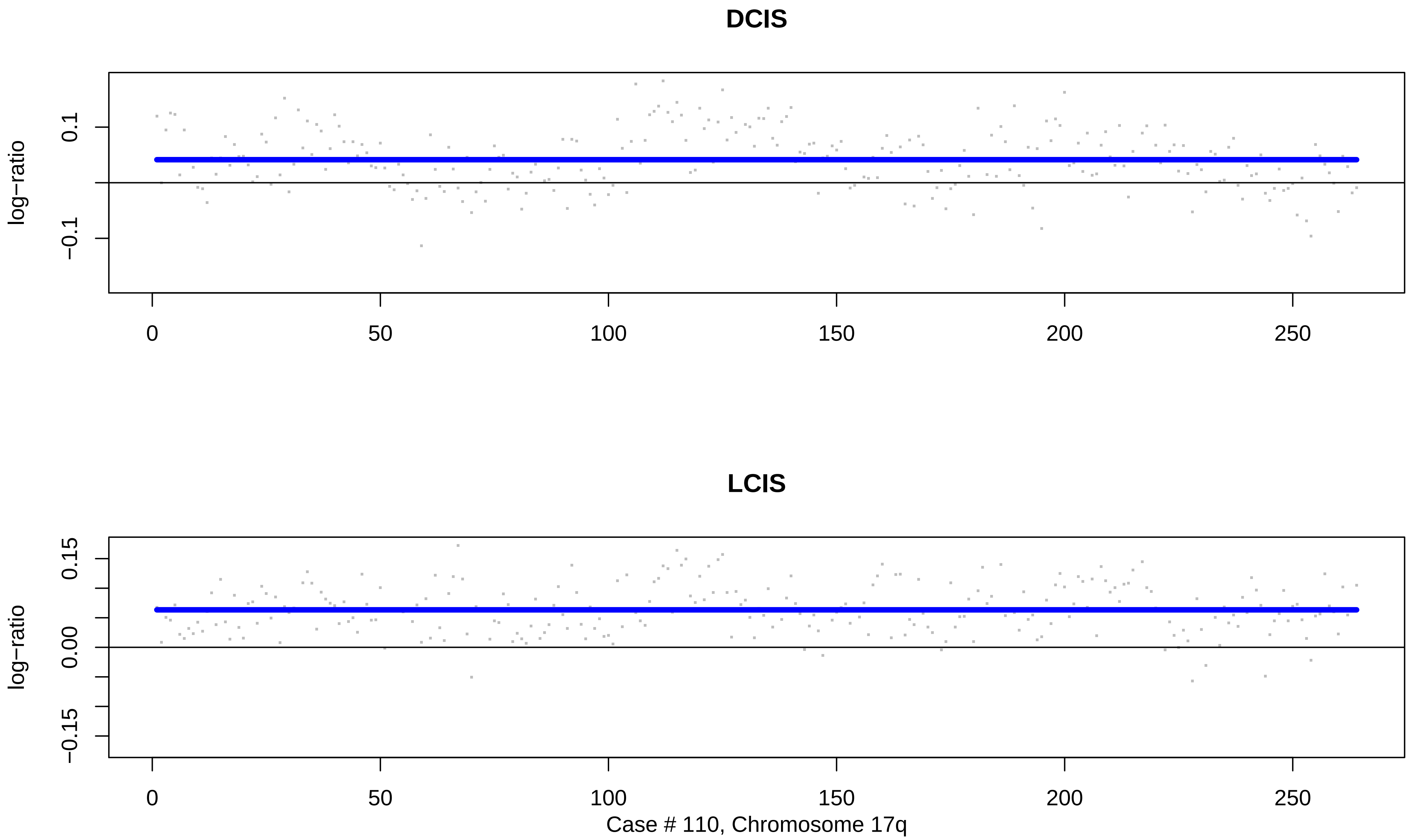
DCIS
log−ratio
0.1
−0.1
0
50
100
150
200
250
LCIS
log−ratio
0.15
0.00
−0.15
0
50
100
150
200
250
Case # 110, Chromosome 17q

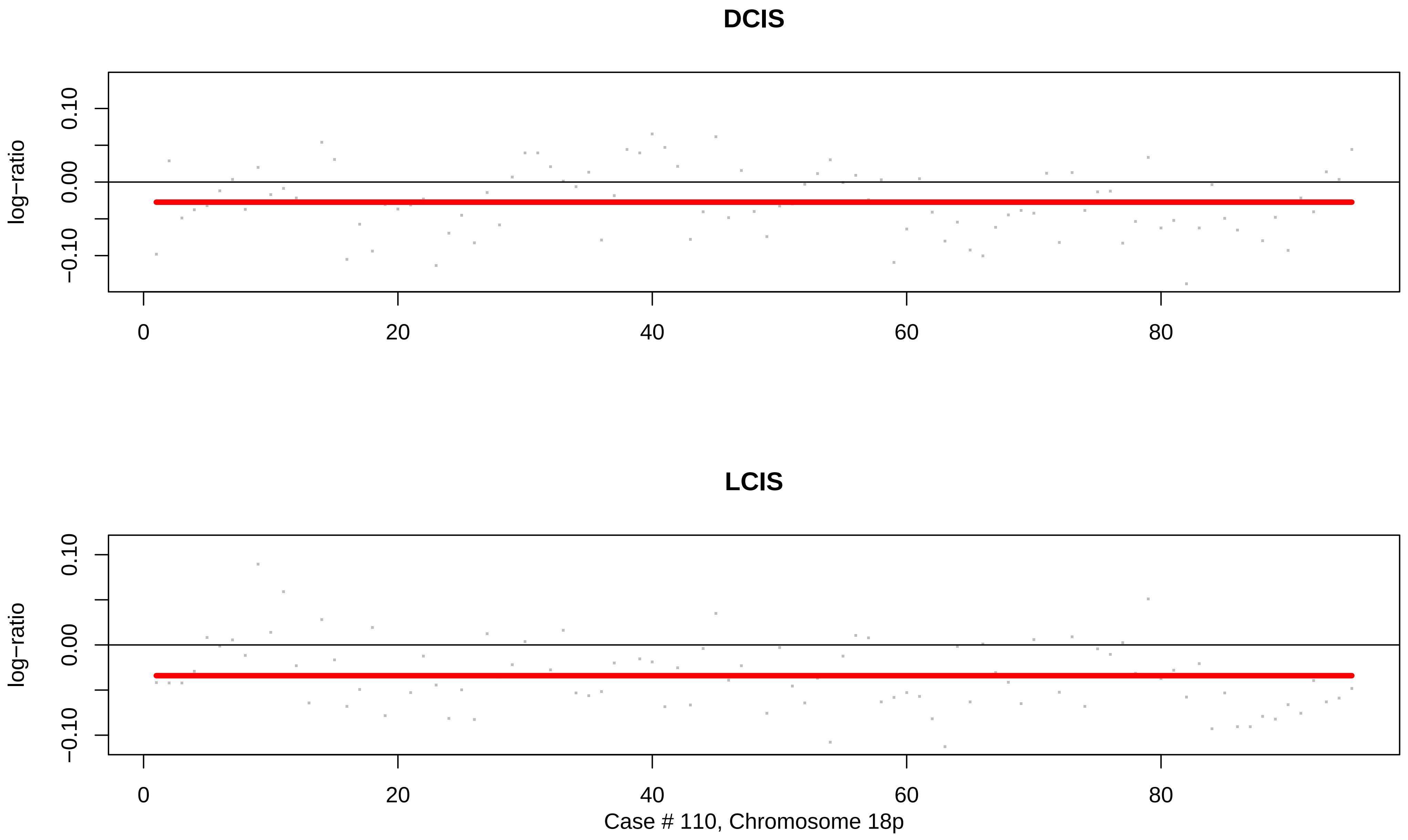
DCIS
log−ratio
0.10
0.00
−0.10
0
20
40
60
80
LCIS
log−ratio
0.10
0.00
−0.10
0
20
40
60
80
Case # 110, Chromosome 18p

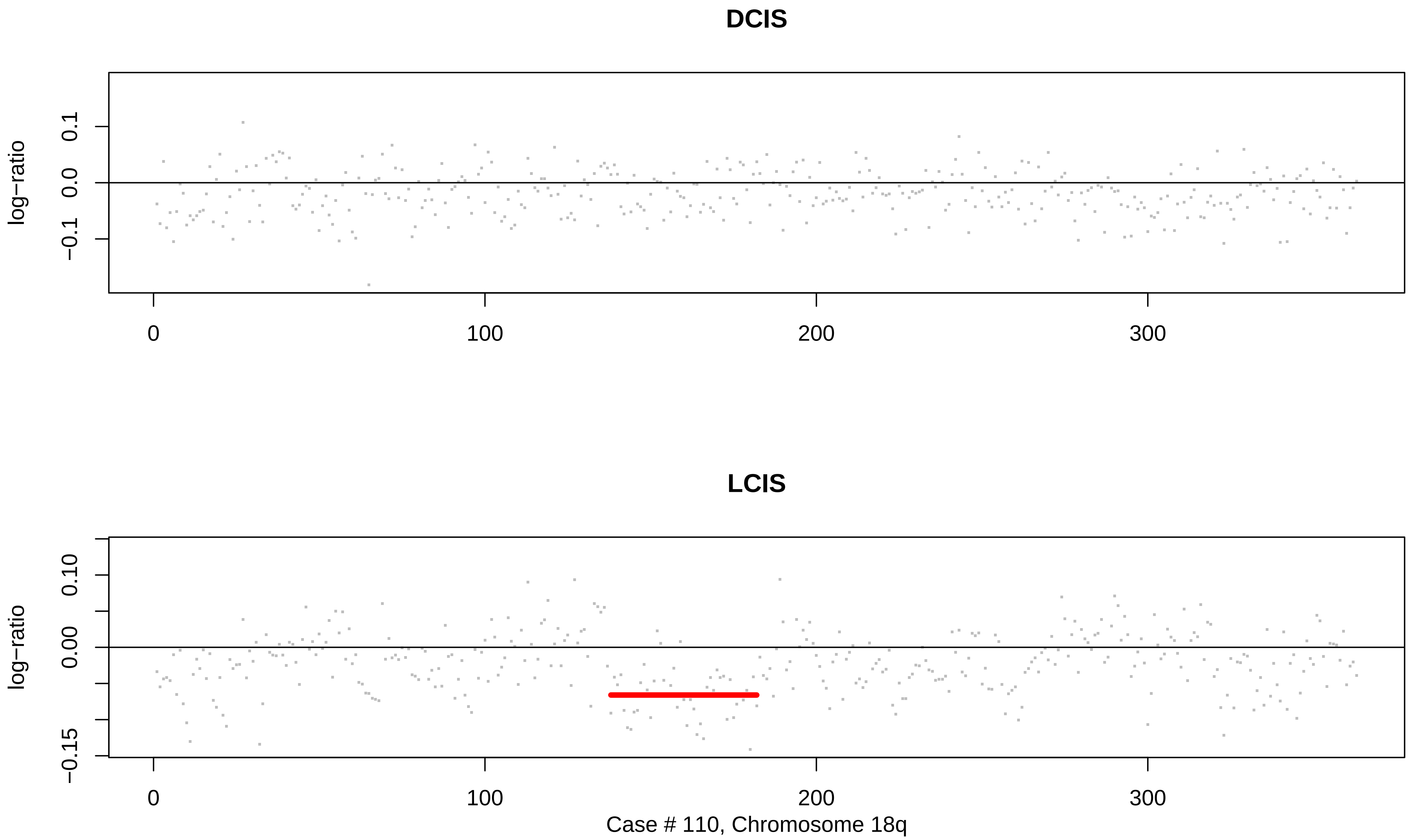
DCIS
log−ratio
0.1
0.0
−0.1
0
100
200
300
LCIS
log−ratio
0.10
0.00
−0.15
0
100
200
300
Case # 110, Chromosome 18q

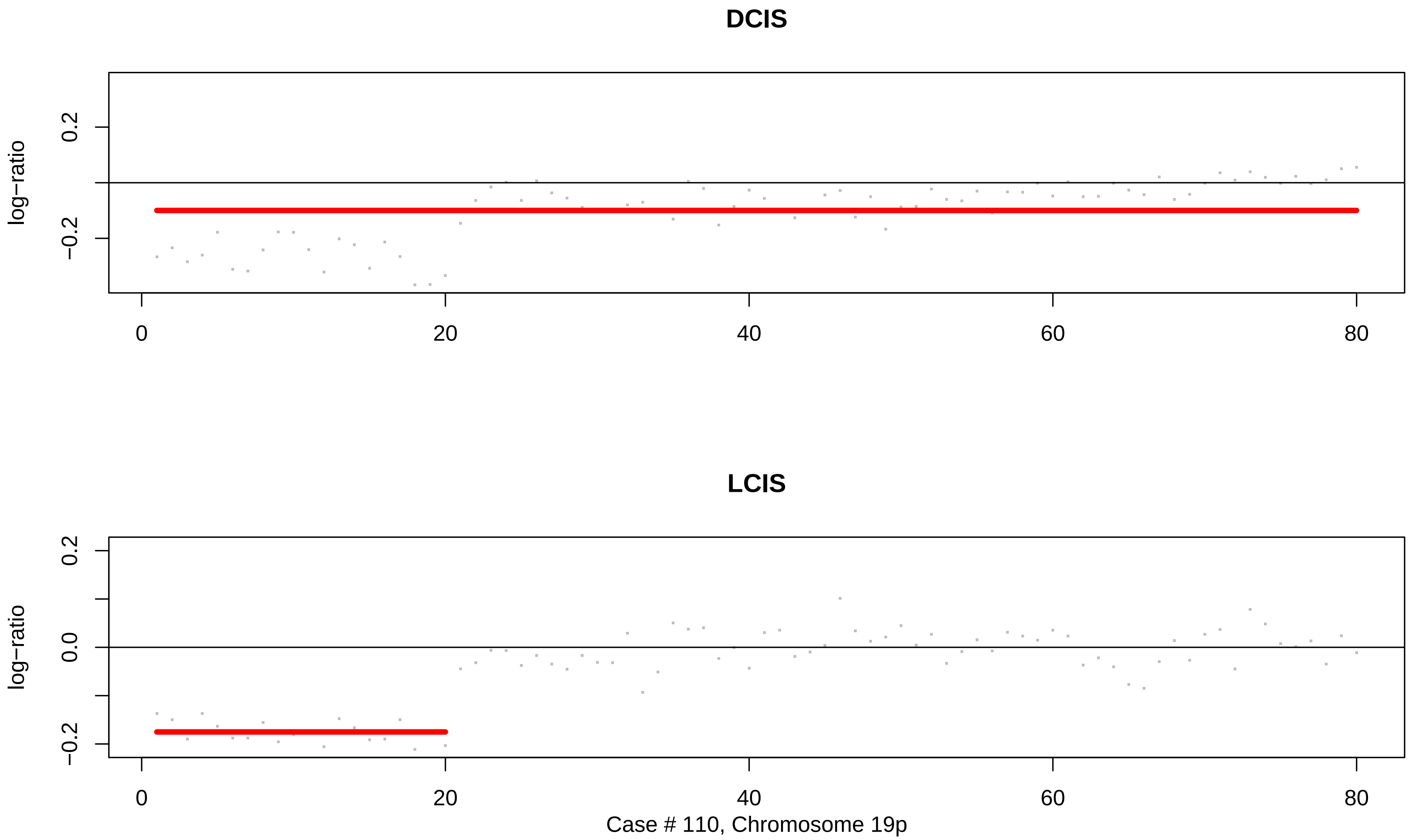
DCIS
log−ratio
0.2
−0.2
0
20
40
60
80
LCIS
log−ratio
0.2
0.0
−0.2
0
20
40
60
80
Case # 110, Chromosome 19p

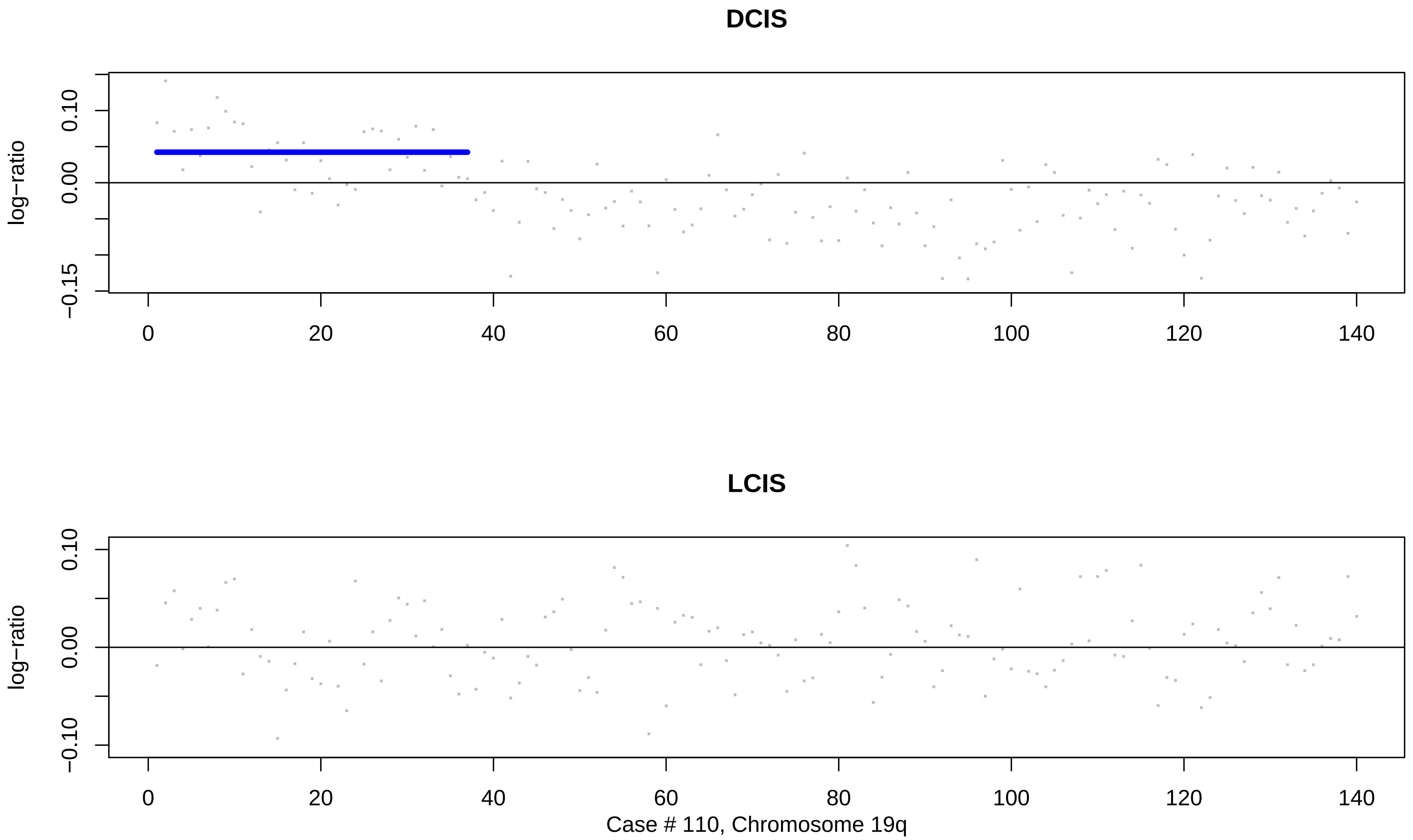
DCIS
log−ratio
0.10
0.00
−0.15
0
20
40
60
80
100
120
140
LCIS
log−ratio
0.10
0.00
−0.10
0
20
40
60
80
100
120
140
Case # 110, Chromosome 19q

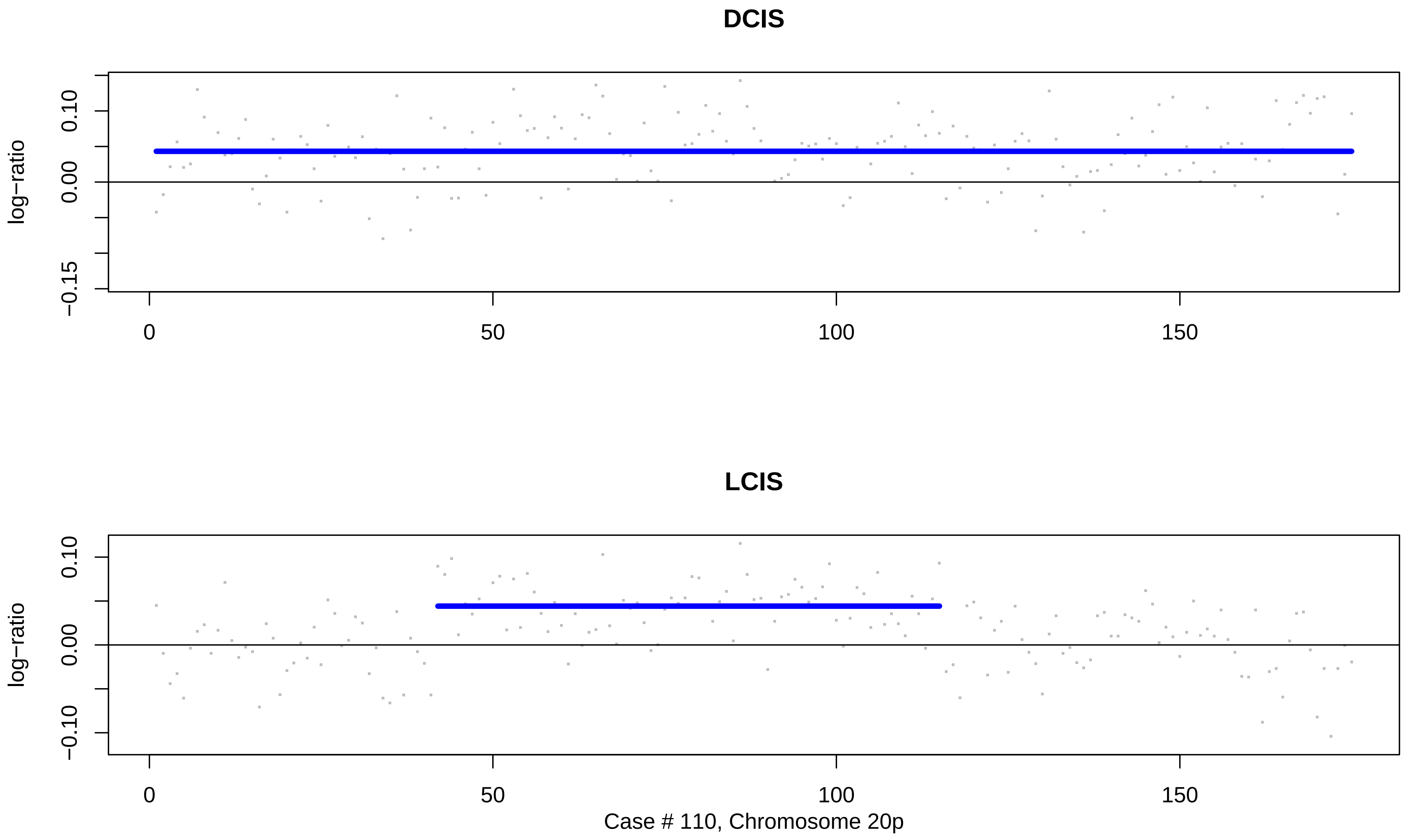

DCIS
log−ratio
−0.15
0.00
0.10
0
50
100
150
LCIS
log−ratio
−0.10
0.00
0.10
0
50
100
150
Case # 110, Chromosome 20p

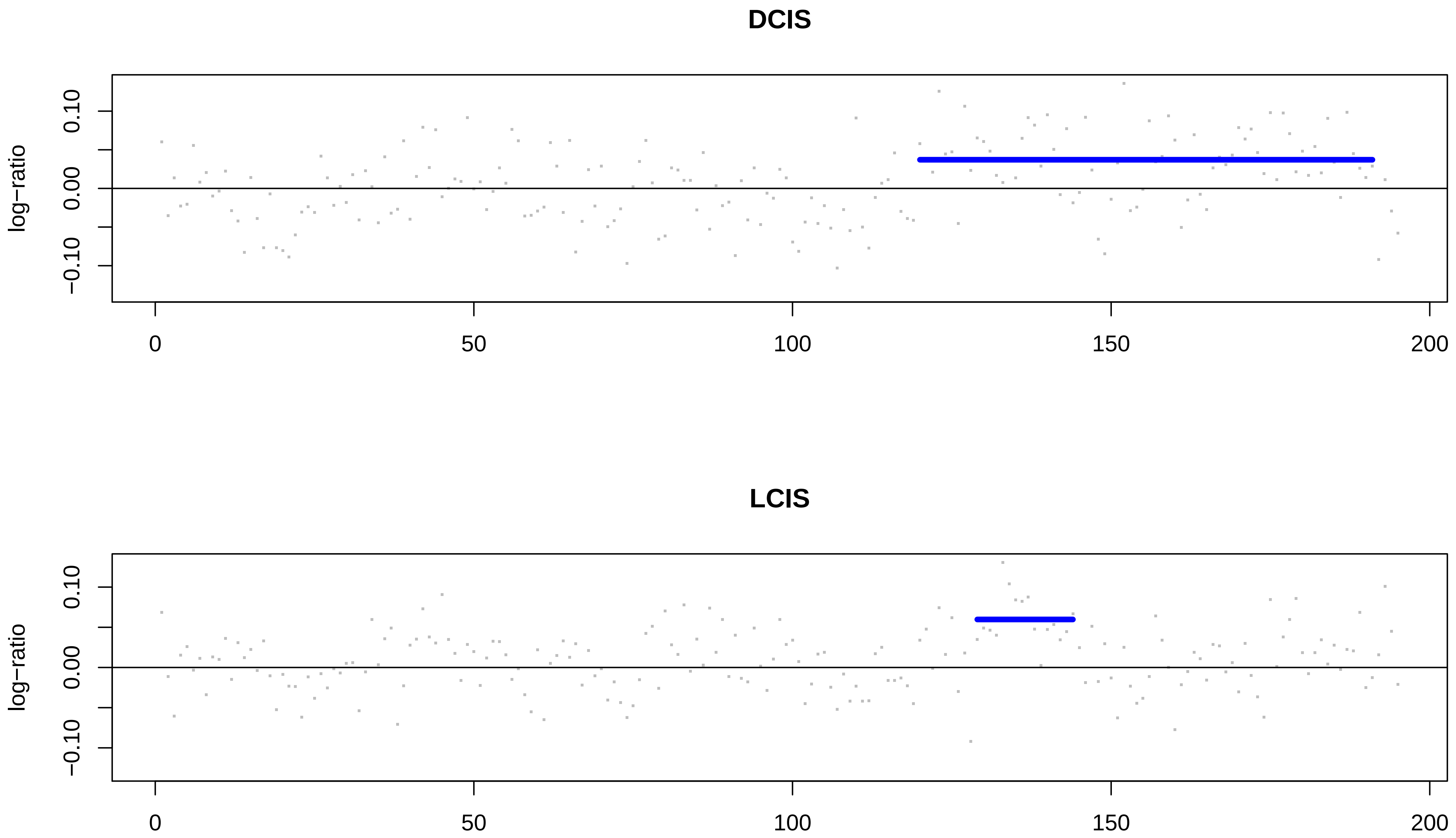

Case # 110, Chromosome 20q
Odds in favor of independence = 3.6

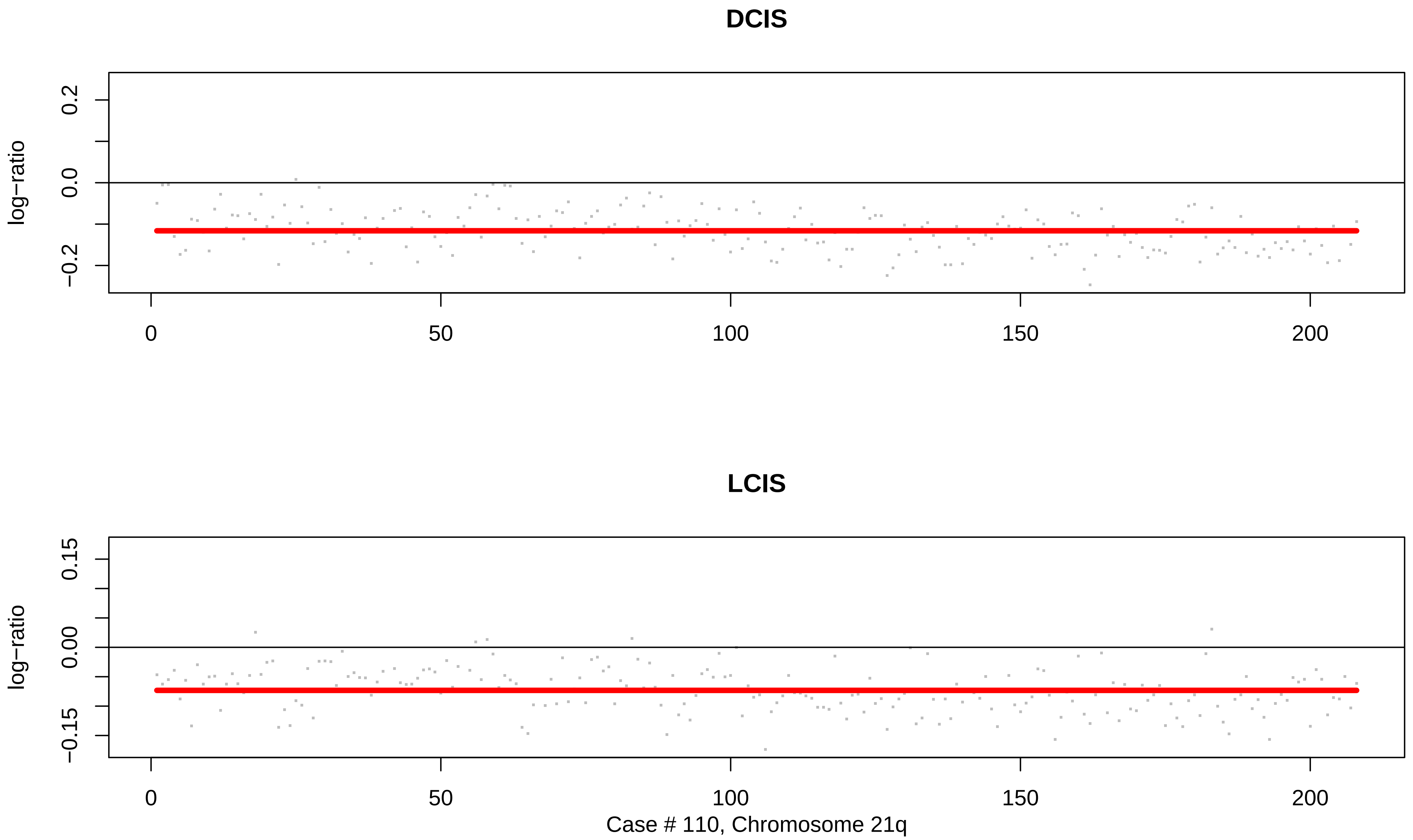
DCIS
log−ratio
0.2
0.0
−0.2
0
50
100
150
200
LCIS
log−ratio
0.15
0.00
−0.15
0
50
100
150
200
Case # 110, Chromosome 21q

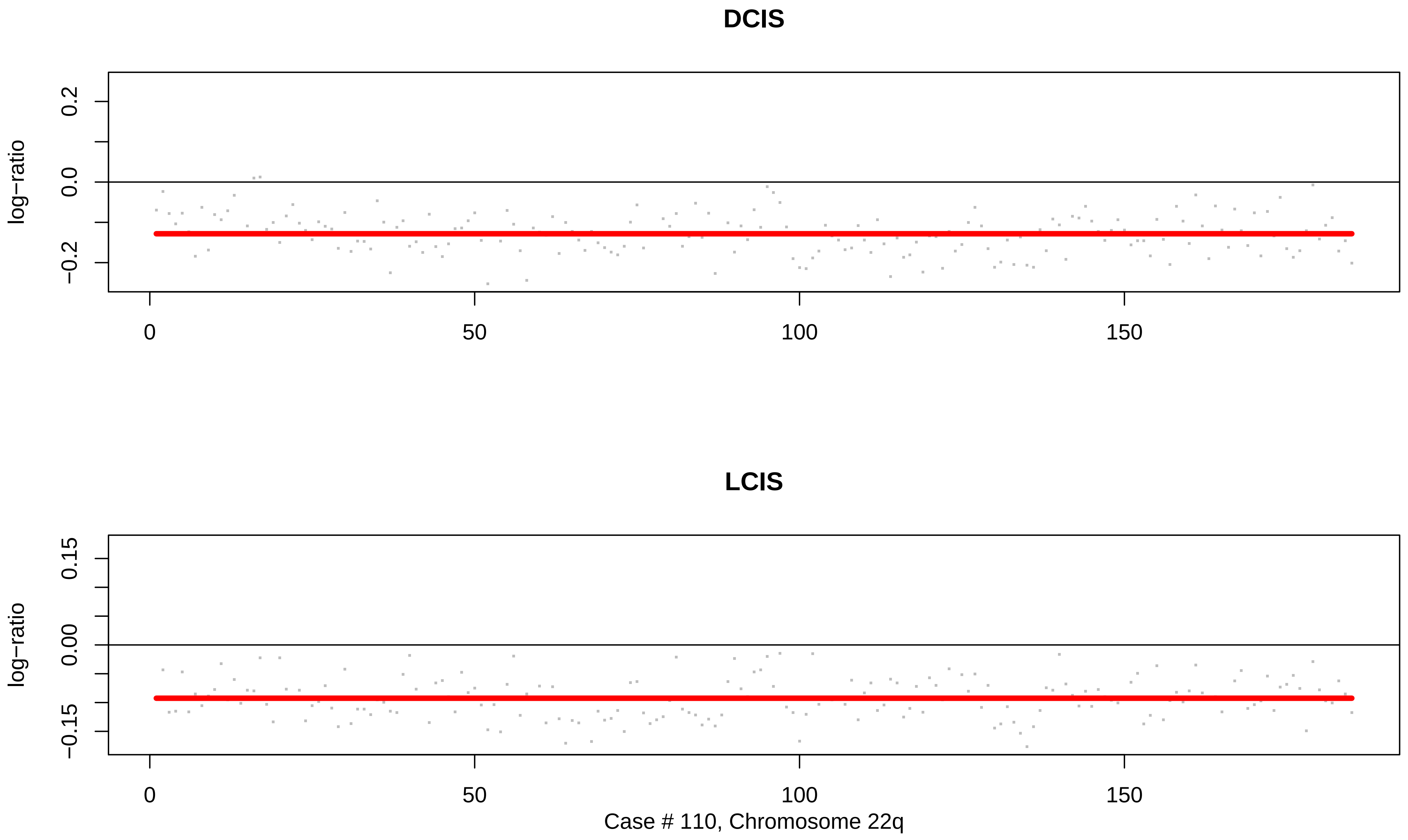
DCIS
log−ratio
0.2
0.0
−0.2
0
50
100
150
LCIS
log−ratio
0.15
0.00
−0.15
0
50
100
150
Case # 110, Chromosome 22q
