# Supplementary material for: Clonal relatedness between lobular carcinoma in situ and synchronous malignant lesions
Source: Breast Cancer Res. 2012 Jul 9;14(4):R103. doi: 10.1186/bcr3222 (PMC3680923; doi:10.1186/bcr3222)
Supplement: Additional file 4 — Magnified version of genome-wide plots with detailed marker plots and segmentation on a chromosome-arm-specific basis. [file bcr3222-S4.ZIP › Case 095 ID.pdf]

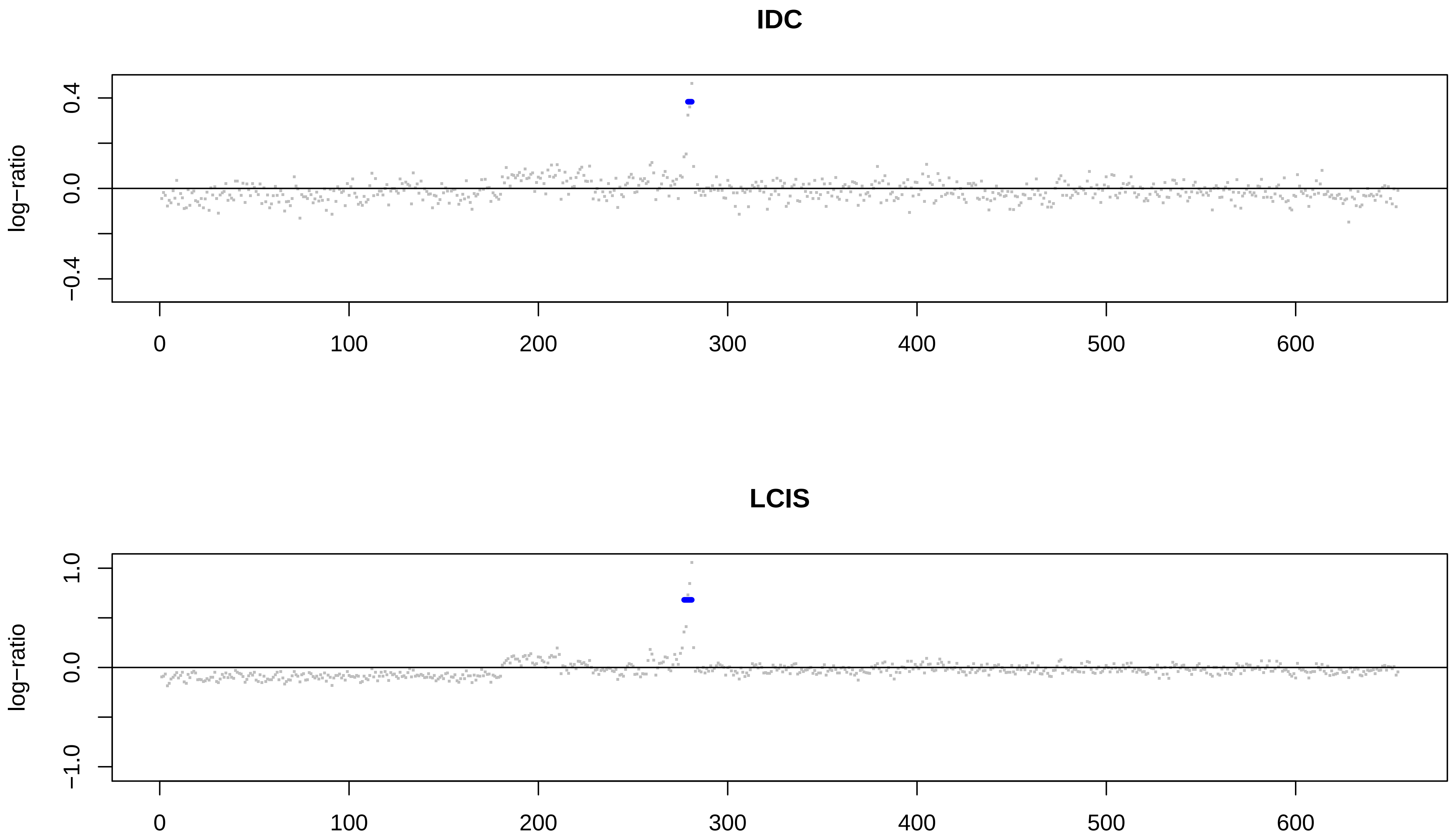

Case # 095, Chromosome 01p

Odds in favor of independence = 3.3

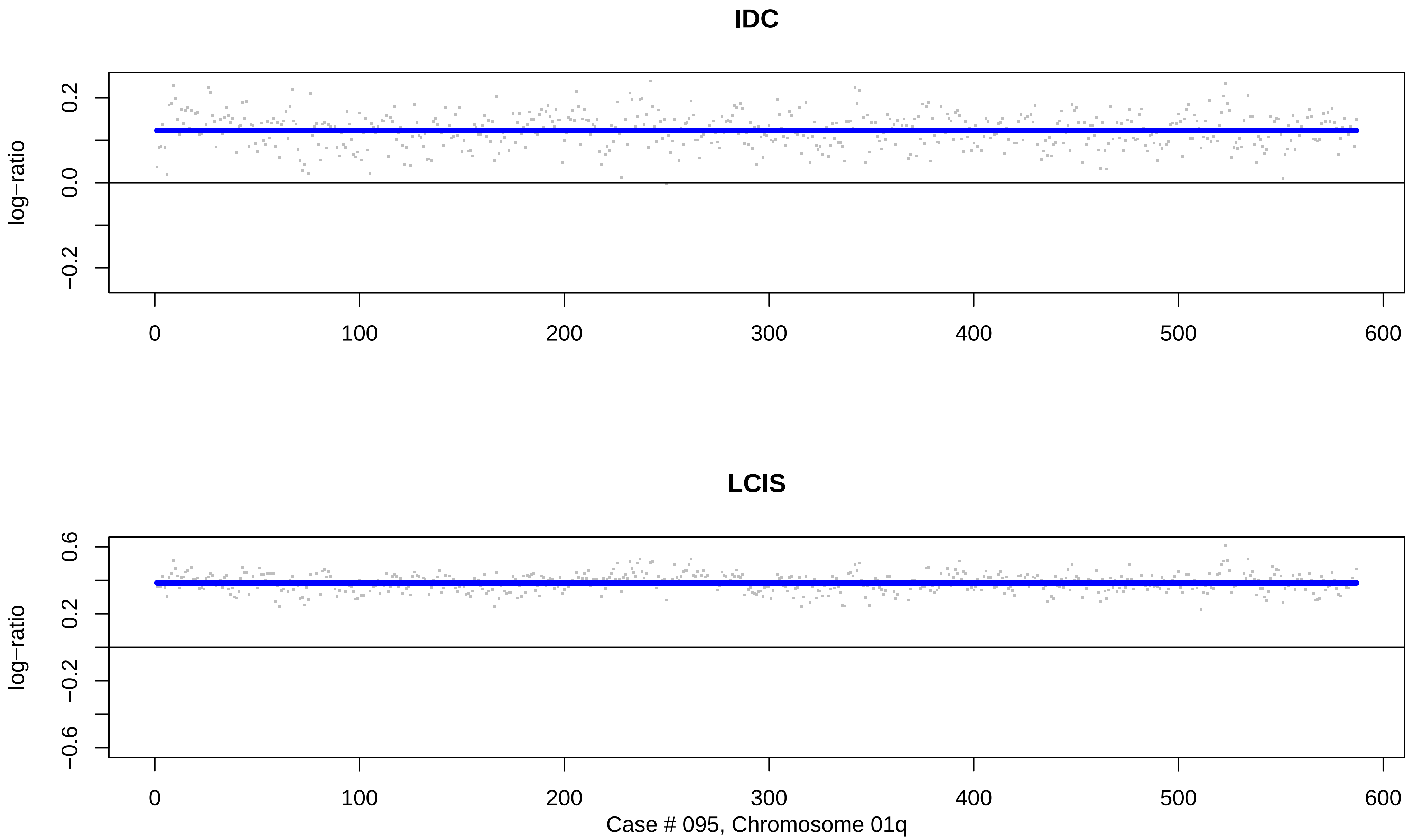
IDC
log−ratio
0.2
0.0
−0.2
0
100
200
300
400
500
600
LCIS
log−ratio
0.6
0.2
−0.2
−0.6
0
100
200
300
400
500
600
Case # 095, Chromosome 01q

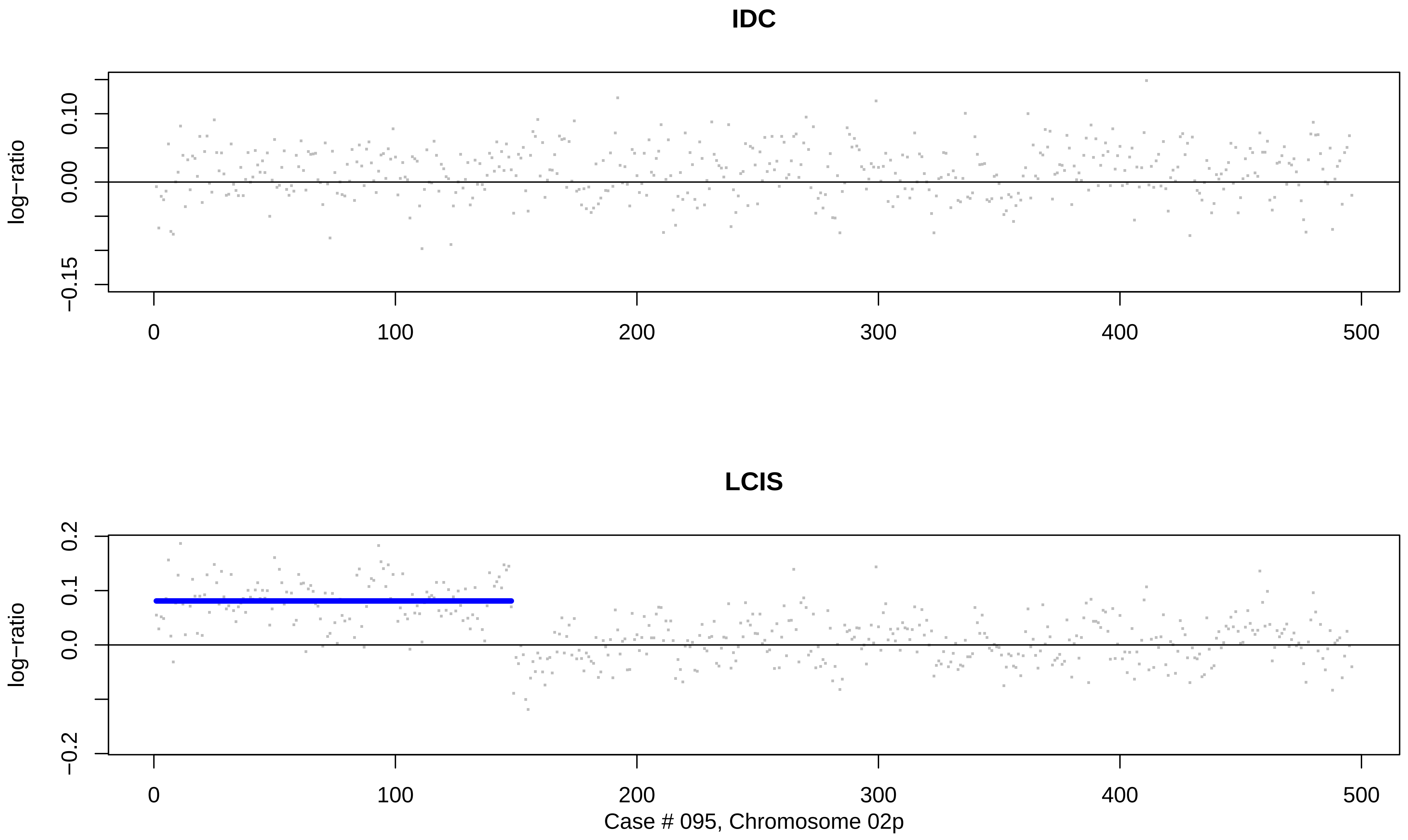
IDC
log−ratio
0.10
0.00
−0.15
0
100
200
300
400
500
LCIS
log−ratio
0.2
0.1
0.0
−0.2
0
100
200
300
400
500
Case # 095, Chromosome 02p

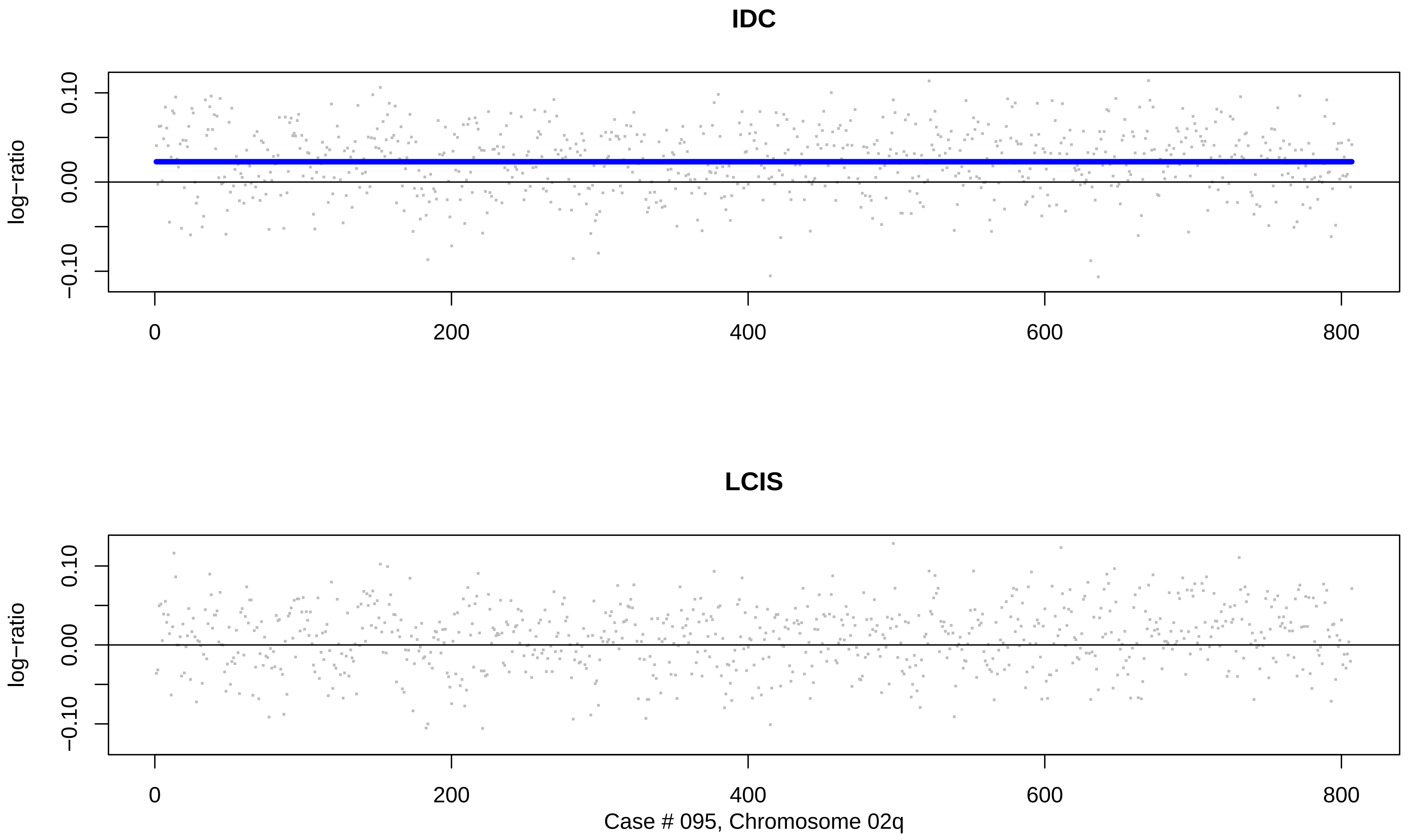
IDC
log−ratio
0.10
0.00
−0.10
0
200
400
600
800
LCIS
log−ratio
0.10
0.00
−0.10
0
200
400
600
800
Case # 095, Chromosome 02q

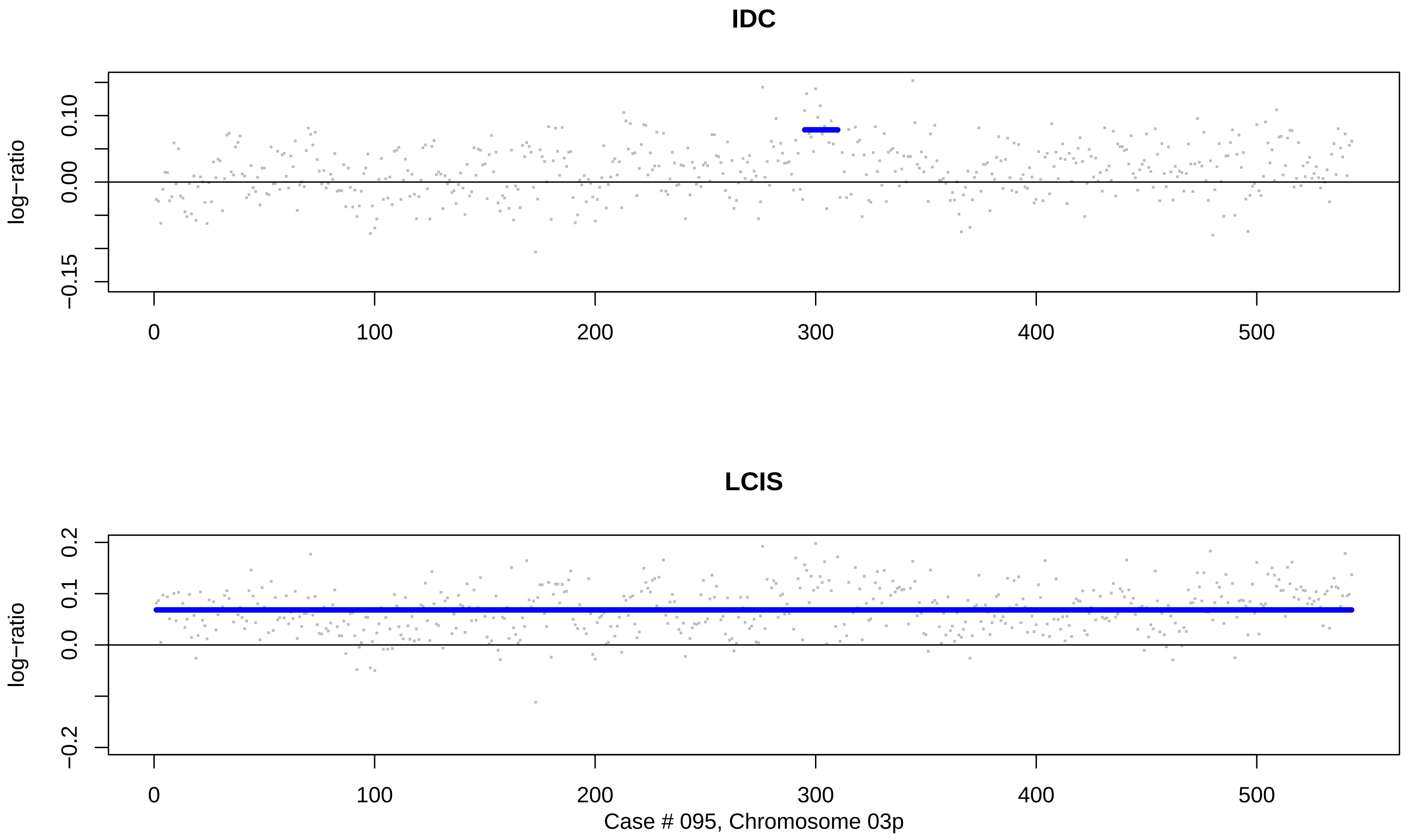

IDC
log−ratio
−0.15
0.00
0.10
0
100
200
300
400
500
LCIS
log−ratio
−0.2
0.0
0.1
0.2
0
100
200
300
400
500
Case # 095, Chromosome 03p

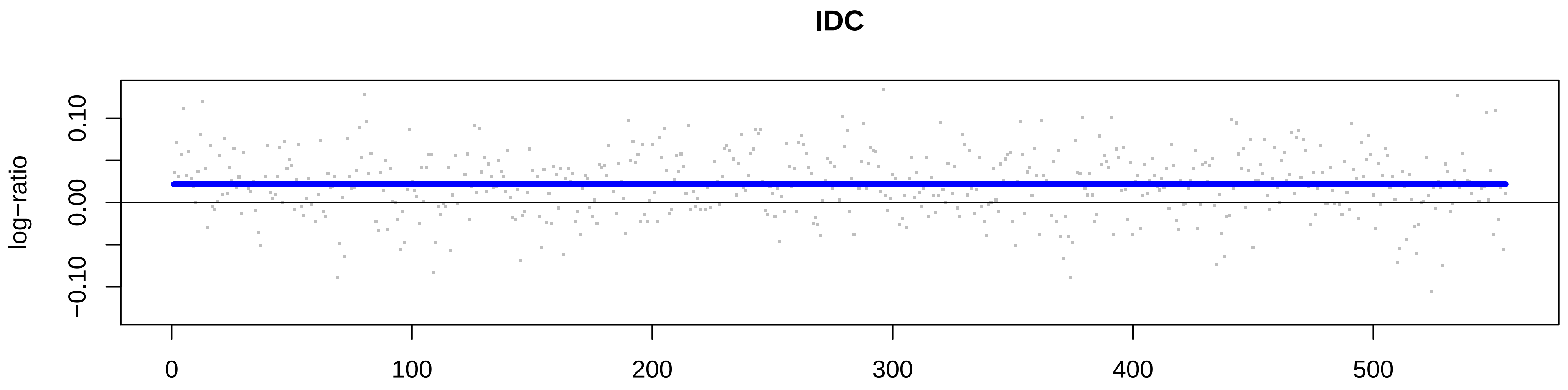
IDC
log−ratio
0.10
0.00
−0.10
0
100
200
300
400
500

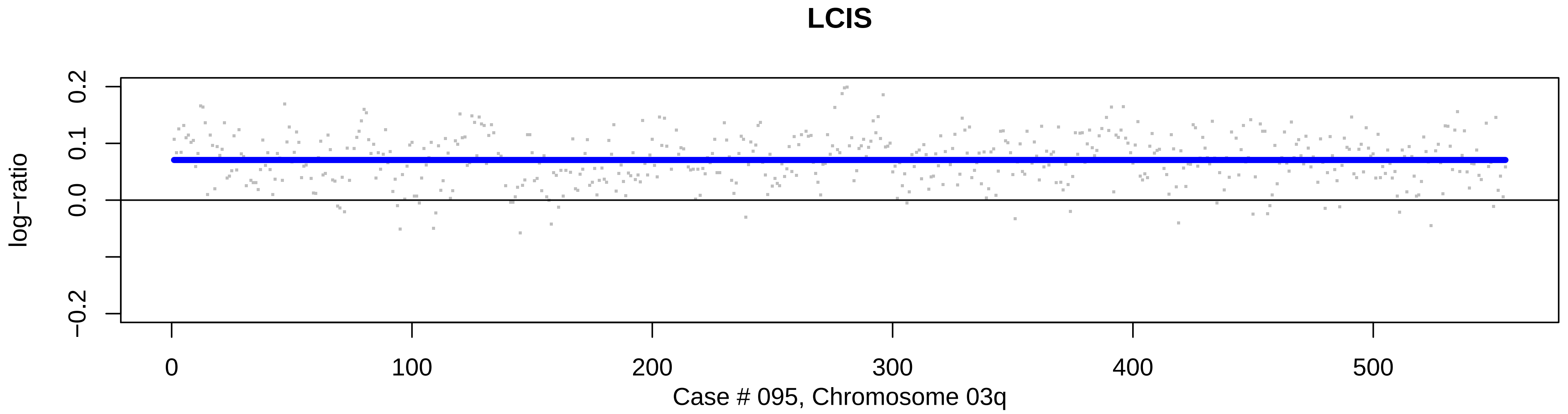
LCIS
log−ratio
0.2
0.1
0.0
−0.2
0
100
200
300
400
500
Case # 095, Chromosome 03q

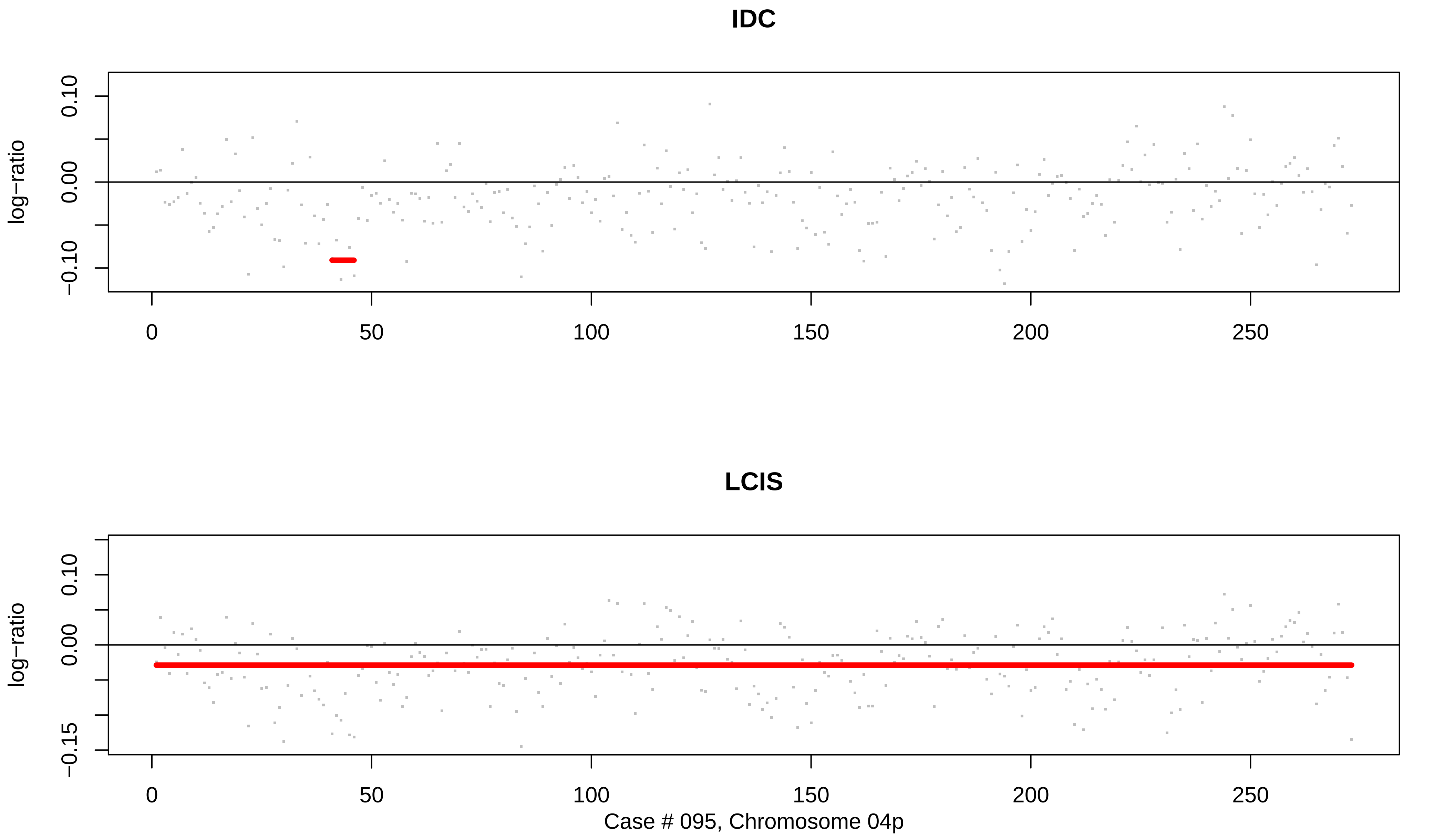

IDC
log−ratio
0.10
0.00
−0.10
0
50
100
150
200
250
LCIS
log−ratio
0.10
0.00
−0.15
0
50
100
150
200
250
Case # 095, Chromosome 04p

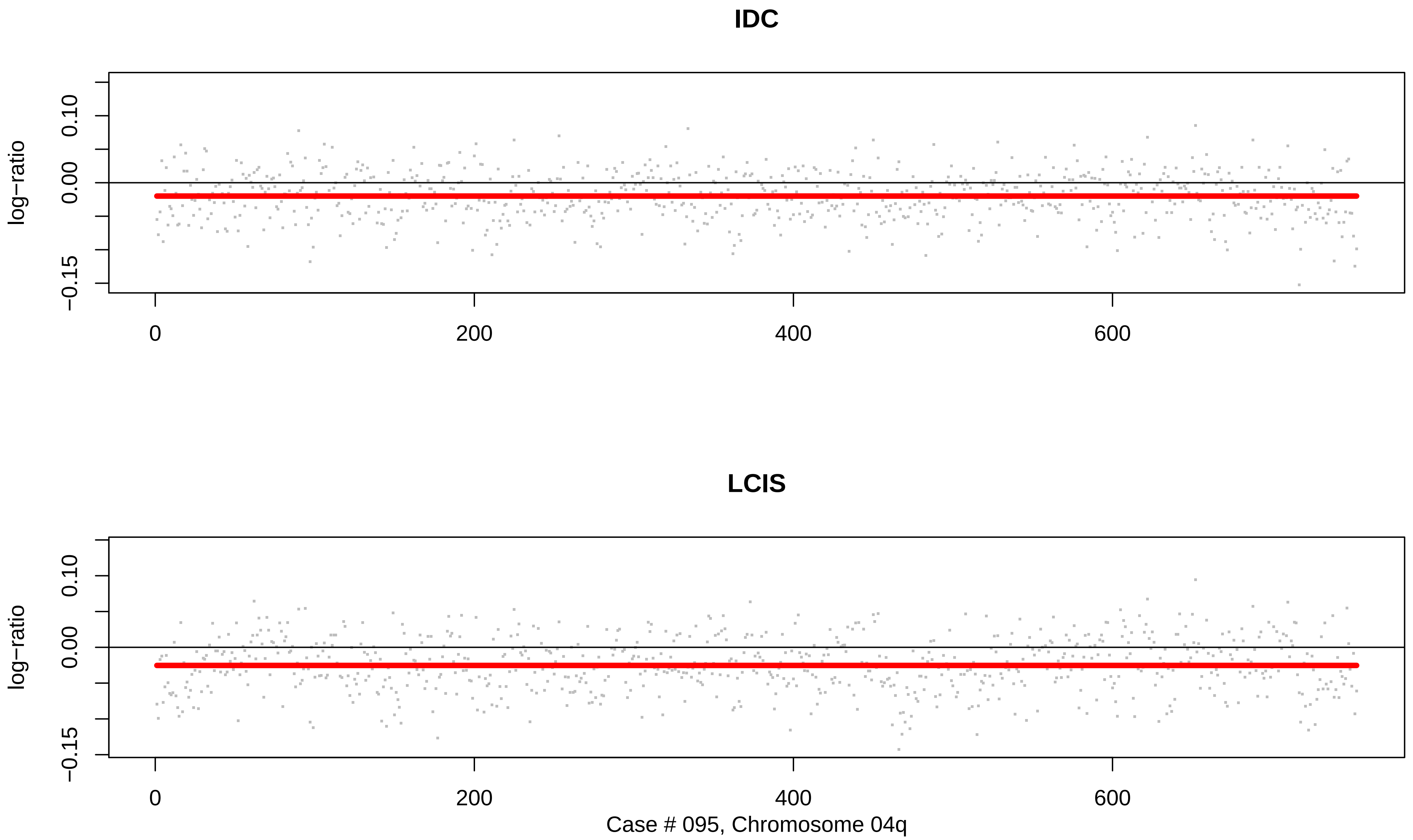
IDC
log−ratio
0.10
0.00
−0.15
0
200
400
600
LCIS
log−ratio
0.10
0.00
−0.15
0
200
400
600
Case # 095, Chromosome 04q

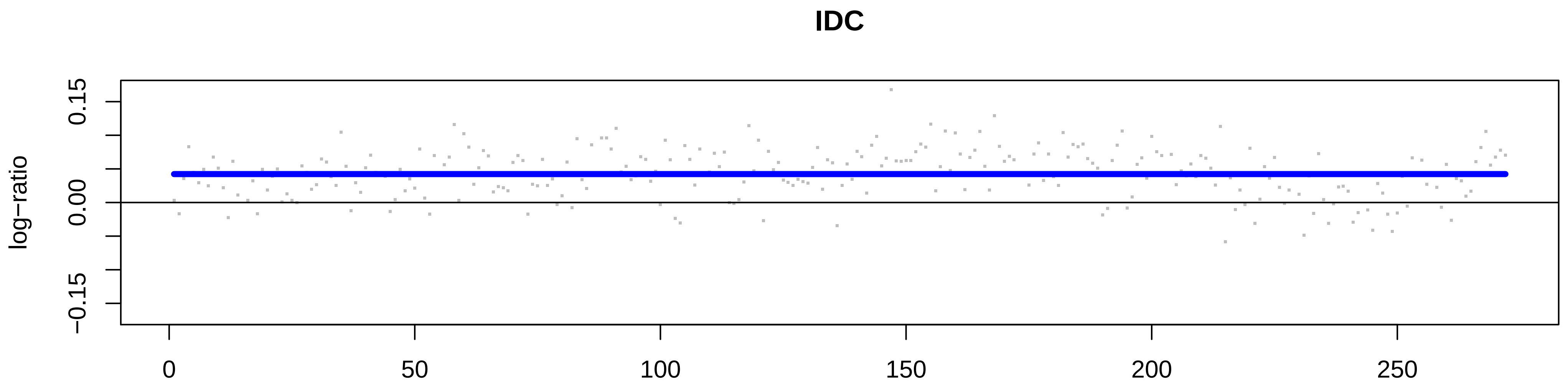
IDC
log−ratio
−0.15
0.00
0.15
0
50
100
150
200
250

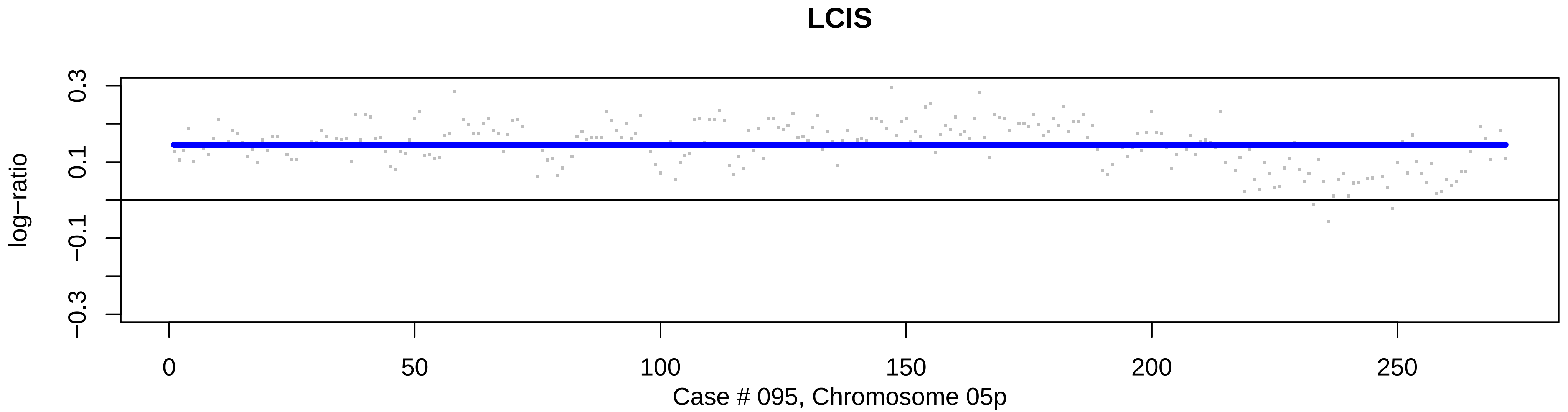
LCIS
log−ratio
−0.3
−0.1
0.1
0.3
0
50
100
150
200
250
Case # 095, Chromosome 05p

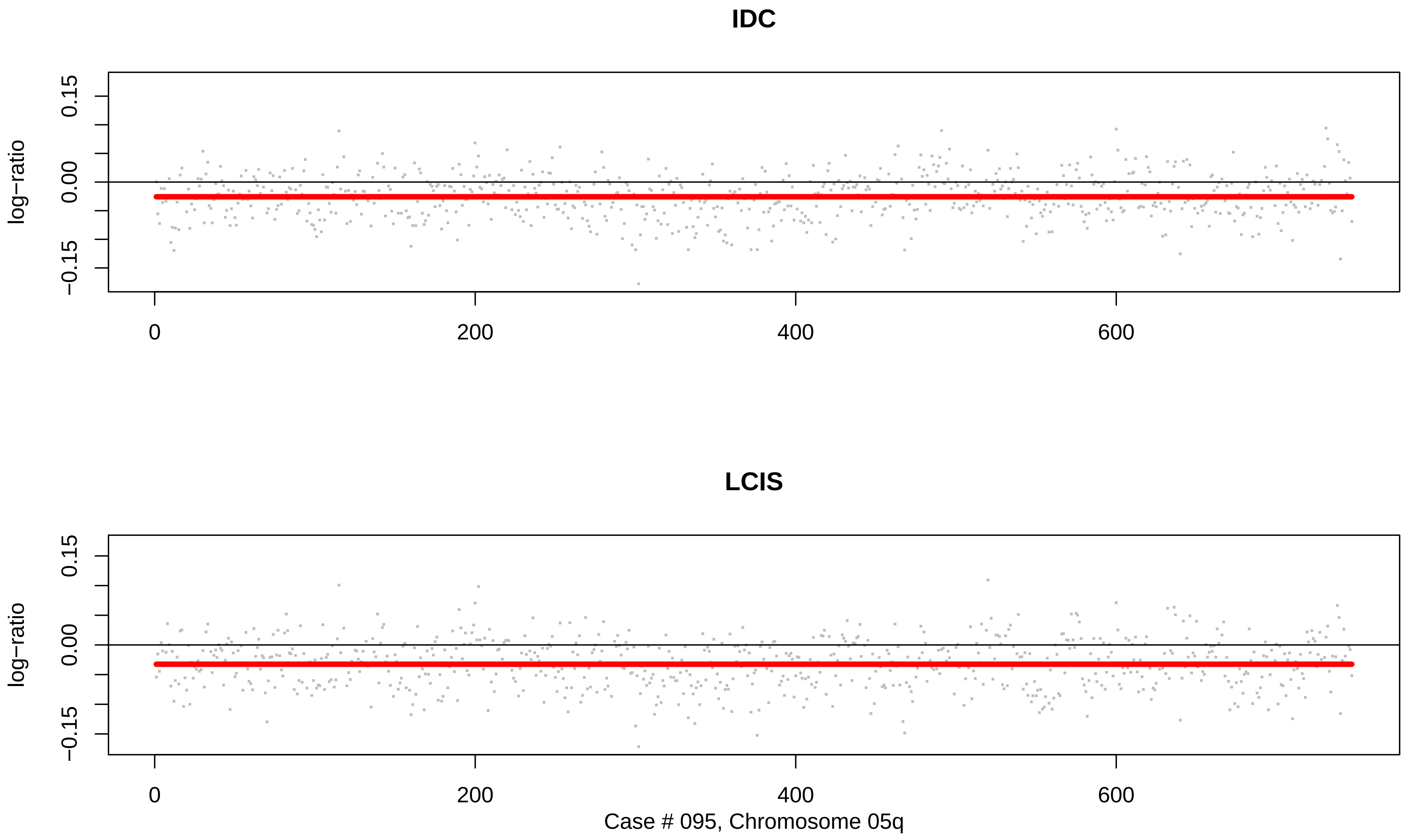
IDC
log−ratio
0.15
0.00
−0.15
0
200
400
600
LCIS
log−ratio
0.15
0.00
−0.15
0
200
400
600
Case # 095, Chromosome 05q

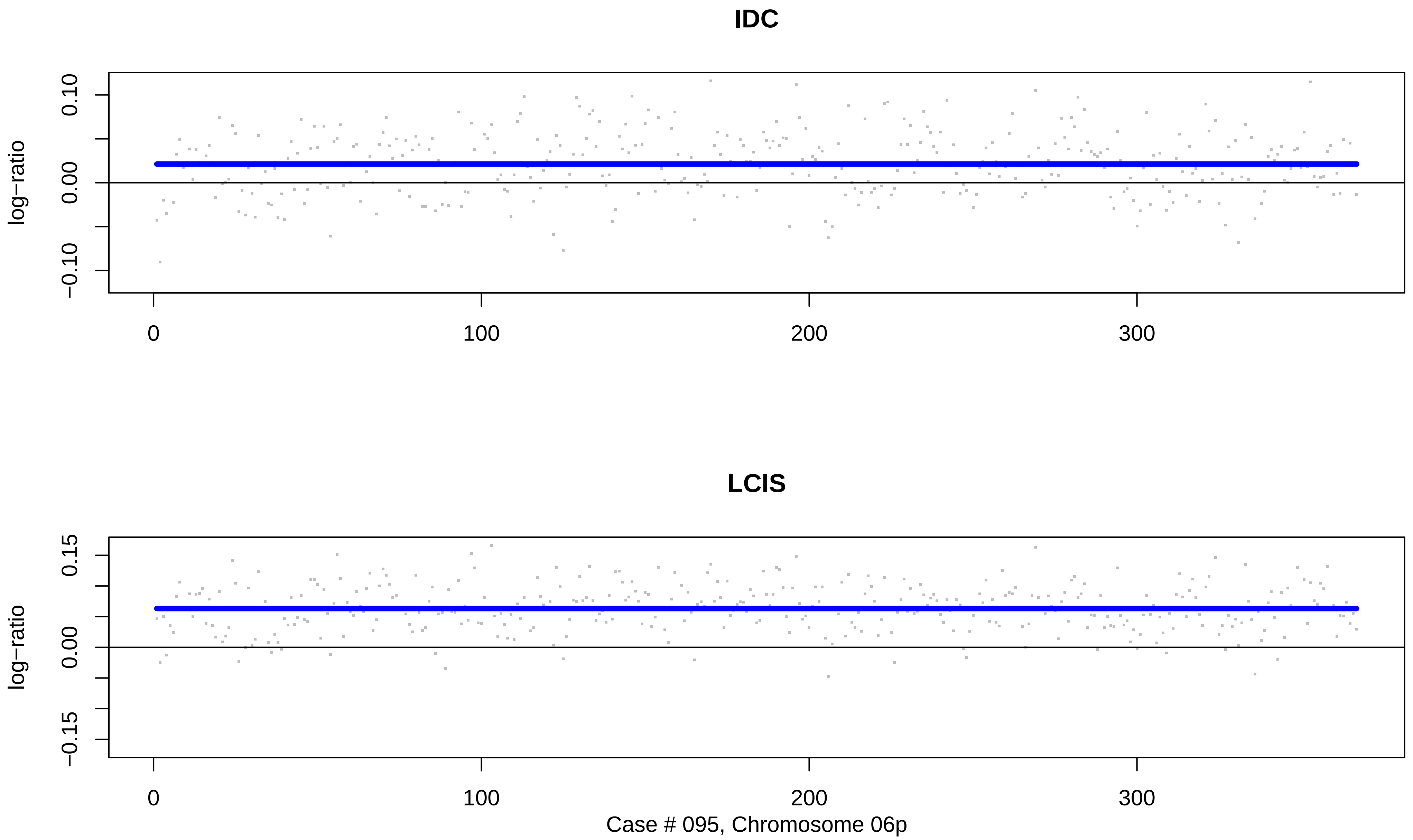
IDC
log−ratio
0.10
0.00
−0.10
0
100
200
300
LCIS
log−ratio
0.15
0.00
−0.15
0
100
200
300
Case # 095, Chromosome 06p

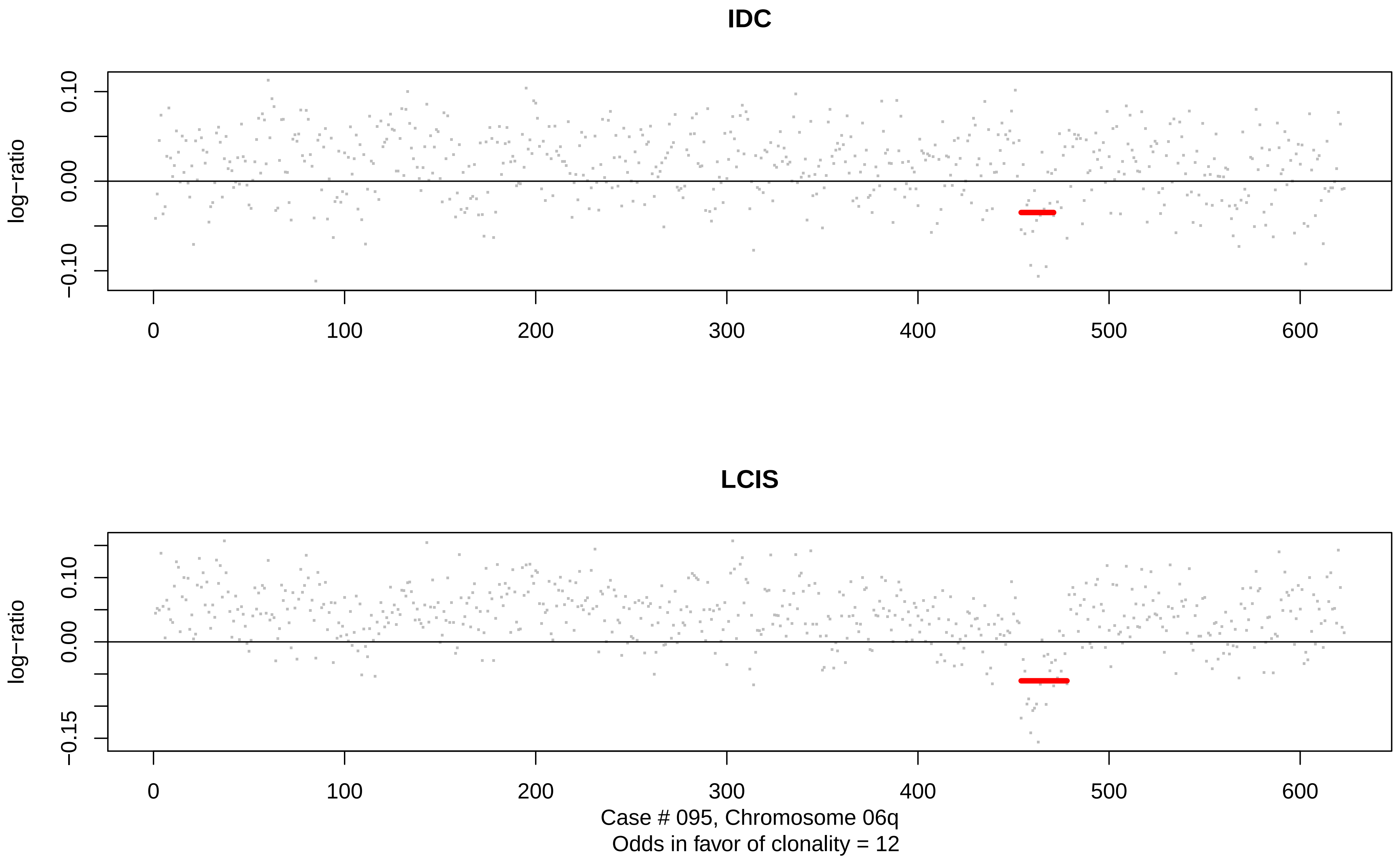
IDC
log−ratio
0.10
0.00
−0.10
0
100
200
300
400
500
600
LCIS
log−ratio
0.10
0.00
−0.15
0
100
200
300
400
500
600
Case # 095, Chromosome 06q
Odds in favor of clonality = 12

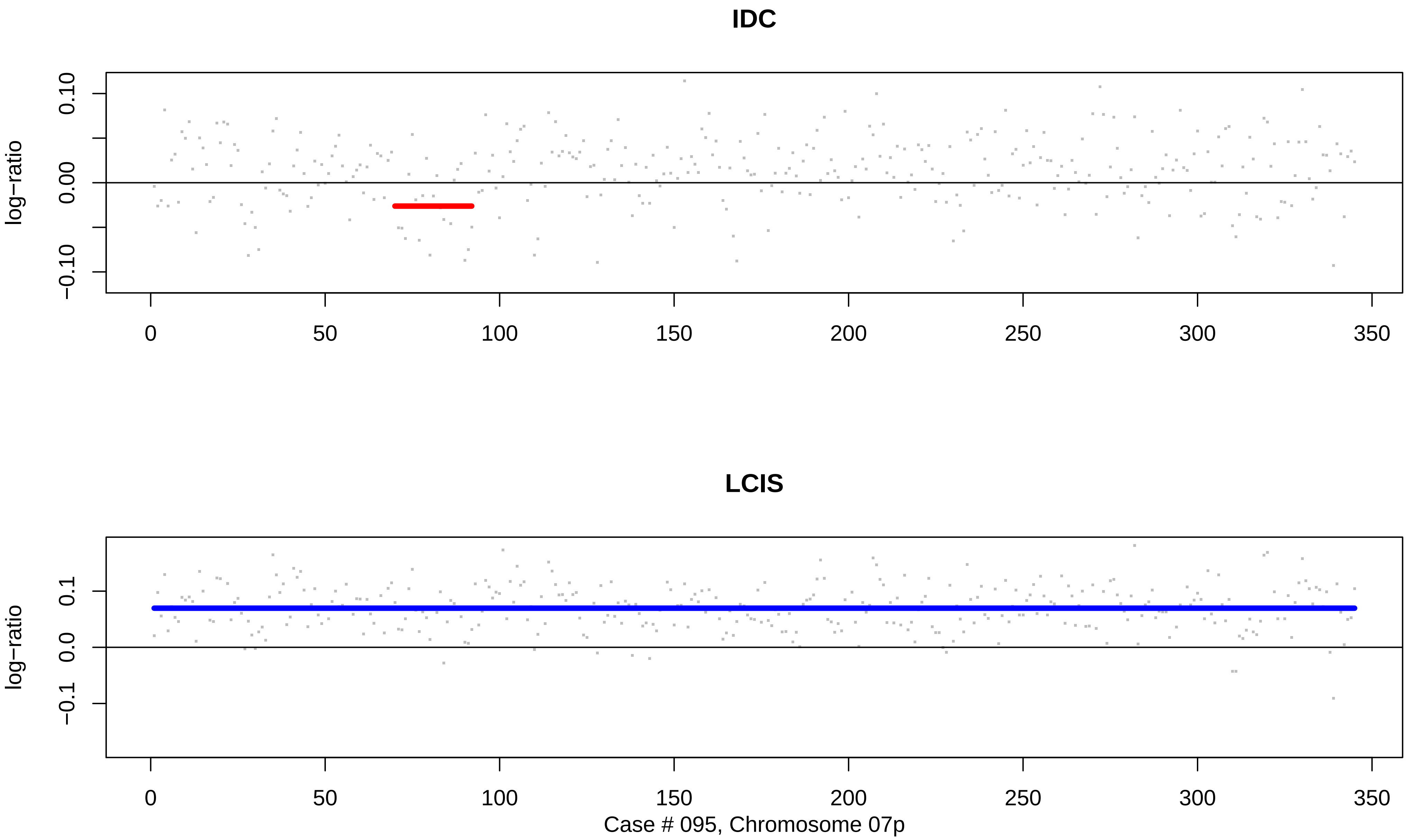
IDC
log−ratio
0.10
0.00
−0.10
0
50
100
150
200
250
300
350
LCIS
log−ratio
0.1
0.0
−0.1
0
50
100
150
200
250
300
350
Case # 095, Chromosome 07p

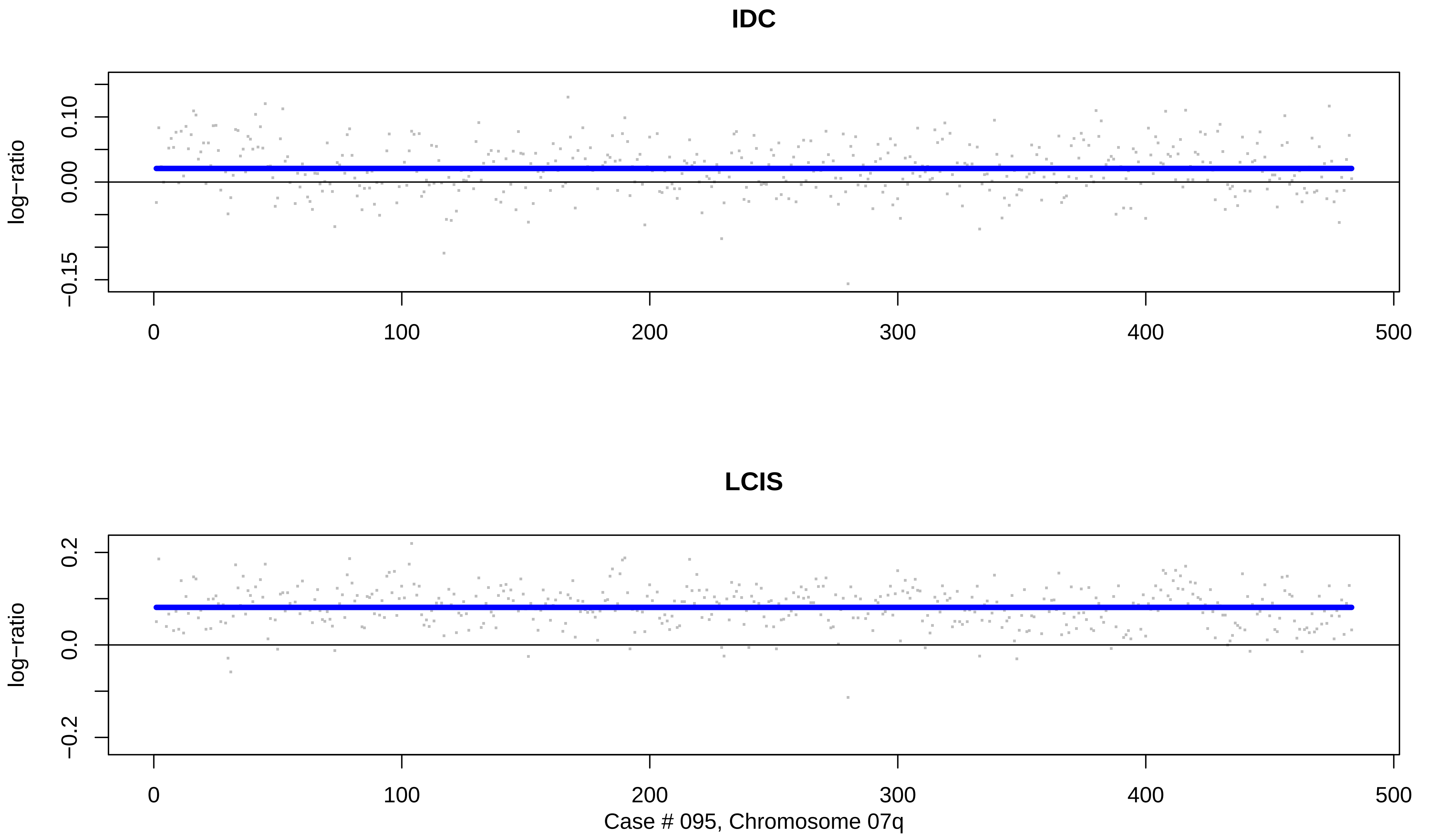
IDC
log−ratio
0.10
0.00
−0.15
0
100
200
300
400
500
LCIS
log−ratio
0.2
0.0
−0.2
0
100
200
300
400
500
Case # 095, Chromosome 07q

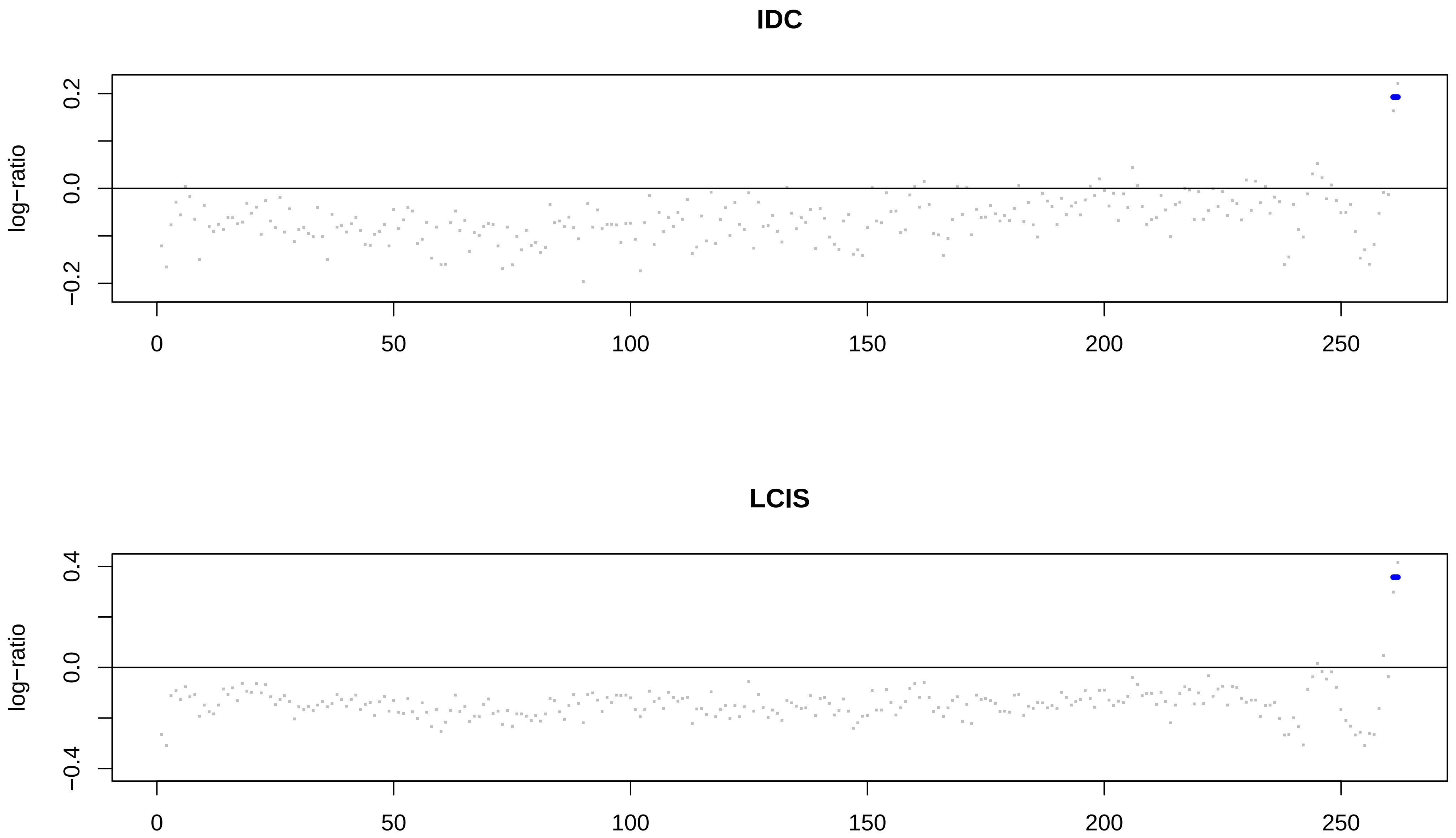

Case # 095, Chromosome 08p
Odds in favor of clonality = 61.5

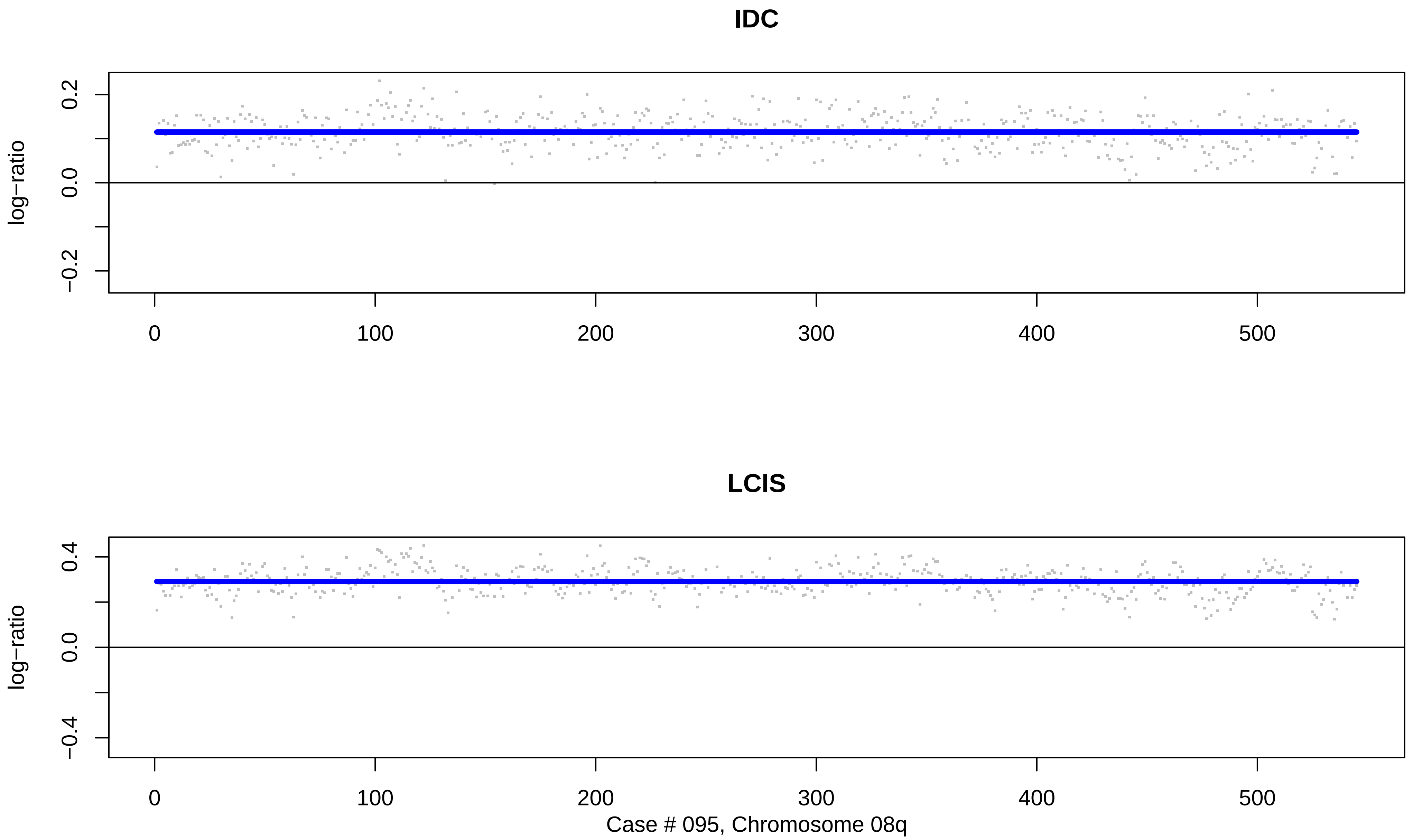

IDC
log−ratio
0.2
0.0
−0.2
0
100
200
300
400
500
LCIS
log−ratio
0.4
0.0
−0.4
0
100
200
300
400
500
Case # 095, Chromosome 08q

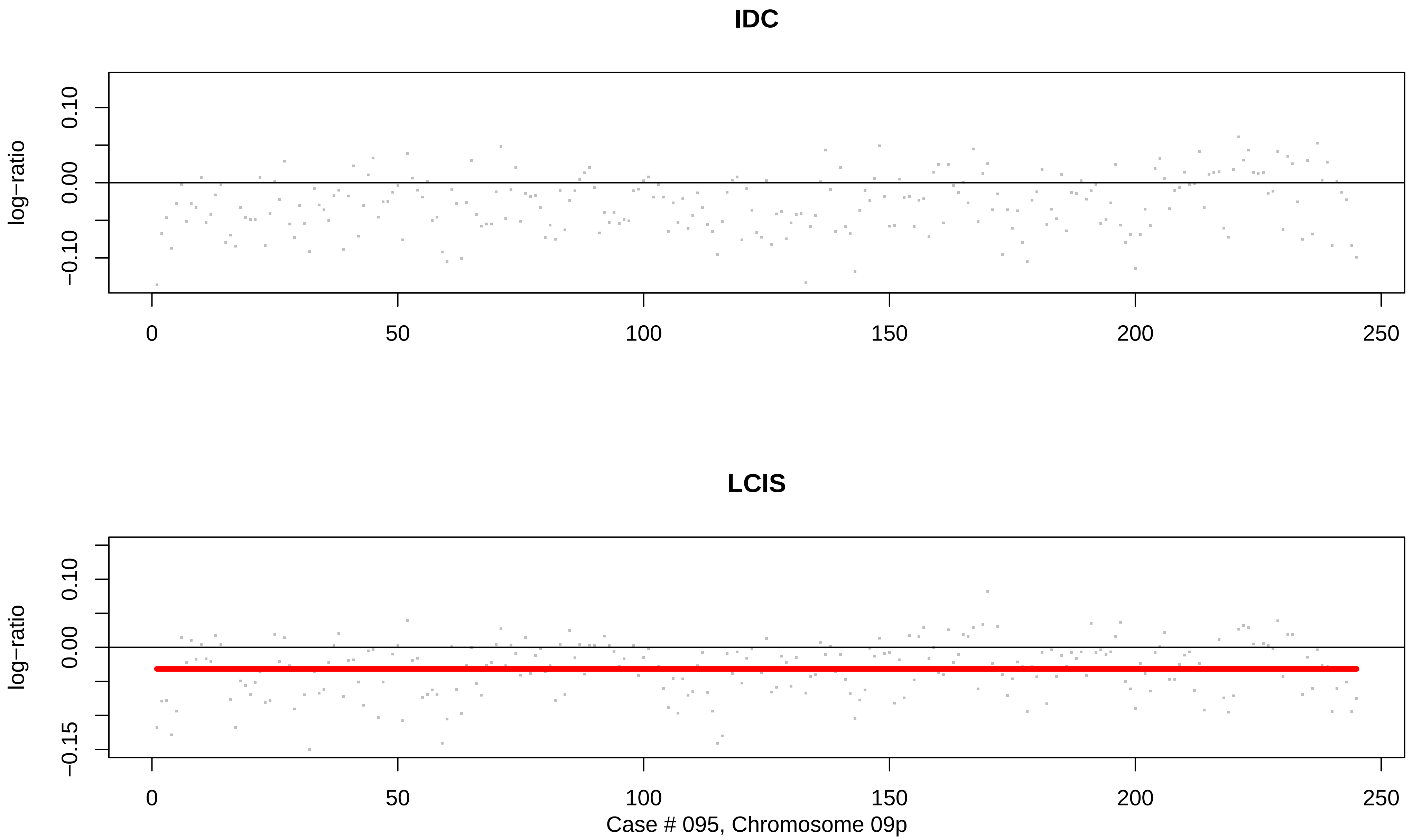
IDC
log−ratio
0.10
0.00
−0.10
0
50
100
150
200
250
LCIS
log−ratio
0.10
0.00
−0.15
0
50
100
150
200
250
Case # 095, Chromosome 09p

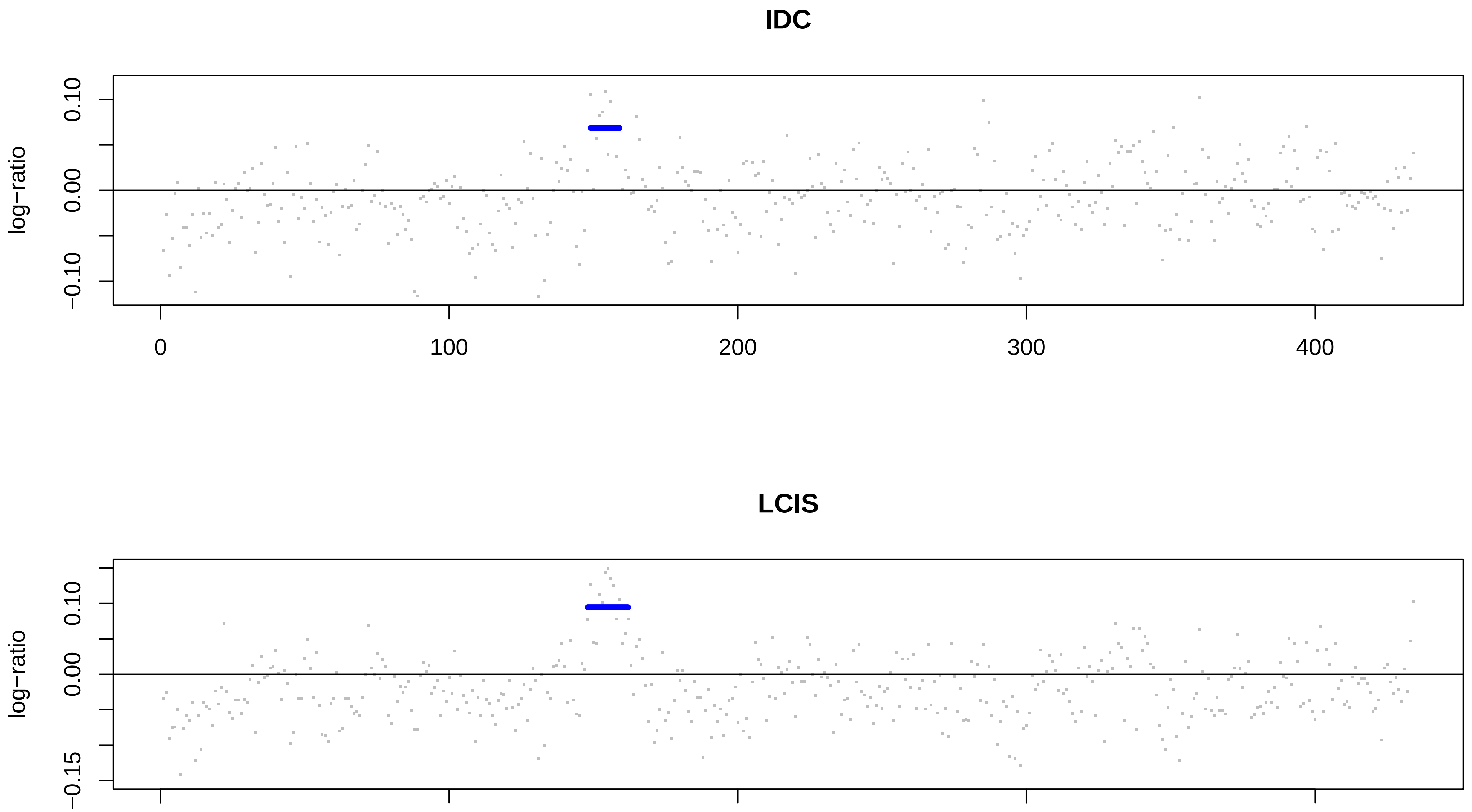

Case # 095, Chromosome 09q
Odds in favor of clonality = 13.1

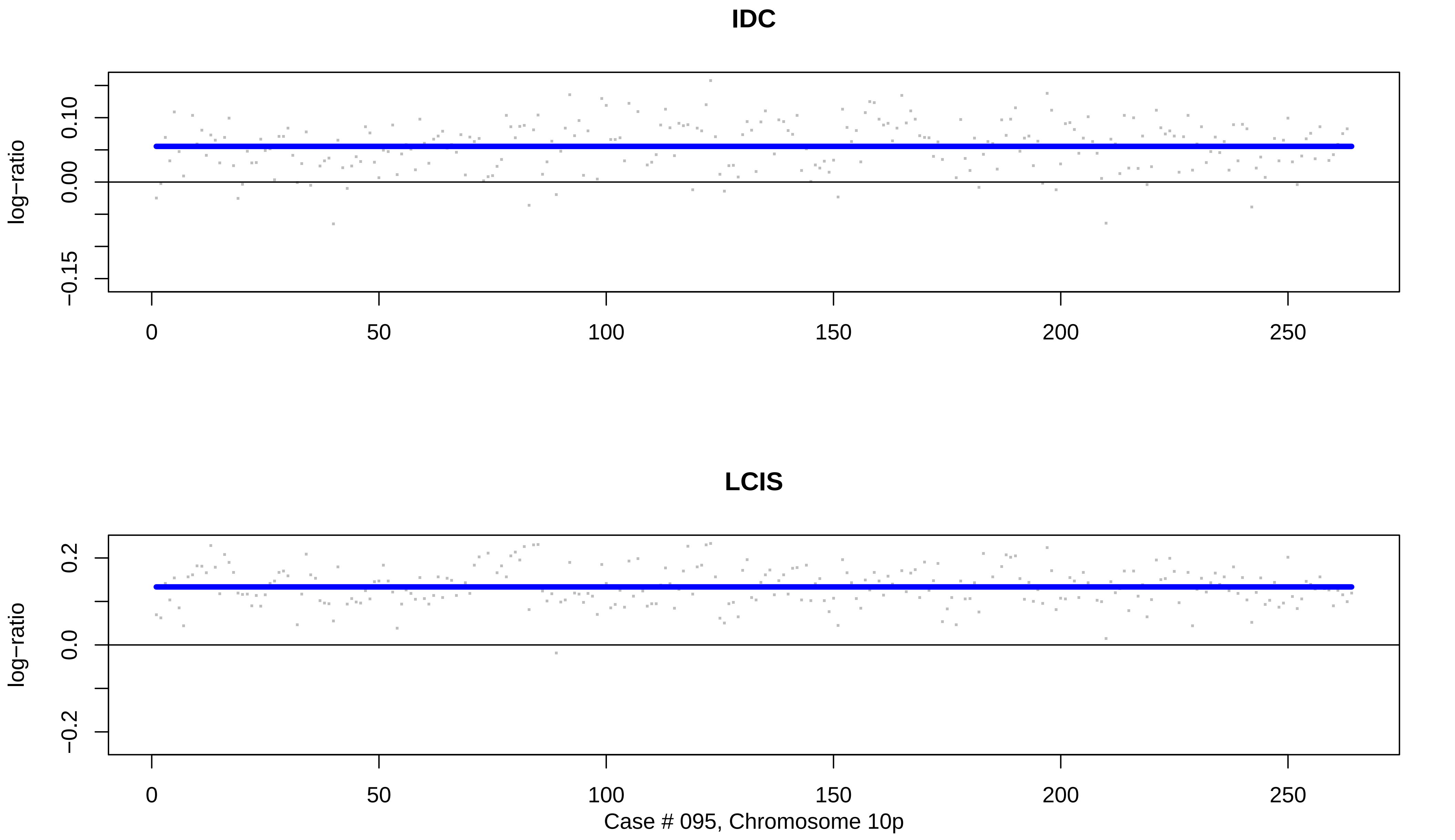
IDC
log−ratio
−0.15
0.00
0.10
0
50
100
150
200
250
LCIS
log−ratio
−0.2
0.0
0.2
0
50
100
150
200
250
Case # 095, Chromosome 10p

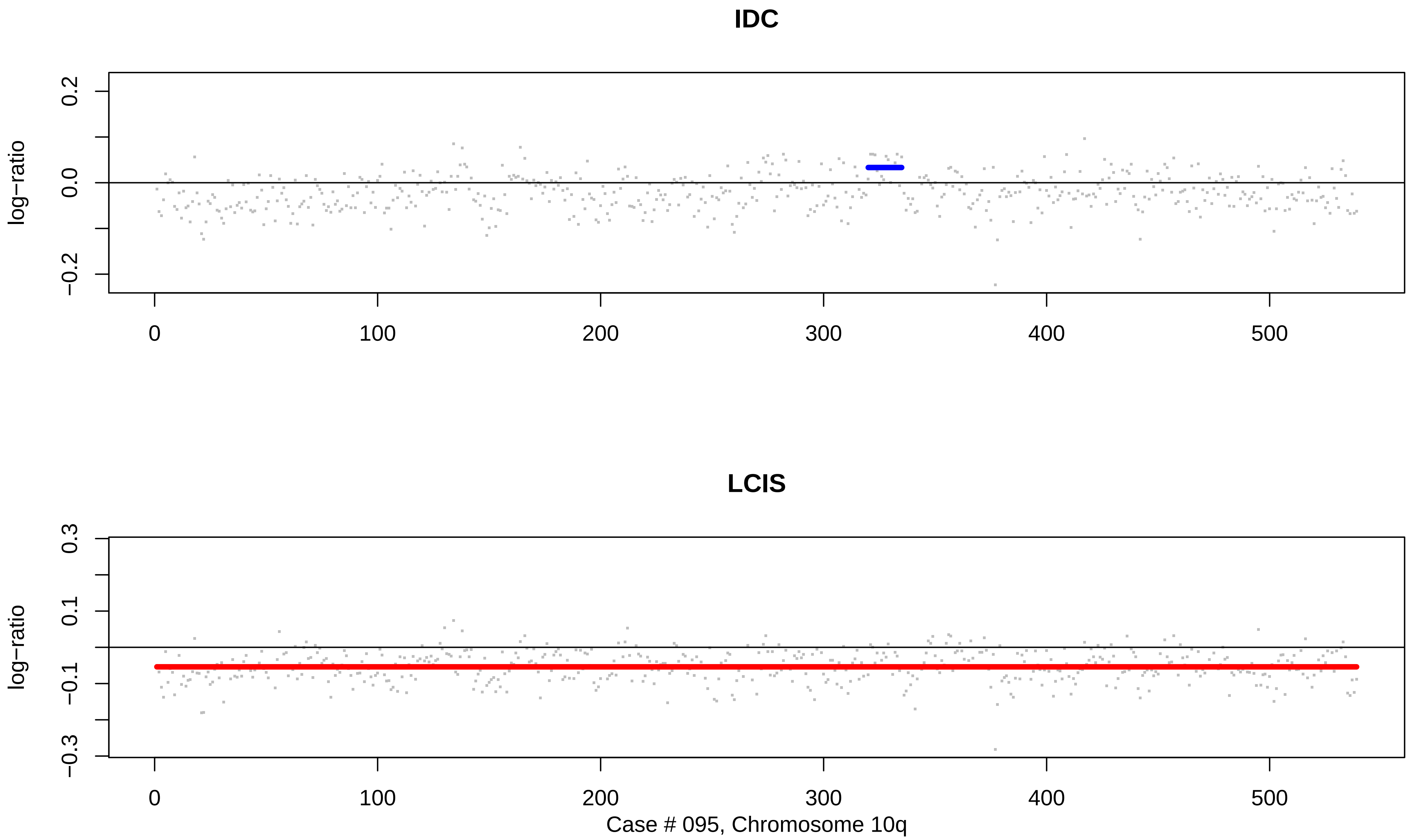

IDC
log−ratio
0.2
0.0
−0.2
0
100
200
300
400
500
LCIS
log−ratio
0.3
0.1
−0.1
−0.3
0
100
200
300
400
500
Case # 095, Chromosome 10q

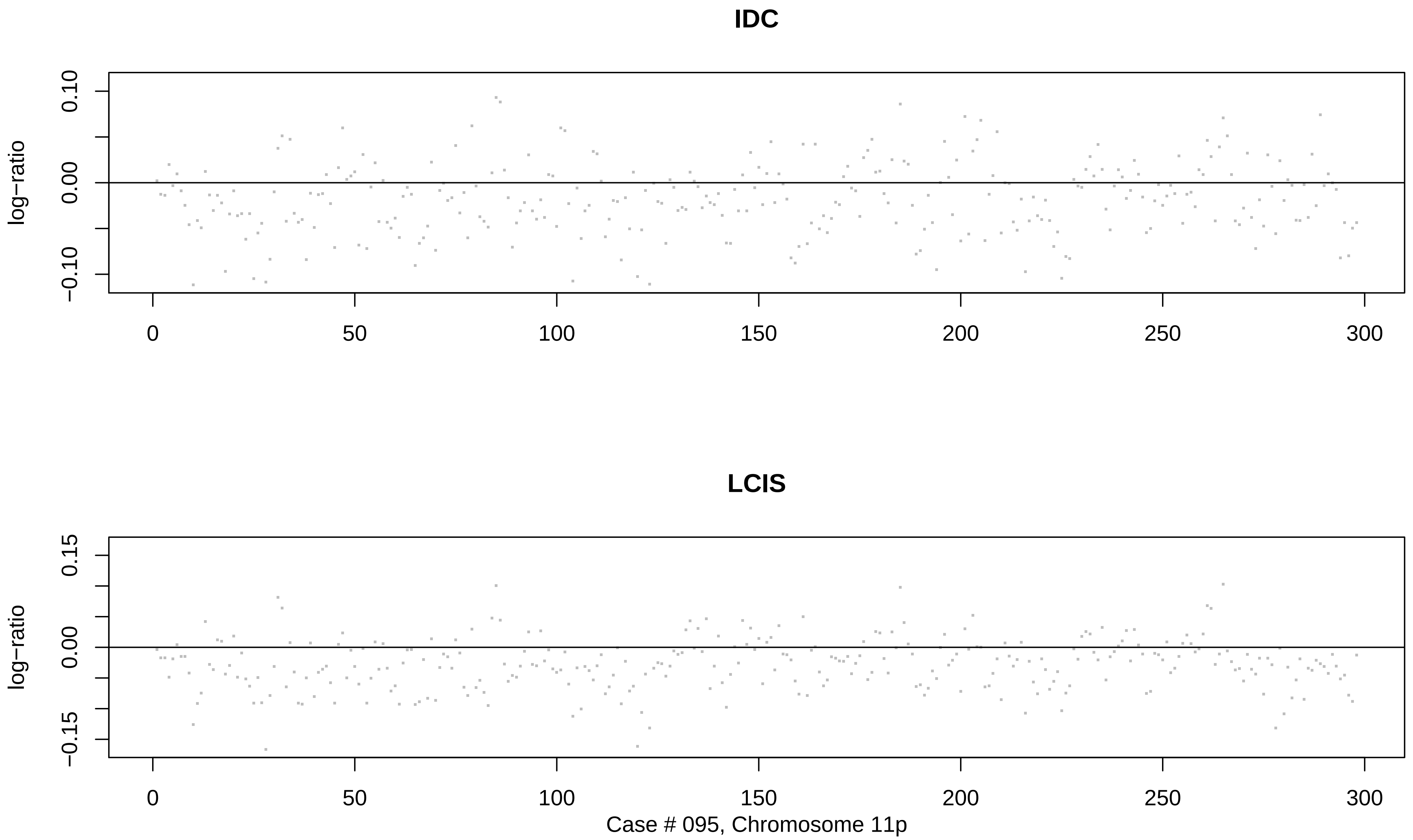
IDC
log−ratio
0.10
0.00
−0.10
0
50
100
150
200
250
300
LCIS
log−ratio
0.15
0.00
−0.15
0
50
100
150
200
250
300
Case # 095, Chromosome 11p

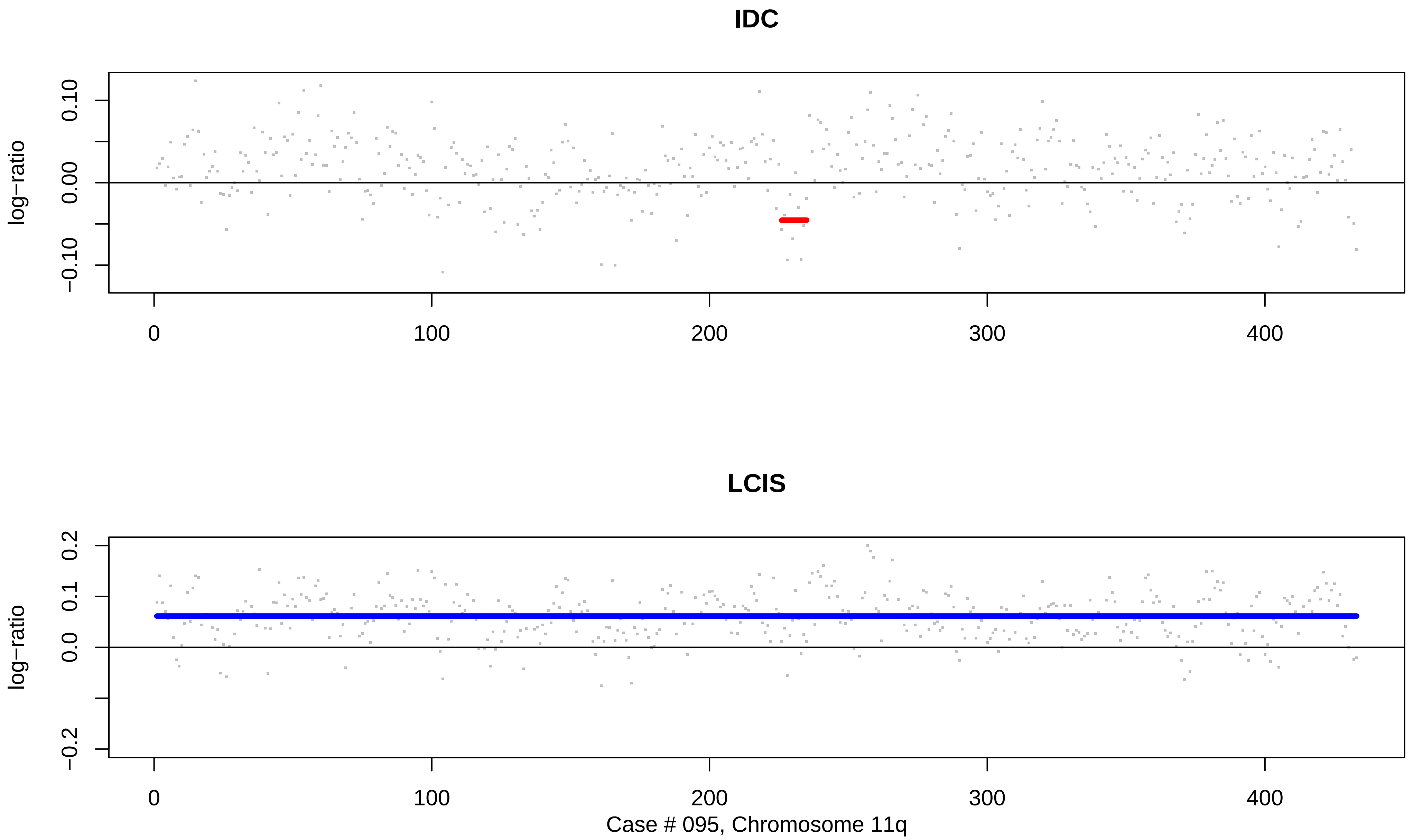
IDC
log−ratio
0.10
0.00
−0.10
0
100
200
300
400
LCIS
log−ratio
0.2
0.1
0.0
−0.2
0
100
200
300
400
Case # 095, Chromosome 11q

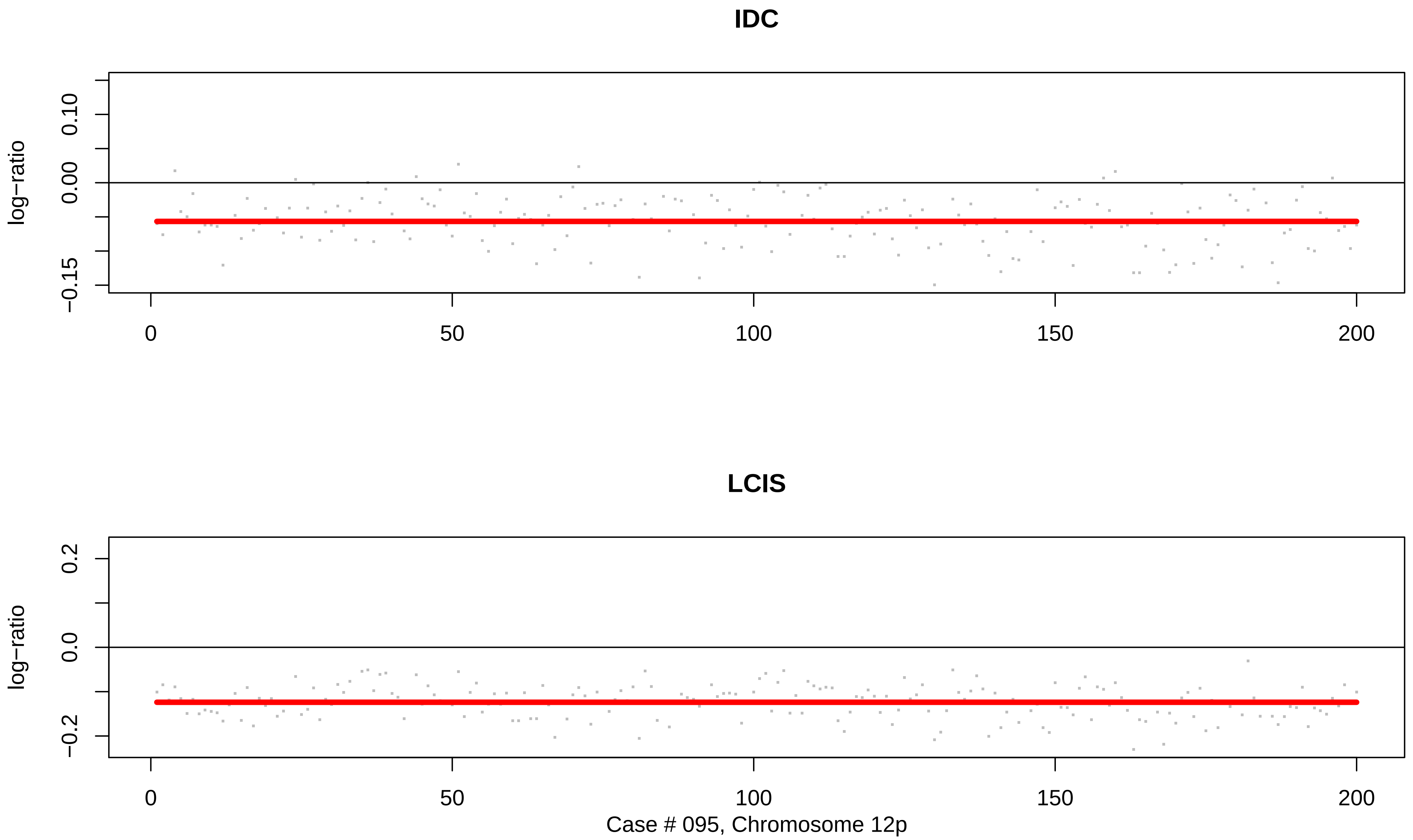
IDC
log−ratio
0.10
0.00
−0.15
0
50
100
150
200
LCIS
log−ratio
0.2
0.0
−0.2
0
50
100
150
200
Case # 095, Chromosome 12p

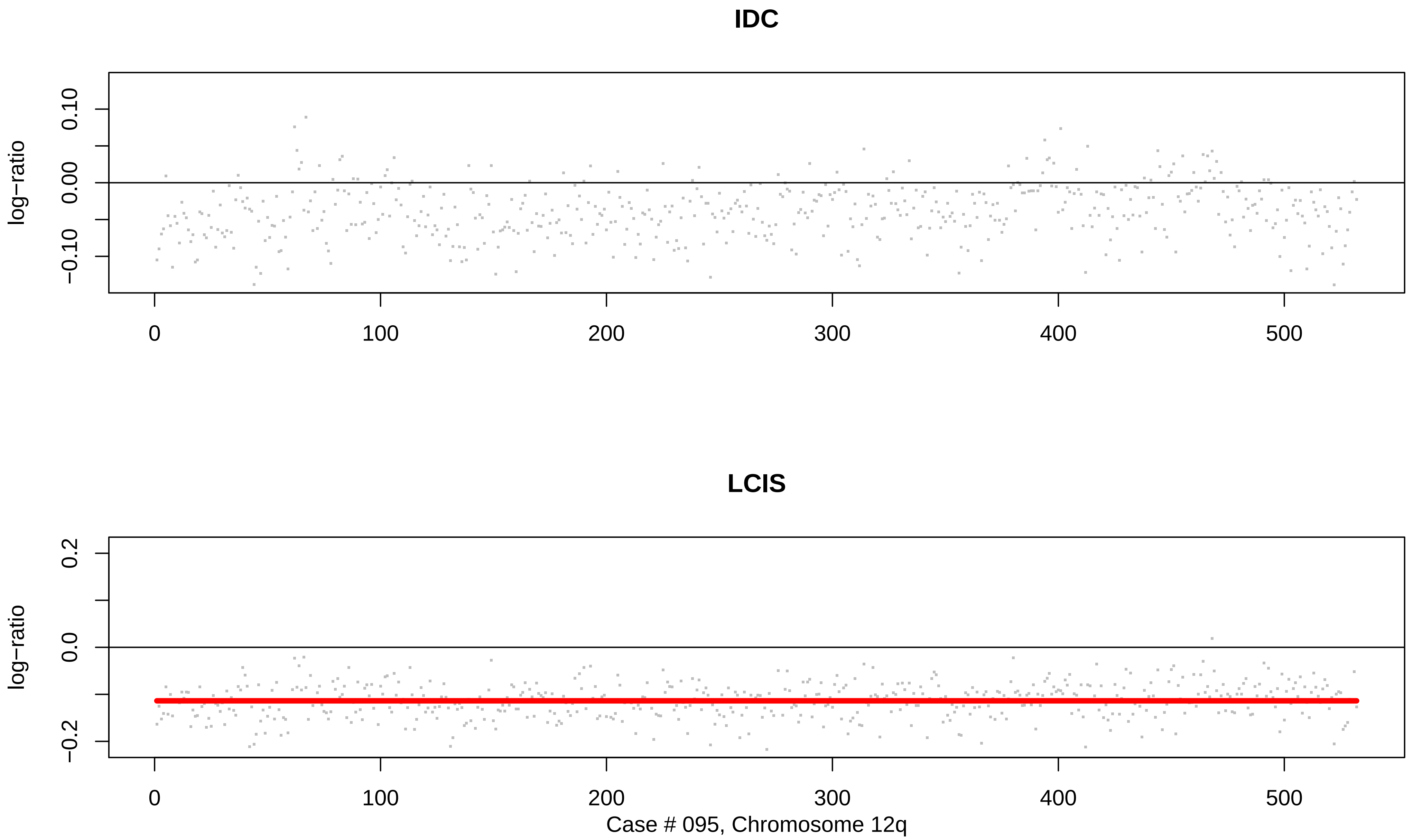

IDC
log−ratio
0.10
0.00
−0.10
0
100
200
300
400
500
LCIS
log−ratio
0.2
0.0
−0.2
0
100
200
300
400
500
Case # 095, Chromosome 12q

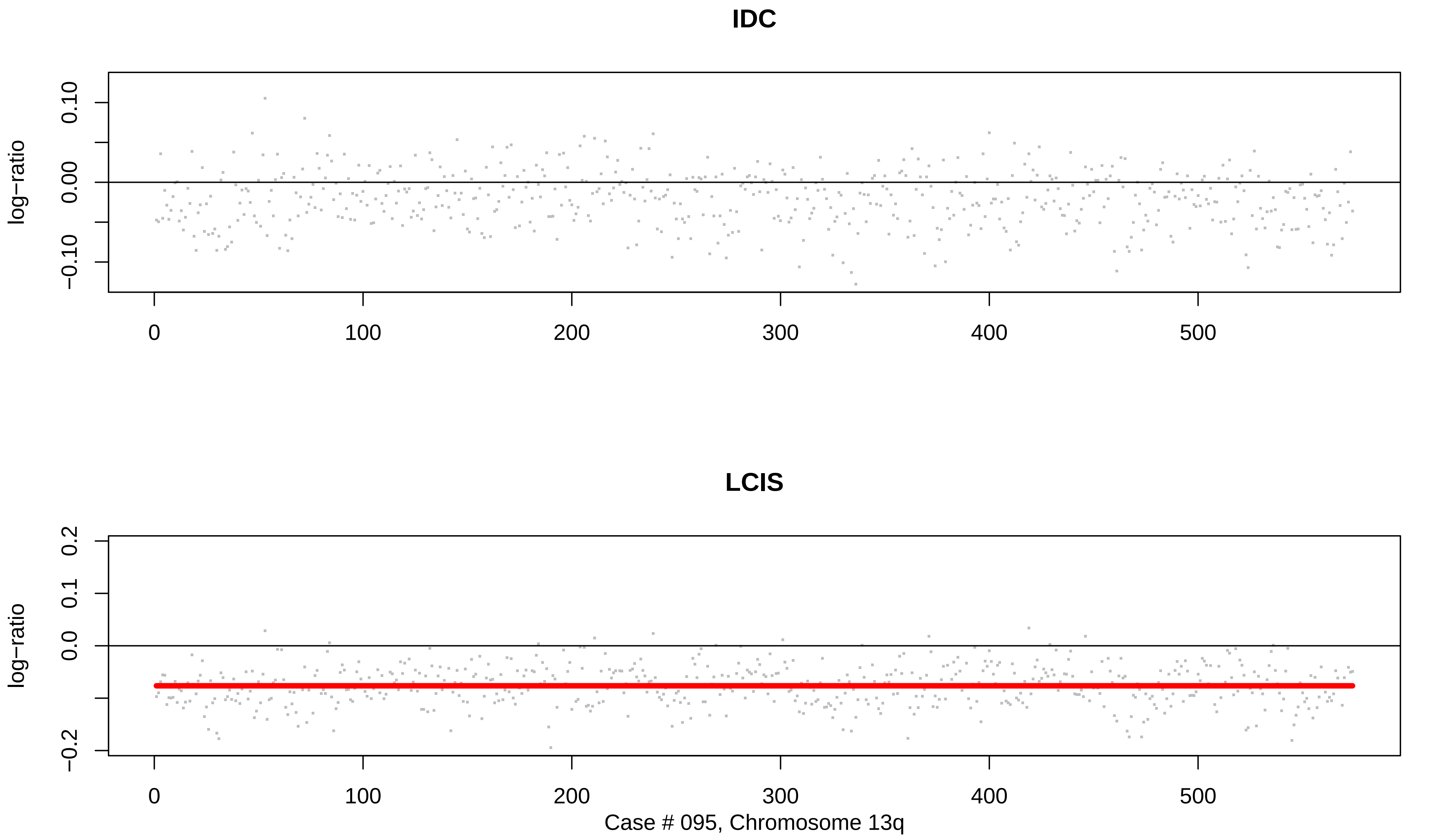
IDC
log−ratio
0.10
0.00
−0.10
0
100
200
300
400
500
LCIS
log−ratio
0.2
0.1
0.0
−0.2
0
100
200
300
400
500
Case # 095, Chromosome 13q

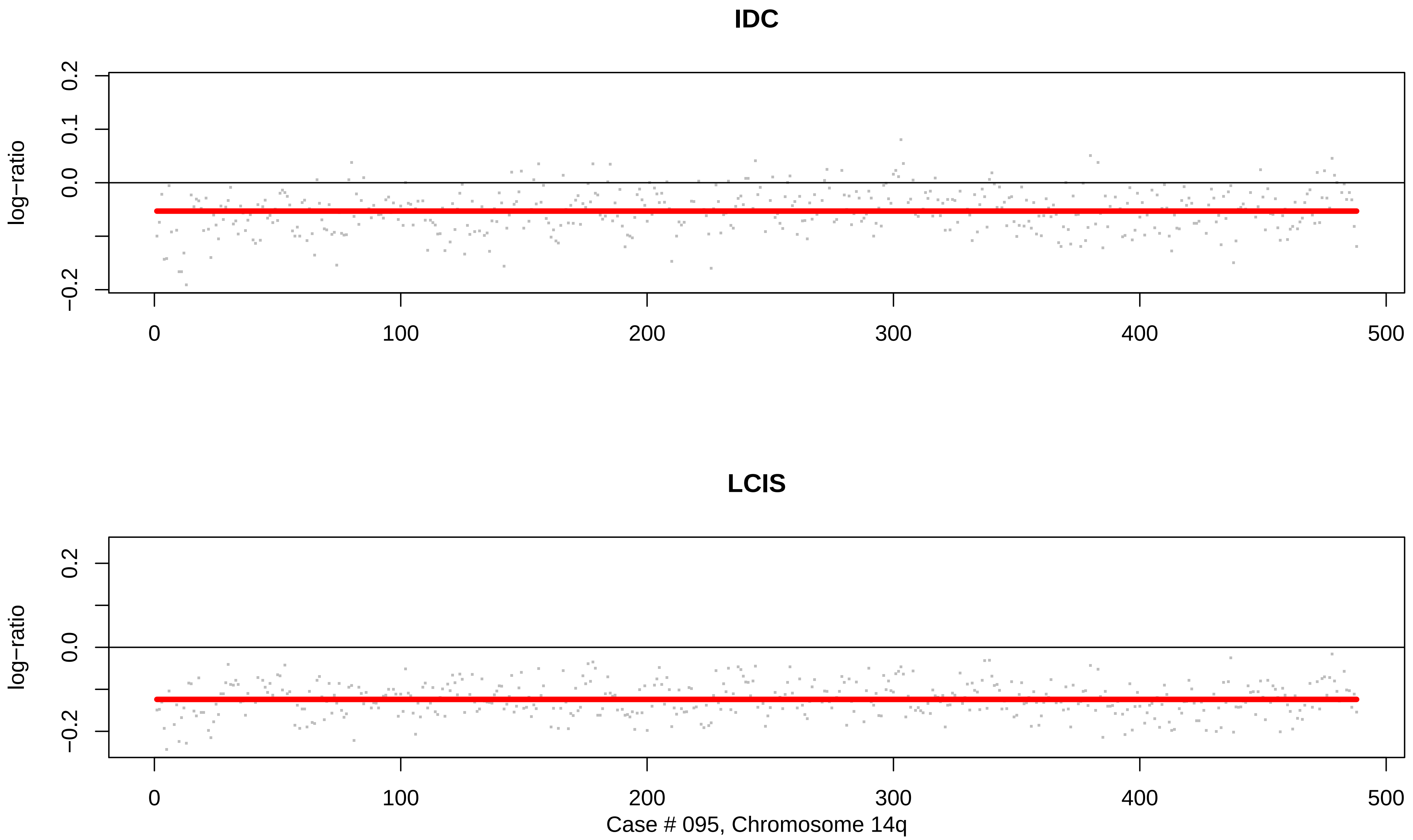
IDC
log−ratio
0.2
0.1
0.0
−0.2
0
100
200
300
400
500
LCIS
log−ratio
0.2
0.0
−0.2
0
100
200
300
400
500
Case # 095, Chromosome 14q

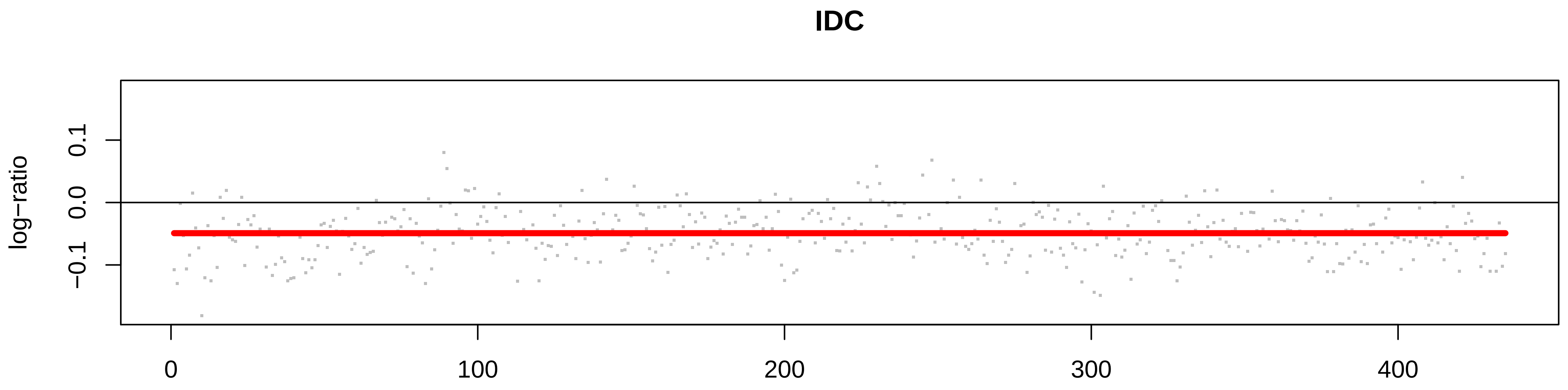
IDC
log−ratio
0.1
0.0
−0.1
0
100
200
300
400

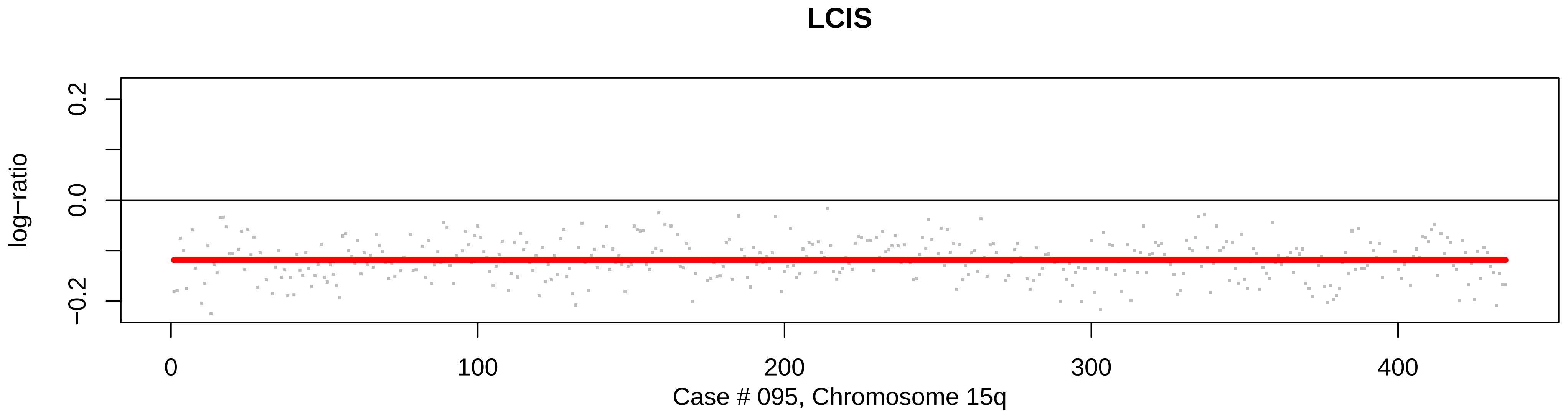
LCIS
log−ratio
0.2
0.0
−0.2
0
100
200
300
400
Case # 095, Chromosome 15q

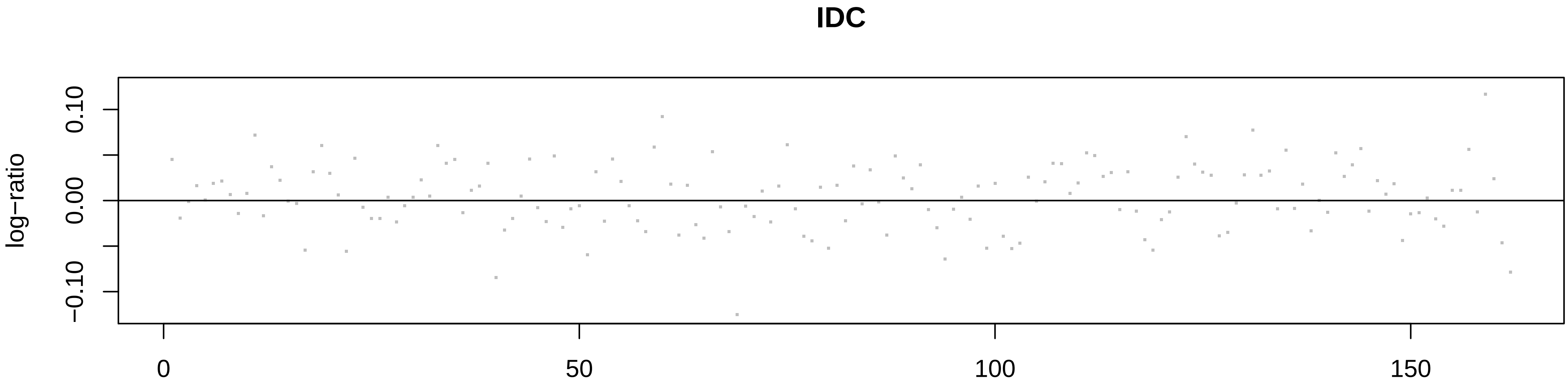
IDC
log−ratio
0.10
0.00
−0.10
0
50
100
150

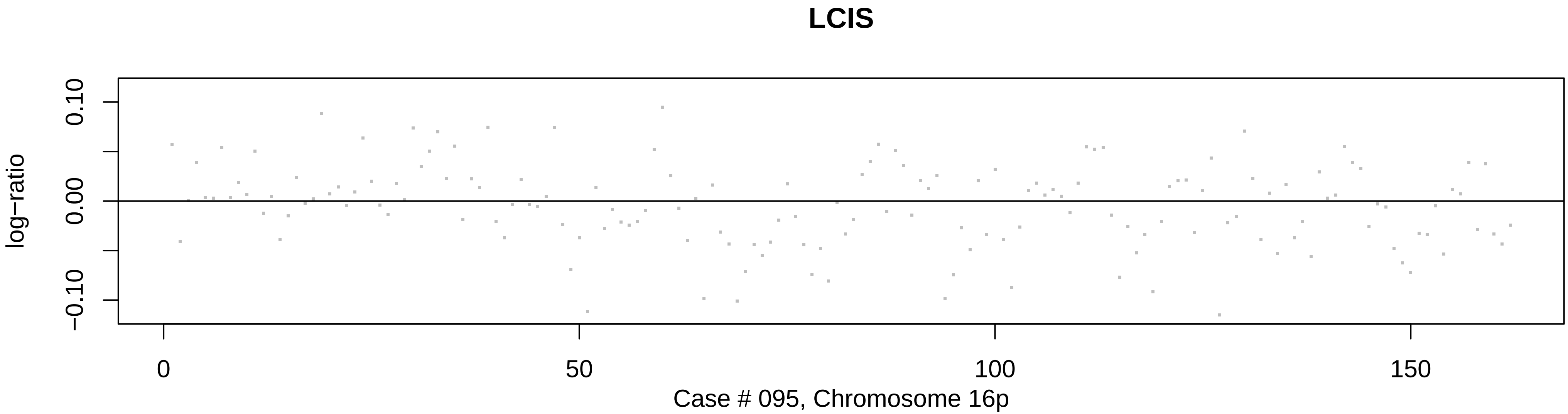
LCIS
log−ratio
0.10
0.00
−0.10
0
50
100
150
Case # 095, Chromosome 16p

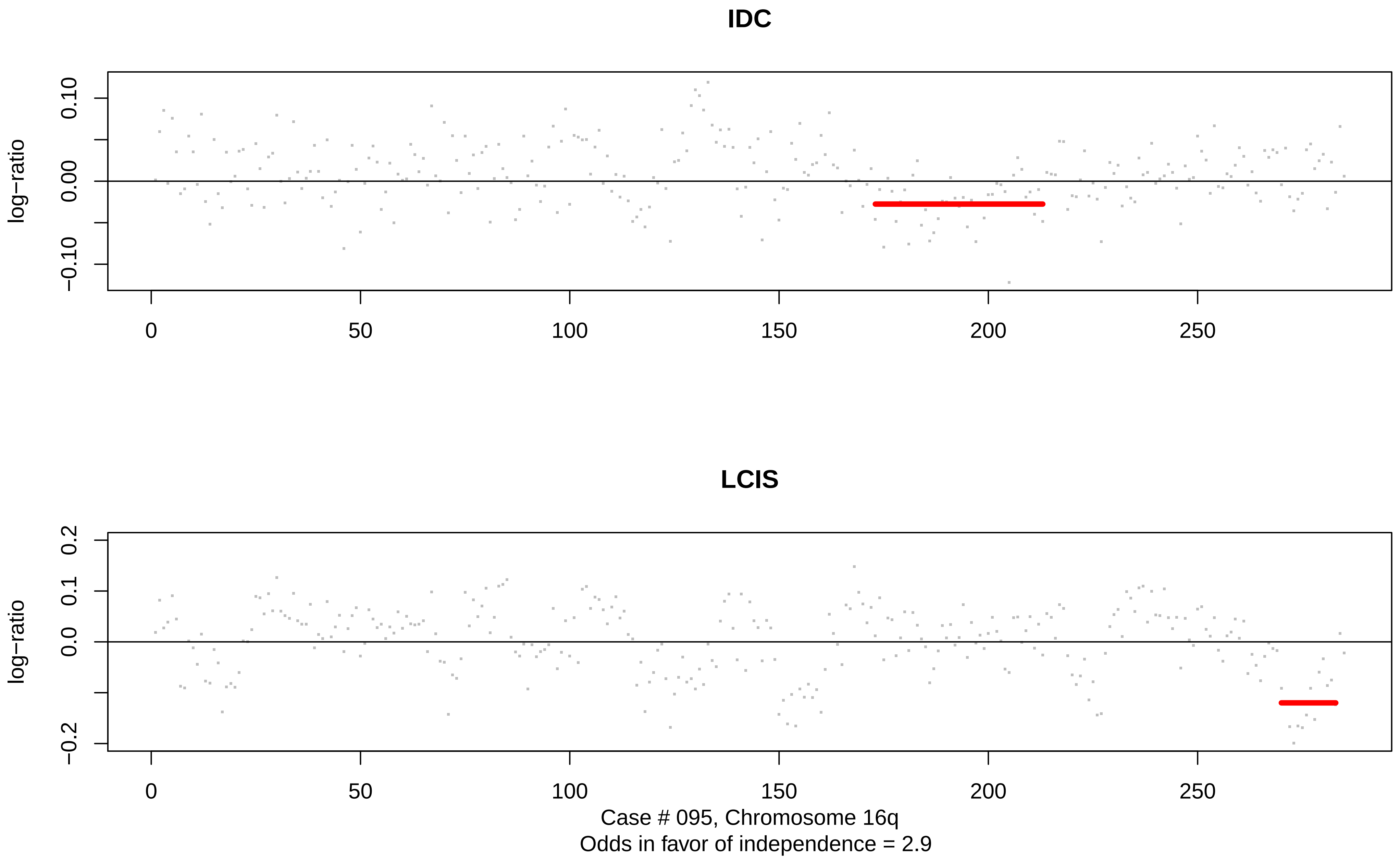
IDC
log−ratio
0.10
0.00
−0.10
0
50
100
150
200
250
LCIS
log−ratio
0.2
0.1
0.0
−0.2
0
50
100
150
200
250
Case # 095, Chromosome 16q
Odds in favor of independence = 2.9

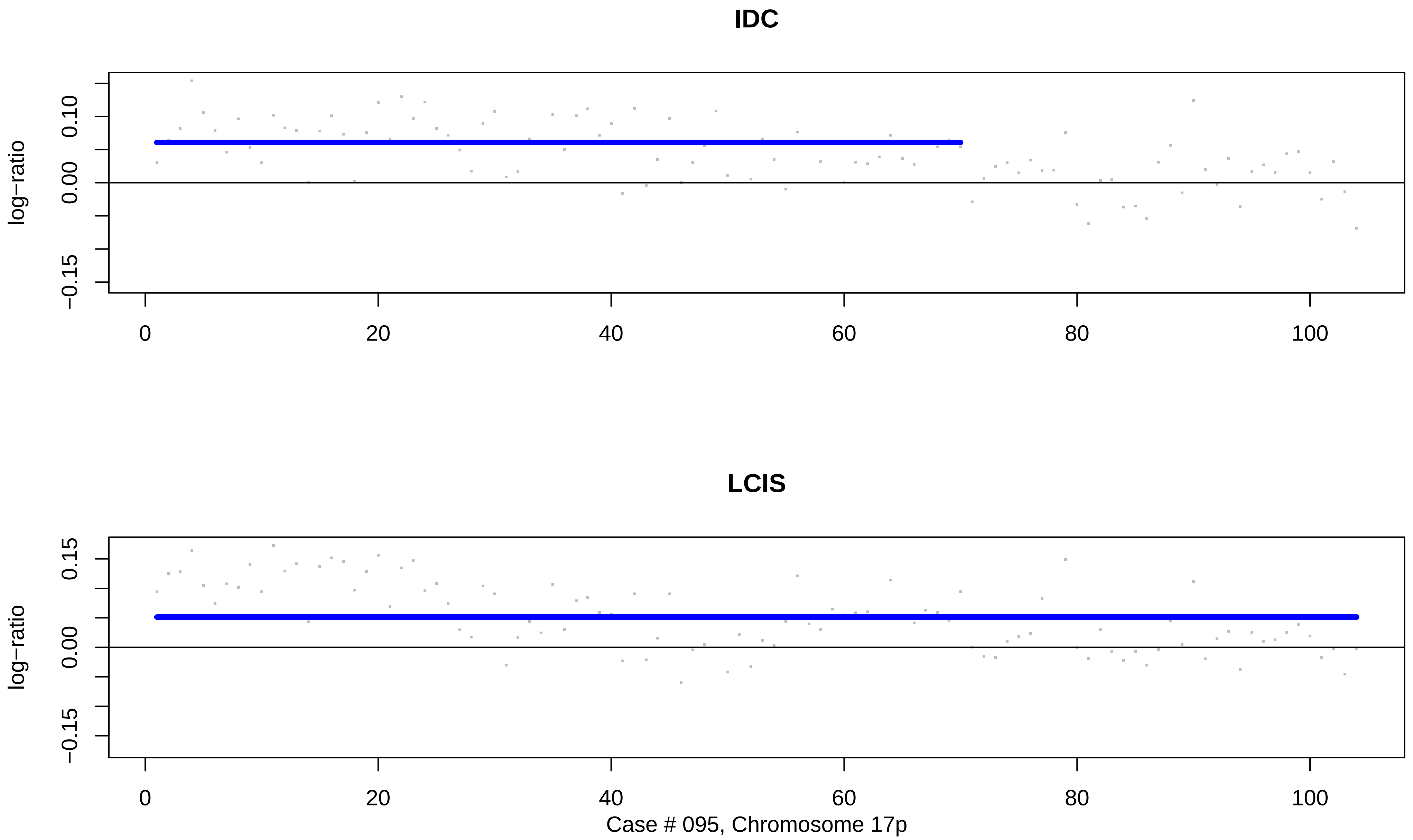
IDC
log−ratio
−0.15
0.00
0.10
0
20
40
60
80
100
LCIS
log−ratio
−0.15
0.00
0.15
0
20
40
60
80
100
Case # 095, Chromosome 17p

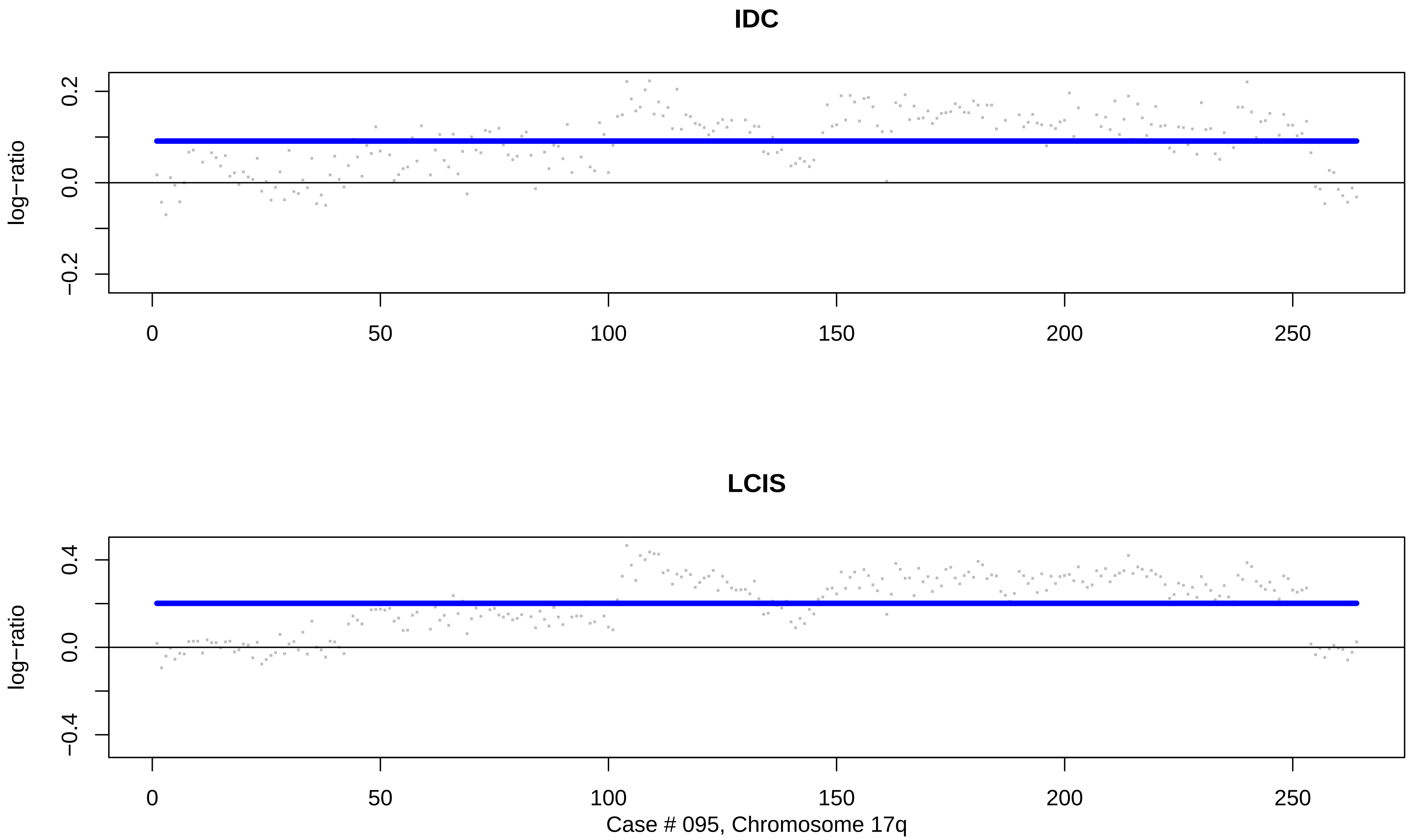
IDC
log−ratio
−0.2
0.0
0.2
0
50
100
150
200
250
LCIS
log−ratio
−0.4
0.0
0.4
0
50
100
150
200
250
Case # 095, Chromosome 17q

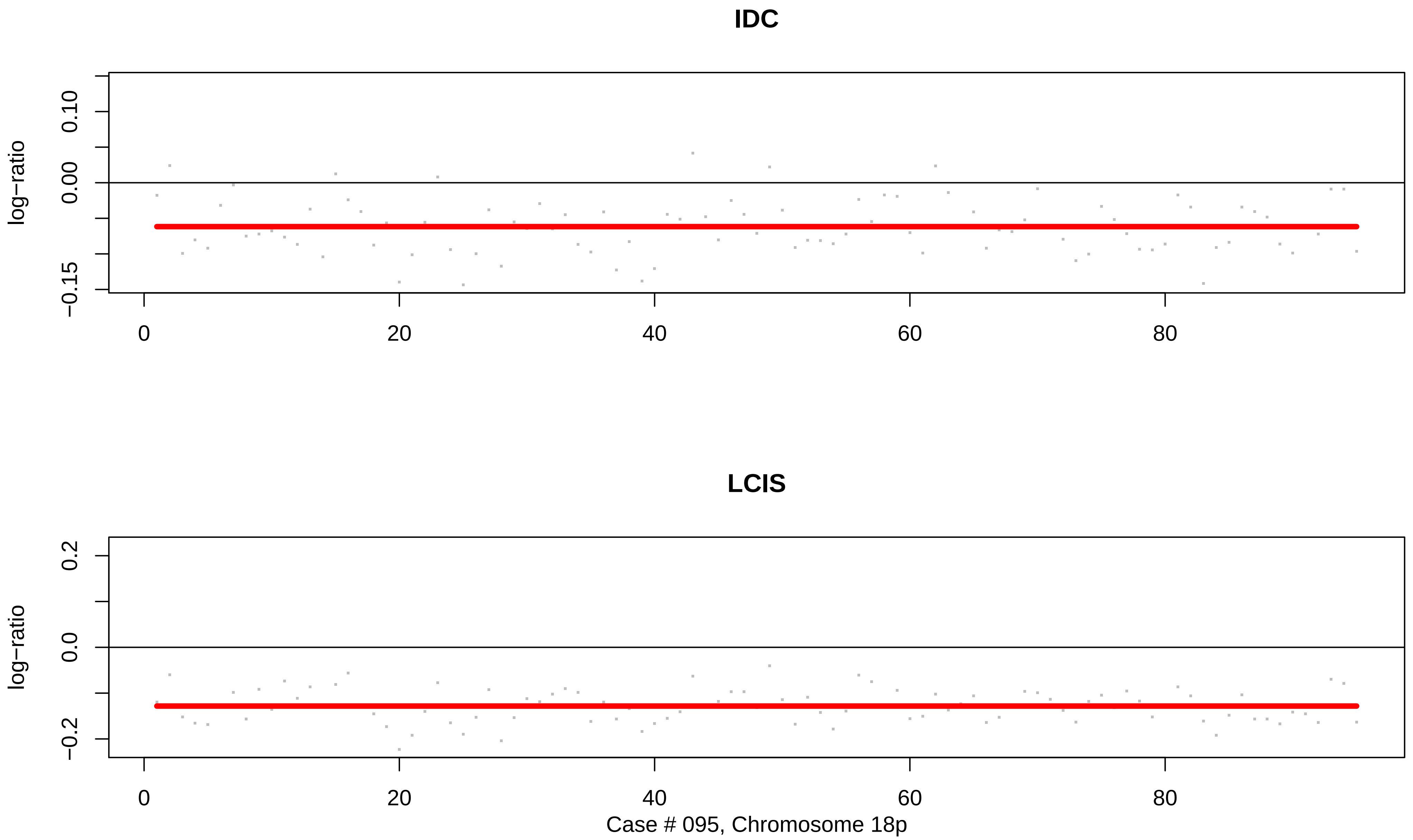
IDC
log−ratio
0.10
0.00
−0.15
0
20
40
60
80
LCIS
log−ratio
0.2
0.0
−0.2
0
20
40
60
80
Case # 095, Chromosome 18p

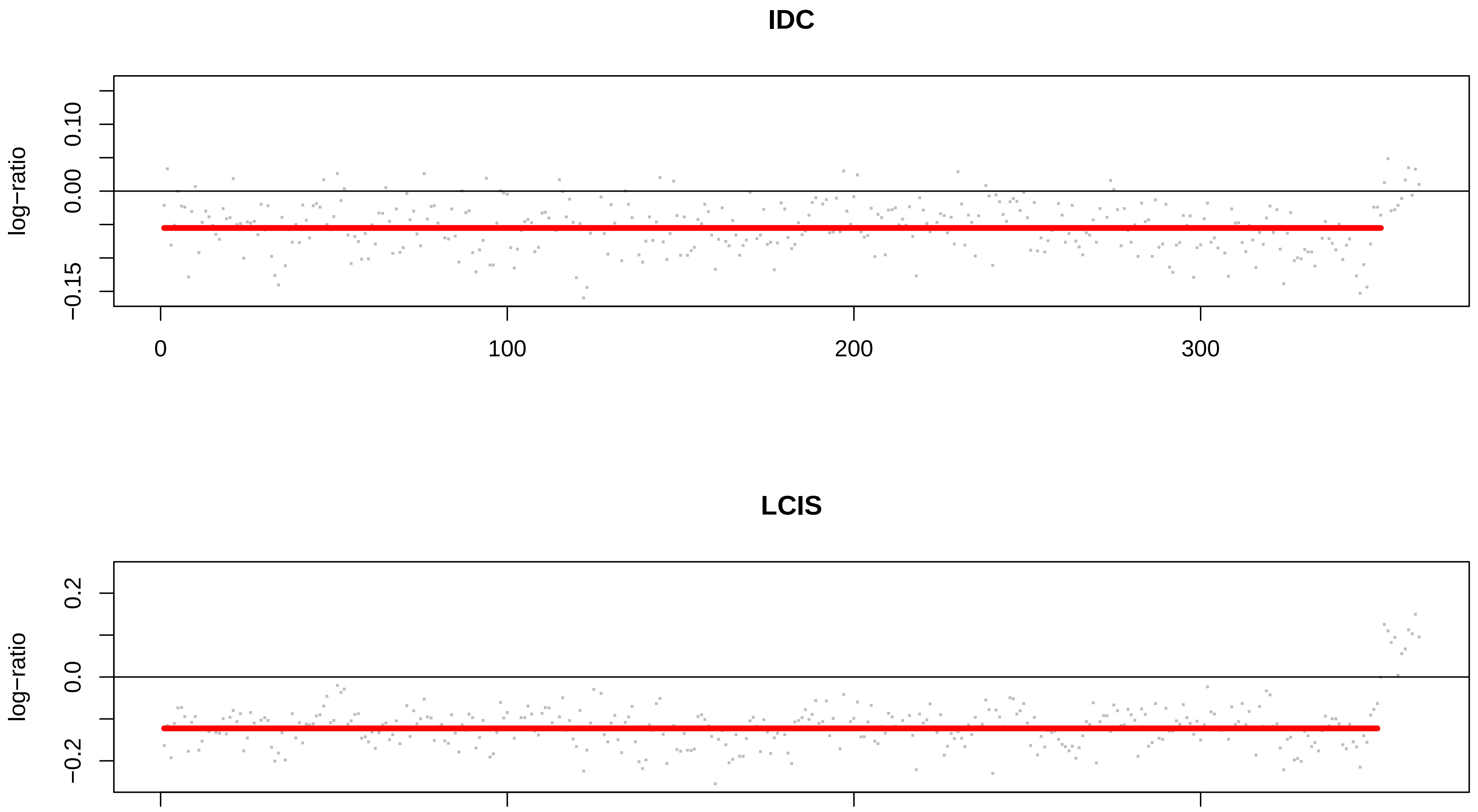

Case # 095, Chromosome 18q
Odds in favor of clonality = 33.2

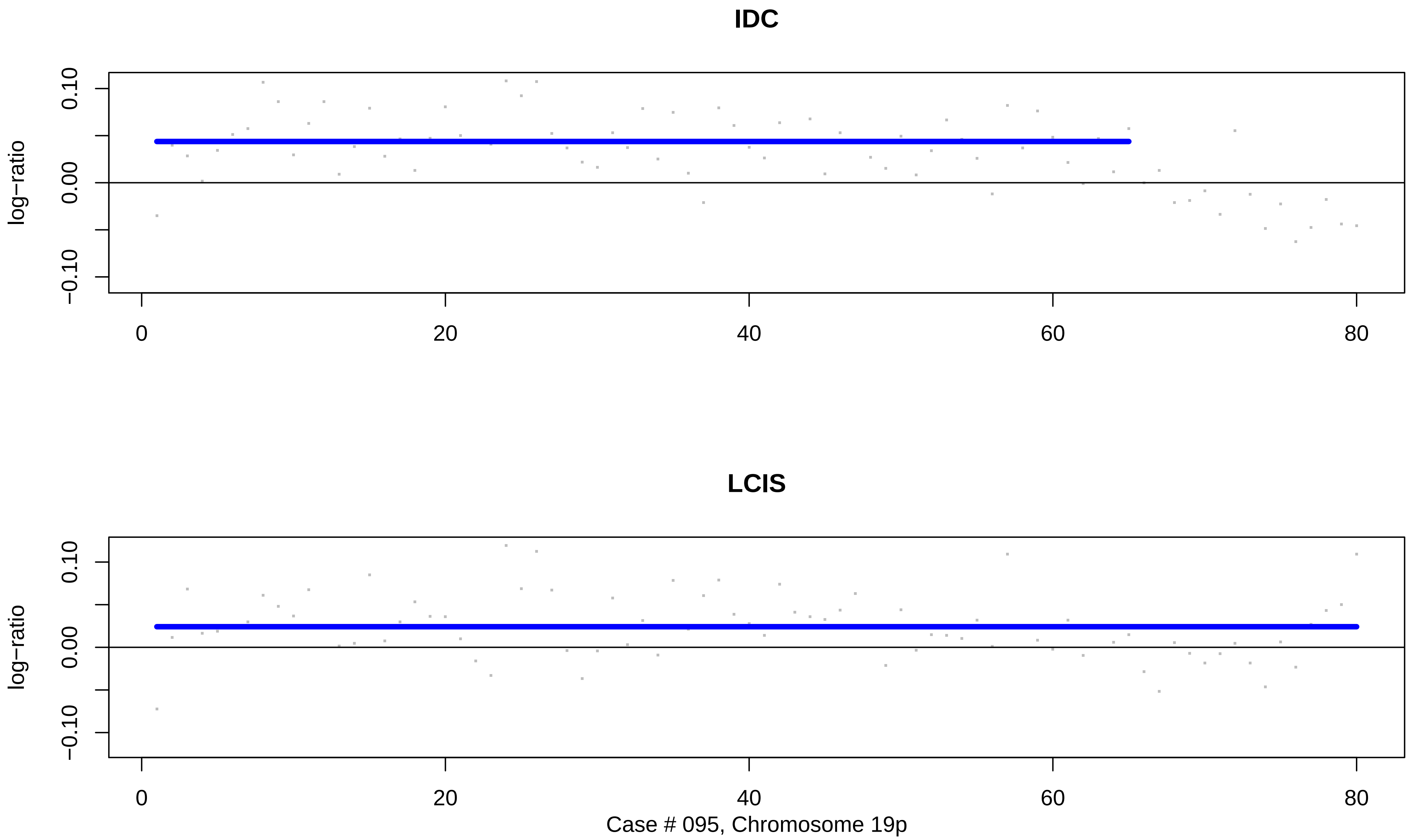
IDC
LCIS
log−ratio
0.10
0.00
−0.10
0
20
40
60
80
Case # 095, Chromosome 19p

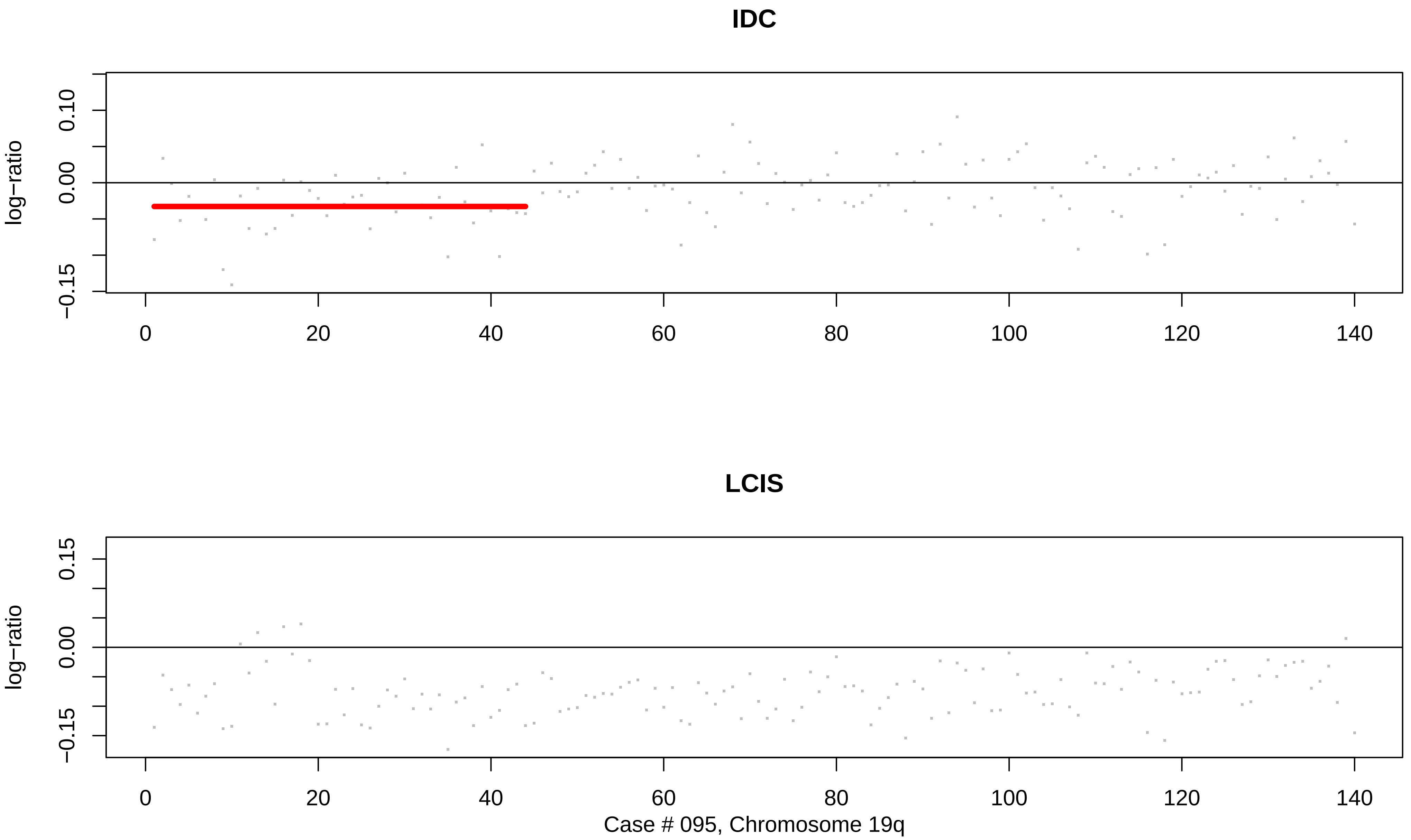
IDC
log−ratio
−0.15
0.00
0.10
0
20
40
60
80
100
120
140
LCIS
log−ratio
−0.15
0.00
0.15
Case # 095, Chromosome 19q

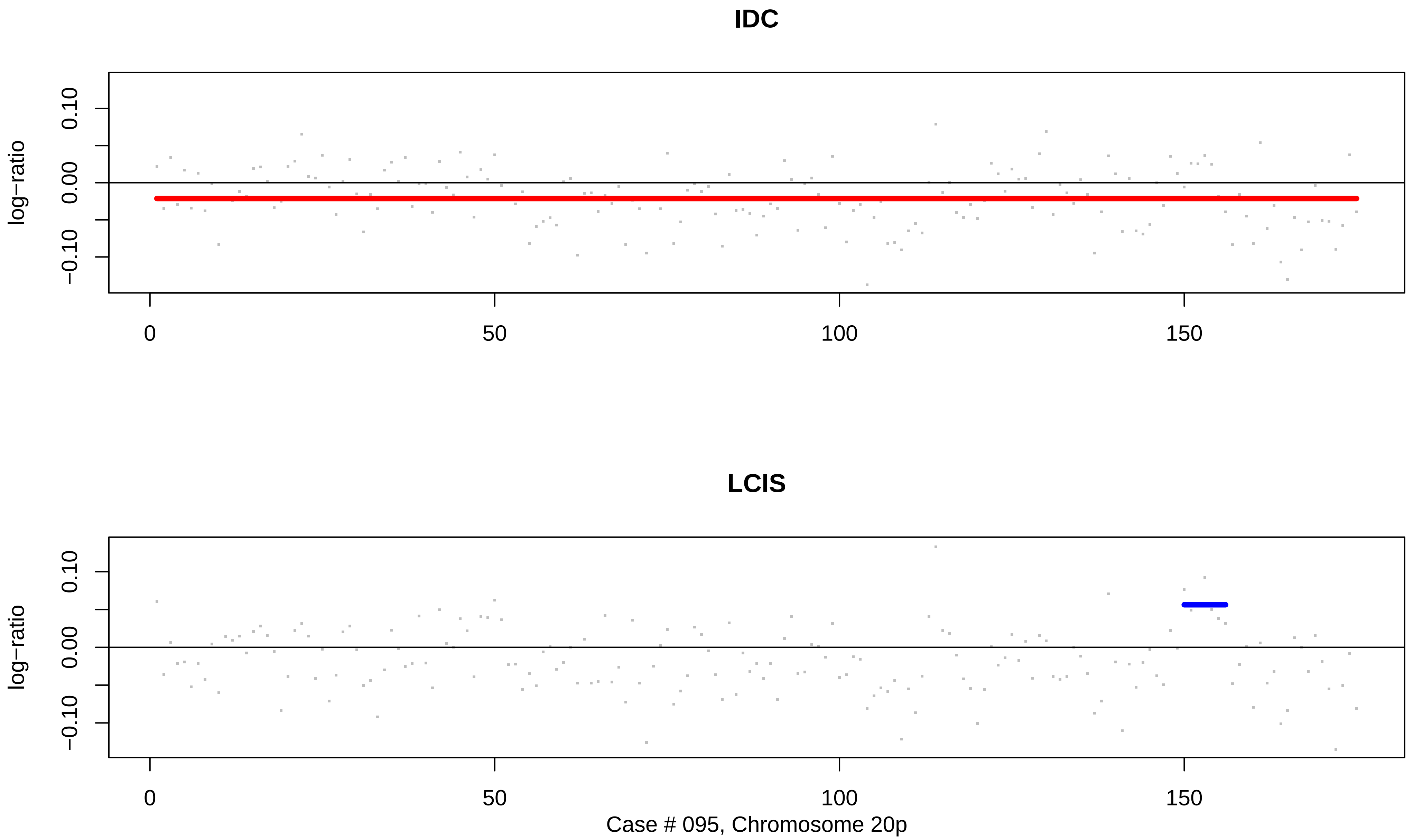
IDC
log−ratio
0.10
0.00
−0.10
0
50
100
150
LCIS
log−ratio
0.10
0.00
−0.10
0
50
100
150
Case # 095, Chromosome 20p

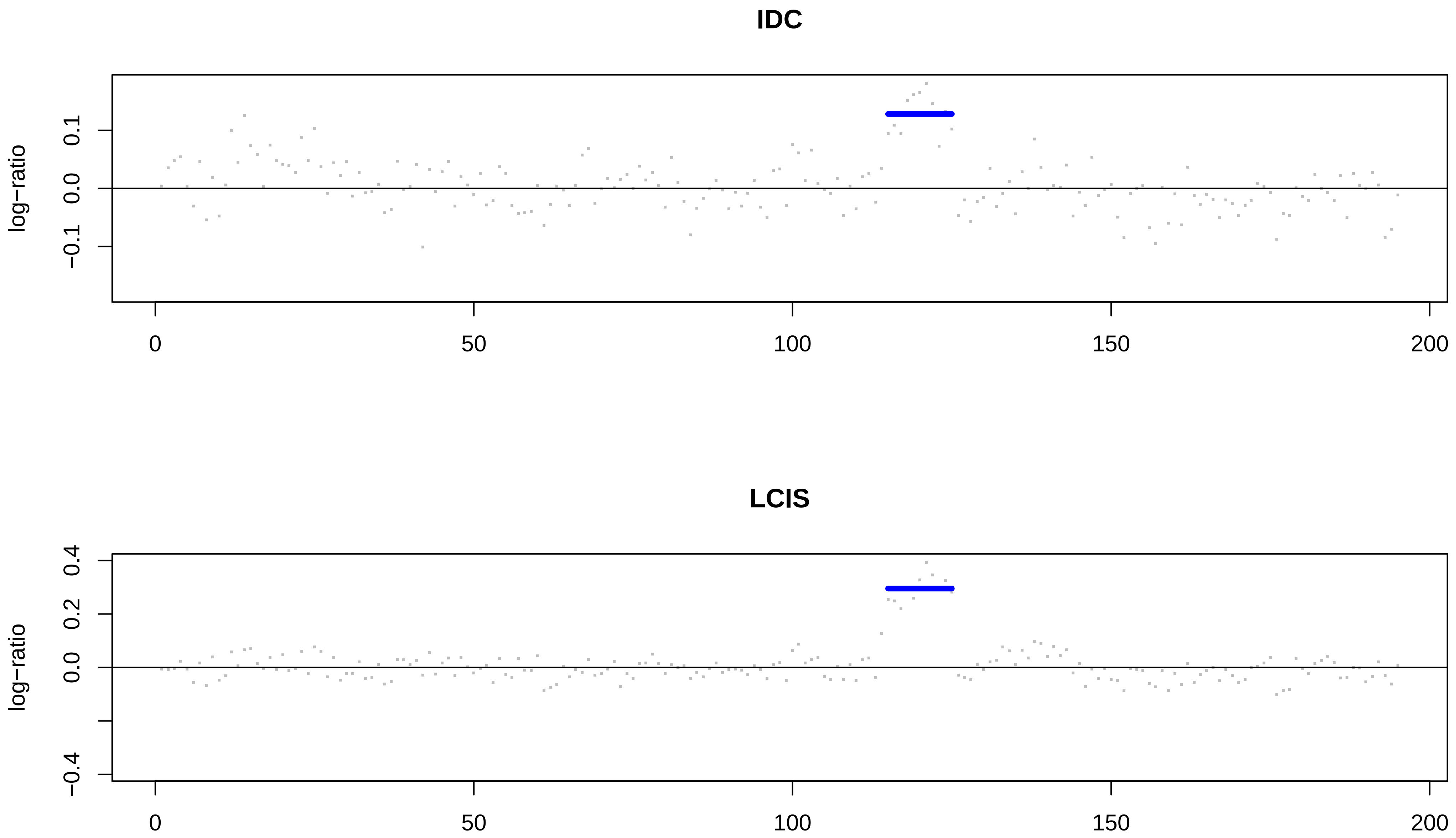

Case # 095, Chromosome 20q
Odds in favor of clonality = 1.6e+03

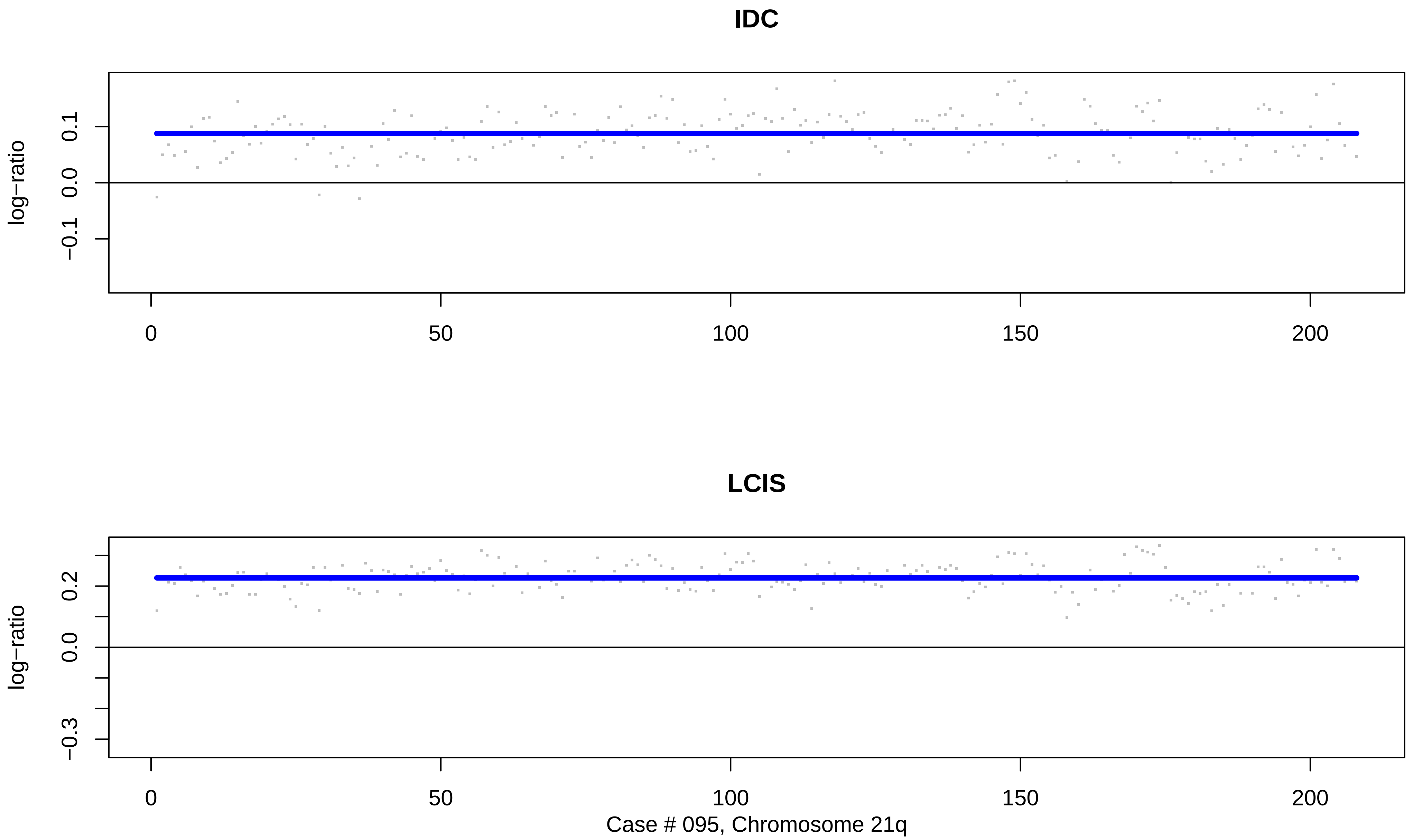
IDC
log−ratio
0.1
0.0
−0.1
0
50
100
150
200
LCIS
log−ratio
0.2
0.0
−0.3
0
50
100
150
200
Case # 095, Chromosome 21q

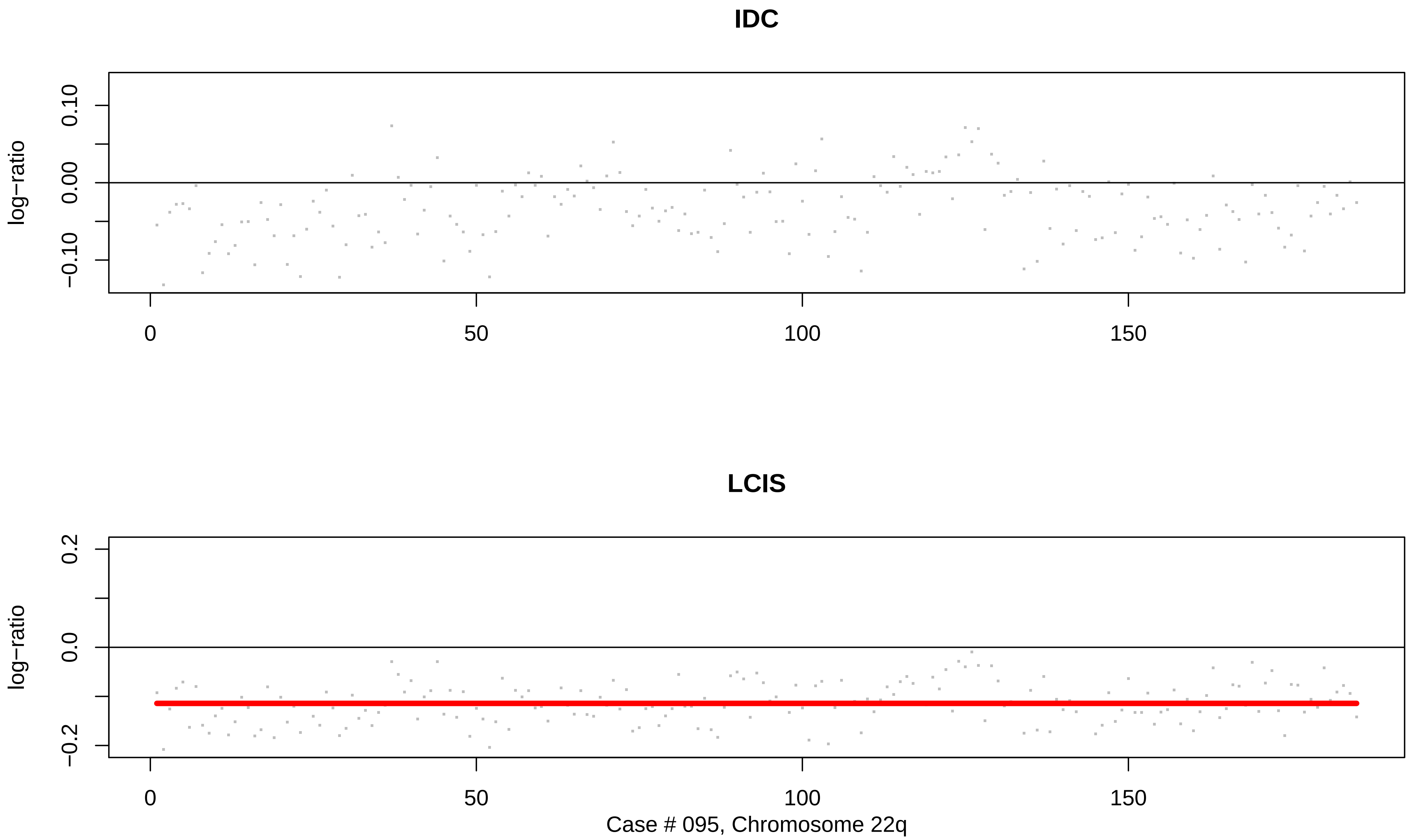

IDC
log−ratio
0.10
0.00
−0.10
0
50
100
150
LCIS
log−ratio
0.2
0.0
−0.2
0
50
100
150
Case # 095, Chromosome 22q
