# Supplementary material for: Clonal relatedness between lobular carcinoma in situ and synchronous malignant lesions
Source: Breast Cancer Res. 2012 Jul 9;14(4):R103. doi: 10.1186/bcr3222 (PMC3680923; doi:10.1186/bcr3222)
Supplement: Additional file 4 — Magnified version of genome-wide plots with detailed marker plots and segmentation on a chromosome-arm-specific basis. [file bcr3222-S4.ZIP › Case 093.pdf]

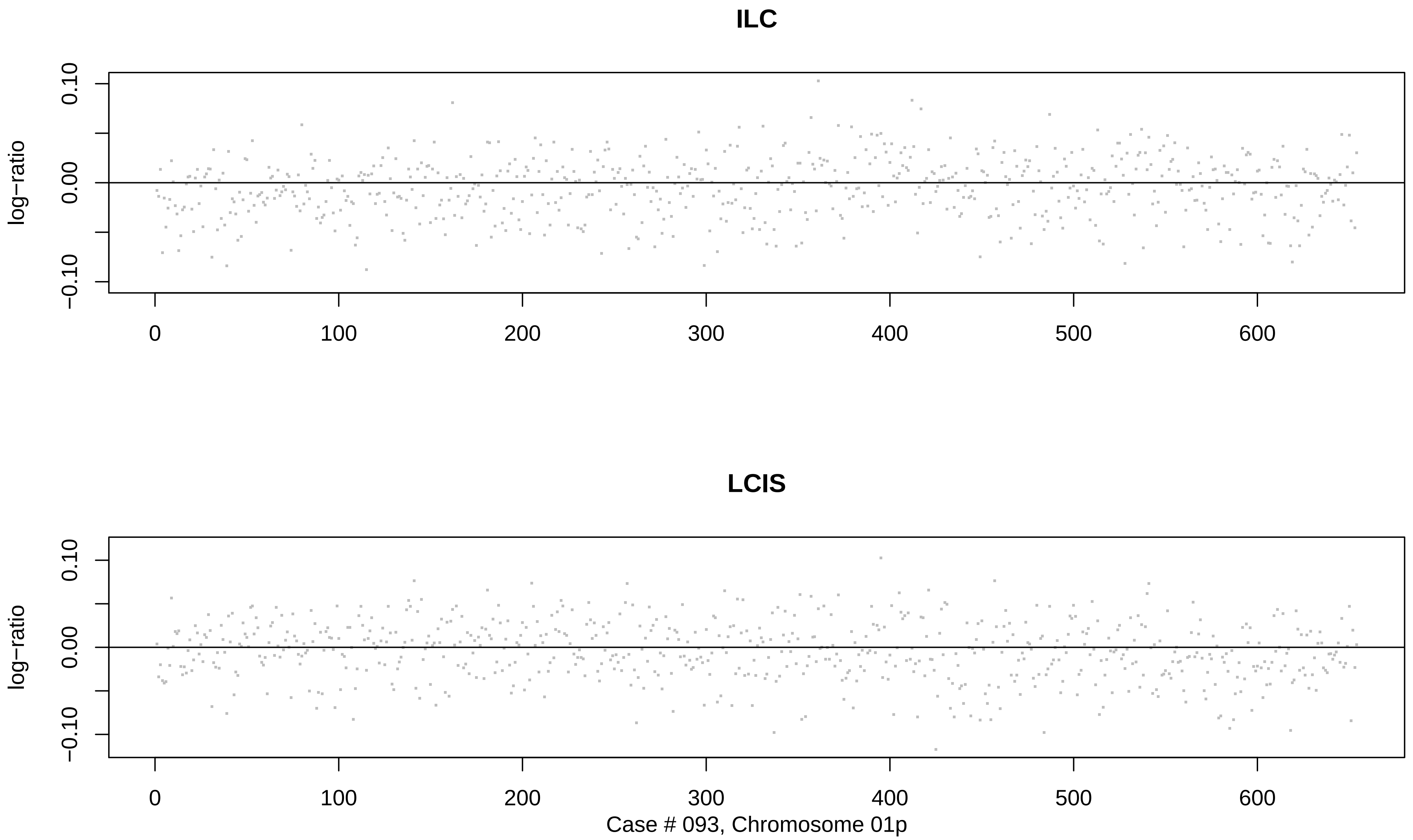
ILC
log−ratio
0.10
0.00
−0.10
0
100
200
300
400
500
600
LCIS
log−ratio
0.10
0.00
−0.10
0
100
200
300
400
500
600
Case # 093, Chromosome 01p

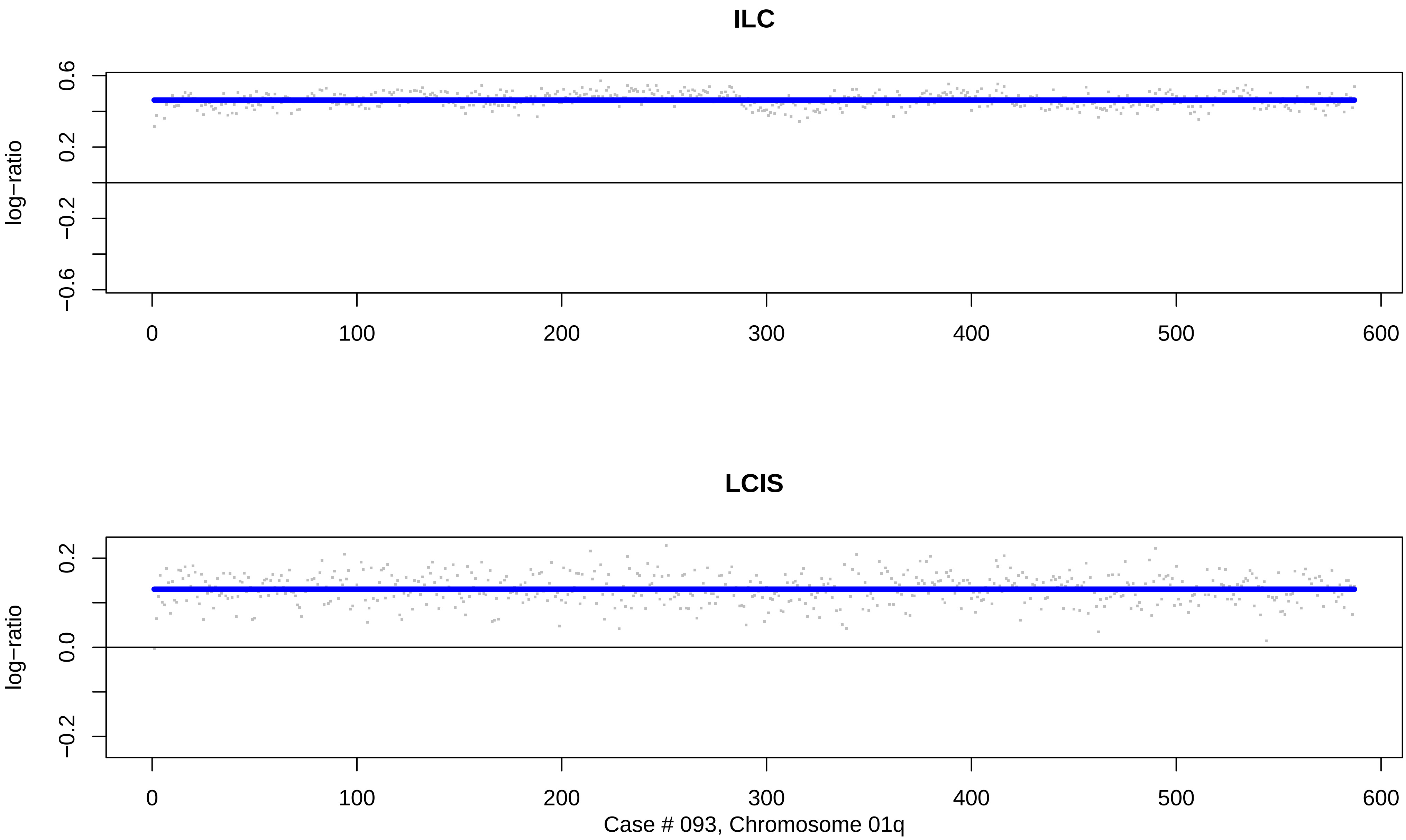
ILC
log−ratio
0.6
0.2
−0.2
−0.6
0
100
200
300
400
500
600
LCIS
log−ratio
0.2
0.0
−0.2
0
100
200
300
400
500
600
Case # 093, Chromosome 01q

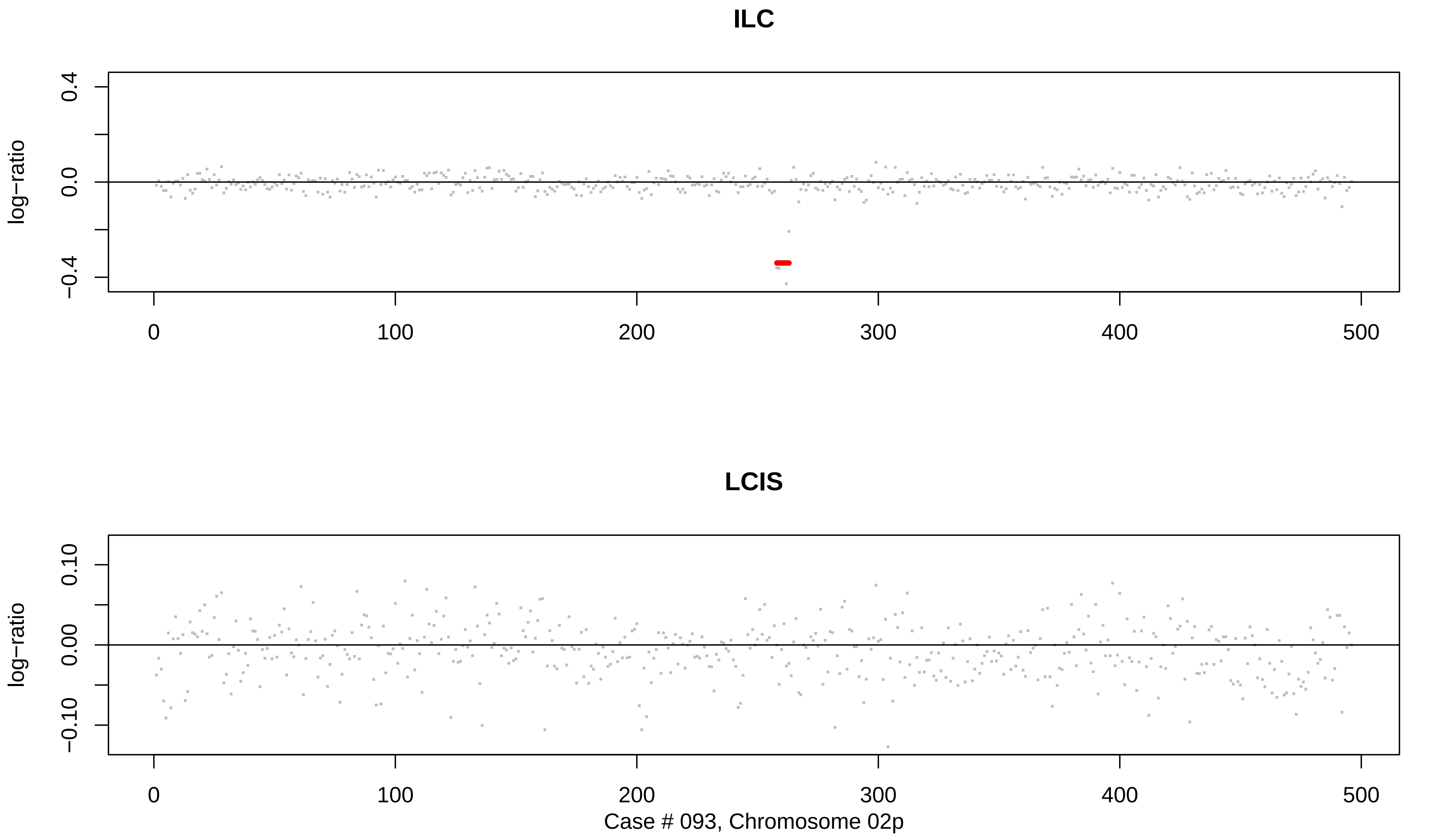
ILC
log−ratio
0.4
0.0
−0.4
0
100
200
300
400
500
LCIS
log−ratio
0.10
0.00
−0.10
0
100
200
300
400
500
Case # 093, Chromosome 02p

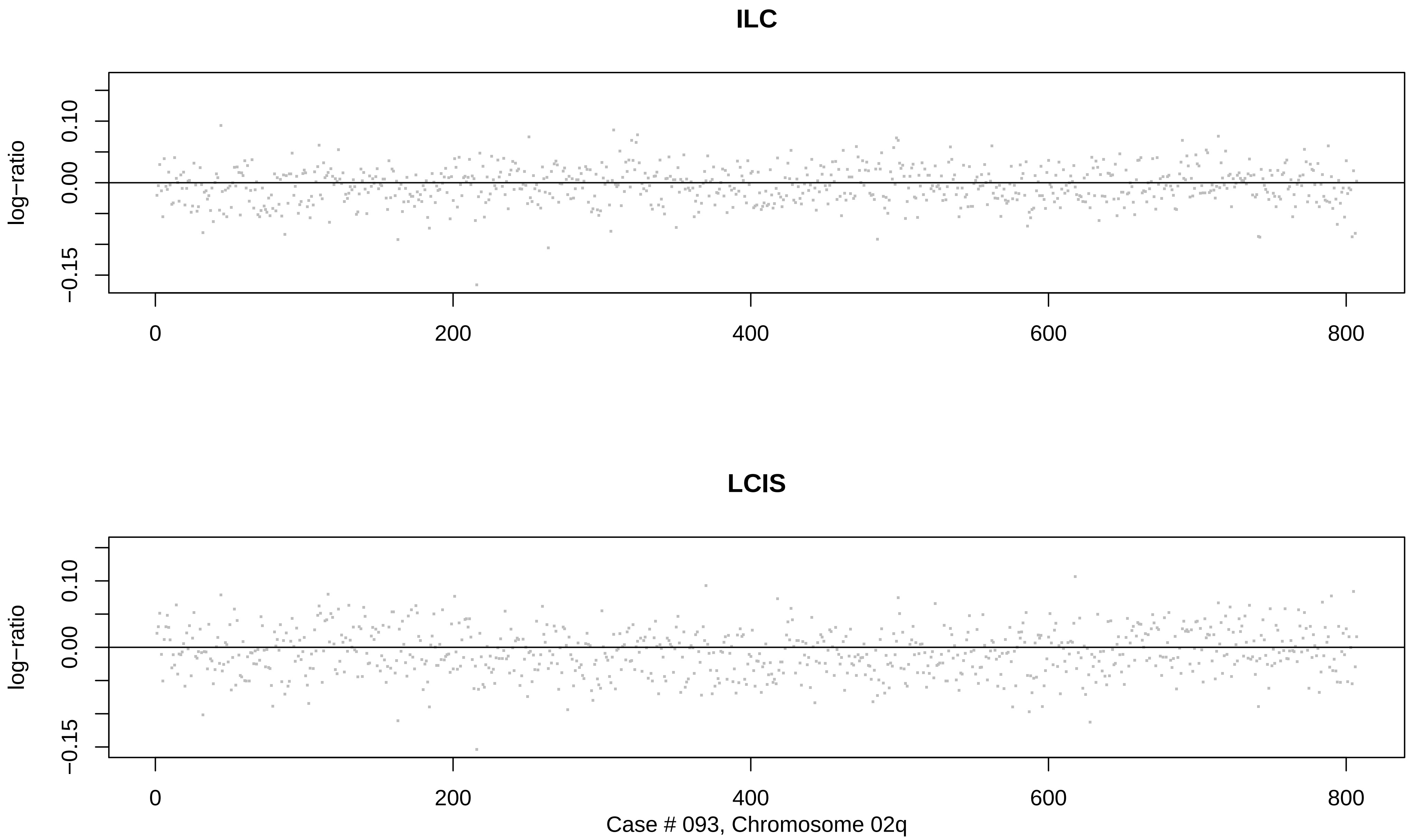
ILC
log−ratio
0.10
0.00
−0.15
0
200
400
600
800
LCIS
log−ratio
0.10
0.00
−0.15
0
200
400
600
800
Case # 093, Chromosome 02q

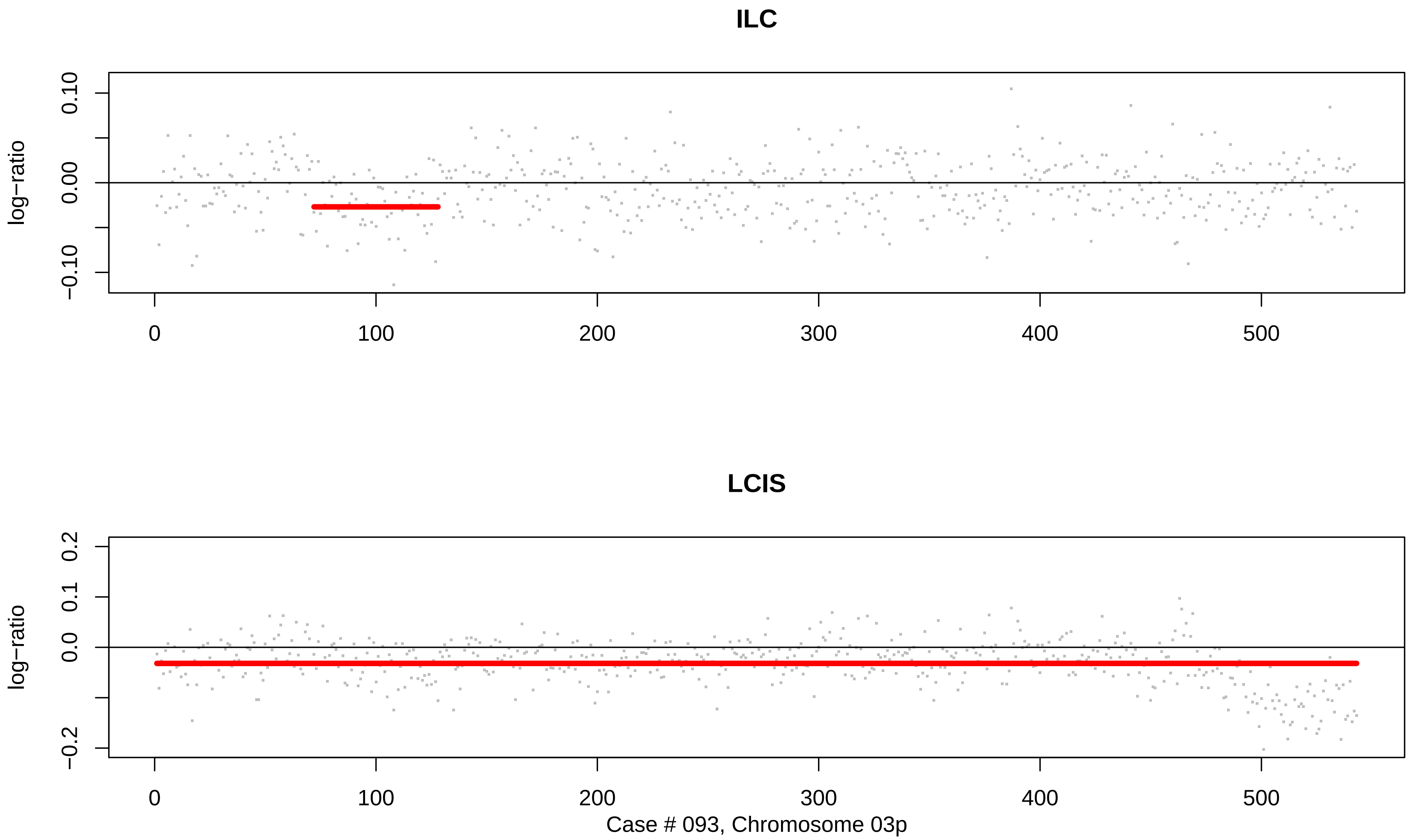

ILC
log−ratio
0.10
0.00
−0.10
0
100
200
300
400
500
LCIS
log−ratio
0.2
0.1
0.0
−0.2
0
100
200
300
400
500
Case # 093, Chromosome 03p

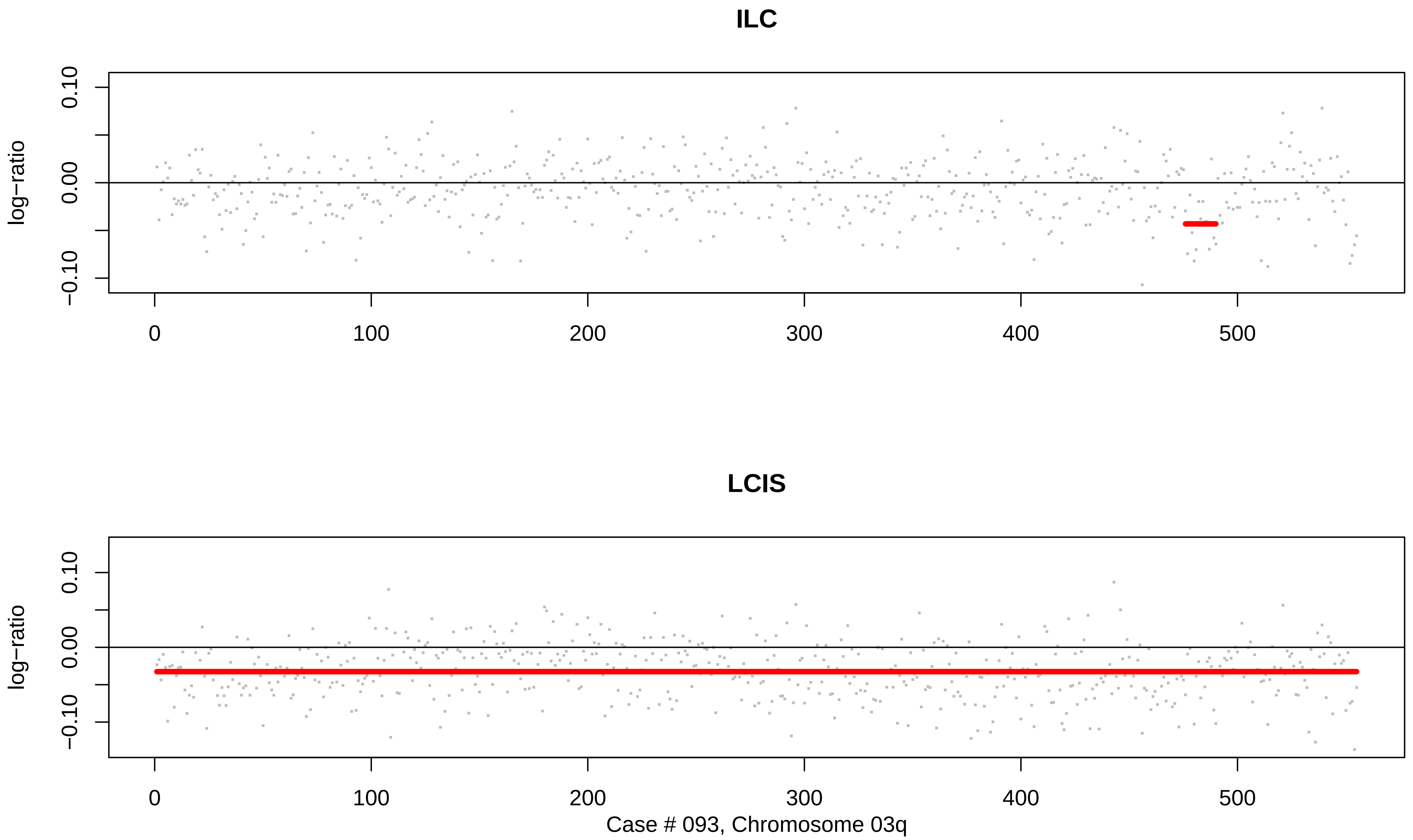
ILC
LCIS
log−ratio
0.10
0.00
−0.10
0
100
200
300
400
500
Case # 093, Chromosome 03q

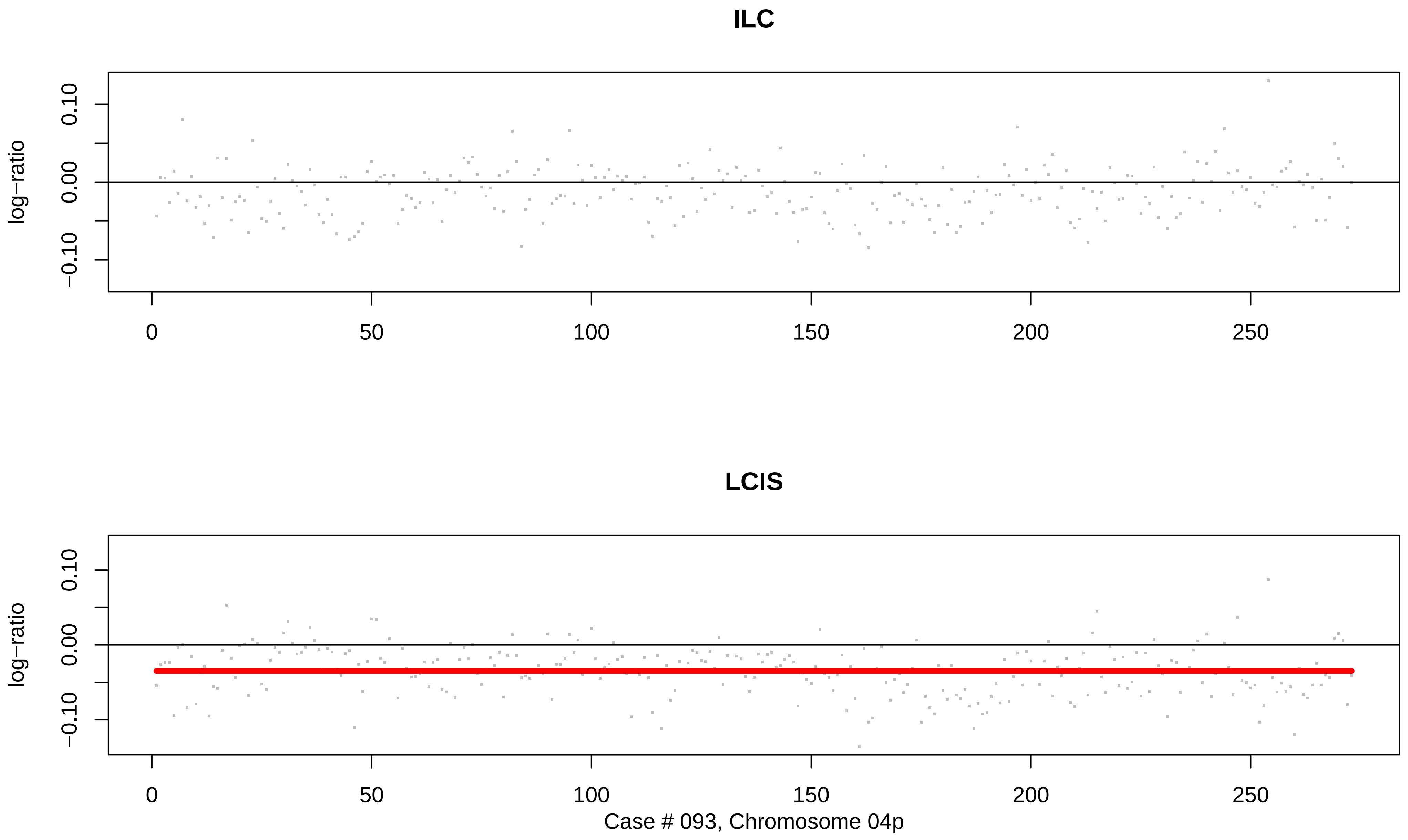
ILC
log−ratio
0.10
0.00
−0.10
0
50
100
150
200
250
LCIS
log−ratio
0.10
0.00
−0.10
0
50
100
150
200
250
Case # 093, Chromosome 04p

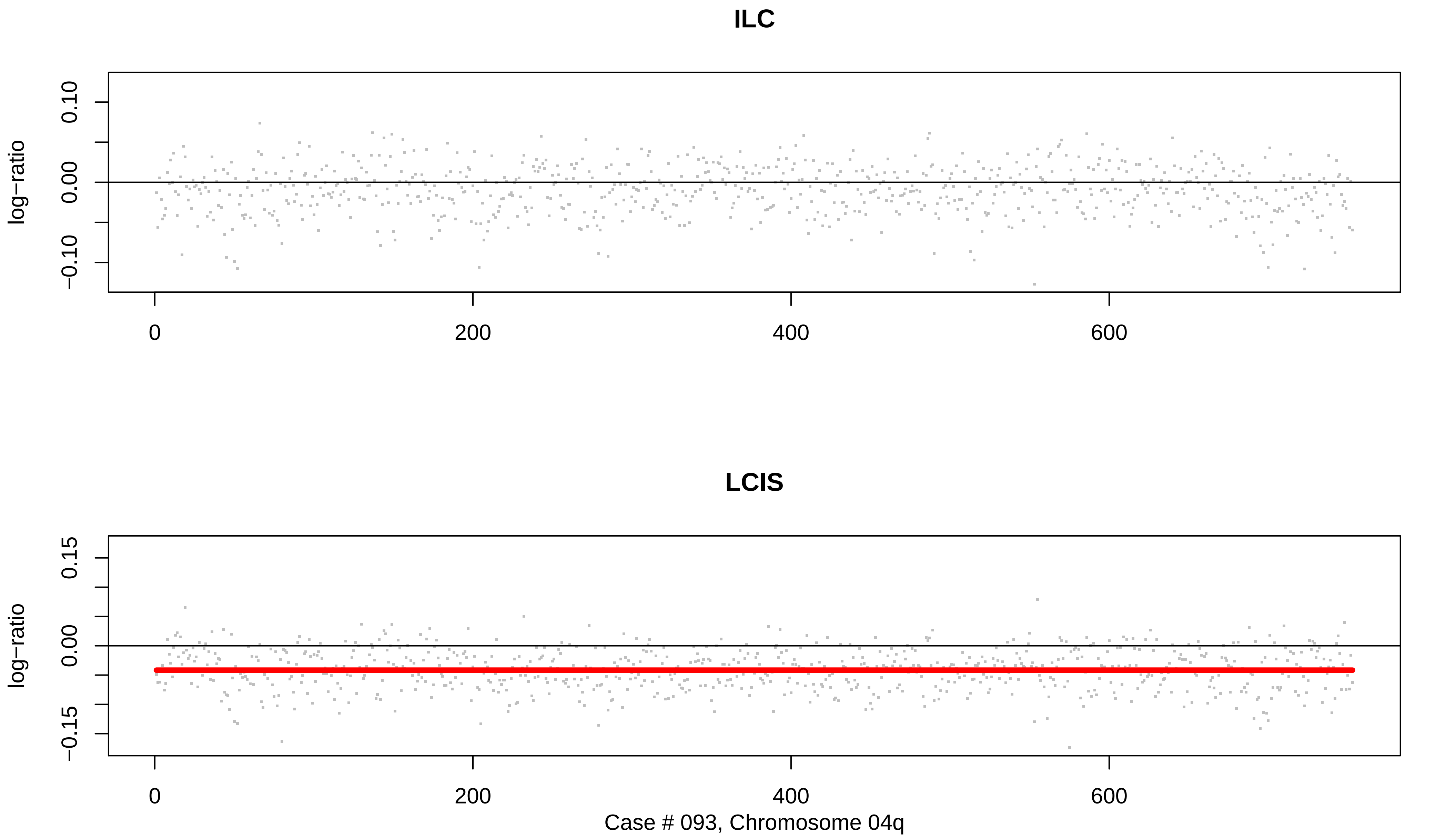

ILC
log−ratio
0.10
0.00
−0.10
0
200
400
600
LCIS
log−ratio
0.15
0.00
−0.15
0
200
400
600
Case # 093, Chromosome 04q

ILC

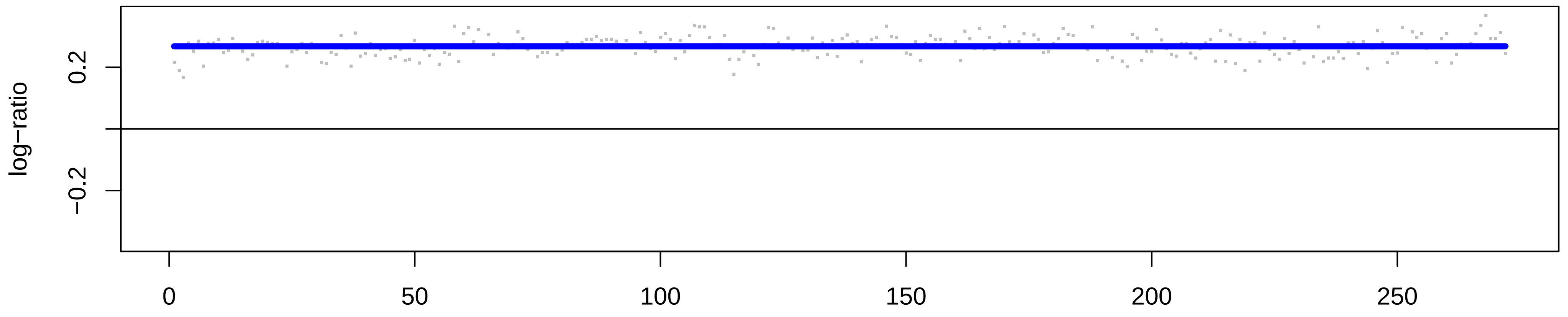

log−ratio
0.2
−0.2
0
50
100
150
200
250

LCIS

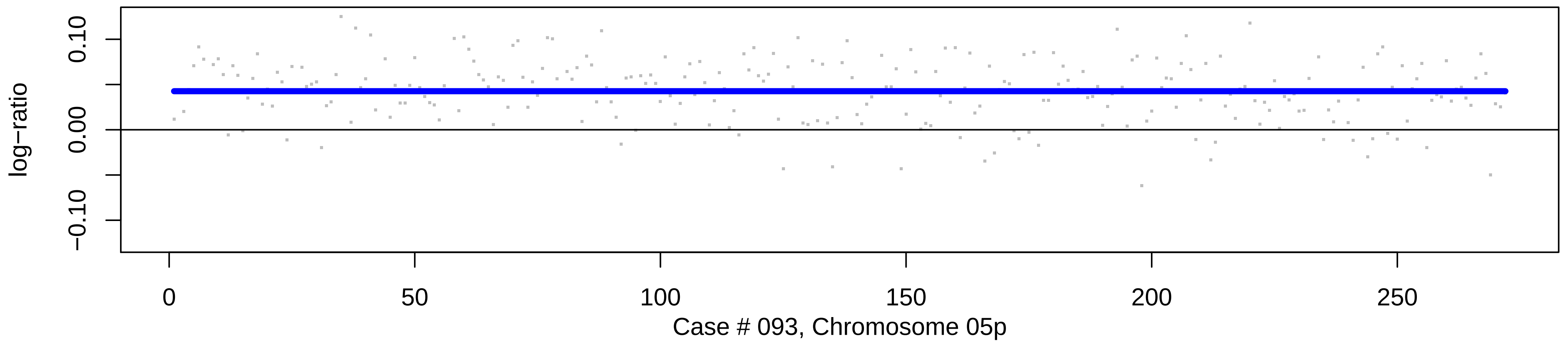

log−ratio
0.10
0.00
−0.10
0
50
100
150
200
250
Case # 093, Chromosome 05p

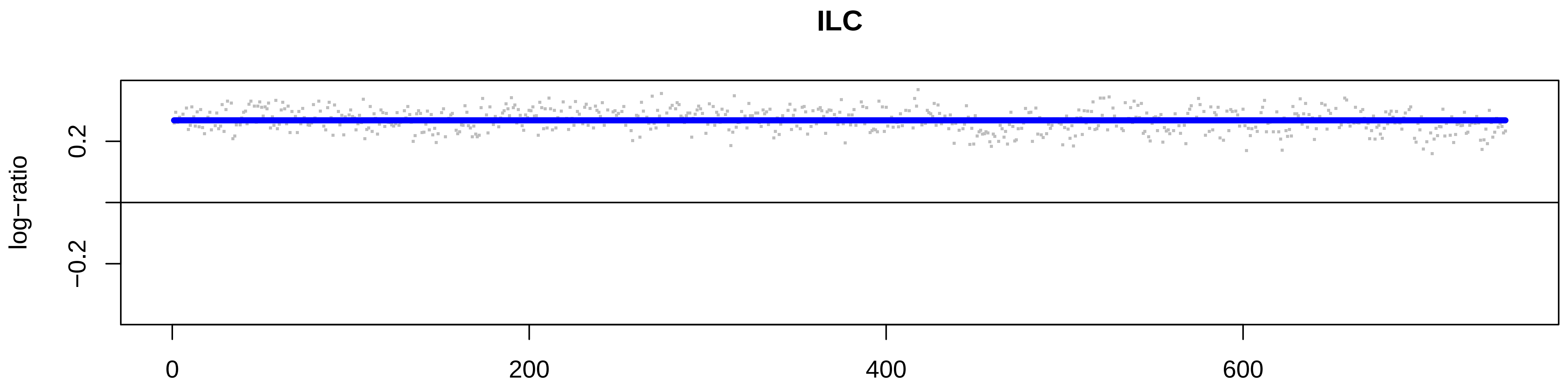

ILC
log−ratio
0.2
−0.2
0
200
400
600

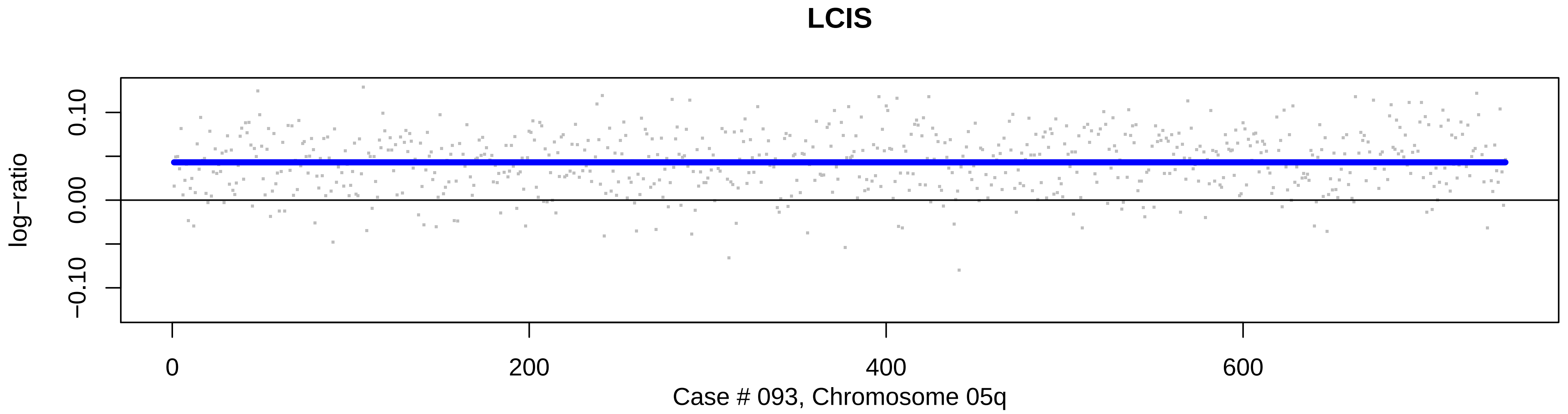

LCIS
log−ratio
0.10
0.00
−0.10
0
200
400
600
Case # 093, Chromosome 05q

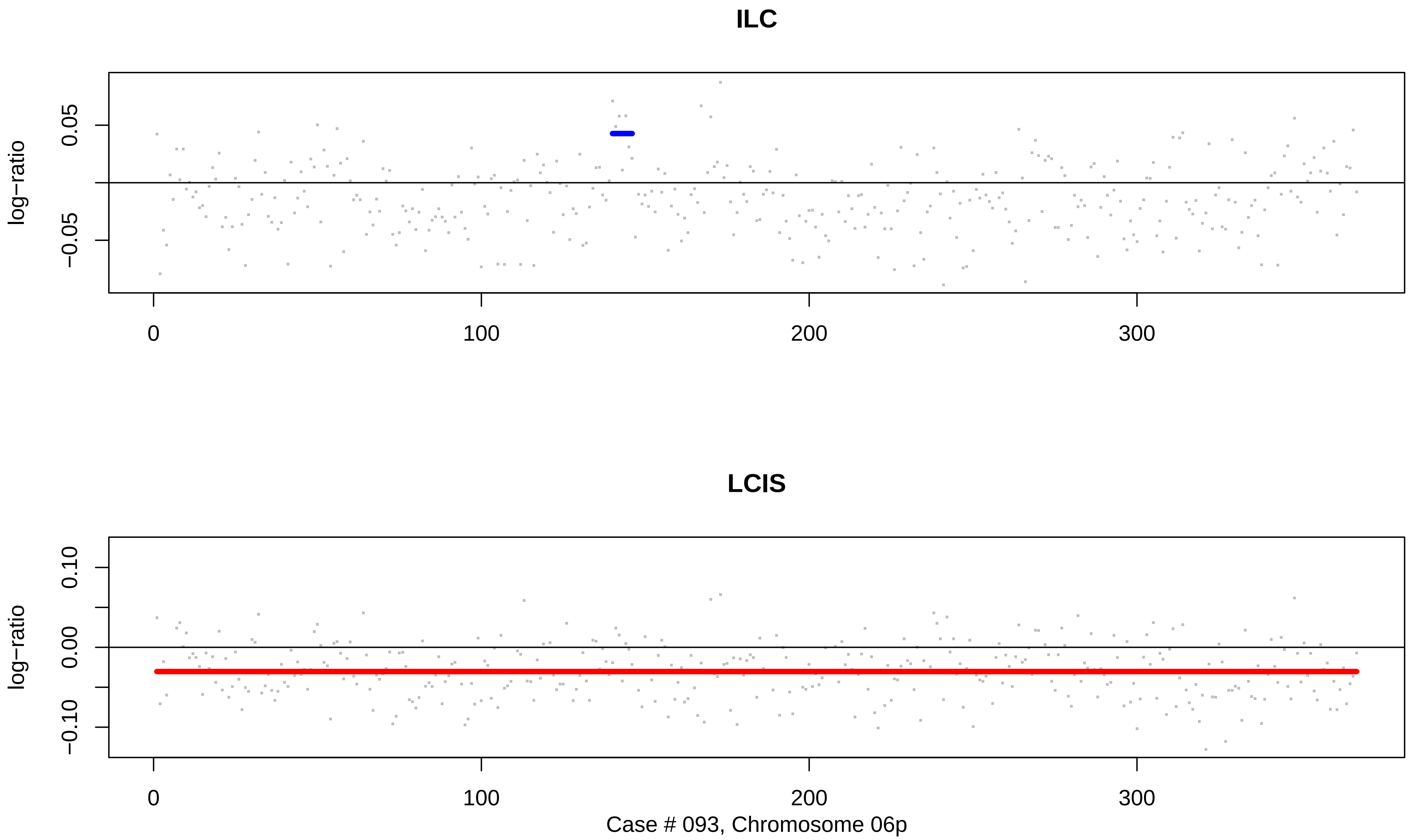
ILC
log−ratio
0.05
−0.05
0
100
200
300
LCIS
log−ratio
0.10
0.00
−0.10
0
100
200
300
Case # 093, Chromosome 06p

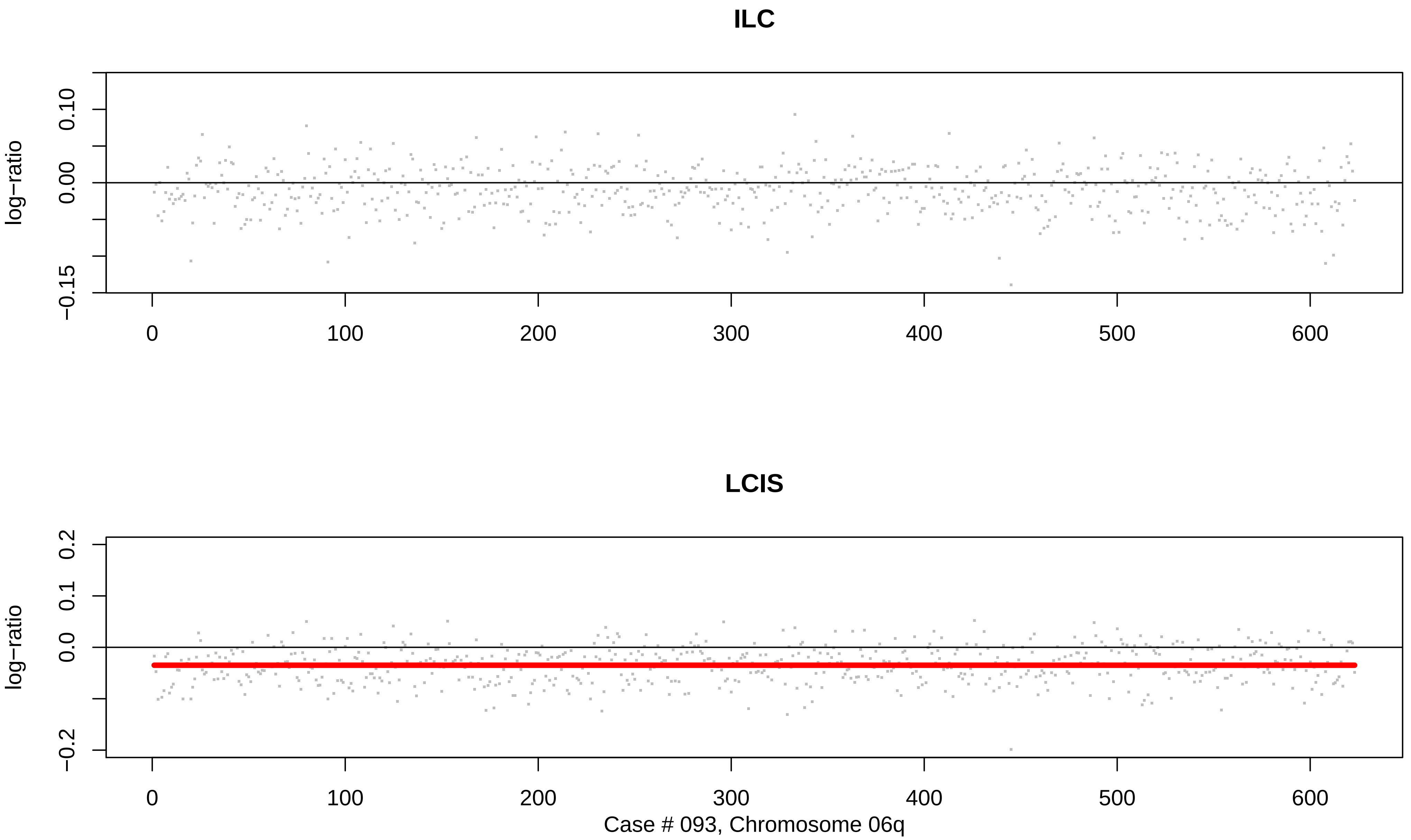

ILC
log−ratio
0.10
0.00
−0.15
0
100
200
300
400
500
600
LCIS
log−ratio
0.2
0.1
0.0
-0.2
0
100
200
300
400
500
600
Case # 093, Chromosome 06q

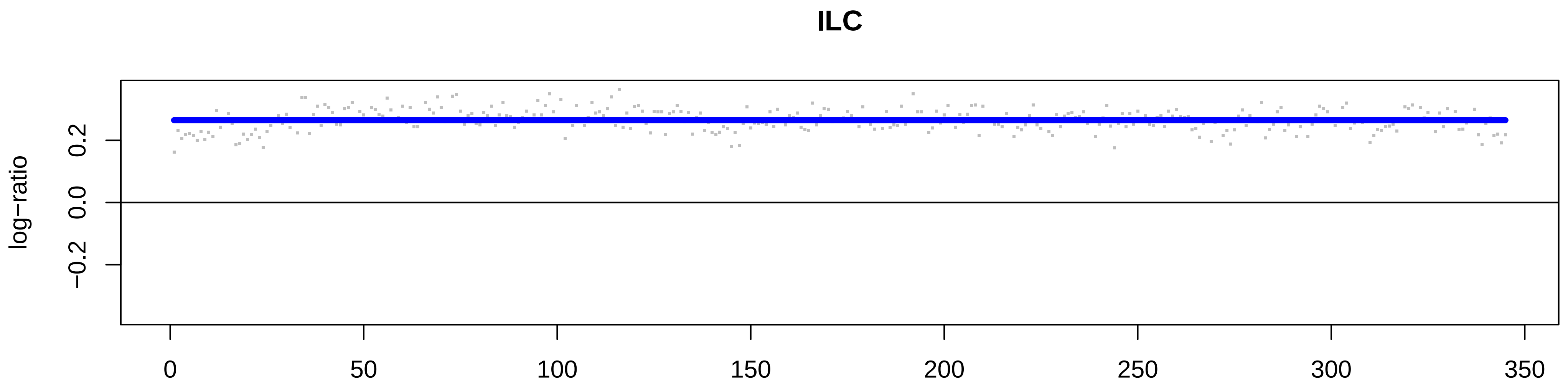
ILC
log−ratio
−0.2
0.0
0.2
0
50
100
150
200
250
300
350

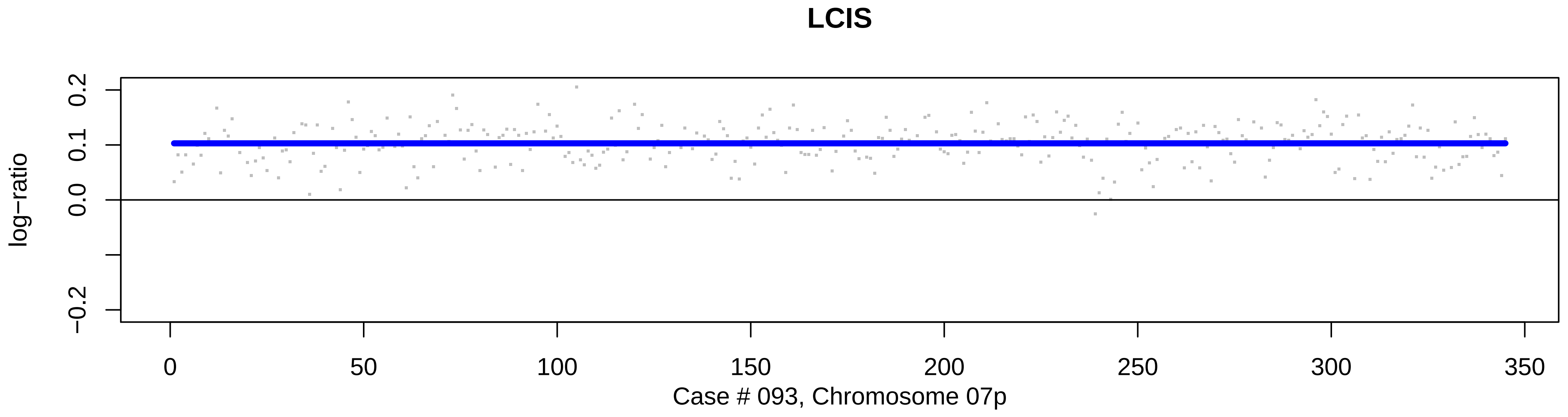
LCIS
log−ratio
−0.2
0.0
0.1
0.2
0
50
100
150
200
250
300
350
Case # 093, Chromosome 07p

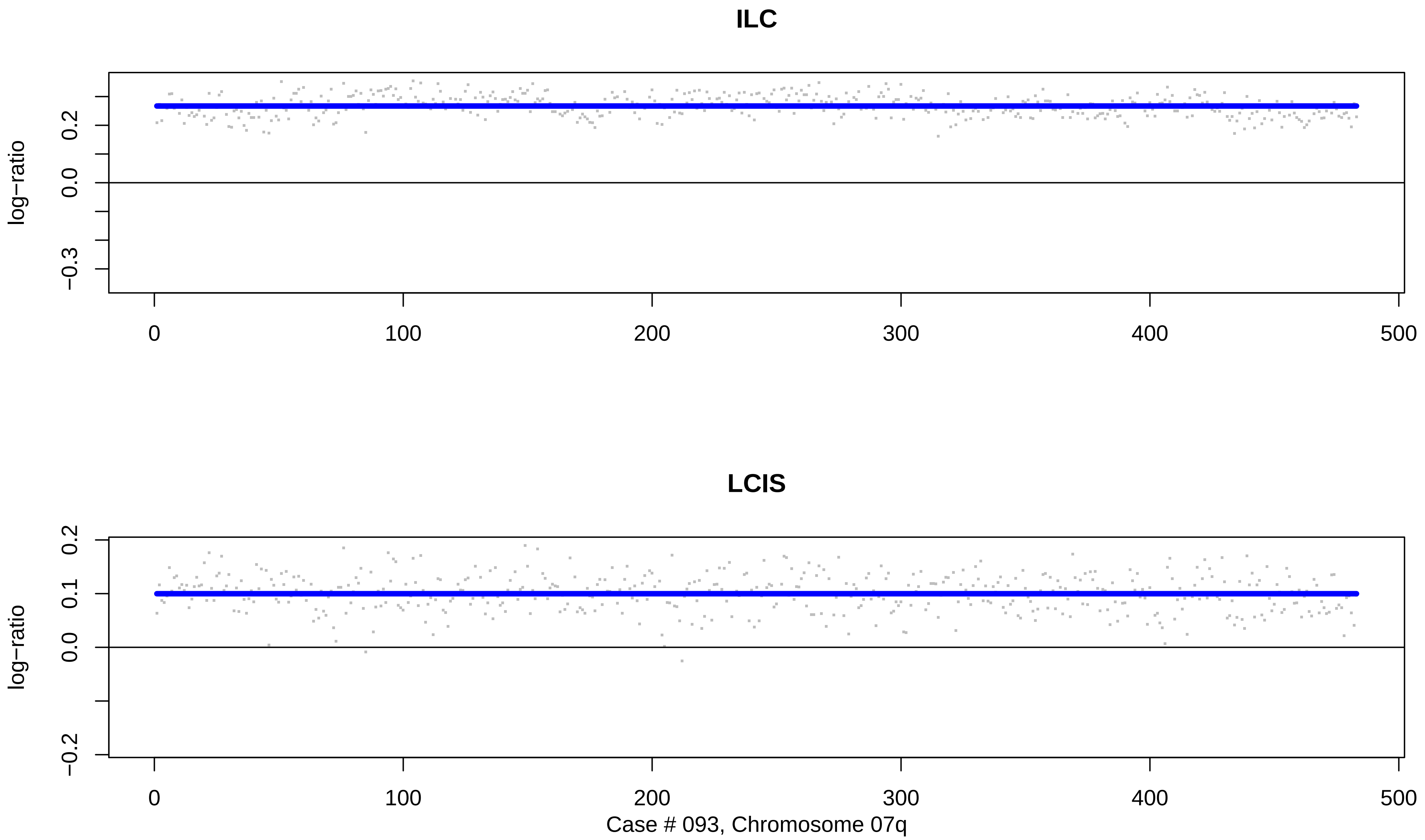

ILC
log−ratio
−0.3
0.0
0.2
0
100
200
300
400
500
LCIS
log−ratio
−0.2
0.0
0.1
0.2
0
100
200
300
400
500
Case # 093, Chromosome 07q

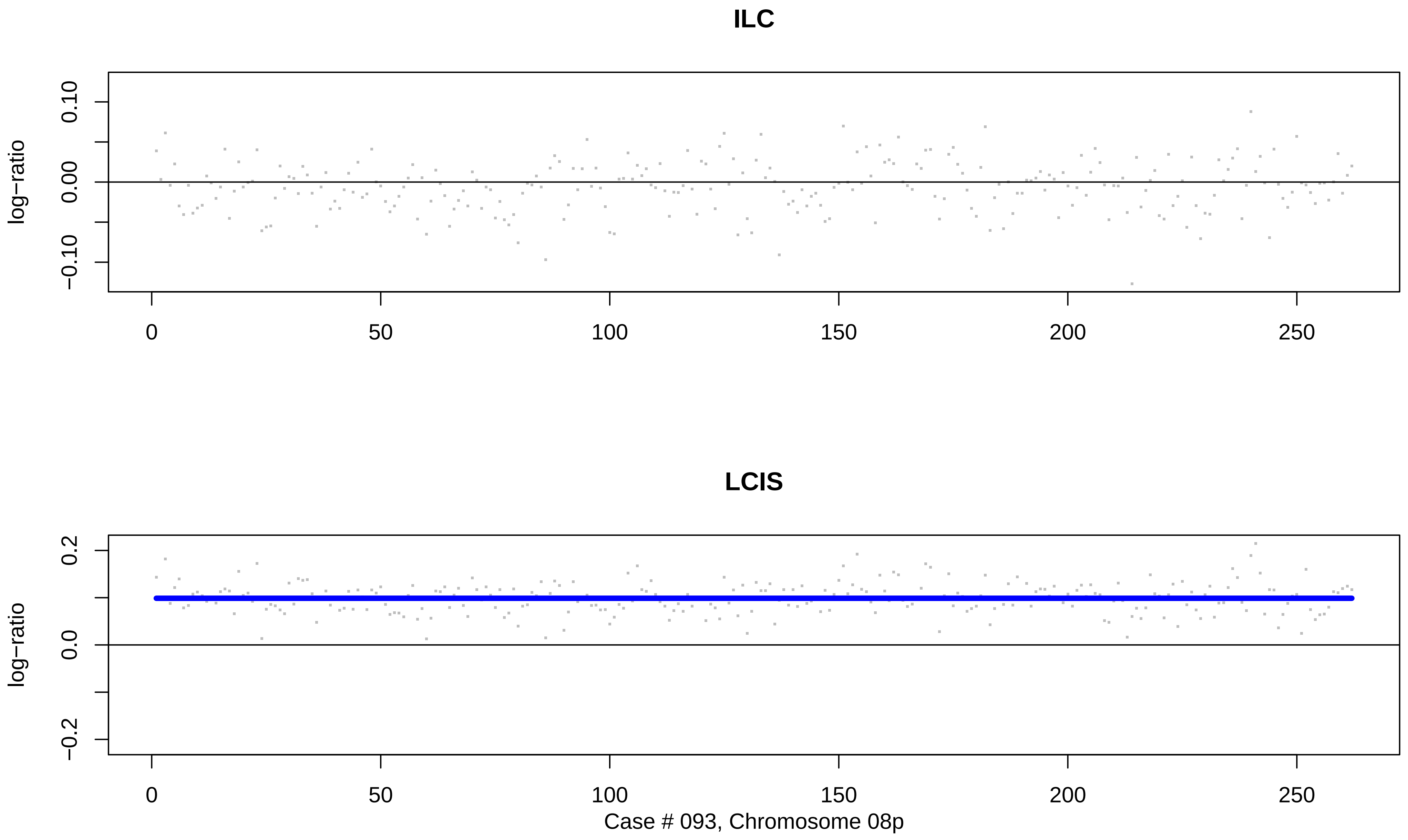
ILC
log−ratio
0.10
0.00
−0.10
0
50
100
150
200
250
LCIS
log−ratio
0.2
0.0
−0.2
0
50
100
150
200
250
Case # 093, Chromosome 08p

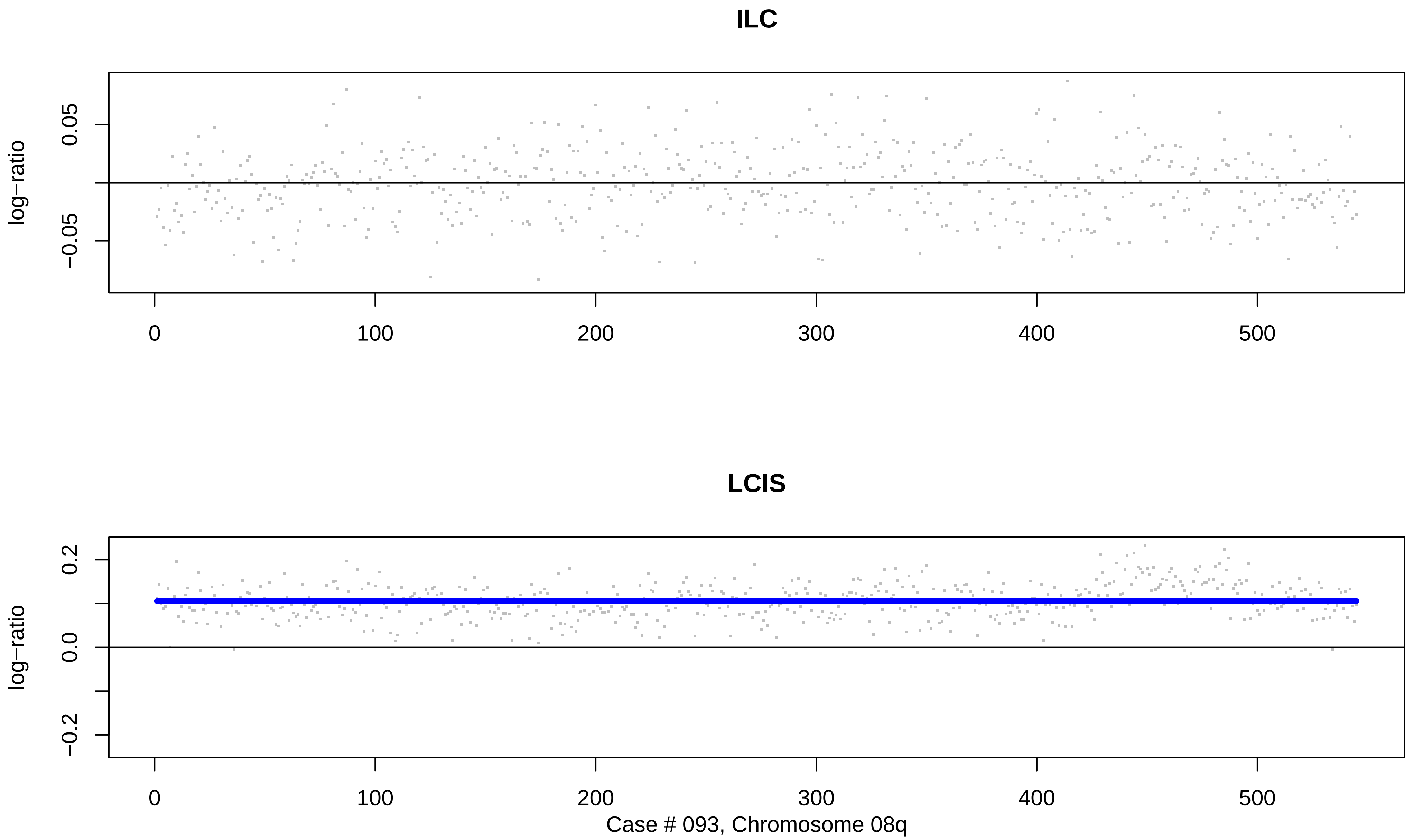
ILC
log−ratio
0.05
−0.05
0
100
200
300
400
500
LCIS
log−ratio
0.2
0.0
−0.2
0
100
200
300
400
500
Case # 093, Chromosome 08q

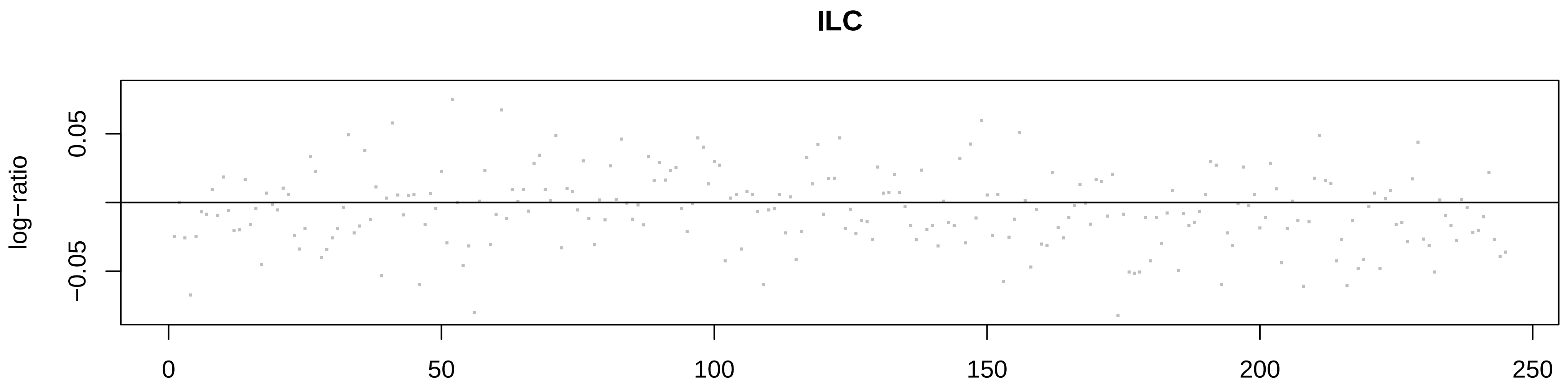
ILC
log−ratio
0.05
−0.05
0
50
100
150
200
250

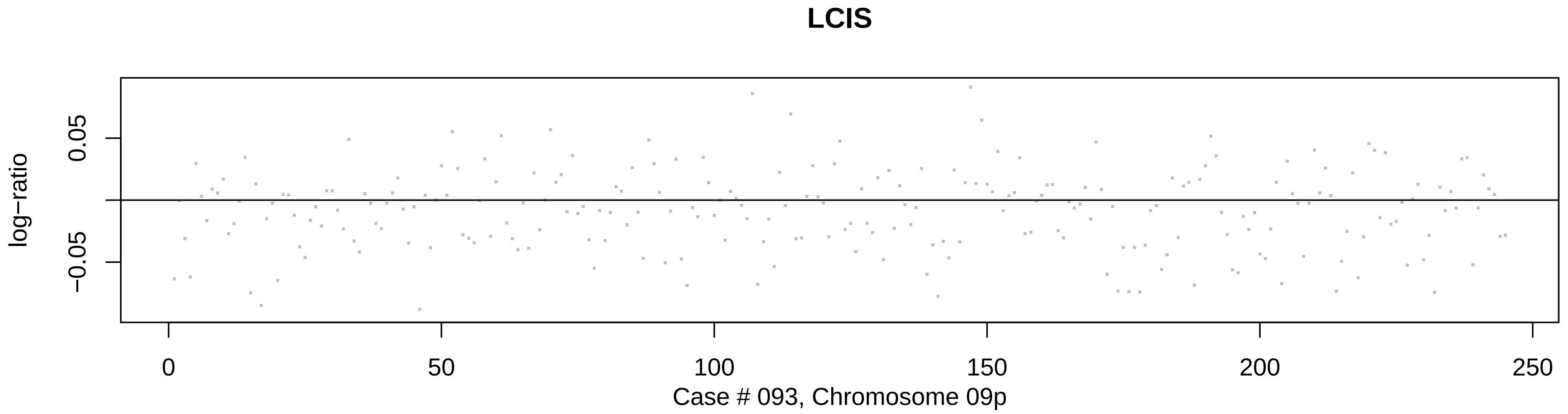
LCIS
log−ratio
0.05
−0.05
0
50
100
150
200
250
Case # 093, Chromosome 09p

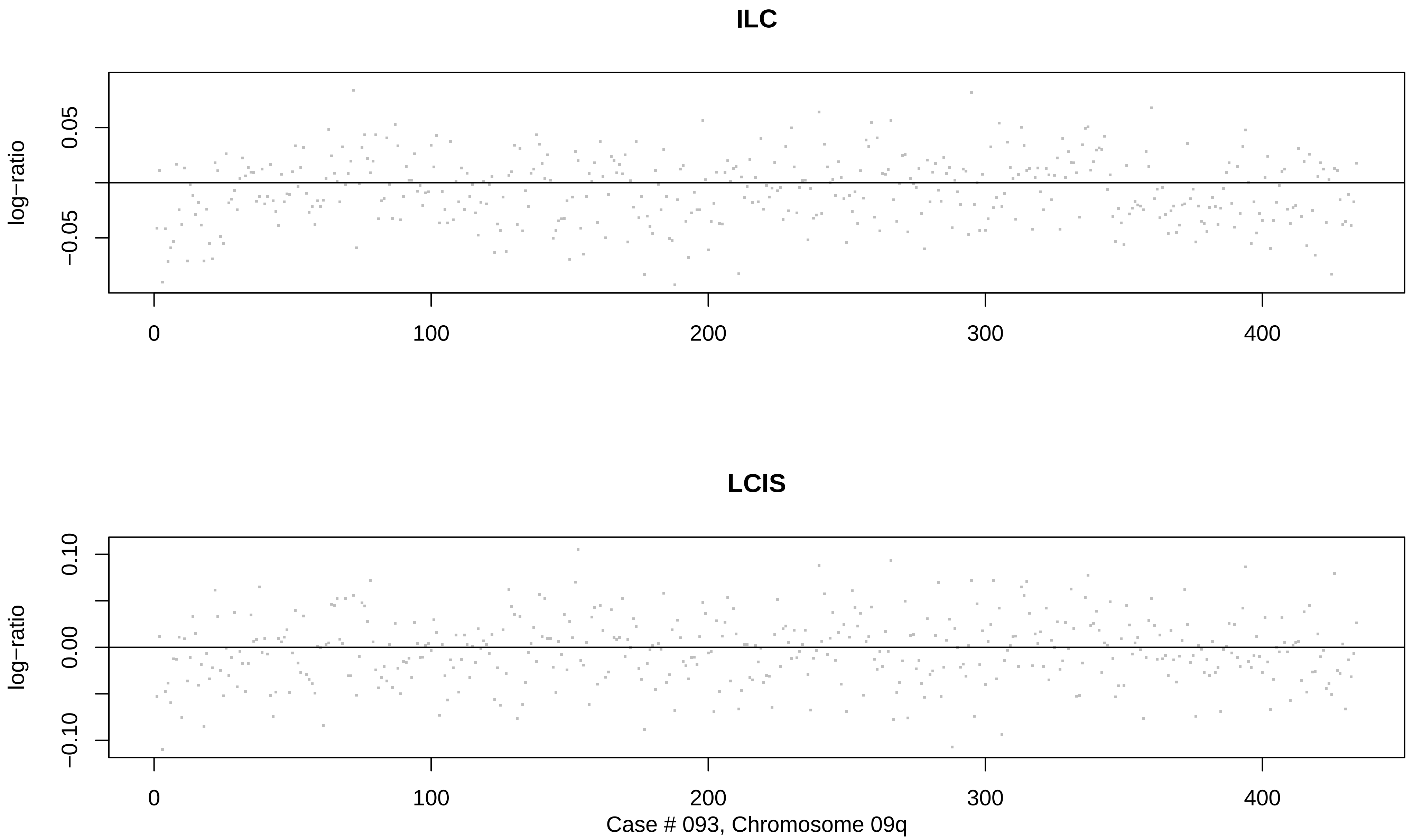
ILC
log−ratio
0.05
−0.05
0
100
200
300
400
LCIS
log−ratio
0.10
0.00
−0.10
0
100
200
300
400
Case # 093, Chromosome 09q

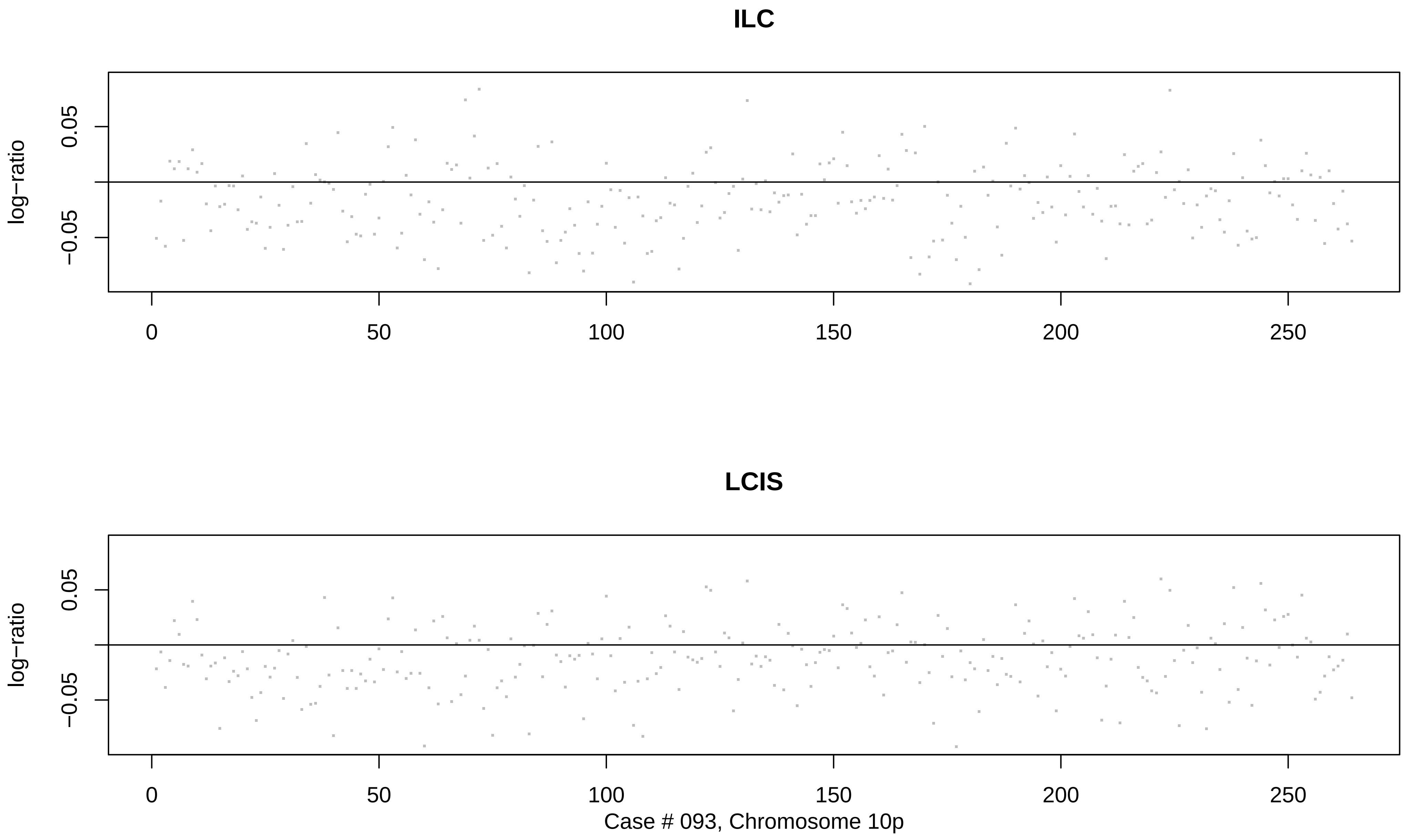

ILC
log−ratio
0.05
−0.05
0
50
100
150
200
250
LCIS
log−ratio
0.05
−0.05
0
50
100
150
200
250
Case # 093, Chromosome 10p

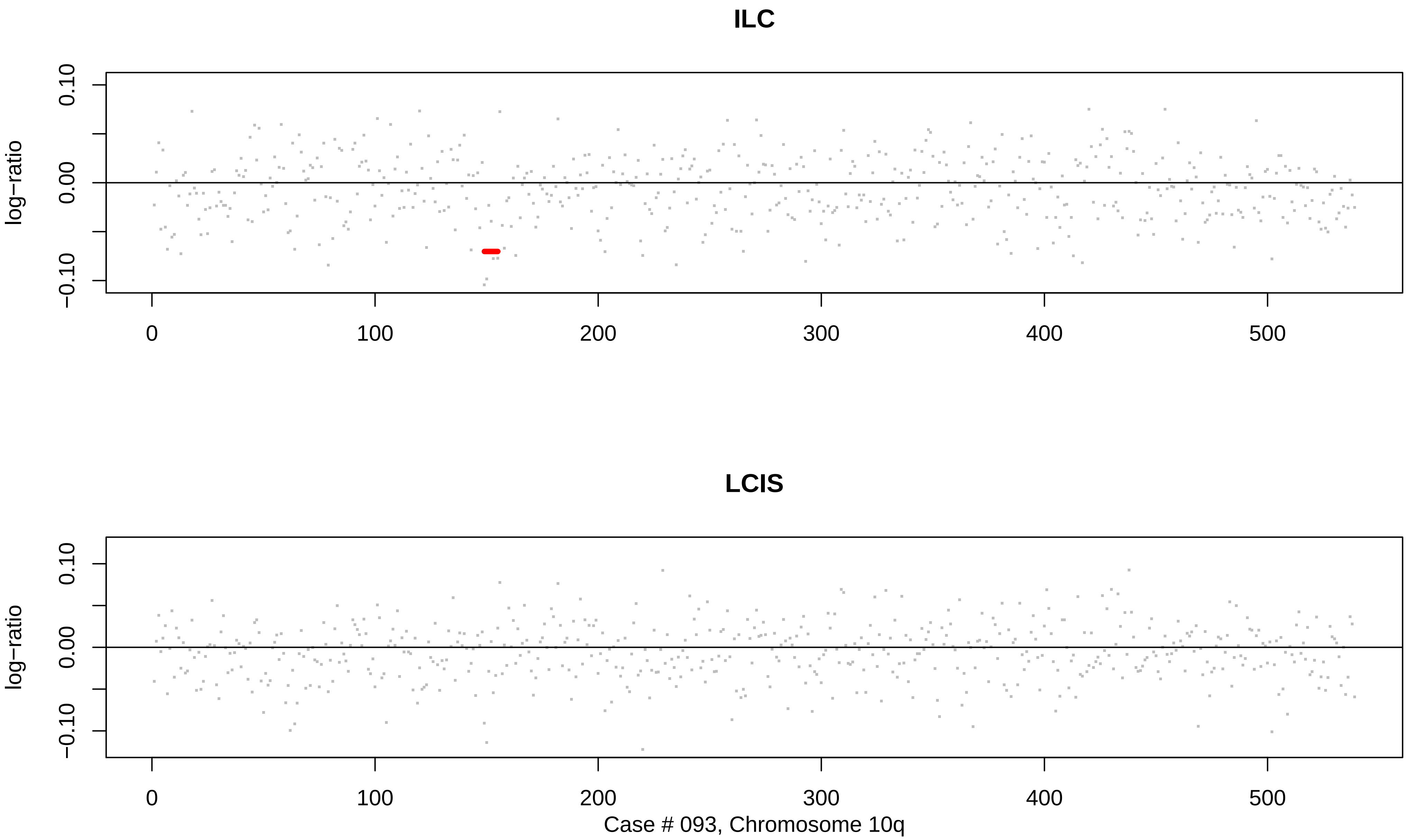
ILC
log−ratio
0.10
0.00
−0.10
0
100
200
300
400
500
LCIS
log−ratio
0.10
0.00
−0.10
0
100
200
300
400
500
Case # 093, Chromosome 10q

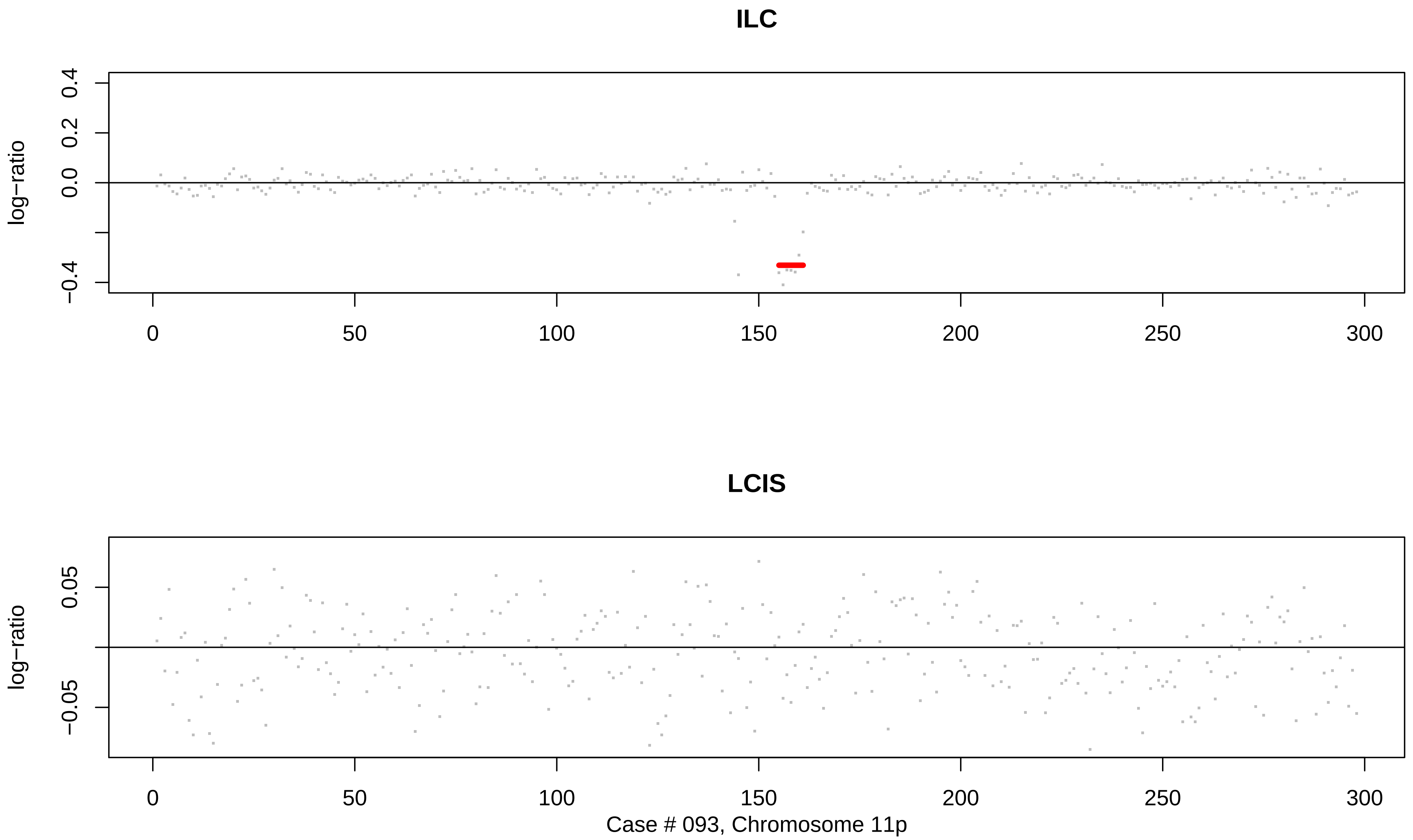
ILC
log−ratio
0.4
0.2
0.0
−0.4
0
50
100
150
200
250
300
LCIS
log−ratio
0.05
−0.05
0
50
100
150
200
250
300
Case # 093, Chromosome 11p

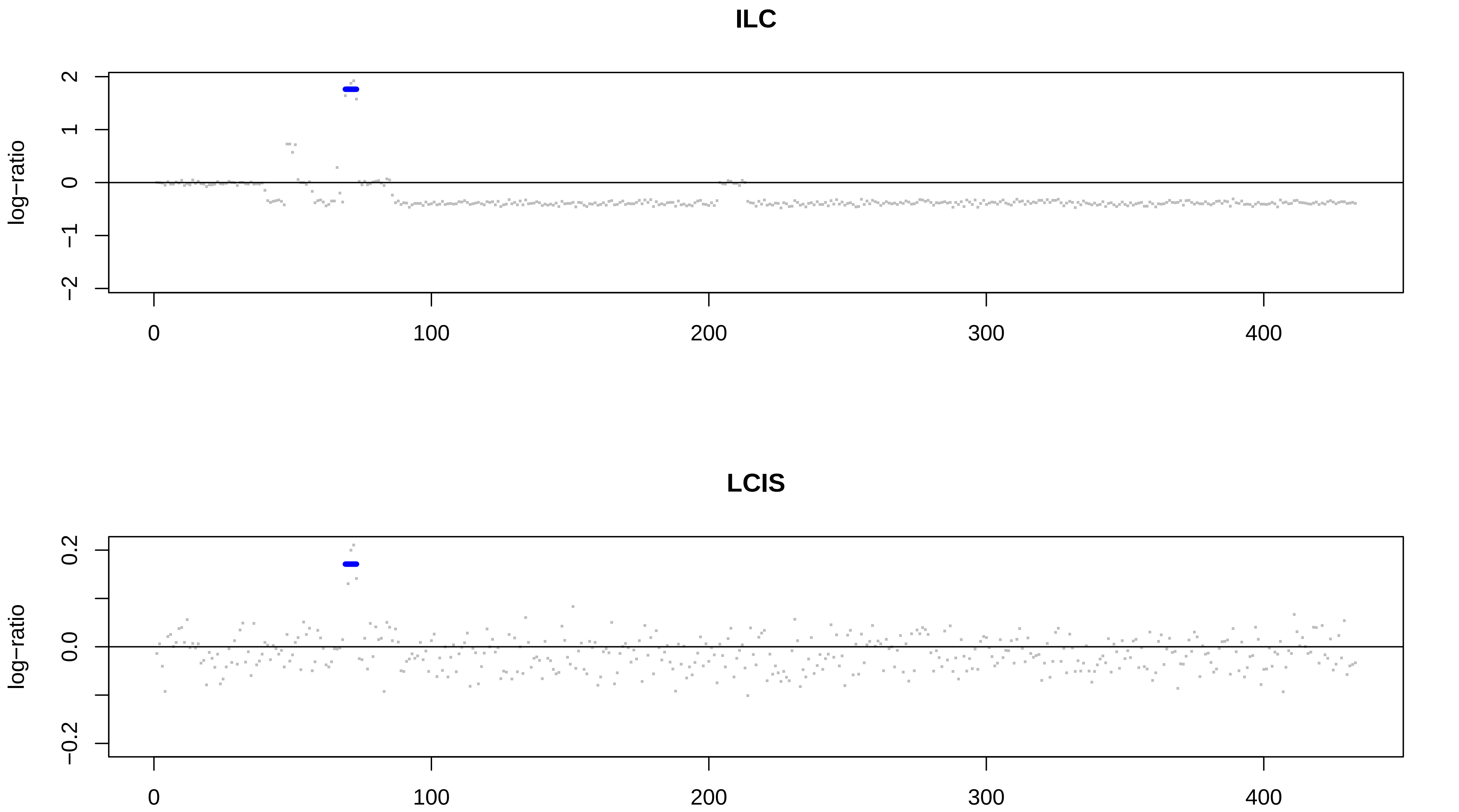

Case # 093, Chromosome 11q
Odds in favor of clonality = 1e+03

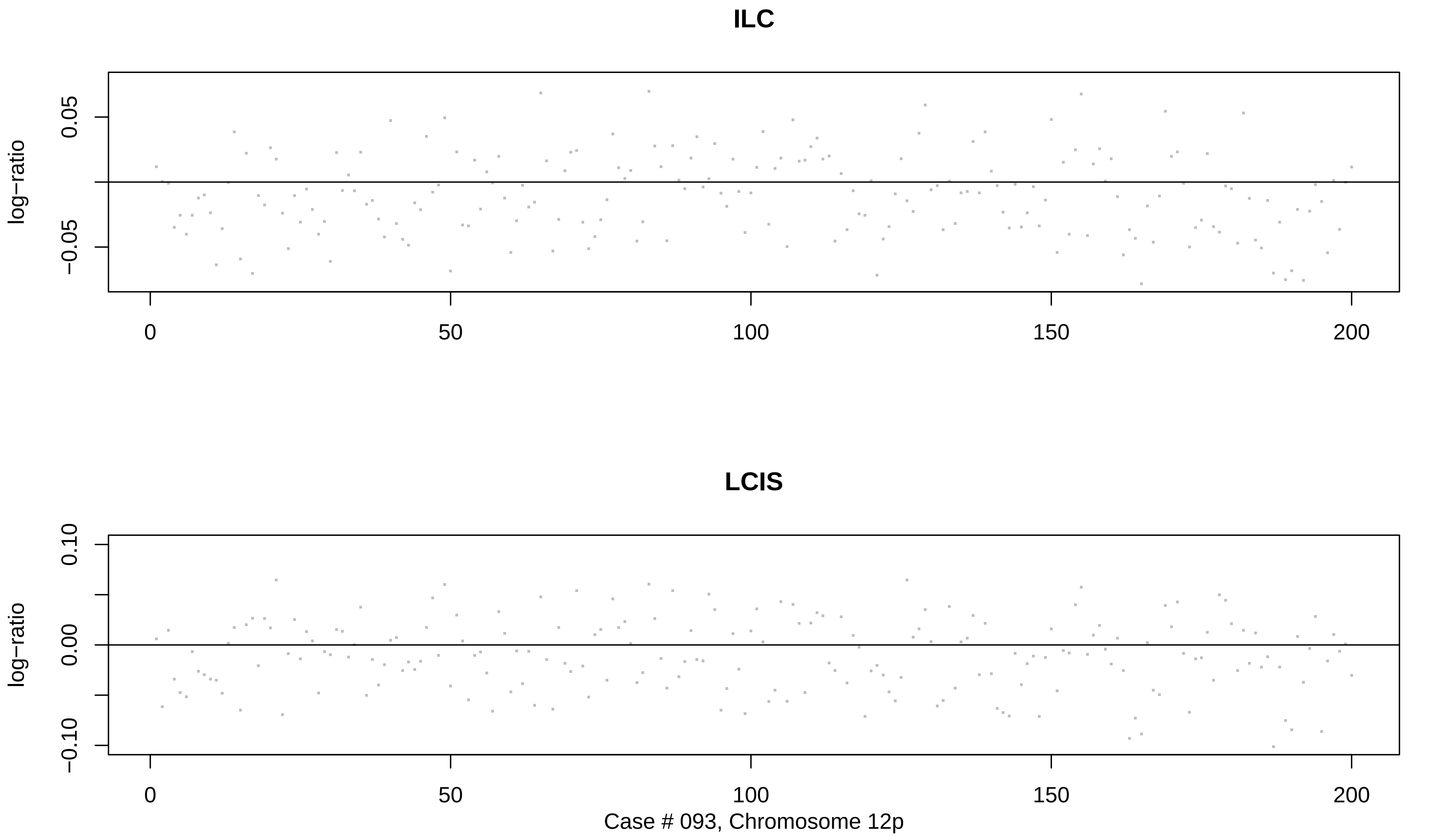
ILC
log−ratio
0.05
−0.05
0
50
100
150
200
LCIS
log−ratio
0.10
0.00
−0.10
0
50
100
150
200
Case # 093, Chromosome 12p

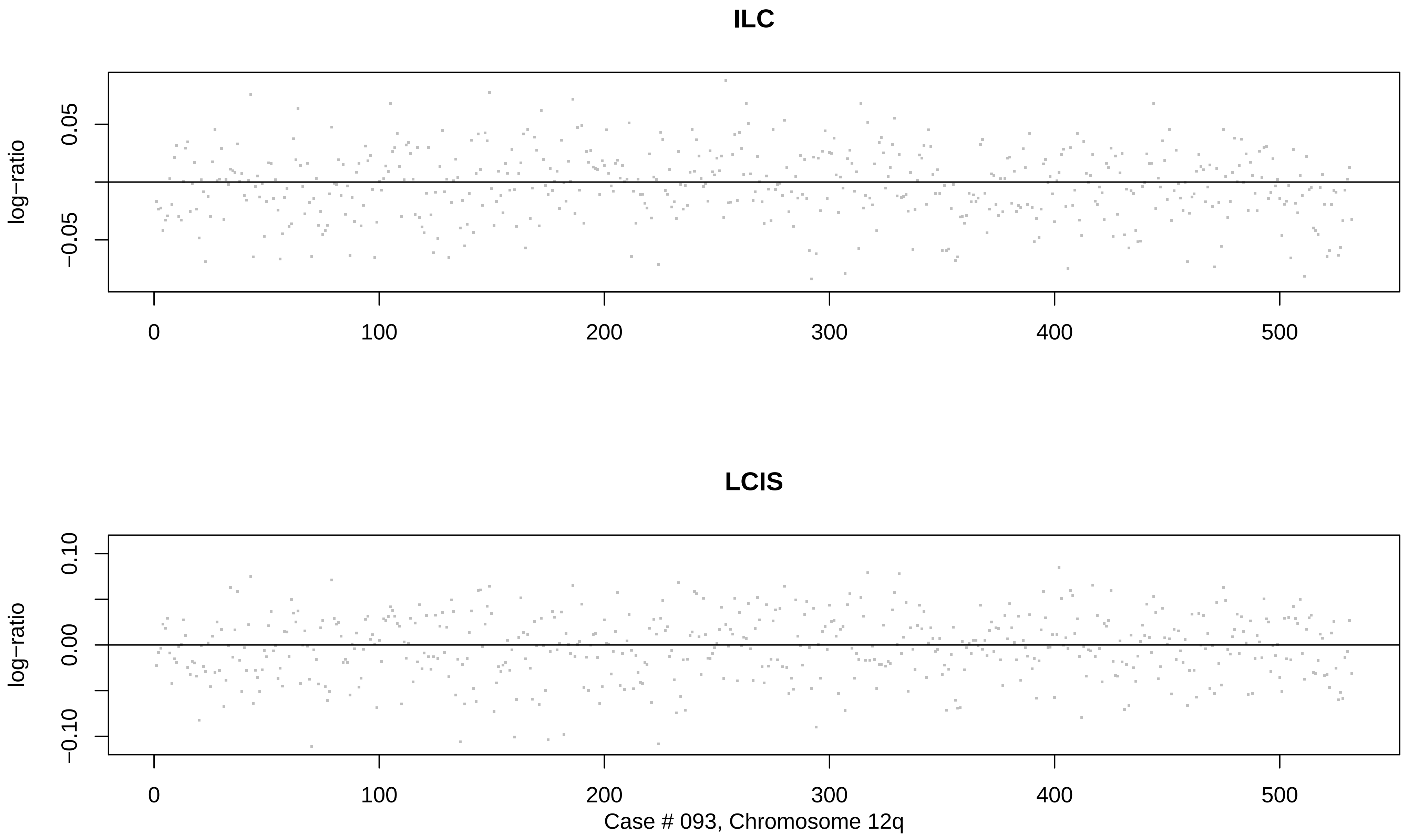
ILC
log−ratio
0.05
−0.05
0
100
200
300
400
500
LCIS
log−ratio
0.10
0.00
−0.10
0
100
200
300
400
500
Case # 093, Chromosome 12q

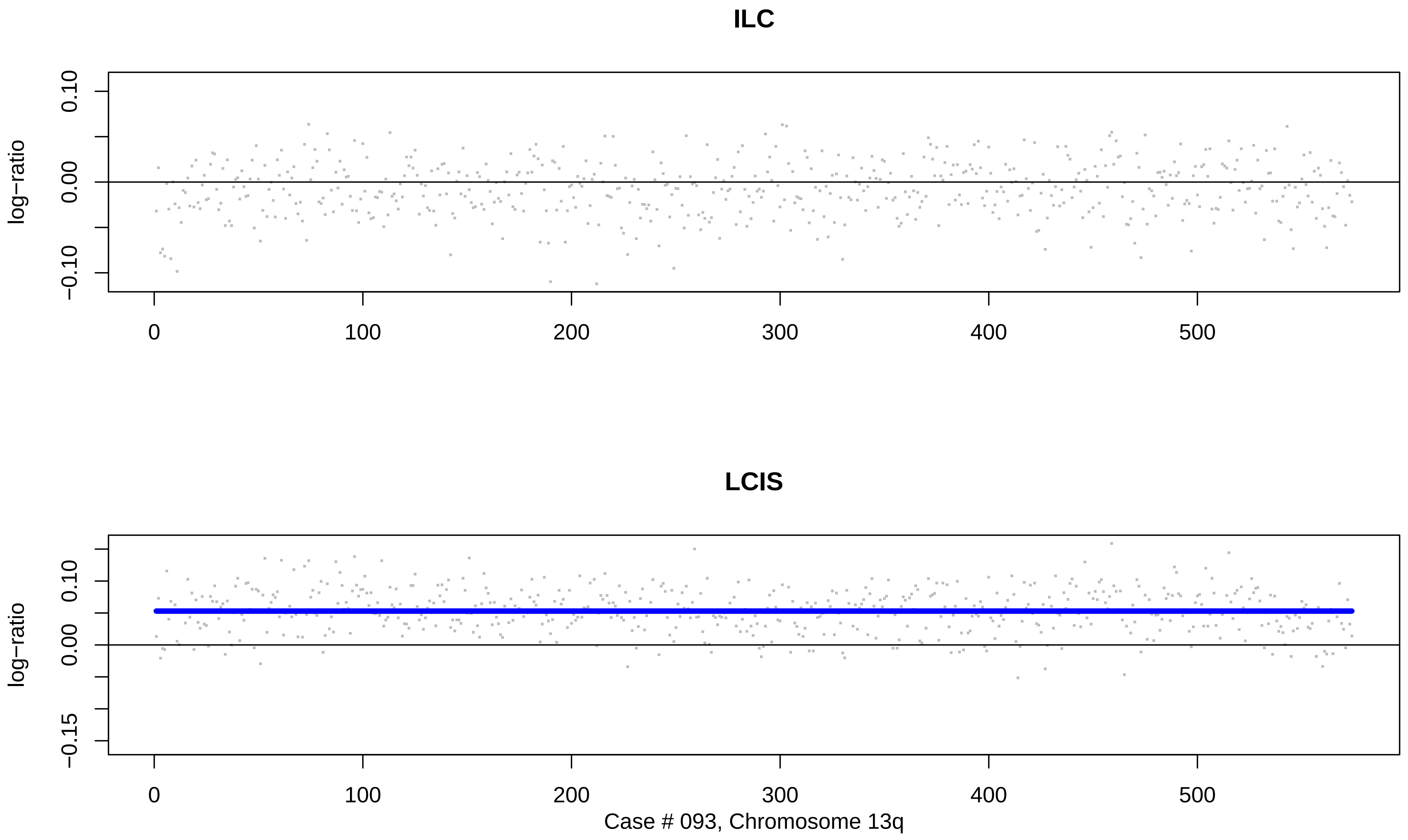
ILC
log−ratio
0.10
0.00
−0.10
0
100
200
300
400
500
LCIS
log−ratio
0.10
0.00
−0.15
0
100
200
300
400
500
Case # 093, Chromosome 13q

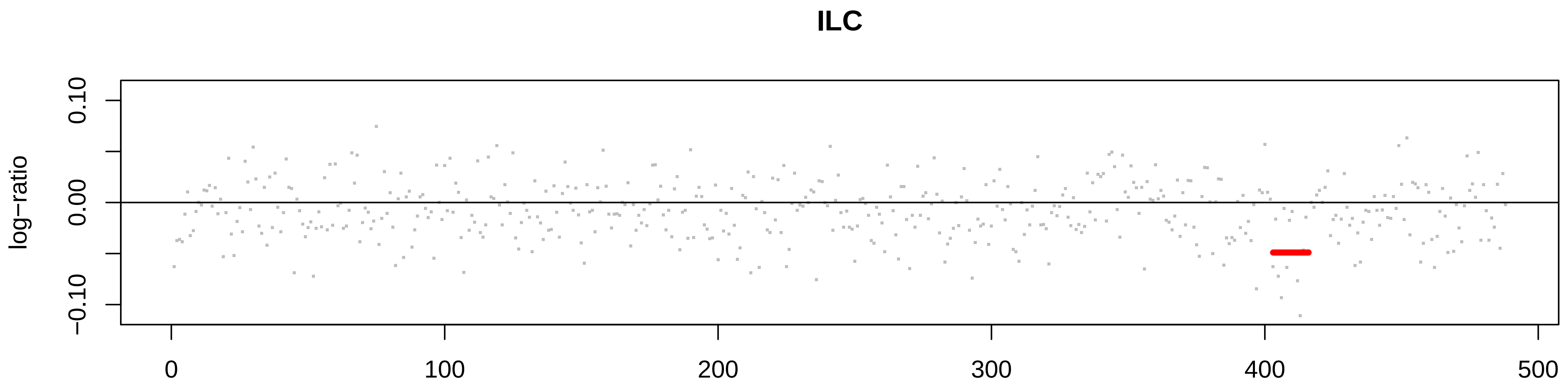

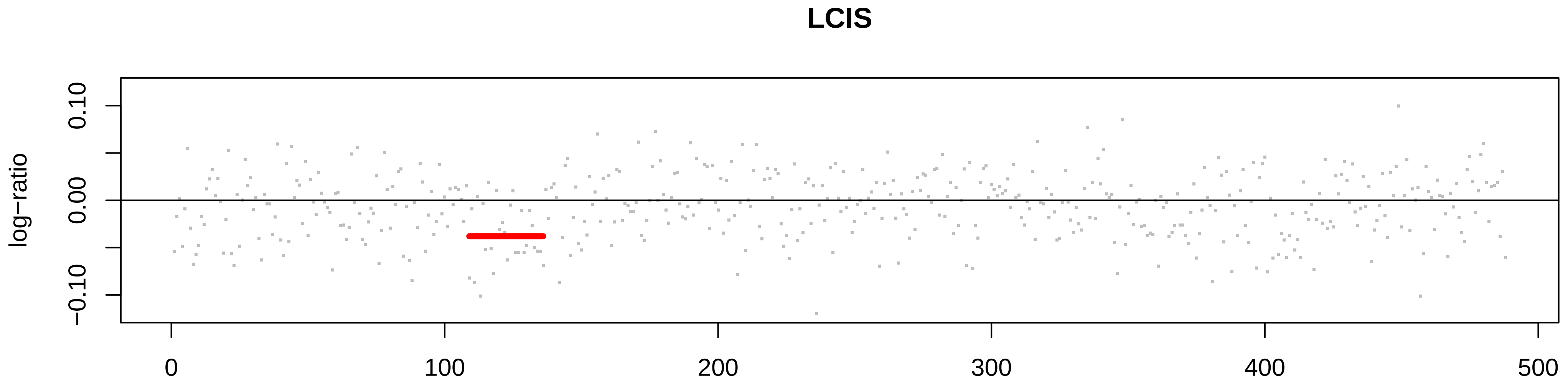

Case # 093, Chromosome 14q
Odds in favor of independence = 4.2

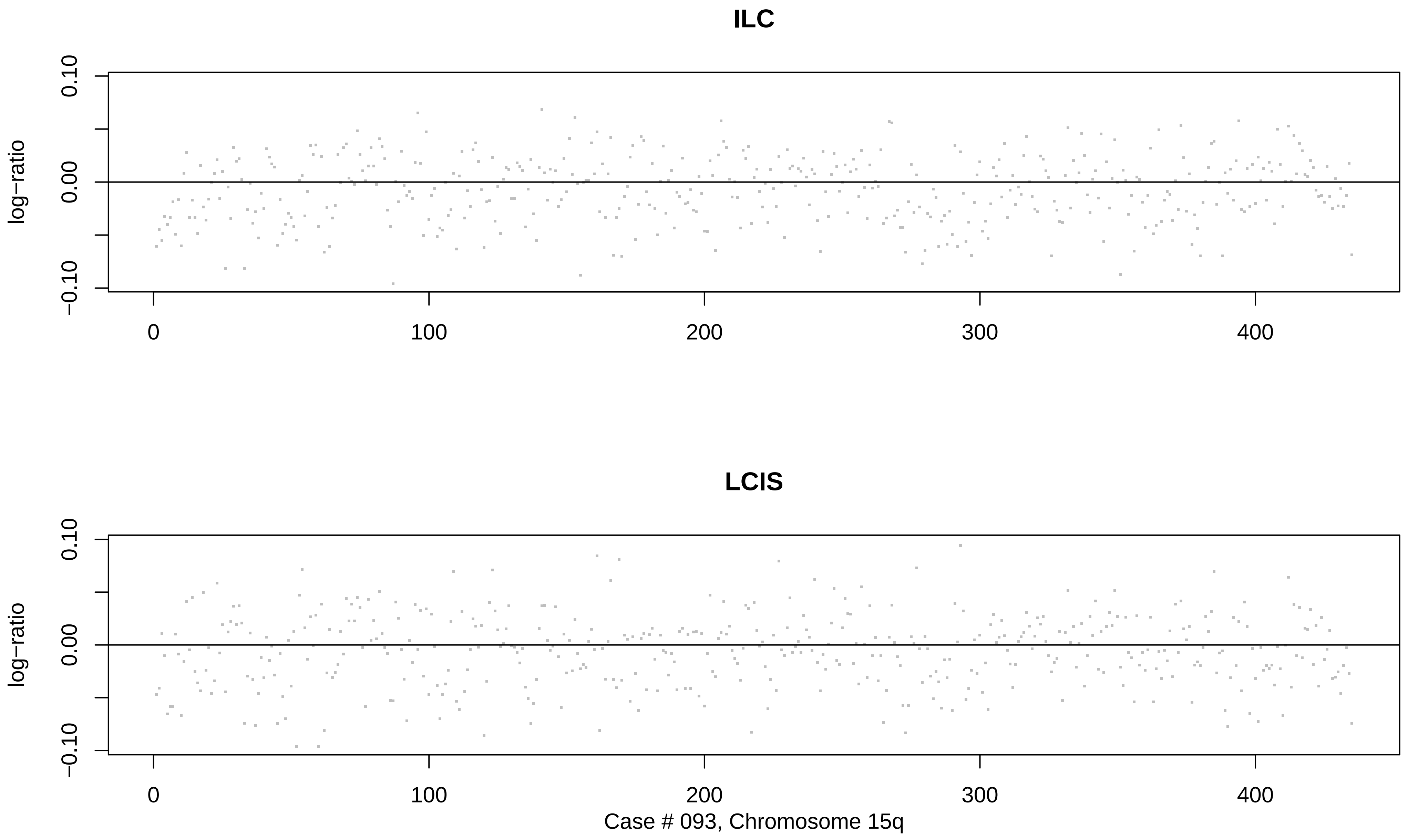
ILC
log−ratio
0.10
0.00
−0.10
0
100
200
300
400
LCIS
log−ratio
0.10
0.00
−0.10
0
100
200
300
400
Case # 093, Chromosome 15q

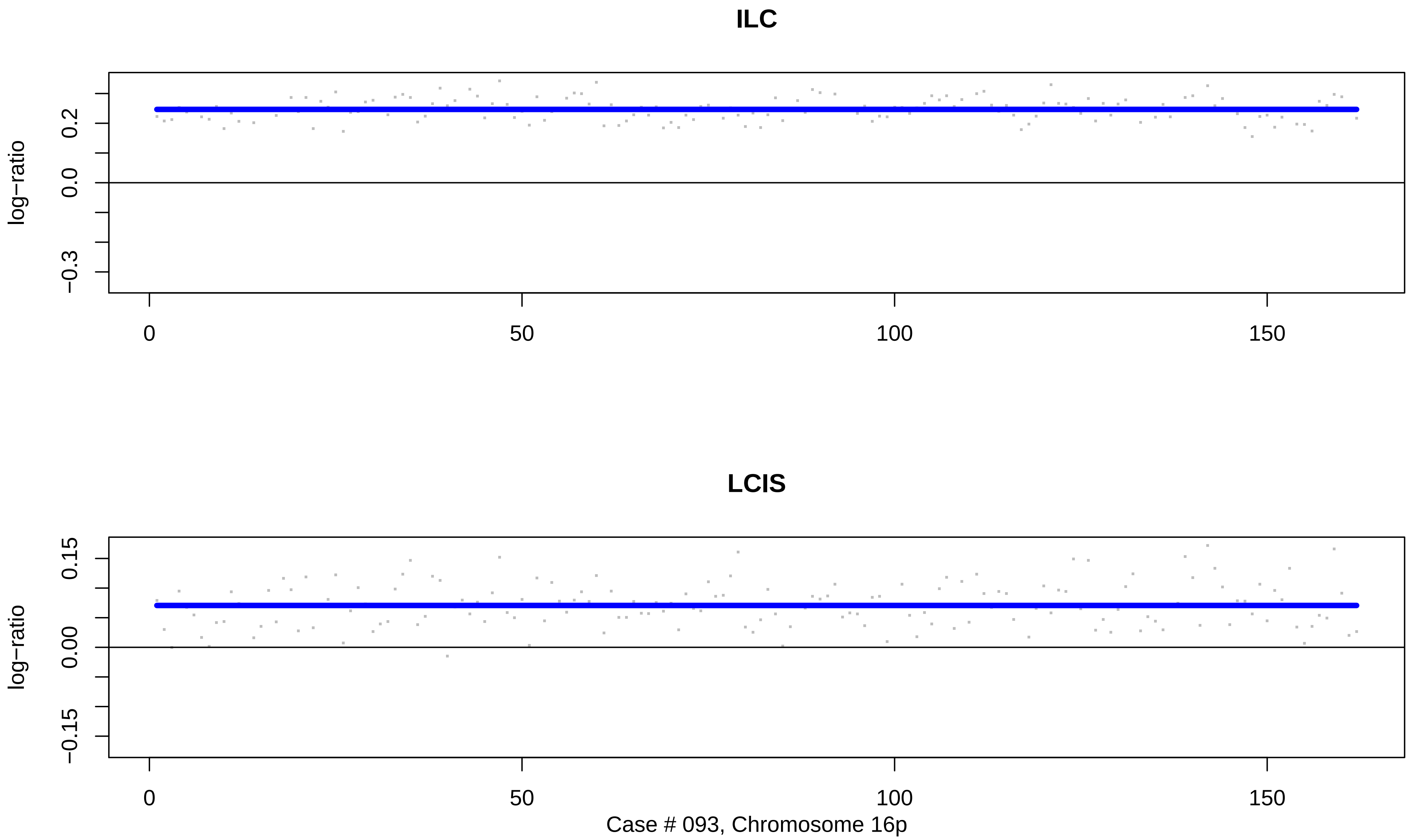

ILC
log−ratio
0.2
0.0
−0.3
0
50
100
150
LCIS
log−ratio
0.15
0.00
−0.15
0
50
100
150
Case # 093, Chromosome 16p

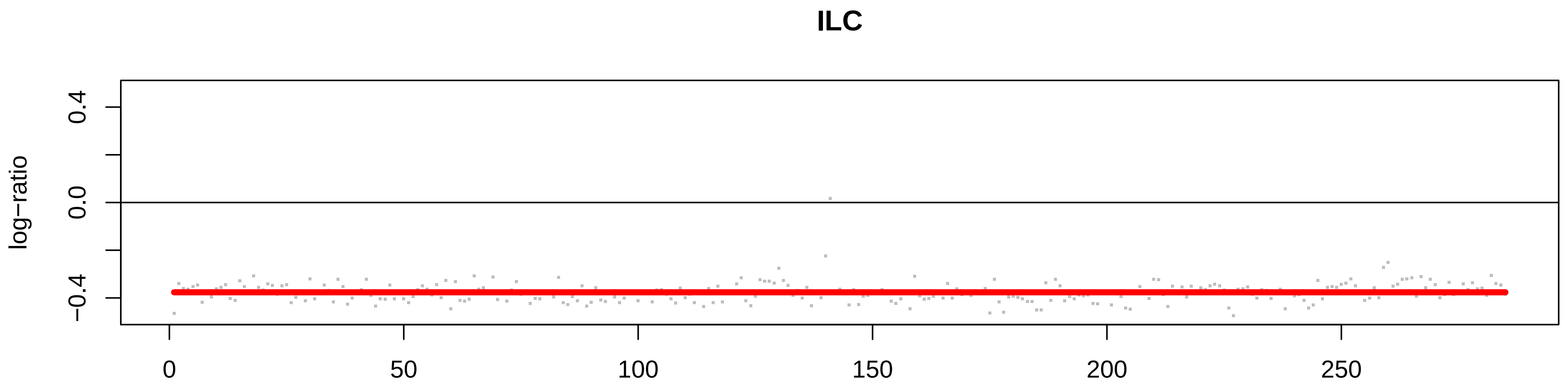
ILC
log−ratio
0.4
0.0
−0.4
0
50
100
150
200
250

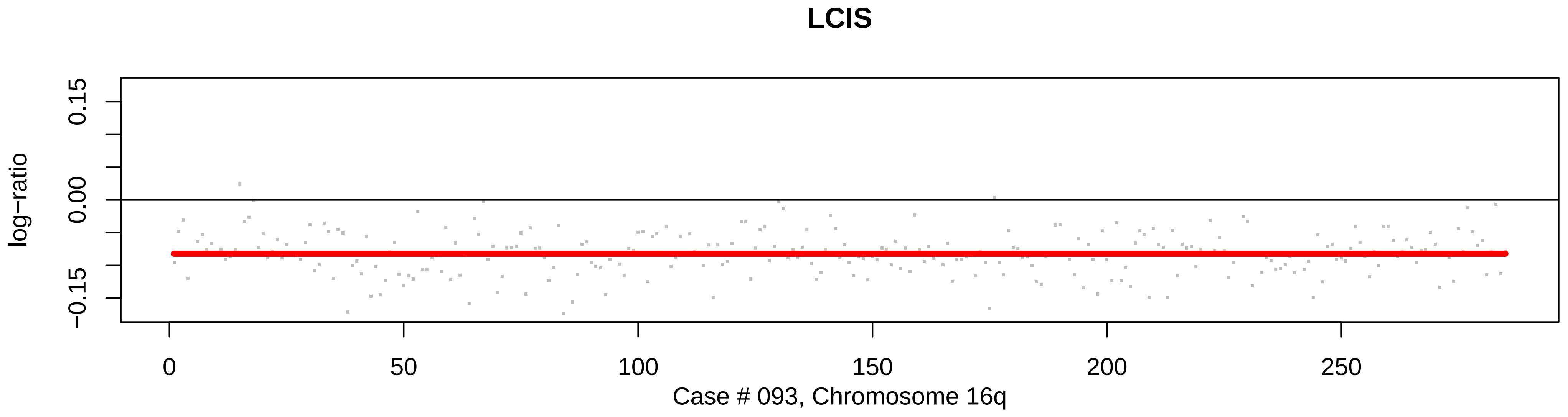
LCIS
log−ratio
0.15
0.00
−0.15
0
50
100
150
200
250
Case # 093, Chromosome 16q

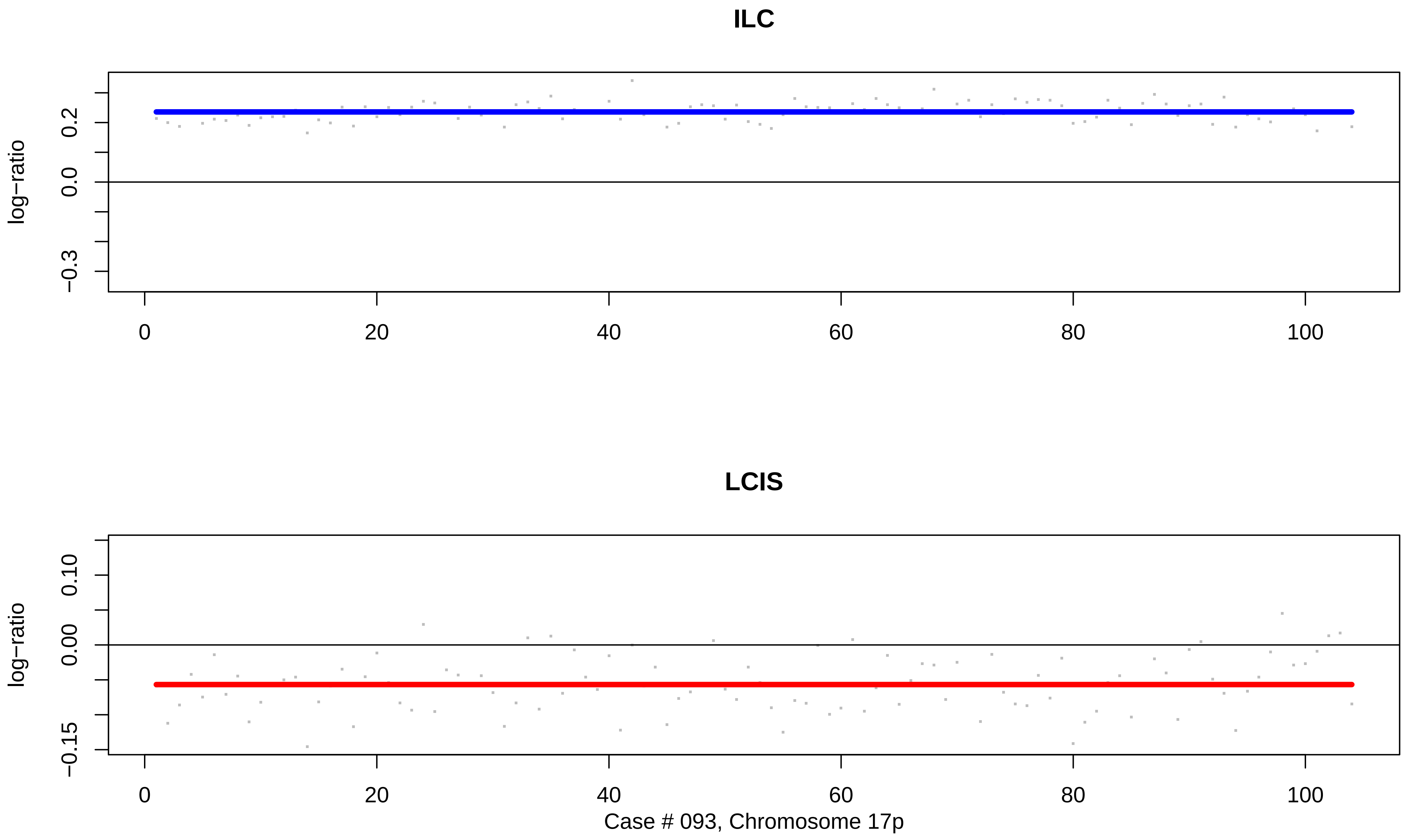
ILC
log−ratio
0.2
0.0
−0.3
0
20
40
60
80
100
LCIS
log−ratio
0.10
0.00
−0.15
0
20
40
60
80
100
Case # 093, Chromosome 17p

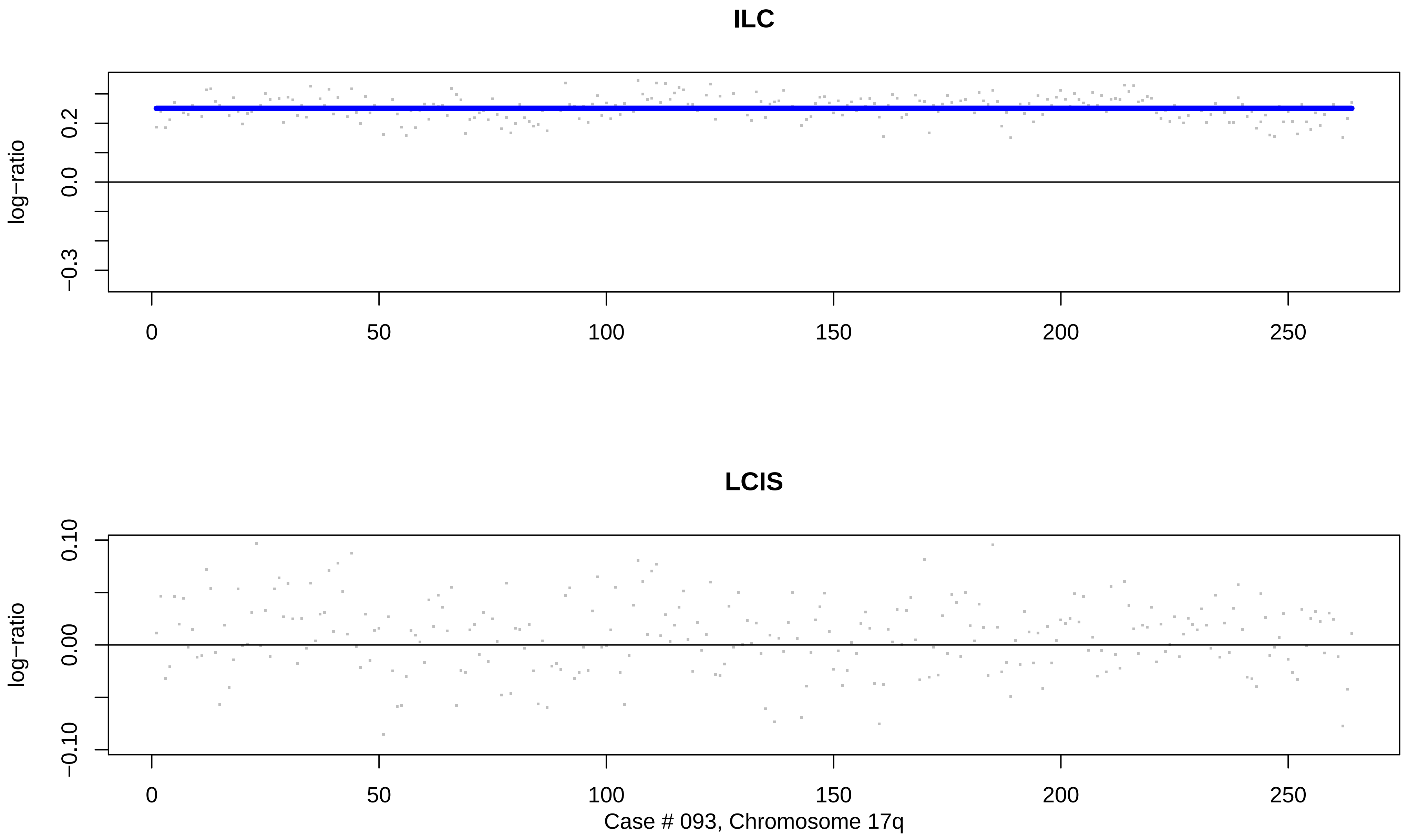
ILC
log−ratio
−0.3
0.0
0.2
0
50
100
150
200
250
LCIS
log−ratio
−0.10
0.00
0.10
0
50
100
150
200
250
Case # 093, Chromosome 17q

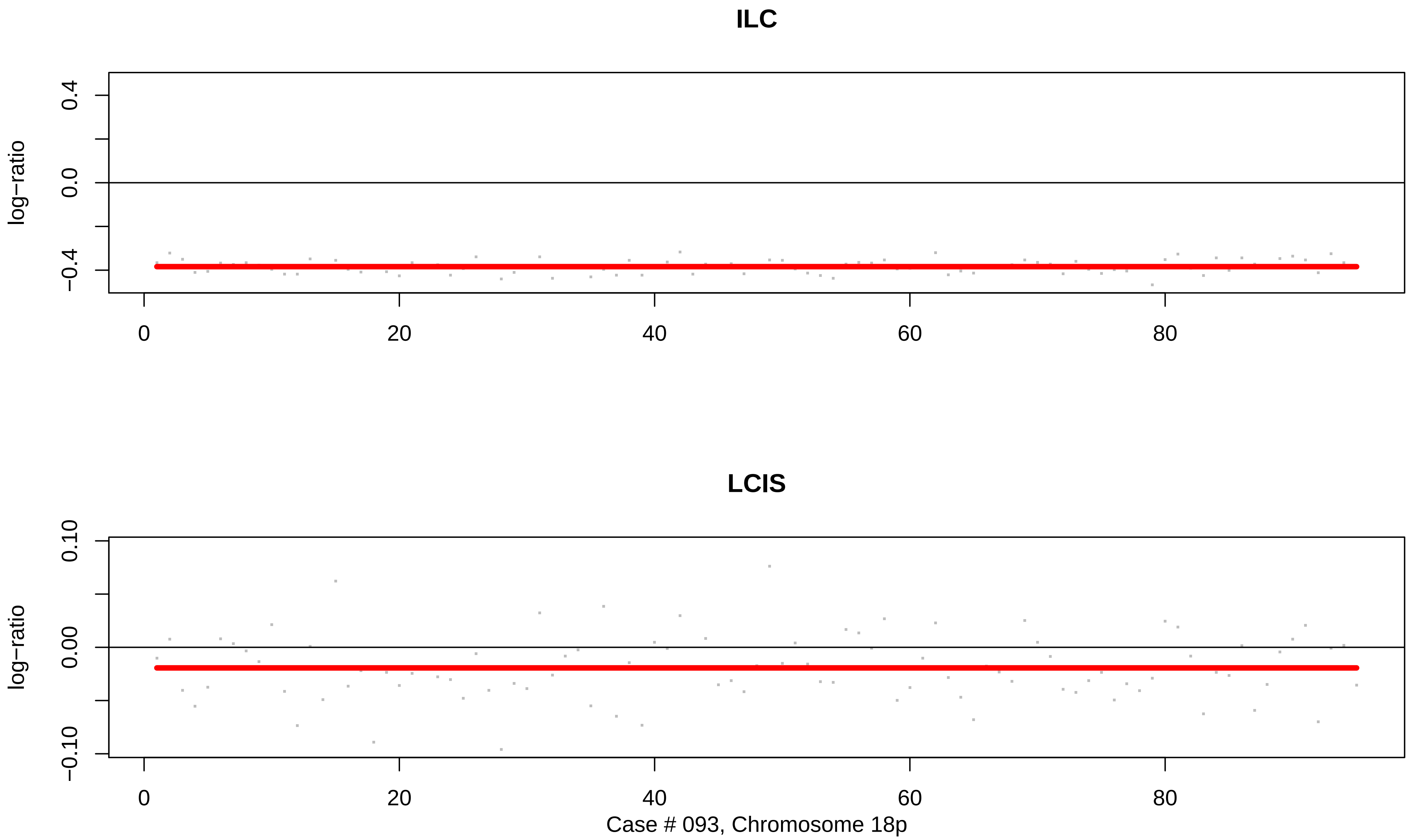
ILC
log−ratio
0.4
0.0
−0.4
0
20
40
60
80
LCIS
log−ratio
0.10
0.00
−0.10
0
20
40
60
80
Case # 093, Chromosome 18p

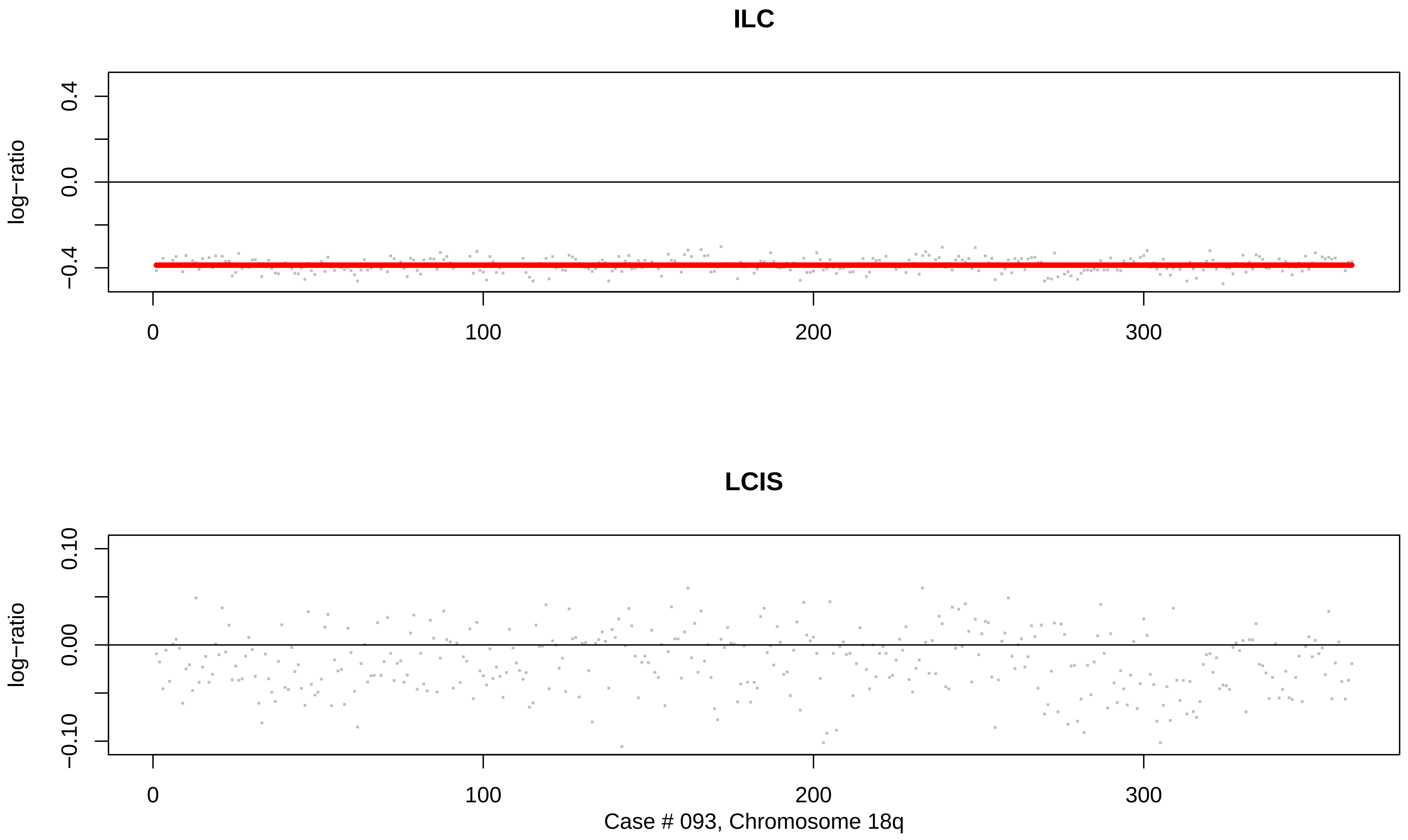
ILC
log−ratio
0.4
0.0
−0.4
0
100
200
300
LCIS
log−ratio
0.10
0.00
−0.10
0
100
200
300
Case # 093, Chromosome 18q

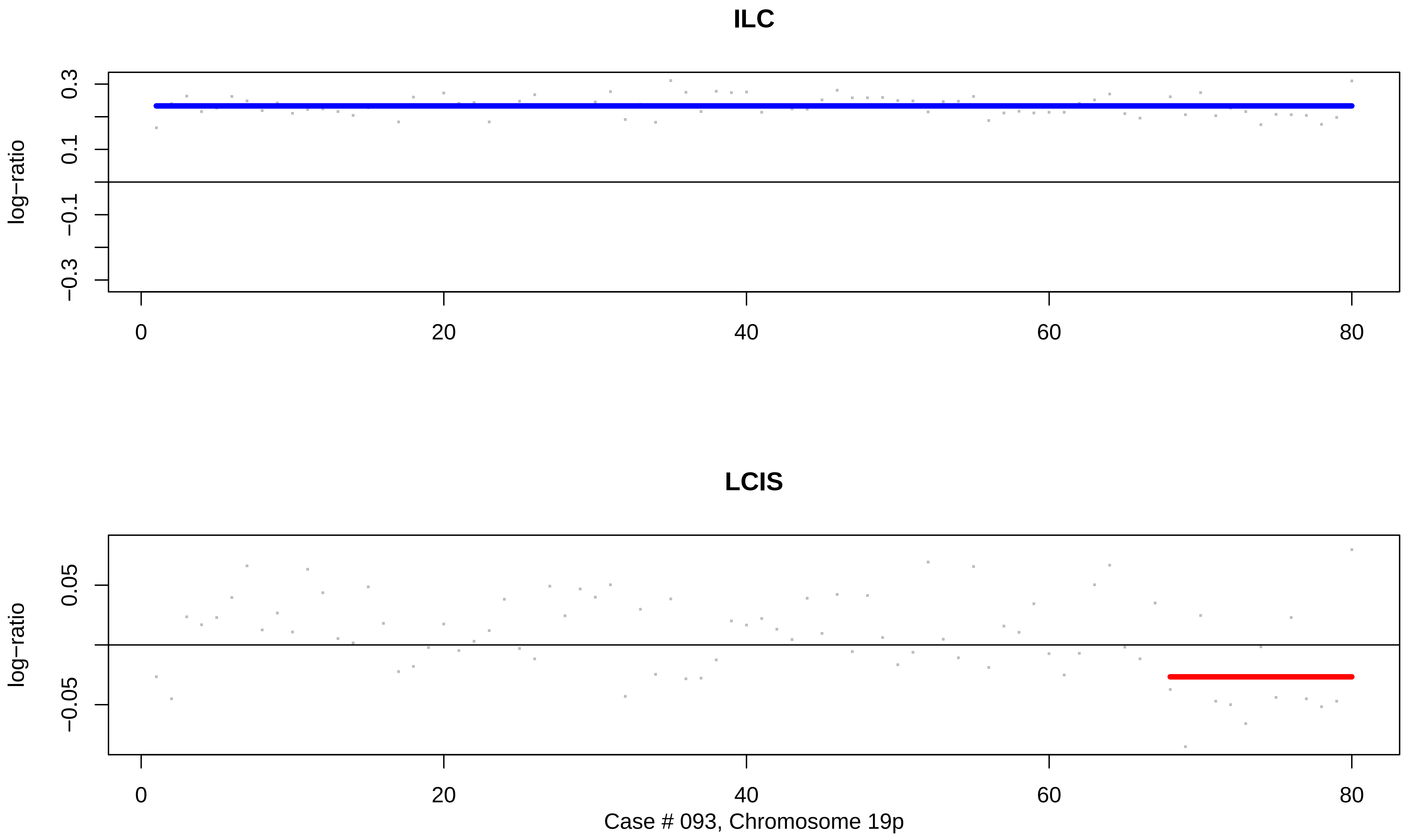
ILC
log−ratio
0.3
0.1
−0.1
−0.3
0
20
40
60
80
LCIS
log−ratio
0.05
−0.05
0
20
40
60
80
Case # 093, Chromosome 19p

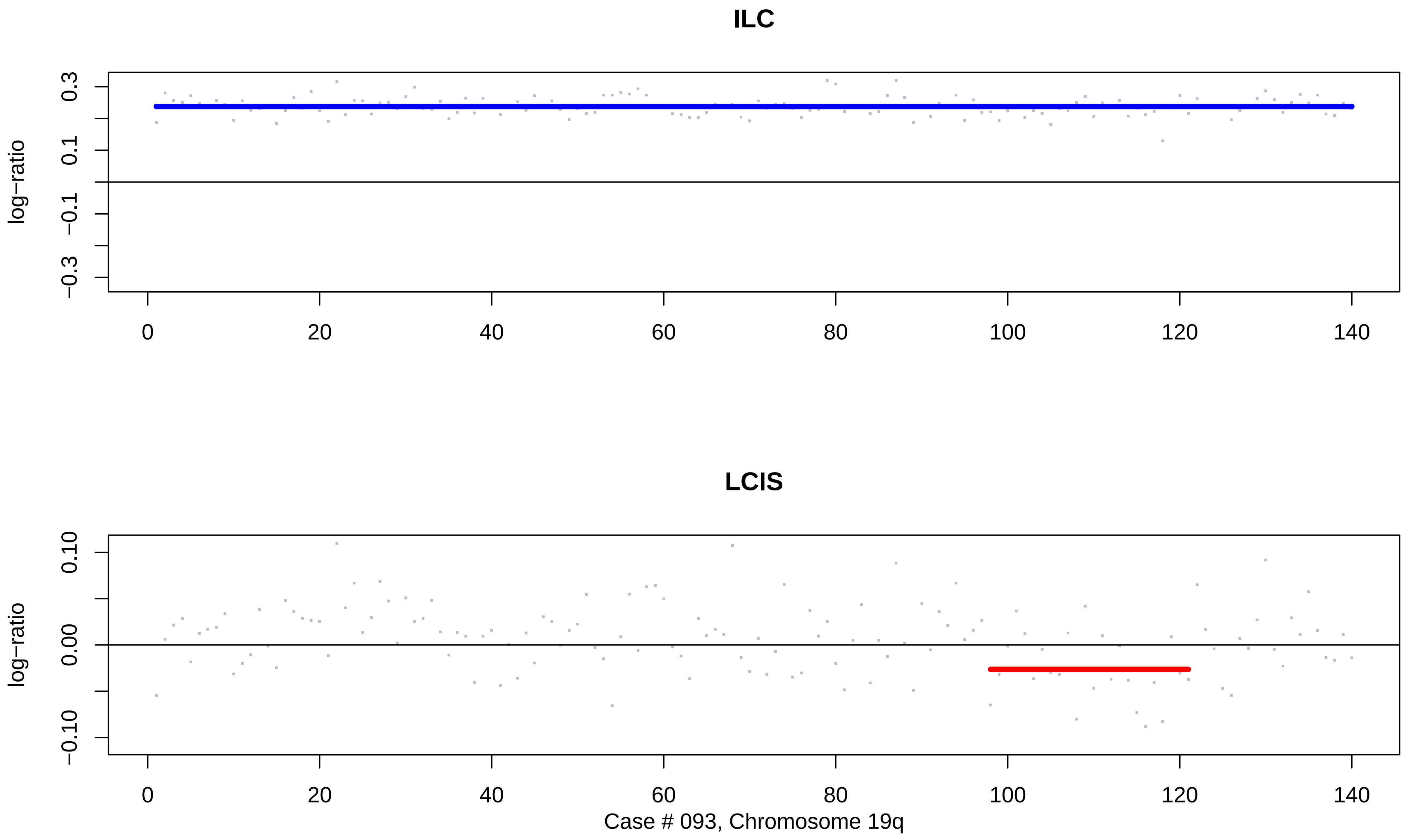
ILC
log−ratio
0.3
0.1
−0.1
−0.3
0
20
40
60
80
100
120
140
LCIS
log−ratio
0.10
0.00
−0.10
0
20
40
60
80
100
120
140
Case # 093, Chromosome 19q

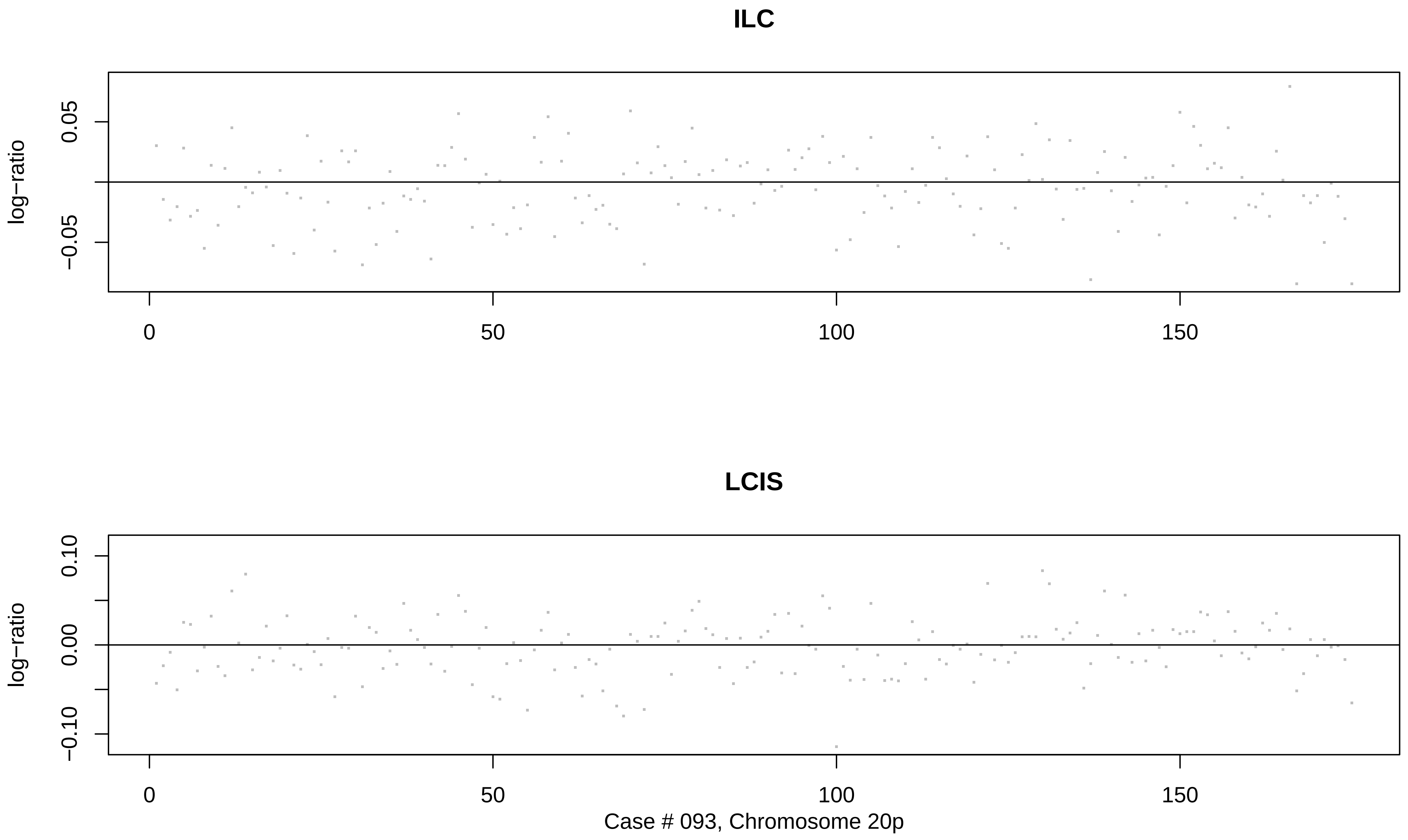
ILC
log−ratio
0.05
−0.05
0
50
100
150
LCIS
log−ratio
0.10
0.00
−0.10
0
50
100
150
Case # 093, Chromosome 20p

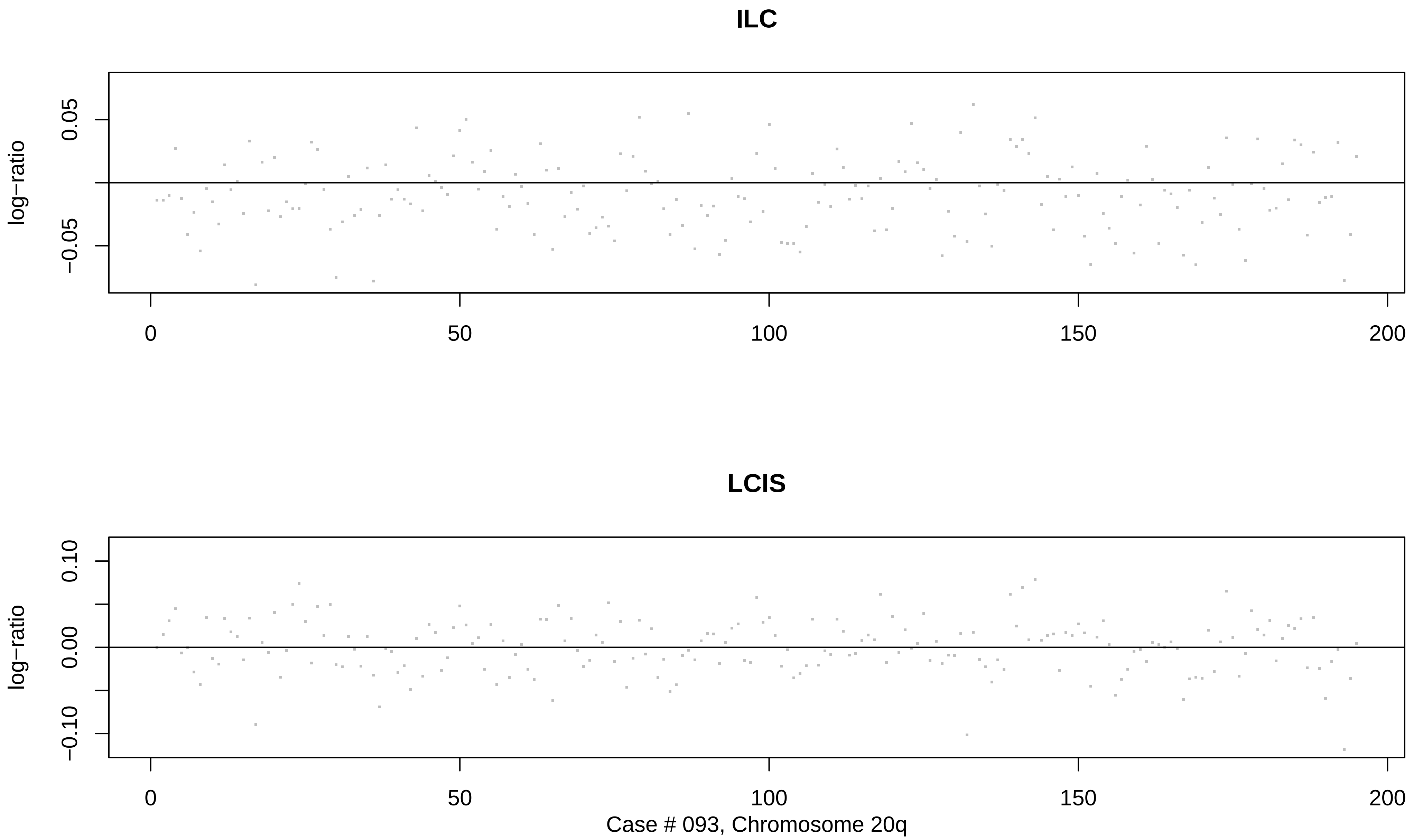
ILC
log−ratio
0.05
−0.05
0
50
100
150
200
LCIS
log−ratio
0.10
0.00
−0.10
0
50
100
150
200
Case # 093, Chromosome 20q

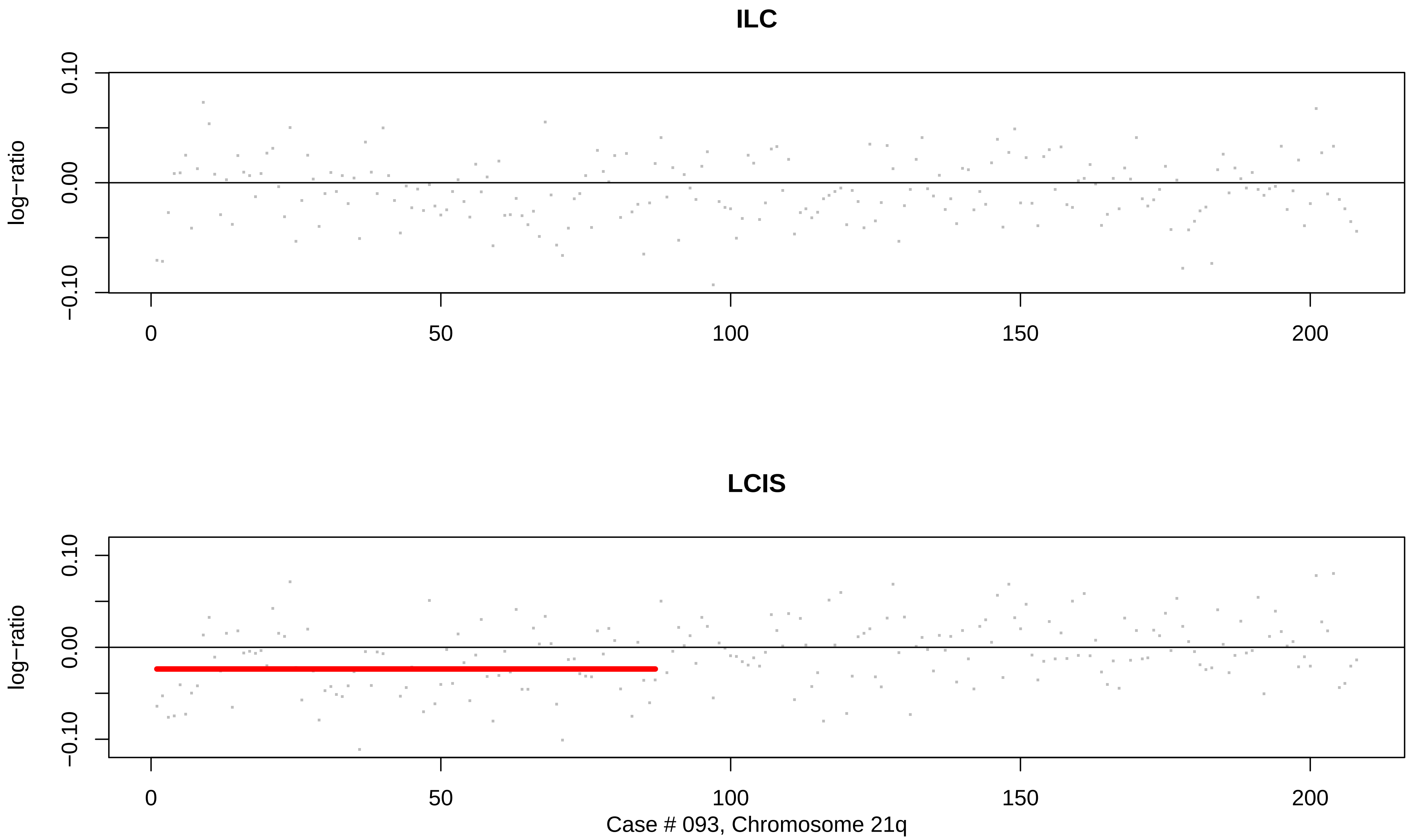

ILC
log−ratio
0.10
0.00
−0.10
0
50
100
150
200
LCIS
log−ratio
0.10
0.00
−0.10
0
50
100
150
200
Case # 093, Chromosome 21q

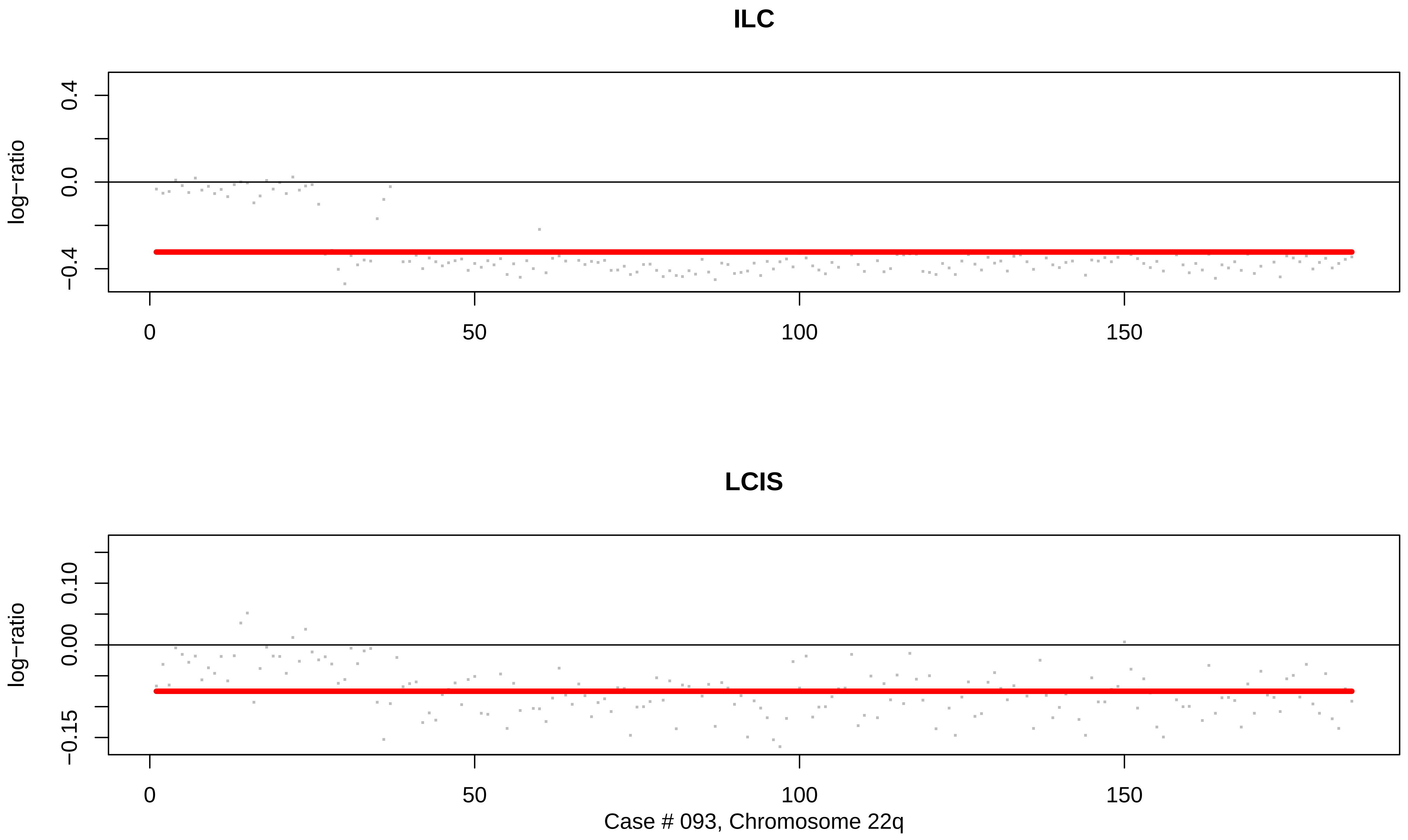
ILC
log−ratio
0.4
0.0
−0.4
0
50
100
150
LCIS
log−ratio
0.10
0.00
−0.15
0
50
100
150
Case # 093, Chromosome 22q
