# Supplementary material for: Clonal relatedness between lobular carcinoma in situ and synchronous malignant lesions
Source: Breast Cancer Res. 2012 Jul 9;14(4):R103. doi: 10.1186/bcr3222 (PMC3680923; doi:10.1186/bcr3222)
Supplement: Additional file 4 — Magnified version of genome-wide plots with detailed marker plots and segmentation on a chromosome-arm-specific basis. [file bcr3222-S4.ZIP › Case 083.pdf]

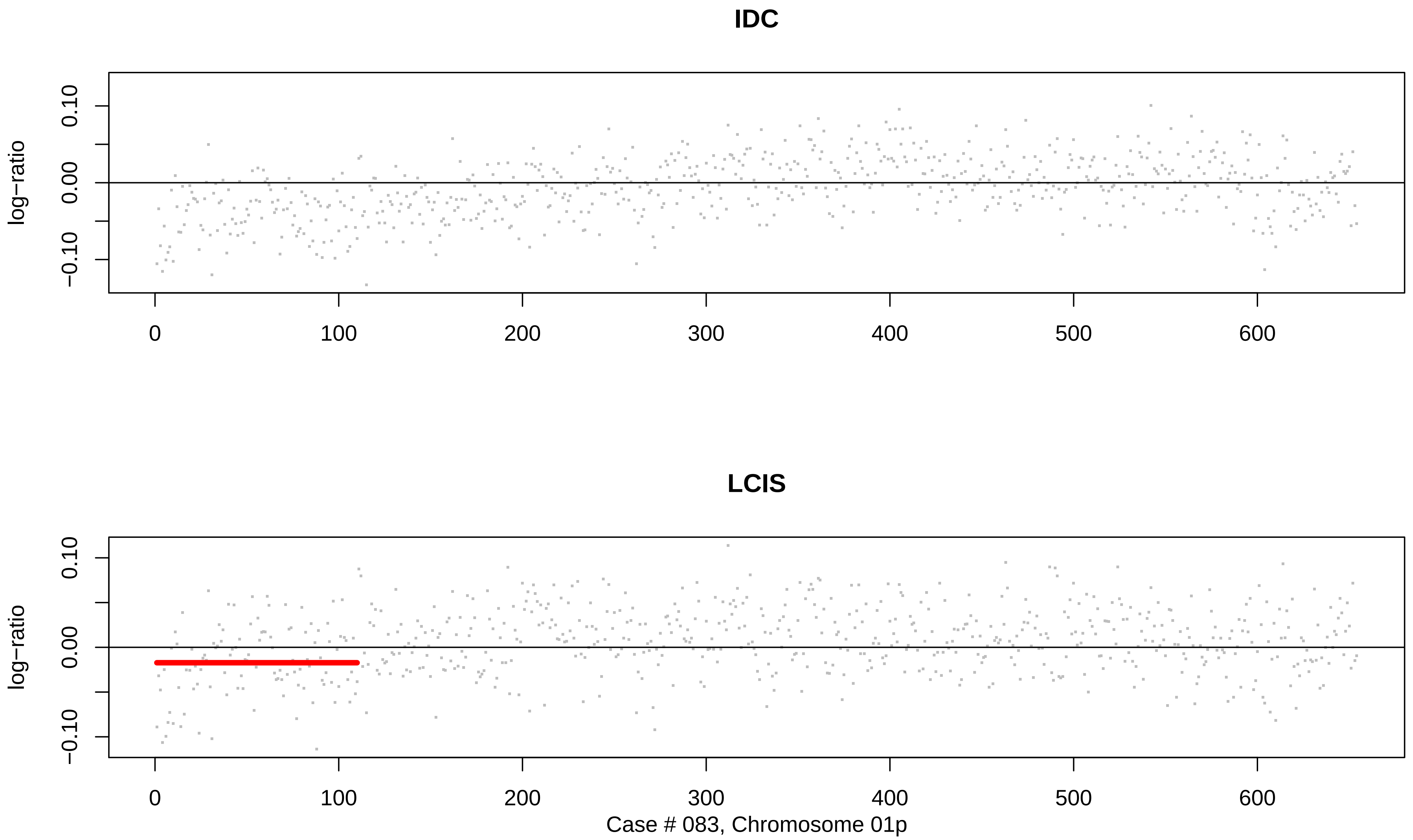

IDC
LCIS
log−ratio
0.10
0.00
−0.10
0
100
200
300
400
500
600
Case # 083, Chromosome 01p

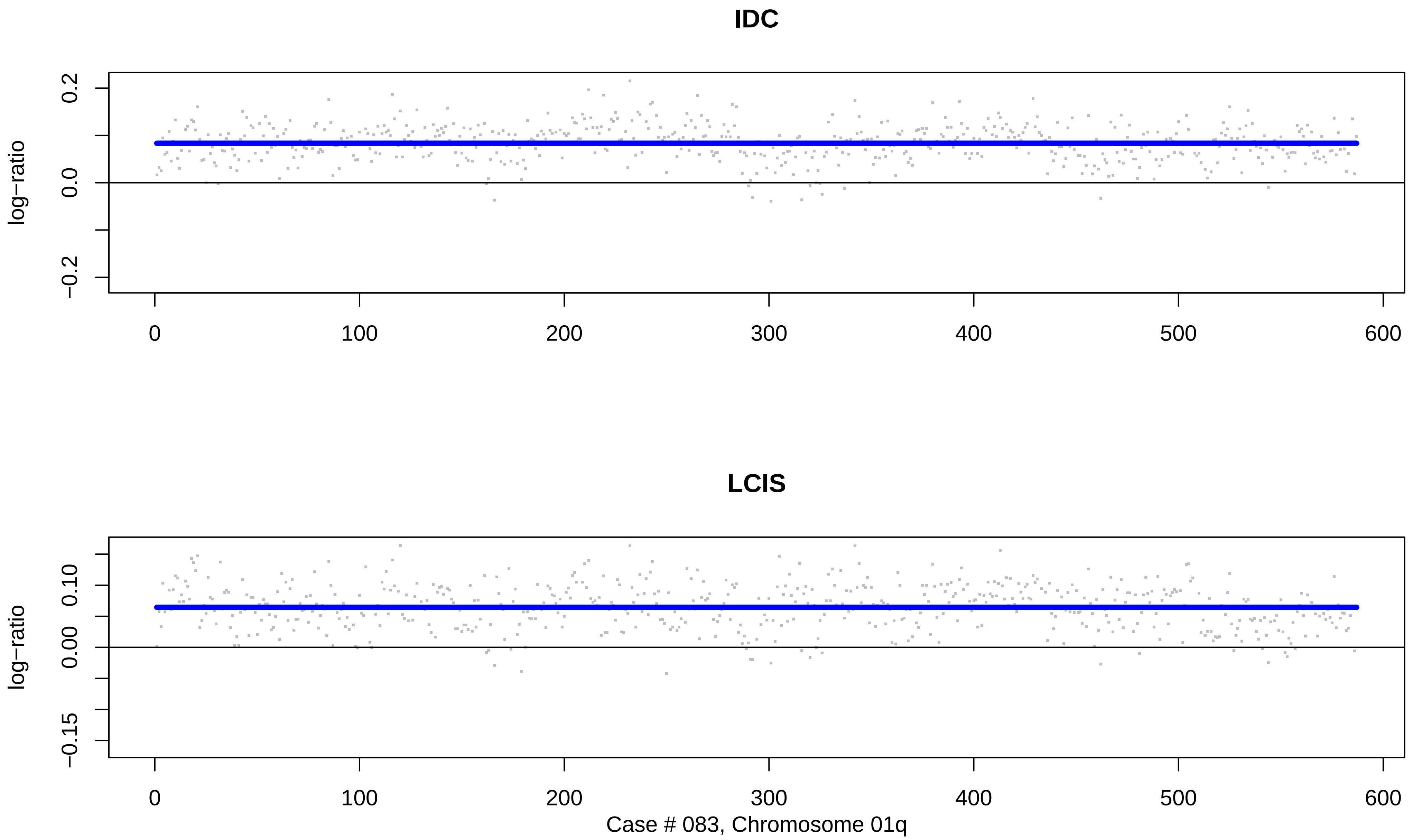

IDC
log−ratio
0.2
0.0
−0.2
0
100
200
300
400
500
600
LCIS
log−ratio
0.10
0.00
−0.15
0
100
200
300
400
500
600
Case # 083, Chromosome 01q

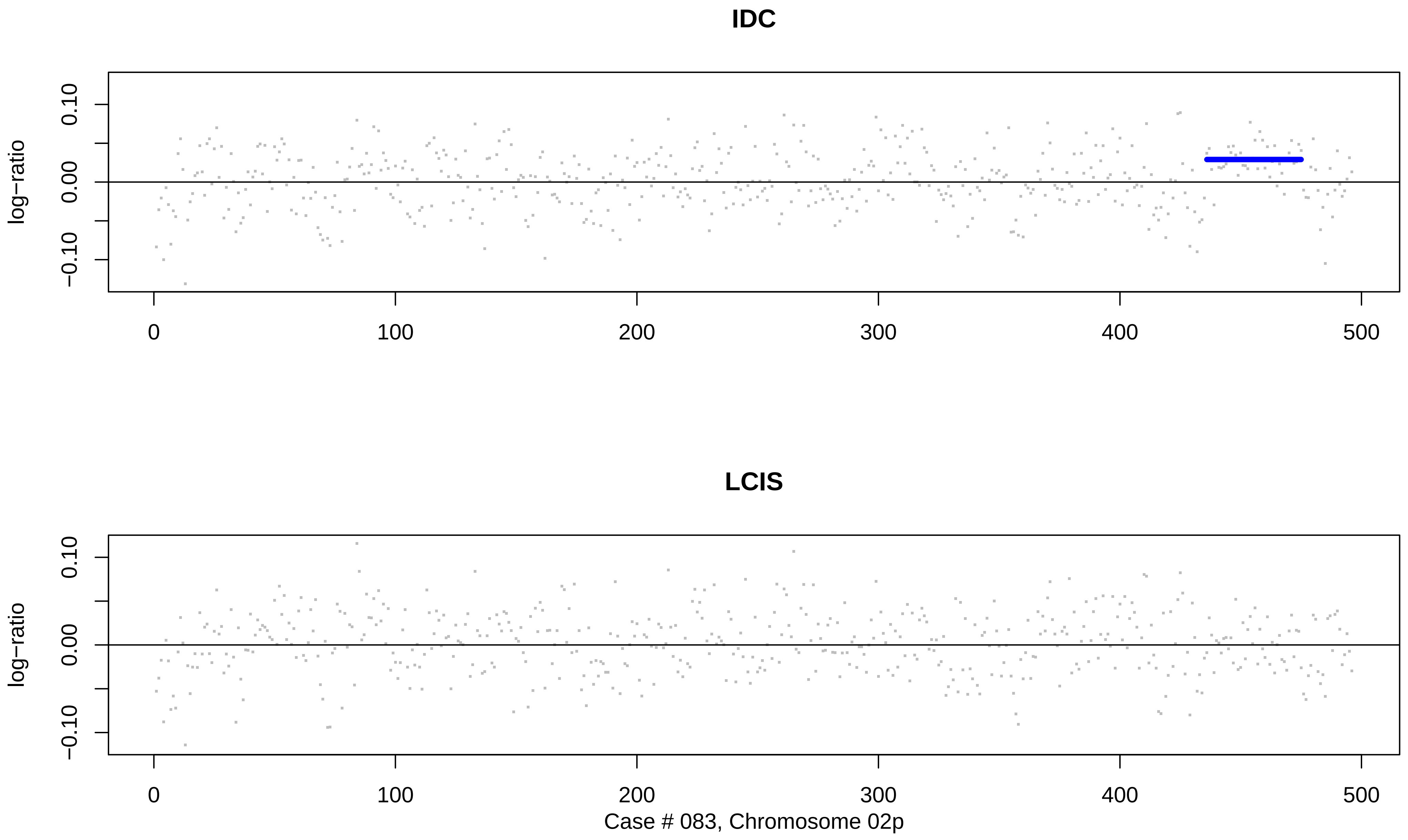
IDC
log−ratio
0.10
0.00
−0.10
0
100
200
300
400
500
LCIS
log−ratio
0.10
0.00
−0.10
0
100
200
300
400
500
Case # 083, Chromosome 02p

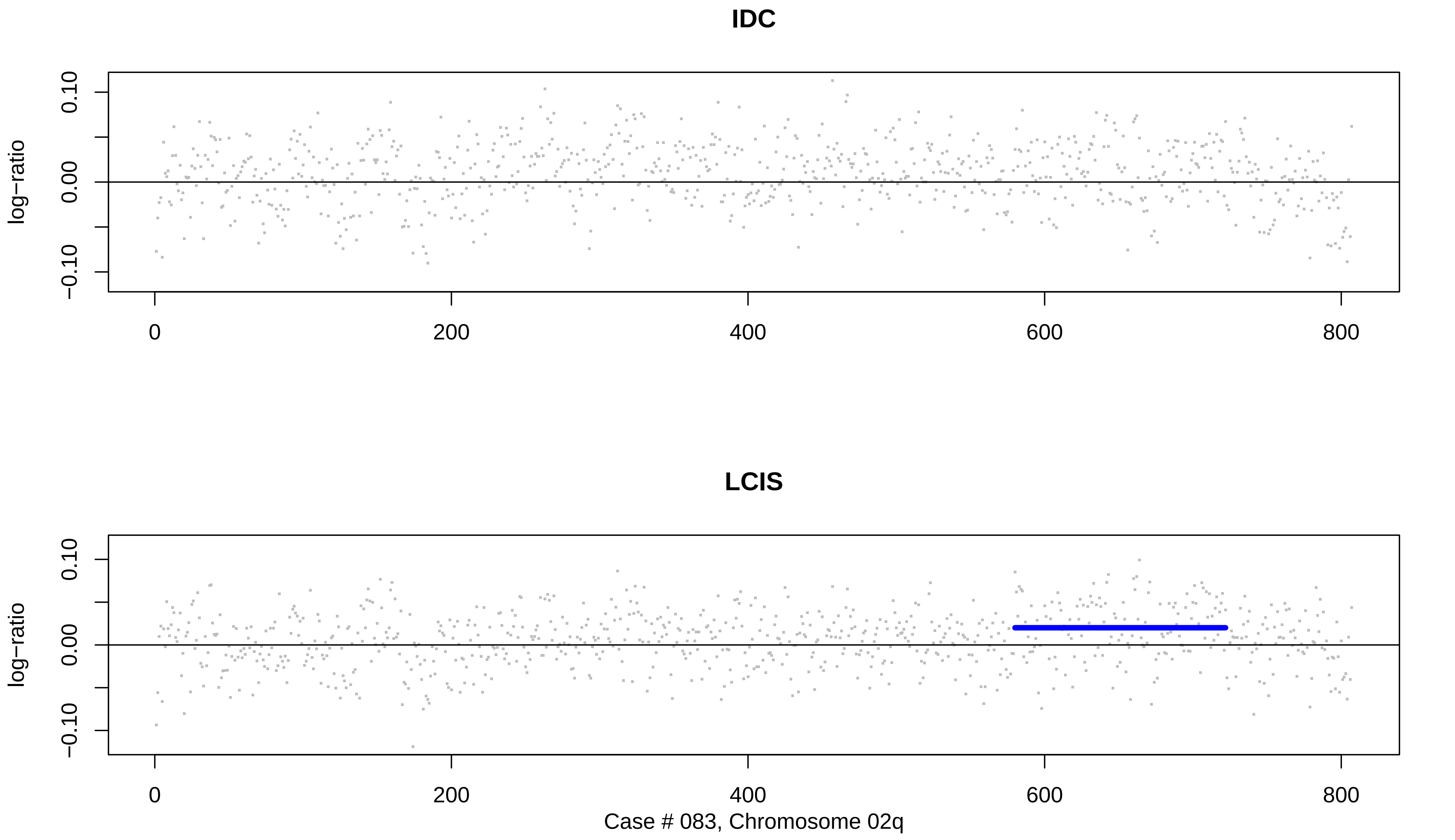
IDC
log−ratio
0.10
0.00
−0.10
0
200
400
600
800
LCIS
log−ratio
0.10
0.00
−0.10
0
200
400
600
800
Case # 083, Chromosome 02q

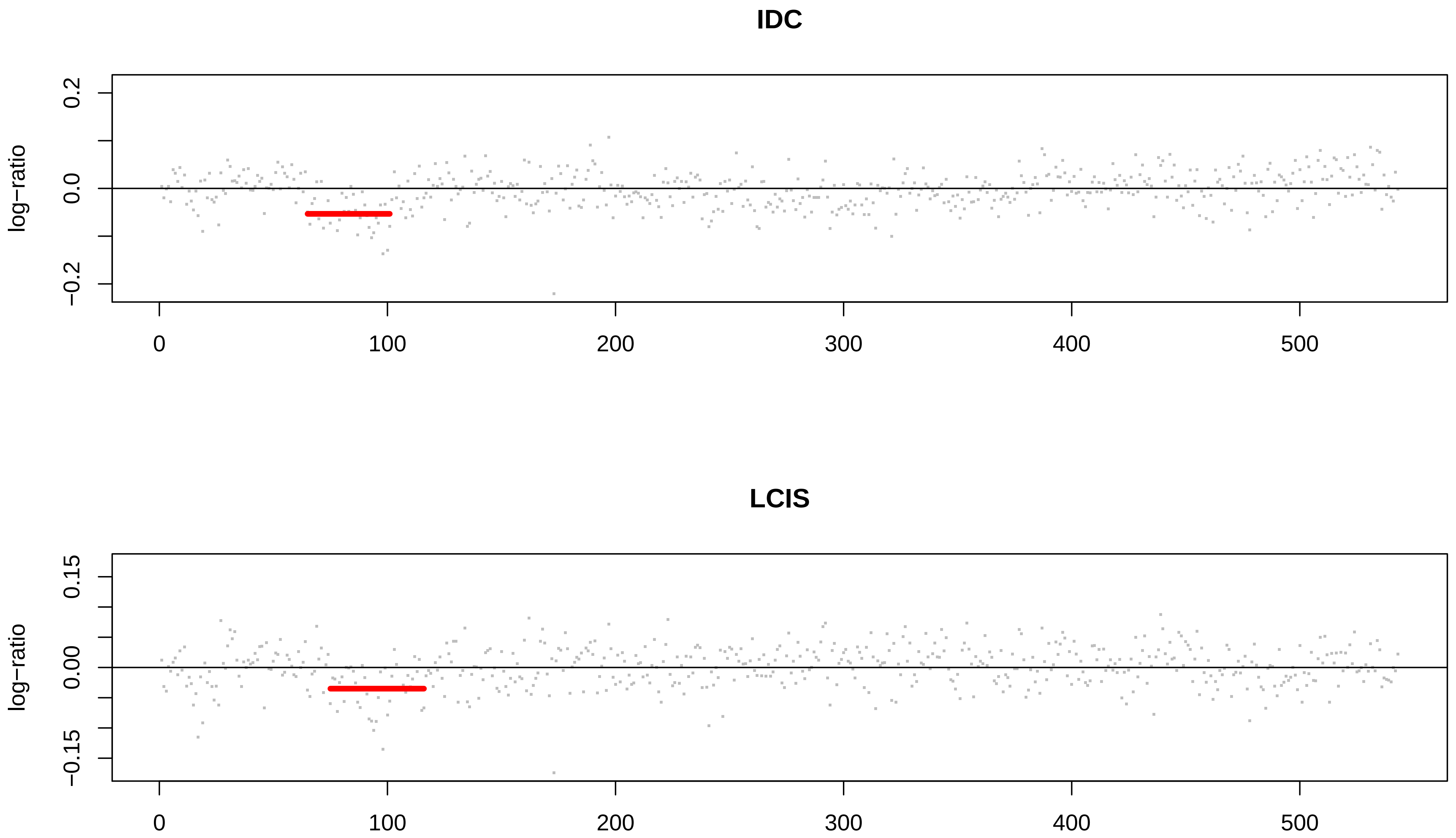

Case # 083, Chromosome 03p
Odds in favor of independence = 1.6

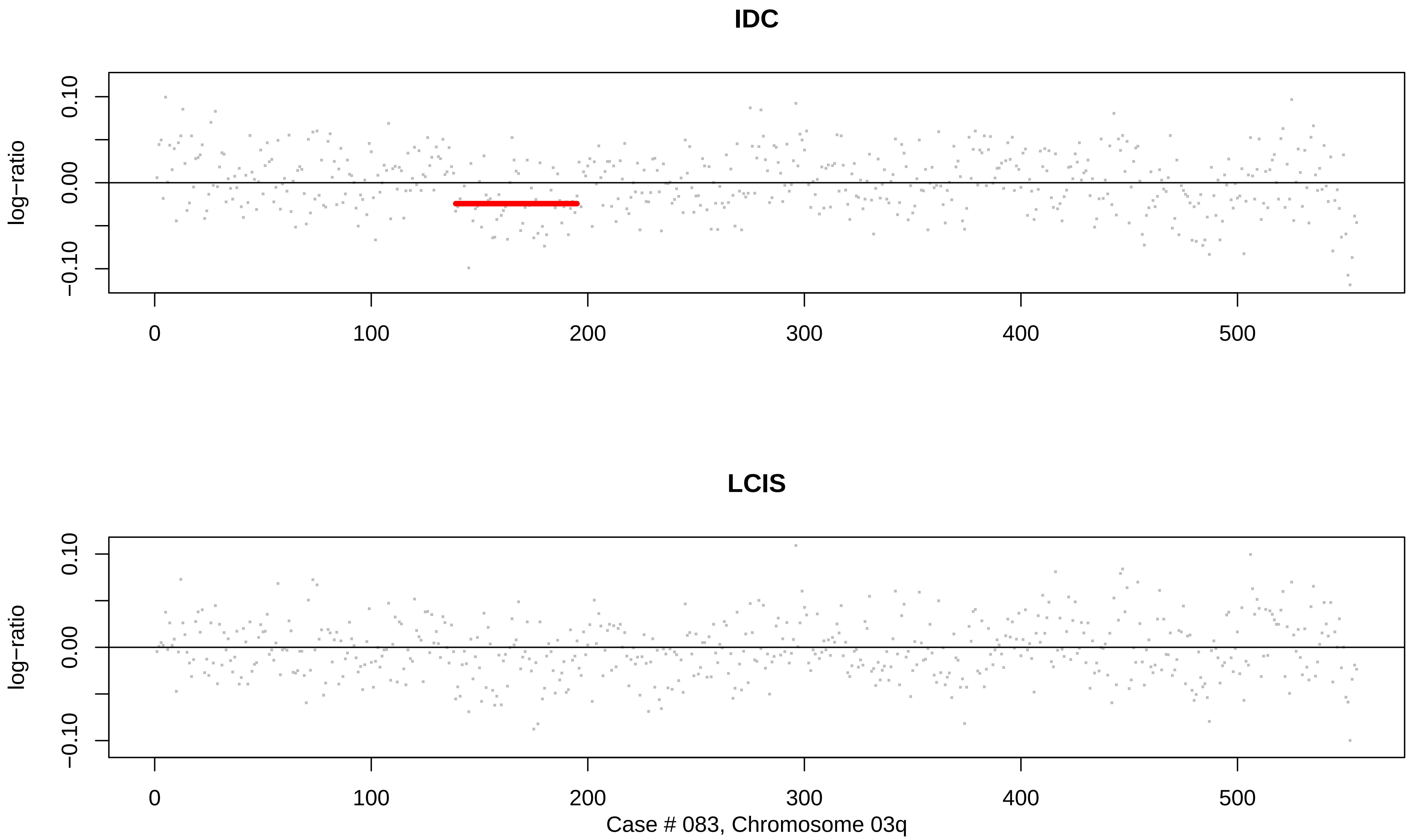
IDC
log−ratio
0.10
0.00
−0.10
0
100
200
300
400
500
LCIS
log−ratio
0.10
0.00
−0.10
0
100
200
300
400
500
Case # 083, Chromosome 03q

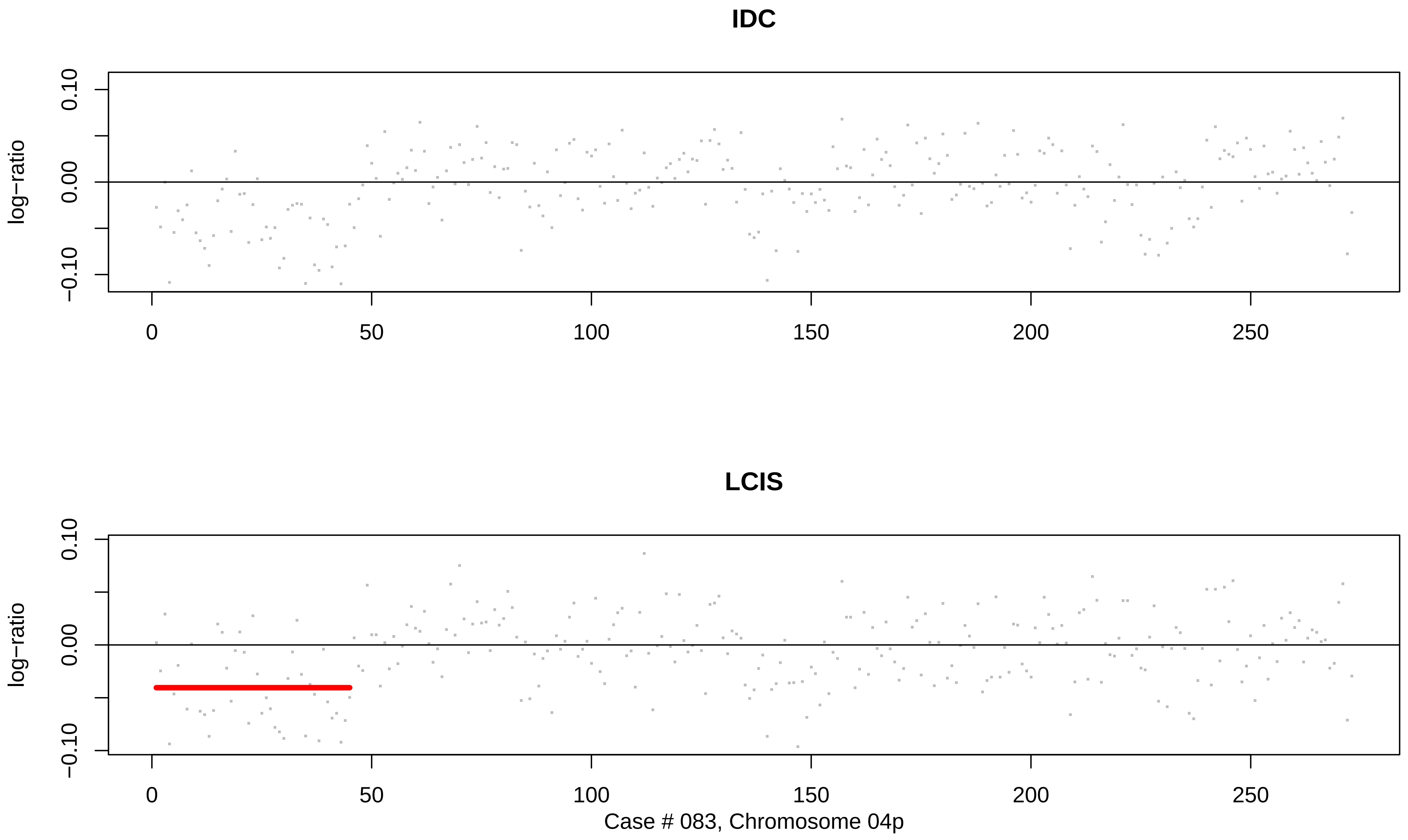
IDC
log−ratio
0.10
0.00
−0.10
0
50
100
150
200
250
LCIS
log−ratio
0.10
0.00
−0.10
0
50
100
150
200
250
Case # 083, Chromosome 04p

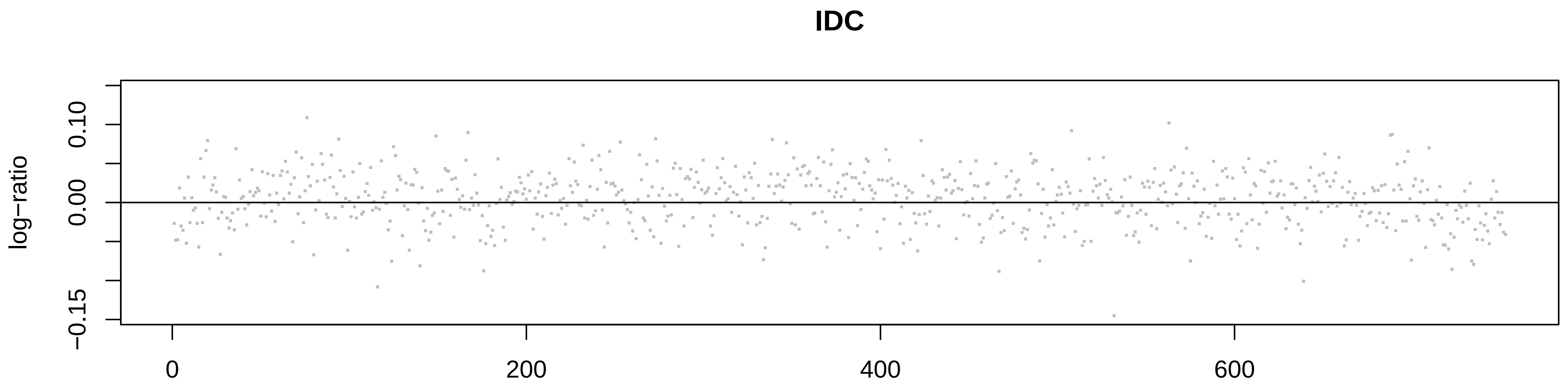
IDC
log−ratio
0.10
0.05
0.00
−0.15
0
200
400
600

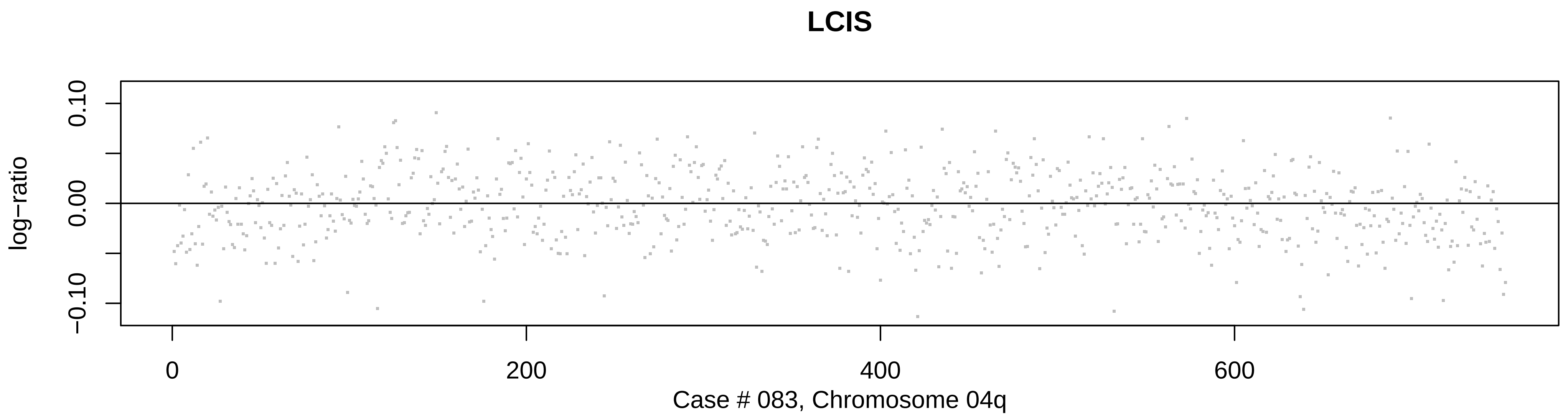
LCIS
log−ratio
0.10
0.00
−0.10
0
200
400
600
Case # 083, Chromosome 04q

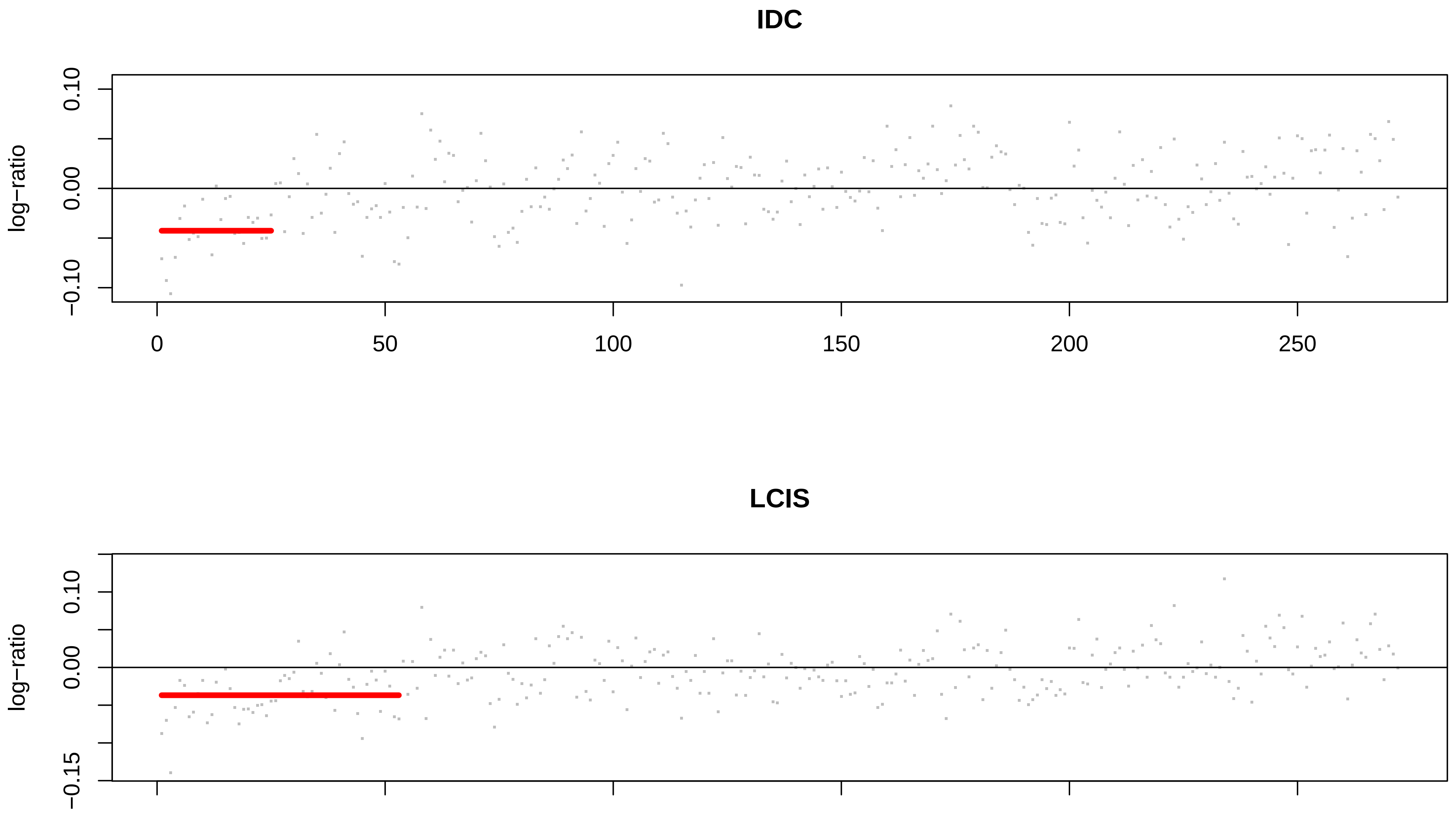

Case # 083, Chromosome 05p
Odds in favor of independence = 6.2

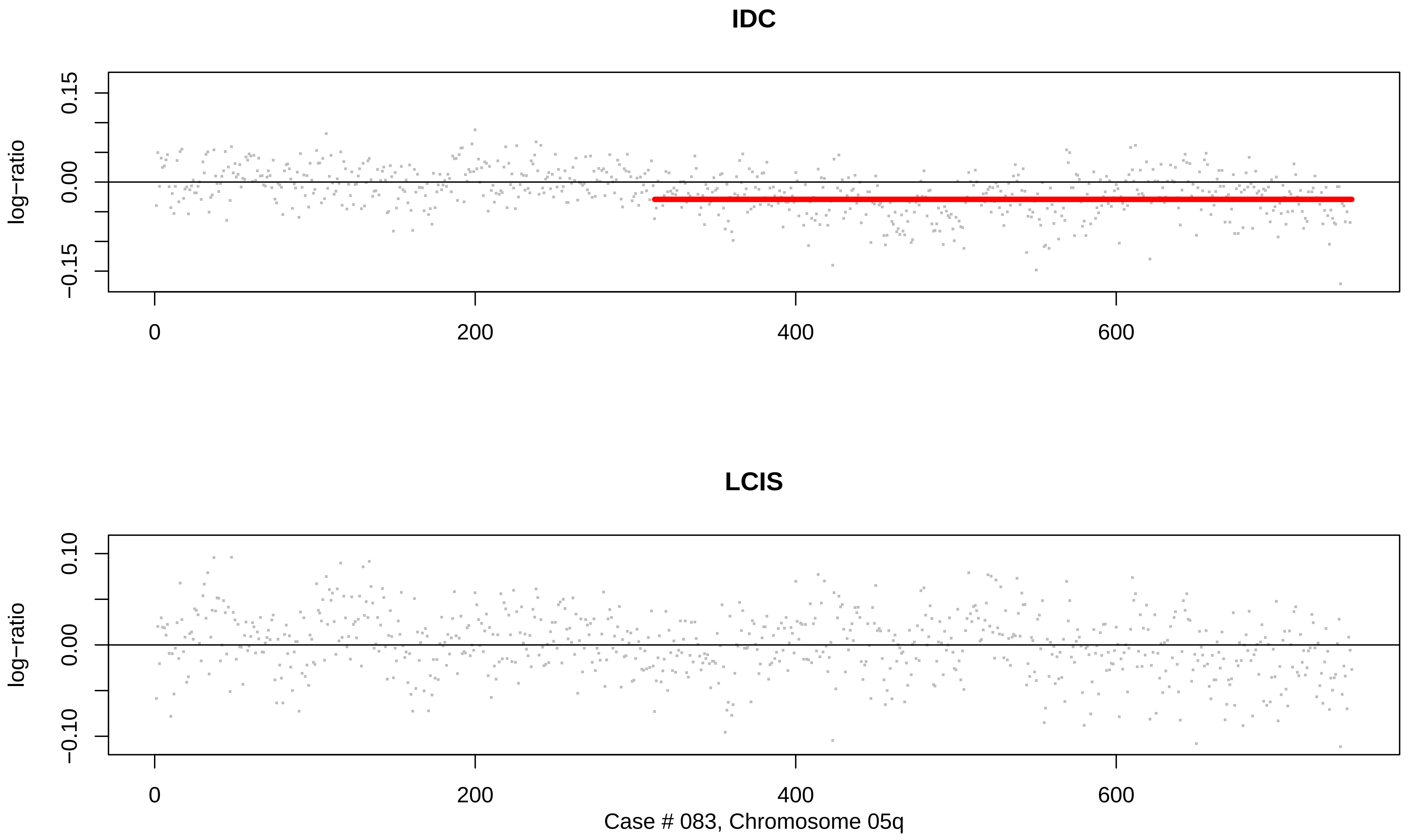
IDC
log−ratio
0.15
0.00
−0.15
0
200
400
600
LCIS
log−ratio
0.10
0.00
−0.10
0
200
400
600
Case # 083, Chromosome 05q

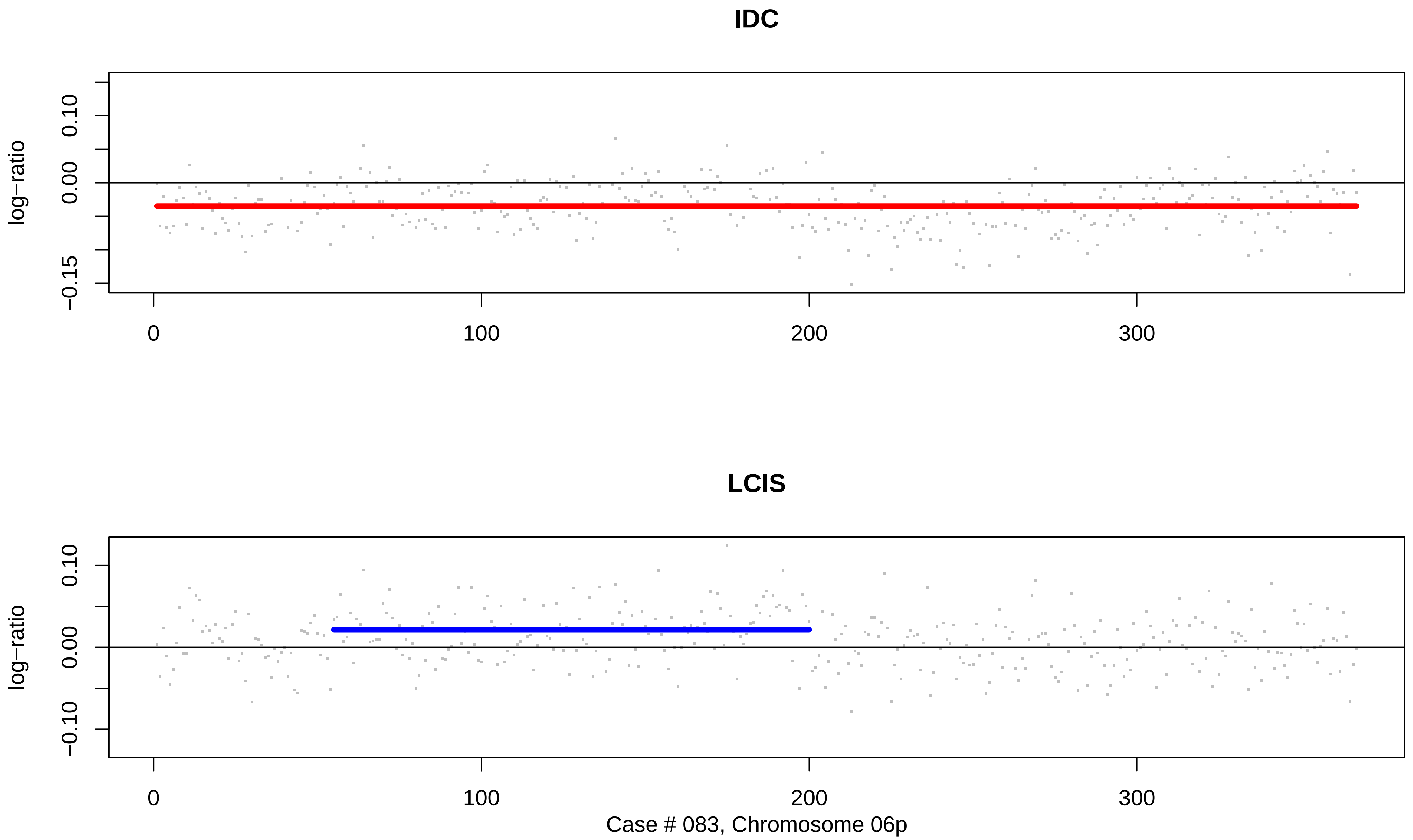
IDC
log−ratio
0.10
0.00
−0.15
0
100
200
300
LCIS
log−ratio
0.10
0.00
−0.10
0
100
200
300
Case # 083, Chromosome 06p

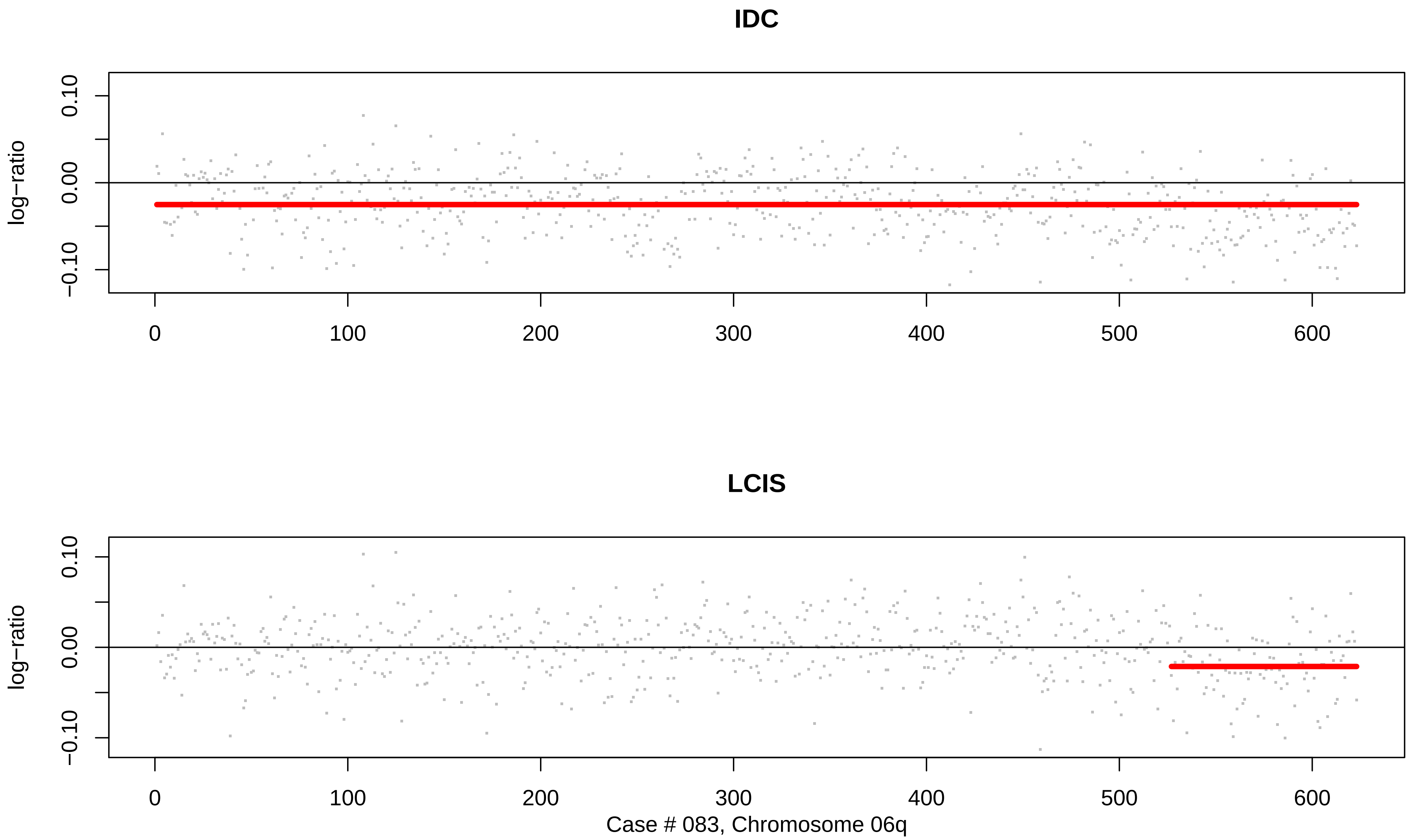
IDC
log−ratio
0.10
0.00
−0.10
0
100
200
300
400
500
600
LCIS
log−ratio
0.10
0.00
−0.10
0
100
200
300
400
500
600
Case # 083, Chromosome 06q

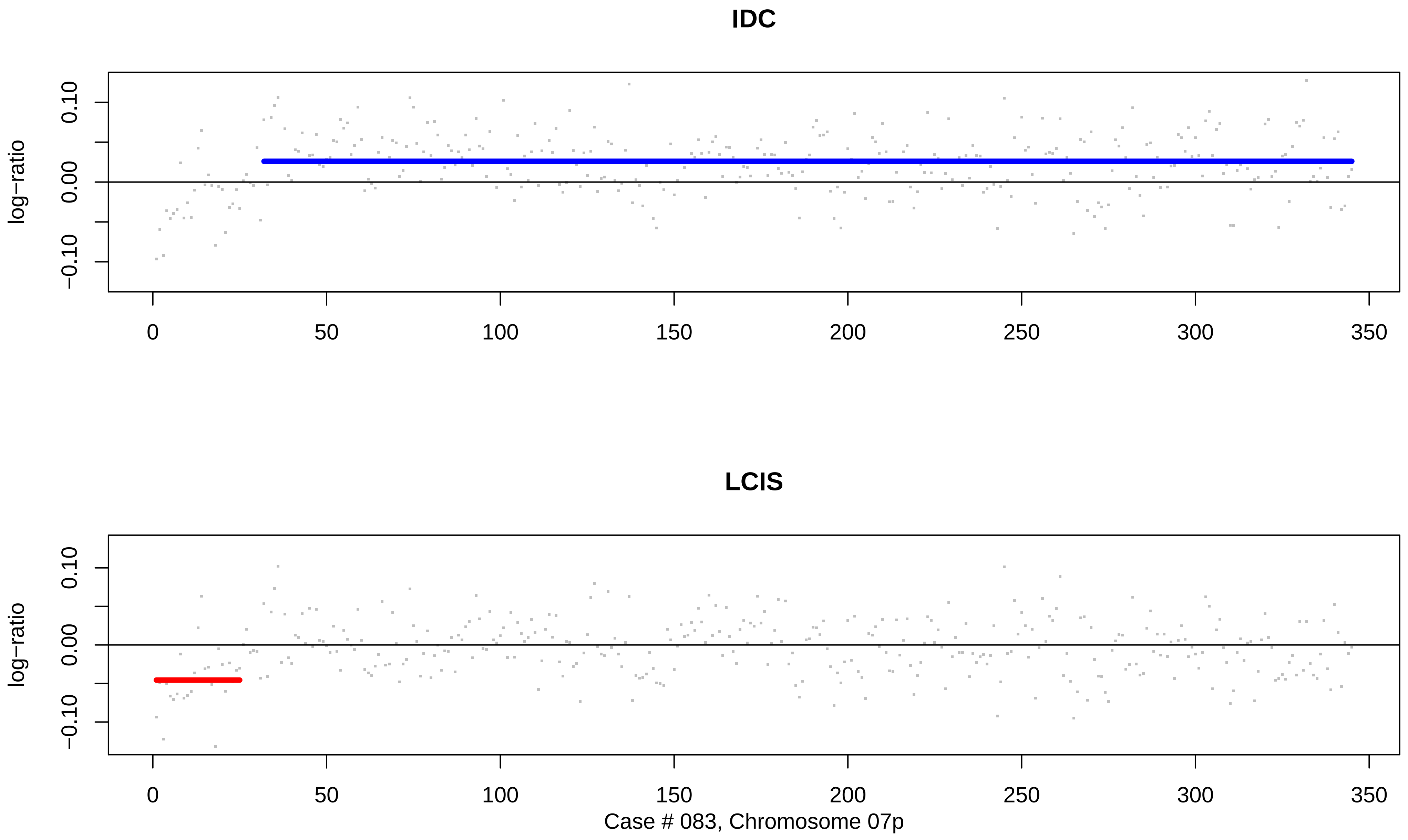

IDC
log−ratio
−0.10
0.00
0.10
0
50
100
150
200
250
300
350
LCIS
log−ratio
−0.10
0.00
0.10
0
50
100
150
200
250
300
350
Case # 083, Chromosome 07p

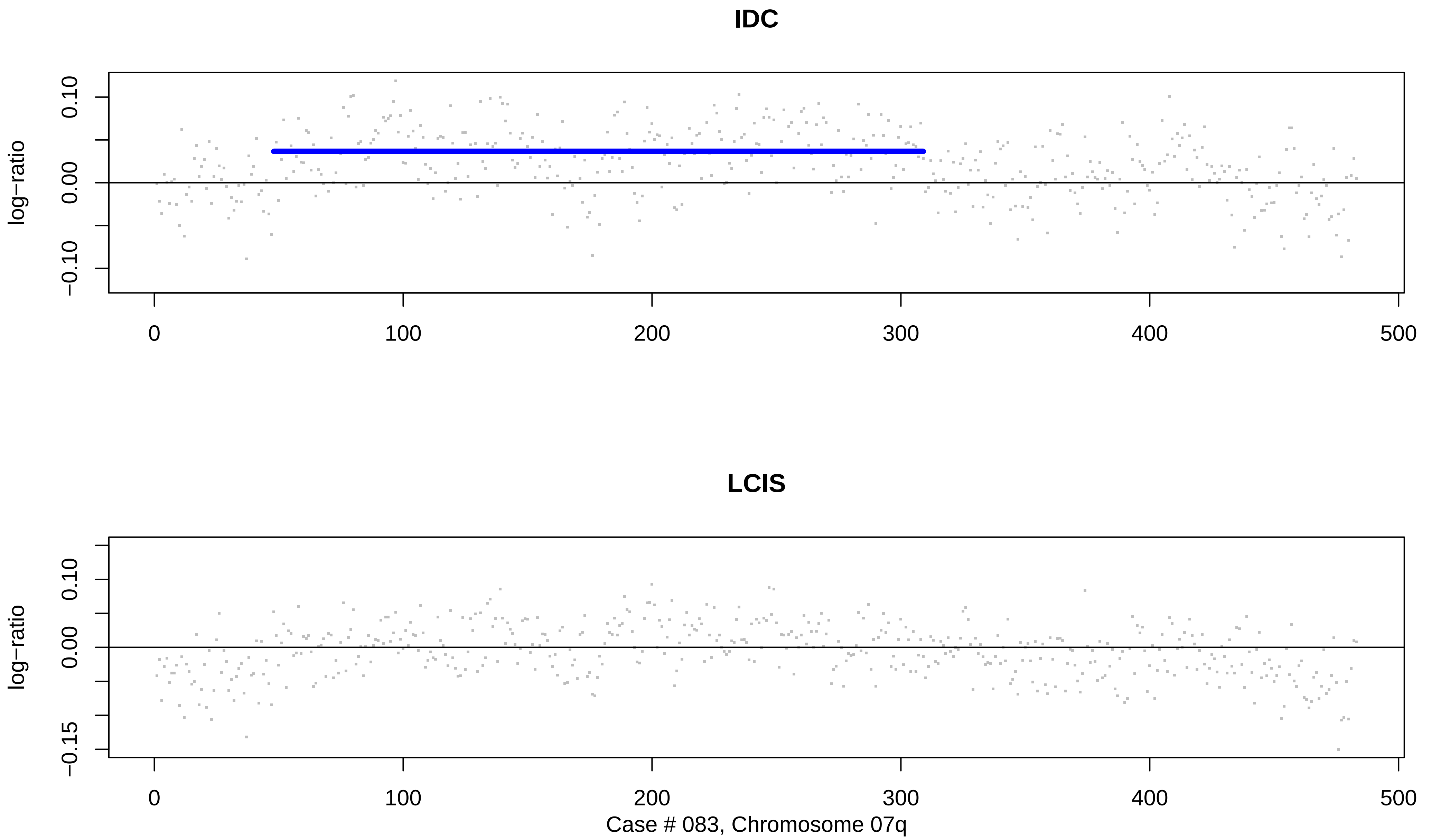
IDC
log−ratio
−0.10
0.00
0.10
0
100
200
300
400
500
LCIS
log−ratio
−0.15
0.00
0.10
0
100
200
300
400
500
Case # 083, Chromosome 07q

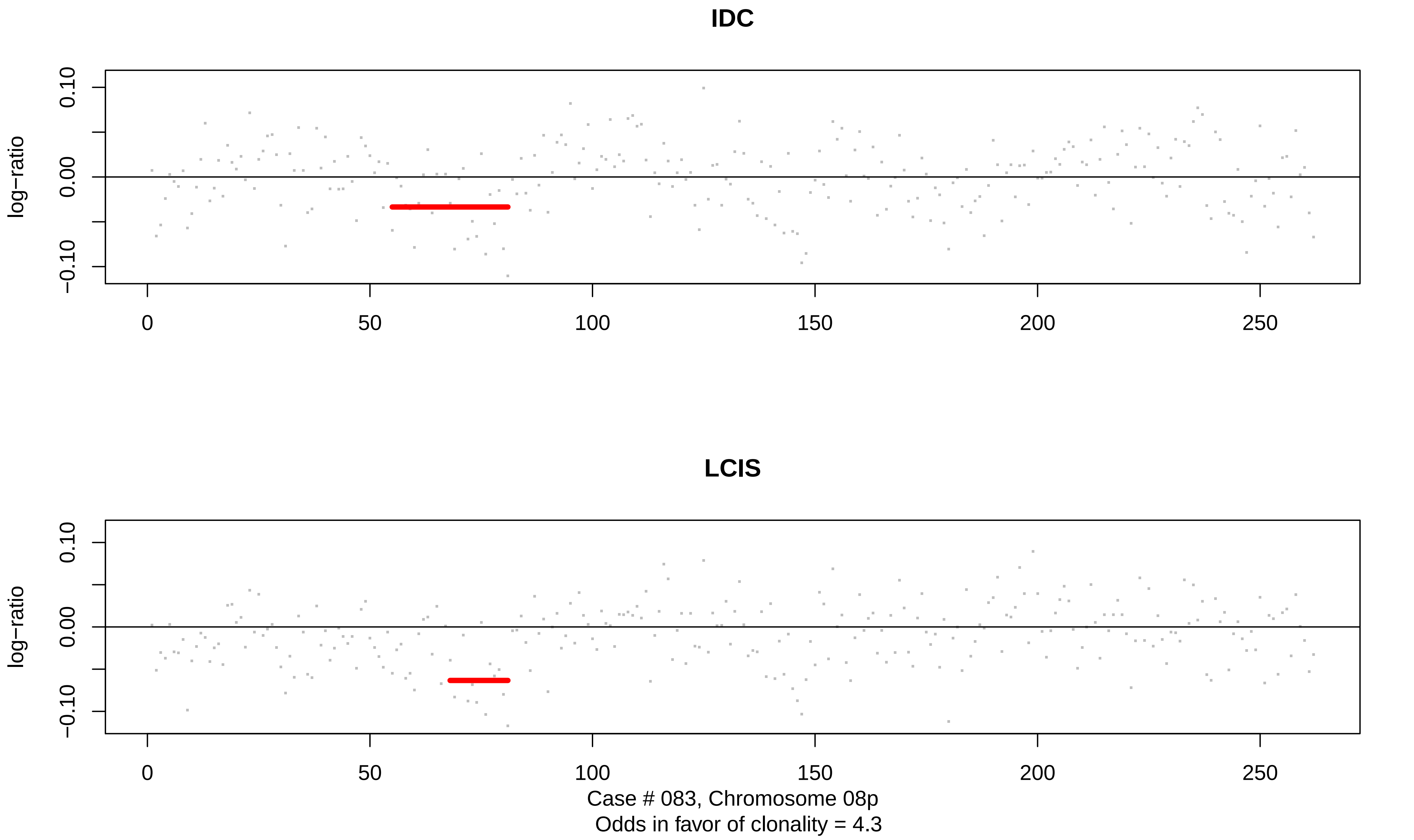
IDC
LCIS
log−ratio
0.10
0.00
−0.10
0
50
100
150
200
250
Case # 083, Chromosome 08p
Odds in favor of clonality = 4.3

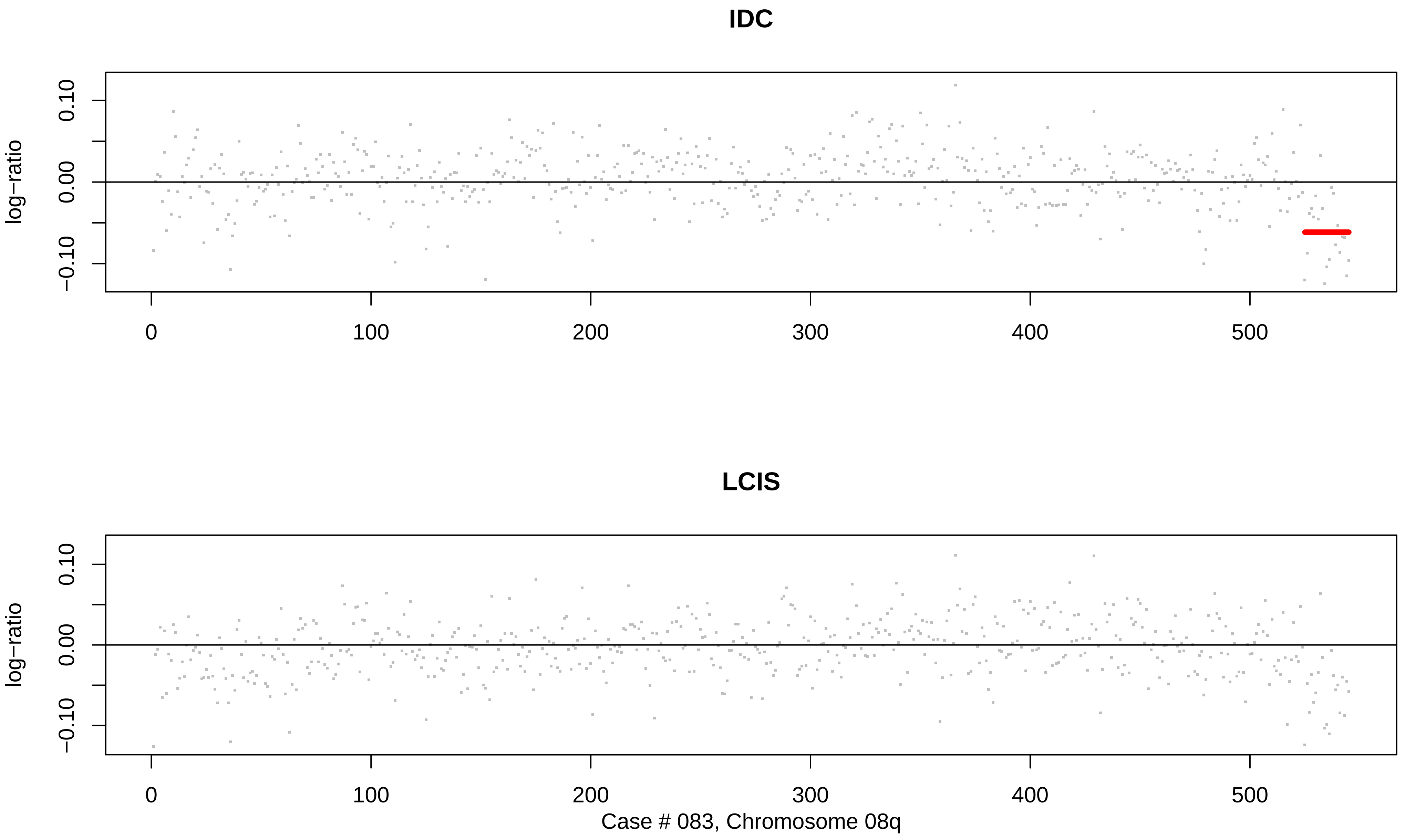
IDC
log−ratio
0.10
0.00
−0.10
0
100
200
300
400
500
LCIS
log−ratio
0.10
0.00
−0.10
0
100
200
300
400
500
Case # 083, Chromosome 08q

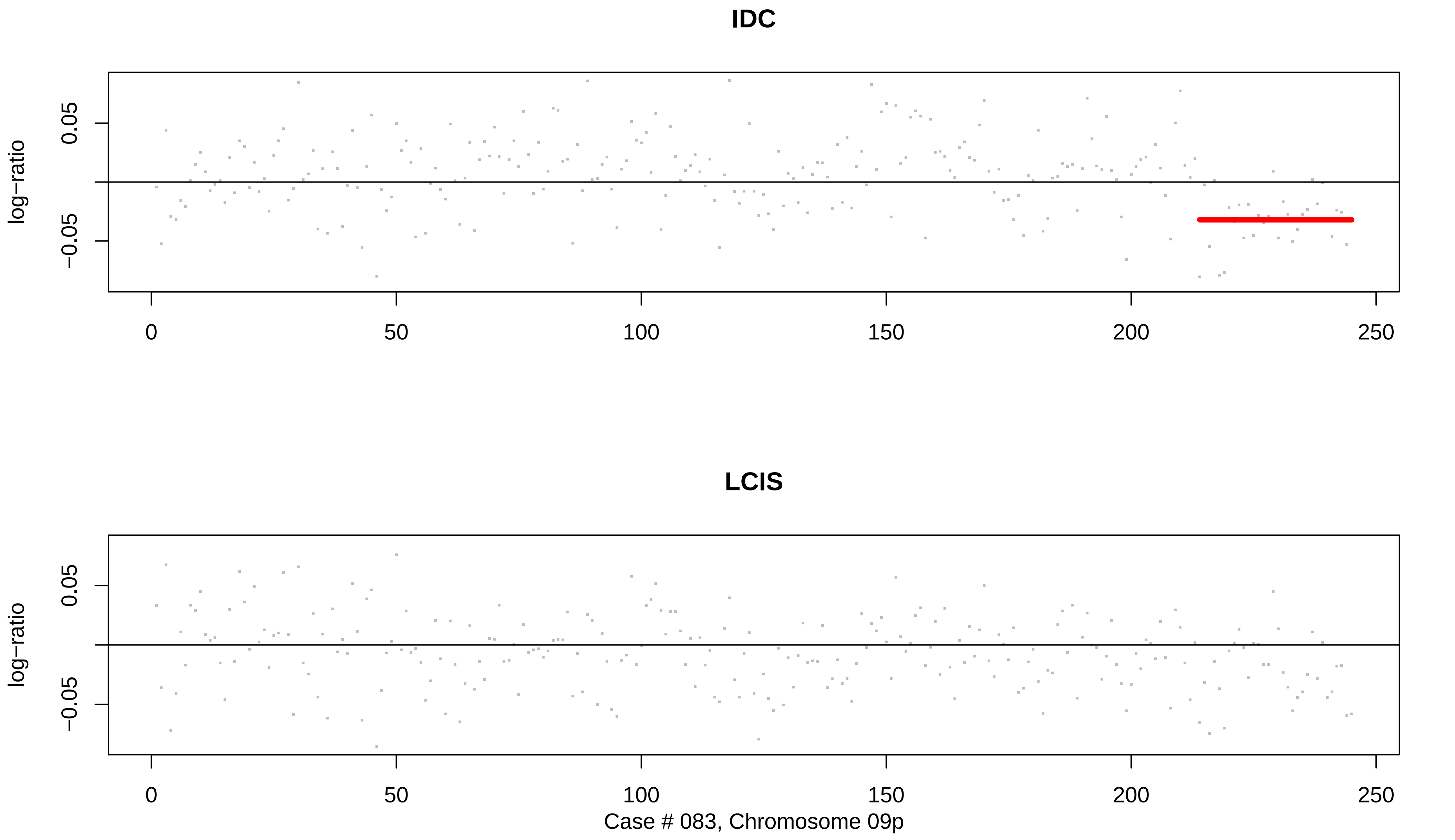
IDC
LCIS
log−ratio
0.05
−0.05
0
50
100
150
200
250
Case # 083, Chromosome 09p

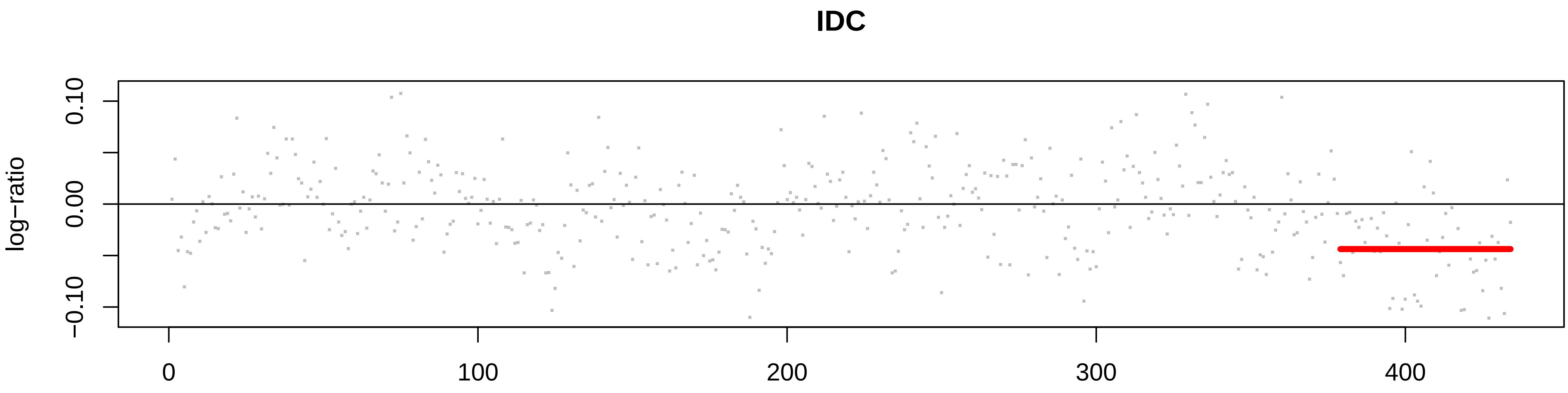

IDC
log−ratio
0.10
0.00
−0.10
0
100
200
300
400

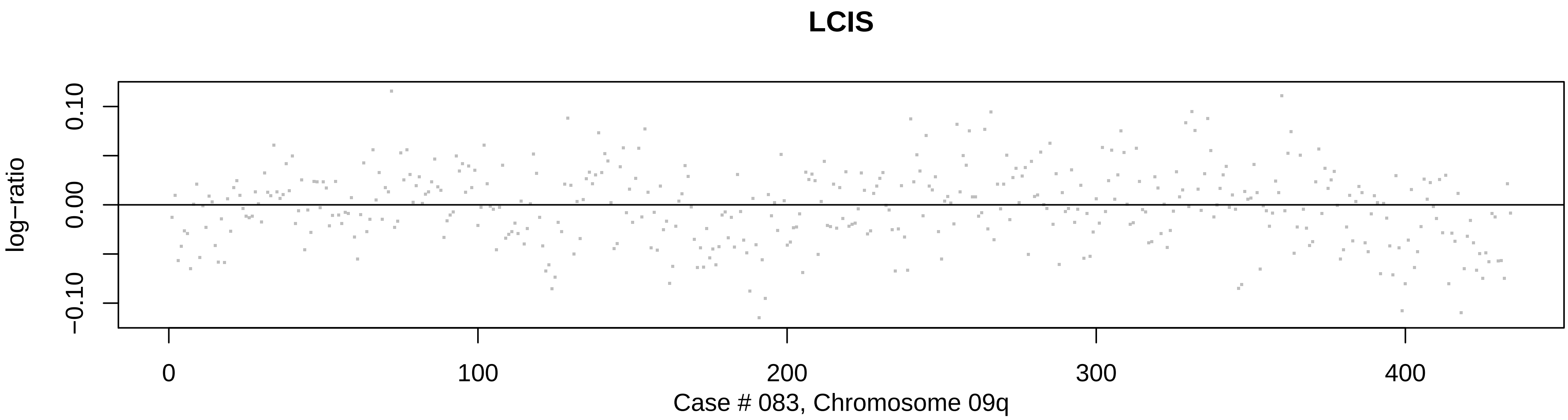

LCIS
log−ratio
0.10
0.00
−0.10
0
100
200
300
400
Case # 083, Chromosome 09q

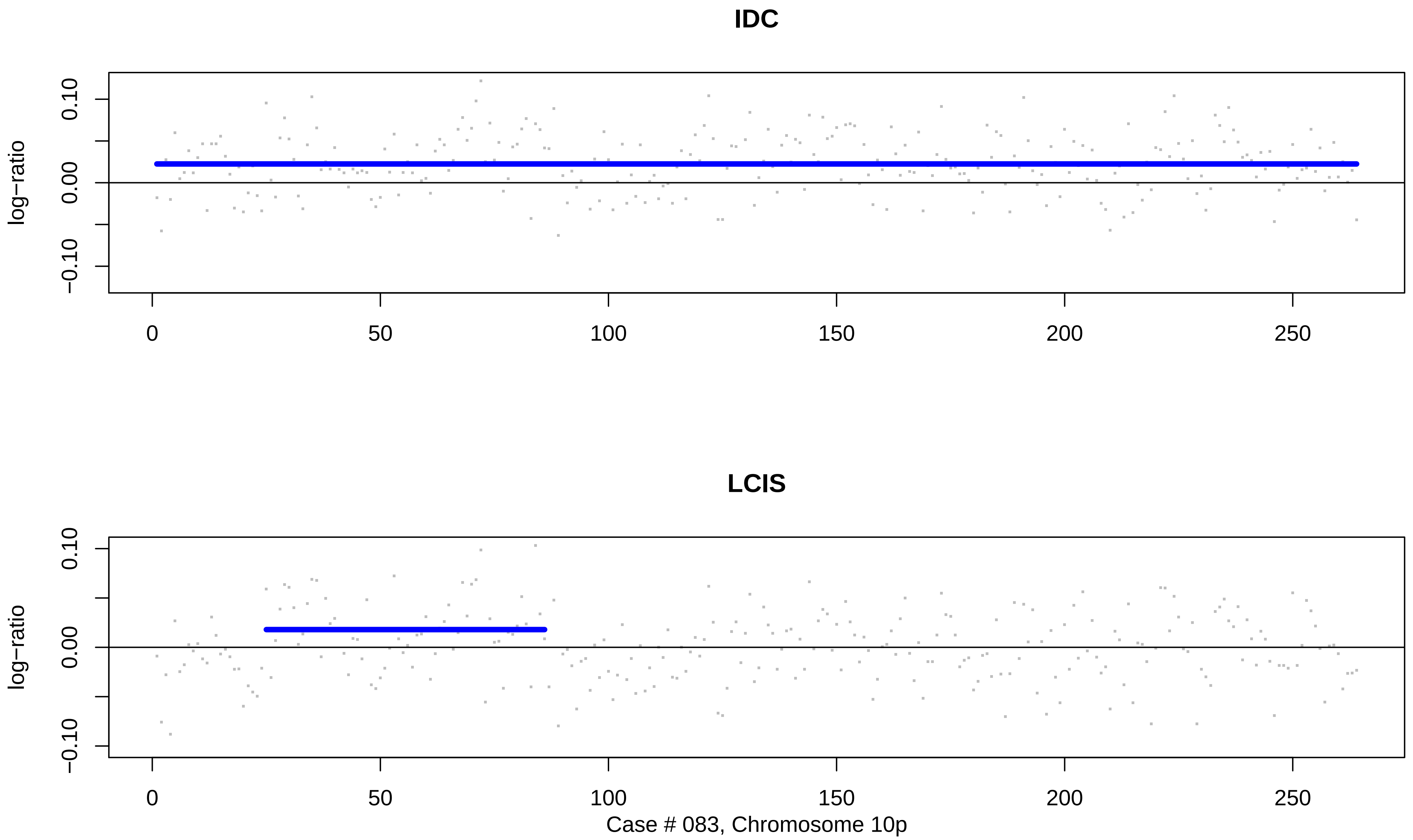
IDC
log−ratio
0.10
0.00
−0.10
0
50
100
150
200
250
LCIS
log−ratio
0.10
0.00
−0.10
0
50
100
150
200
250
Case # 083, Chromosome 10p

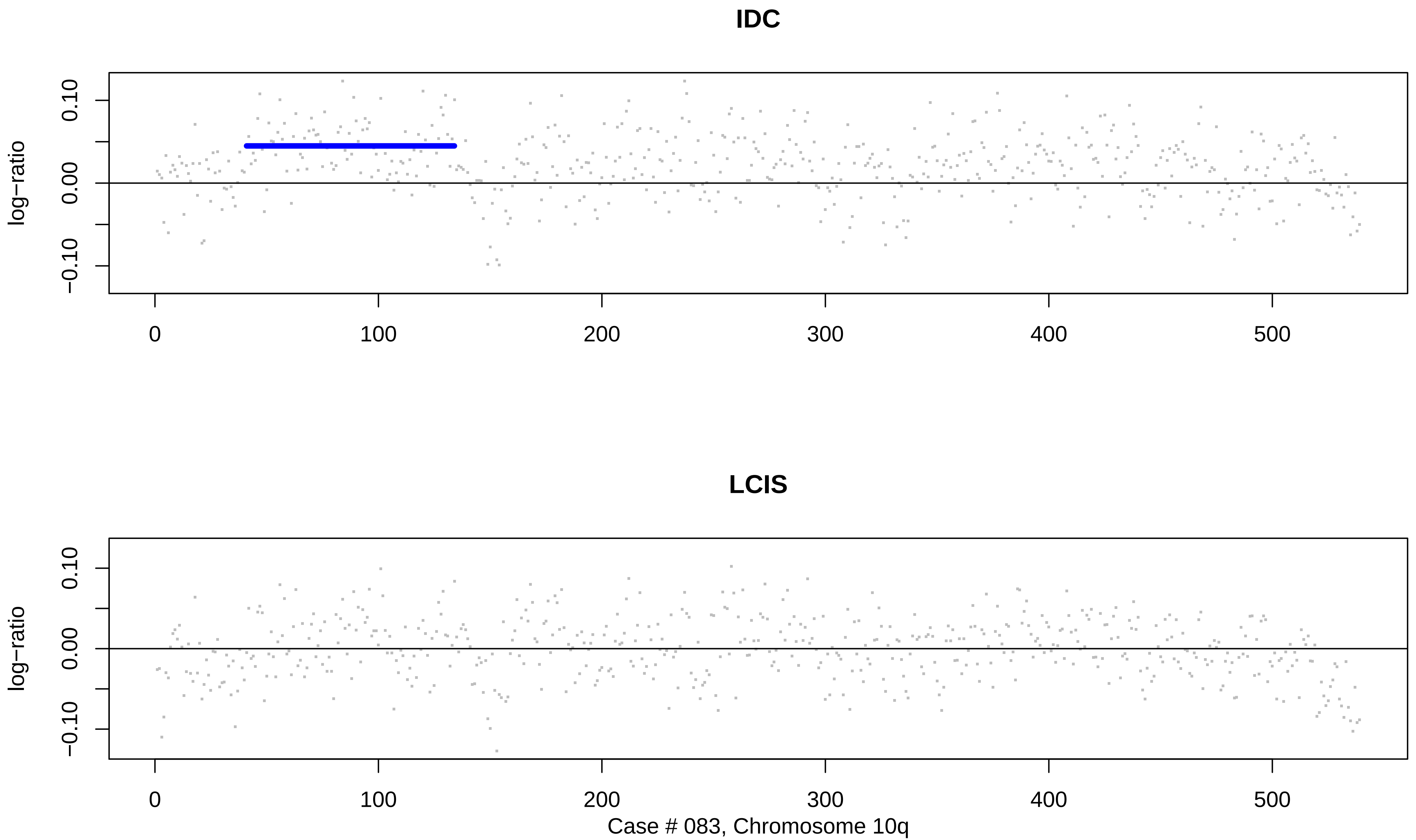
IDC
log−ratio
0.10
0.00
−0.10
0
100
200
300
400
500
LCIS
log−ratio
0.10
0.00
−0.10
0
100
200
300
400
500
Case # 083, Chromosome 10q

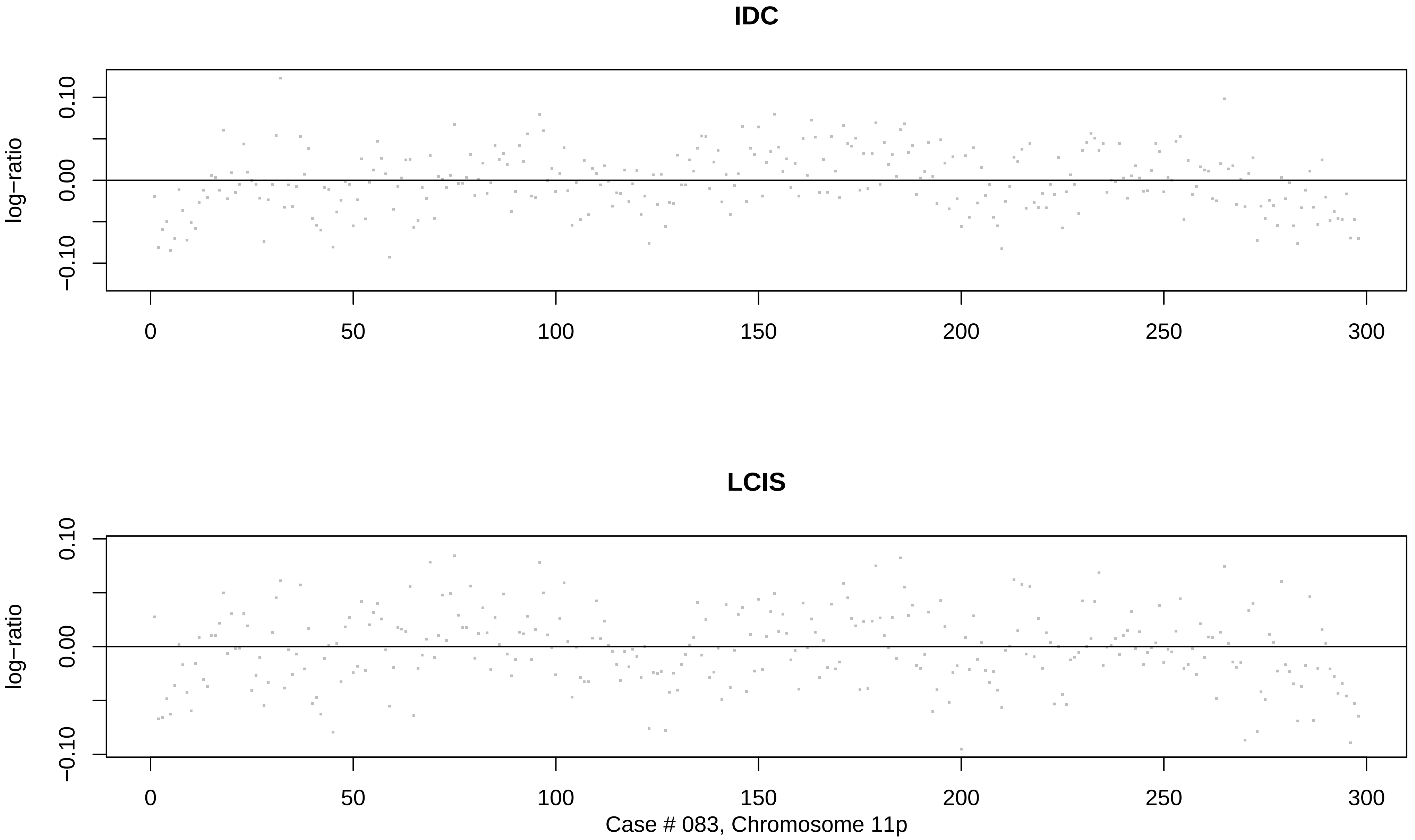
IDC
log−ratio
0.10
0.00
−0.10
0
50
100
150
200
250
300
LCIS
log−ratio
0.10
0.00
−0.10
0
50
100
150
200
250
300
Case # 083, Chromosome 11p

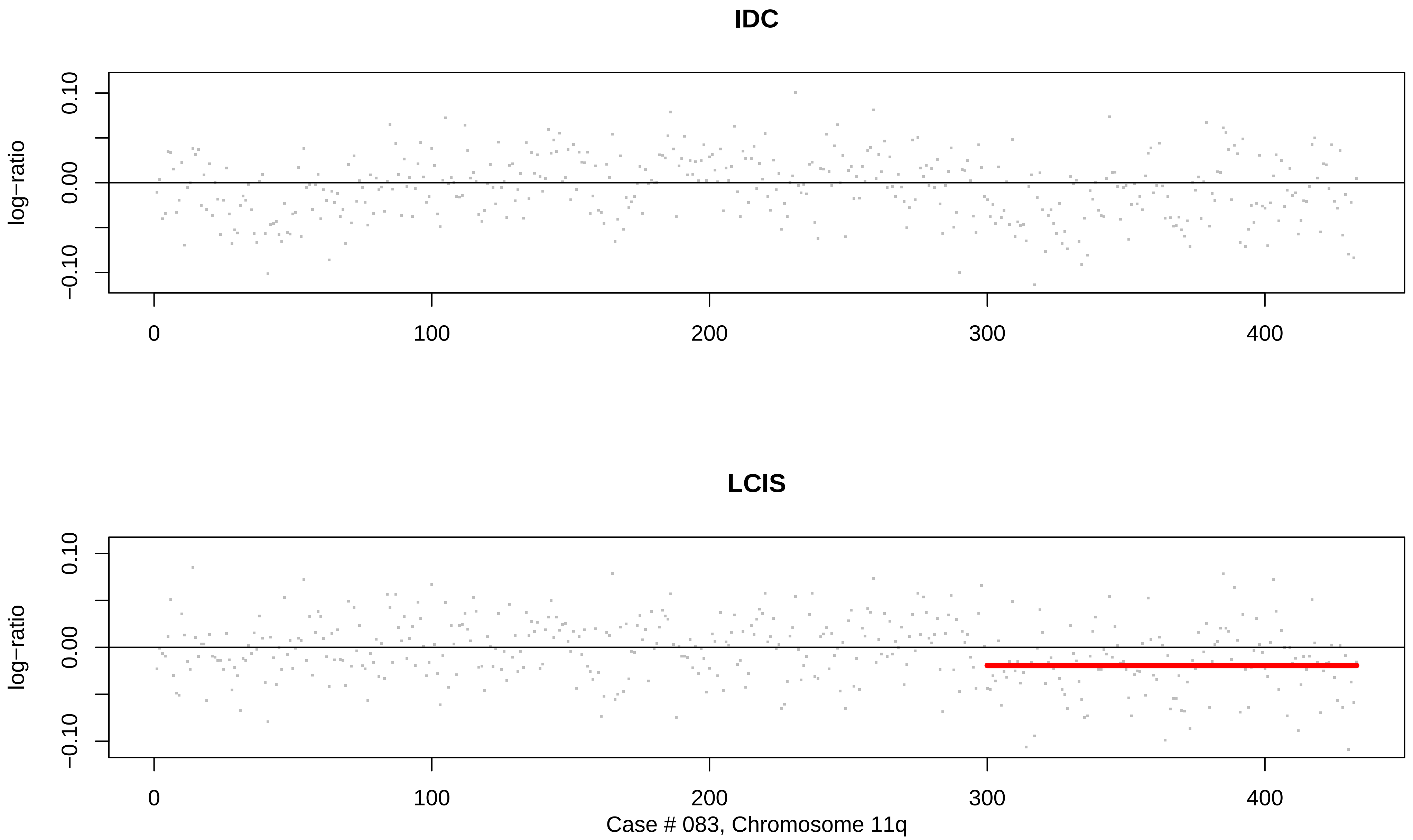
IDC
LCIS
log−ratio
0.10
0.00
−0.10
0
100
200
300
400
Case # 083, Chromosome 11q

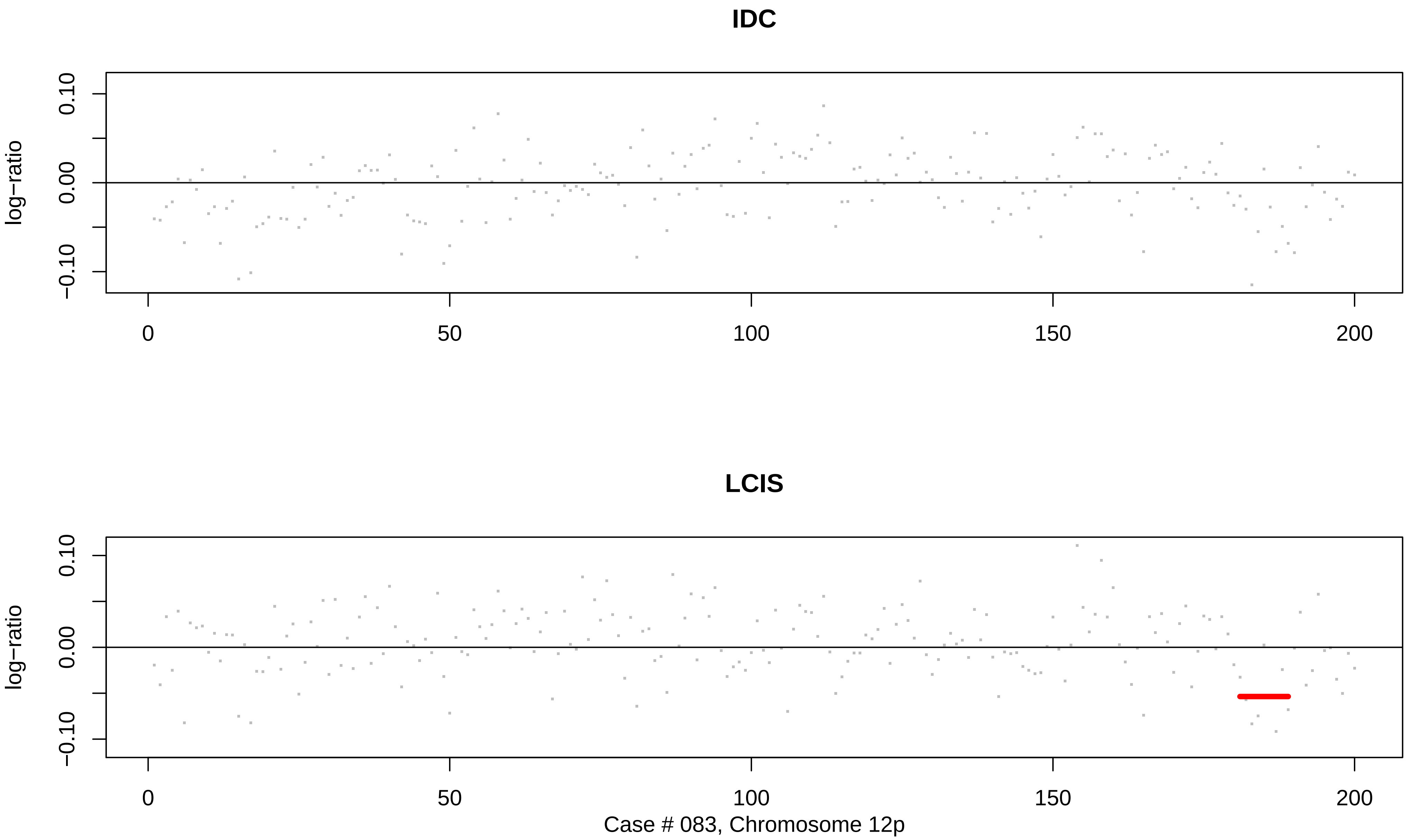
IDC
log−ratio
0.10
0.00
−0.10
0
50
100
150
200
LCIS
log−ratio
0.10
0.00
−0.10
0
50
100
150
200
Case # 083, Chromosome 12p

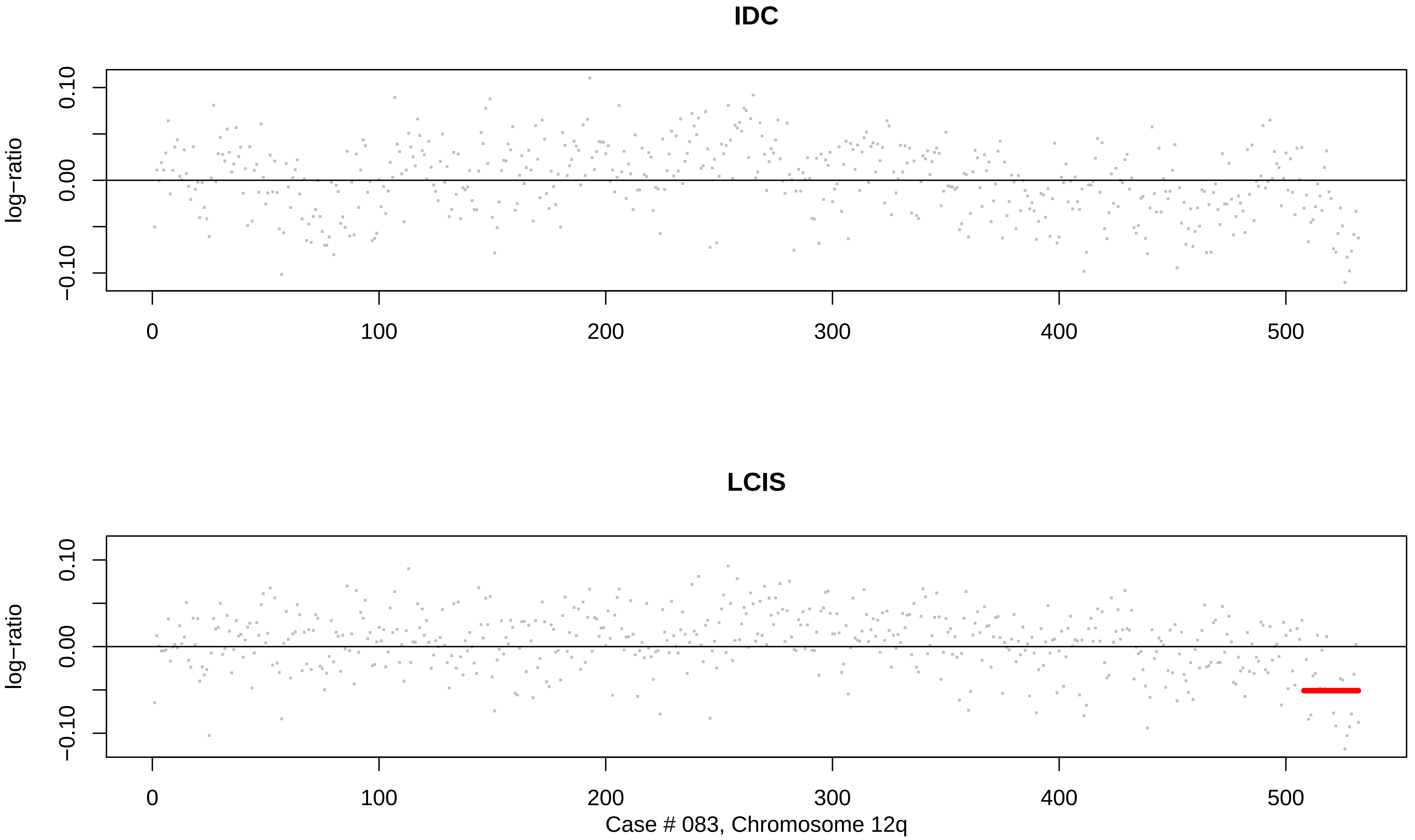
IDC
LCIS
log−ratio
0.10
0.00
−0.10
0
100
200
300
400
500
Case # 083, Chromosome 12q

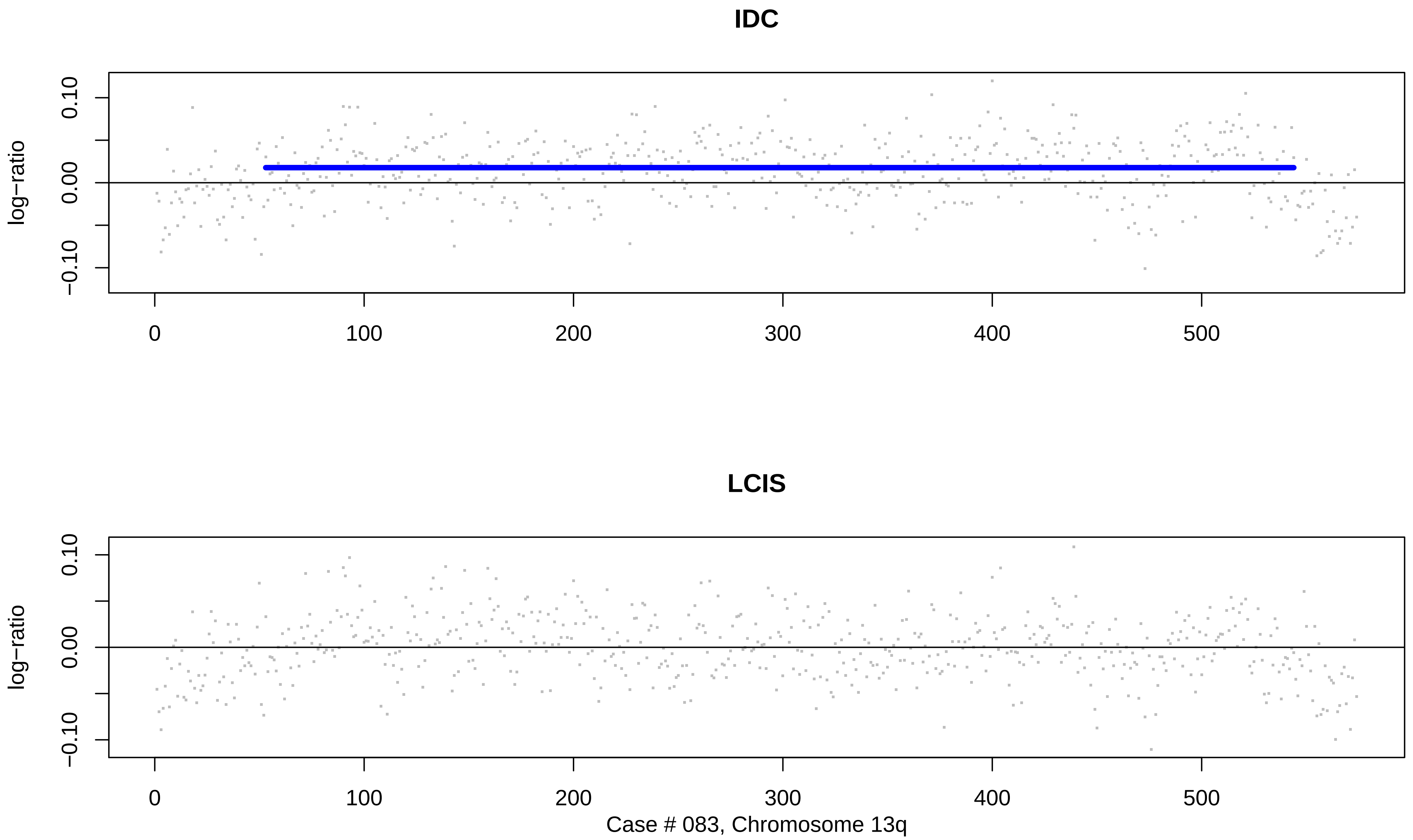
IDC
log−ratio
0.10
0.00
−0.10
0
100
200
300
400
500
LCIS
log−ratio
0.10
0.00
−0.10
0
100
200
300
400
500
Case # 083, Chromosome 13q

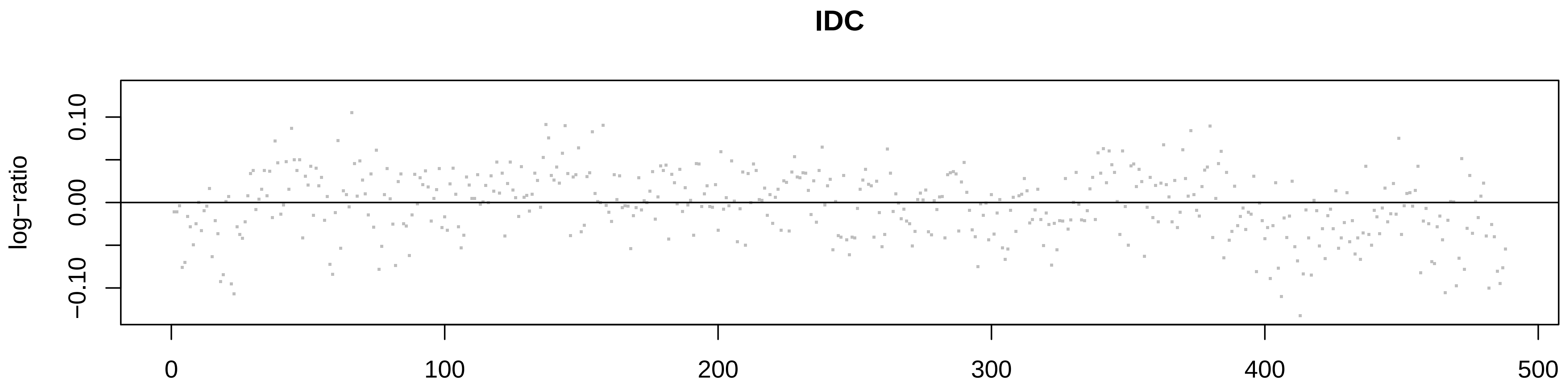
IDC
log−ratio
0.10
0.00
−0.10
0
100
200
300
400
500

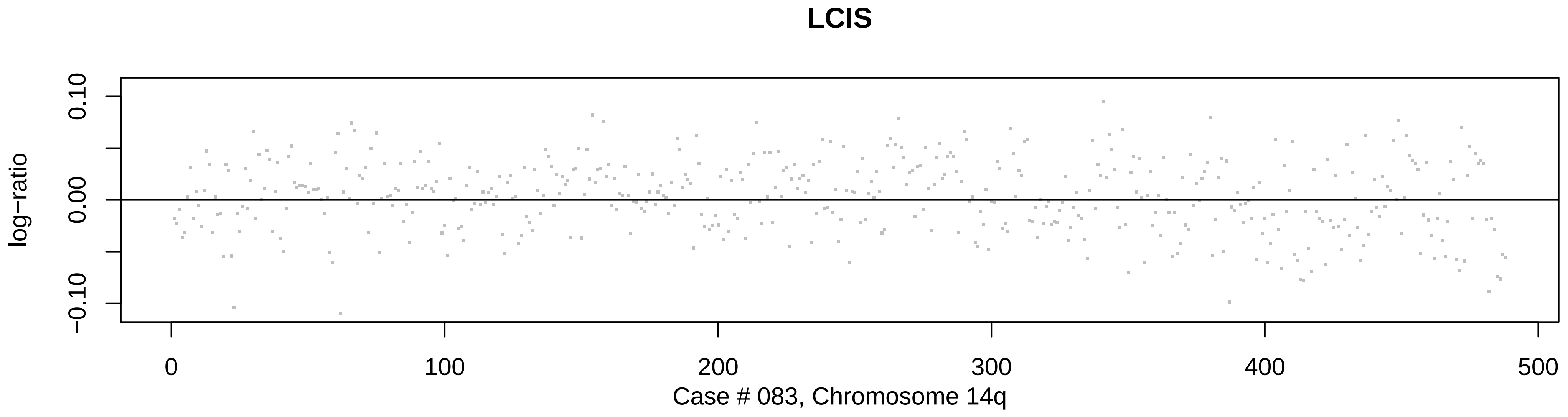
LCIS
log−ratio
0.10
0.00
−0.10
0
100
200
300
400
500
Case # 083, Chromosome 14q

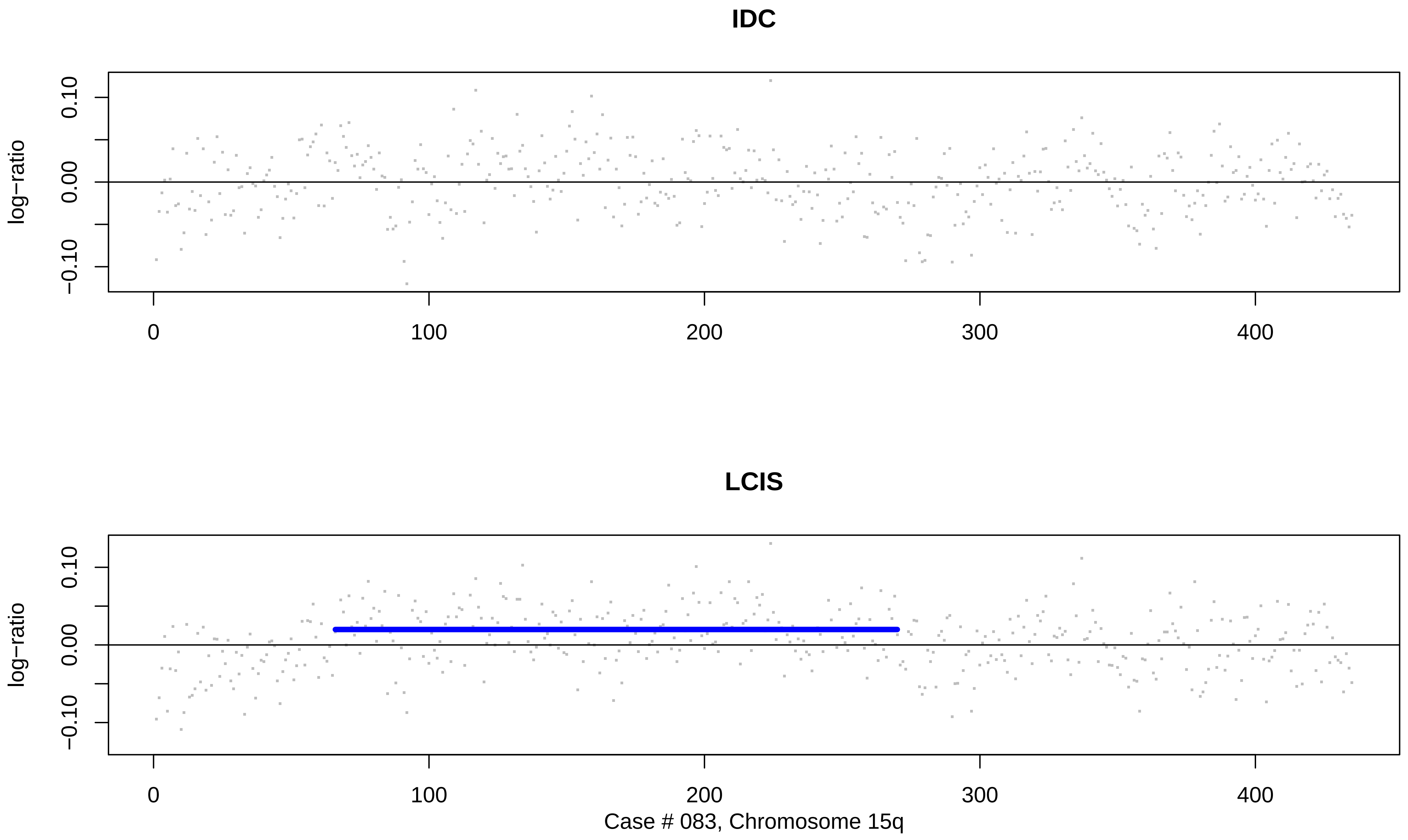
IDC
log−ratio
0.10
0.00
−0.10
0
100
200
300
400
LCIS
log−ratio
0.10
0.00
−0.10
0
100
200
300
400
Case # 083, Chromosome 15q

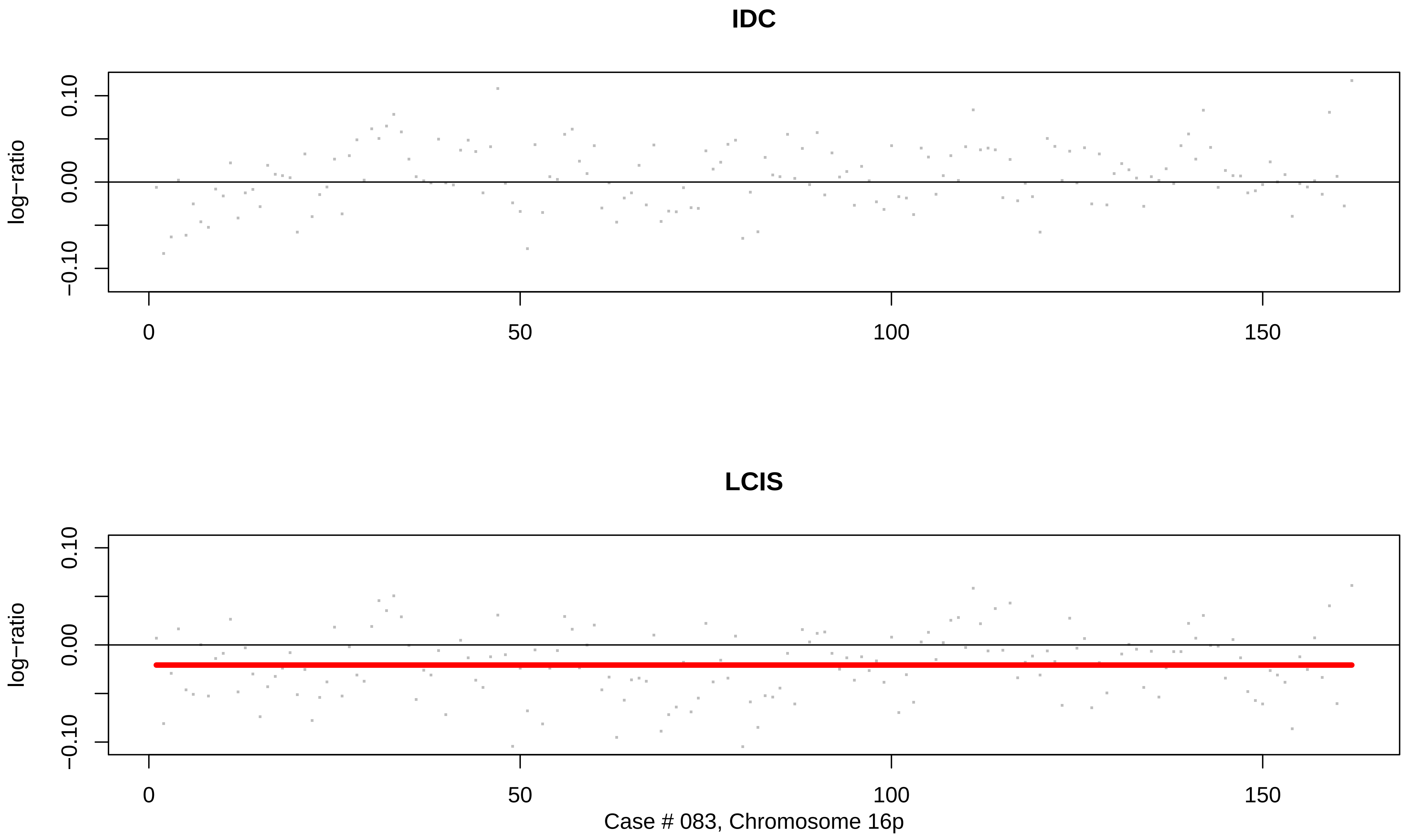

IDC
log−ratio
0.10
0.00
−0.10
0
50
100
150
LCIS
log−ratio
0.10
0.00
−0.10
0
50
100
150
Case # 083, Chromosome 16p

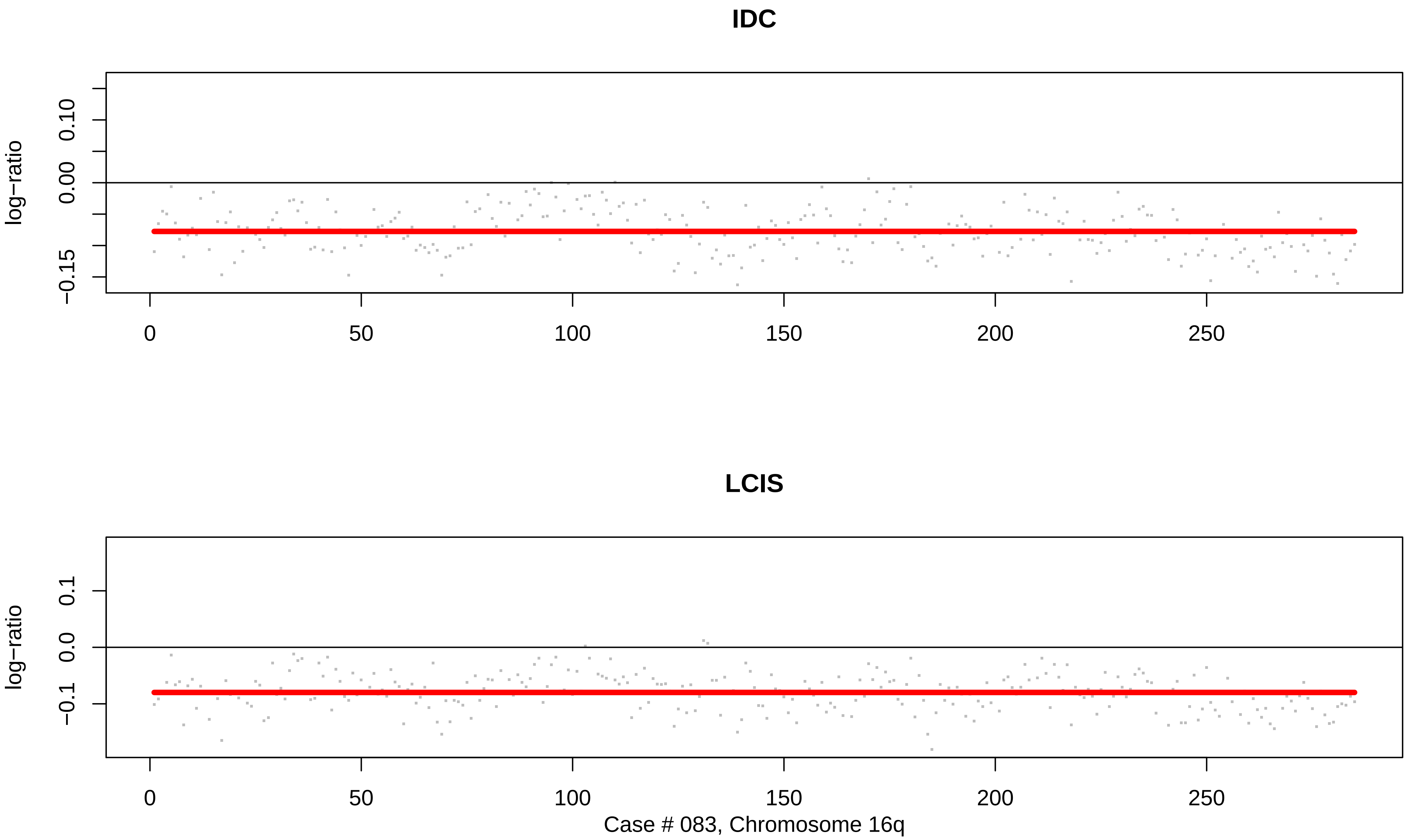

IDC
log−ratio
−0.15
0.00
0.10
0
50
100
150
200
250
LCIS
log−ratio
−0.1
0.0
0.1
0
50
100
150
200
250
Case # 083, Chromosome 16q

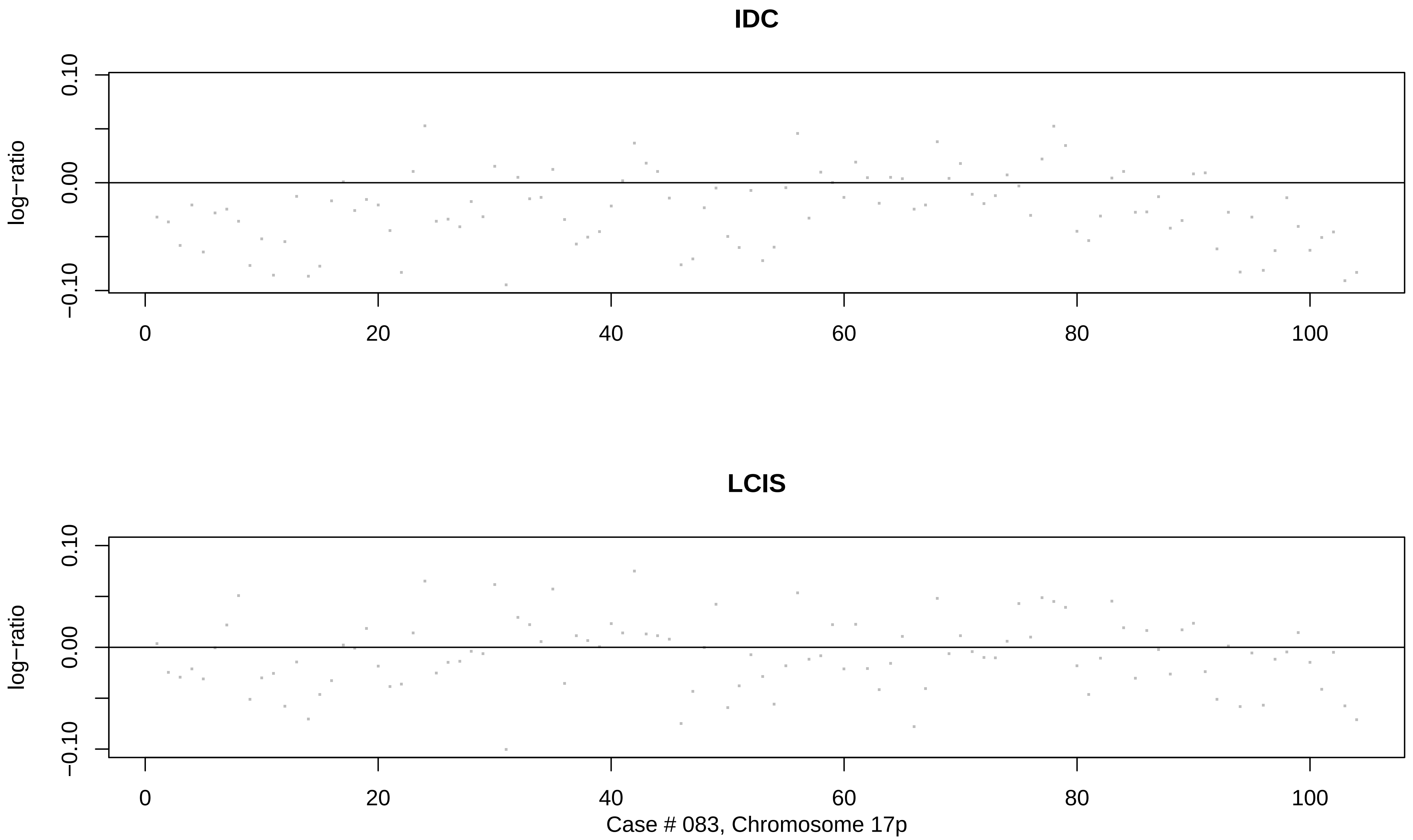

IDC
log−ratio
0.10
0.00
−0.10
0
20
40
60
80
100
LCIS
log−ratio
0.10
0.00
−0.10
0
20
40
60
80
100
Case # 083, Chromosome 17p

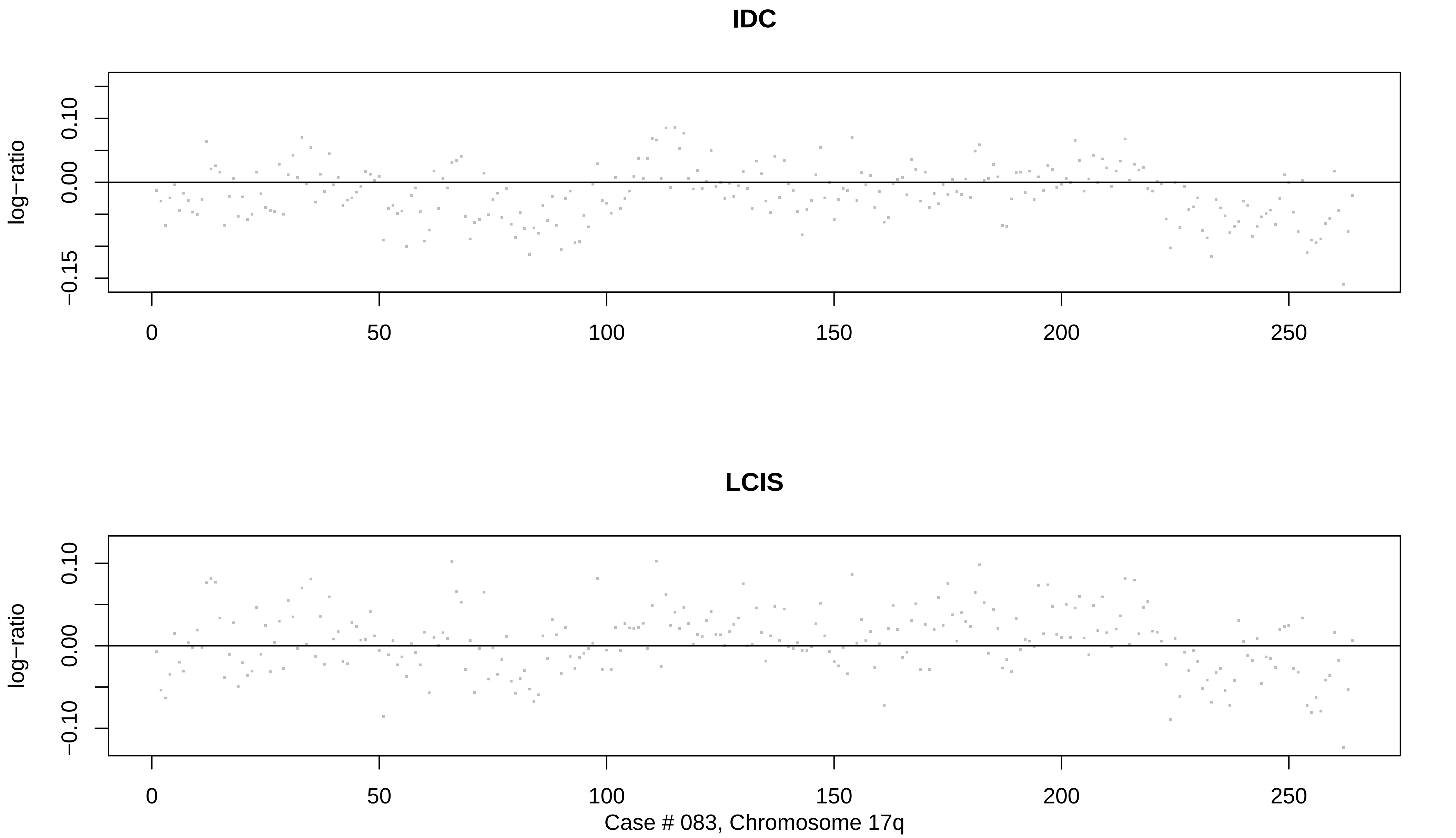

IDC
log−ratio
0.10
0.00
−0.15
0
50
100
150
200
250
LCIS
log−ratio
0.10
0.00
−0.10
0
50
100
150
200
250
Case # 083, Chromosome 17q

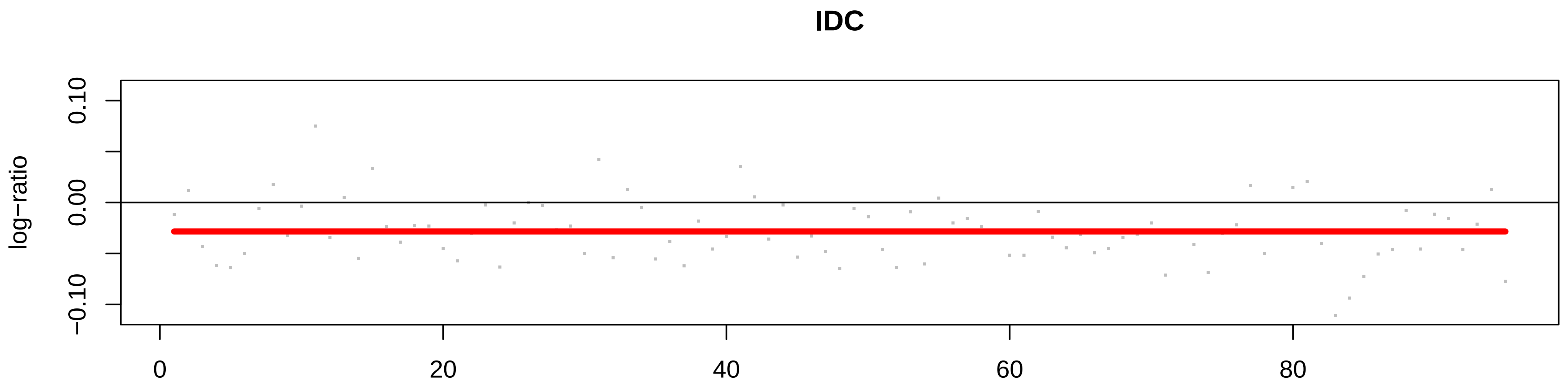

IDC
log−ratio
0.10
0.00
−0.10
0
20
40
60
80

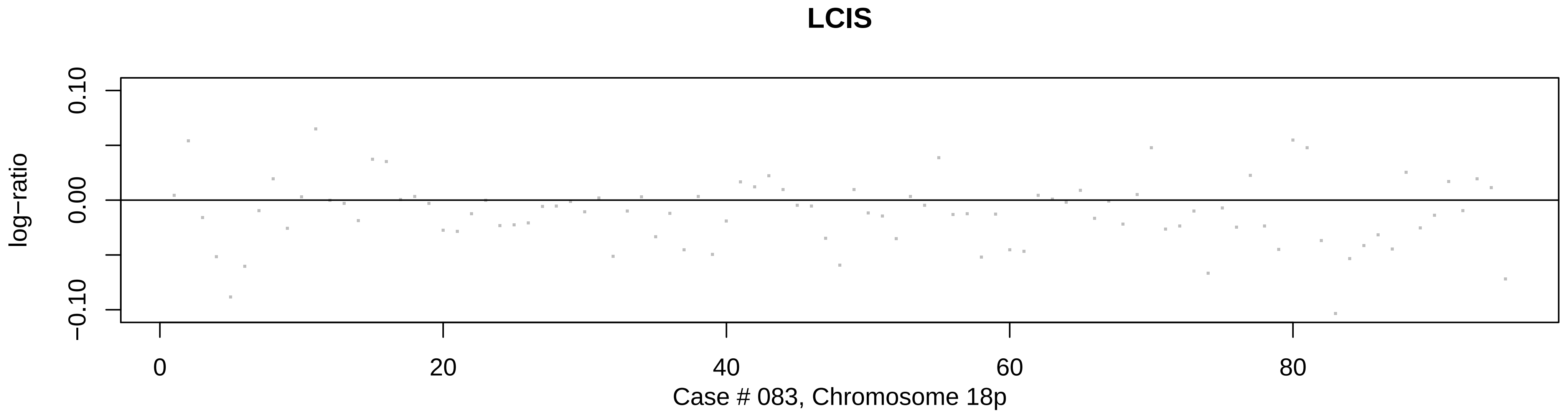

LCIS
log−ratio
0.10
0.00
−0.10
0
20
40
60
80
Case # 083, Chromosome 18p

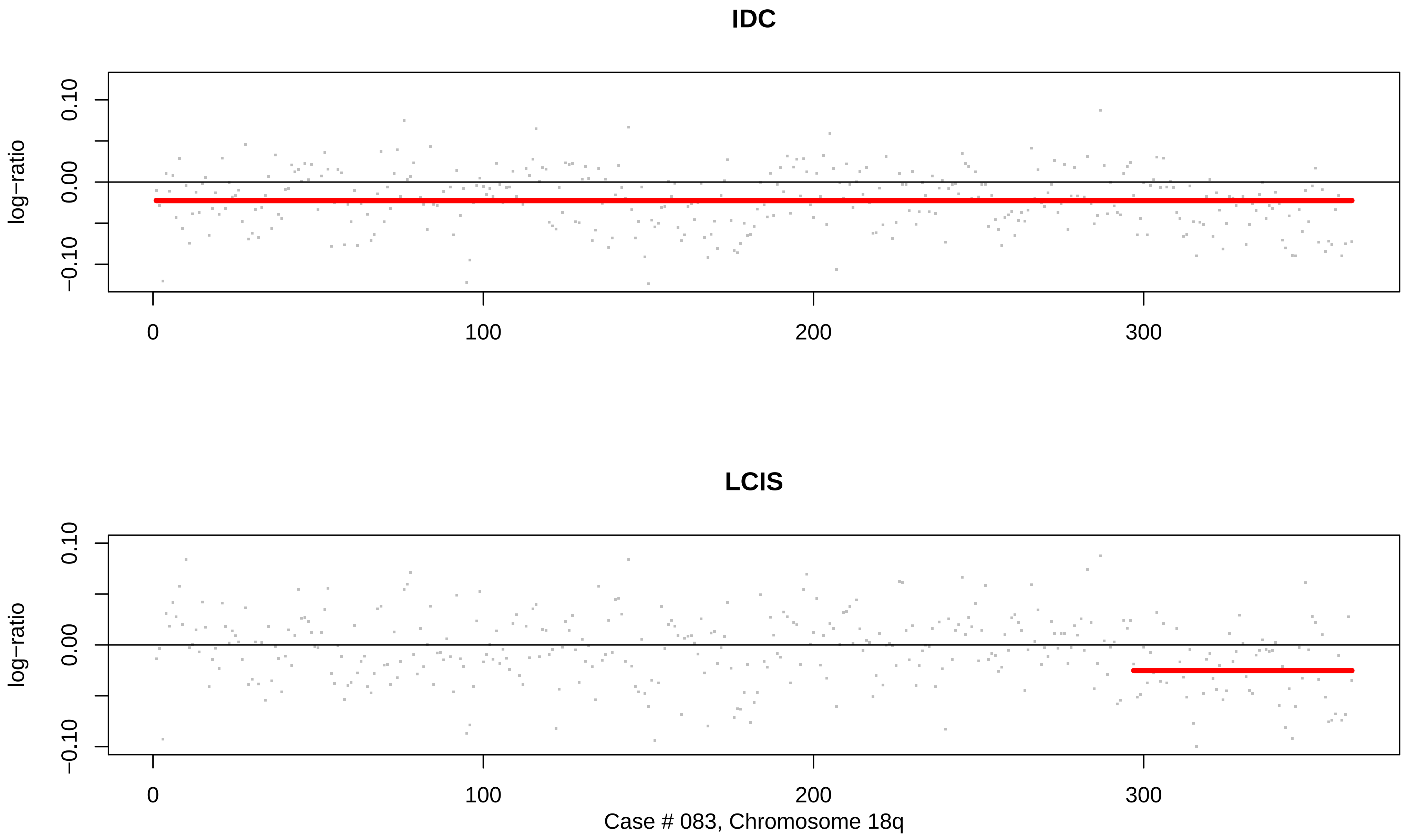
IDC
log−ratio
0.10
0.00
−0.10
0
100
200
300
LCIS
log−ratio
0.10
0.00
−0.10
0
100
200
300
Case # 083, Chromosome 18q

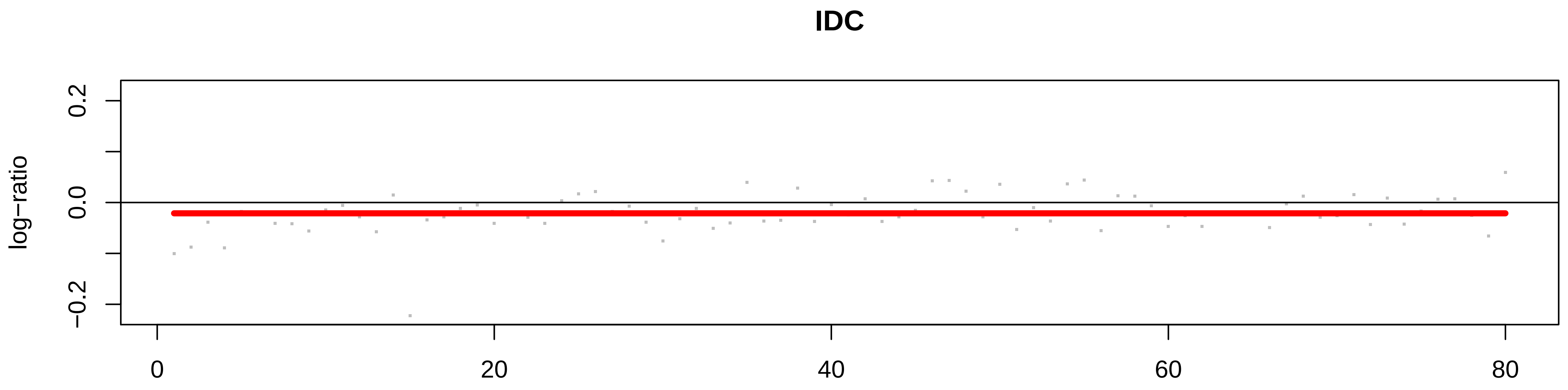

IDC
log−ratio
0.2
0.0
−0.2
0
20
40
60
80

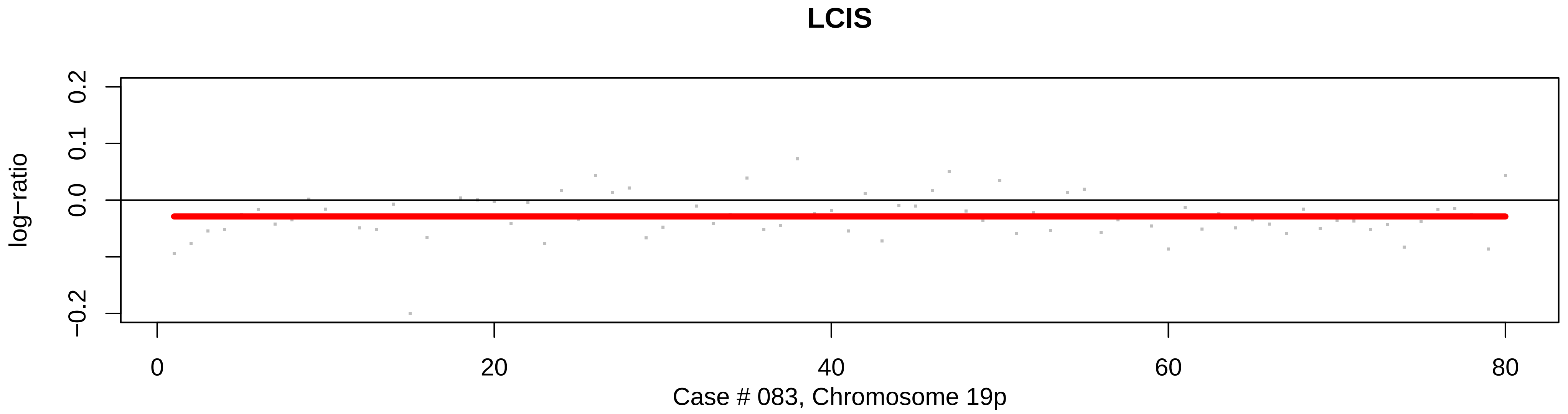

LCIS
log−ratio
0.2
0.1
0.0
−0.2
0
20
40
60
80
Case # 083, Chromosome 19p

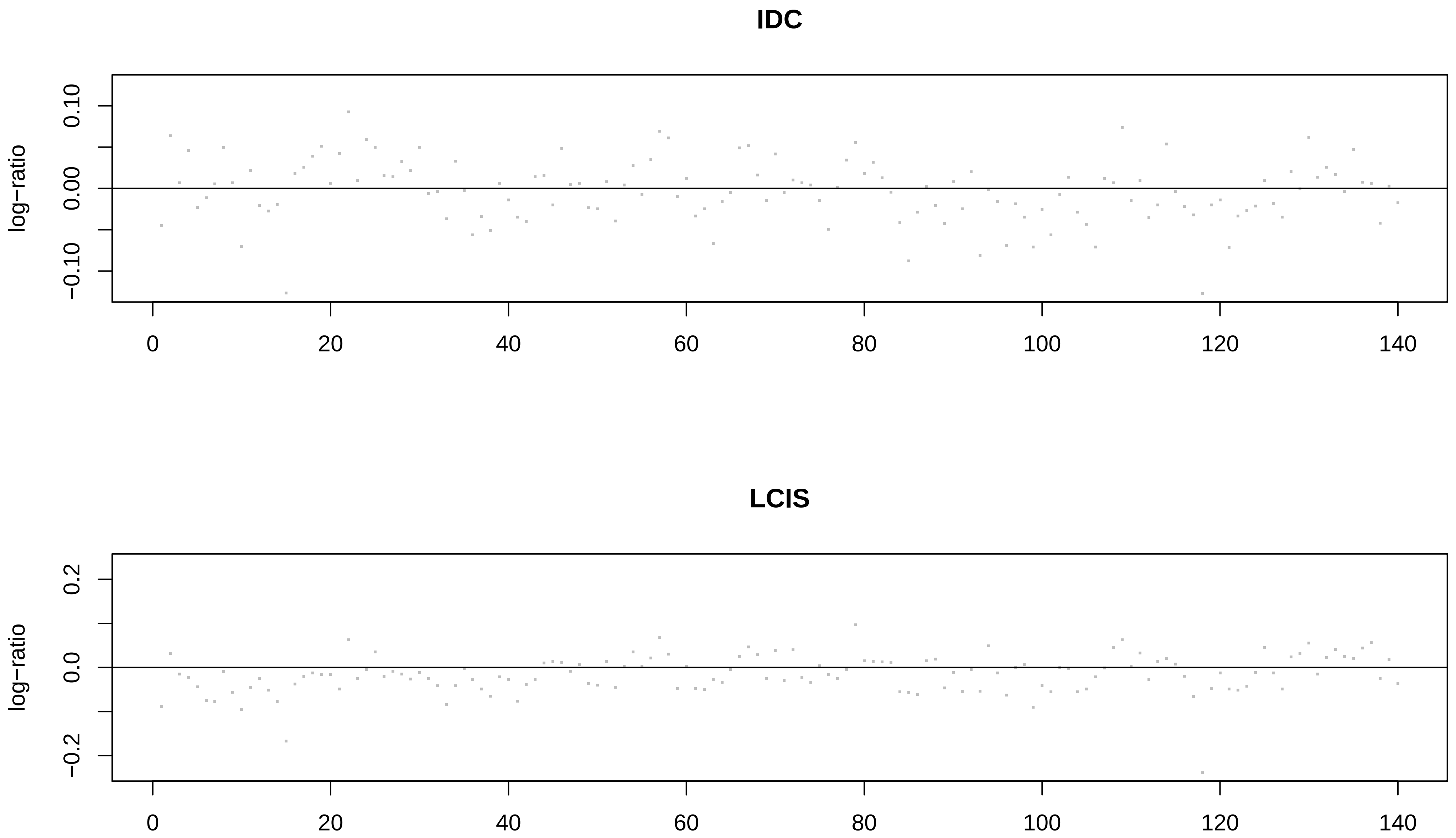

Case # 083, Chromosome 19q

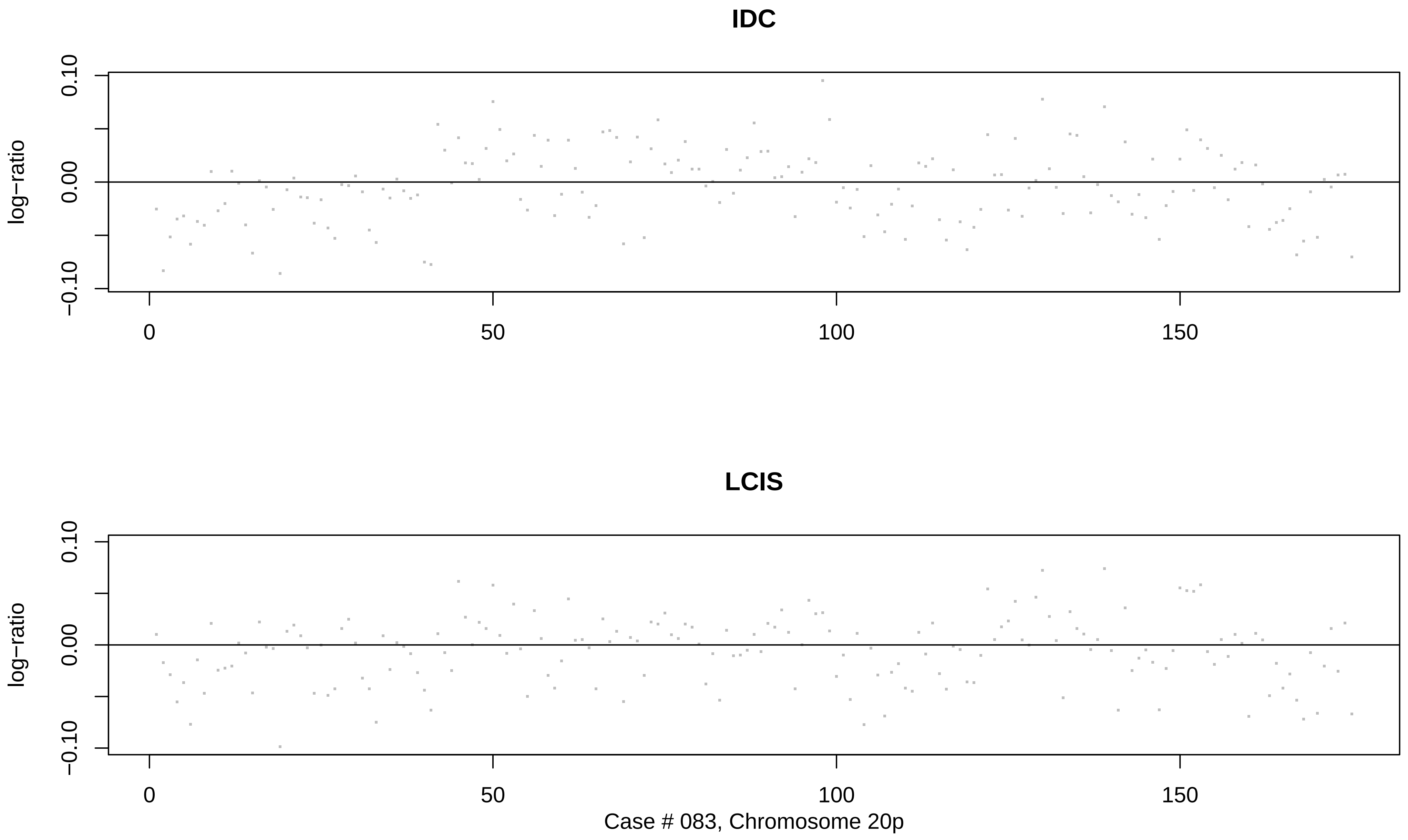
IDC
log−ratio
0.10
0.00
−0.10
0
50
100
150
LCIS
log−ratio
0.10
0.00
−0.10
0
50
100
150
Case # 083, Chromosome 20p

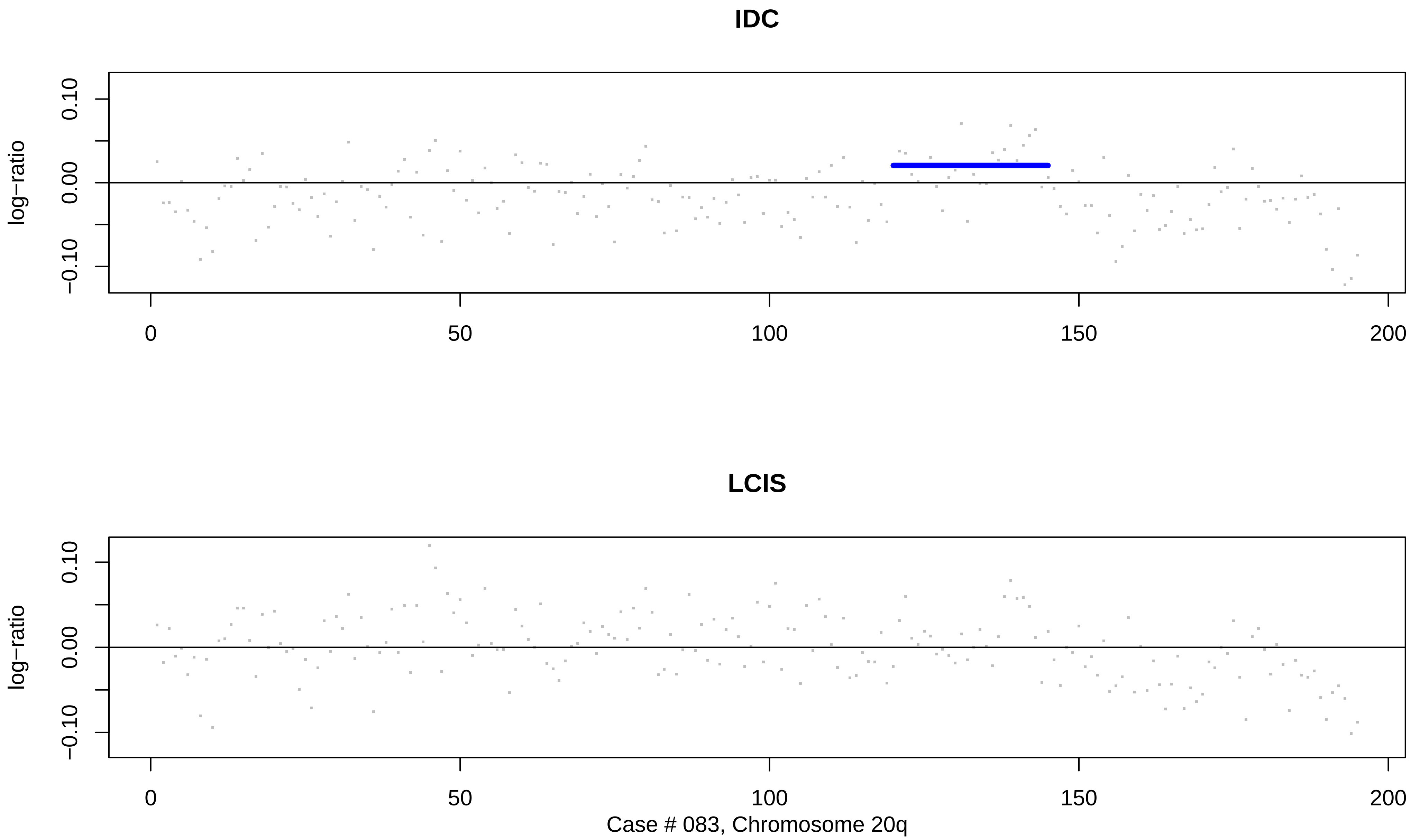
IDC
log−ratio
0.10
0.00
−0.10
0
50
100
150
200
LCIS
log−ratio
0.10
0.00
−0.10
0
50
100
150
200
Case # 083, Chromosome 20q

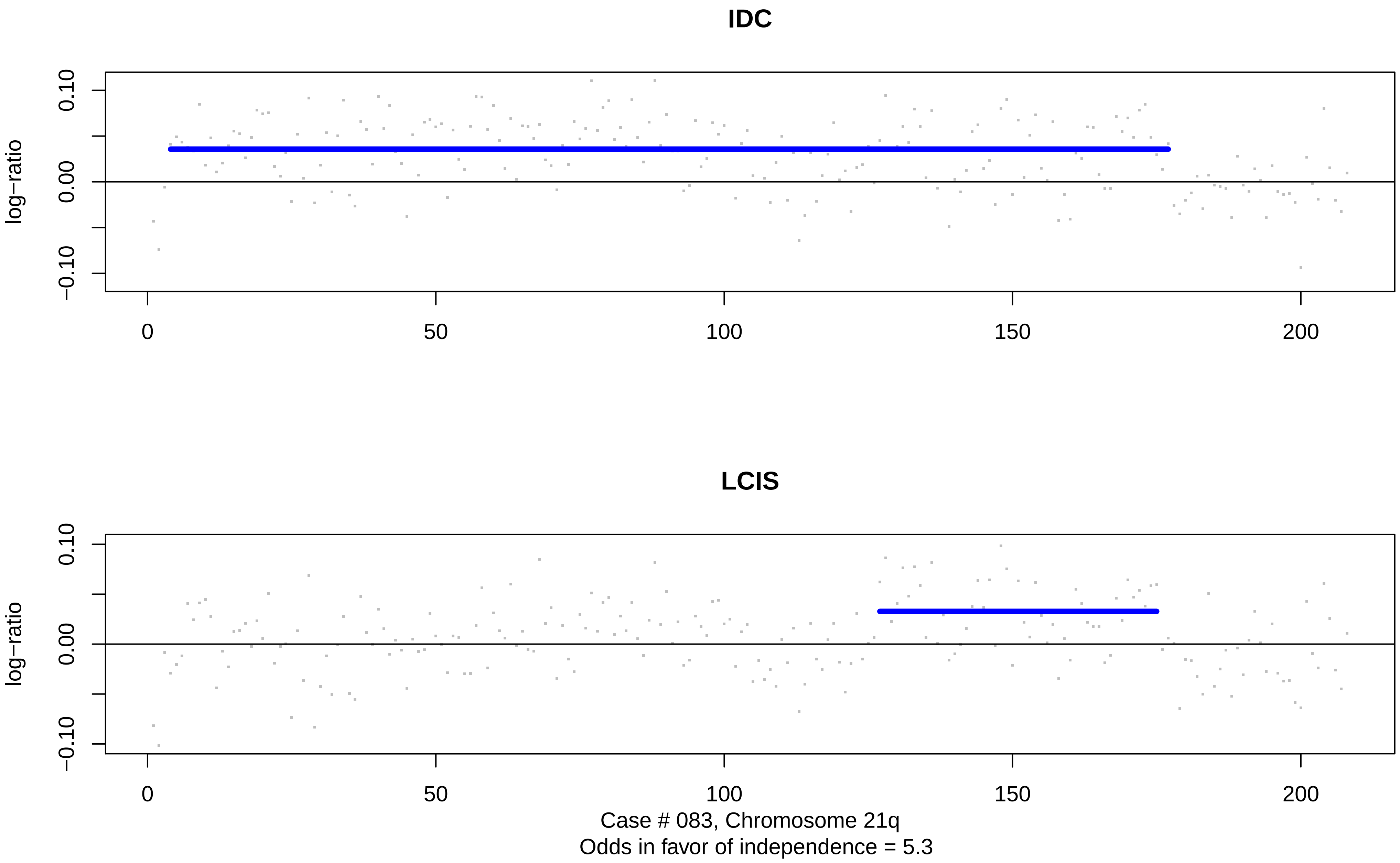
IDC
log−ratio
0.10
0.00
−0.10
0
50
100
150
200
LCIS
log−ratio
0.10
0.00
−0.10
0
50
100
150
200
Case # 083, Chromosome 21q
Odds in favor of independence = 5.3

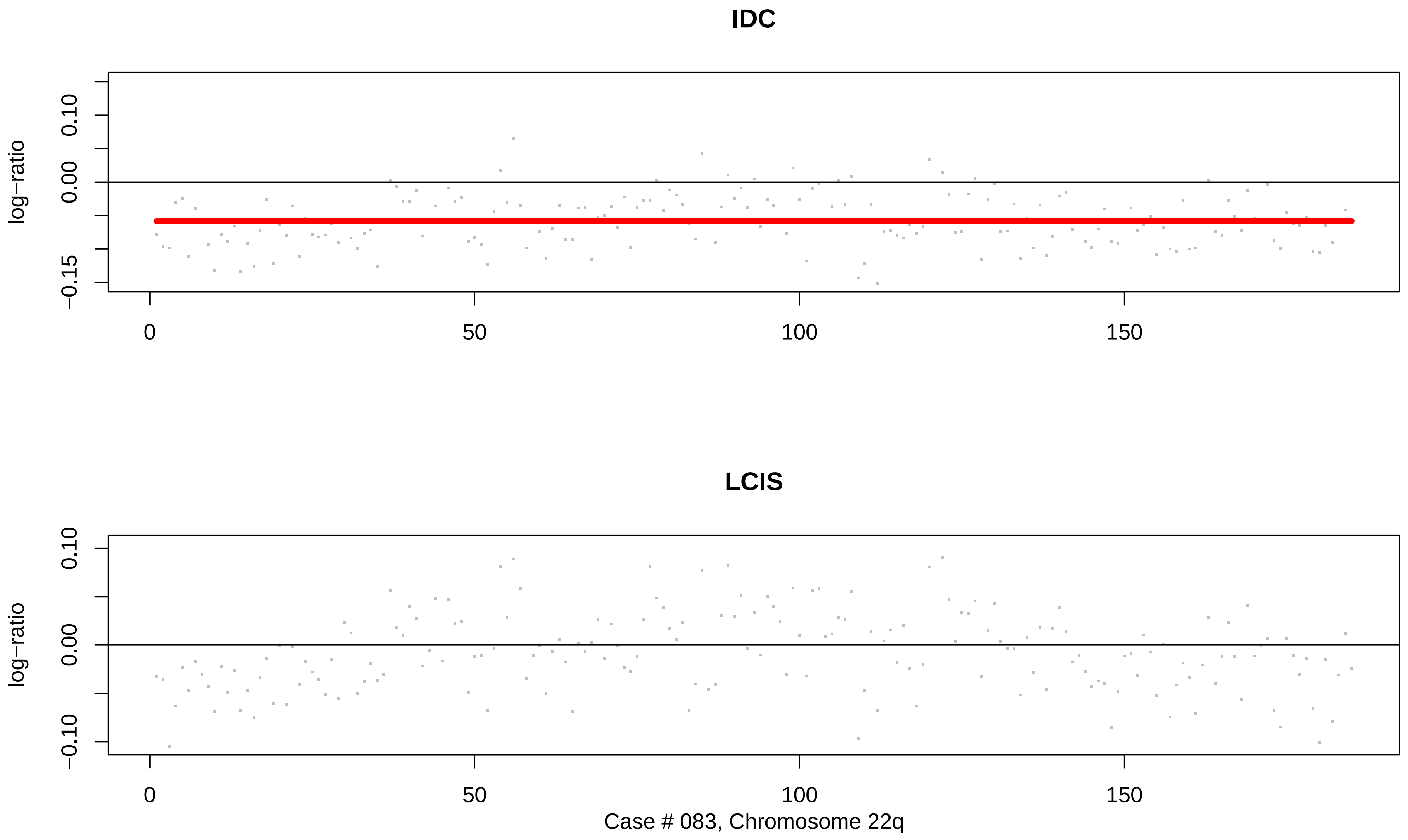
IDC
log−ratio
−0.15
0.00
0.10
0
50
100
150
LCIS
log−ratio
−0.10
0.00
0.10
0
50
100
150
Case # 083, Chromosome 22q
