# Supplementary material for: Clonal relatedness between lobular carcinoma in situ and synchronous malignant lesions
Source: Breast Cancer Res. 2012 Jul 9;14(4):R103. doi: 10.1186/bcr3222 (PMC3680923; doi:10.1186/bcr3222)
Supplement: Additional file 4 — Magnified version of genome-wide plots with detailed marker plots and segmentation on a chromosome-arm-specific basis. [file bcr3222-S4.ZIP › Case 076.pdf]

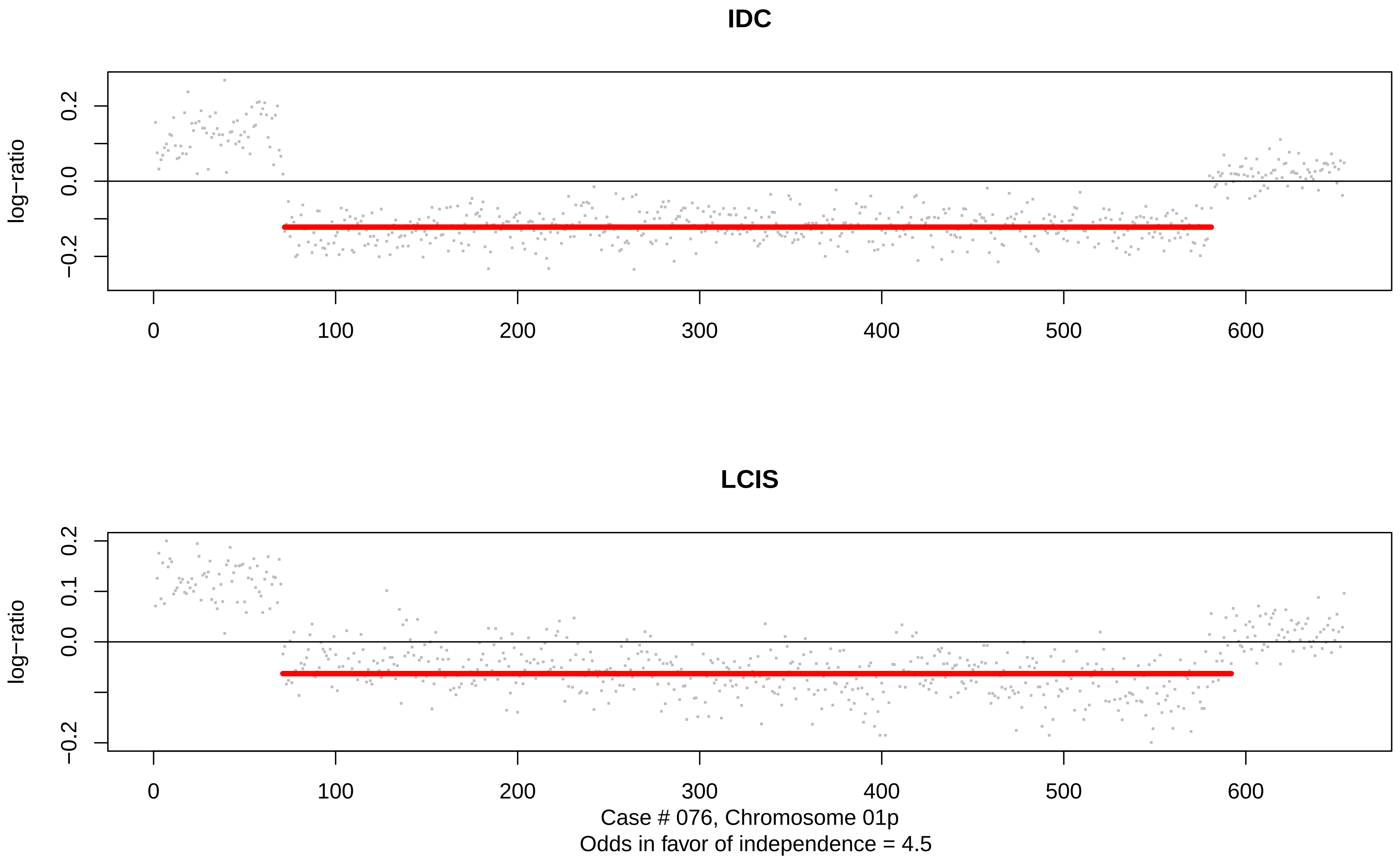
IDC
LCIS
log−ratio
log−ratio
0.2
0.1
0.0
−0.2
0
100
200
300
400
500
600
Case # 076, Chromosome 01p
Odds in favor of independence = 4.5

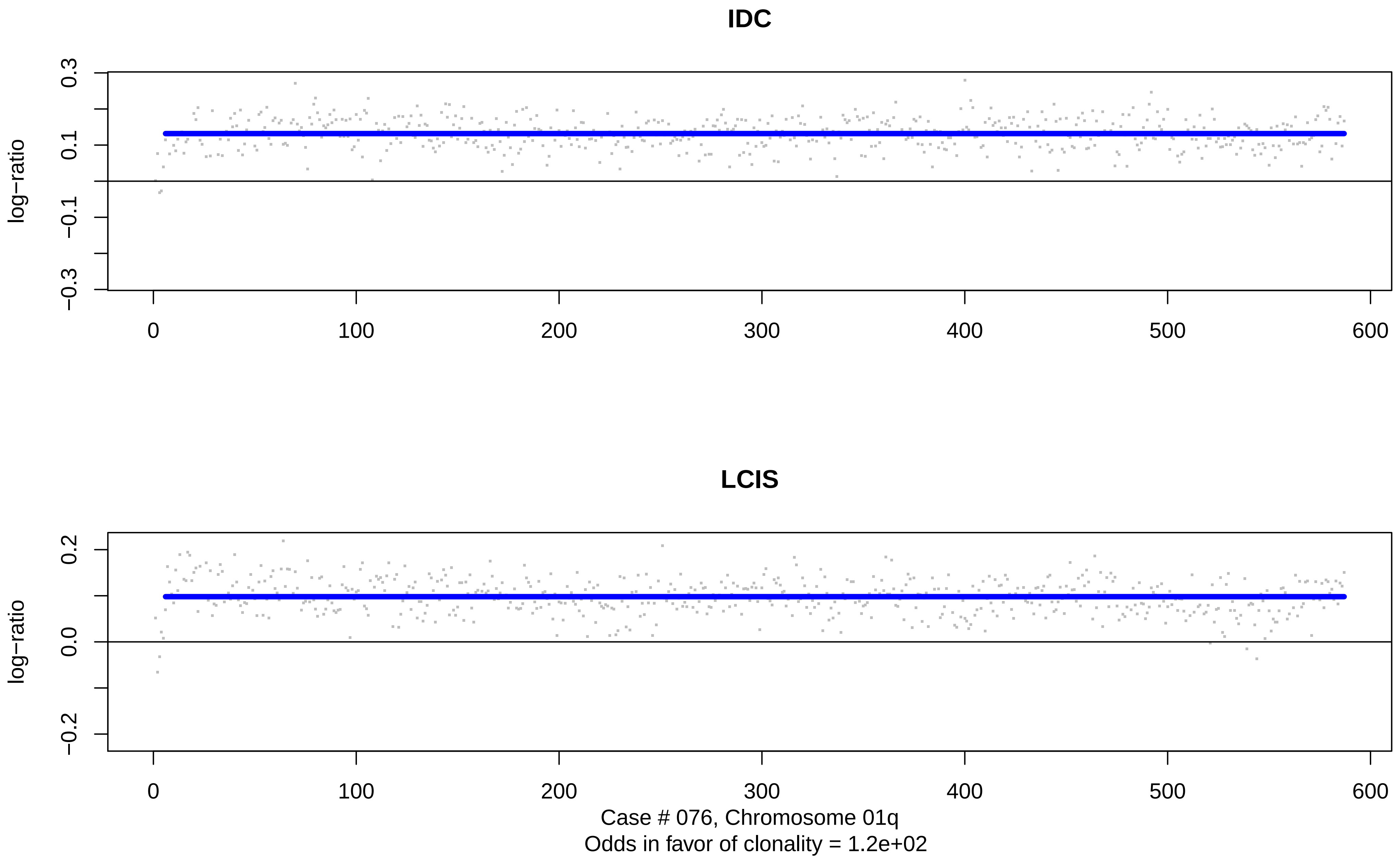

IDC
log−ratio
0.3
0.1
−0.1
−0.3
0
100
200
300
400
500
600
LCIS
log−ratio
0.2
0.0
−0.2
0
100
200
300
400
500
600
Case # 076, Chromosome 01q
Odds in favor of clonality = 1.2e+02

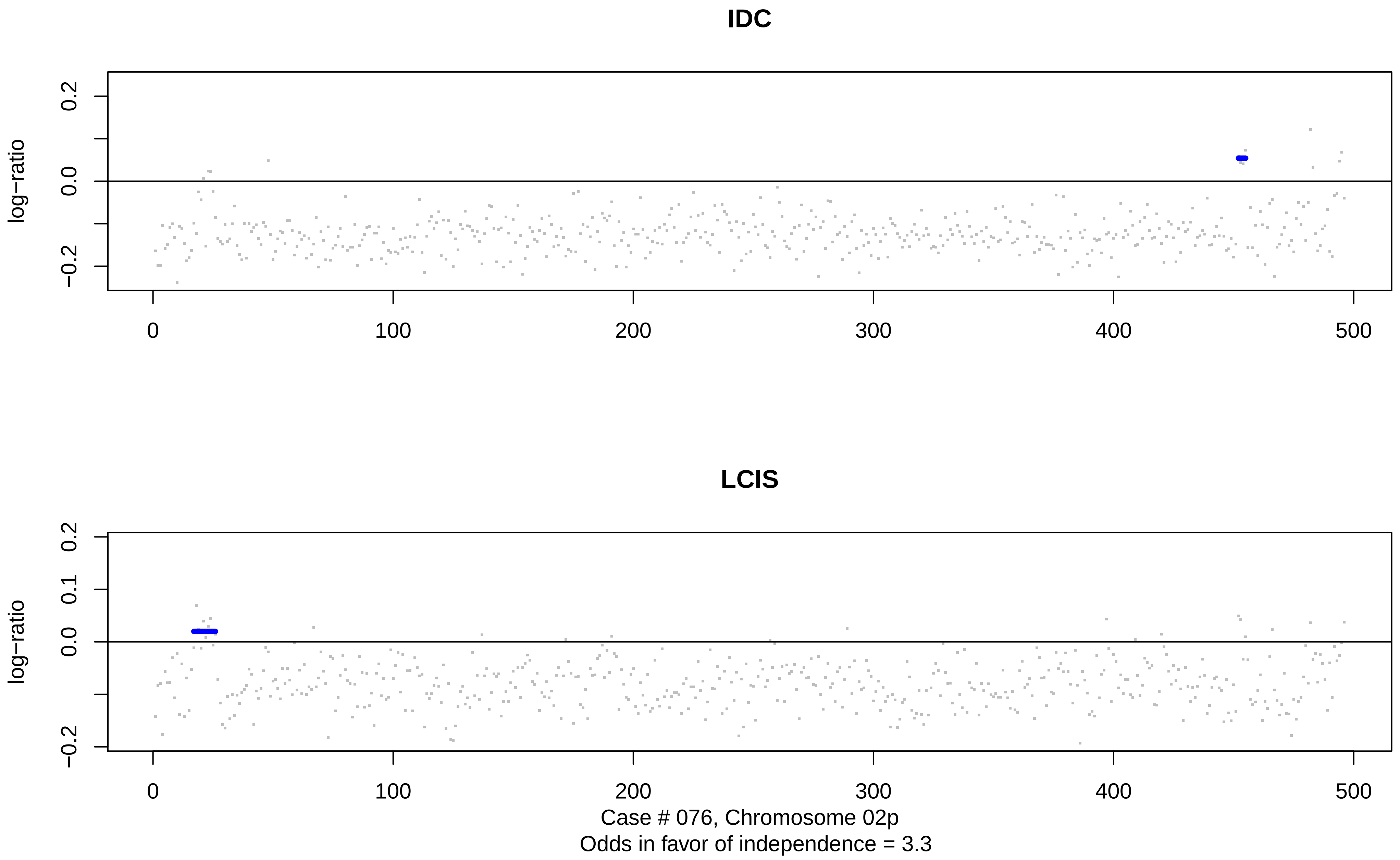
IDC
log−ratio
0.2
0.0
−0.2
0
100
200
300
400
500
LCIS
log−ratio
0.2
0.1
0.0
−0.2
0
100
200
300
400
500
Case # 076, Chromosome 02p
Odds in favor of independence = 3.3

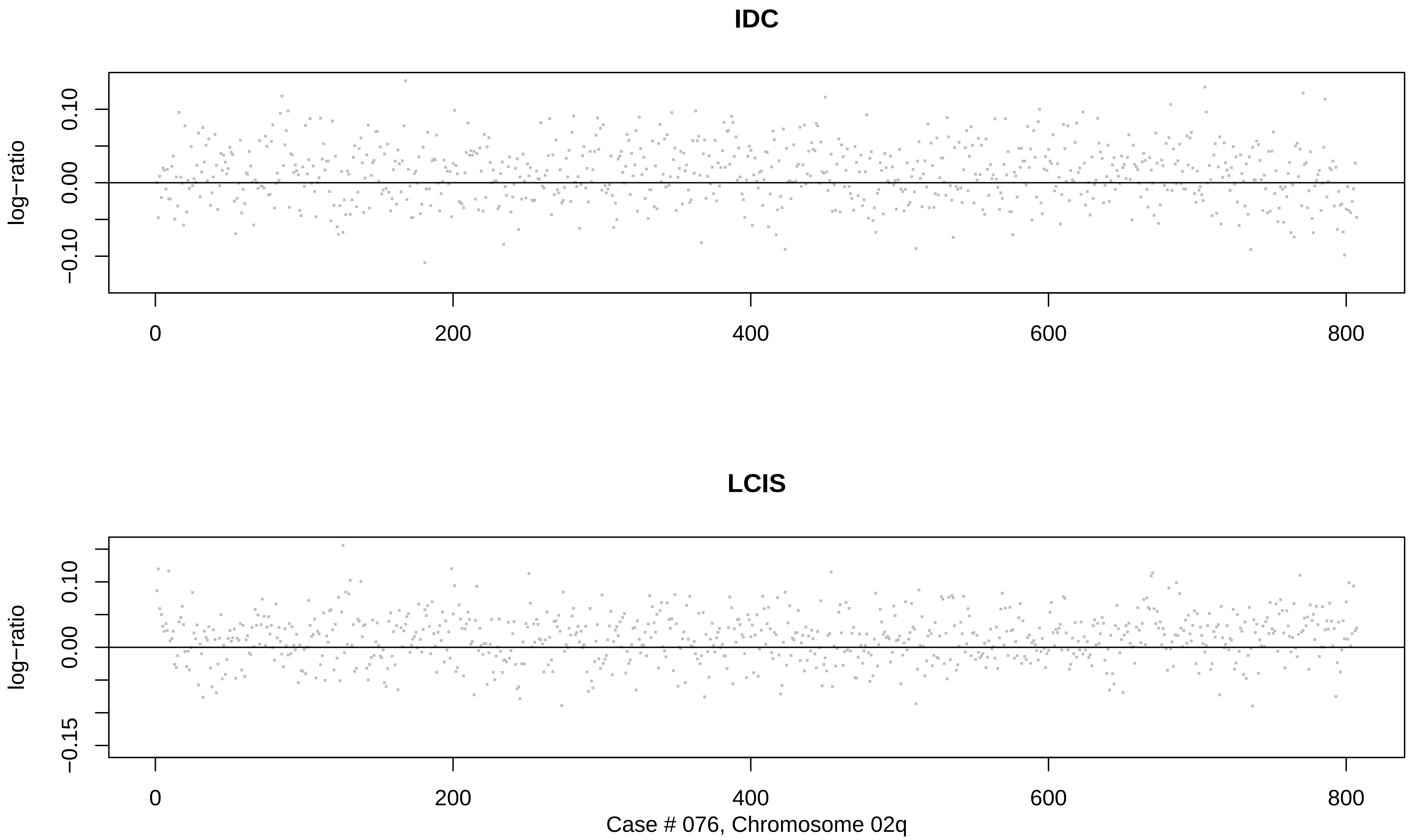
IDC
log−ratio
0.10
0.00
−0.10
0
200
400
600
800
LCIS
log−ratio
0.10
0.00
−0.15
0
200
400
600
800
Case # 076, Chromosome 02q

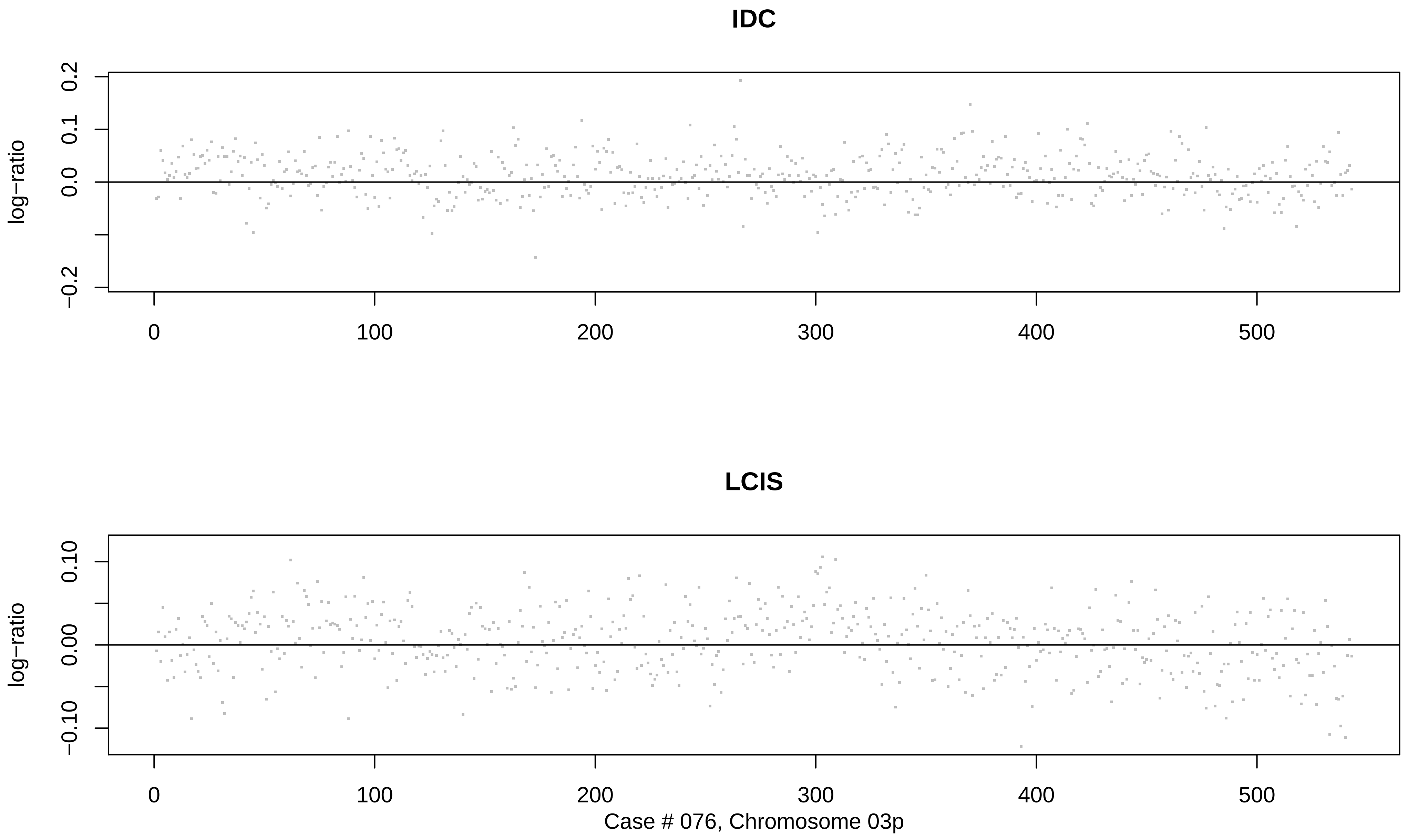

IDC
log−ratio
0.2
0.1
0.0
−0.2
0
100
200
300
400
500
LCIS
log−ratio
0.10
0.00
−0.10
0
100
200
300
400
500
Case # 076, Chromosome 03p

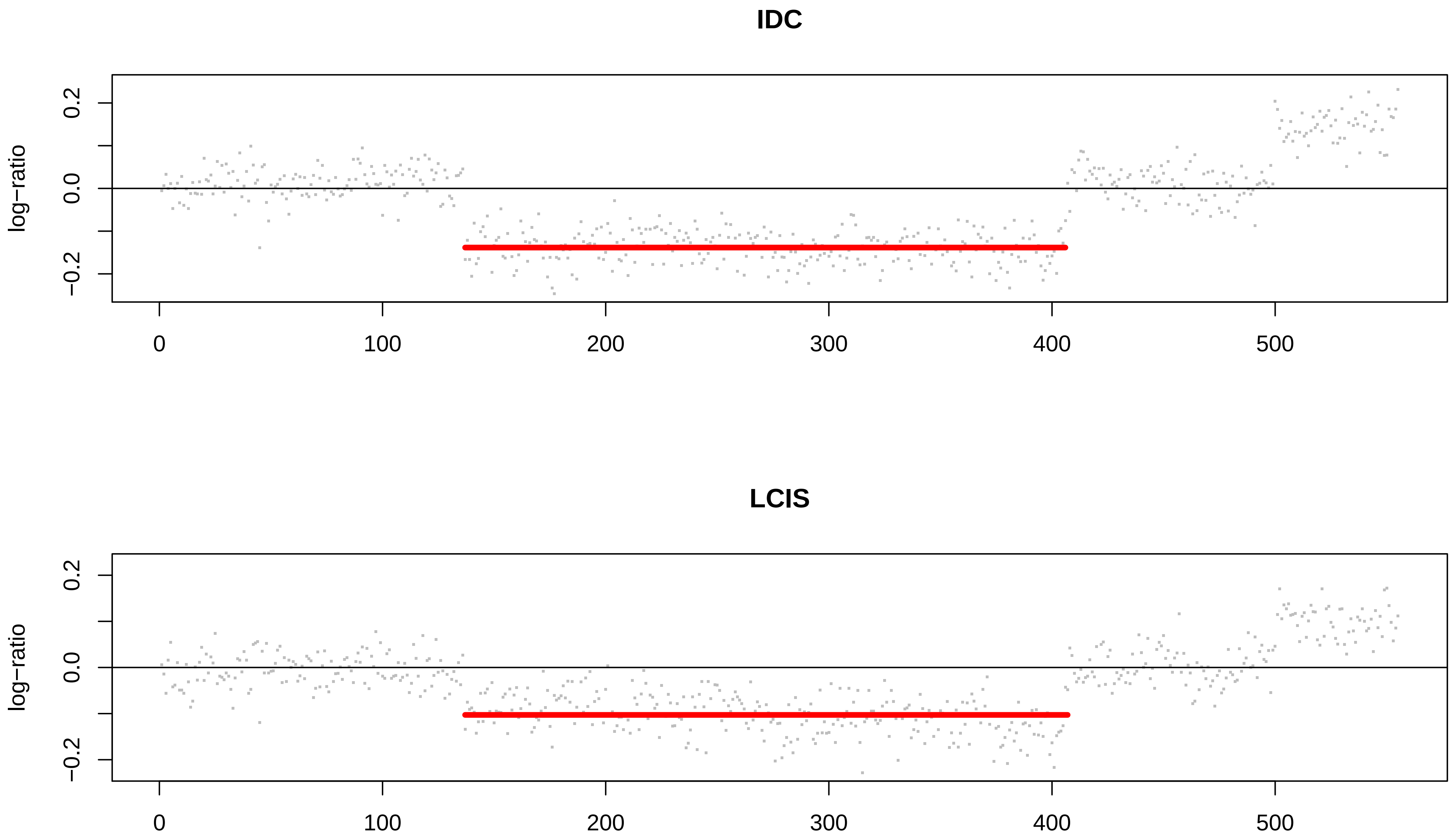

Case # 076, Chromosome 03q
Odds in favor of clonality = 4.6e+02

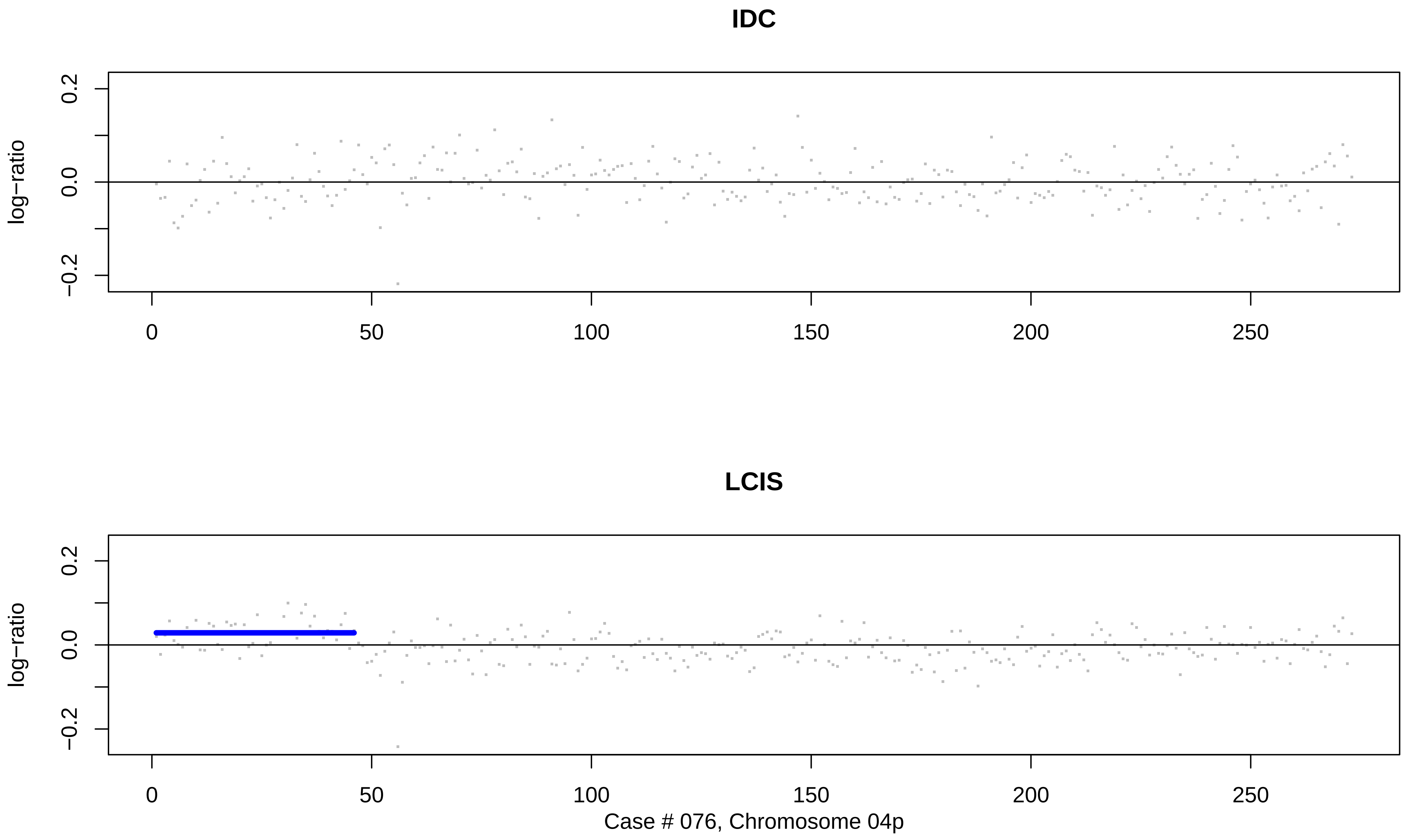
IDC
LCIS
log−ratio
0.2
0.0
−0.2
0
50
100
150
200
250
Case # 076, Chromosome 04p

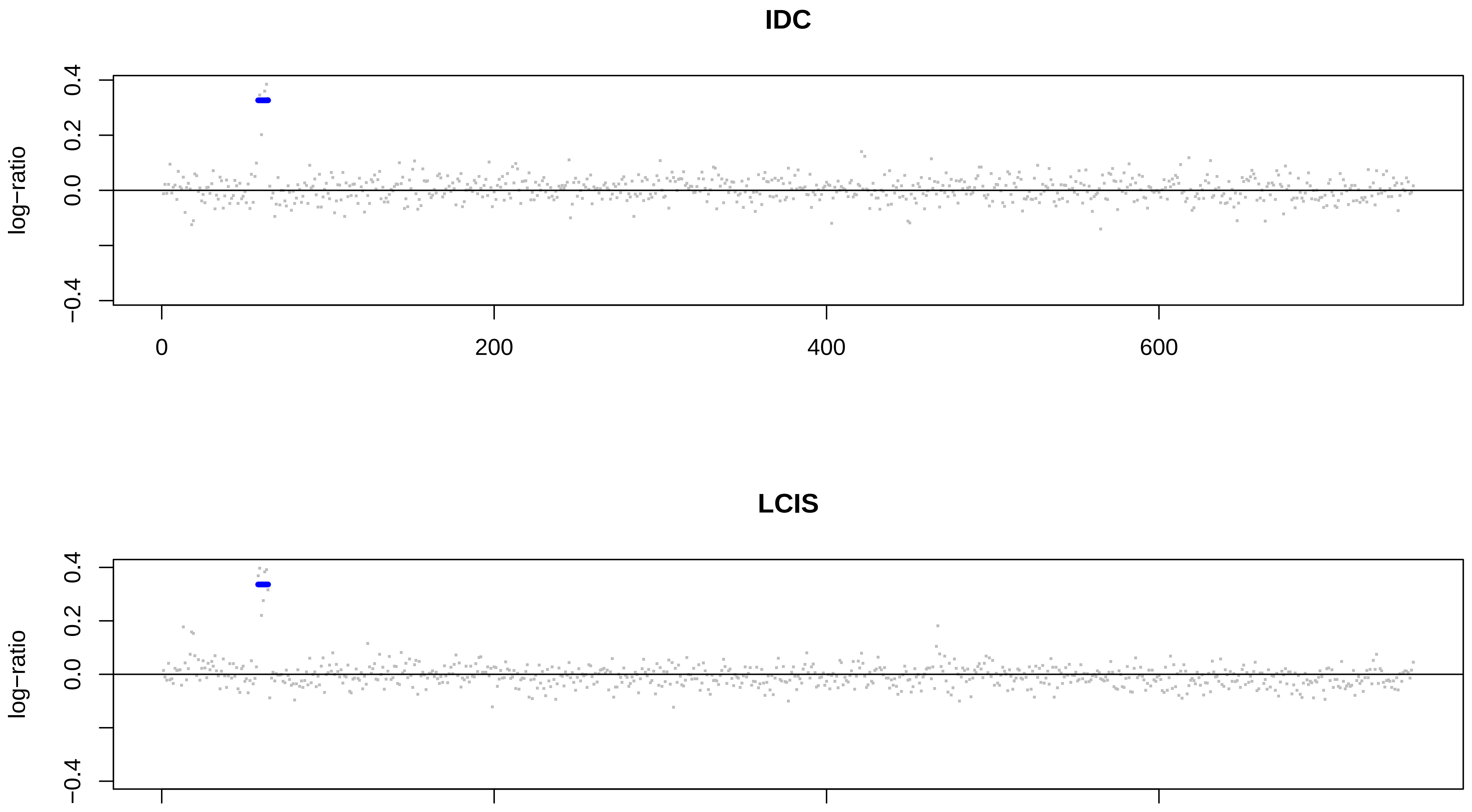

Case # 076, Chromosome 04q
Odds in favor of clonality = 8.3e+02

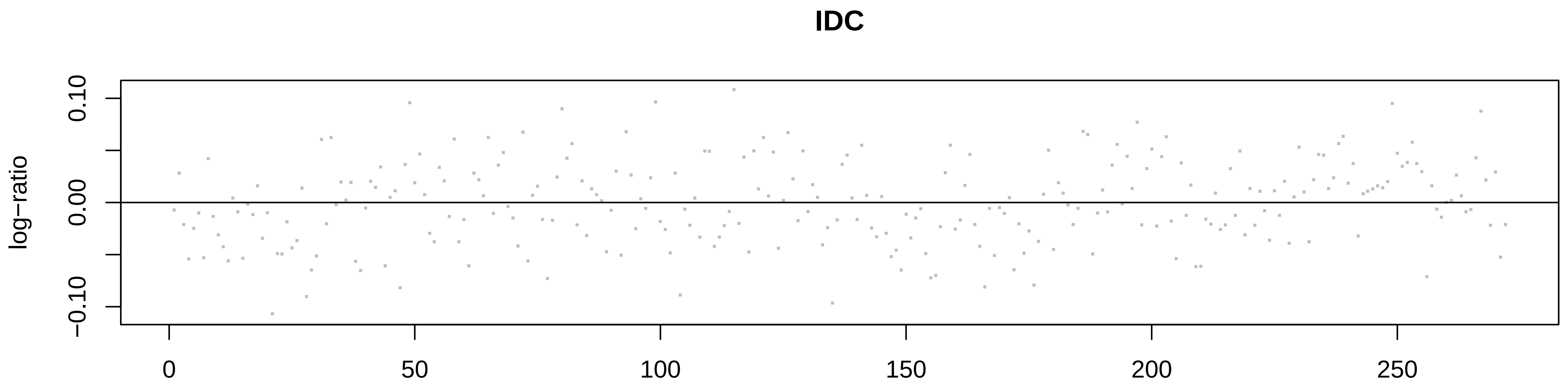
IDC
log−ratio
0.10
0.00
−0.10
0
50
100
150
200
250

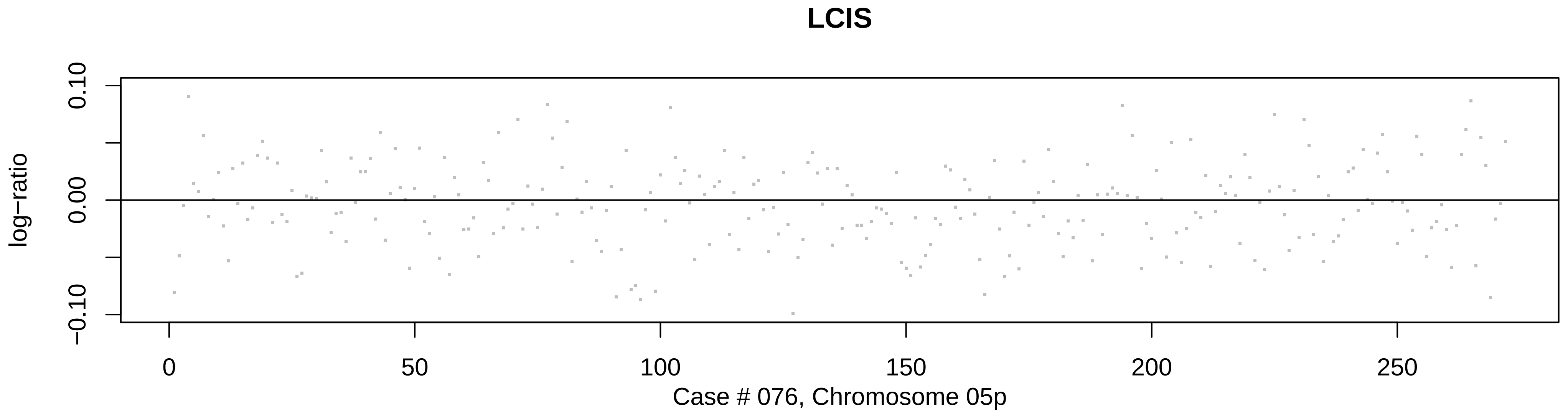
LCIS
log−ratio
0.10
0.00
−0.10
0
50
100
150
200
250
Case # 076, Chromosome 05p

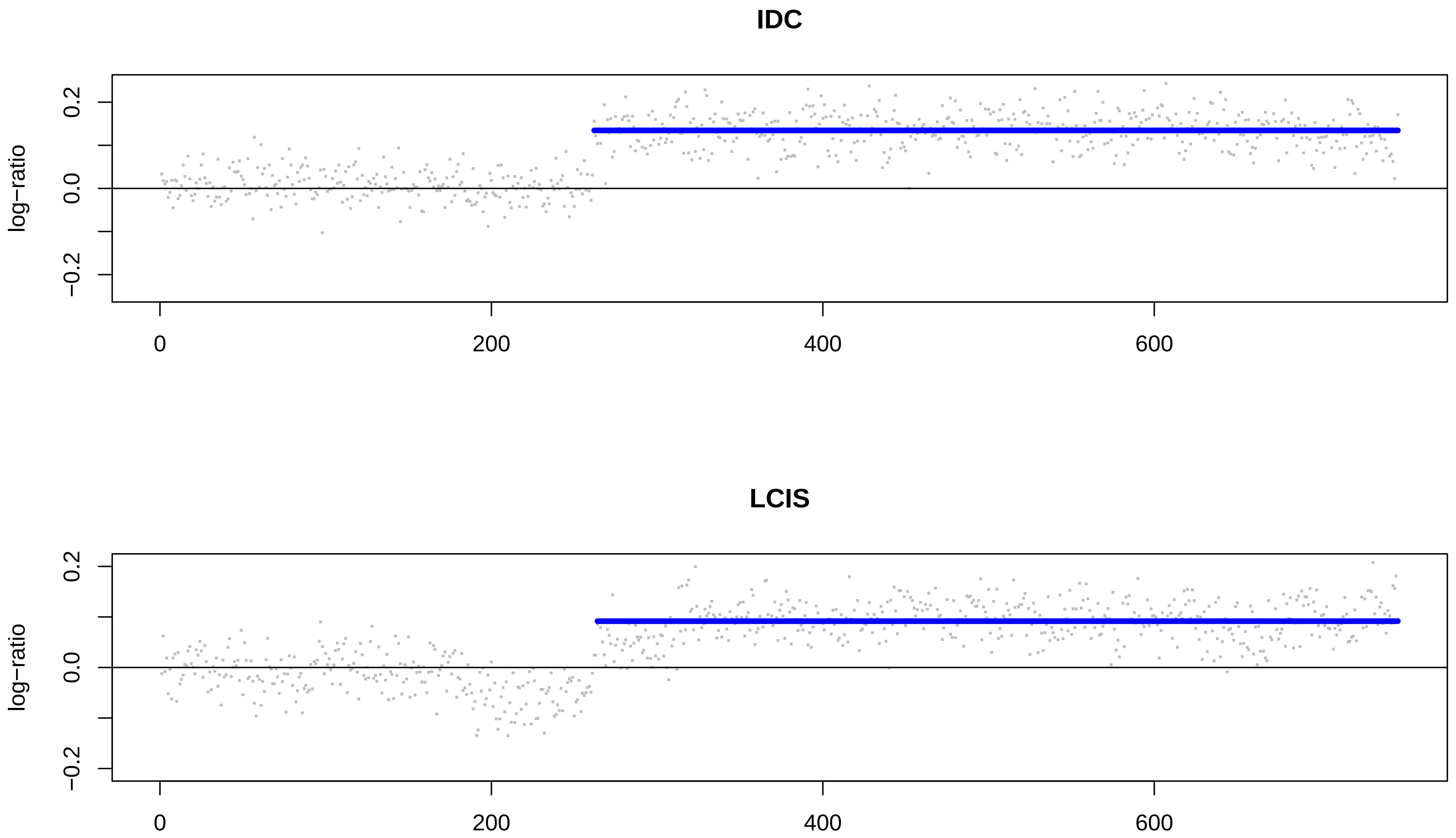

Case # 076, Chromosome 05q
Odds in favor of clonality = 1.7e+02

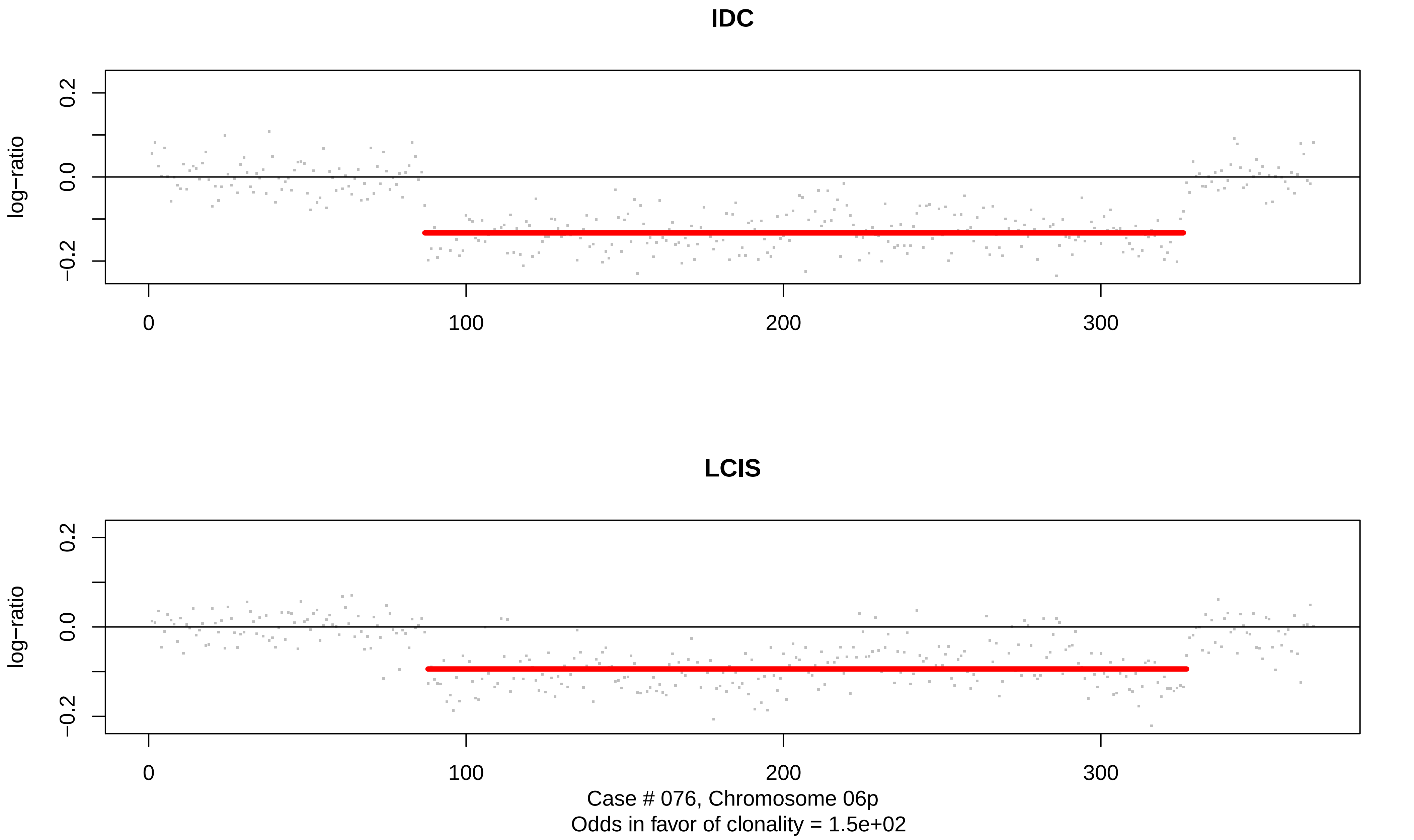
IDC
log−ratio
0.2
0.0
−0.2
0
100
200
300
LCIS
log−ratio
0.2
0.0
−0.2
0
100
200
300
Case # 076, Chromosome 06p
Odds in favor of clonality = 1.5e+02

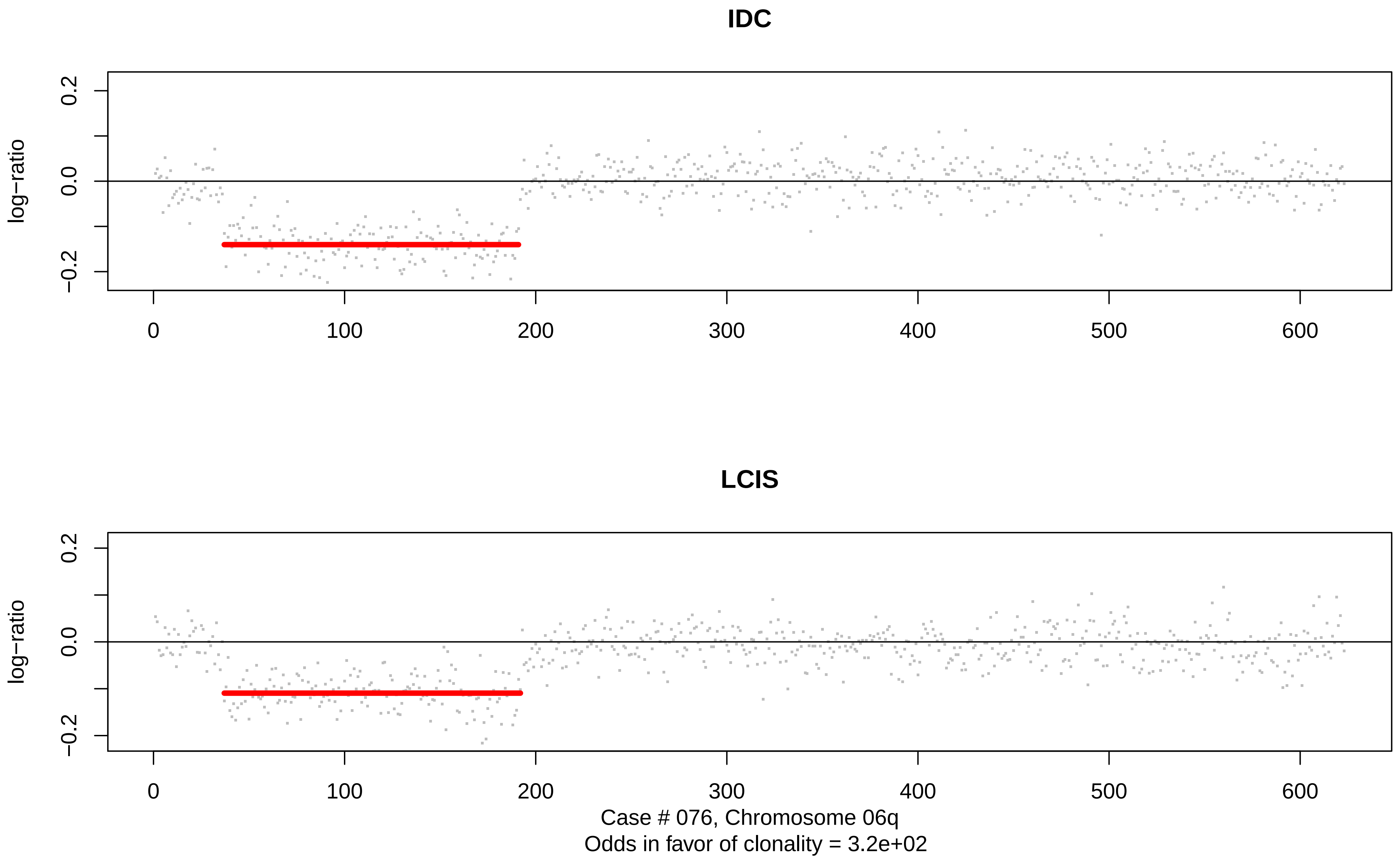
IDC
log−ratio
0.2
0.0
−0.2
0
100
200
300
400
500
600
LCIS
log−ratio
0.2
0.0
−0.2
0
100
200
300
400
500
600
Case # 076, Chromosome 06q
Odds in favor of clonality = 3.2e+02

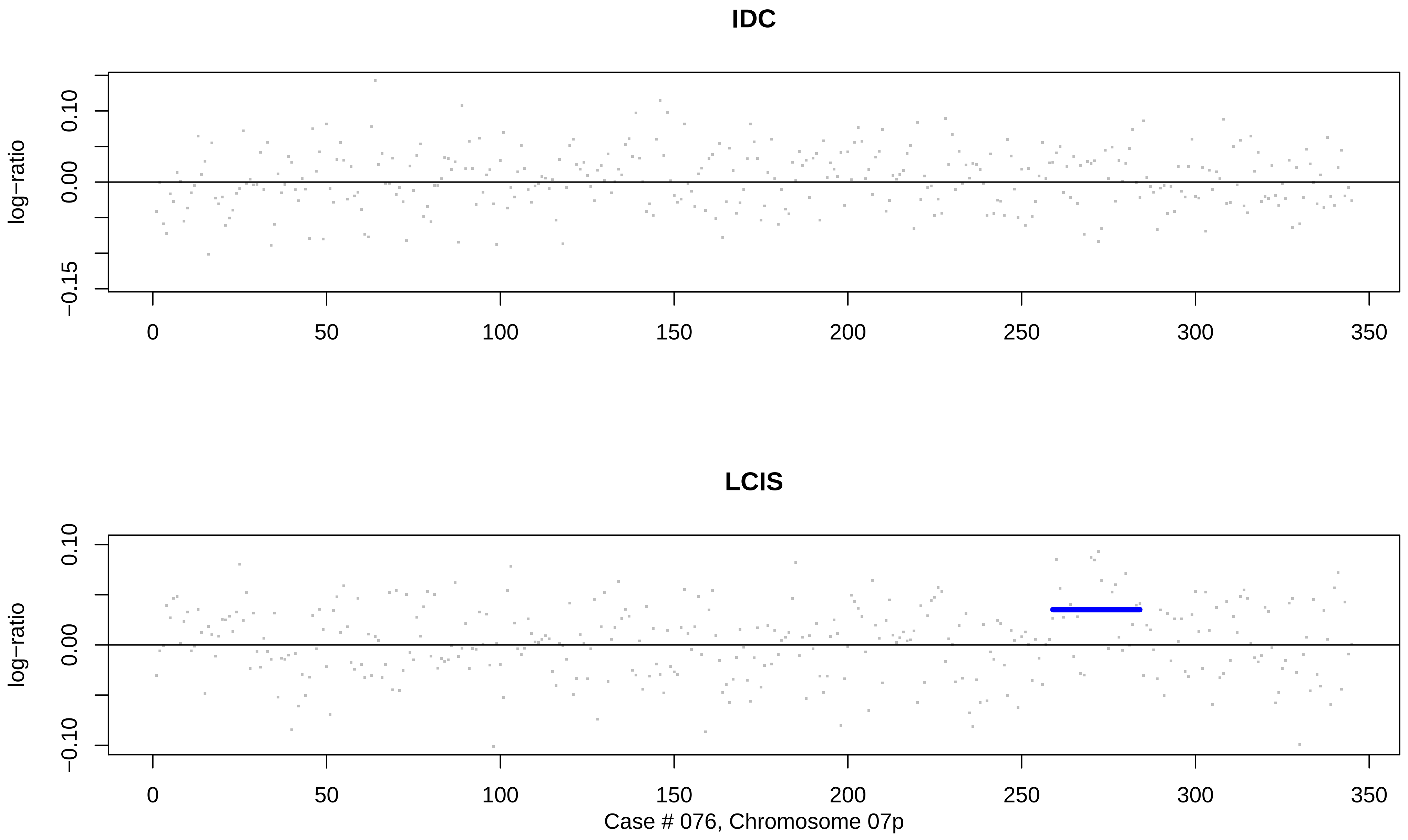
IDC
log−ratio
−0.15
0.00
0.10
0
50
100
150
200
250
300
350
LCIS
log−ratio
−0.10
0.00
0.10
0
50
100
150
200
250
300
350
Case # 076, Chromosome 07p

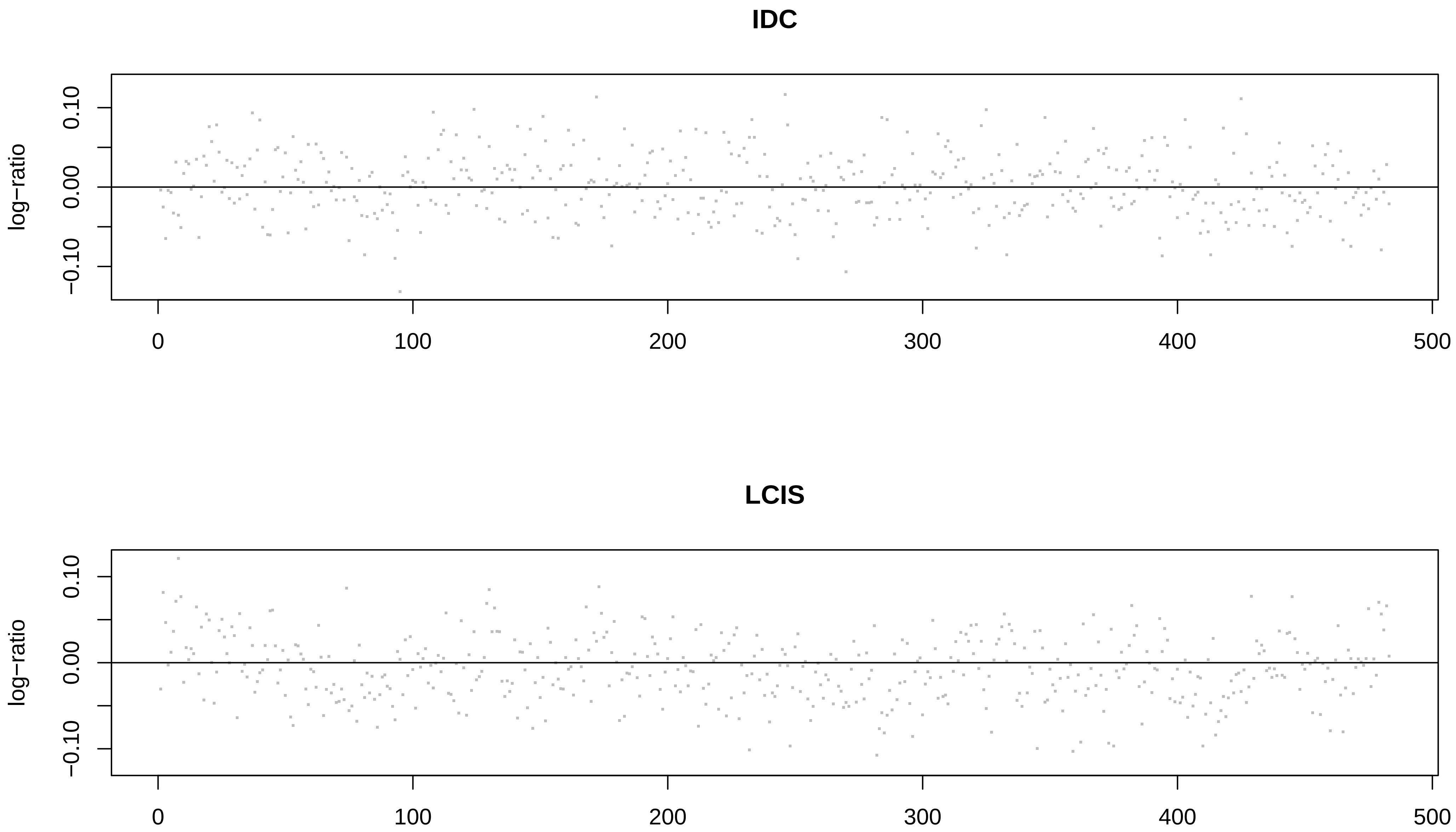

Case # 076, Chromosome 07q

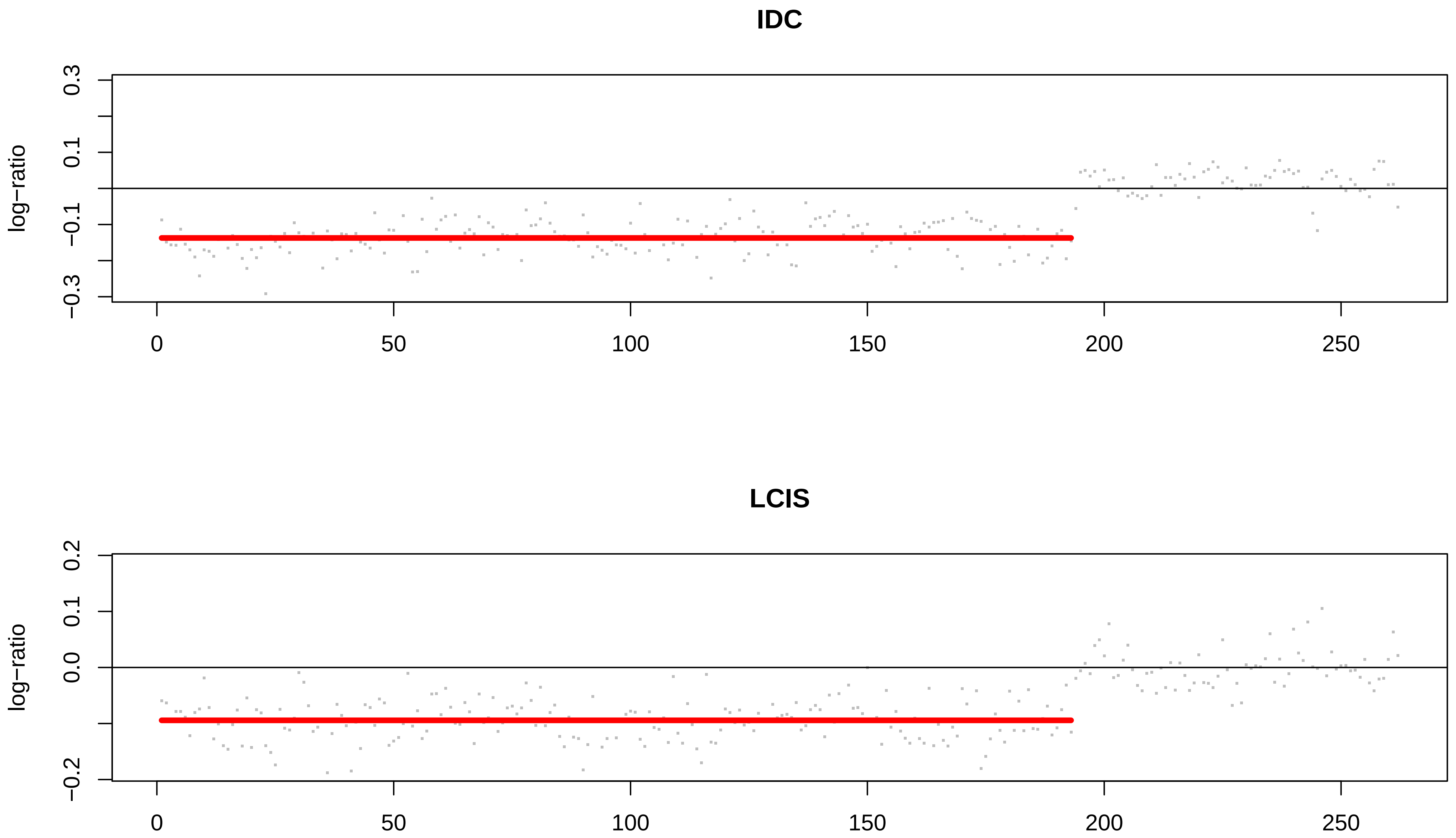

Case # 076, Chromosome 08p
Odds in favor of clonality = 1.1e+02

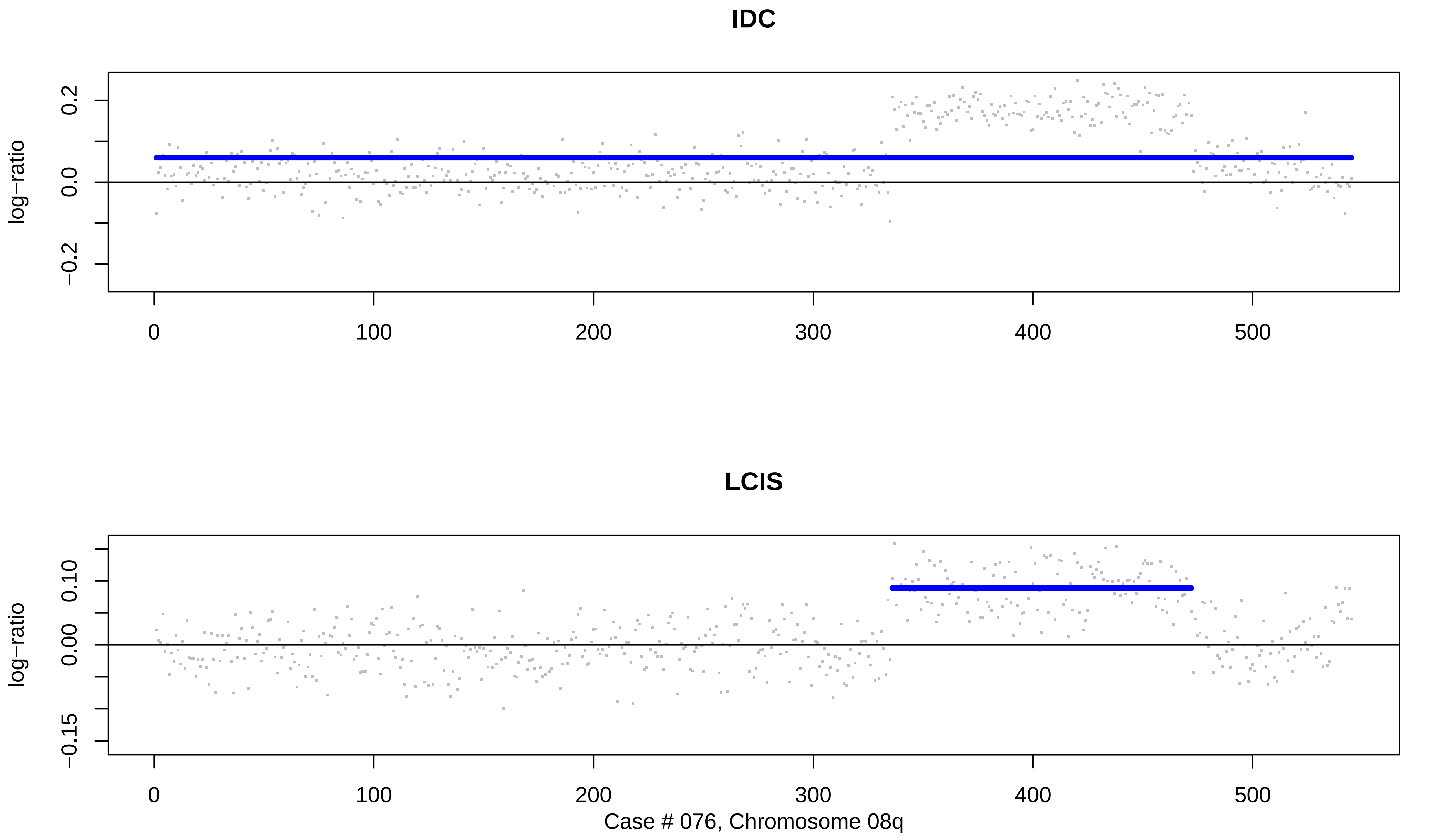

IDC
log−ratio
0.2
0.0
−0.2
0
100
200
300
400
500
LCIS
log−ratio
0.10
0.00
−0.15
0
100
200
300
400
500
Case # 076, Chromosome 08q

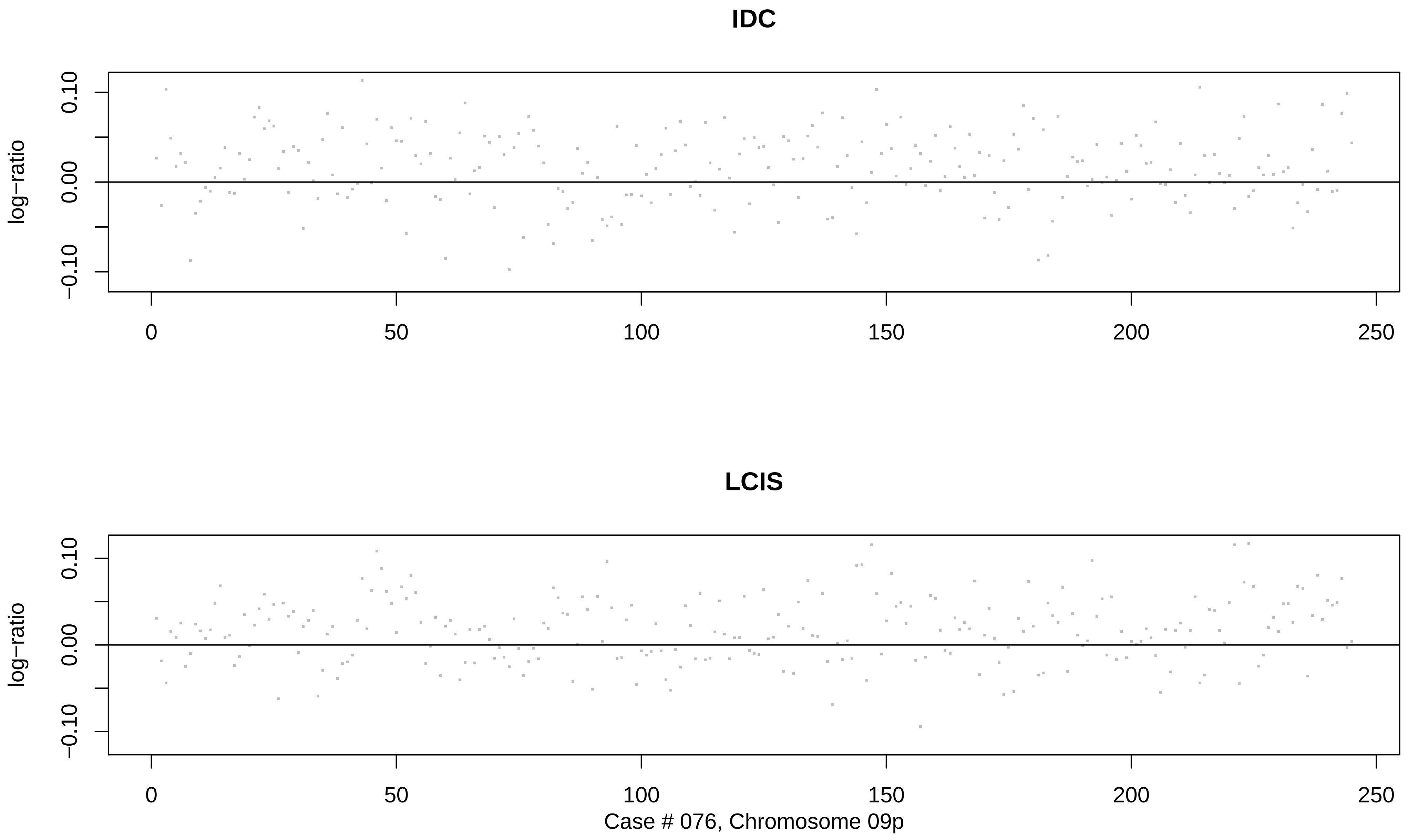
IDC
log−ratio
0.10
0.00
−0.10
0
50
100
150
200
250
LCIS
log−ratio
0.10
0.00
−0.10
0
50
100
150
200
250
Case # 076, Chromosome 09p

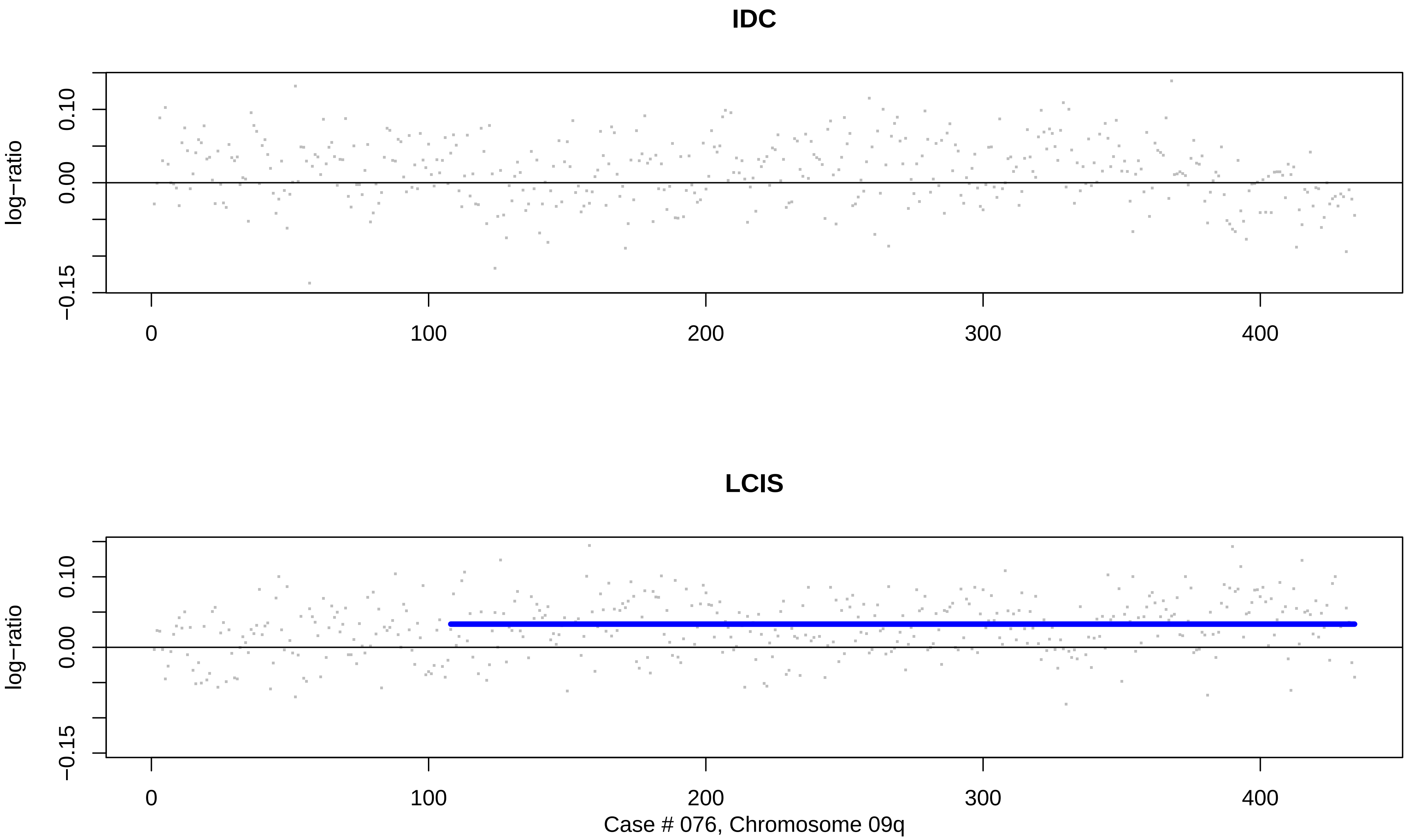
IDC
log−ratio
0.10
0.00
−0.15
0
100
200
300
400
LCIS
log−ratio
0.10
0.00
−0.15
0
100
200
300
400
Case # 076, Chromosome 09q

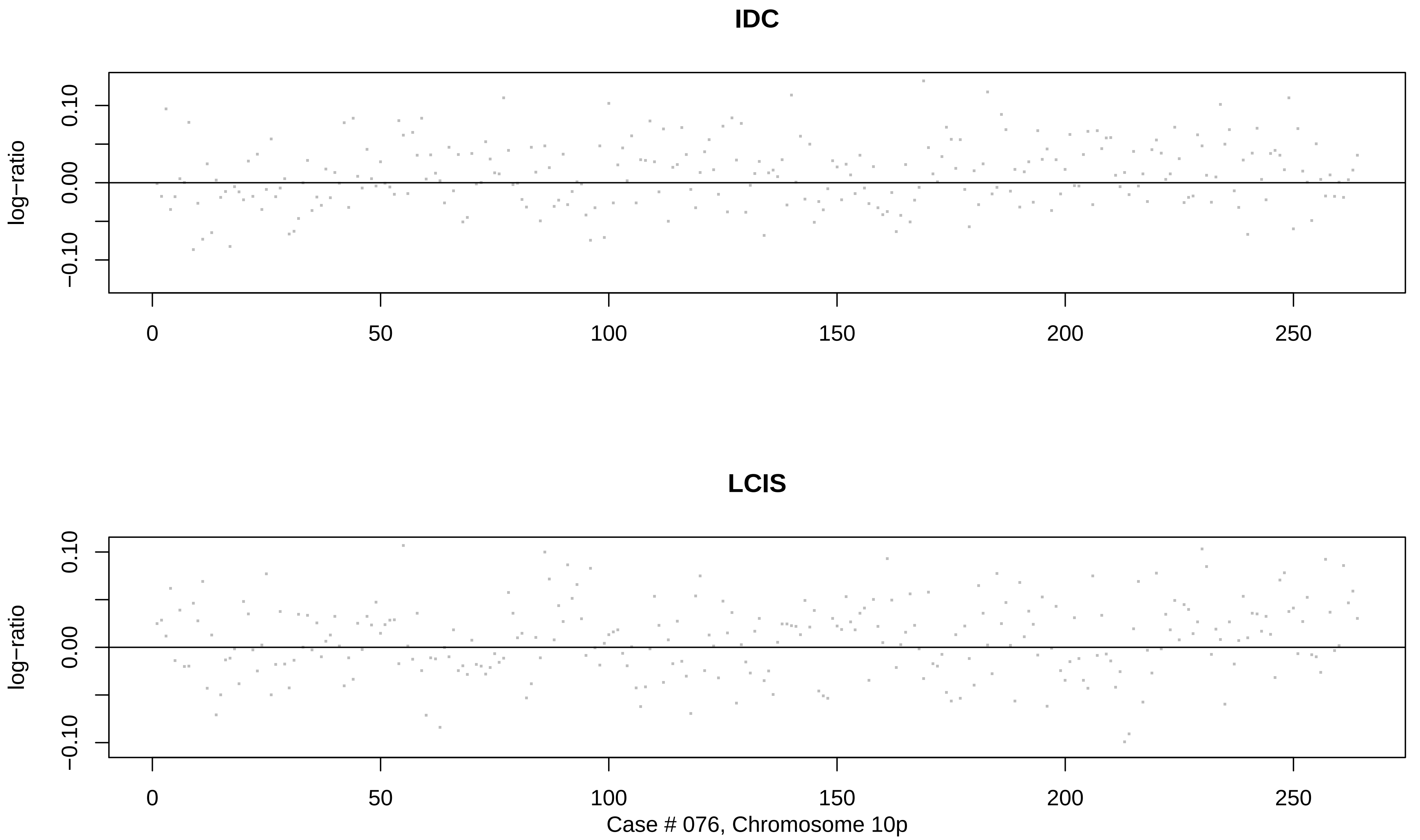
IDC
log−ratio
−0.10
0.00
0.10
0
50
100
150
200
250
LCIS
log−ratio
−0.10
0.00
0.10
0
50
100
150
200
250
Case # 076, Chromosome 10p

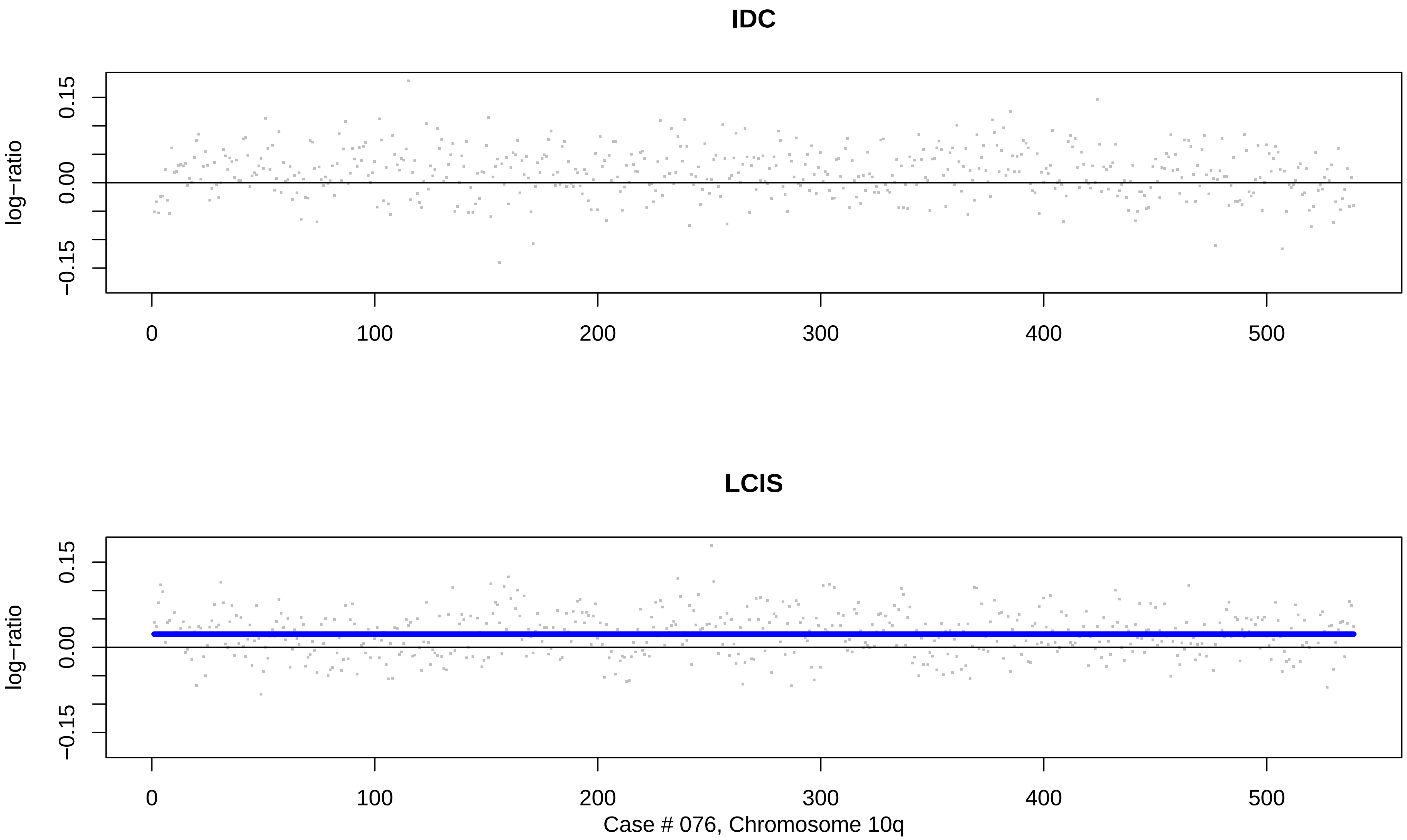
IDC
log−ratio
0.15
0.00
−0.15
0
100
200
300
400
500
LCIS
log−ratio
0.15
0.00
−0.15
0
100
200
300
400
500
Case # 076, Chromosome 10q

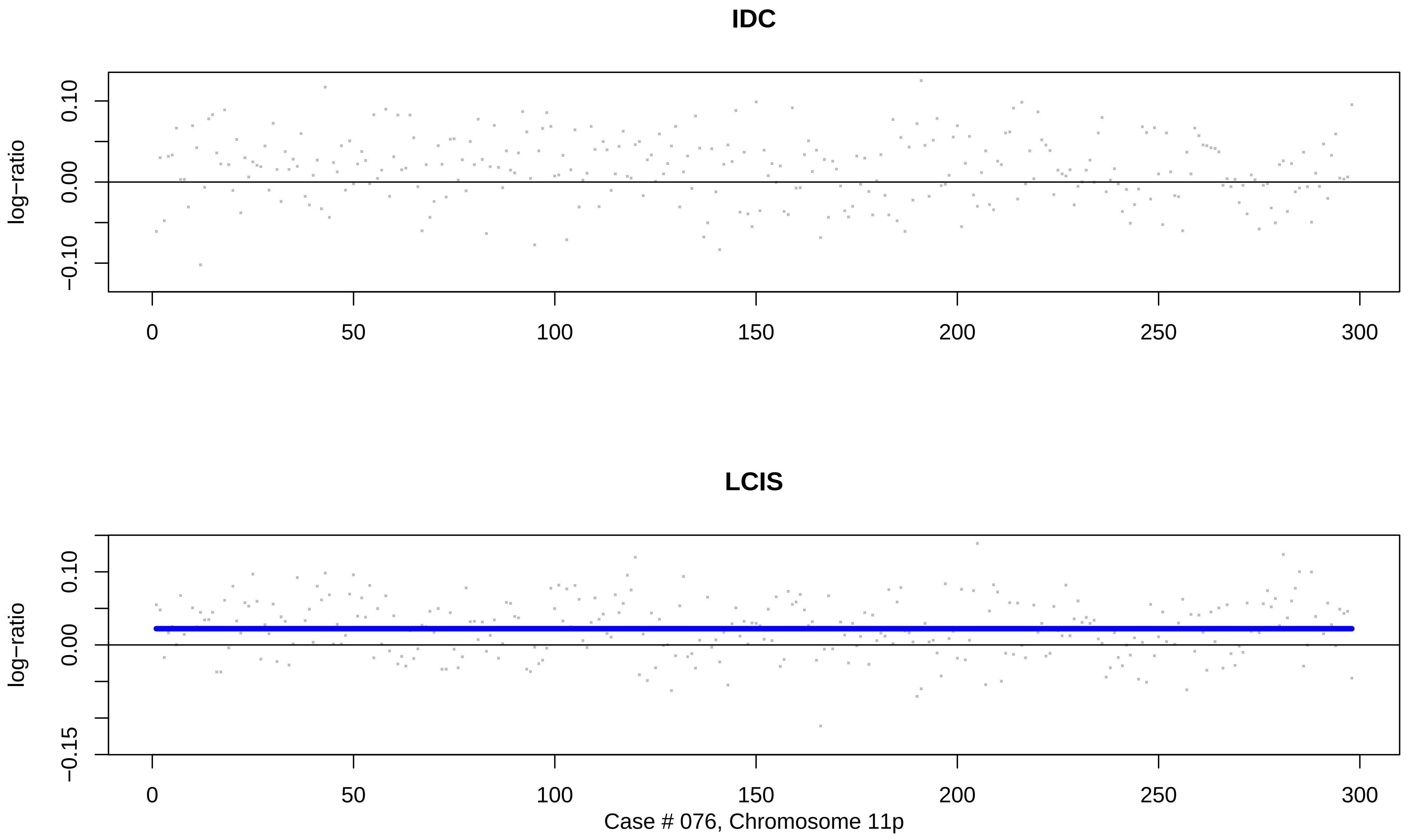
IDC
log−ratio
0.10
0.00
−0.10
0
50
100
150
200
250
300
LCIS
log−ratio
0.10
0.00
−0.15
0
50
100
150
200
250
300
Case # 076, Chromosome 11p

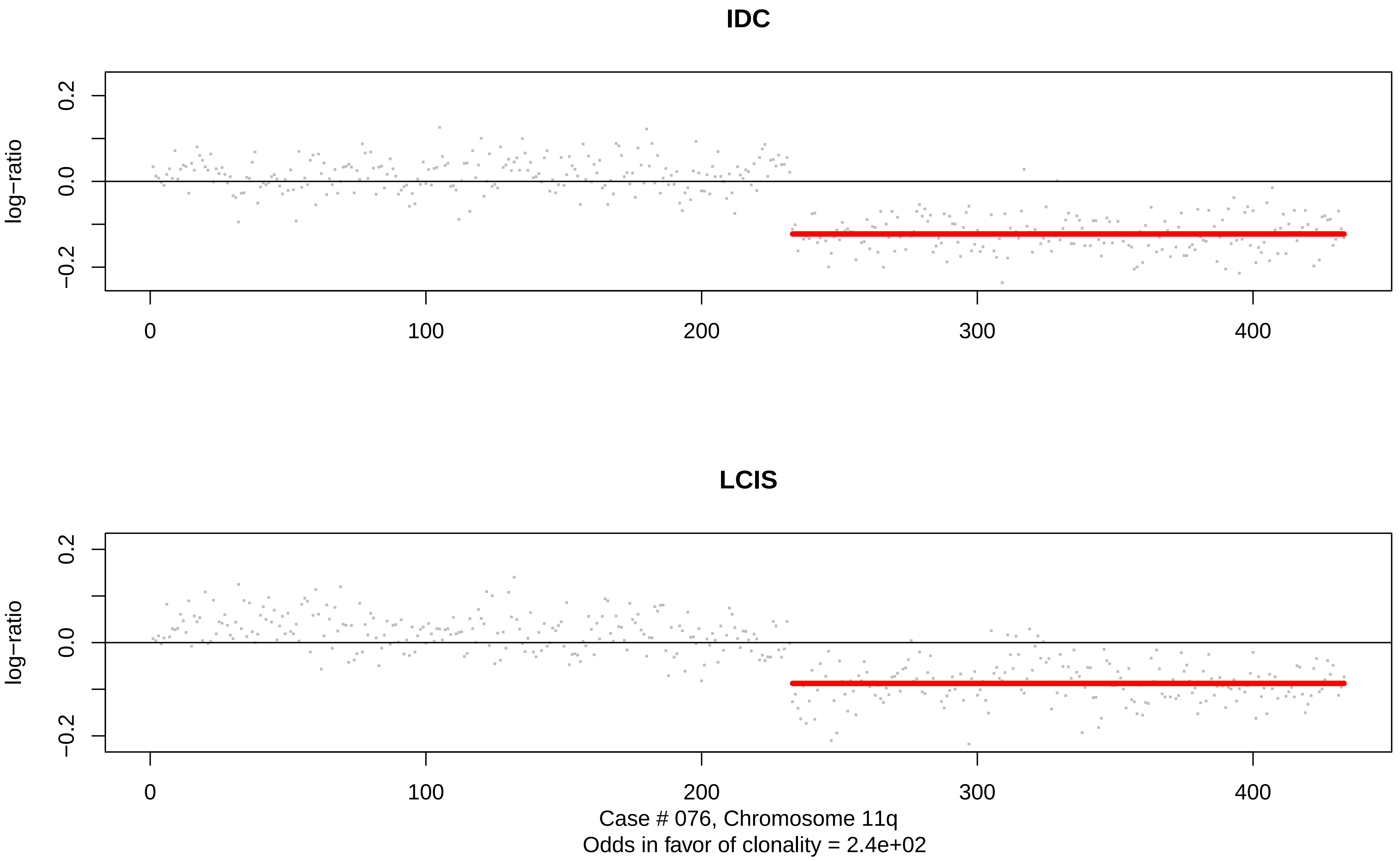

IDC
log−ratio
0.2
0.0
−0.2
0
100
200
300
400
LCIS
log−ratio
0.2
0.0
−0.2
0
100
200
300
400
Case # 076, Chromosome 11q
Odds in favor of clonality = 2.4e+02

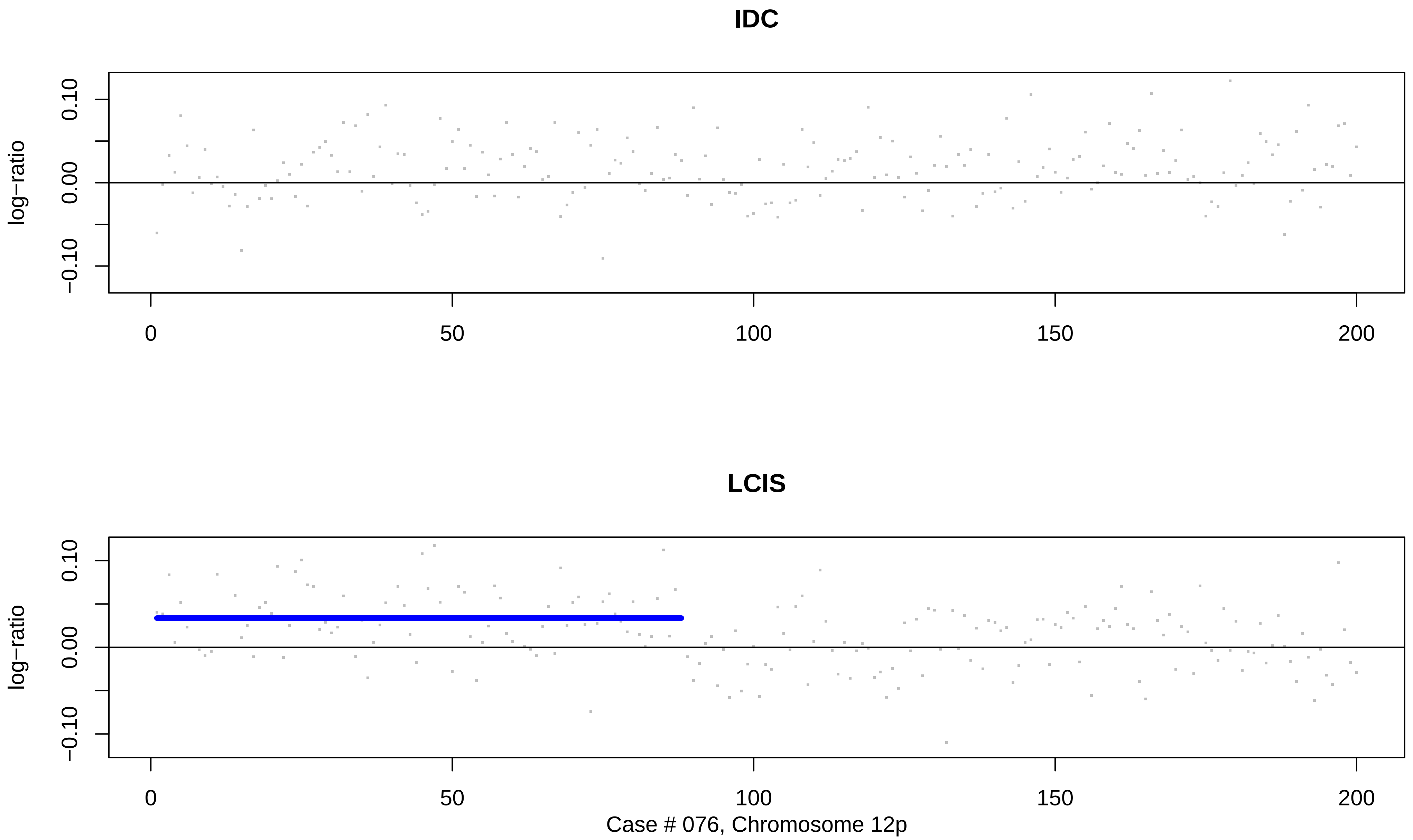
IDC
log−ratio
0.10
0.00
−0.10
0
50
100
150
200
LCIS
log−ratio
0.10
0.00
−0.10
0
50
100
150
200
Case # 076, Chromosome 12p

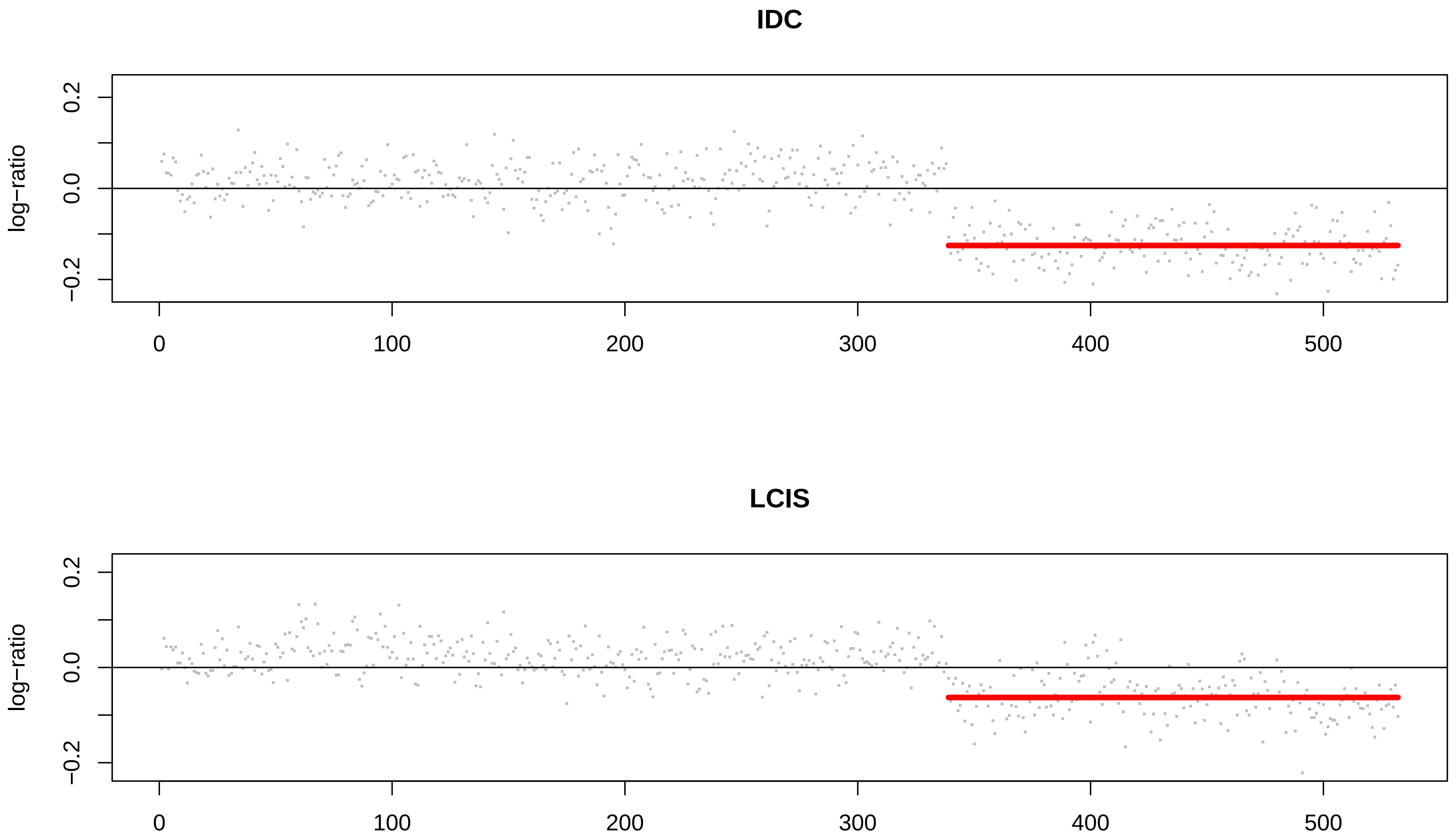

Case # 076, Chromosome 12q
Odds in favor of clonality = 4e+02

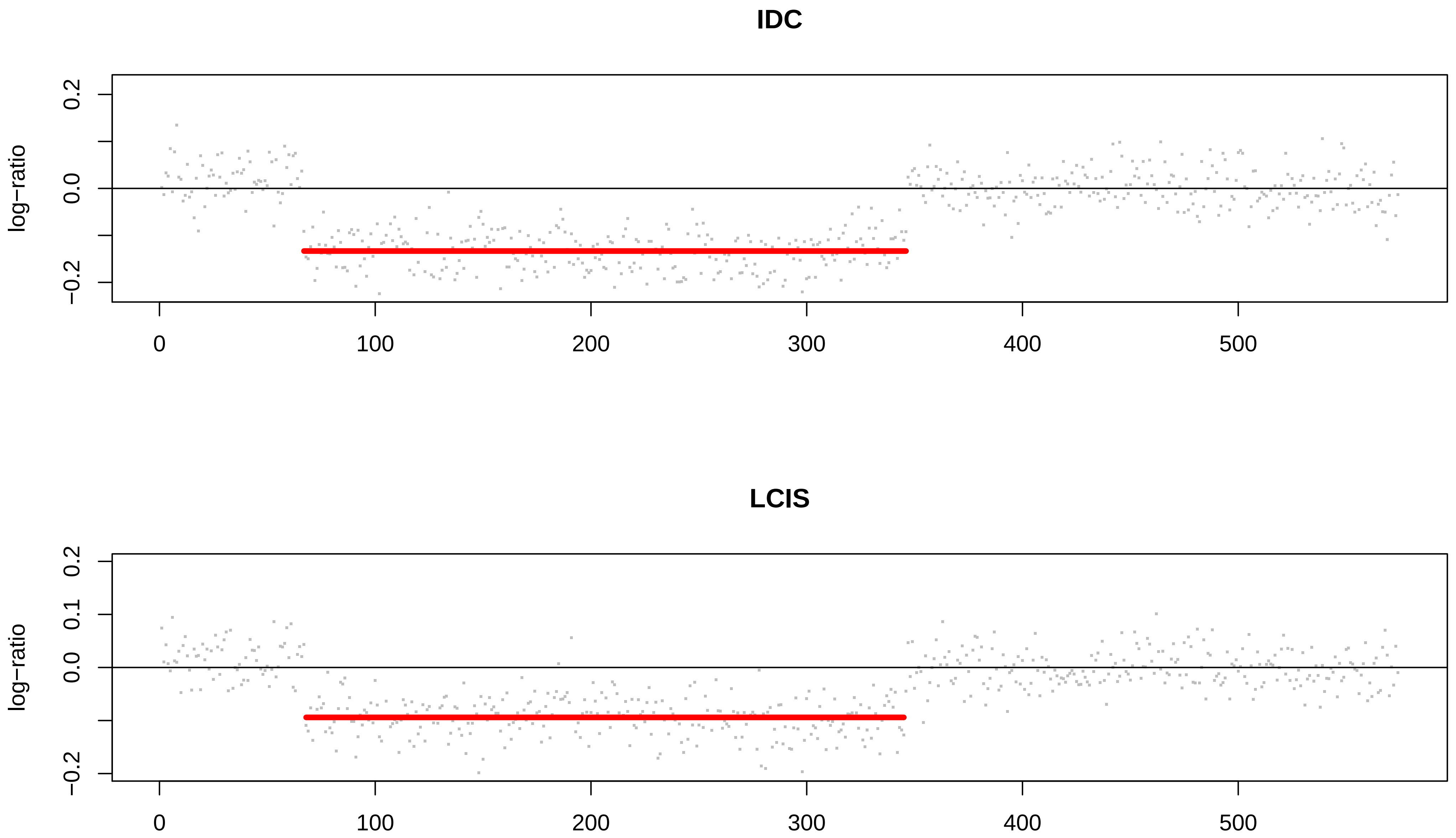

Case # 076, Chromosome 13q
Odds in favor of clonality = 1.2e+02

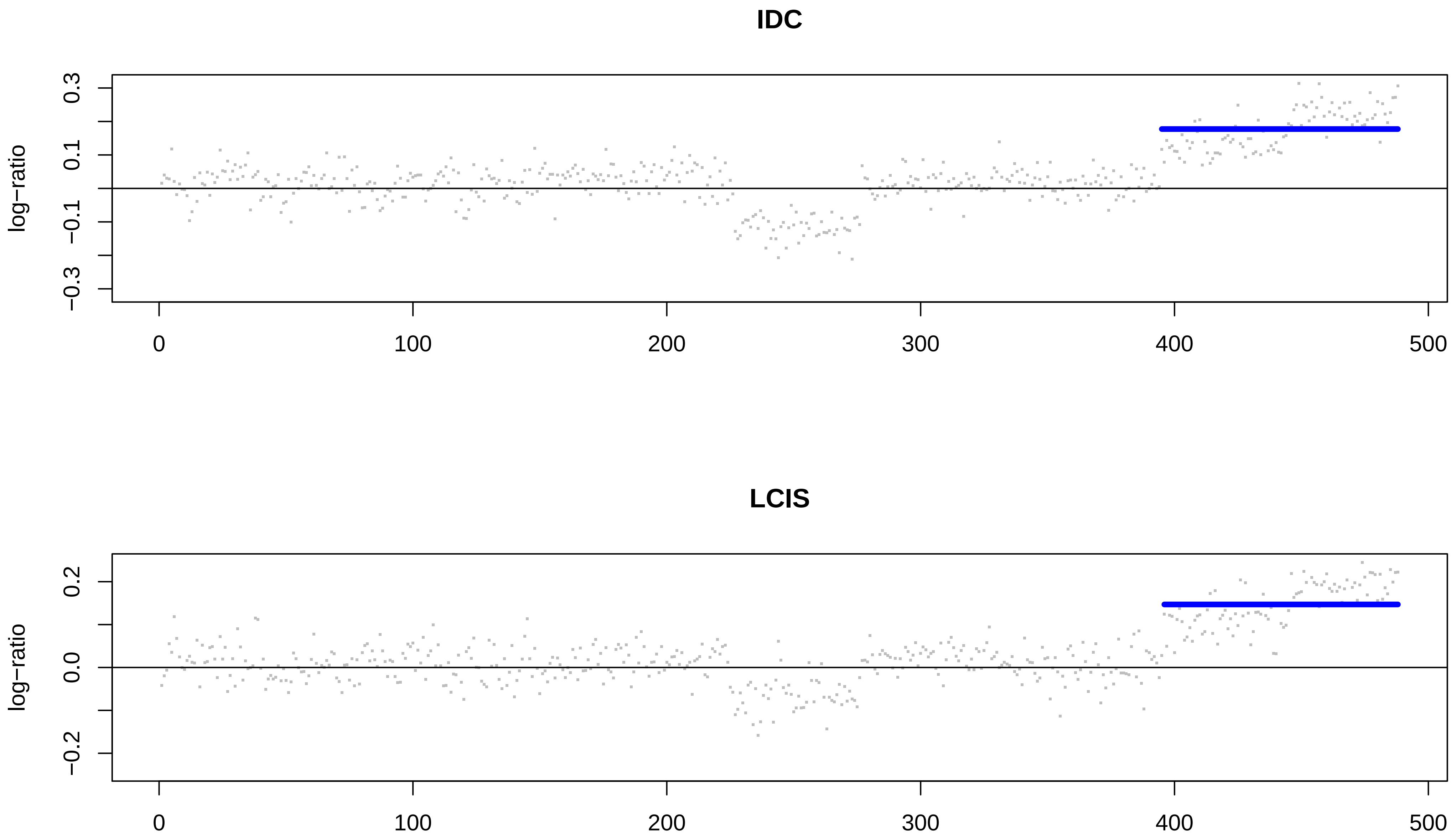

Case # 076, Chromosome 14q
Odds in favor of clonality = 1.2e+02

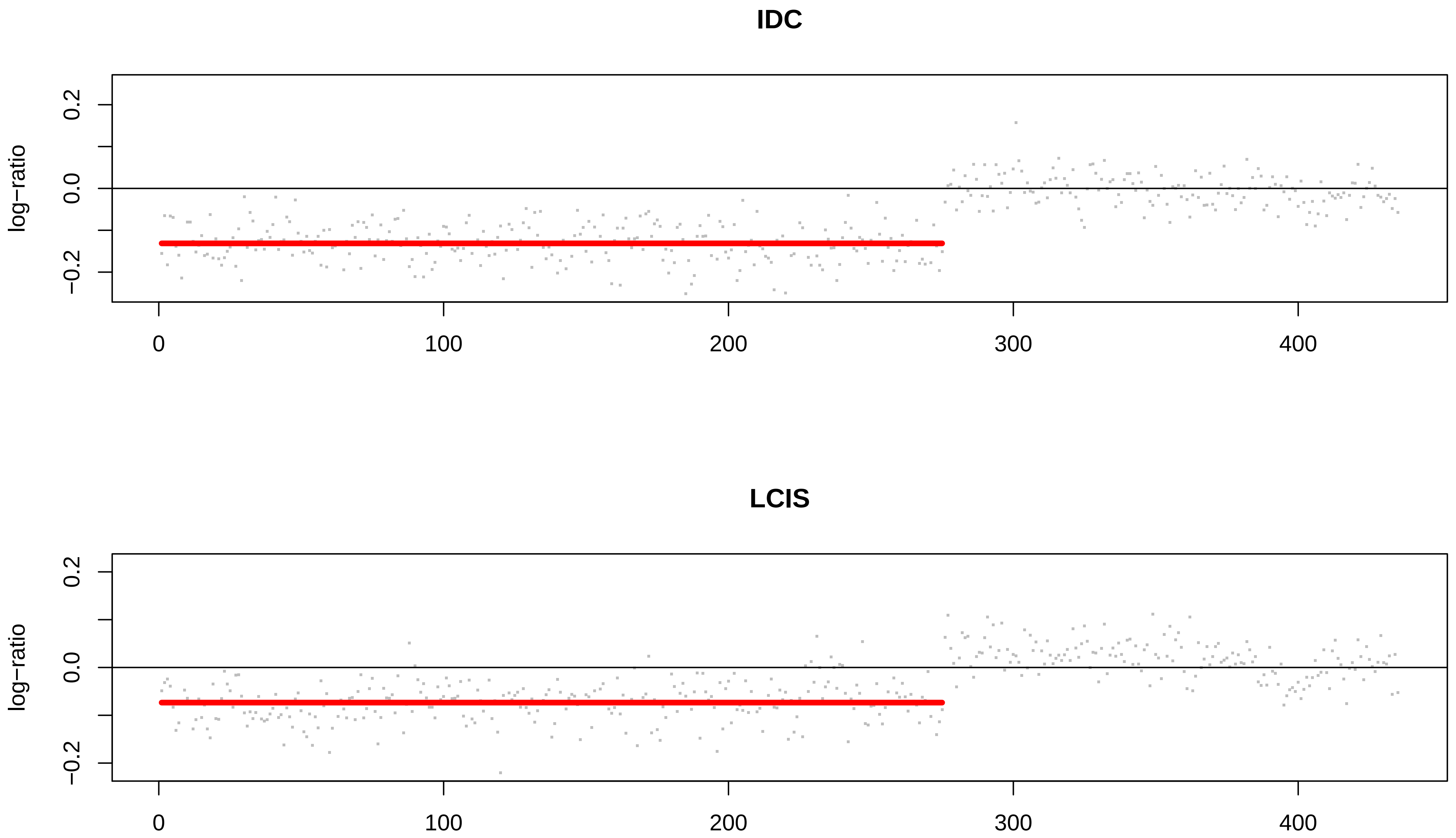

Case # 076, Chromosome 15q
Odds in favor of clonality = 3.1e+02

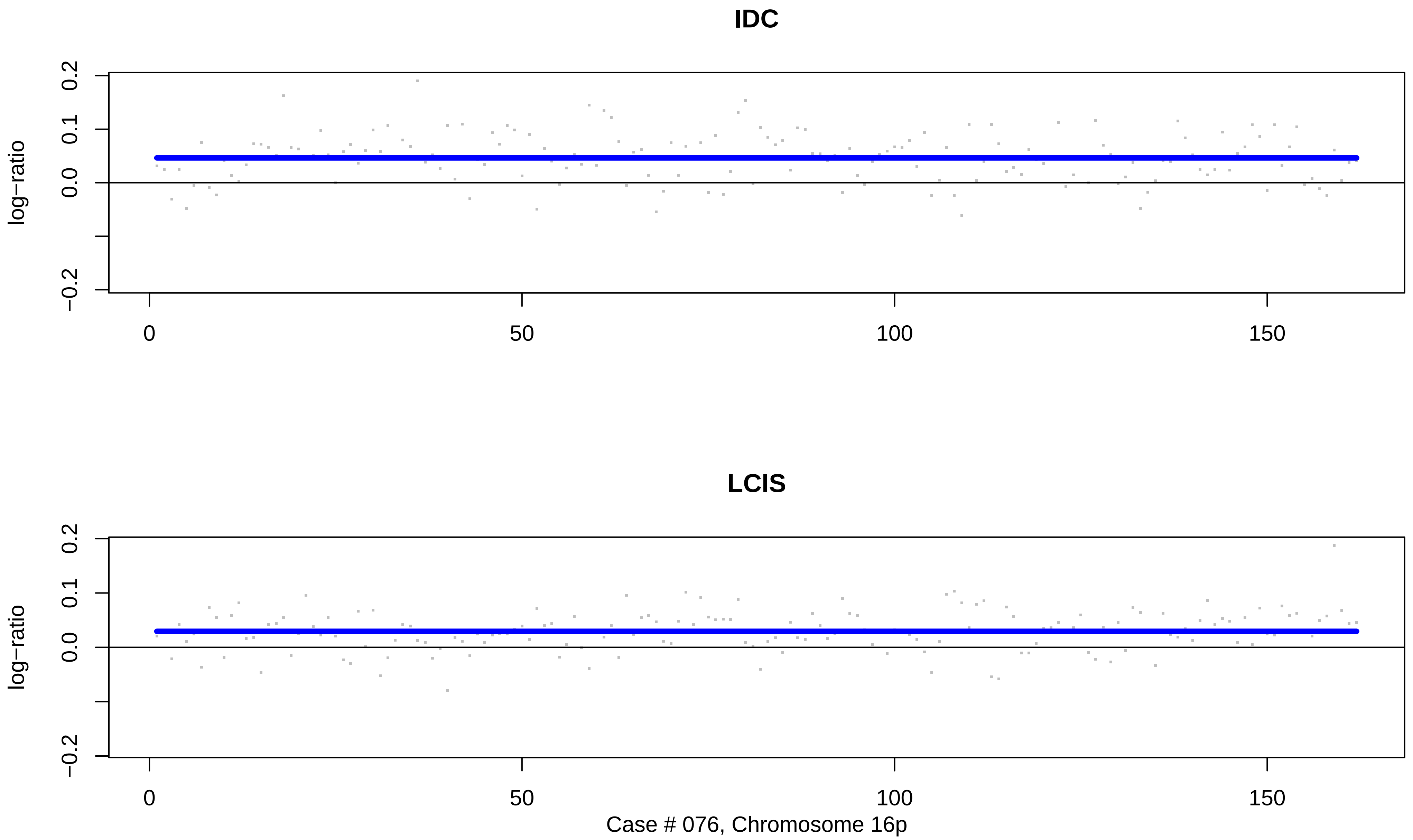
IDC
log−ratio
−0.2
0.0
0.1
0.2
0
50
100
150
LCIS
log−ratio
−0.2
0.0
0.1
0.2
0
50
100
150
Case # 076, Chromosome 16p

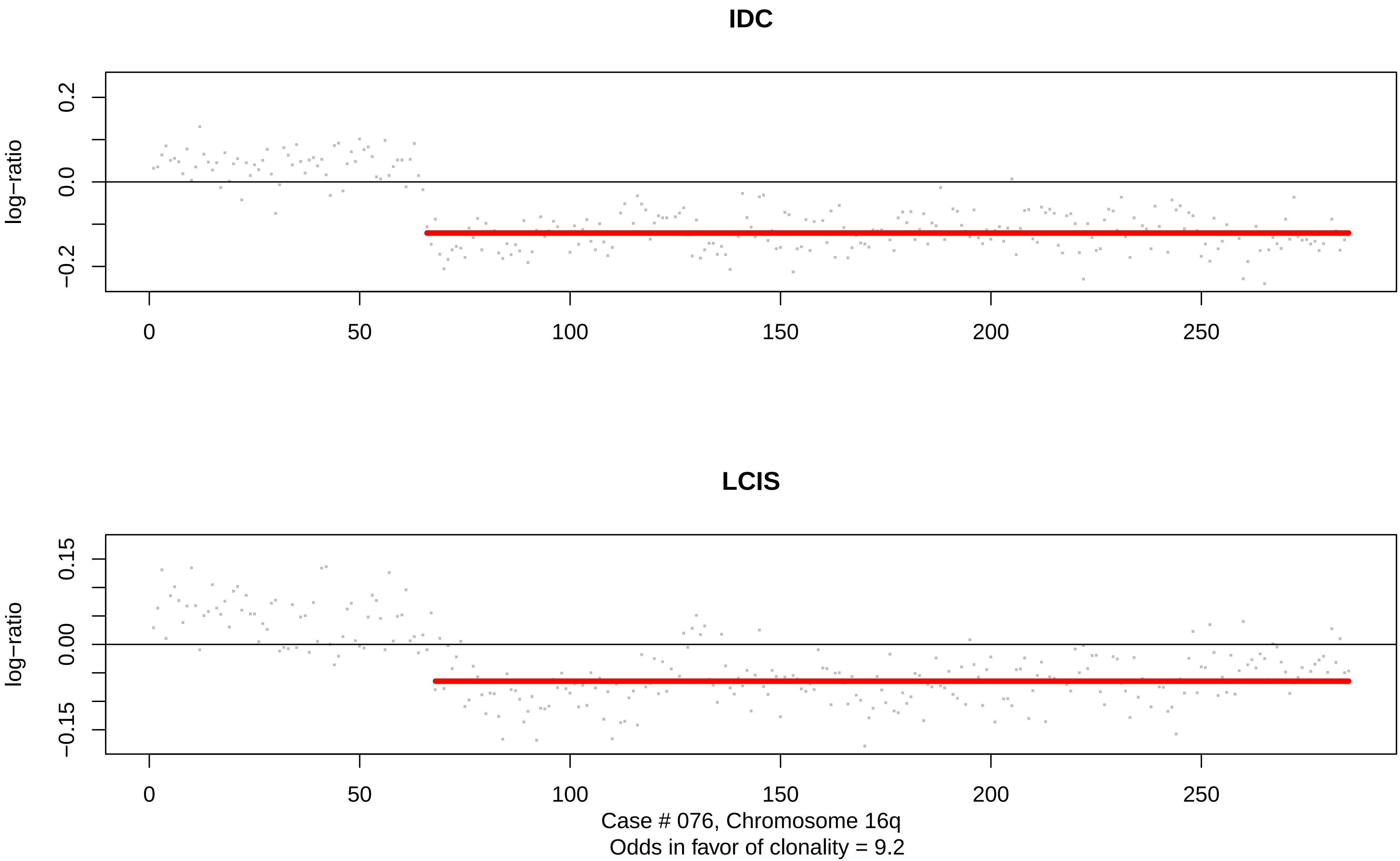
IDC
log−ratio
0.2
0.0
−0.2
0
50
100
150
200
250
LCIS
log−ratio
0.15
0.00
−0.15
0
50
100
150
200
250
Case # 076, Chromosome 16q
Odds in favor of clonality = 9.2

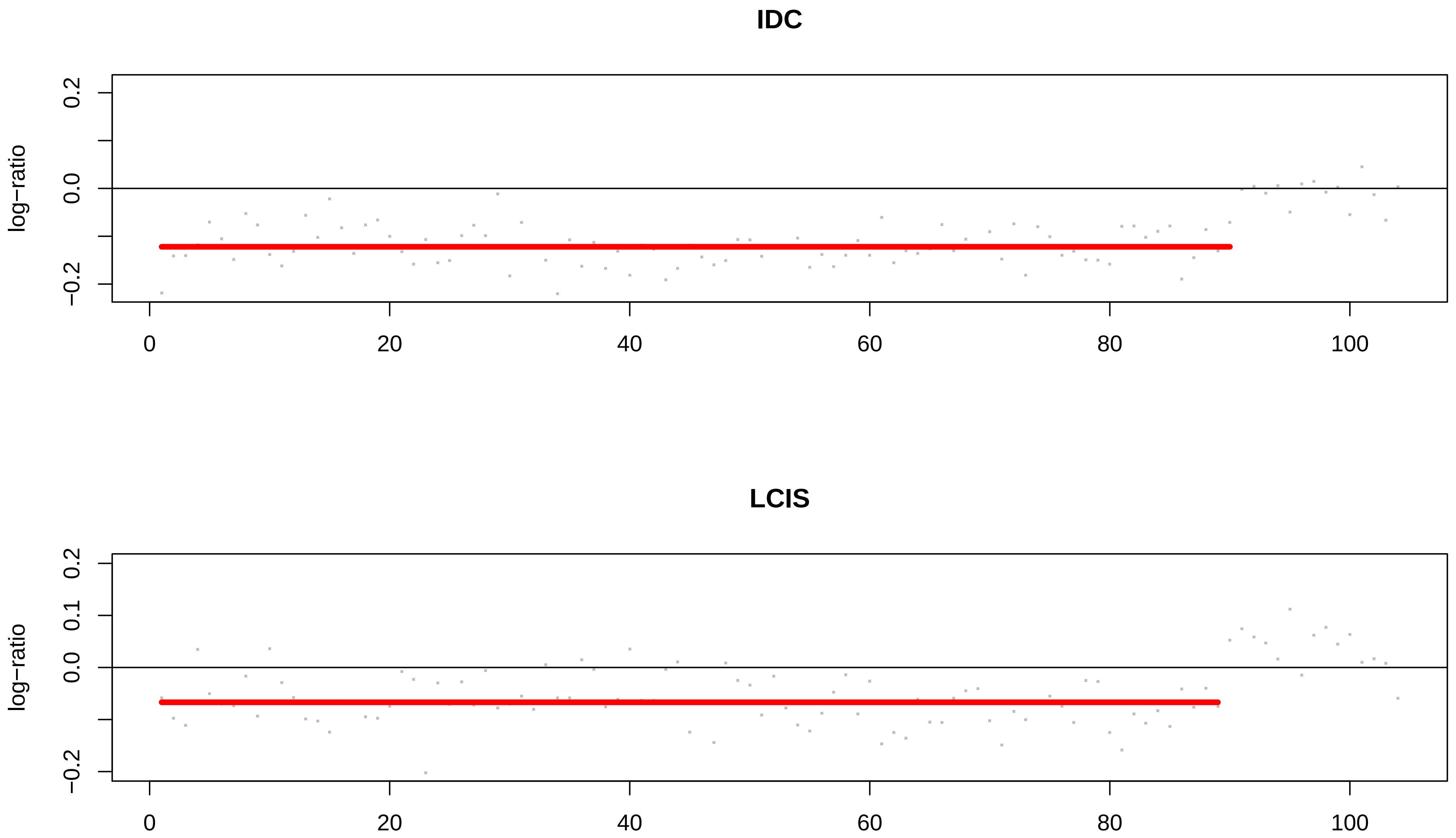

Case # 076, Chromosome 17p
Odds in favor of clonality = 10.5

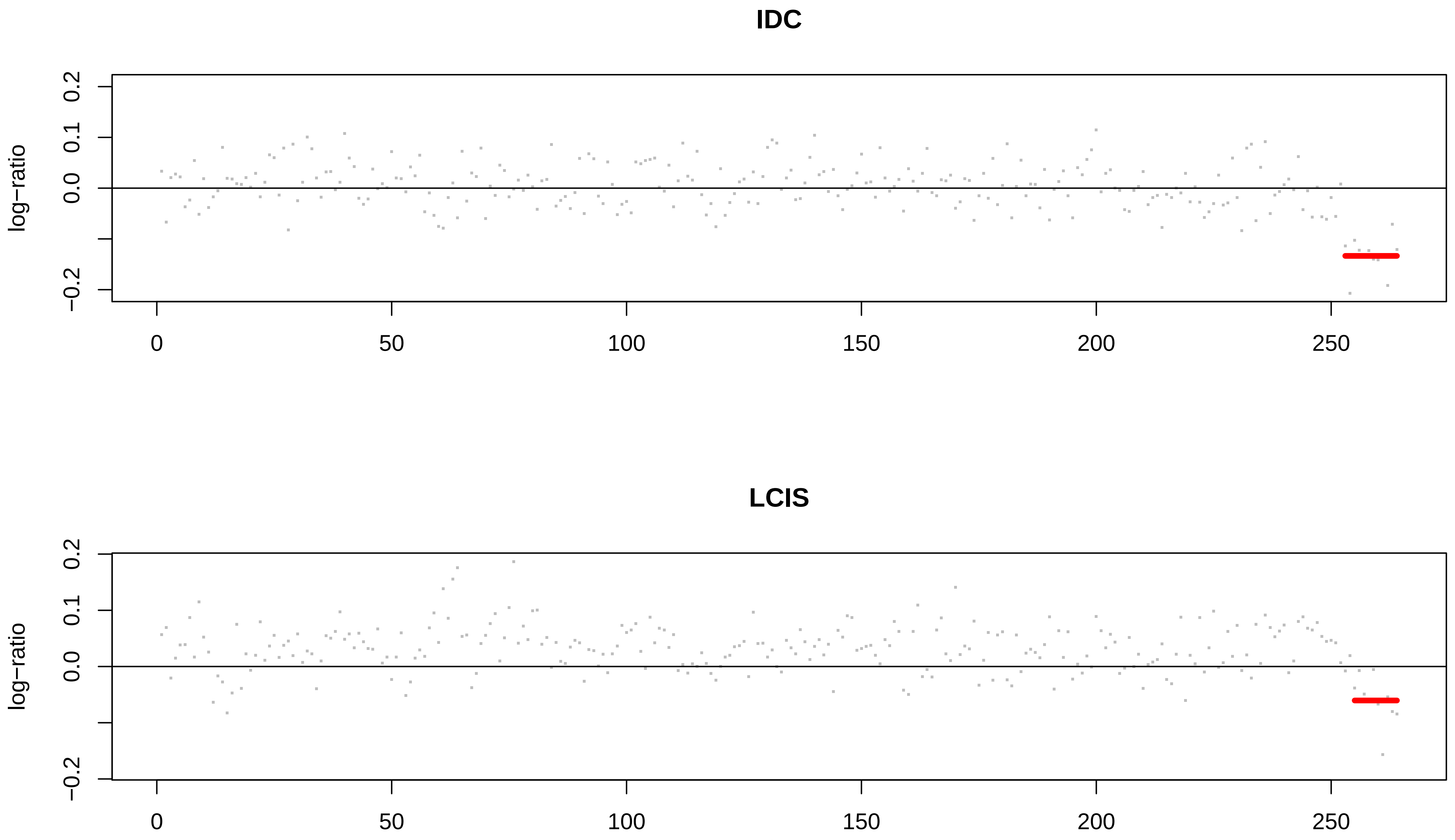

Case # 076, Chromosome 17q
Odds in favor of clonality = 24.2

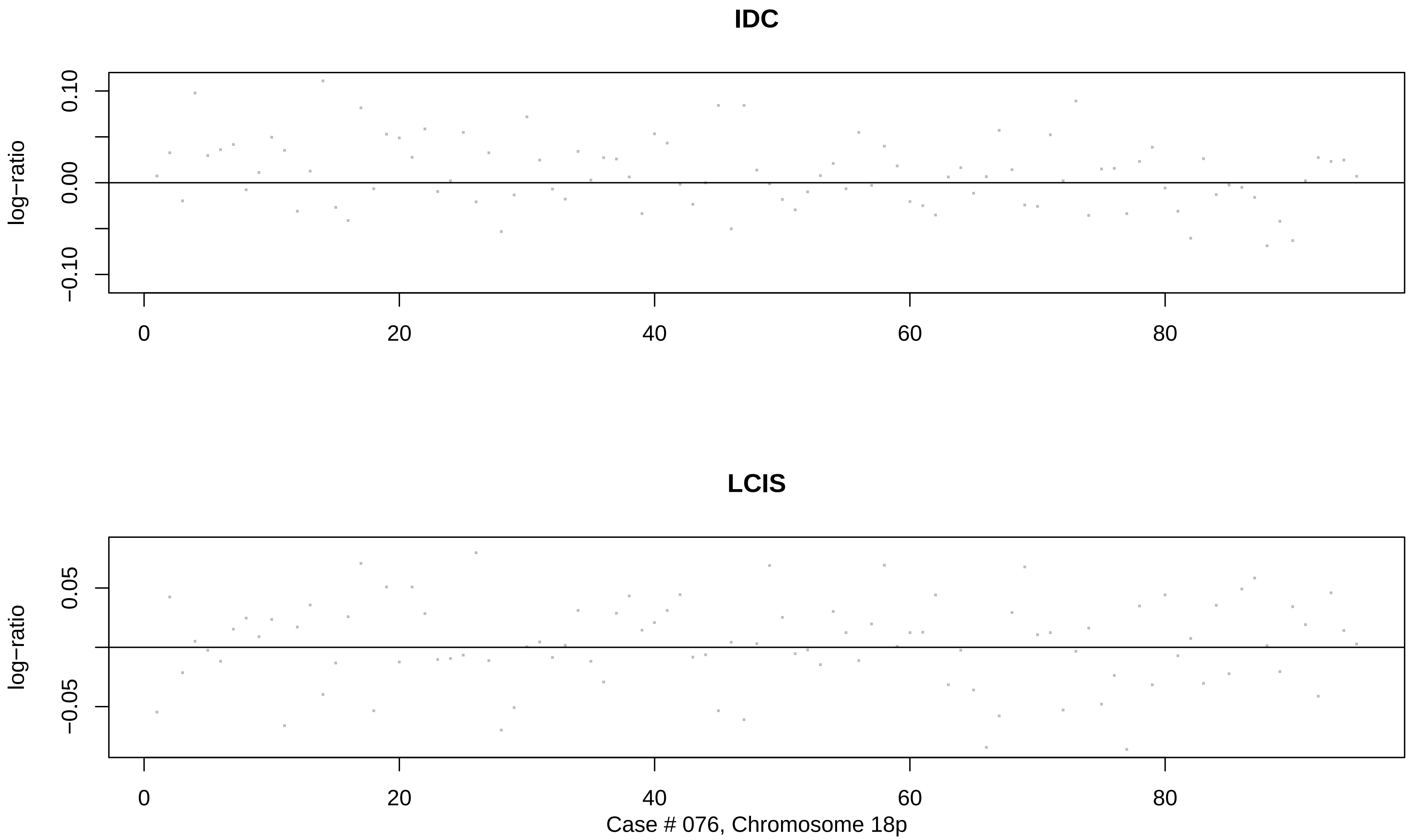

IDC
LCIS
log−ratio
0.10
0.00
−0.10
0.05
−0.05
0
20
40
60
80
Case # 076, Chromosome 18p

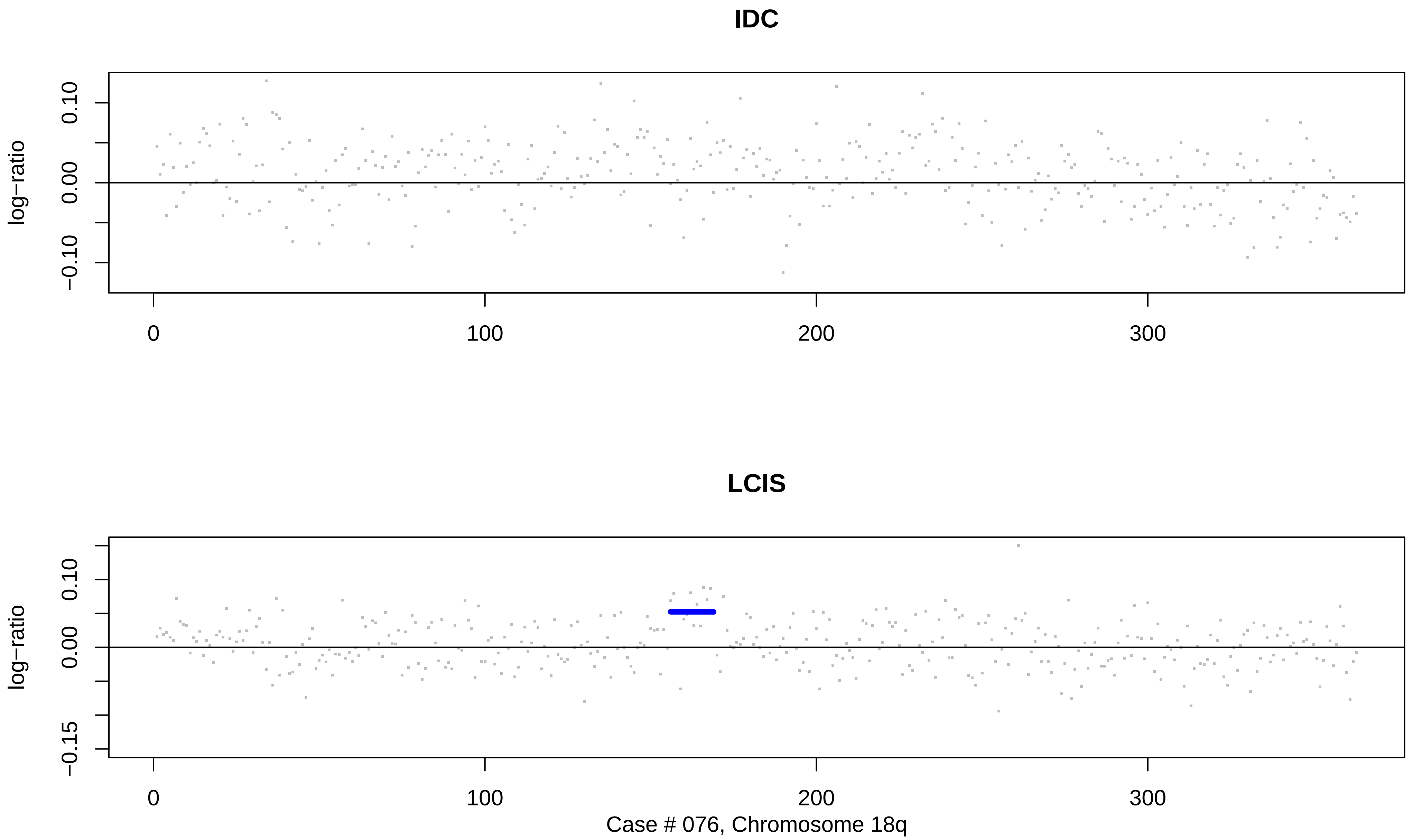
IDC
LCIS
log−ratio
0.10
0.00
−0.10
−0.15
0
100
200
300
Case # 076, Chromosome 18q

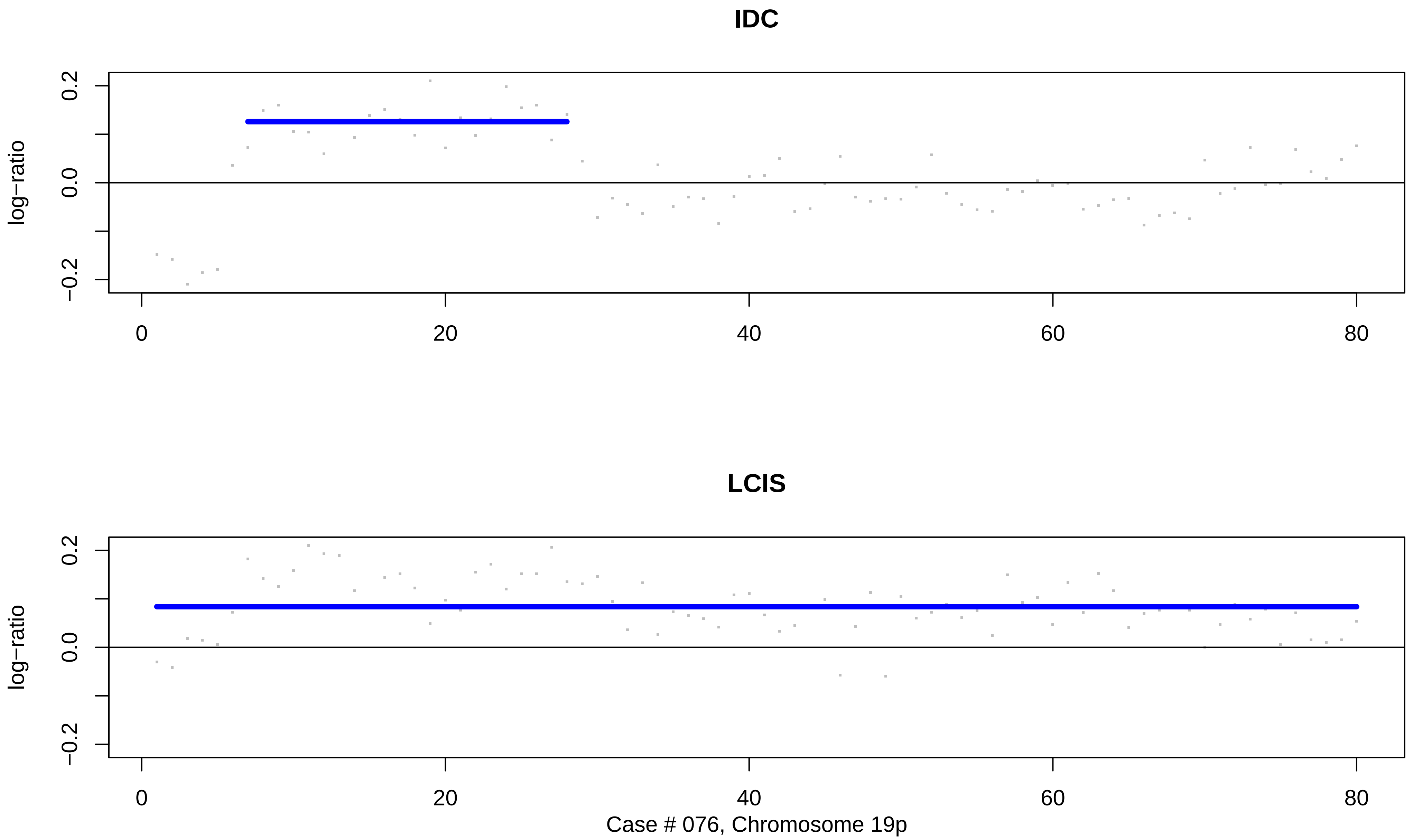
IDC
log−ratio
0.2
0.0
−0.2
0
20
40
60
80
LCIS
log−ratio
0.2
0.0
−0.2
0
20
40
60
80
Case # 076, Chromosome 19p

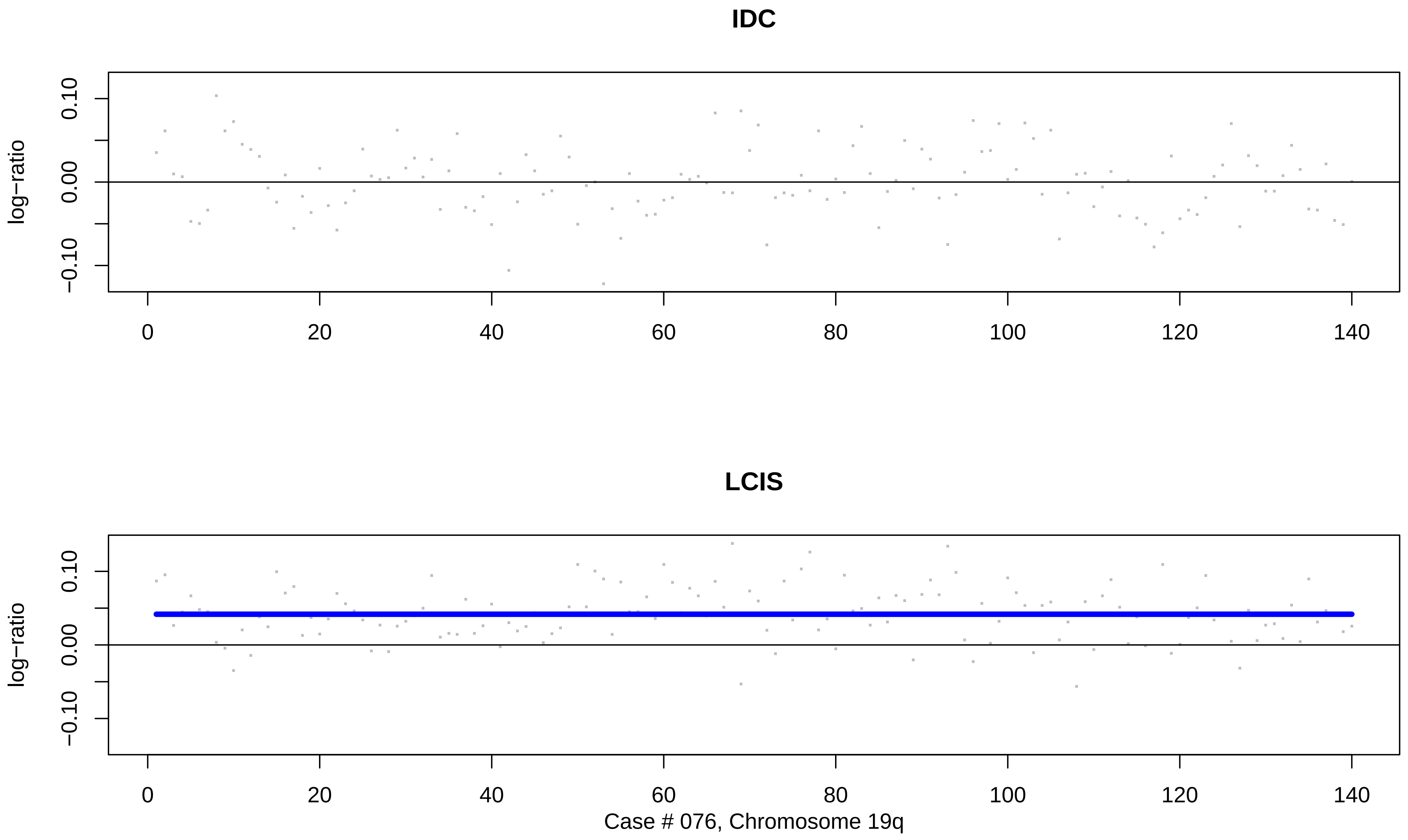

IDC
log−ratio
0.10
0.00
−0.10
0
20
40
60
80
100
120
140
LCIS
log−ratio
0.10
0.00
−0.10
0
20
40
60
80
100
120
140
Case # 076, Chromosome 19q

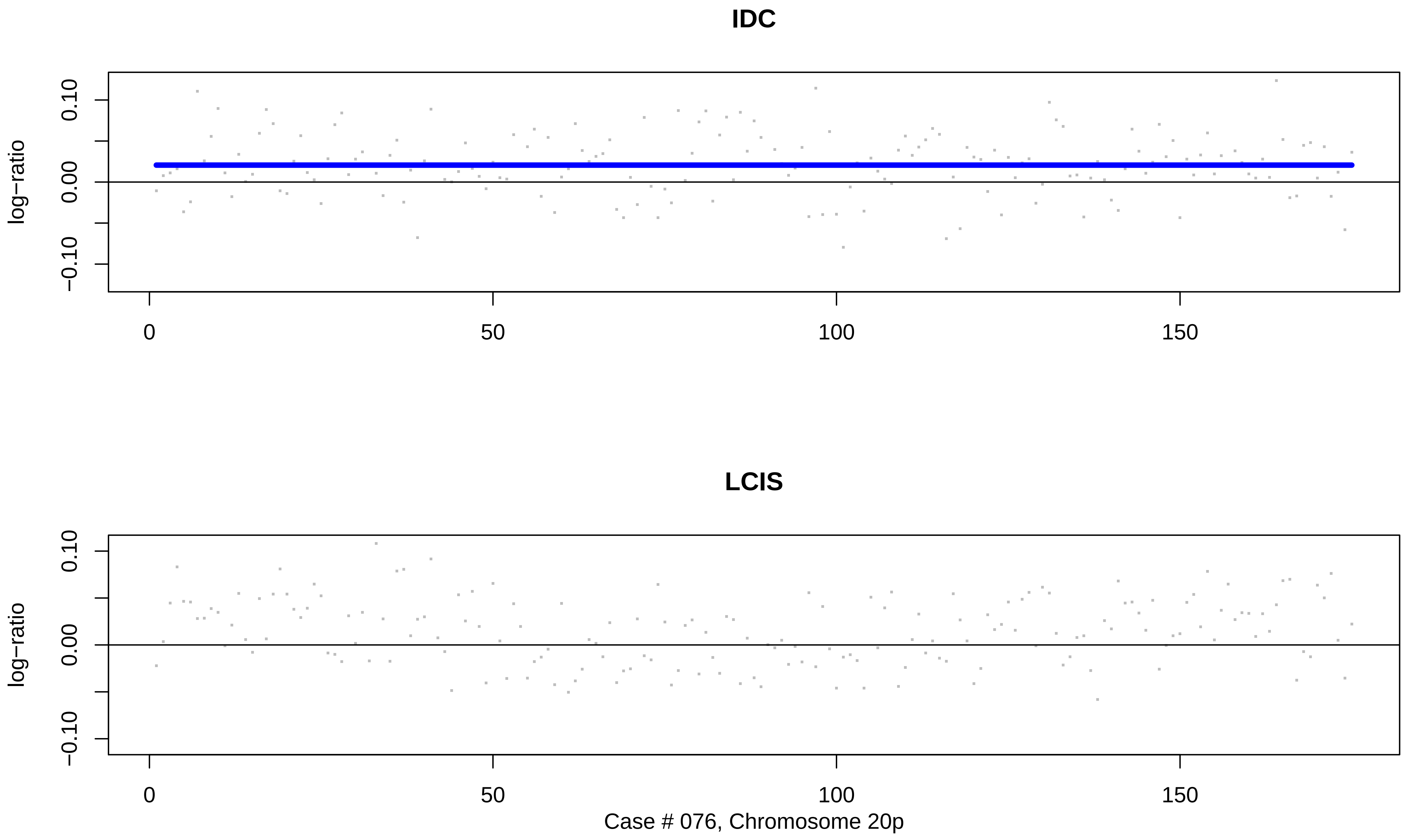
IDC
log−ratio
0.10
0.00
−0.10
0
50
100
150
LCIS
log−ratio
0.10
0.00
−0.10
0
50
100
150
Case # 076, Chromosome 20p

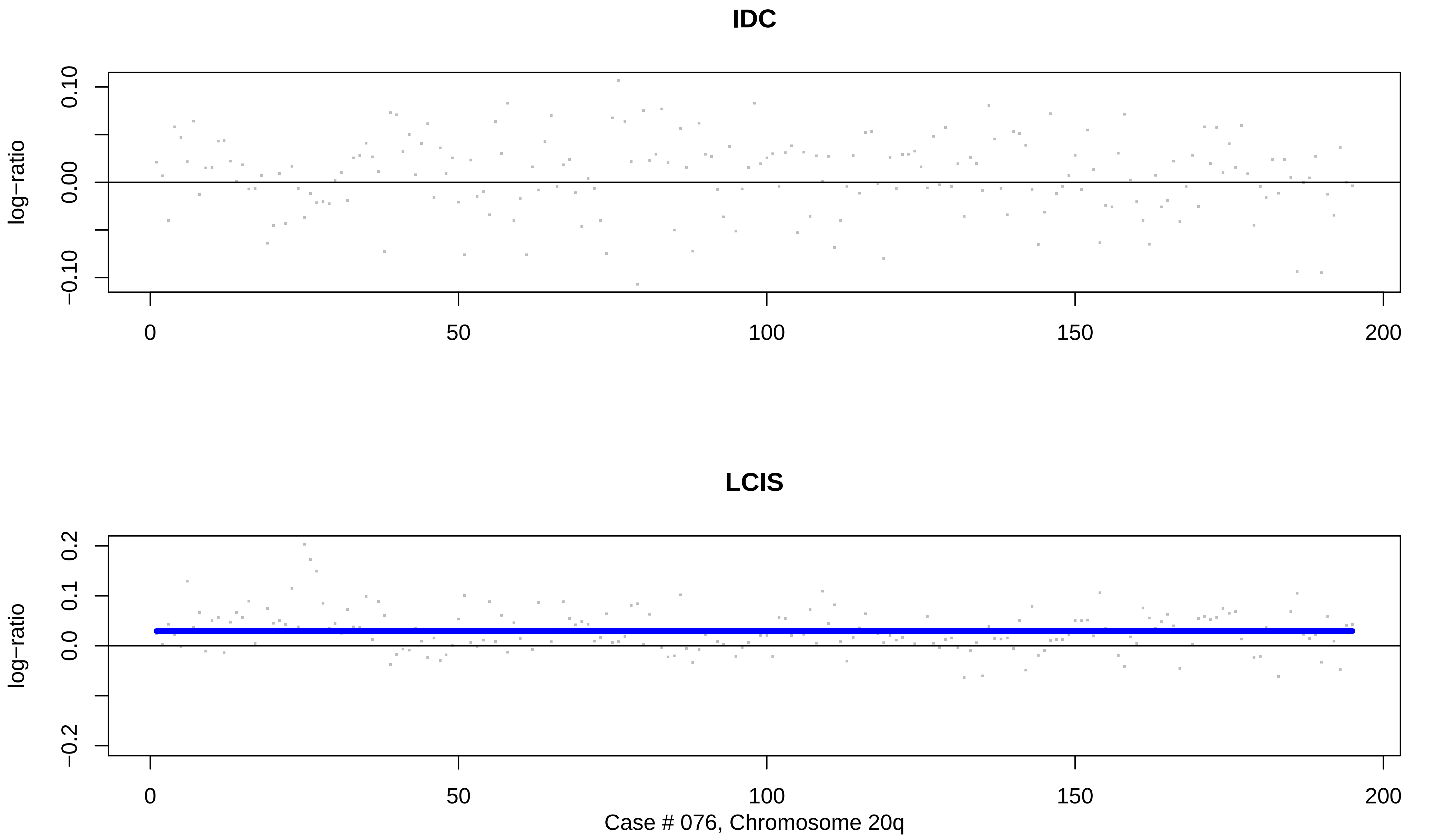
IDC
log−ratio
0.10
0.00
−0.10
0
50
100
150
200
LCIS
log−ratio
0.2
0.1
0.0
−0.2
0
50
100
150
200
Case # 076, Chromosome 20q

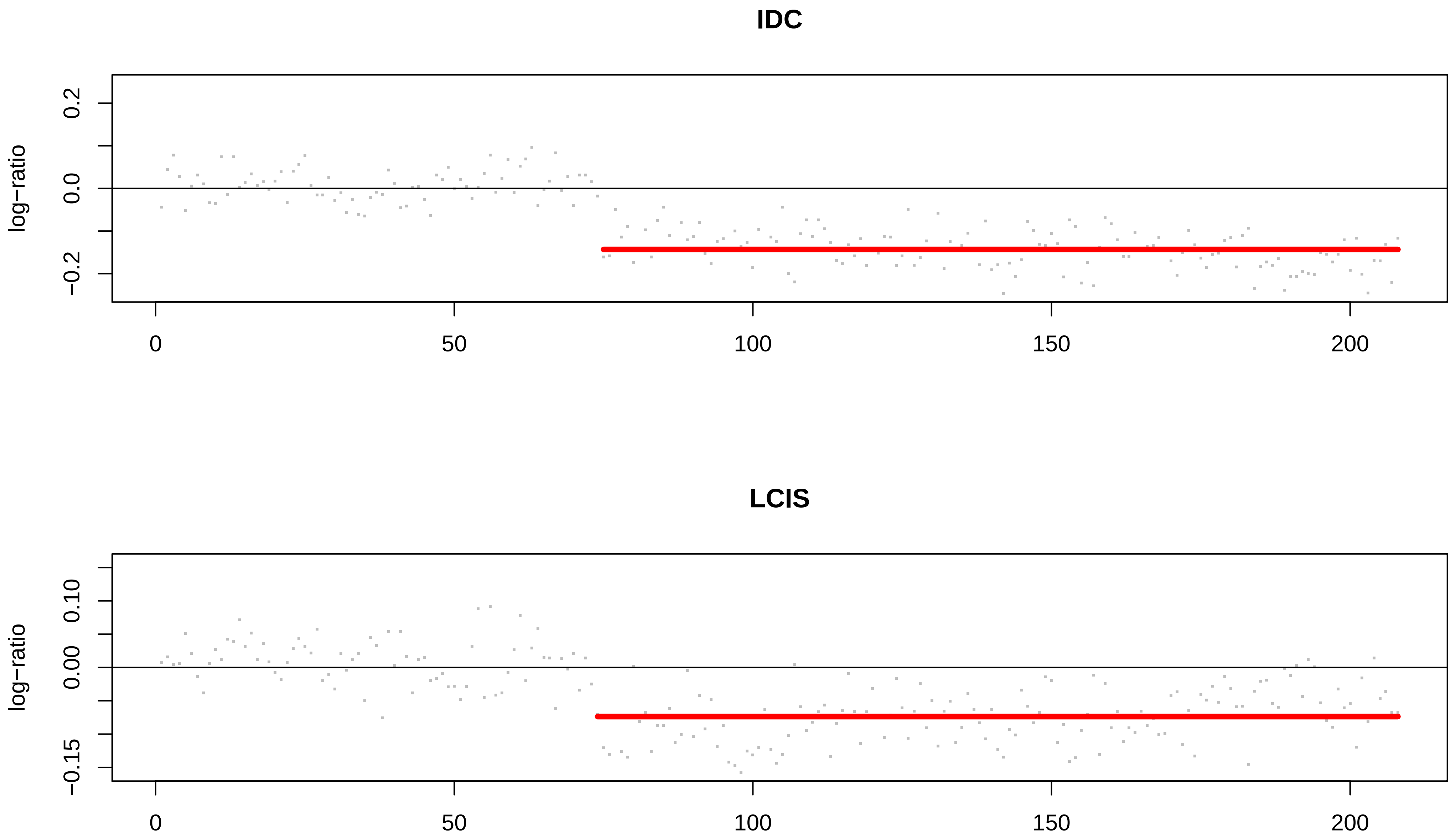

Case # 076, Chromosome 21q
Odds in favor of clonality = 29.3

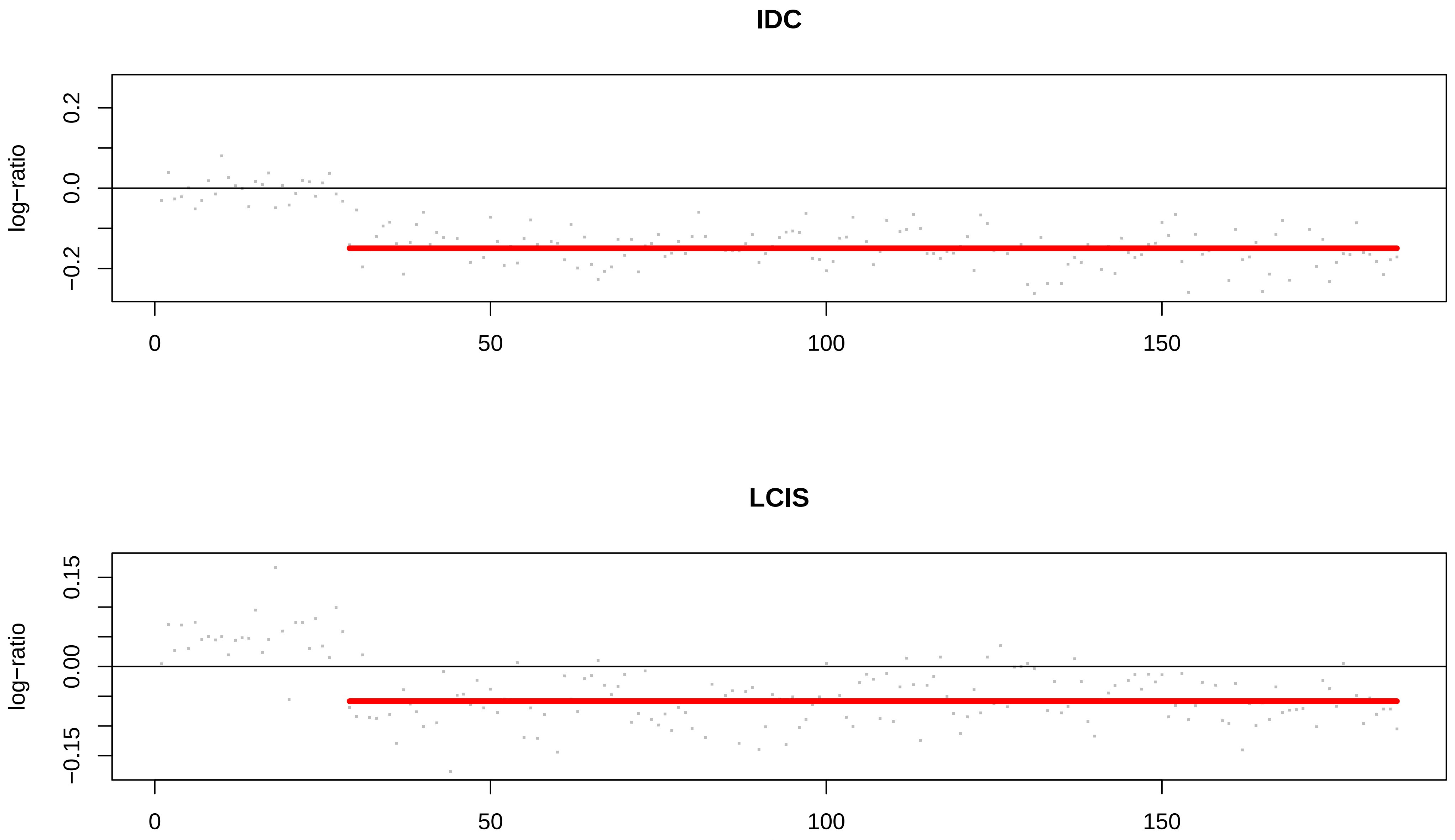

Case # 076, Chromosome 22q
Odds in favor of clonality = 71.5
