# Supplementary material for: Clonal relatedness between lobular carcinoma in situ and synchronous malignant lesions
Source: Breast Cancer Res. 2012 Jul 9;14(4):R103. doi: 10.1186/bcr3222 (PMC3680923; doi:10.1186/bcr3222)
Supplement: Additional file 4 — Magnified version of genome-wide plots with detailed marker plots and segmentation on a chromosome-arm-specific basis. [file bcr3222-S4.ZIP › Case 032.pdf]

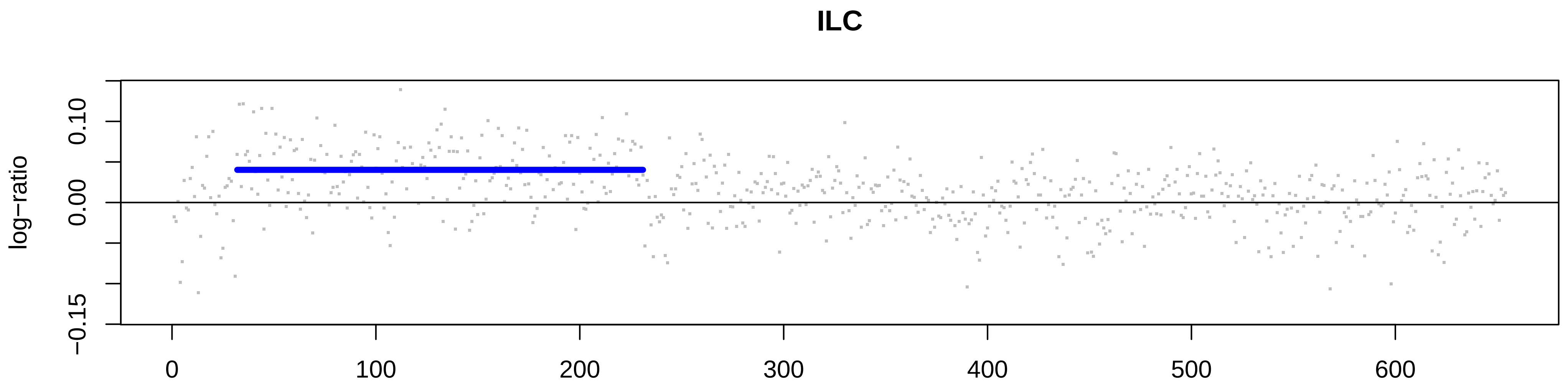
ILC
log−ratio
0.10
0.00
−0.15
0
100
200
300
400
500
600

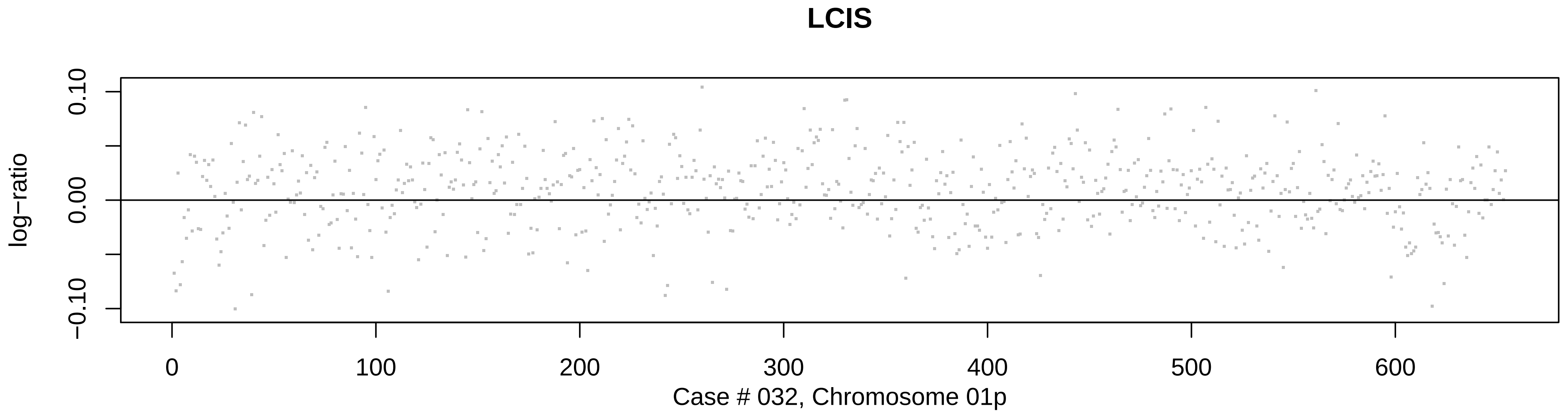
LCIS
log−ratio
0.10
0.00
−0.10
0
100
200
300
400
500
600
Case # 032, Chromosome 01p

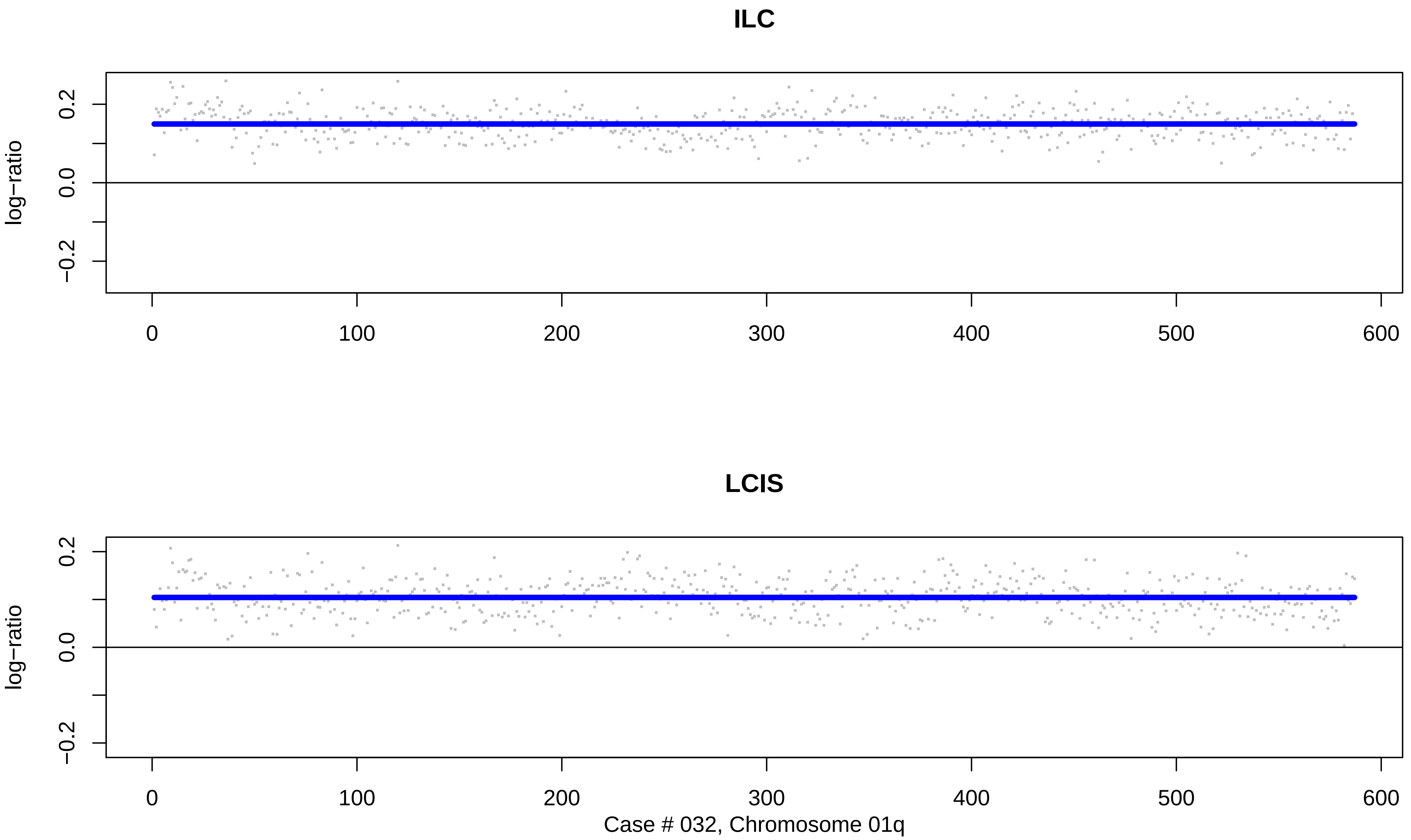
ILC
log−ratio
−0.2
0.0
0.2
0
100
200
300
400
500
600
LCIS
log−ratio
−0.2
0.0
0.2
0
100
200
300
400
500
600
Case # 032, Chromosome 01q

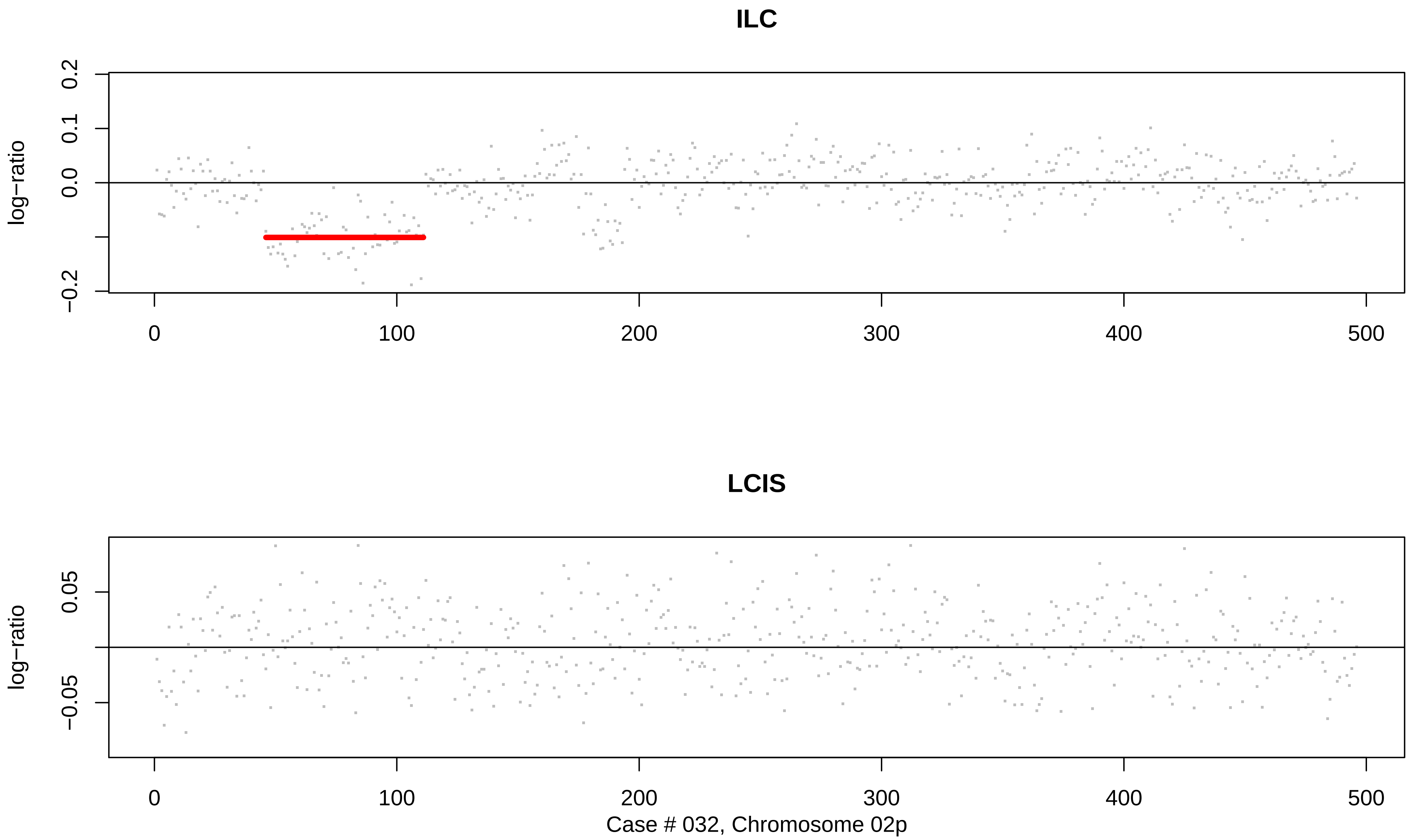

ILC
log−ratio
0.2
0.1
0.0
−0.2
0
100
200
300
400
500
LCIS
log−ratio
0.05
−0.05
0
100
200
300
400
500
Case # 032, Chromosome 02p

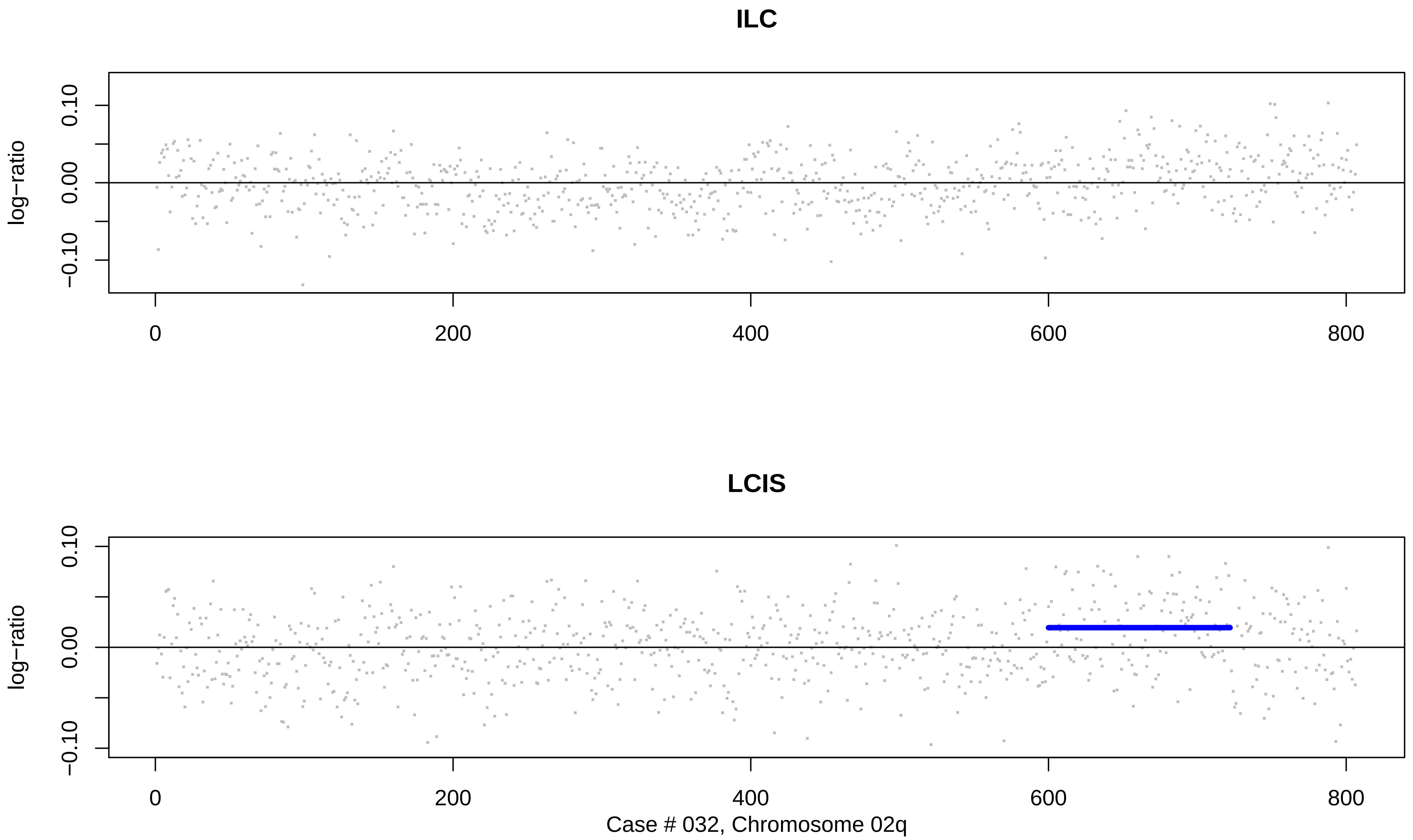
ILC
log−ratio
0.10
0.00
−0.10
0
200
400
600
800
LCIS
log−ratio
0.10
0.00
−0.10
0
200
400
600
800
Case # 032, Chromosome 02q

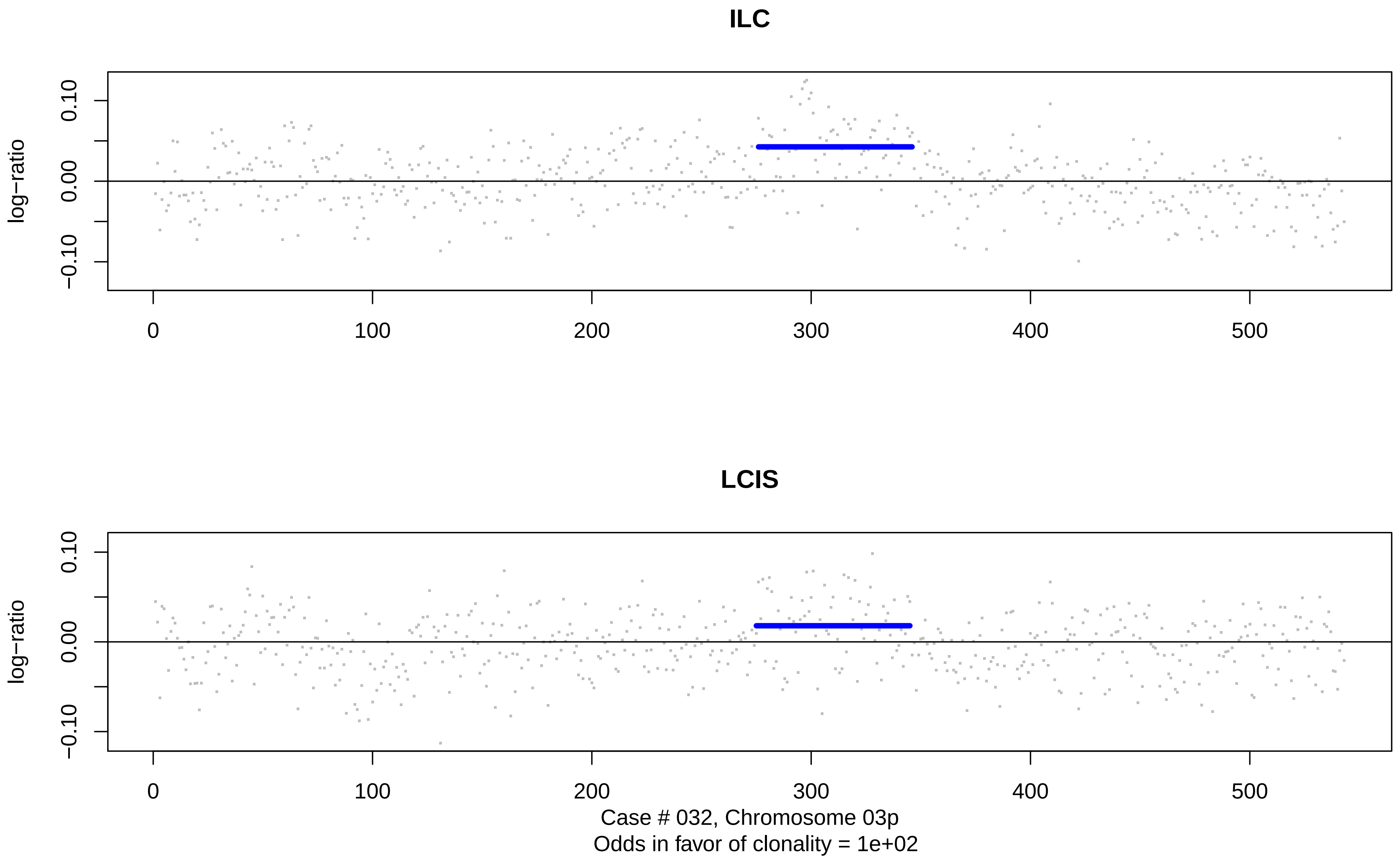
ILC
log−ratio
0.10
0.00
−0.10
0
100
200
300
400
500
LCIS
log−ratio
0.10
0.00
−0.10
0
100
200
300
400
500
Case # 032, Chromosome 03p
Odds in favor of clonality = 1e+02

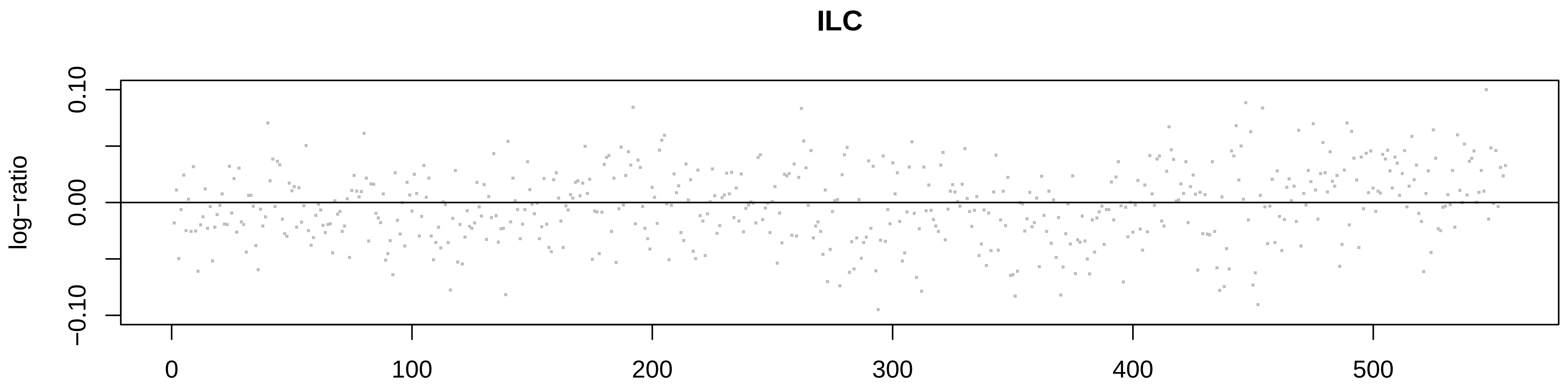

ILC
log−ratio
0.10
0.00
−0.10
0
100
200
300
400
500

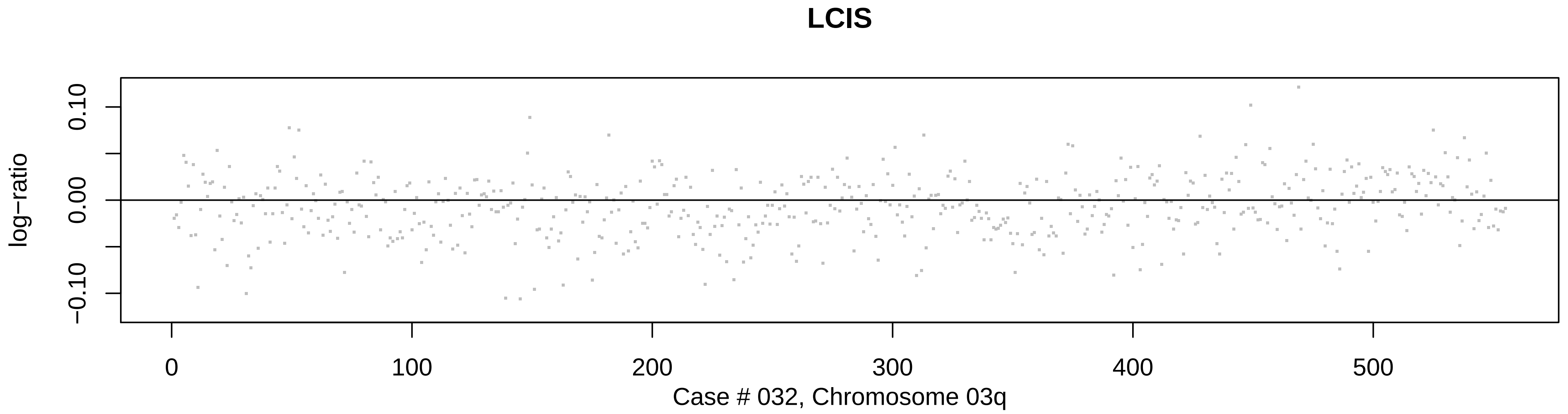

LCIS
log−ratio
0.10
0.00
−0.10
0
100
200
300
400
500
Case # 032, Chromosome 03q

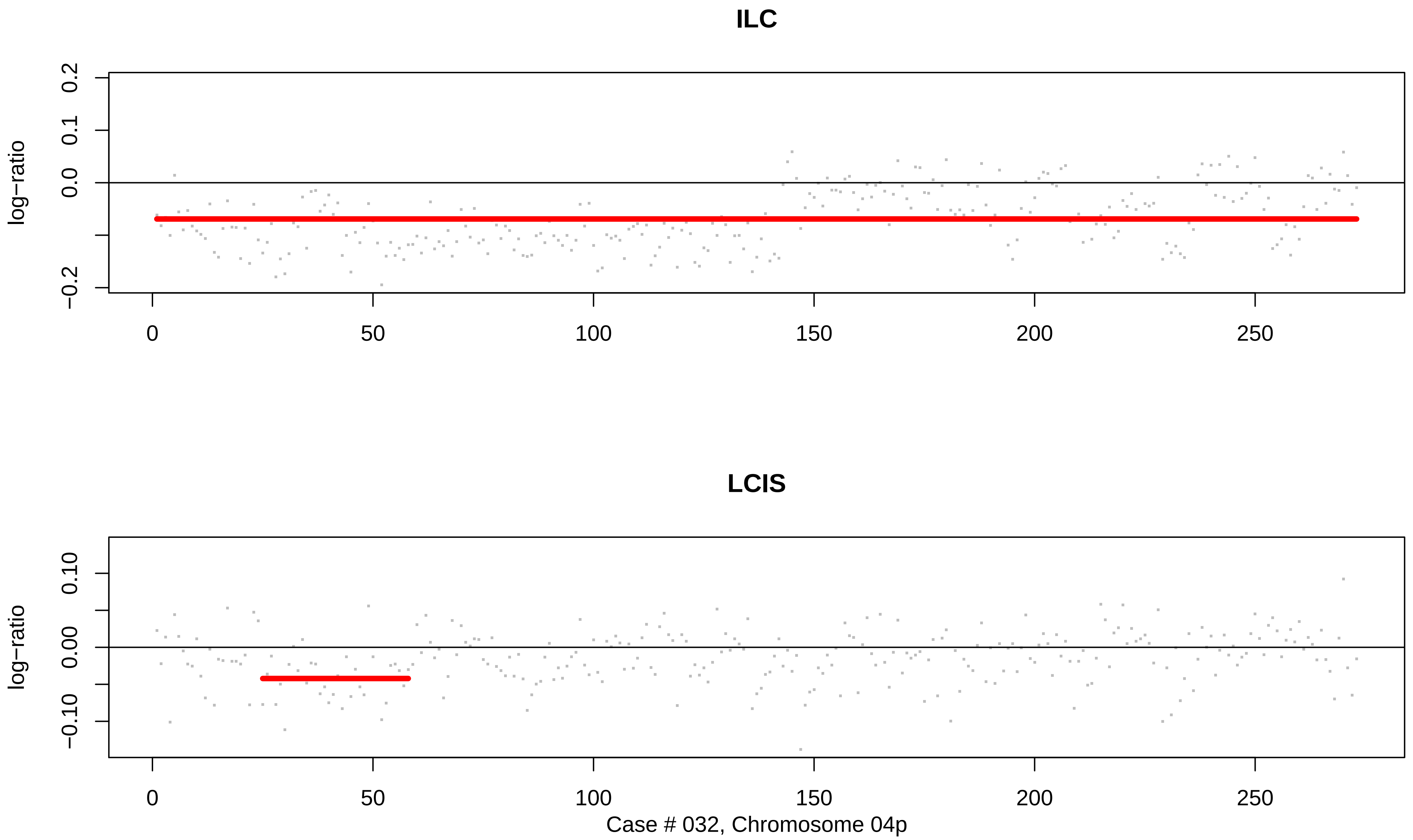

ILC
log−ratio
0.2
0.1
0.0
−0.2
0
50
100
150
200
250
LCIS
log−ratio
0.10
0.00
−0.10
0
50
100
150
200
250
Case # 032, Chromosome 04p

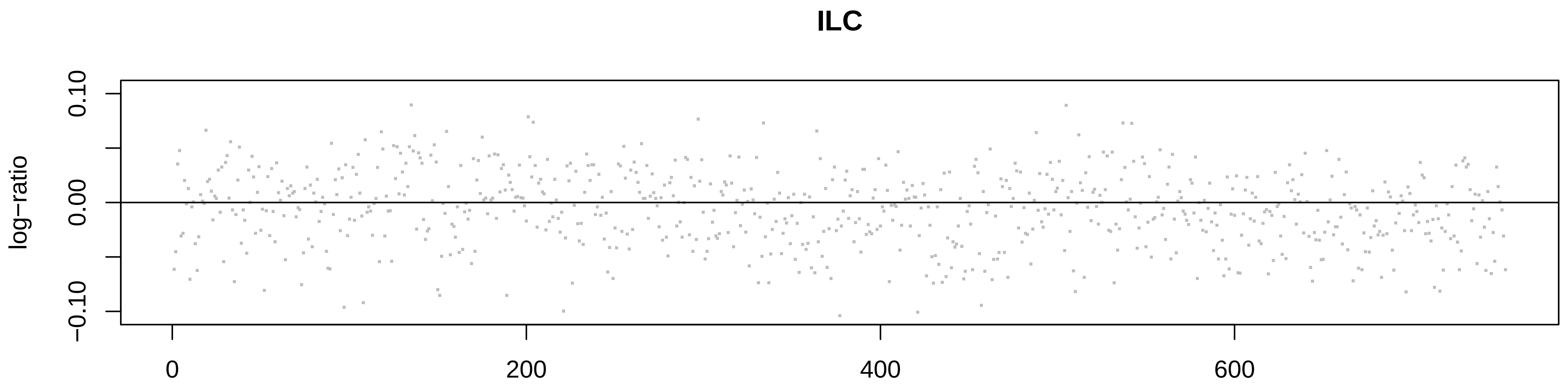
ILC
log−ratio
0.10
0.00
−0.10
0
200
400
600

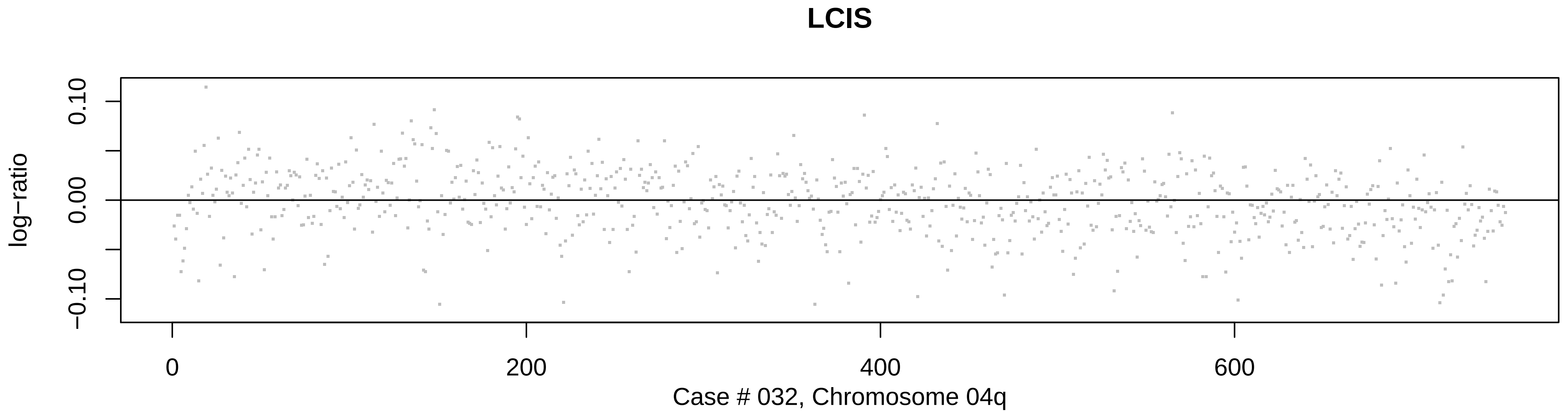
LCIS
log−ratio
0.10
0.00
−0.10
0
200
400
600
Case # 032, Chromosome 04q

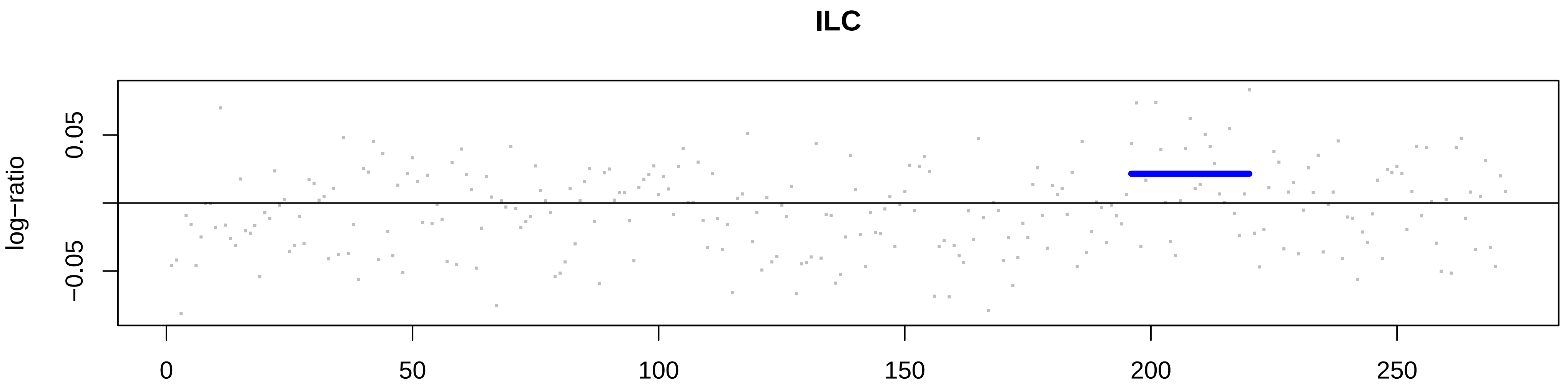
ILC
log−ratio
0.05
−0.05
0
50
100
150
200
250

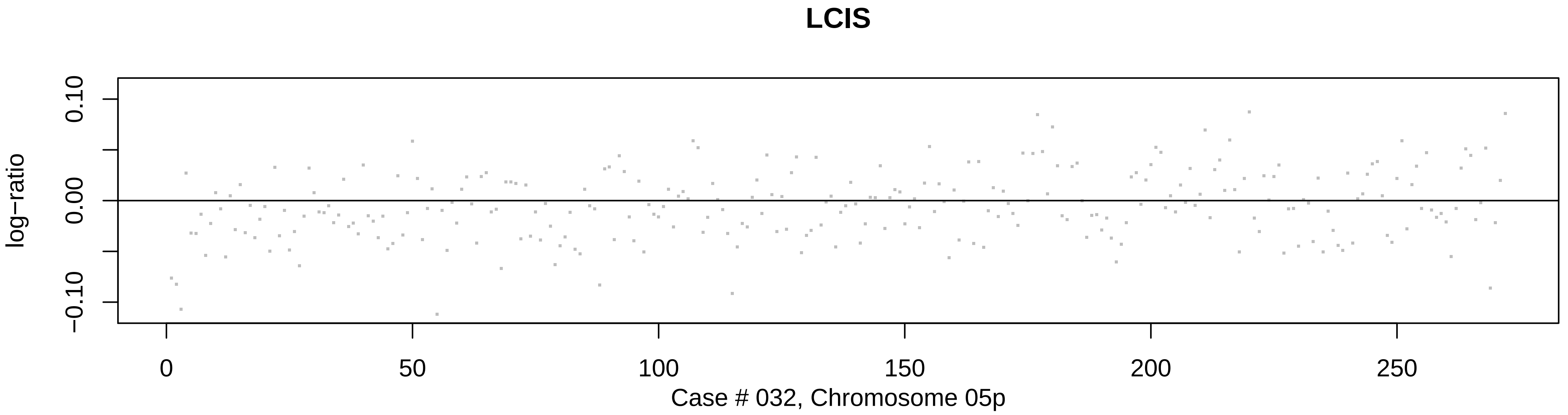
LCIS
log−ratio
0.10
0.00
−0.10
0
50
100
150
200
250
Case # 032, Chromosome 05p

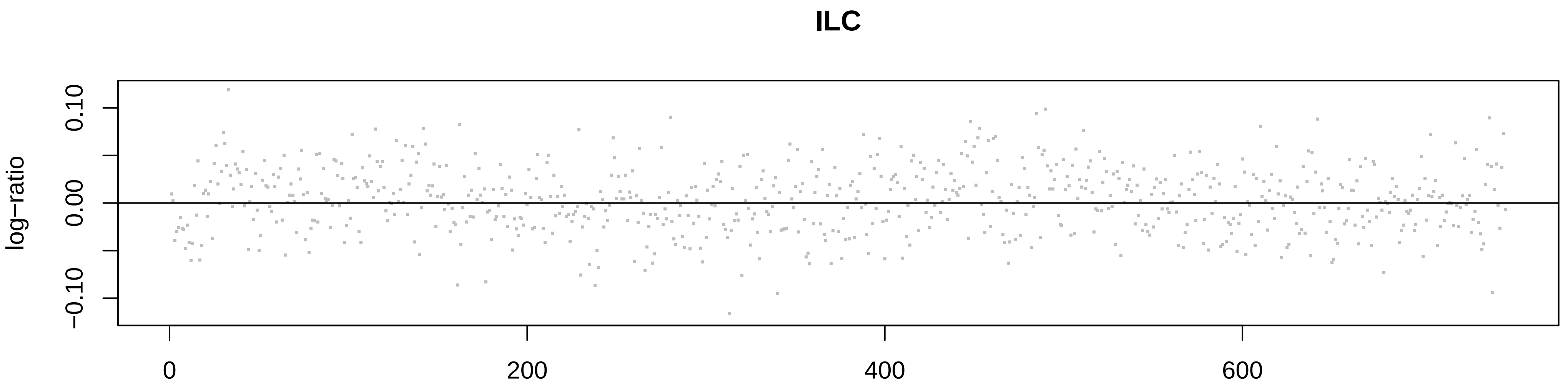
ILC
log−ratio
0.10
0.00
−0.10
0
200
400
600

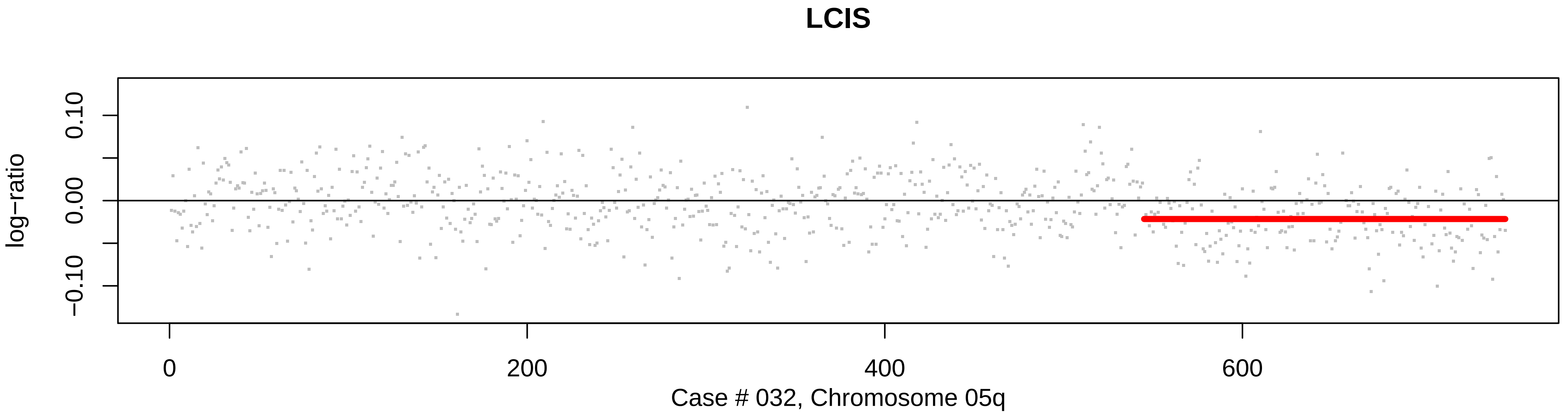
LCIS
log−ratio
0.10
0.00
−0.10
0
200
400
600
Case # 032, Chromosome 05q

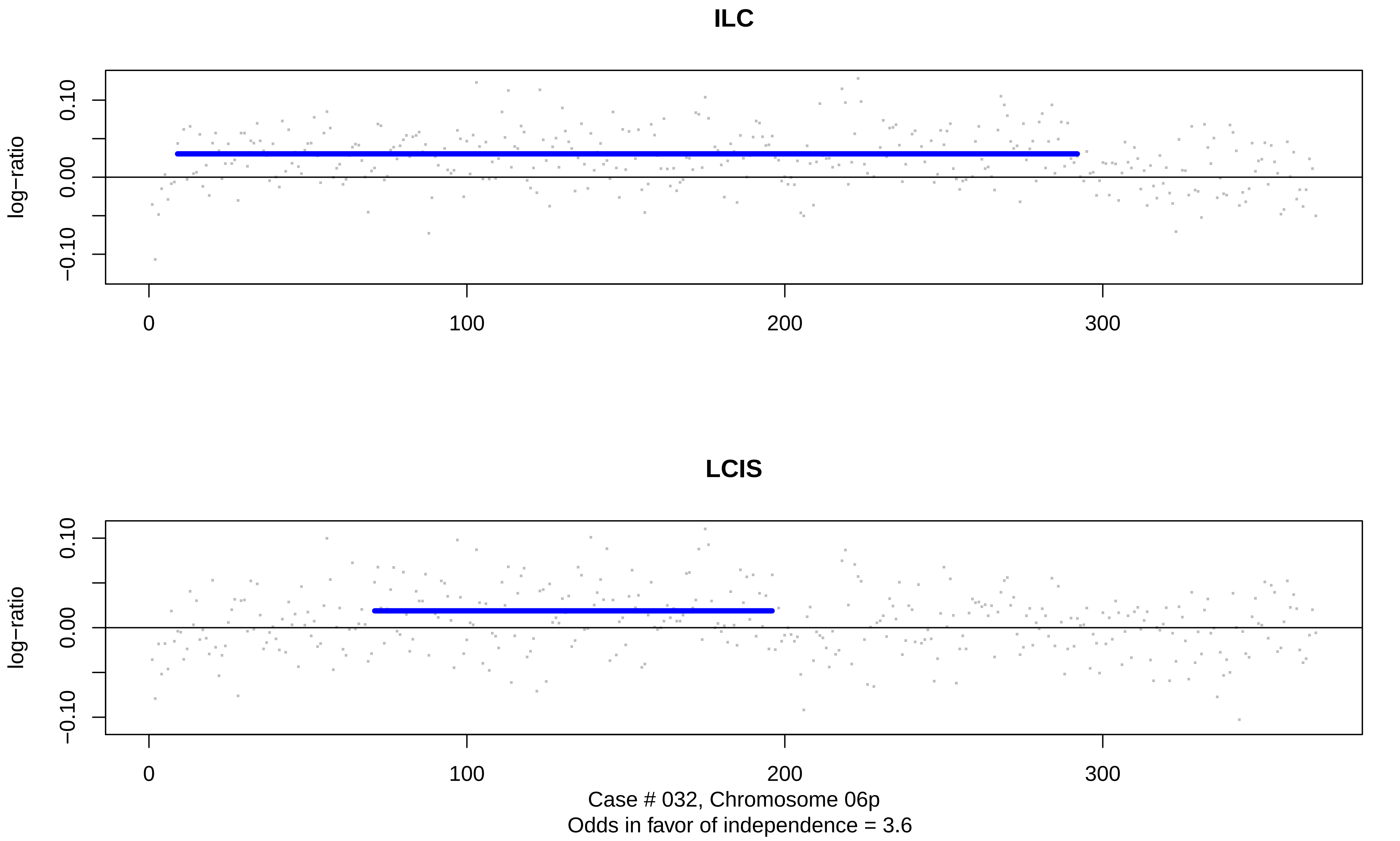
ILC
log−ratio
0.10
0.00
−0.10
0
100
200
300
LCIS
log−ratio
0.10
0.00
−0.10
0
100
200
300
Case # 032, Chromosome 06p
Odds in favor of independence = 3.6

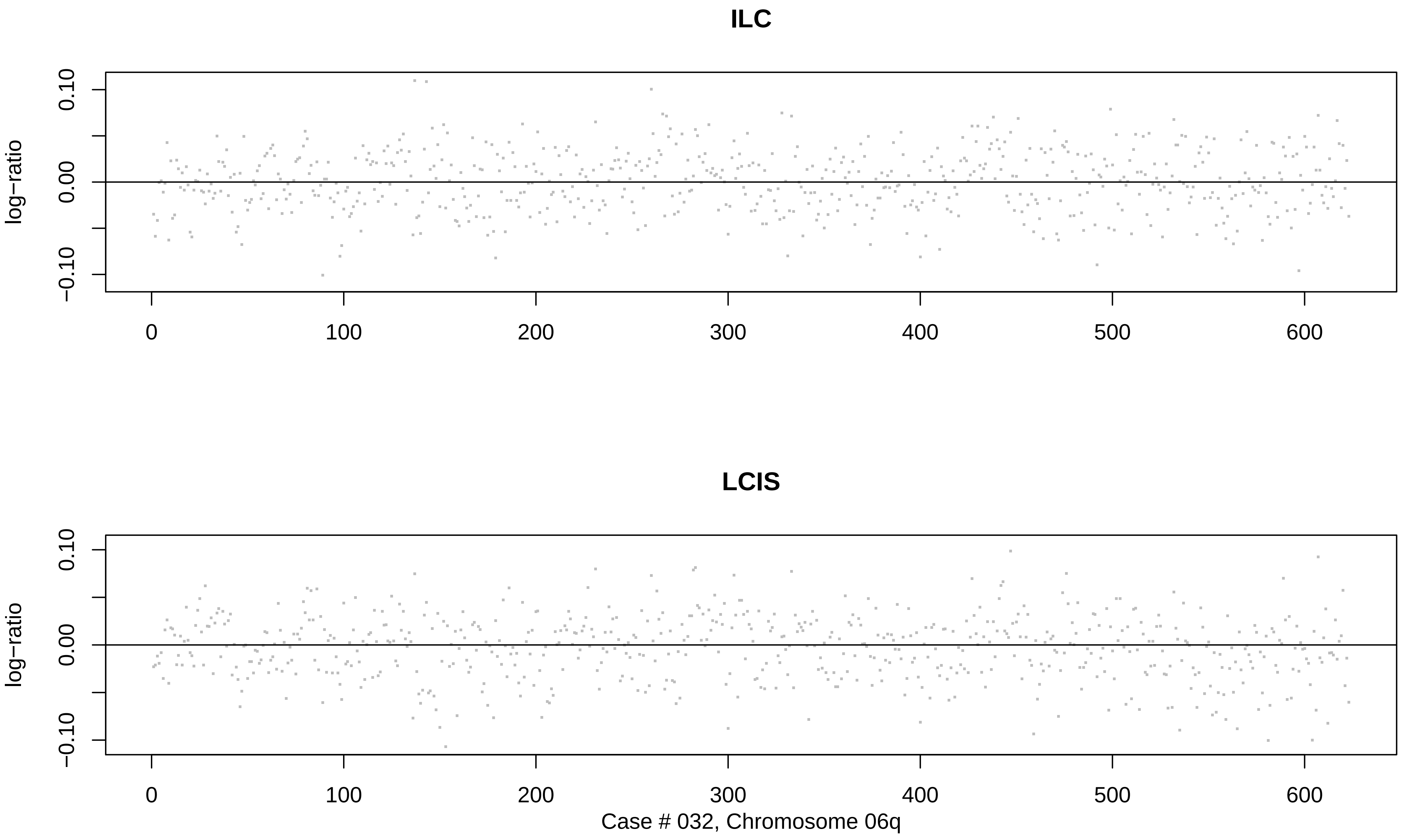
ILC
log−ratio
0.10
0.00
−0.10
0
100
200
300
400
500
600
LCIS
log−ratio
0.10
0.00
−0.10
0
100
200
300
400
500
600
Case # 032, Chromosome 06q

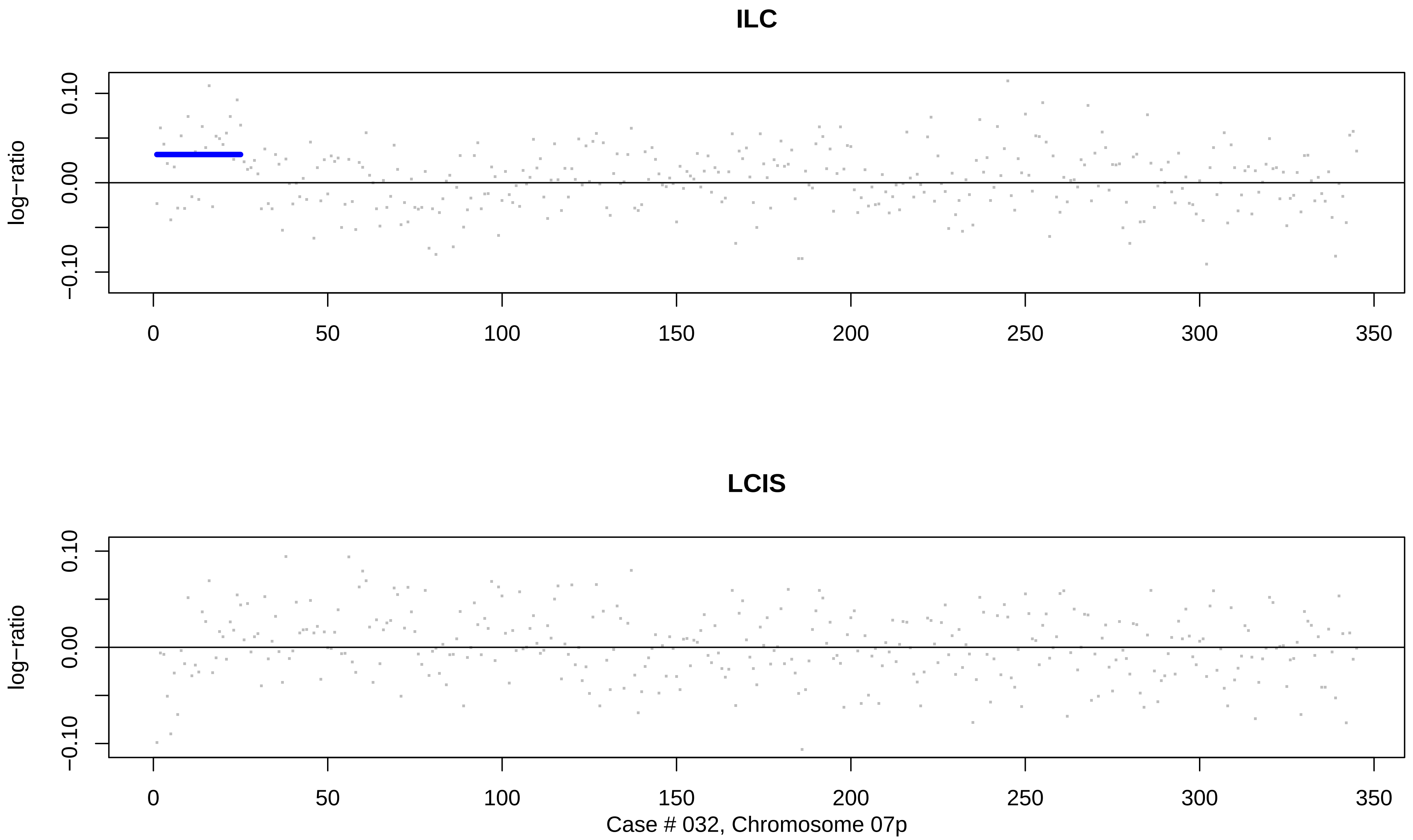

ILC
log−ratio
0.10
0.00
−0.10
0
50
100
150
200
250
300
350
LCIS
log−ratio
0.10
0.00
−0.10
0
50
100
150
200
250
300
350
Case # 032, Chromosome 07p

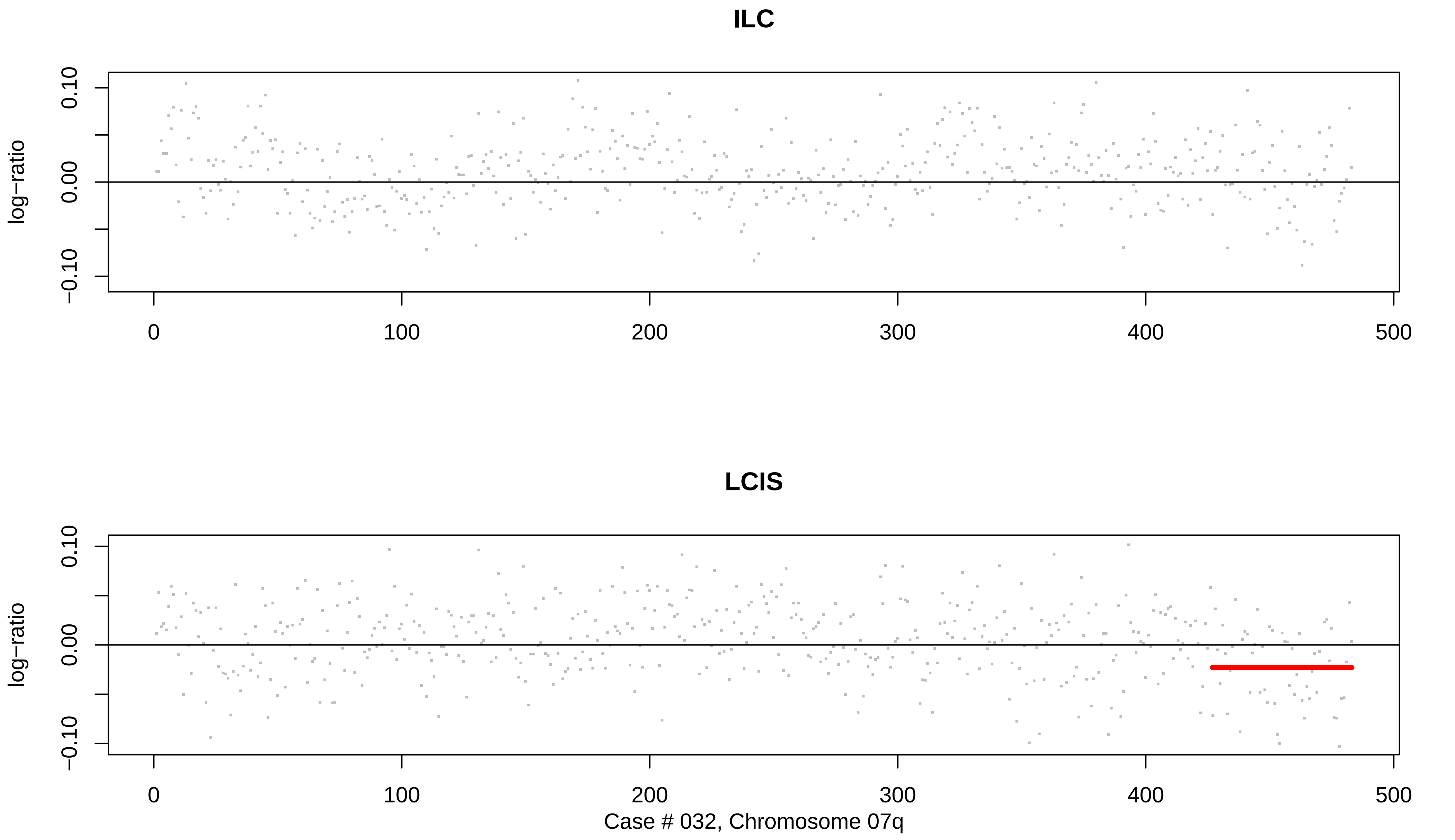
ILC
log−ratio
0.10
0.00
−0.10
0
100
200
300
400
500
LCIS
log−ratio
0.10
0.00
−0.10
0
100
200
300
400
500
Case # 032, Chromosome 07q

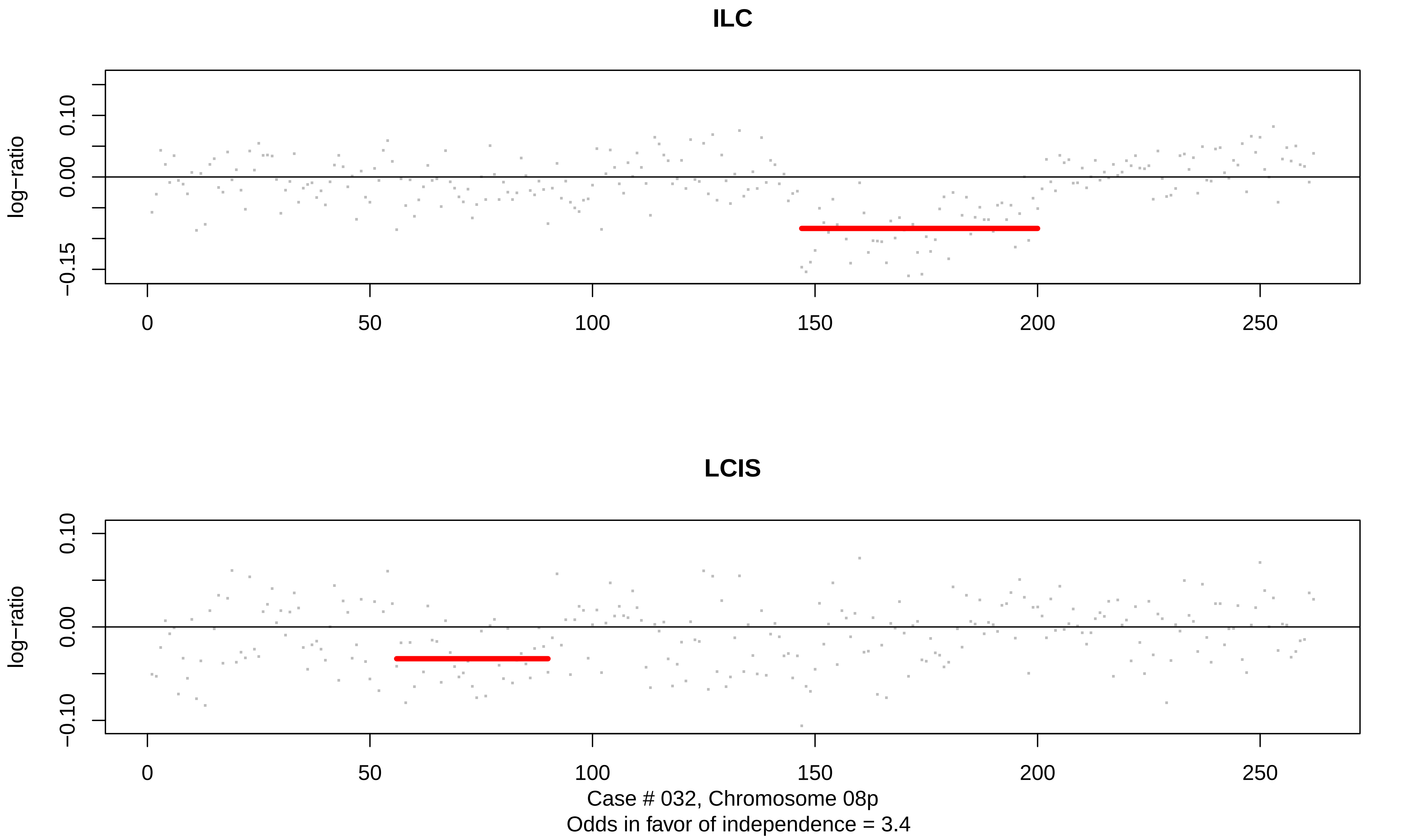
ILC
LCIS
log−ratio
0.10
0.00
−0.15
−0.10
0
50
100
150
200
250
Case # 032, Chromosome 08p
Odds in favor of independence = 3.4

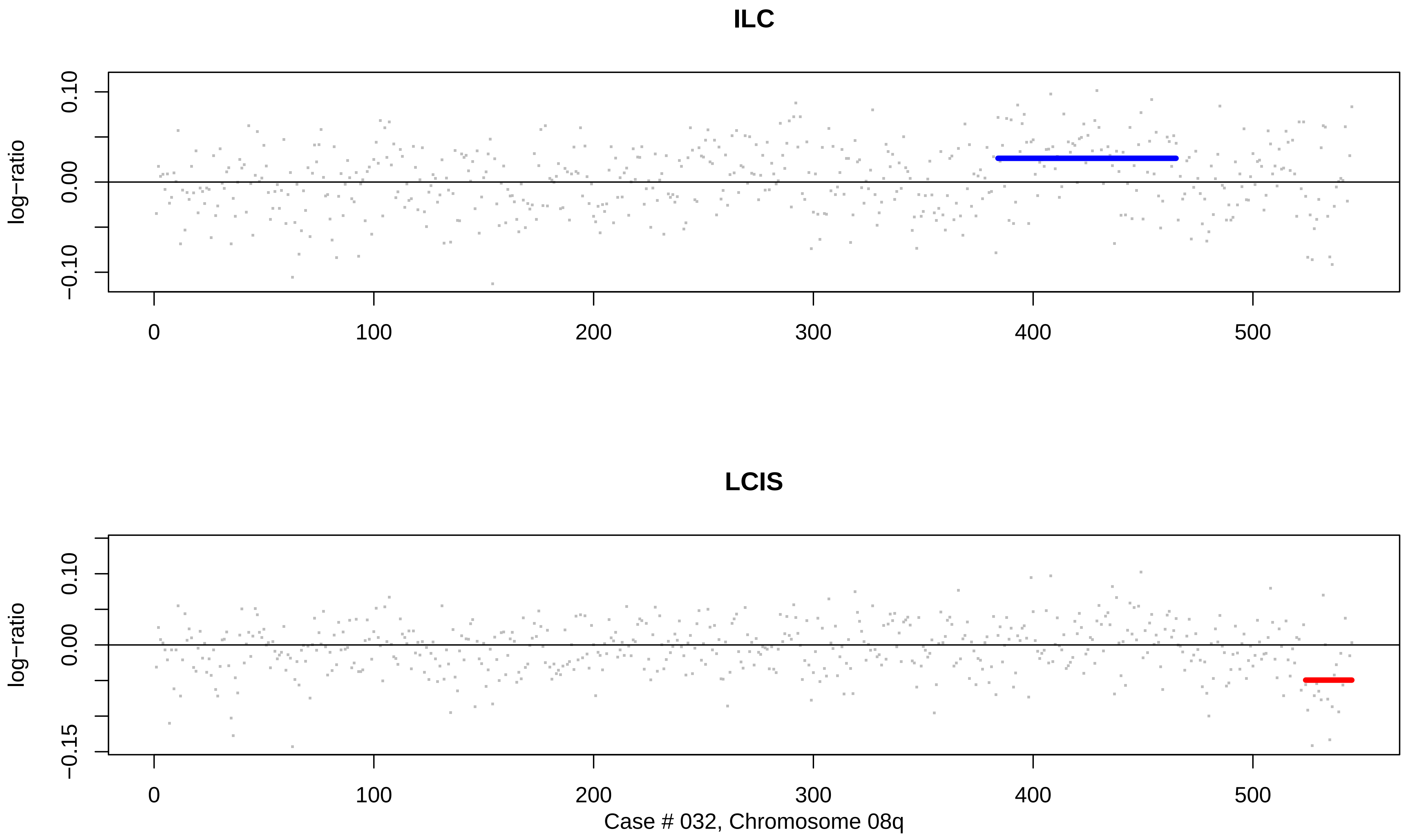

ILC
log−ratio
0.10
0.00
−0.10
0
100
200
300
400
500
LCIS
log−ratio
0.10
0.00
−0.15
0
100
200
300
400
500
Case # 032, Chromosome 08q

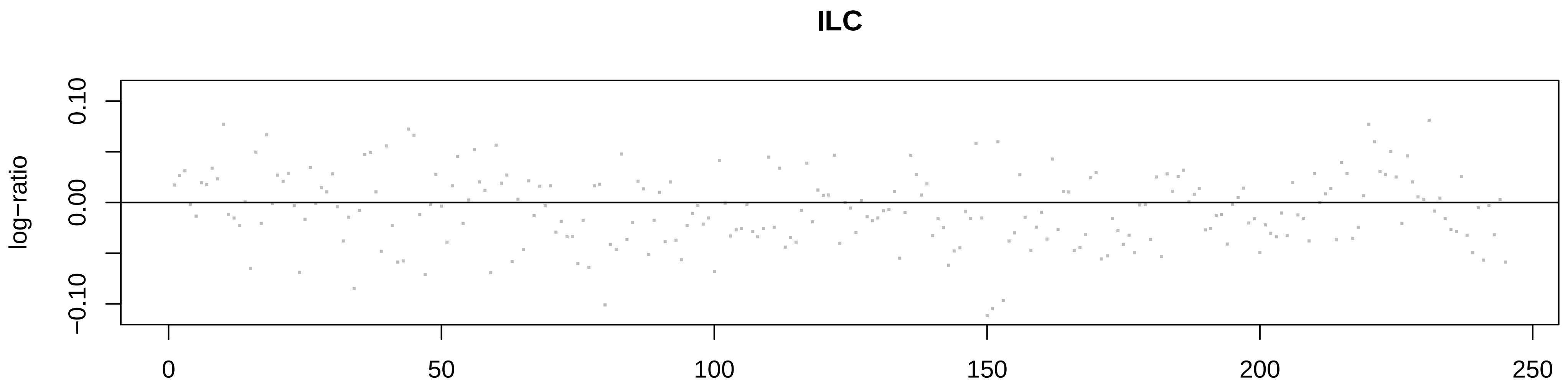
ILC
log−ratio
0.10
0.00
−0.10
0
50
100
150
200
250

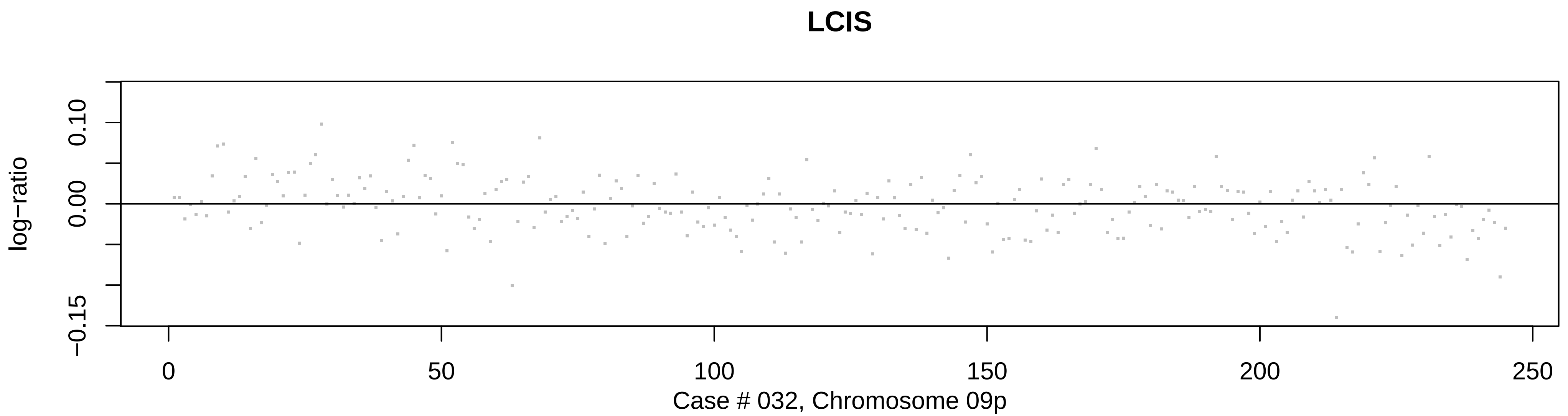
LCIS
log−ratio
0.10
0.00
−0.15
0
50
100
150
200
250
Case # 032, Chromosome 09p

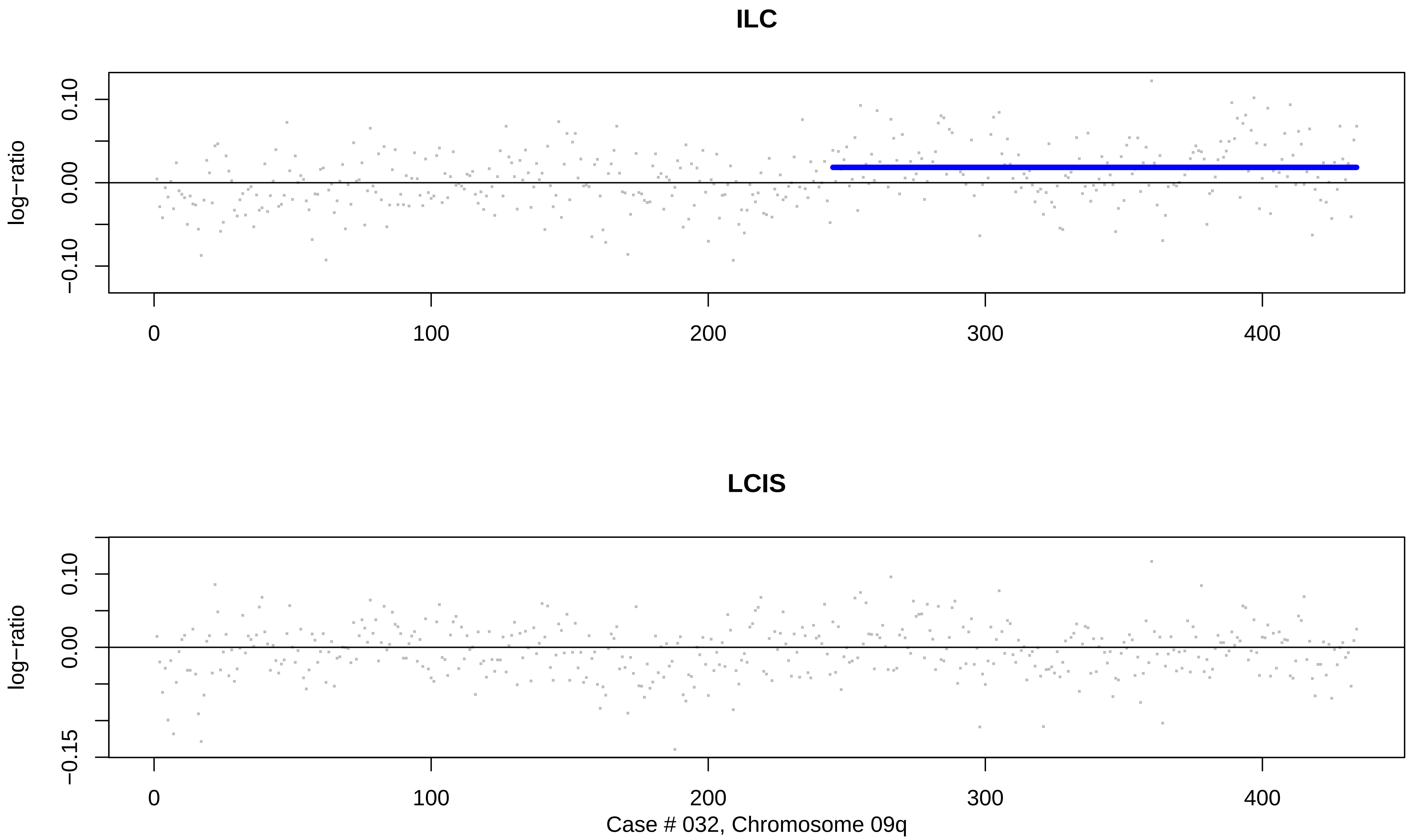

ILC
log−ratio
0.10
0.00
−0.10
0
100
200
300
400
LCIS
log−ratio
0.10
0.00
−0.15
0
100
200
300
400
Case # 032, Chromosome 09q

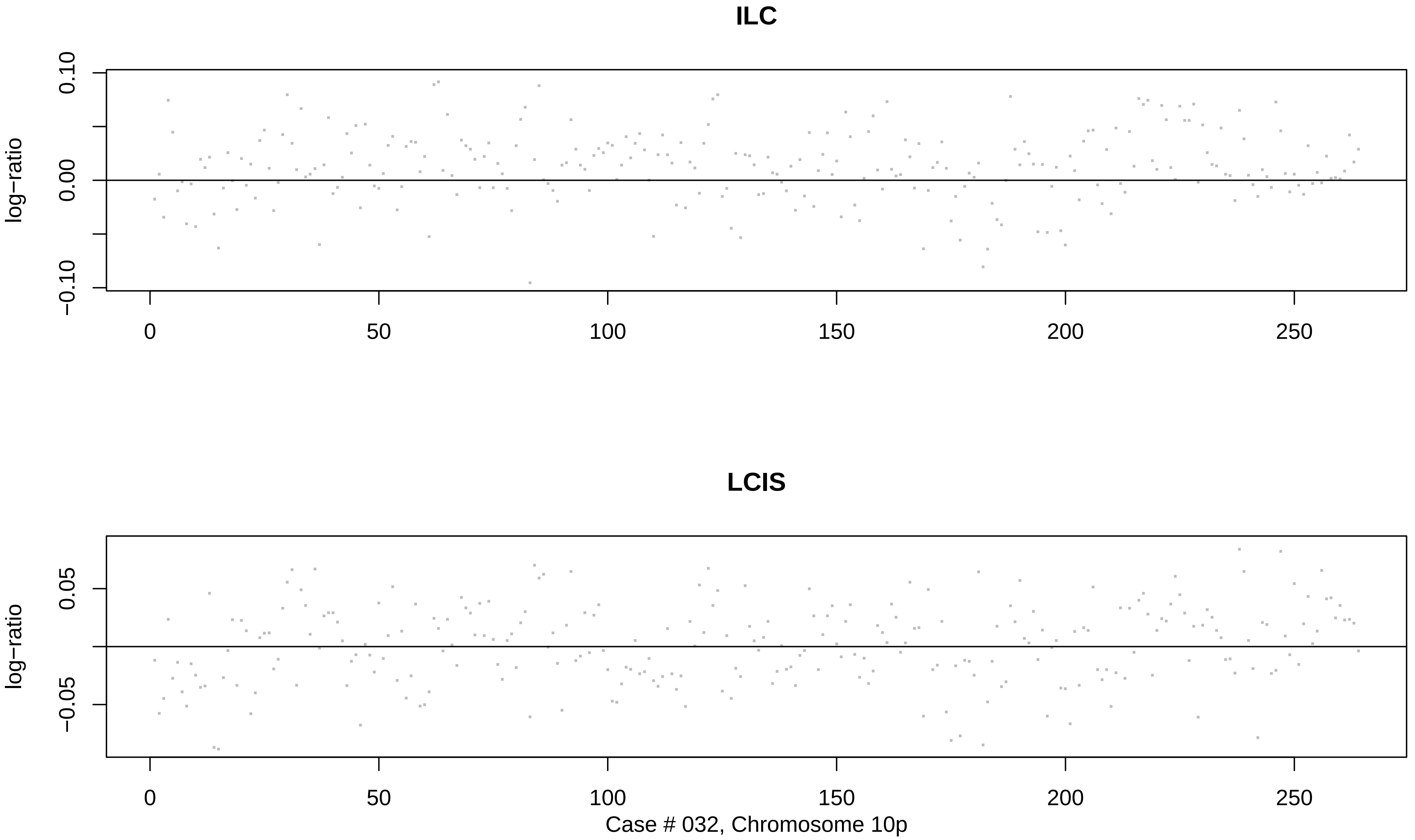
ILC
log−ratio
0.10
0.00
−0.10
0
50
100
150
200
250
LCIS
log−ratio
0.05
−0.05
0
50
100
150
200
250
Case # 032, Chromosome 10p

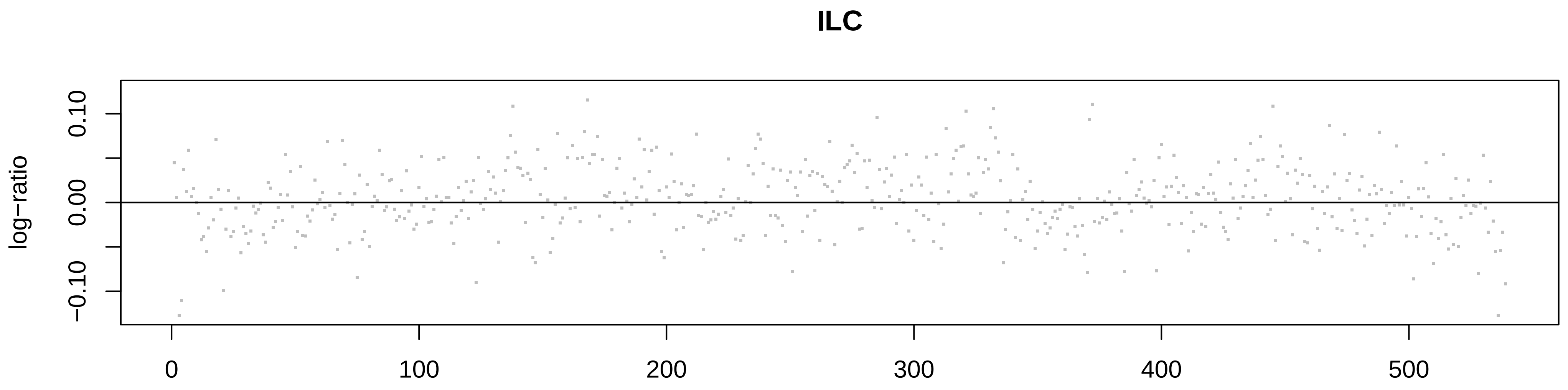
ILC
log−ratio
0.10
0.00
−0.10
0
100
200
300
400
500

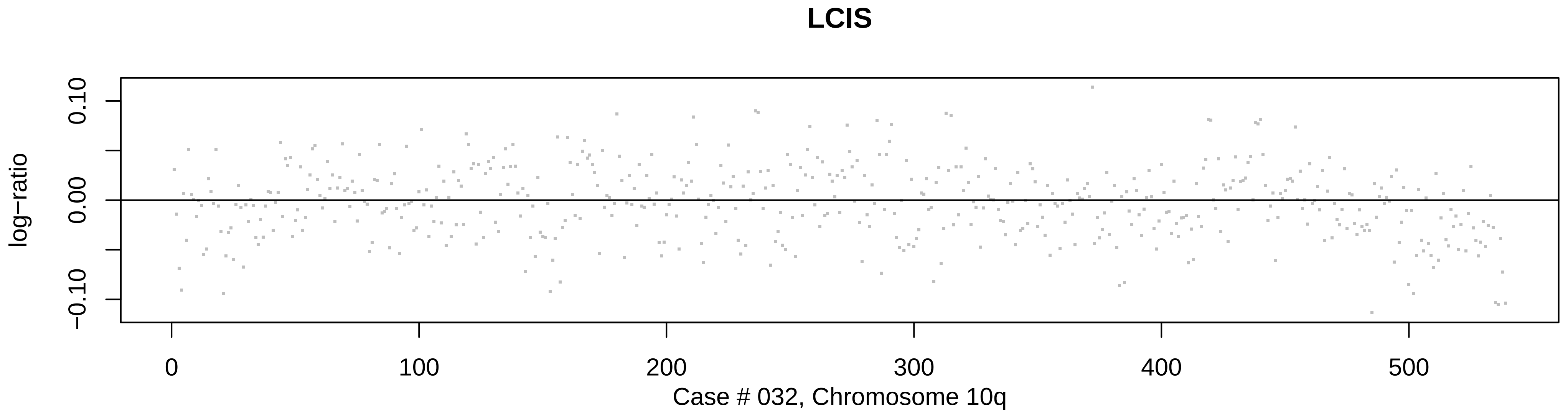
LCIS
log−ratio
0.10
0.00
−0.10
0
100
200
300
400
500
Case # 032, Chromosome 10q

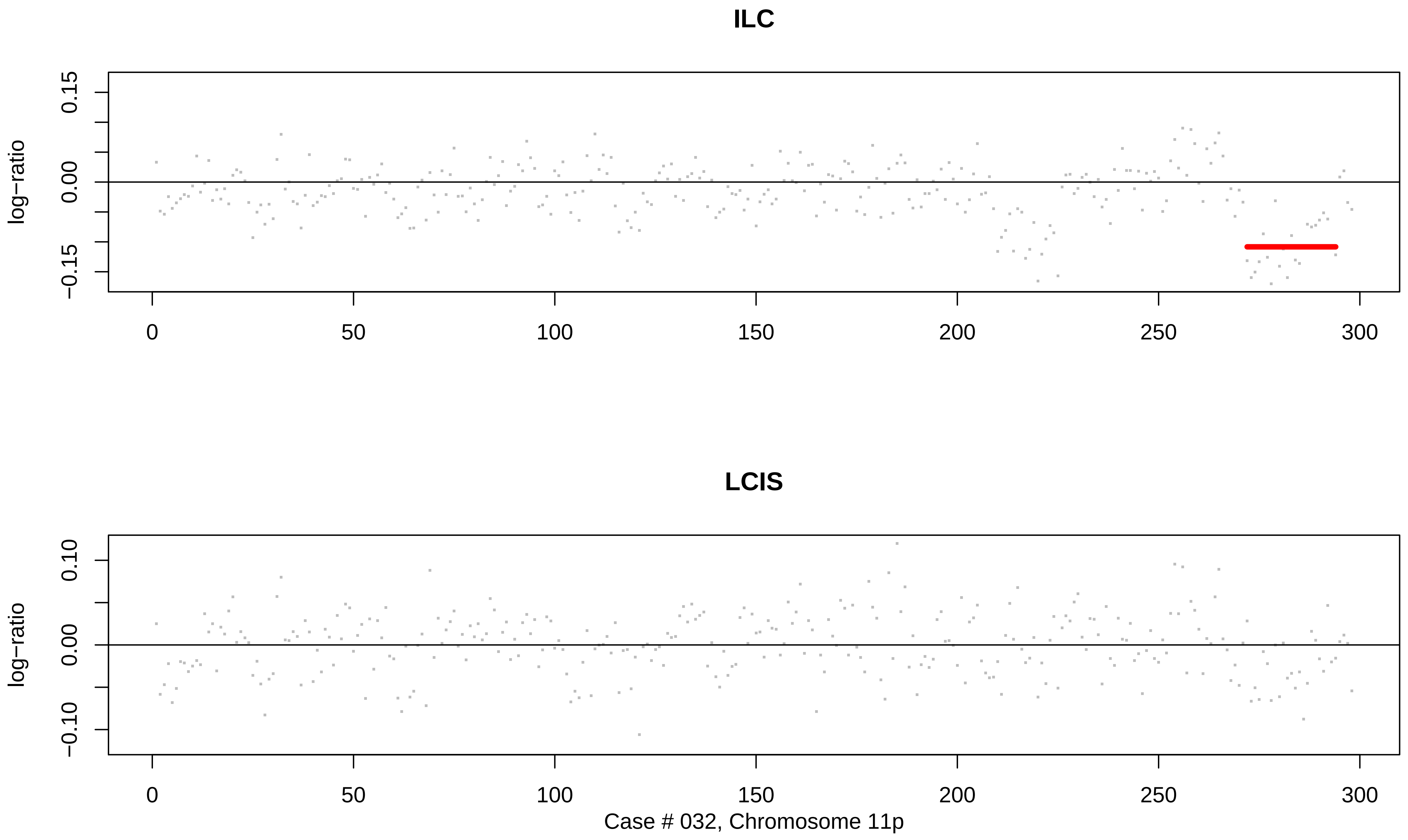

ILC
log−ratio
0.15
0.00
−0.15
0
50
100
150
200
250
300
LCIS
log−ratio
0.10
0.00
−0.10
0
50
100
150
200
250
300
Case # 032, Chromosome 11p

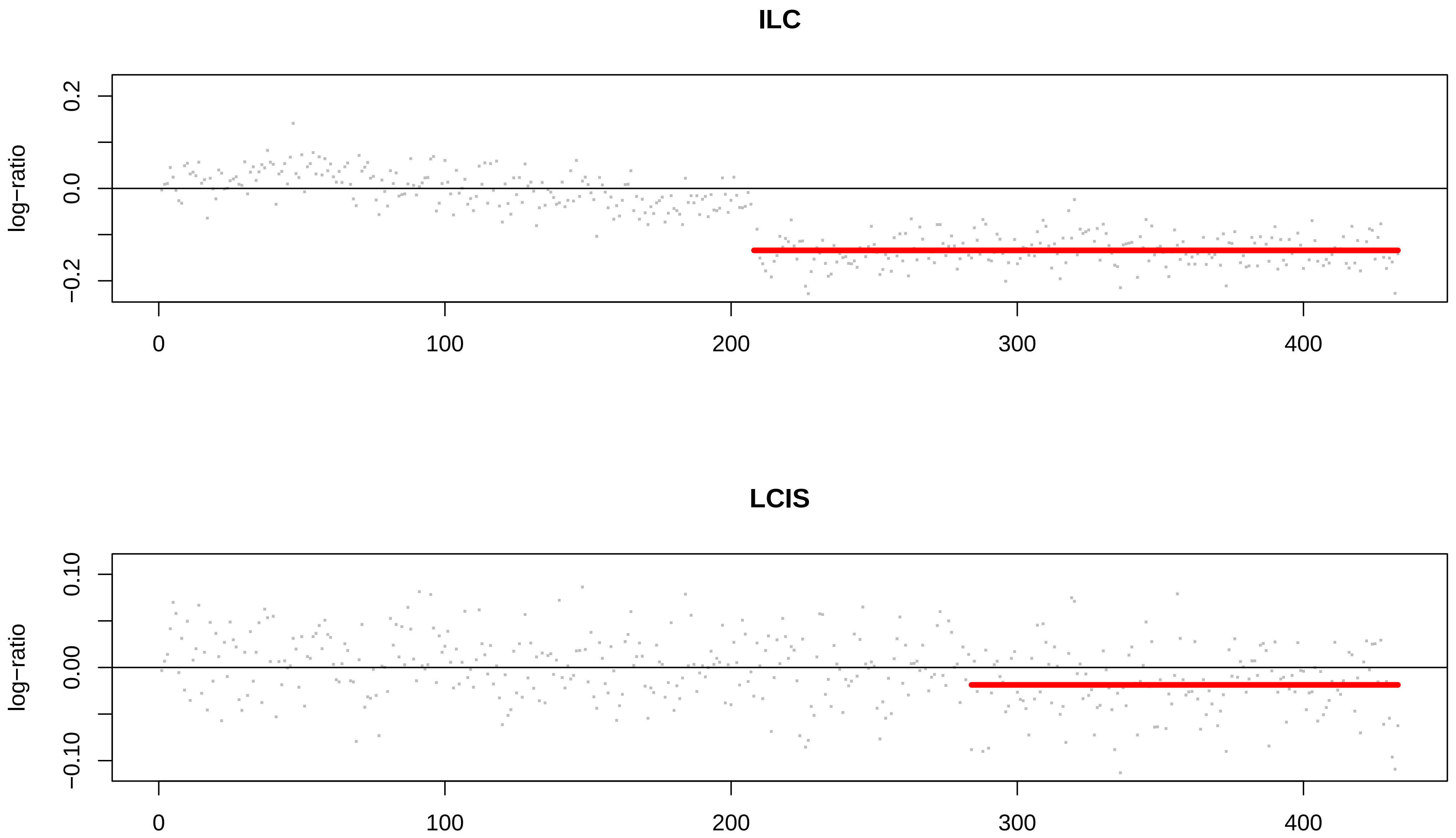

Case # 032, Chromosome 11q

Odds in favor of independence = 2.9

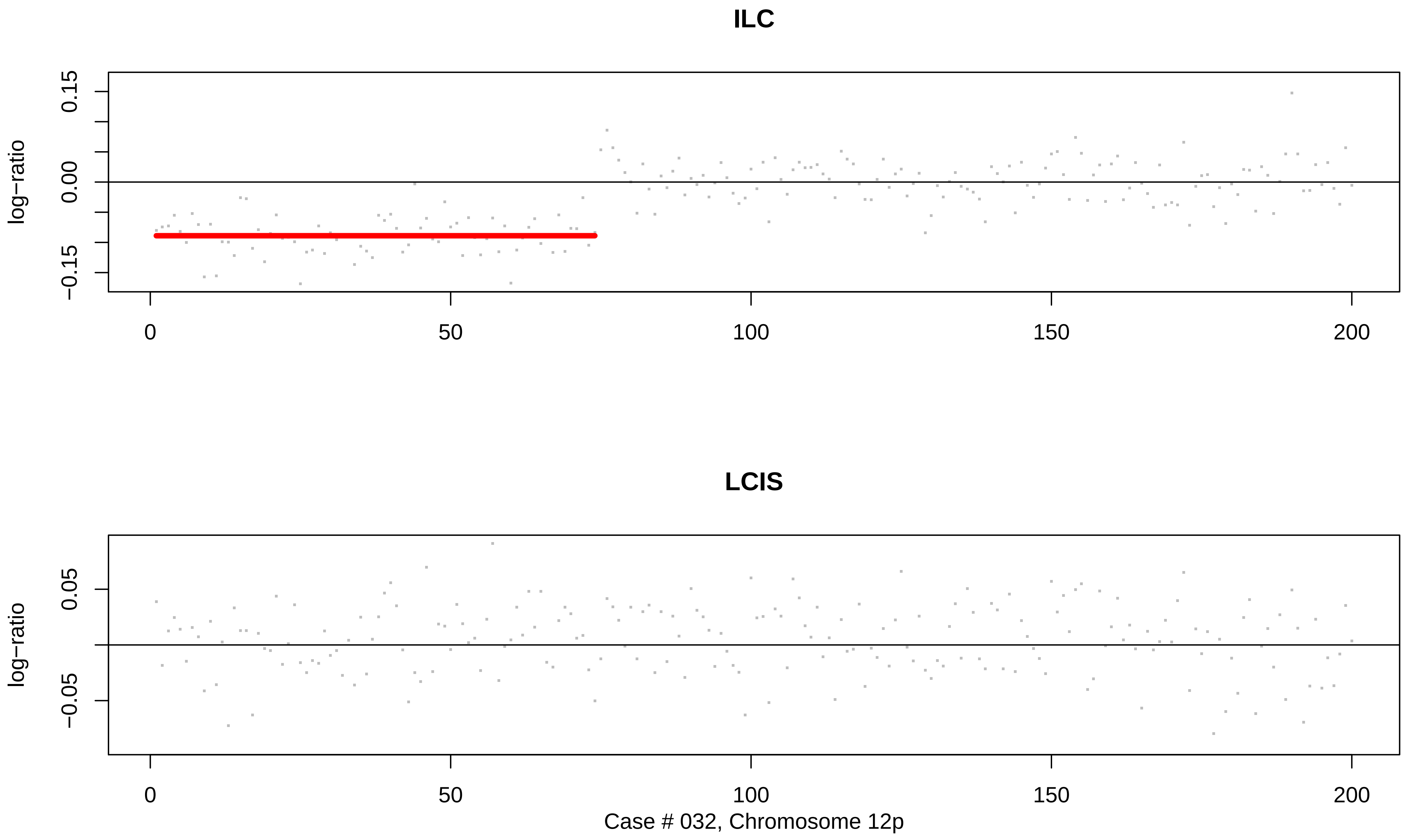
ILC
log−ratio
0.15
0.00
−0.15
0
50
100
150
200
LCIS
log−ratio
0.05
−0.05
0
50
100
150
200
Case # 032, Chromosome 12p

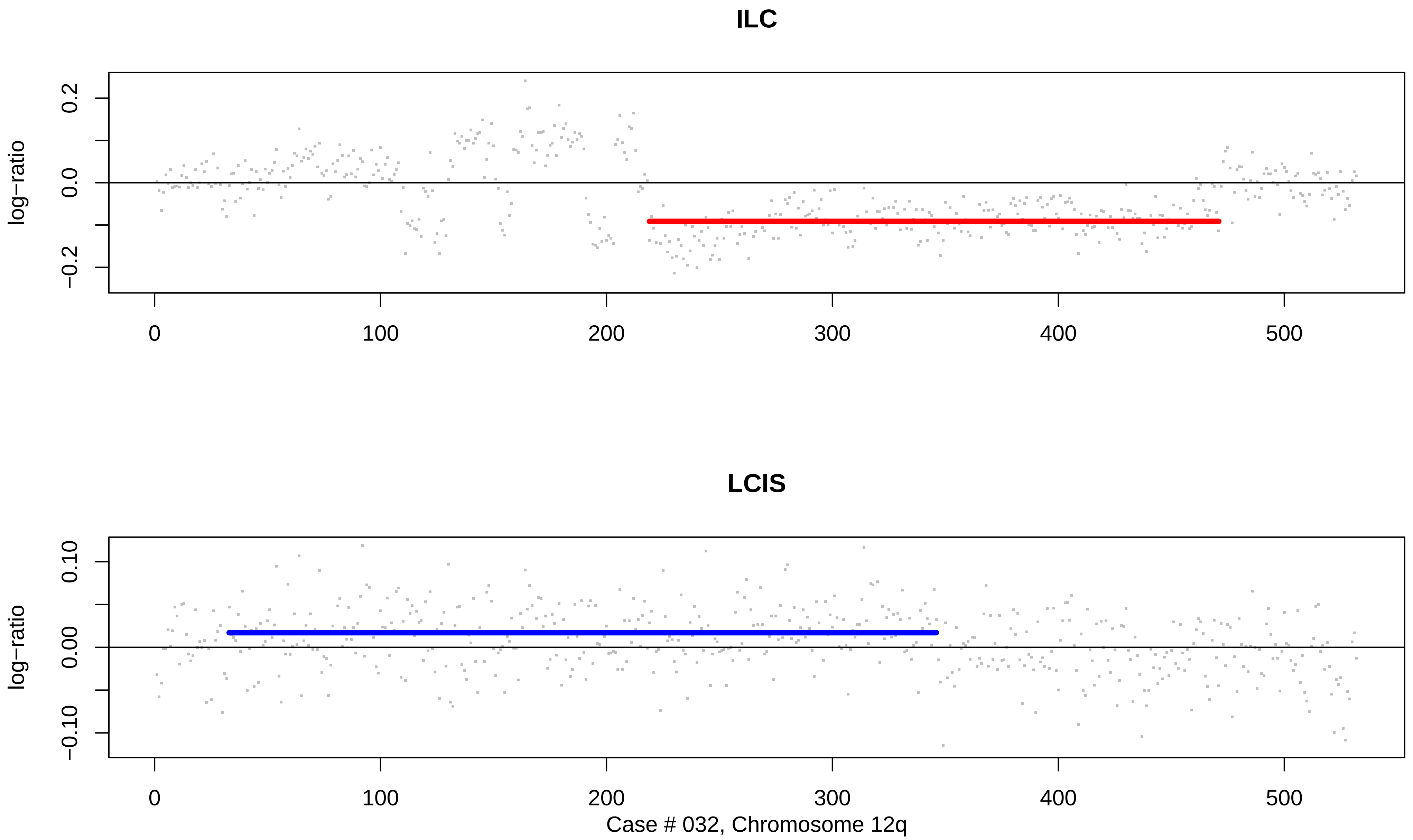

ILC
log−ratio
0.2
0.0
−0.2
0
100
200
300
400
500
LCIS
log−ratio
0.10
0.00
−0.10
0
100
200
300
400
500
Case # 032, Chromosome 12q

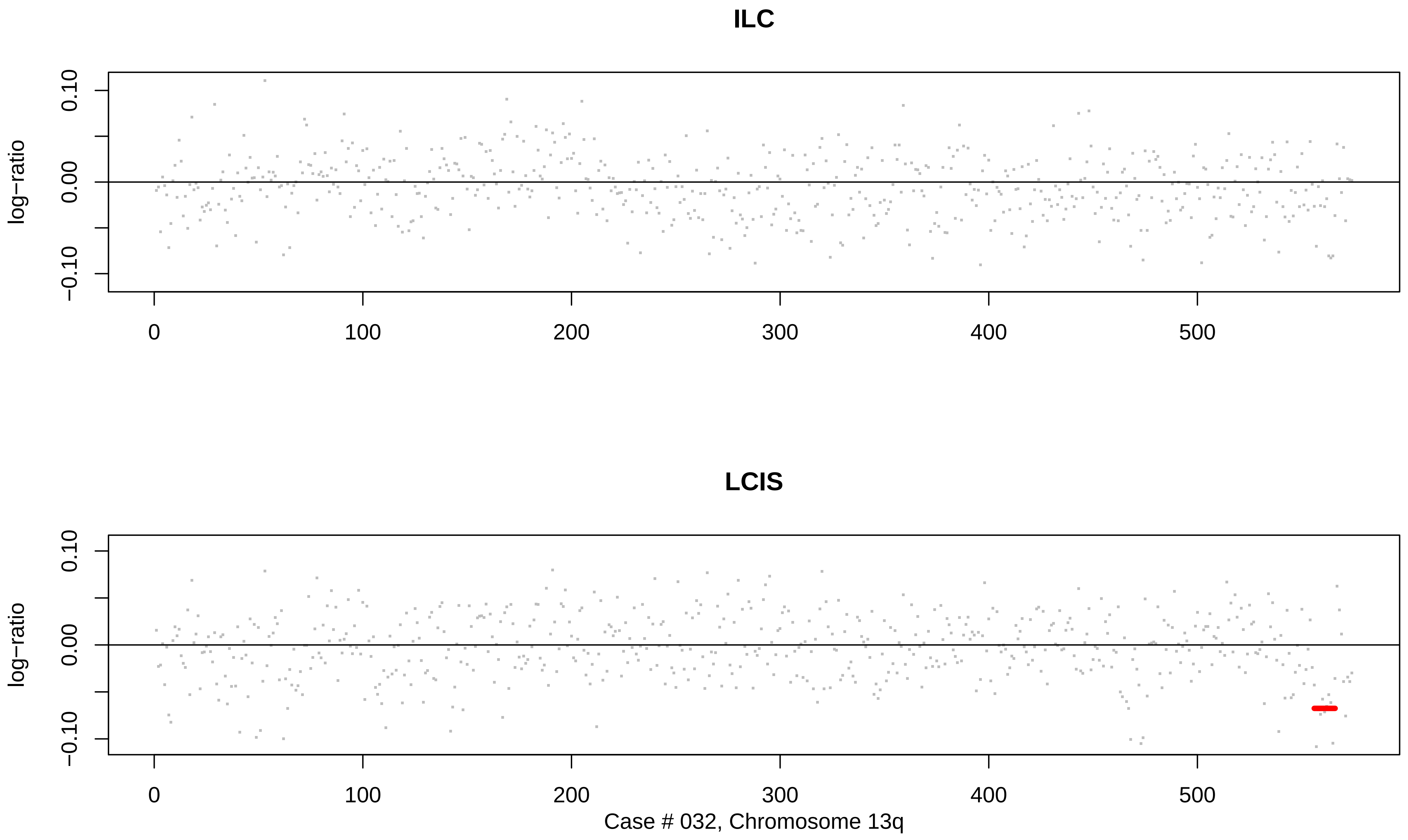
ILC
log−ratio
0.10
0.00
−0.10
0
100
200
300
400
500
LCIS
log−ratio
0.10
0.00
−0.10
0
100
200
300
400
500
Case # 032, Chromosome 13q

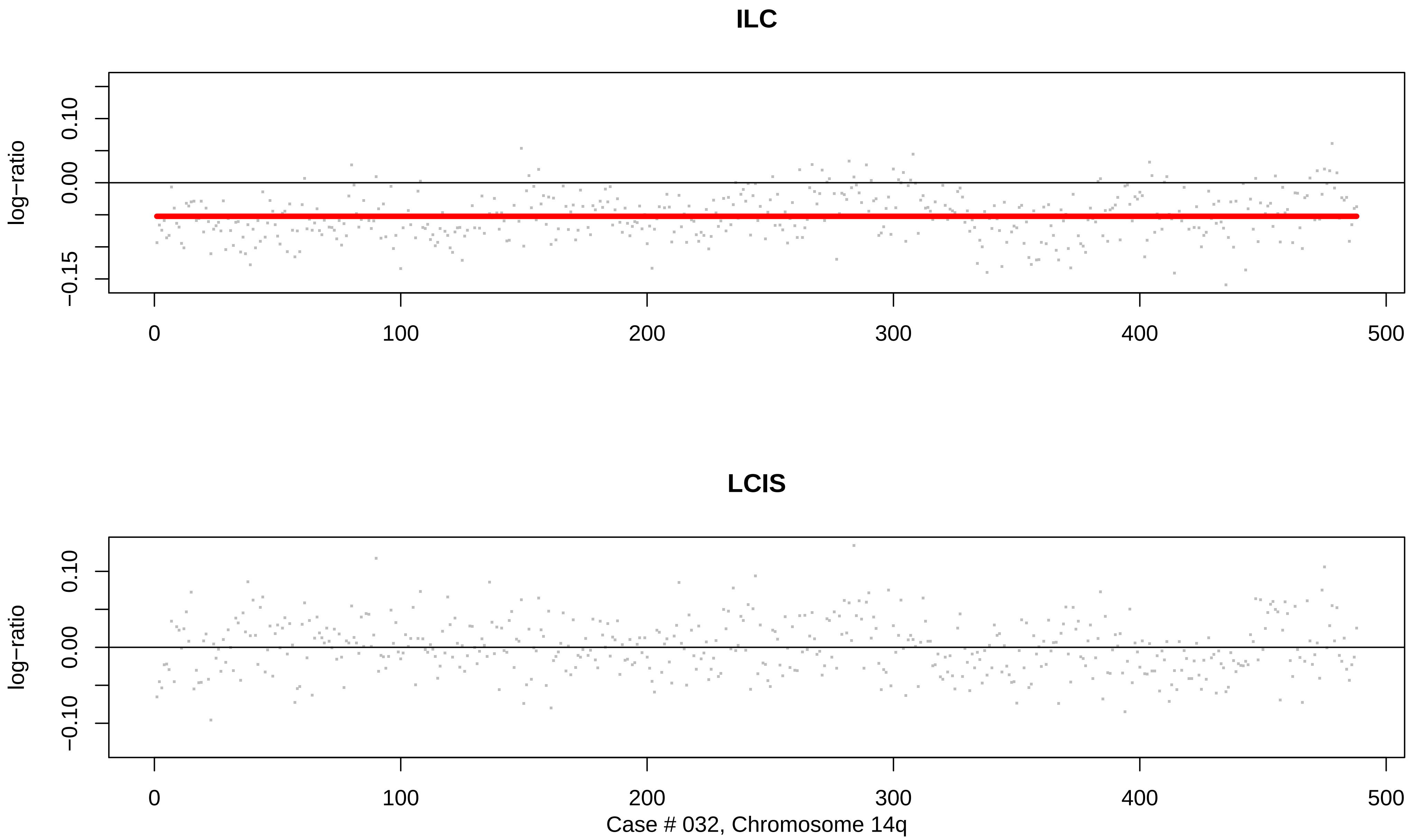
ILC
log−ratio
−0.15
0.00
0.10
0
100
200
300
400
500
LCIS
log−ratio
−0.10
0.00
0.10
0
100
200
300
400
500
Case # 032, Chromosome 14q

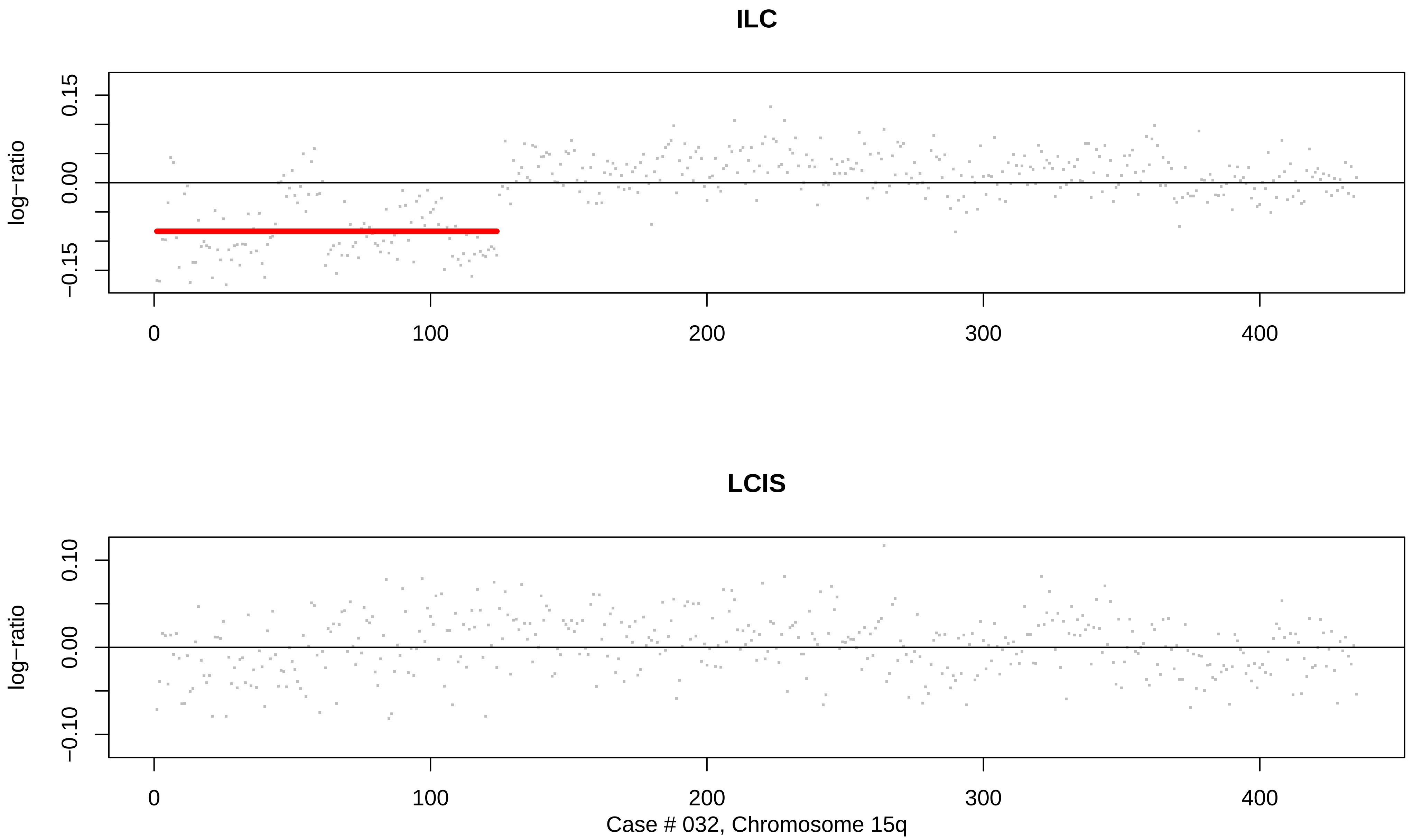

ILC
log−ratio
0.15
0.00
−0.15
0
100
200
300
400
LCIS
log−ratio
0.10
0.00
−0.10
0
100
200
300
400
Case # 032, Chromosome 15q

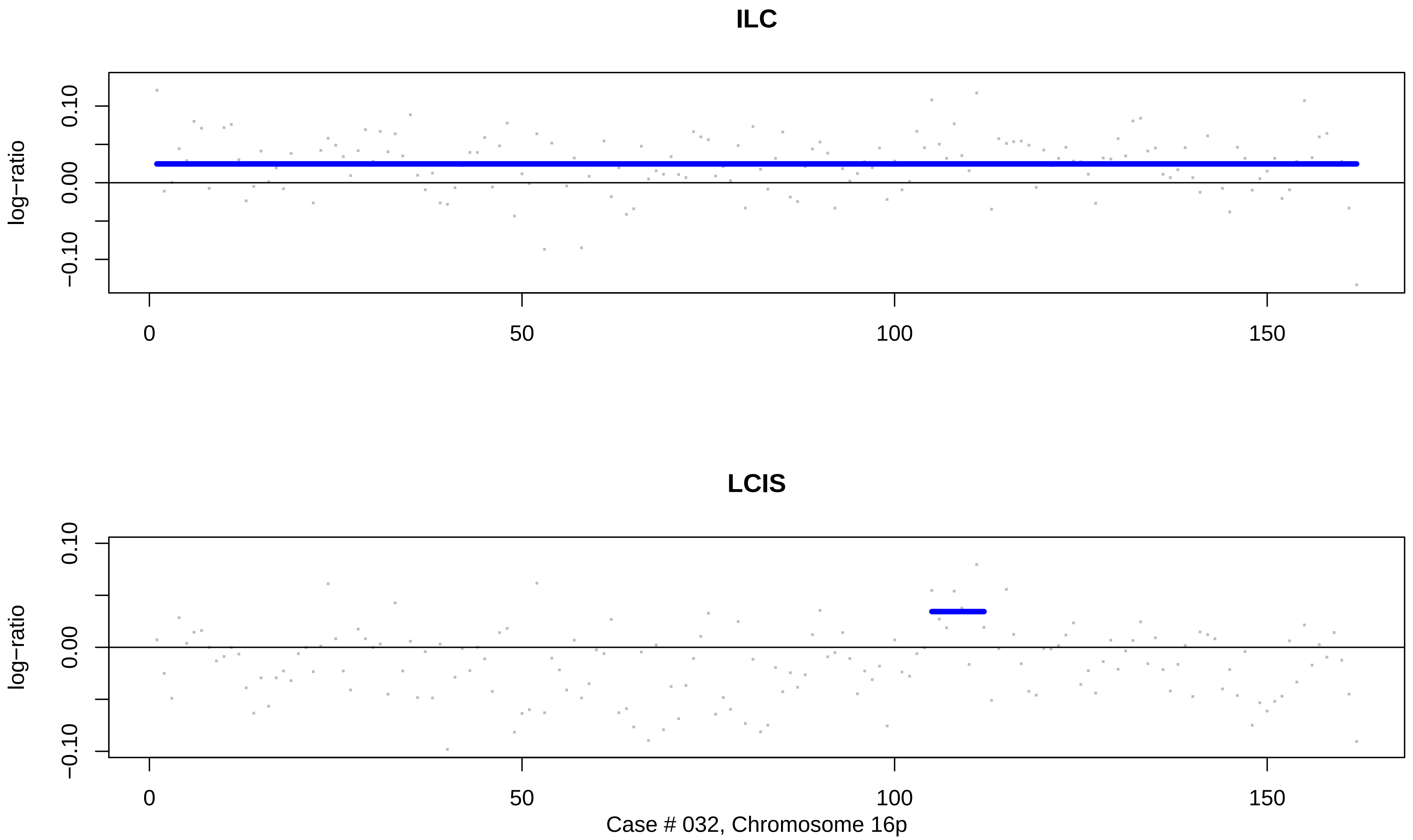
ILC
log−ratio
0.10
0.00
−0.10
0
50
100
150
LCIS
log−ratio
0.10
0.00
−0.10
0
50
100
150
Case # 032, Chromosome 16p

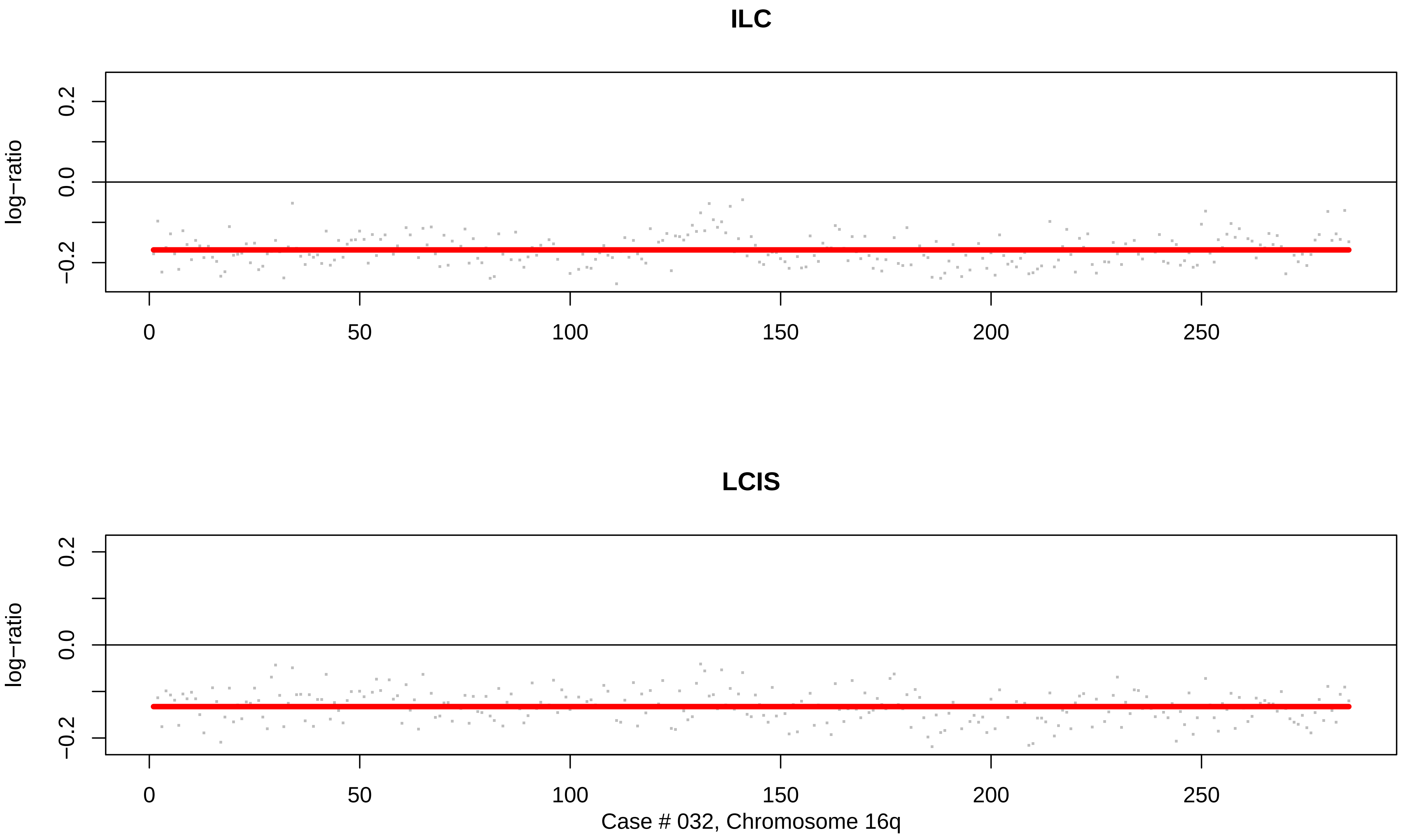

ILC
log−ratio
0.2
0.0
−0.2
0
50
100
150
200
250
LCIS
log−ratio
0.2
0.0
−0.2
0
50
100
150
200
250
Case # 032, Chromosome 16q

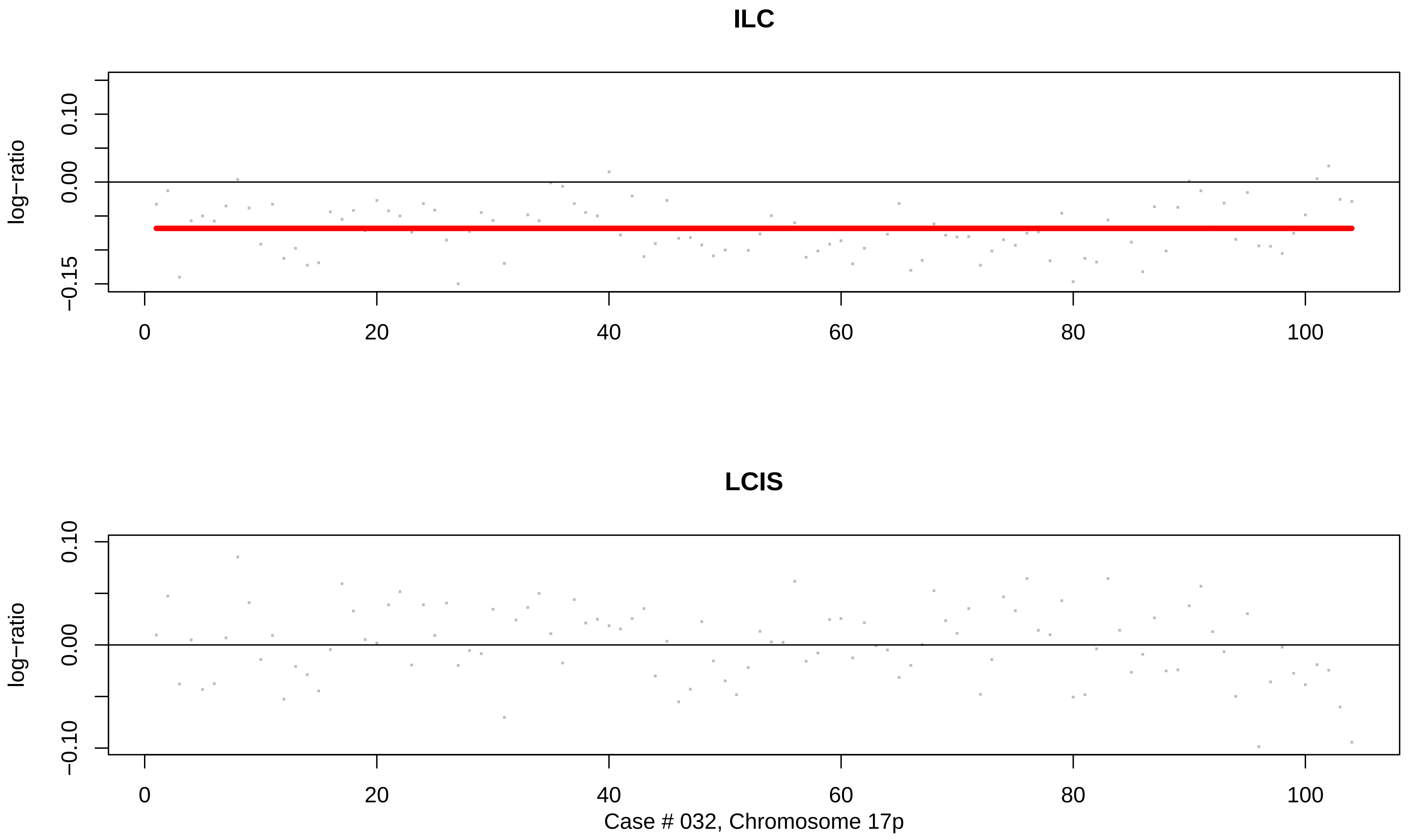
ILC
log−ratio
−0.15
0.00
0.10
0
20
40
60
80
100
LCIS
log−ratio
−0.10
0.00
0.10
0
20
40
60
80
100
Case # 032, Chromosome 17p

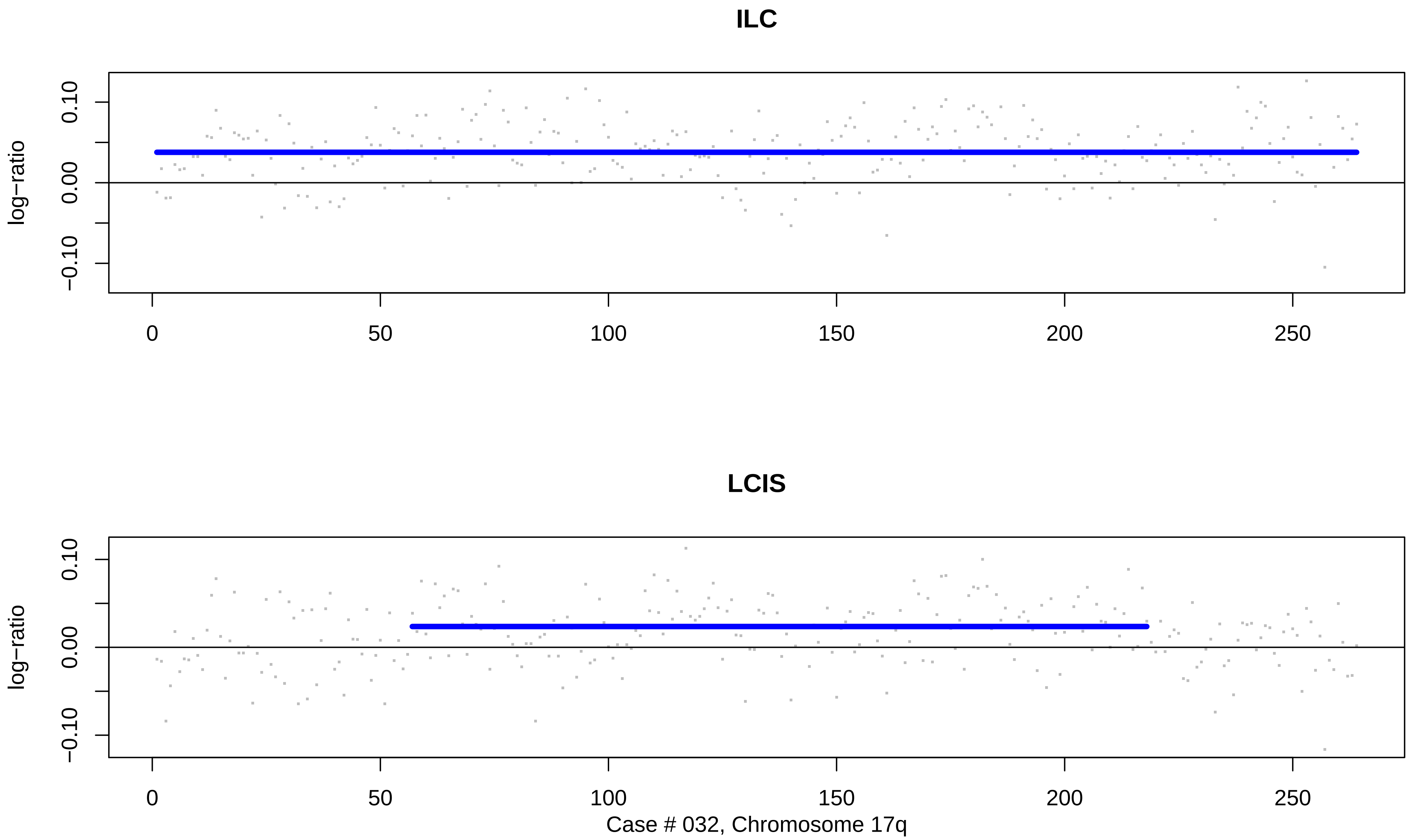
ILC
log−ratio
0.10
0.00
−0.10
0
50
100
150
200
250
LCIS
log−ratio
0.10
0.00
−0.10
0
50
100
150
200
250
Case # 032, Chromosome 17q

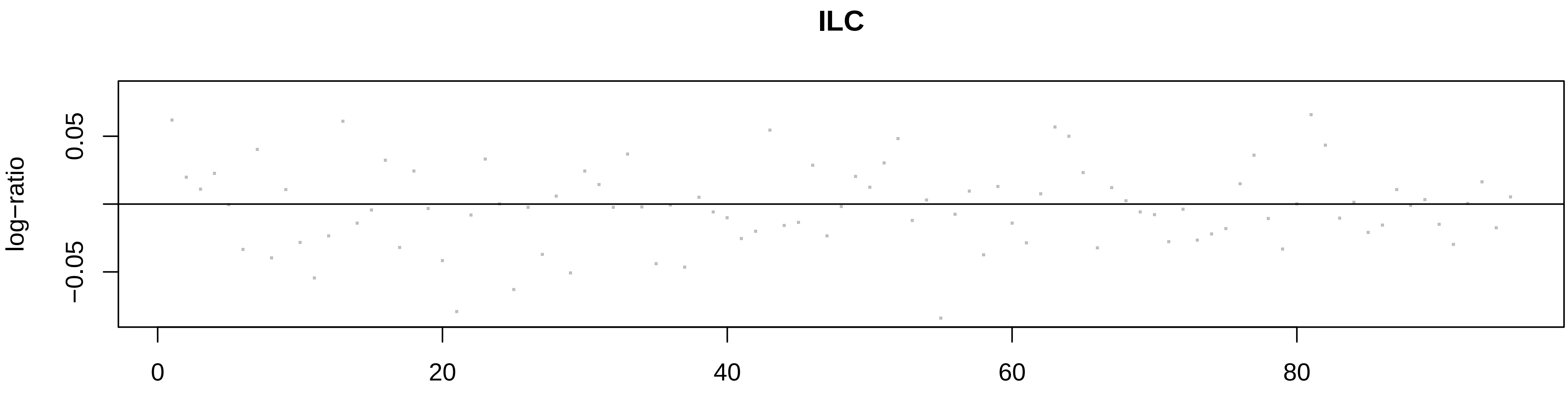

ILC
log−ratio
0.05
−0.05
0
20
40
60
80

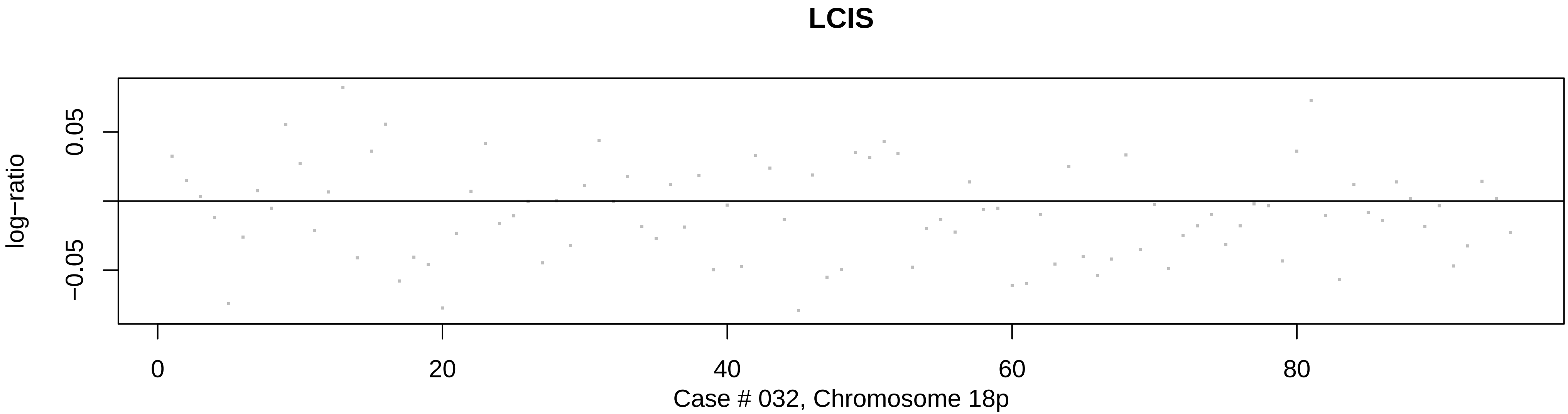

LCIS
log−ratio
0.05
−0.05
0
20
40
60
80
Case # 032, Chromosome 18p

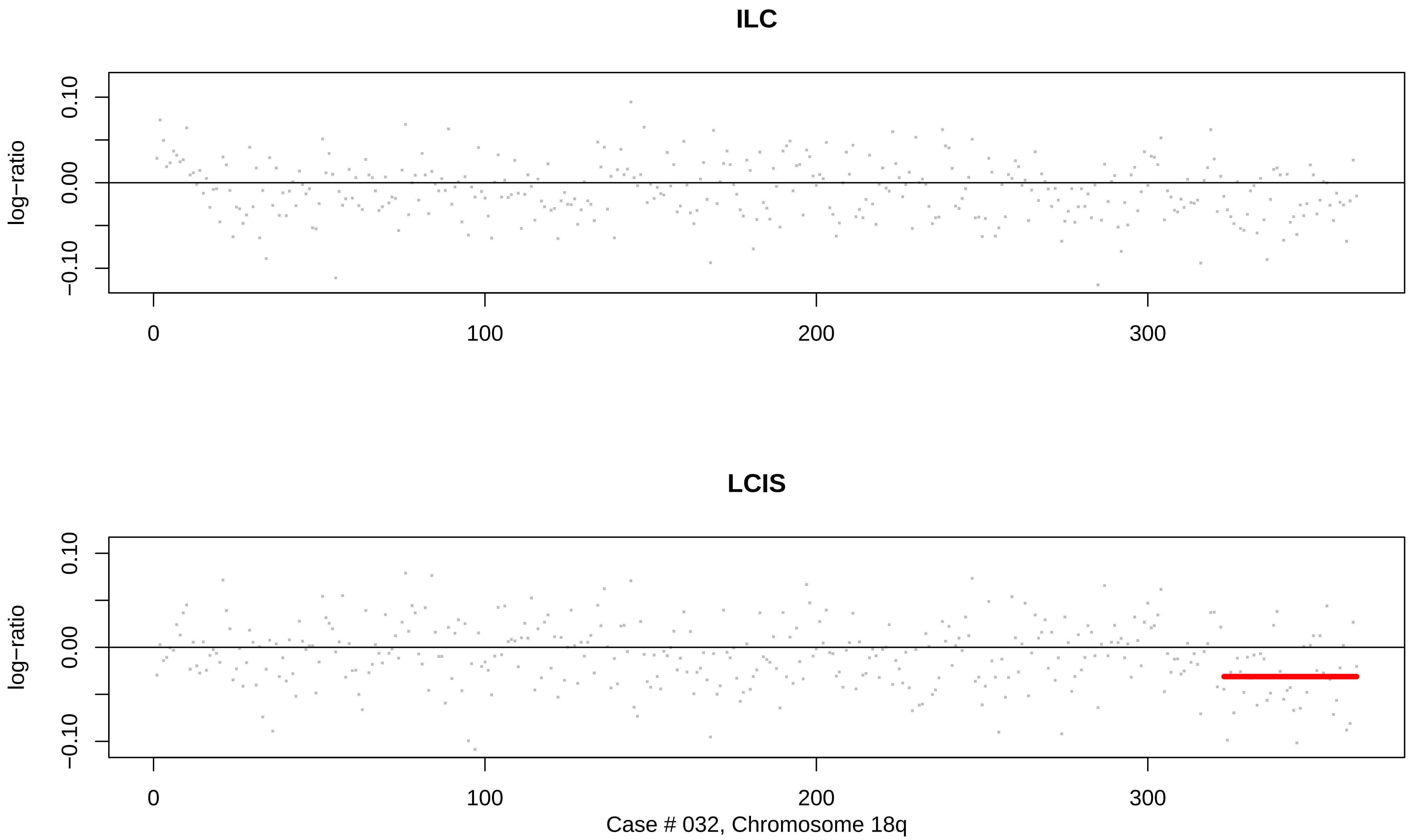
ILC
log−ratio
0.10
0.00
−0.10
0
100
200
300
LCIS
log−ratio
0.10
0.00
−0.10
0
100
200
300
Case # 032, Chromosome 18q

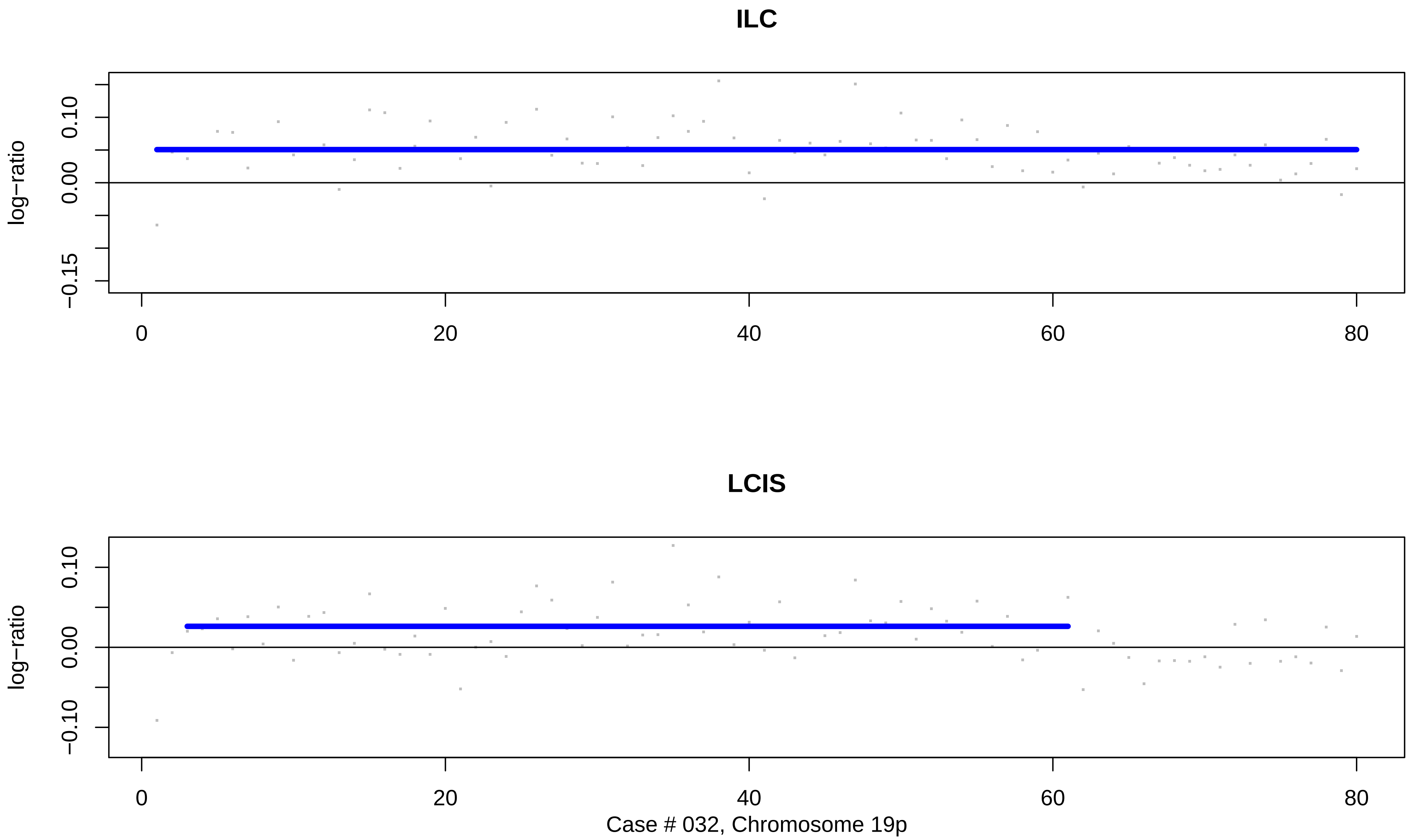
ILC
log−ratio
−0.15
0.00
0.10
0
20
40
60
80
LCIS
log−ratio
−0.10
0.00
0.10
0
20
40
60
80
Case # 032, Chromosome 19p

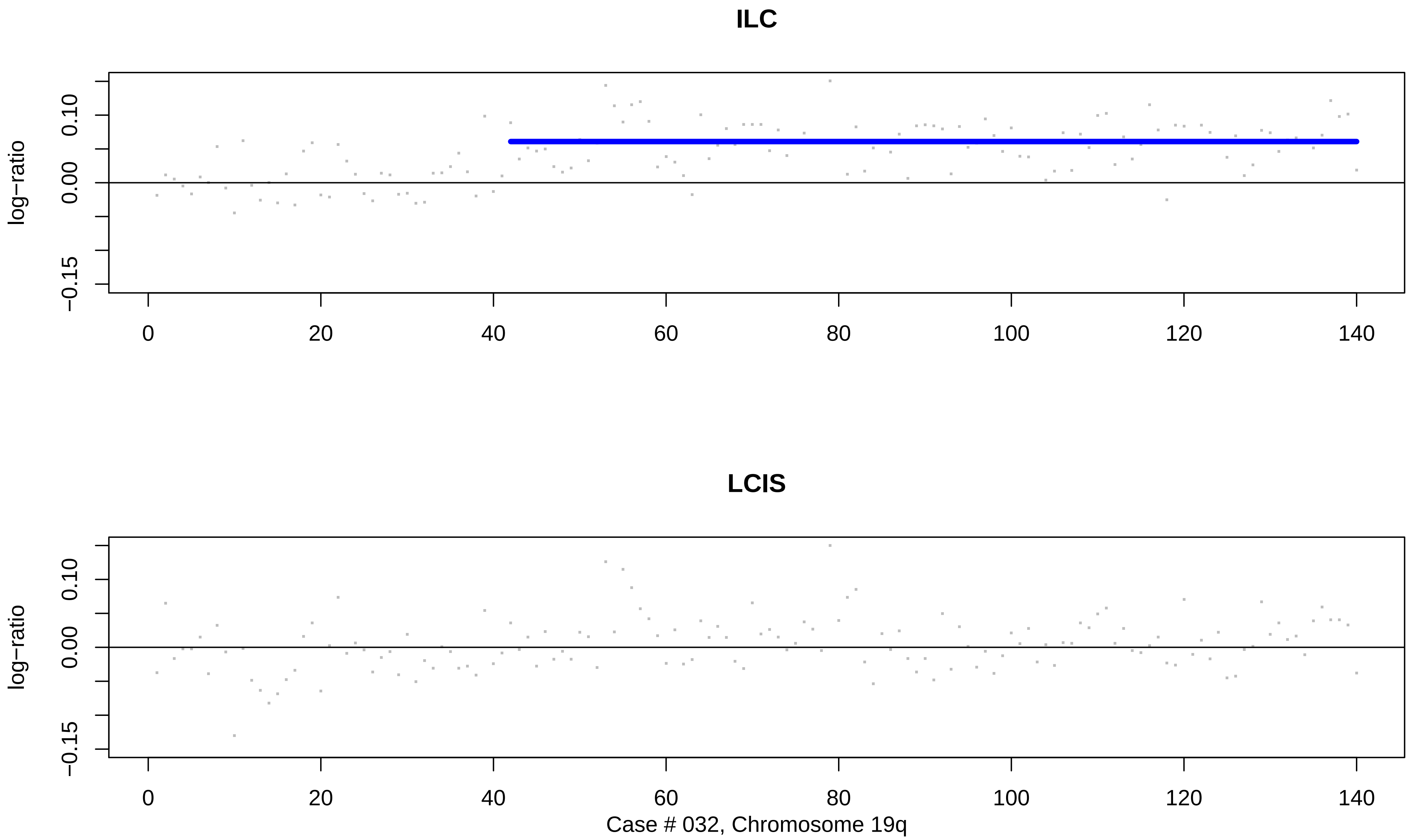
ILC
log−ratio
−0.15
0.00
0.10
0
20
40
60
80
100
120
140
LCIS
log−ratio
−0.15
0.00
0.10
0
20
40
60
80
100
120
140
Case # 032, Chromosome 19q

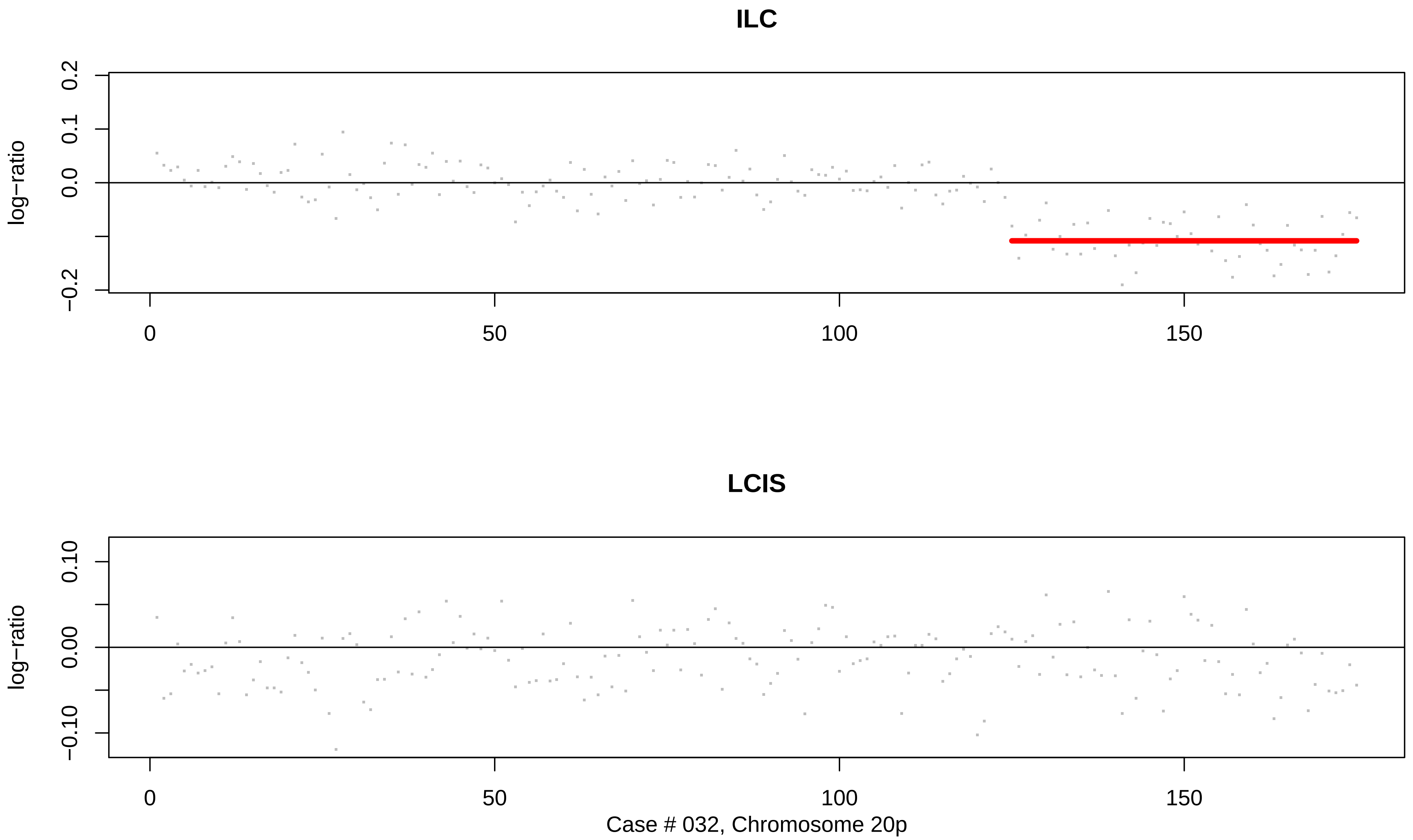

ILC
log−ratio
0.2
0.1
0.0
−0.2
0
50
100
150
LCIS
log−ratio
0.10
0.00
−0.10
0
50
100
150
Case # 032, Chromosome 20p

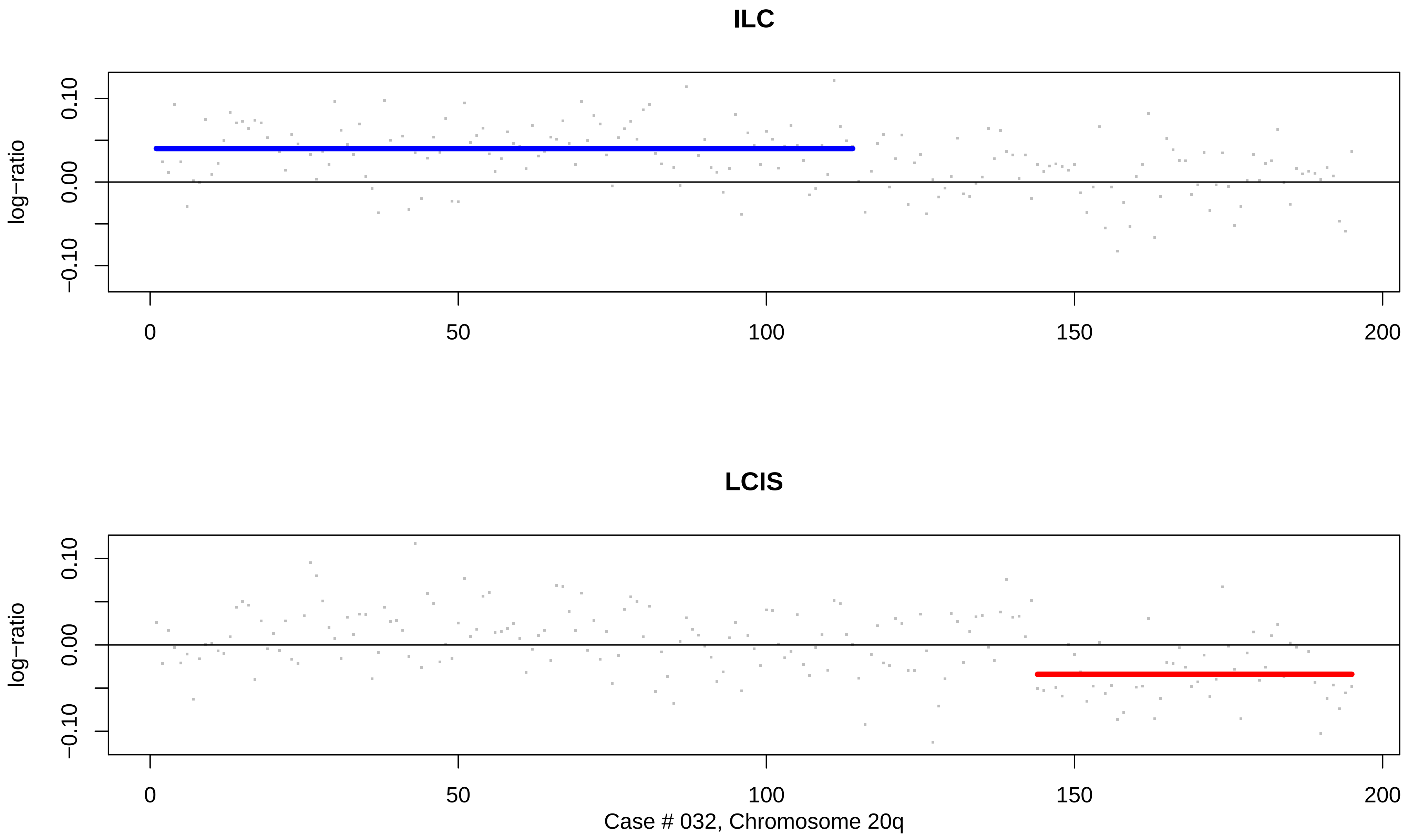

ILC
log−ratio
0.10
0.00
−0.10
0
50
100
150
200
LCIS
log−ratio
0.10
0.00
−0.10
0
50
100
150
200
Case # 032, Chromosome 20q

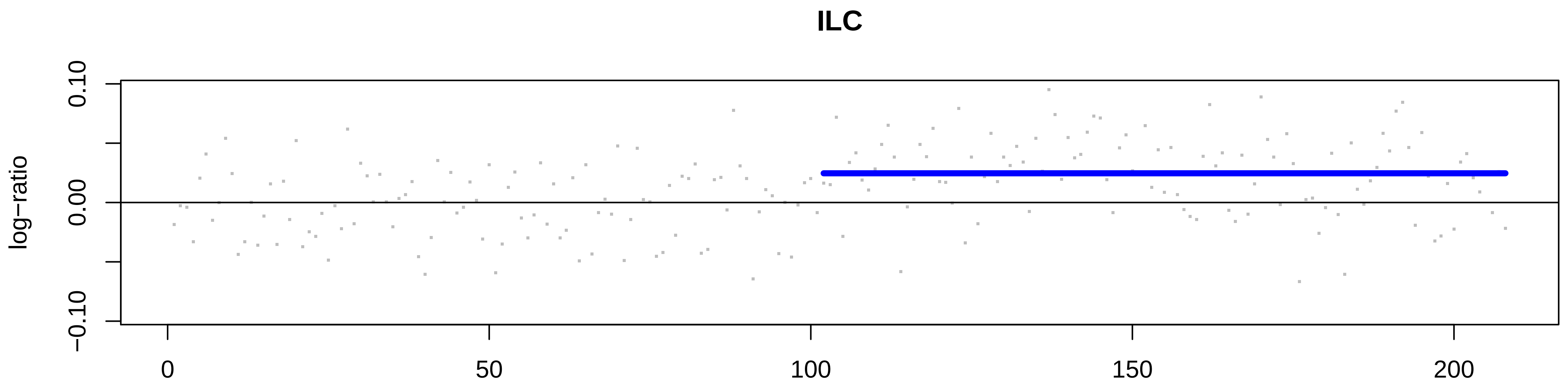

ILC
log−ratio
0.10
0.00
−0.10
0
50
100
150
200

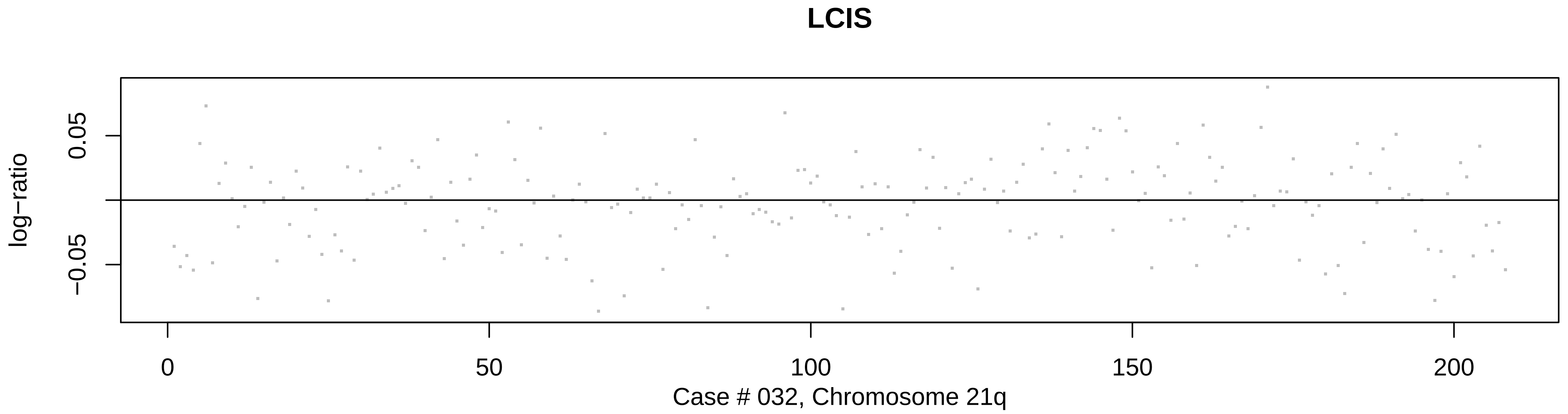

LCIS
log−ratio
0.05
−0.05
0
50
100
150
200
Case # 032, Chromosome 21q

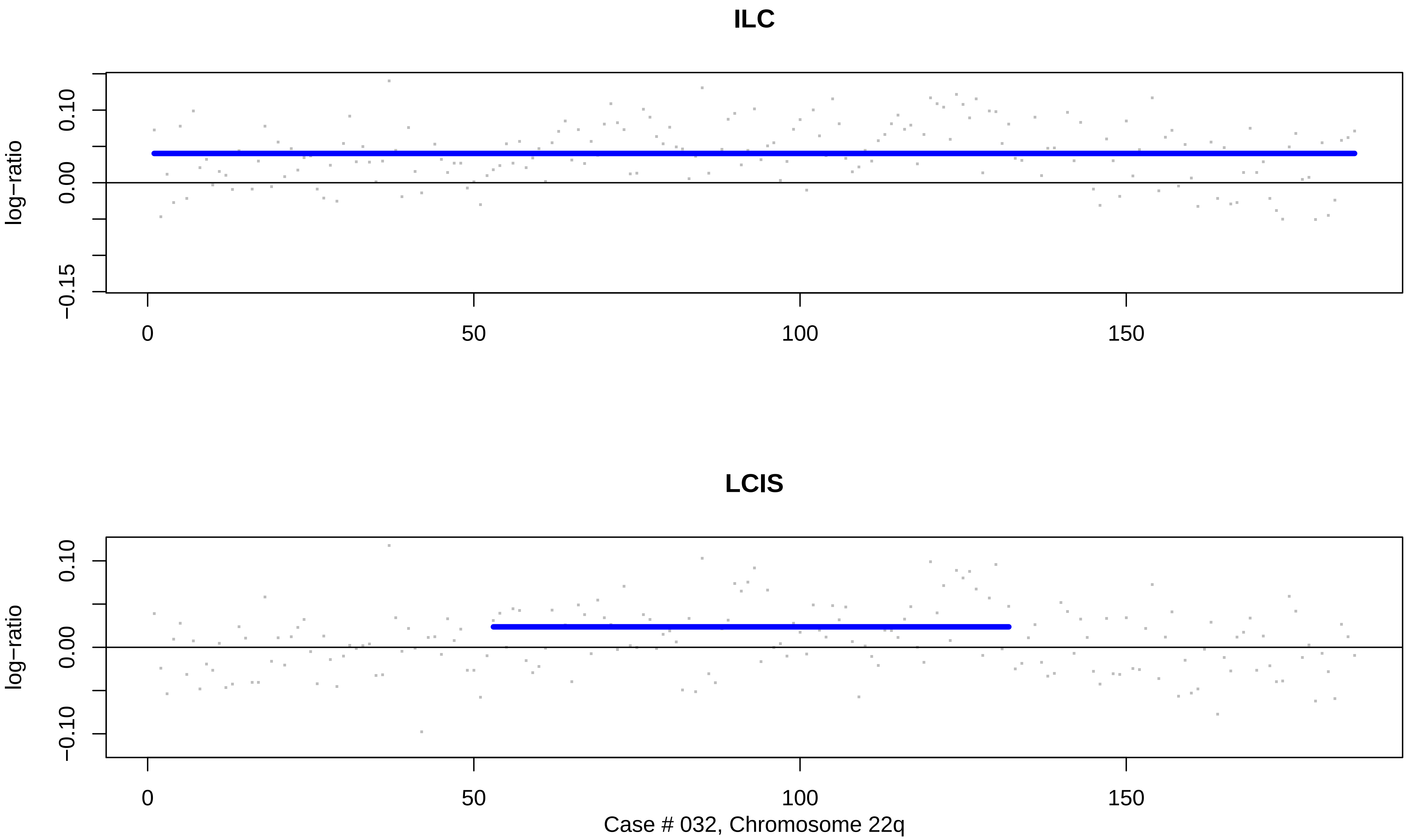

ILC
log−ratio
−0.15
0.00
0.10
0
50
100
150
LCIS
log−ratio
−0.10
0.00
0.10
0
50
100
150
Case # 032, Chromosome 22q
