# Supplementary material for: Clonal relatedness between lobular carcinoma in situ and synchronous malignant lesions
Source: Breast Cancer Res. 2012 Jul 9;14(4):R103. doi: 10.1186/bcr3222 (PMC3680923; doi:10.1186/bcr3222)
Supplement: Additional file 4 — Magnified version of genome-wide plots with detailed marker plots and segmentation on a chromosome-arm-specific basis. [file bcr3222-S4.ZIP › Case 114 b.pdf]

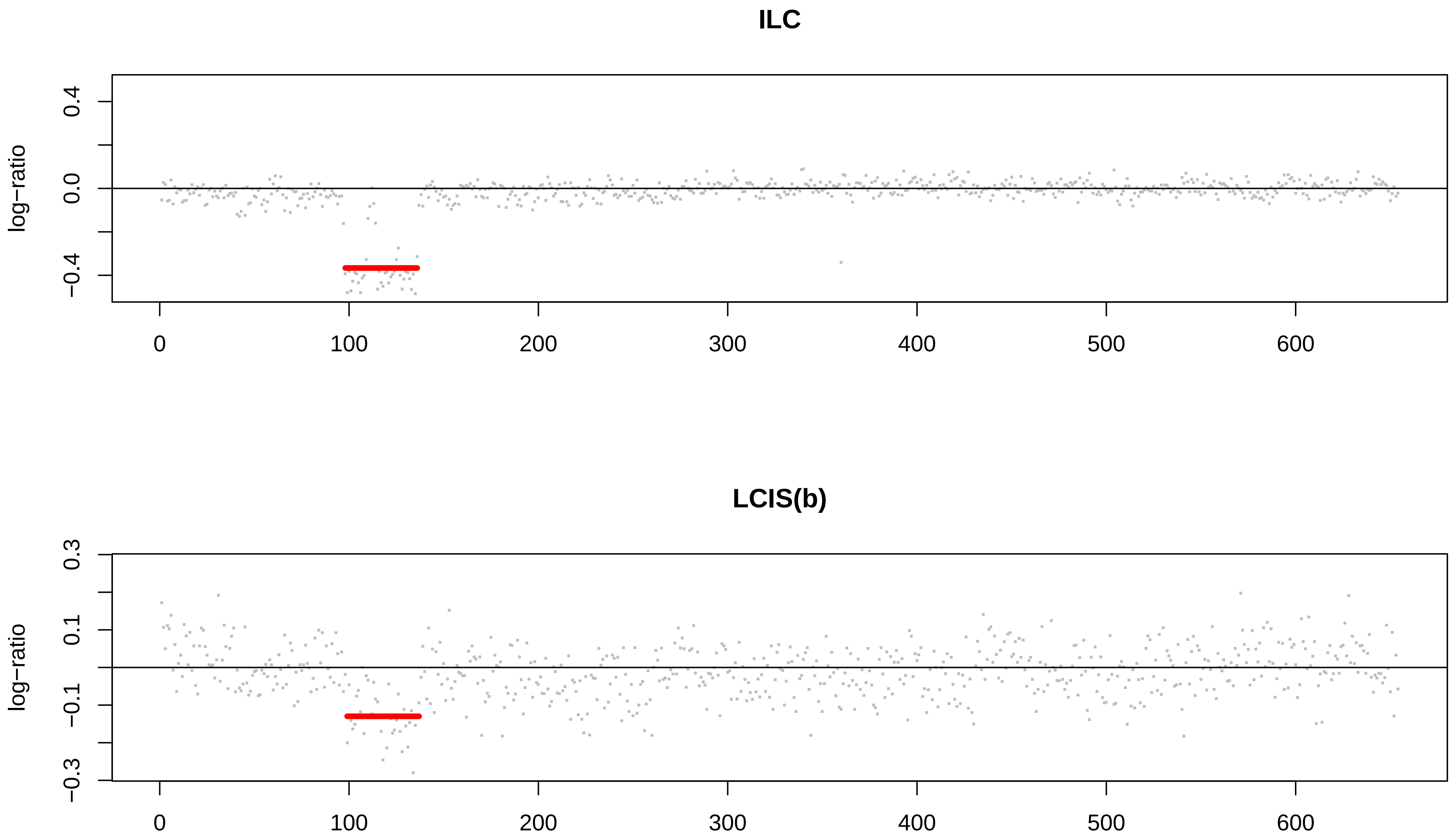

Case # 114, Chromosome 01p
Odds in favor of clonality = 84.3

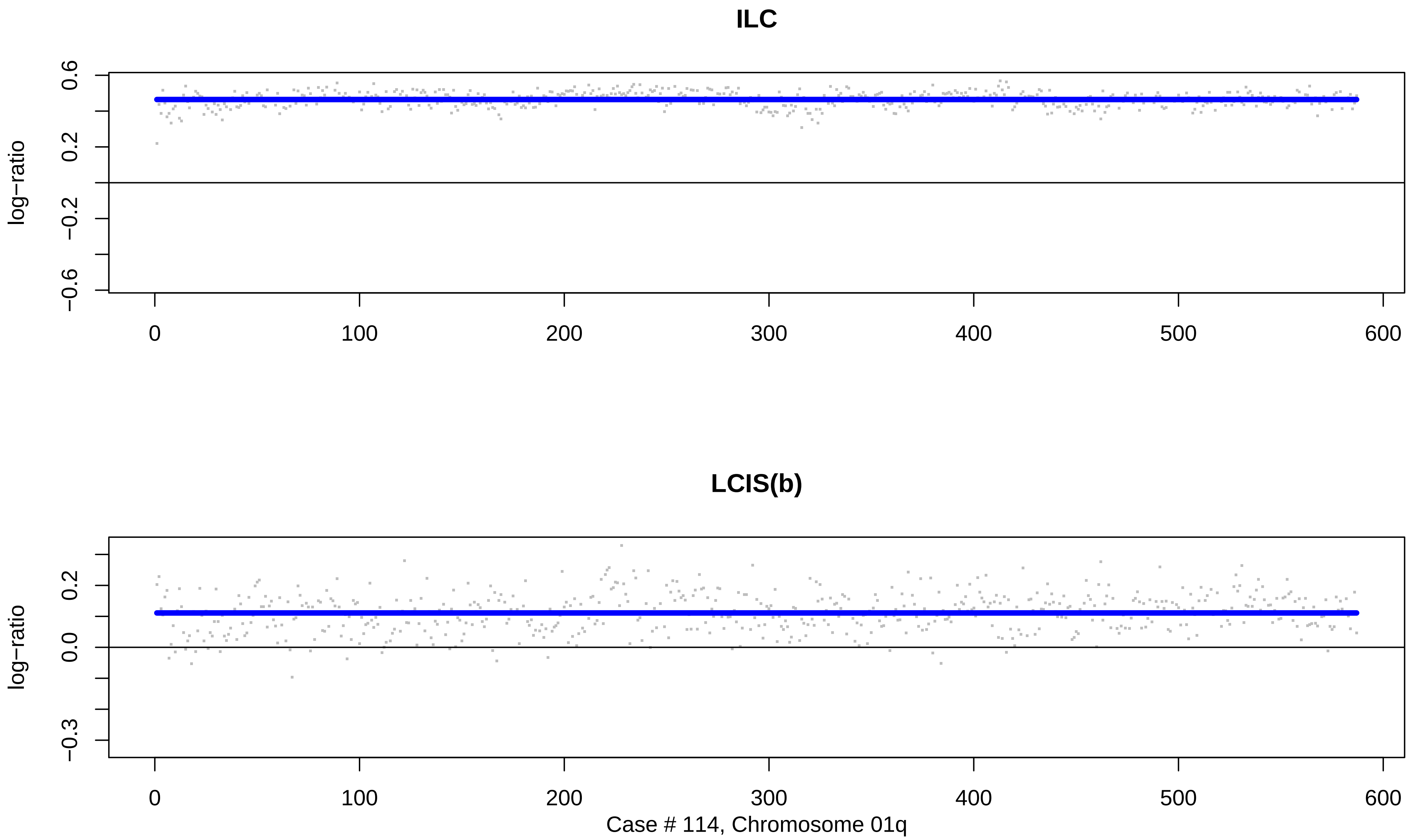
ILC
log−ratio
0.6
0.2
−0.2
−0.6
0
100
200
300
400
500
600
LCIS(b)
log−ratio
0.2
0.0
−0.3
0
100
200
300
400
500
600
Case # 114, Chromosome 01q

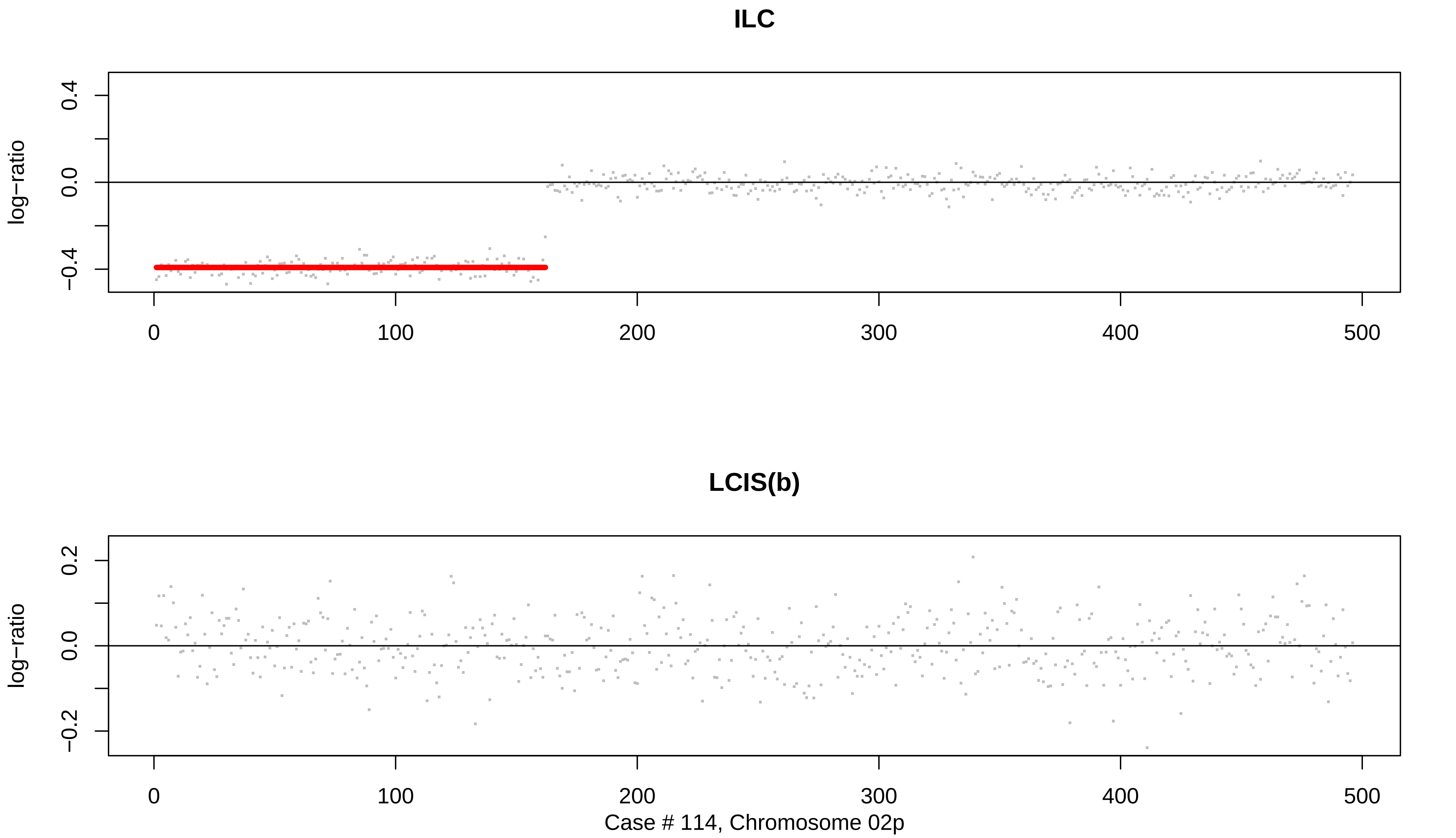
ILC
log−ratio
0.4
0.0
−0.4
0
100
200
300
400
500
LCIS(b)
log−ratio
0.2
0.0
−0.2
0
100
200
300
400
500
Case # 114, Chromosome 02p

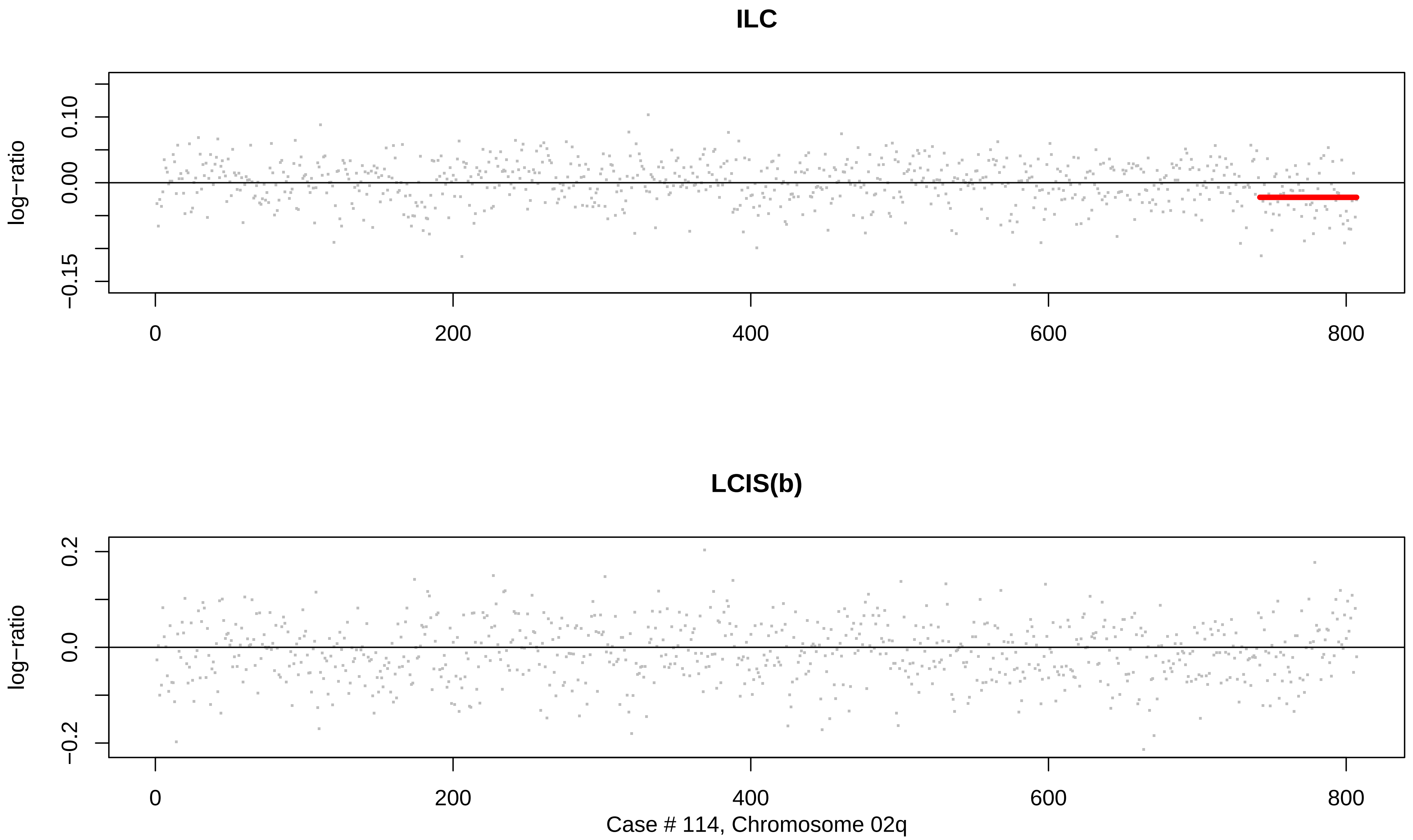
ILC
log−ratio
0.10
0.00
−0.15
0
200
400
600
800
LCIS(b)
log−ratio
0.2
0.0
−0.2
0
200
400
600
800
Case # 114, Chromosome 02q

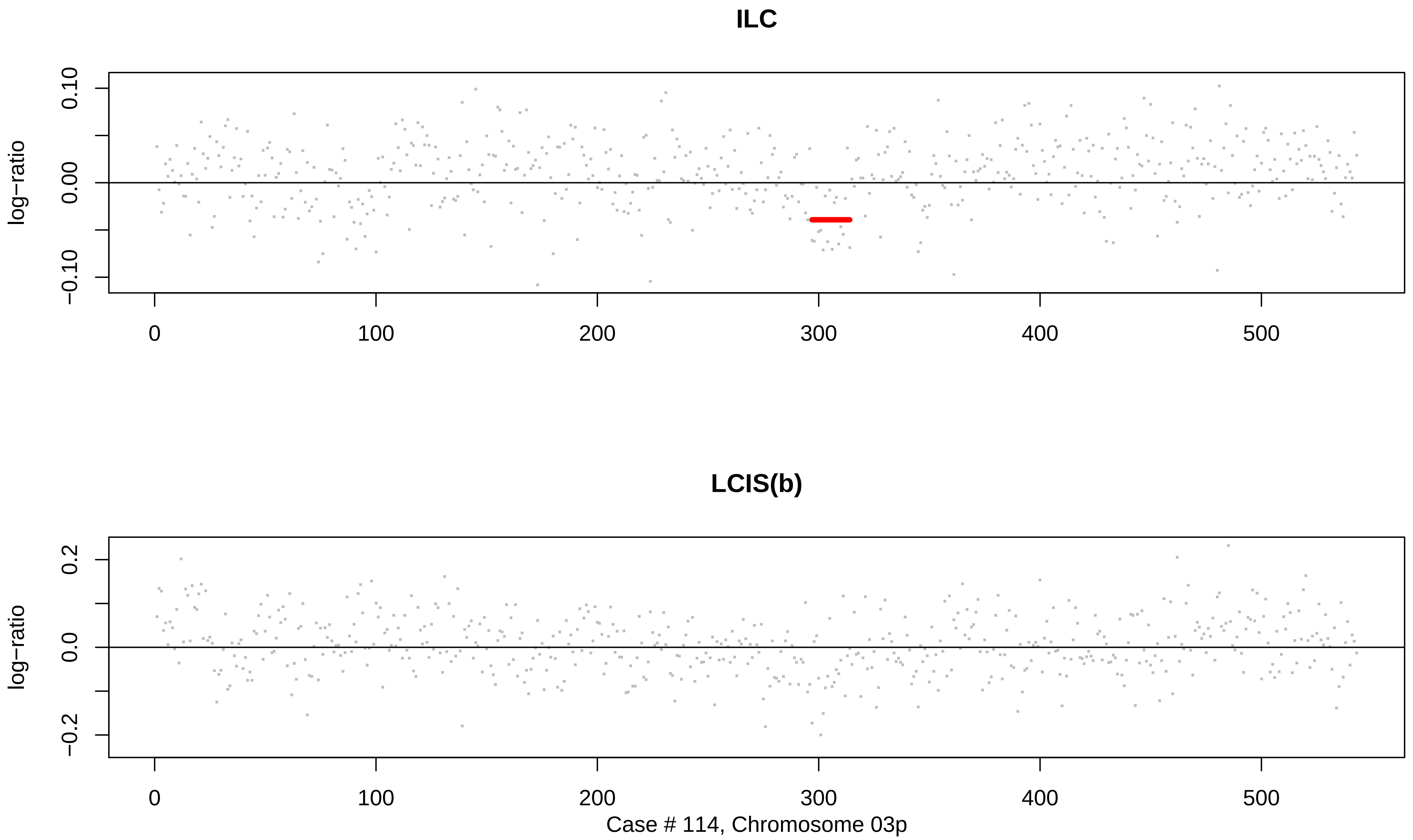
ILC
log−ratio
0.10
0.00
−0.10
0
100
200
300
400
500
LCIS(b)
log−ratio
0.2
0.0
−0.2
0
100
200
300
400
500
Case # 114, Chromosome 03p

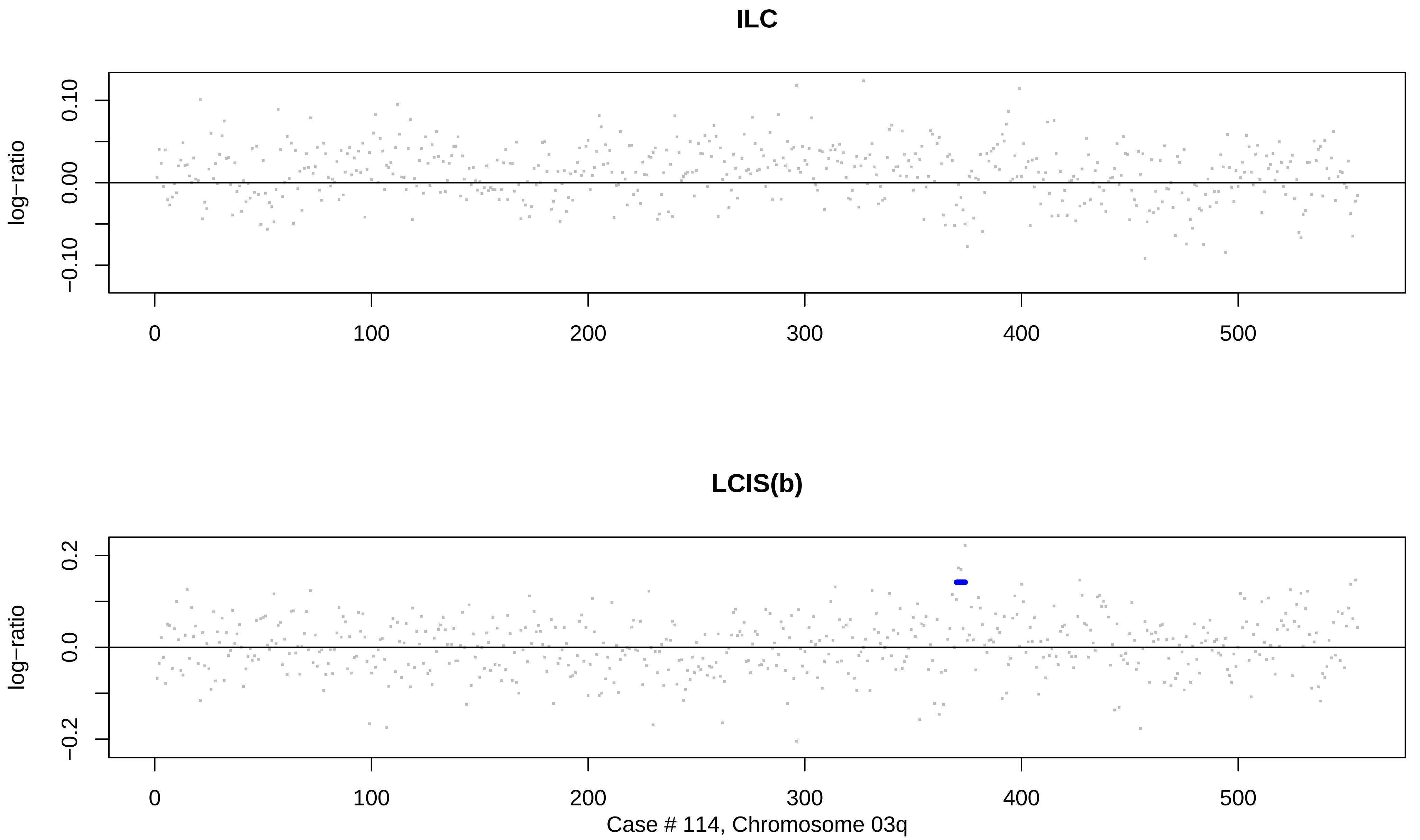
ILC
log−ratio
0.10
0.00
−0.10
0
100
200
300
400
500
LCIS(b)
log−ratio
0.2
0.0
−0.2
0
100
200
300
400
500
Case # 114, Chromosome 03q

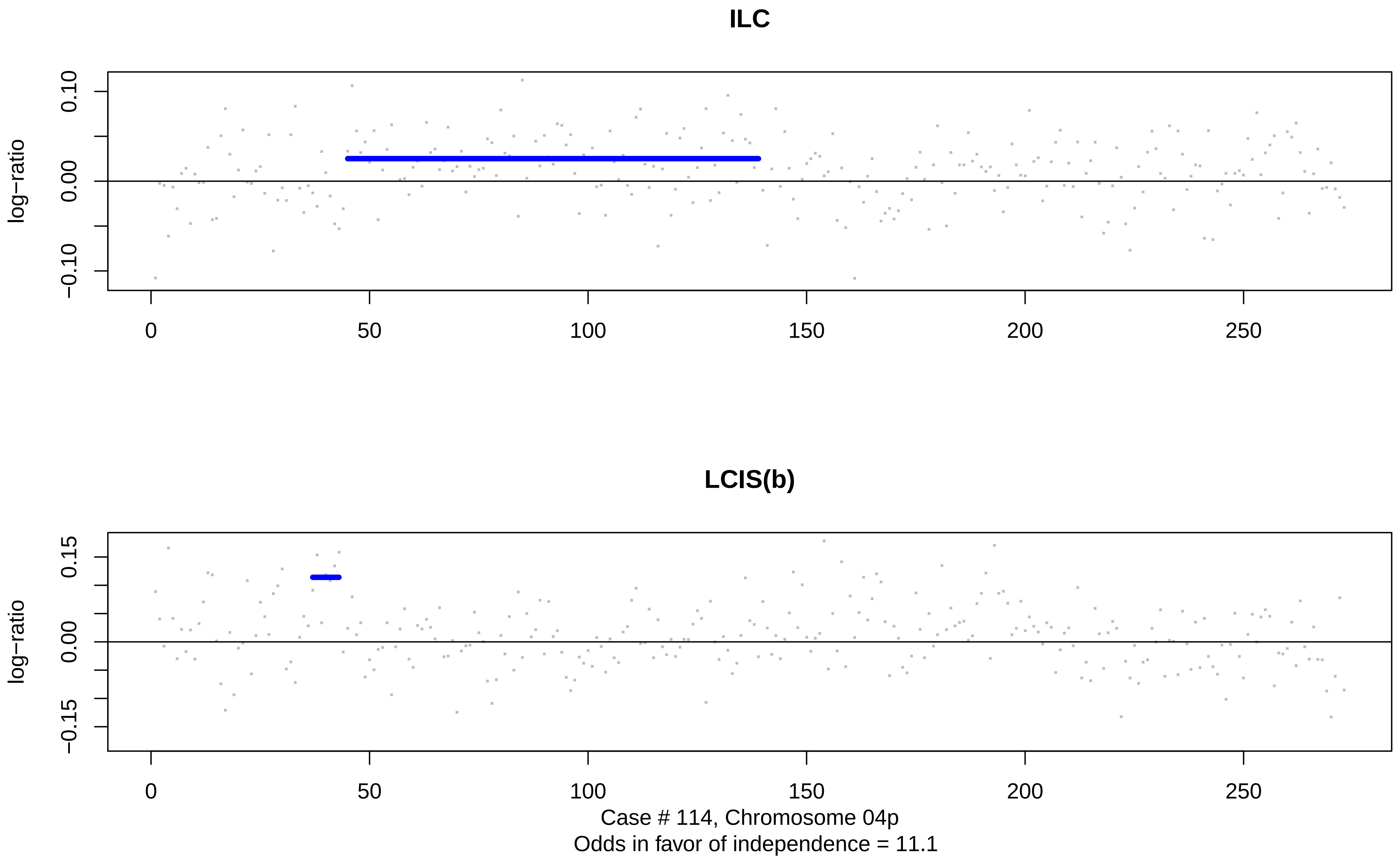

ILC
log−ratio
0.10
0.00
−0.10
0
50
100
150
200
250
LCIS(b)
log−ratio
0.15
0.00
−0.15
0
50
100
150
200
250
Case # 114, Chromosome 04p
Odds in favor of independence = 11.1

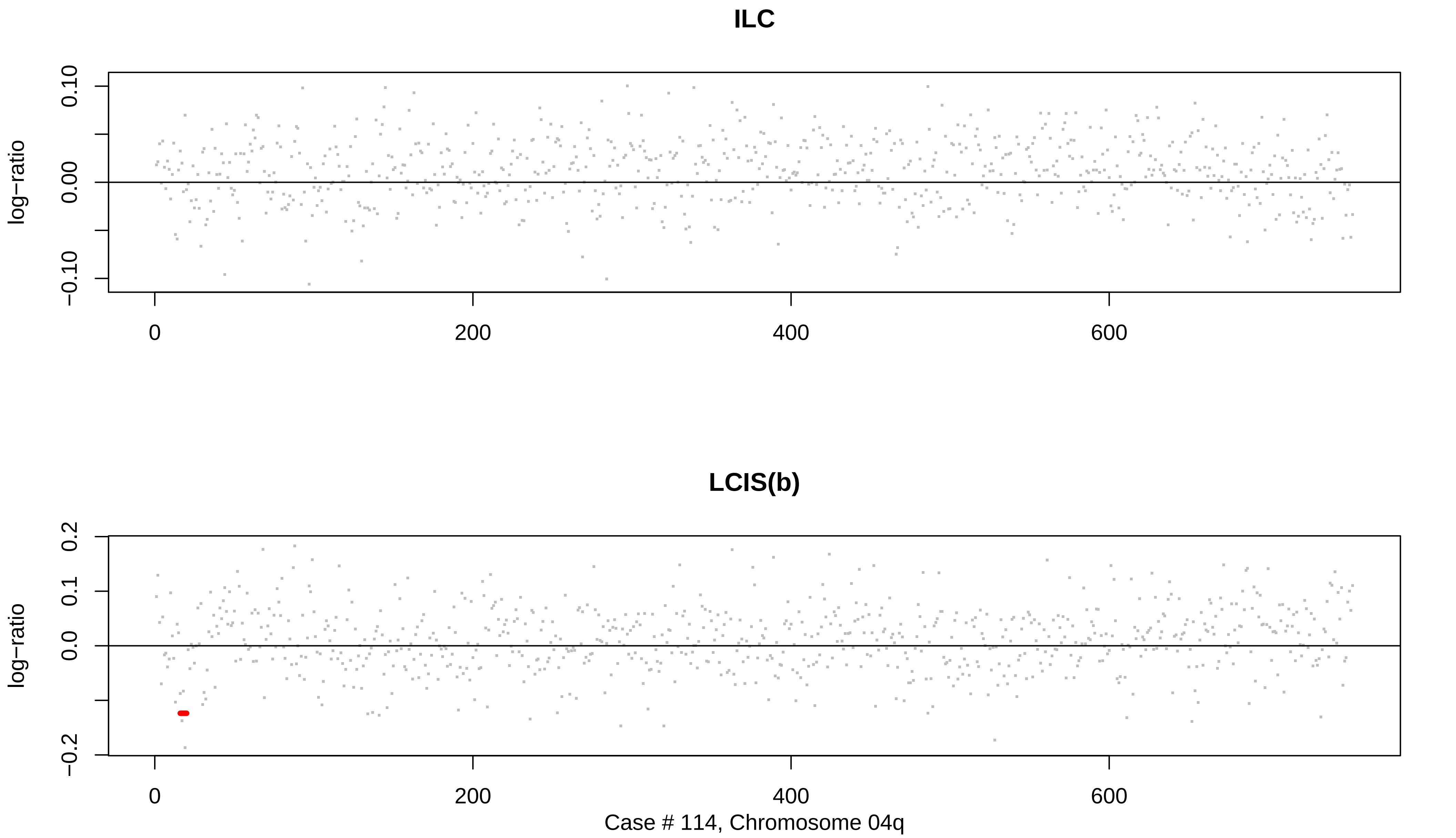

ILC
log−ratio
0.10
0.00
−0.10
0
200
400
600
LCIS(b)
log−ratio
0.2
0.1
0.0
−0.2
0
200
400
600
Case # 114, Chromosome 04q

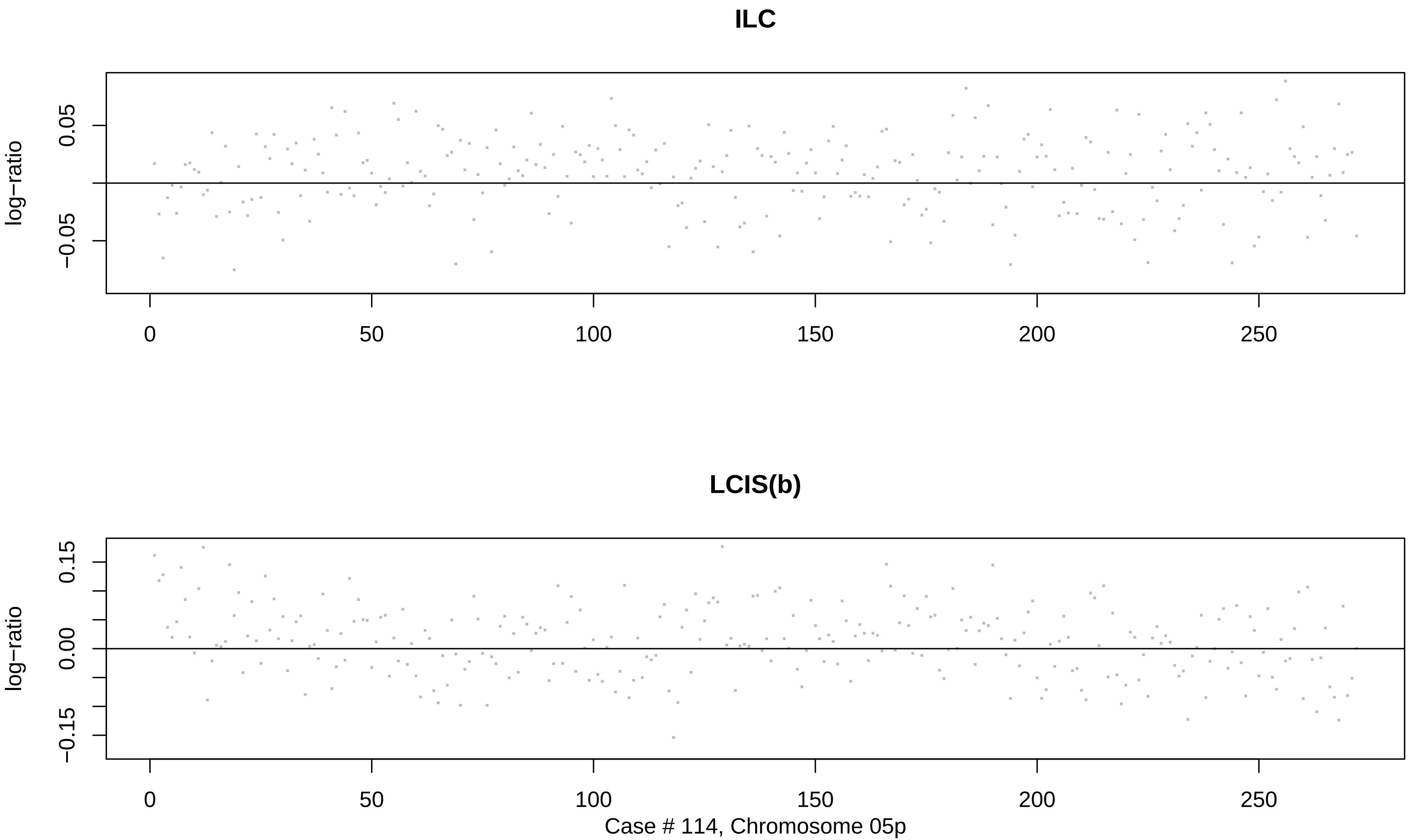

ILC
log−ratio
0.05
−0.05
0
50
100
150
200
250
LCIS(b)
log−ratio
0.15
0.00
−0.15
0
50
100
150
200
250
Case # 114, Chromosome 05p

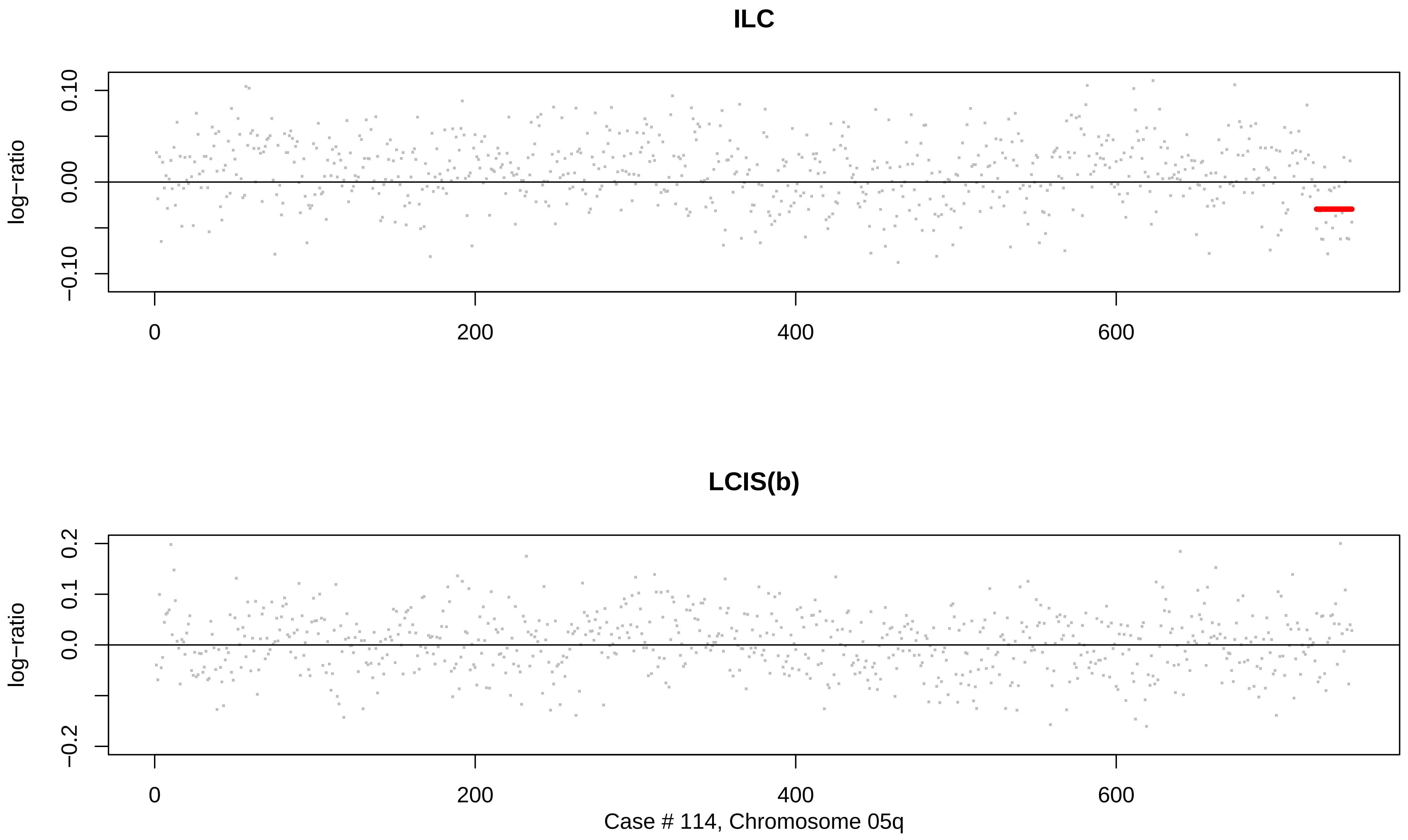
ILC
log−ratio
−0.10
0.00
0.10
0
200
400
600
LCIS(b)
log−ratio
−0.2
0.0
0.1
0.2
0
200
400
600
Case # 114, Chromosome 05q

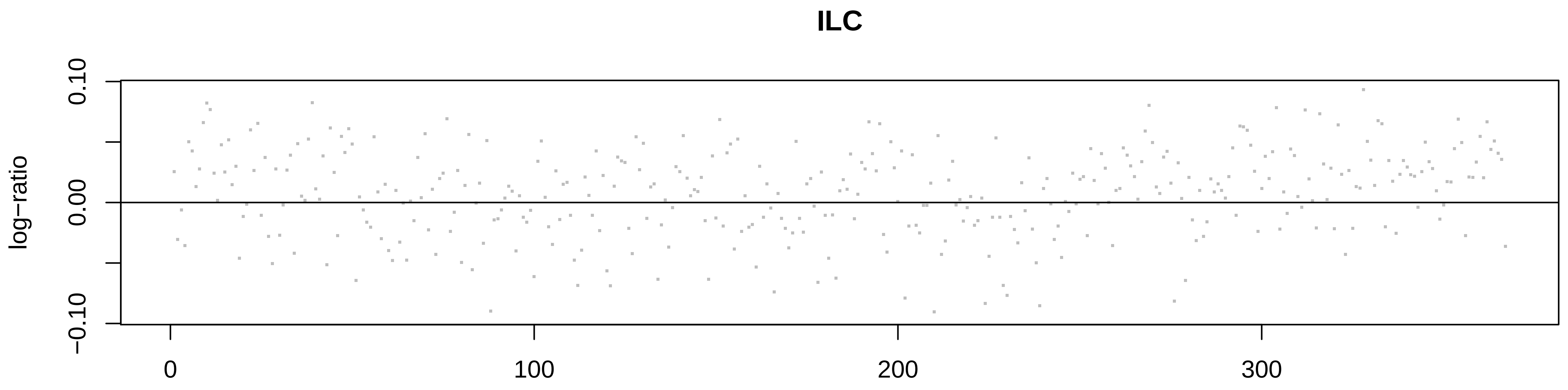
ILC
log−ratio
0.10
0.00
−0.10
0
100
200
300

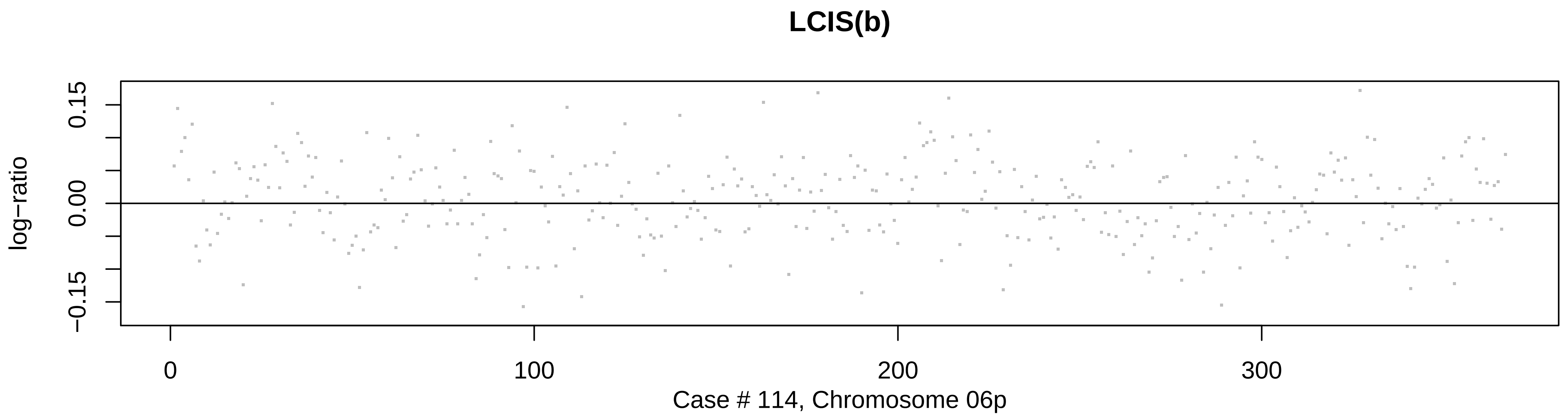
LCIS(b)
log−ratio
0.15
0.00
−0.15
0
100
200
300
Case # 114, Chromosome 06p

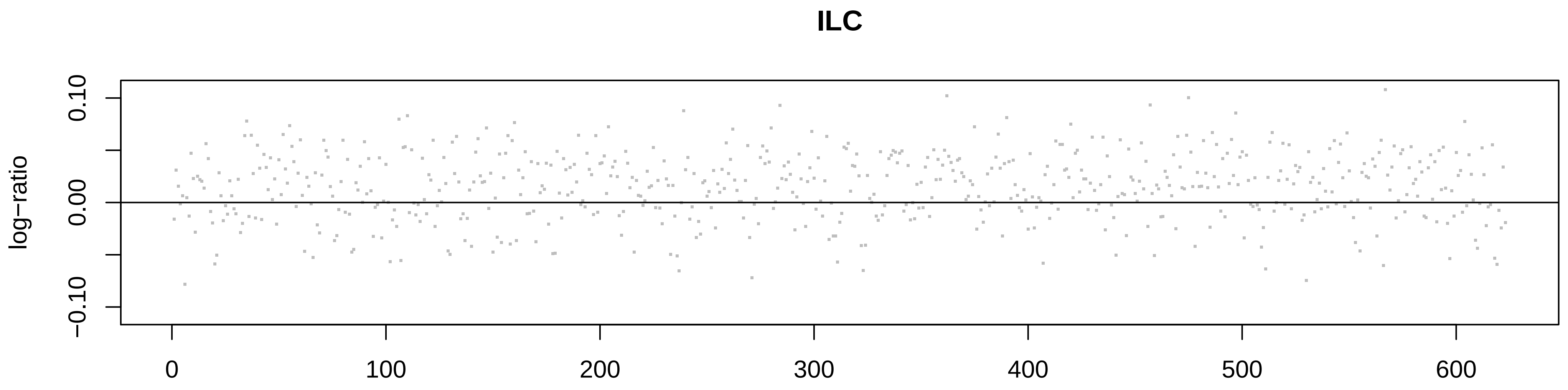
ILC
log−ratio
0.10
0.00
−0.10
0
100
200
300
400
500
600

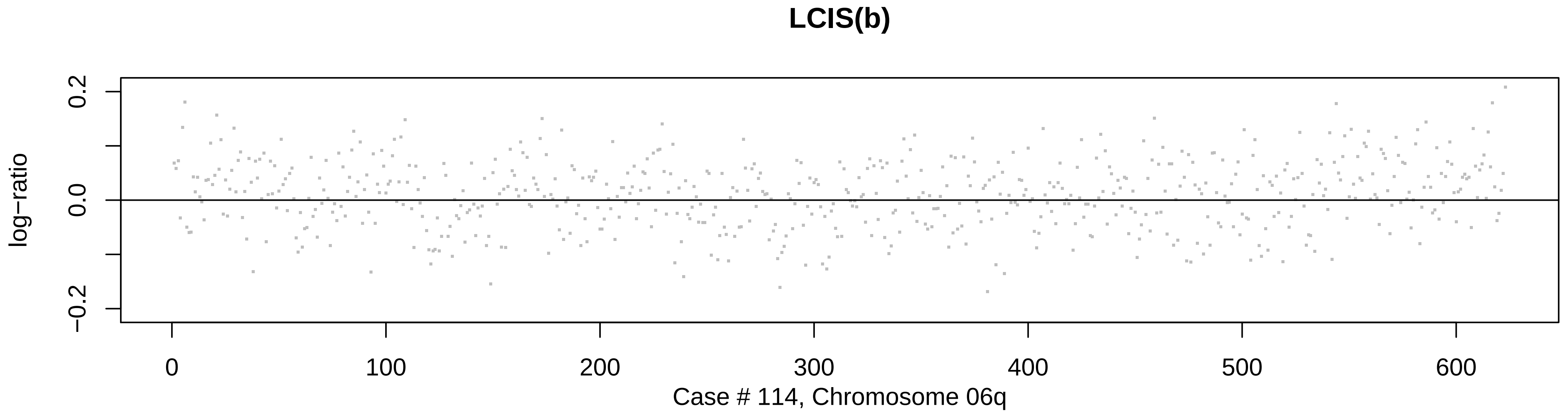
LCIS(b)
log−ratio
0.2
0.0
−0.2
0
100
200
300
400
500
600
Case # 114, Chromosome 06q

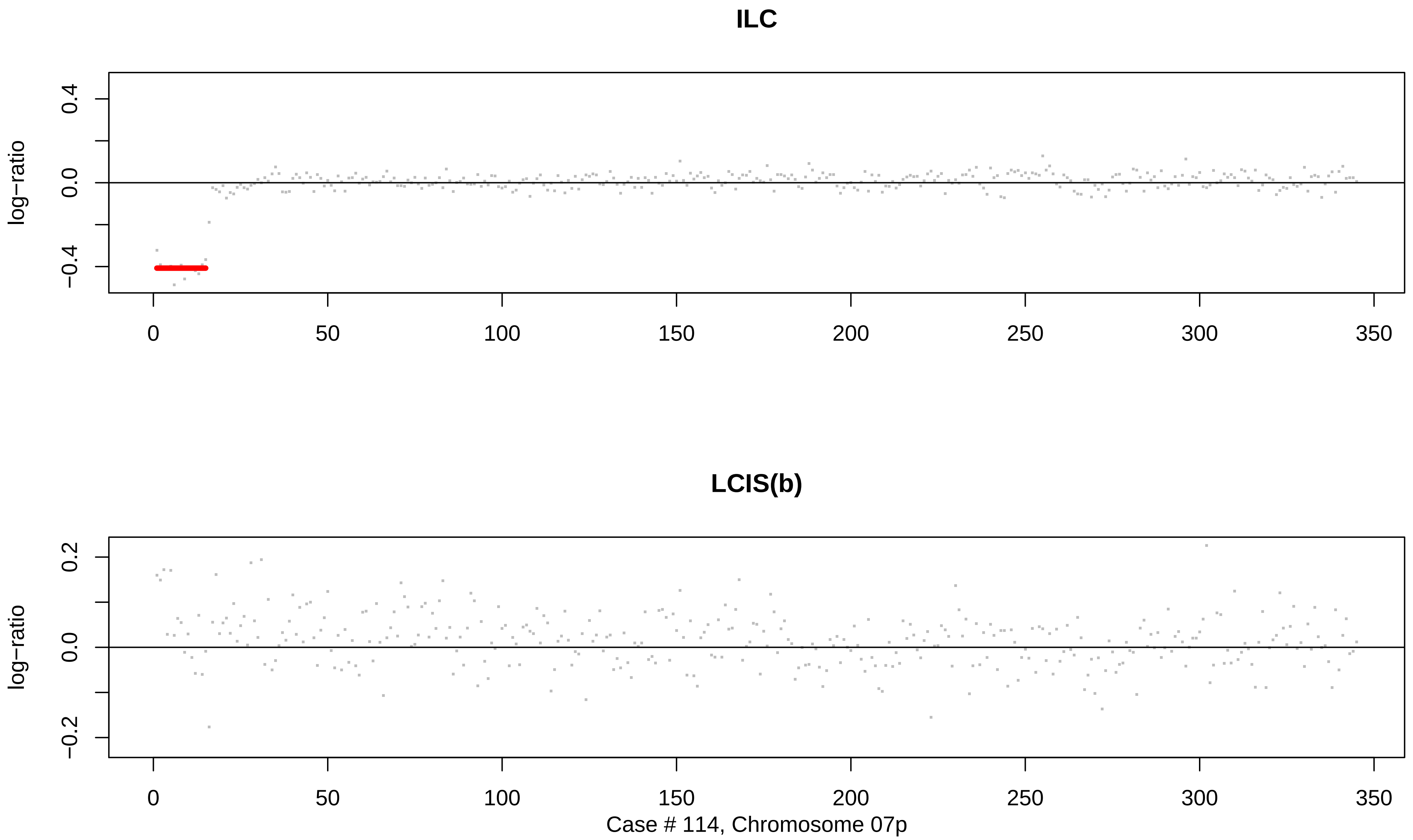
ILC
log−ratio
0.4
0.0
−0.4
0
50
100
150
200
250
300
350
LCIS(b)
log−ratio
0.2
0.0
−0.2
0
50
100
150
200
250
300
350
Case # 114, Chromosome 07p

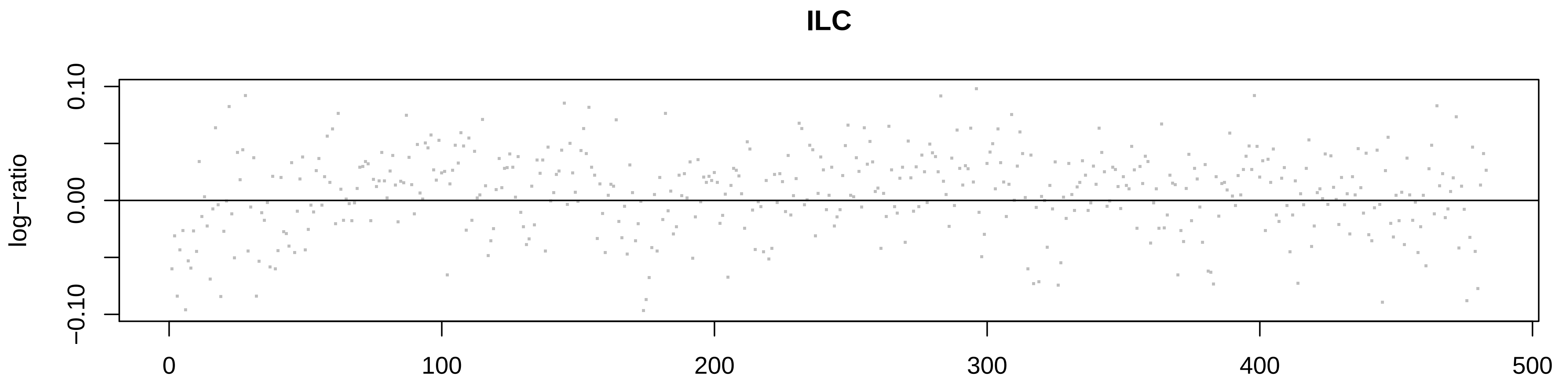
ILC
log−ratio
0.10
0.00
−0.10
0
100
200
300
400
500

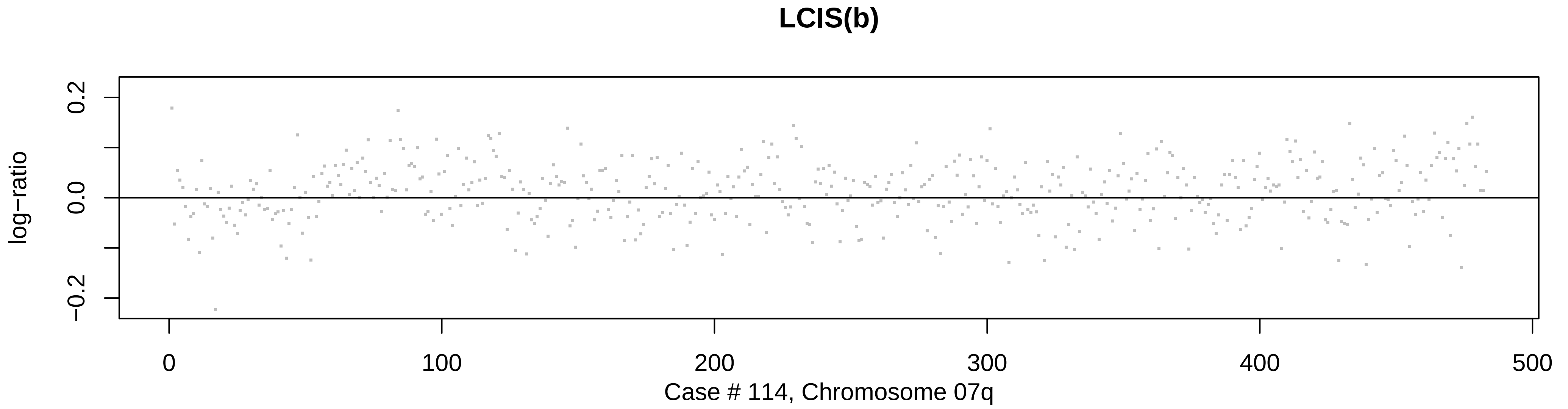
LCIS(b)
log−ratio
0.2
0.0
−0.2
0
100
200
300
400
500
Case # 114, Chromosome 07q

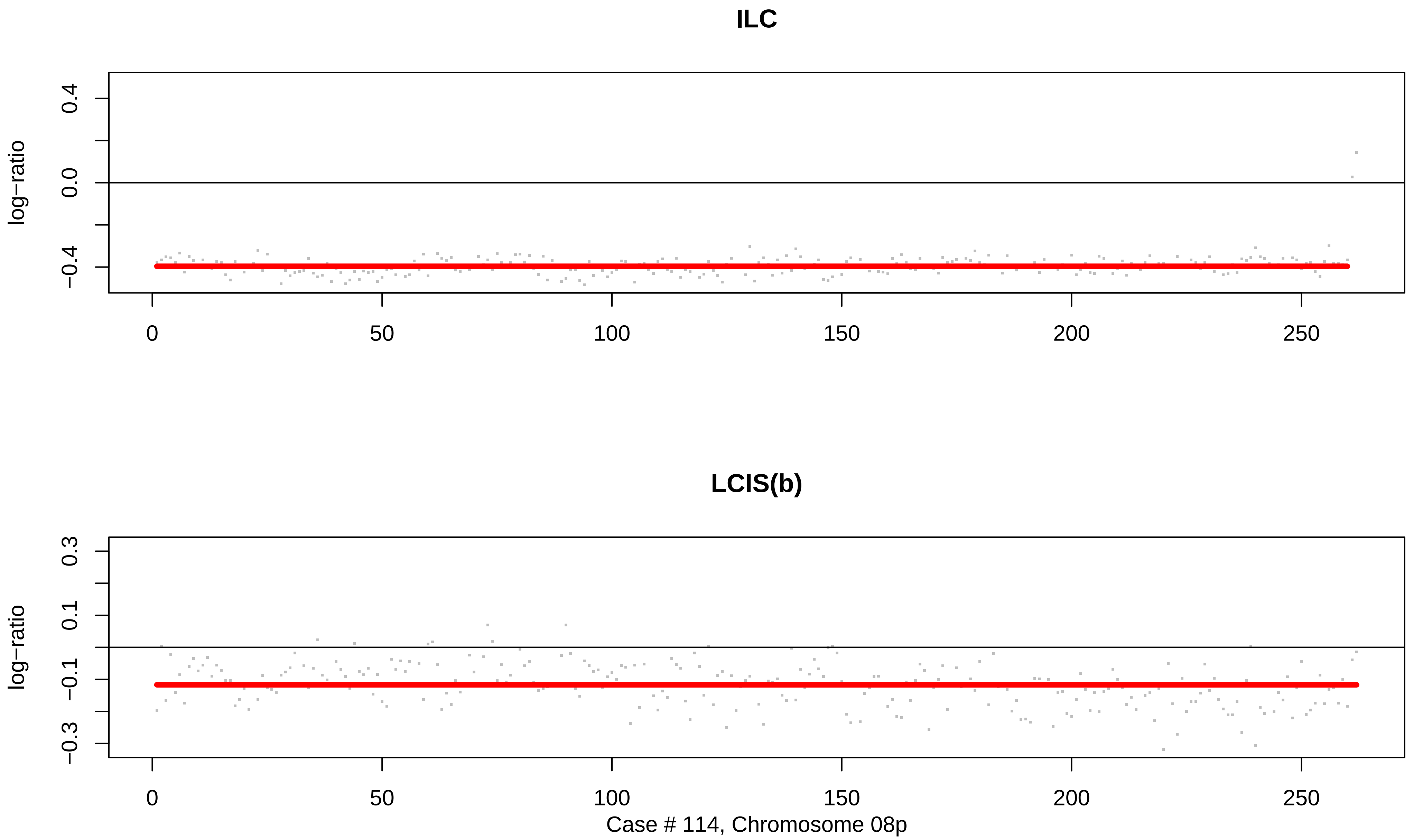
ILC
log−ratio
0.4
0.0
−0.4
0
50
100
150
200
250
LCIS(b)
log−ratio
0.3
0.1
−0.1
−0.3
0
50
100
150
200
250
Case # 114, Chromosome 08p

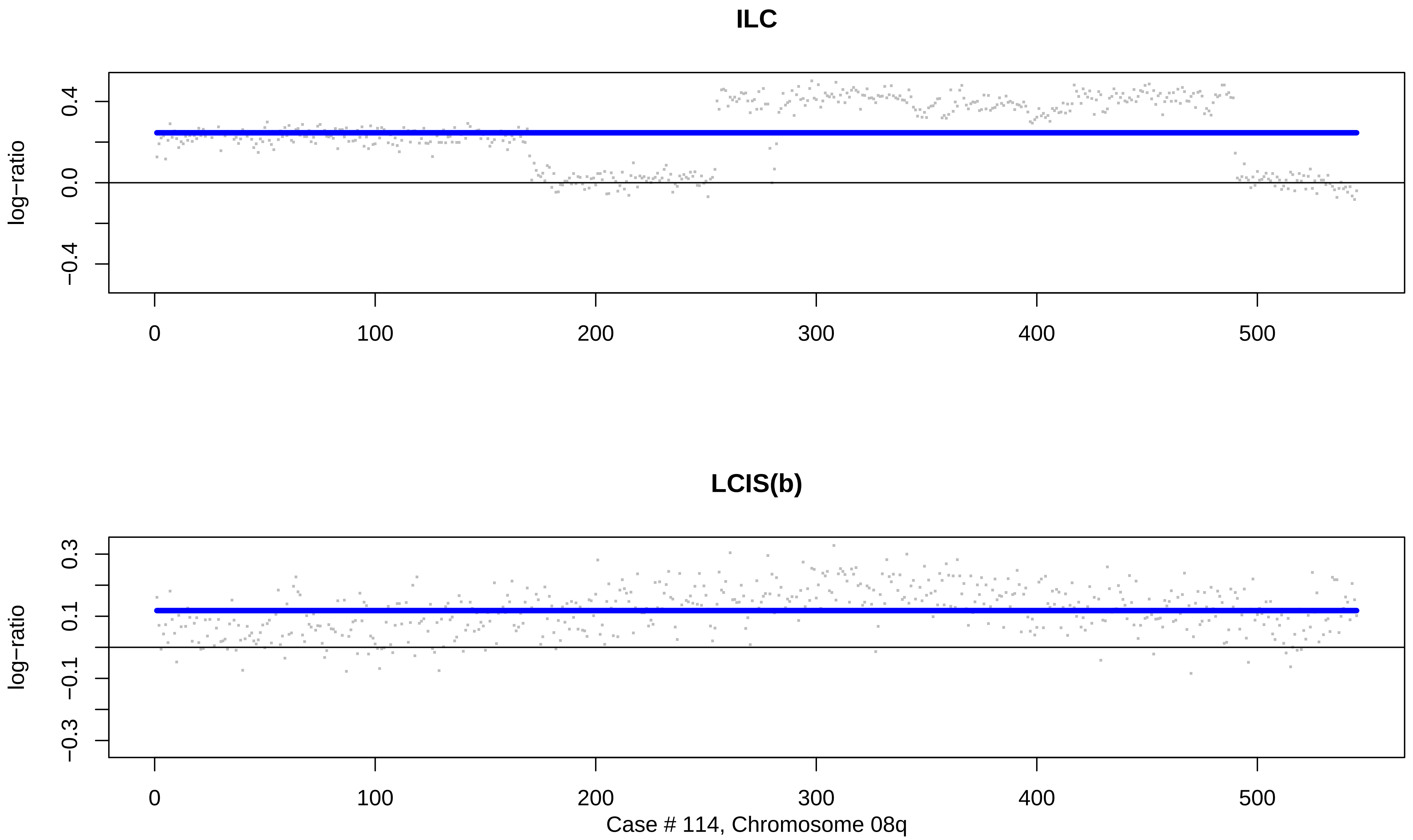
ILC
log−ratio
0.4
0.0
−0.4
0
100
200
300
400
500
LCIS(b)
log−ratio
0.3
0.1
−0.1
−0.3
0
100
200
300
400
500
Case # 114, Chromosome 08q

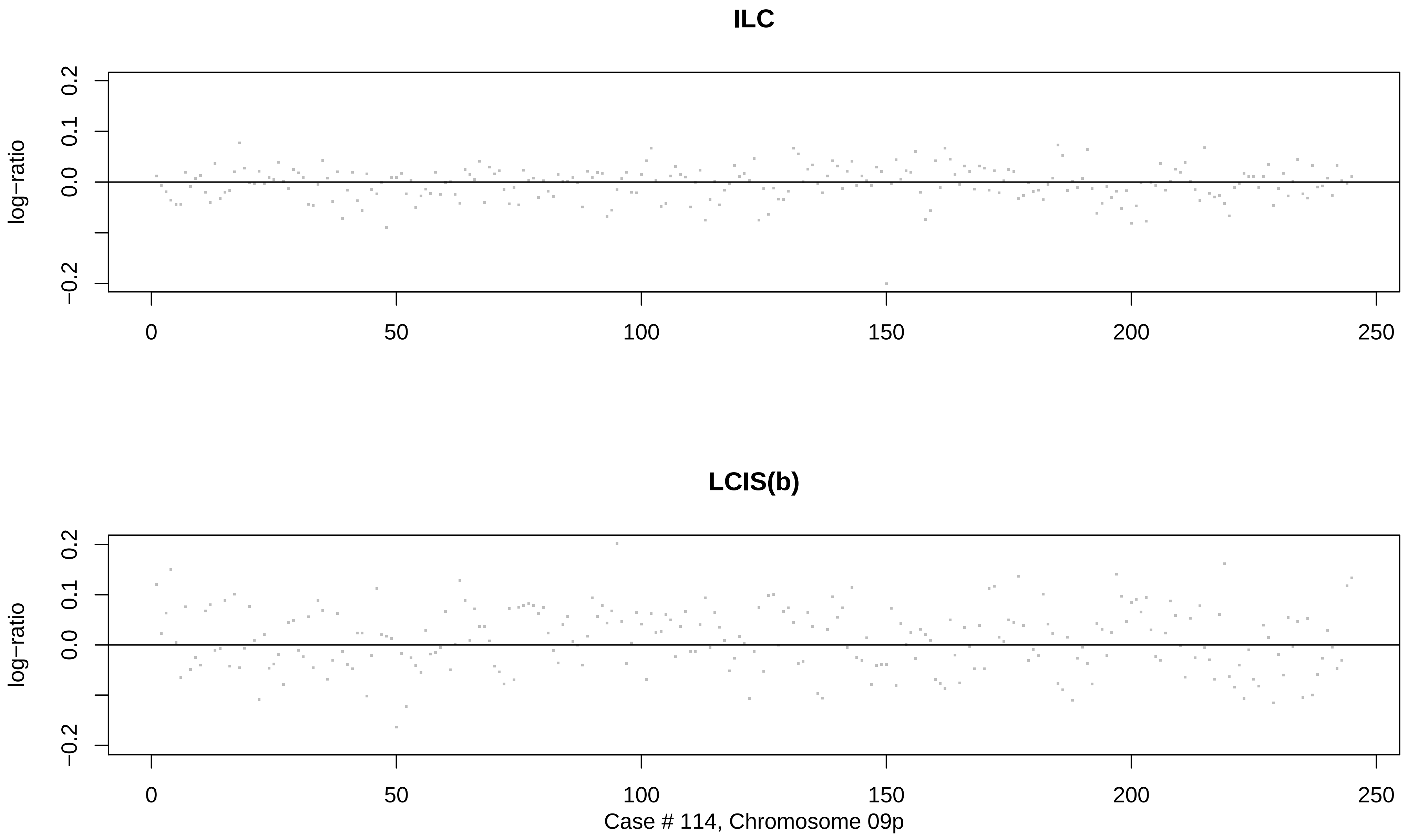

ILC
log−ratio
0.2
0.1
0.0
−0.2
0
50
100
150
200
250
LCIS(b)
log−ratio
0.2
0.1
0.0
−0.2
0
50
100
150
200
250
Case # 114, Chromosome 09p

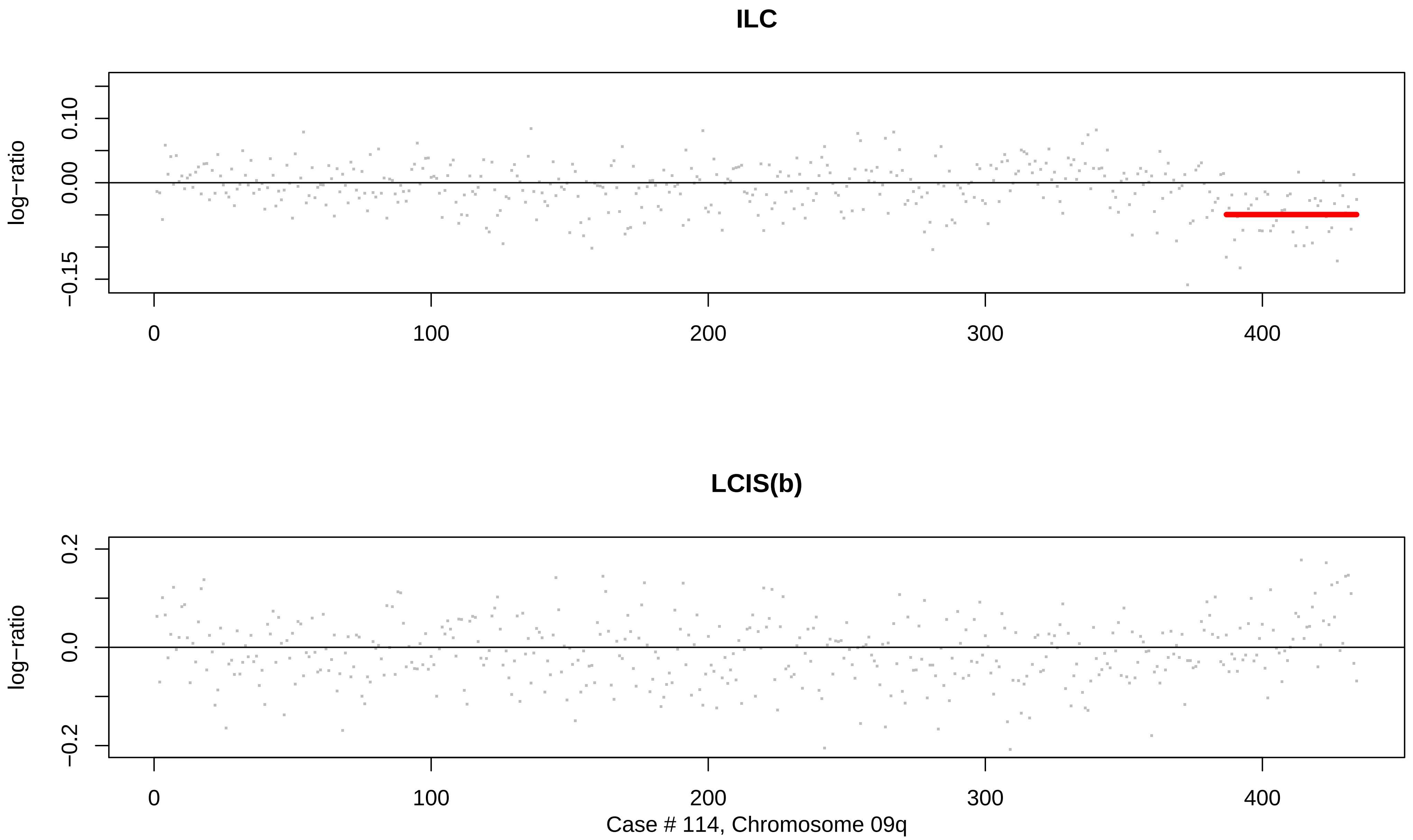

ILC
log−ratio
−0.15
0.00
0.10
0
100
200
300
400
LCIS(b)
log−ratio
−0.2
0.0
0.2
0
100
200
300
400
Case # 114, Chromosome 09q

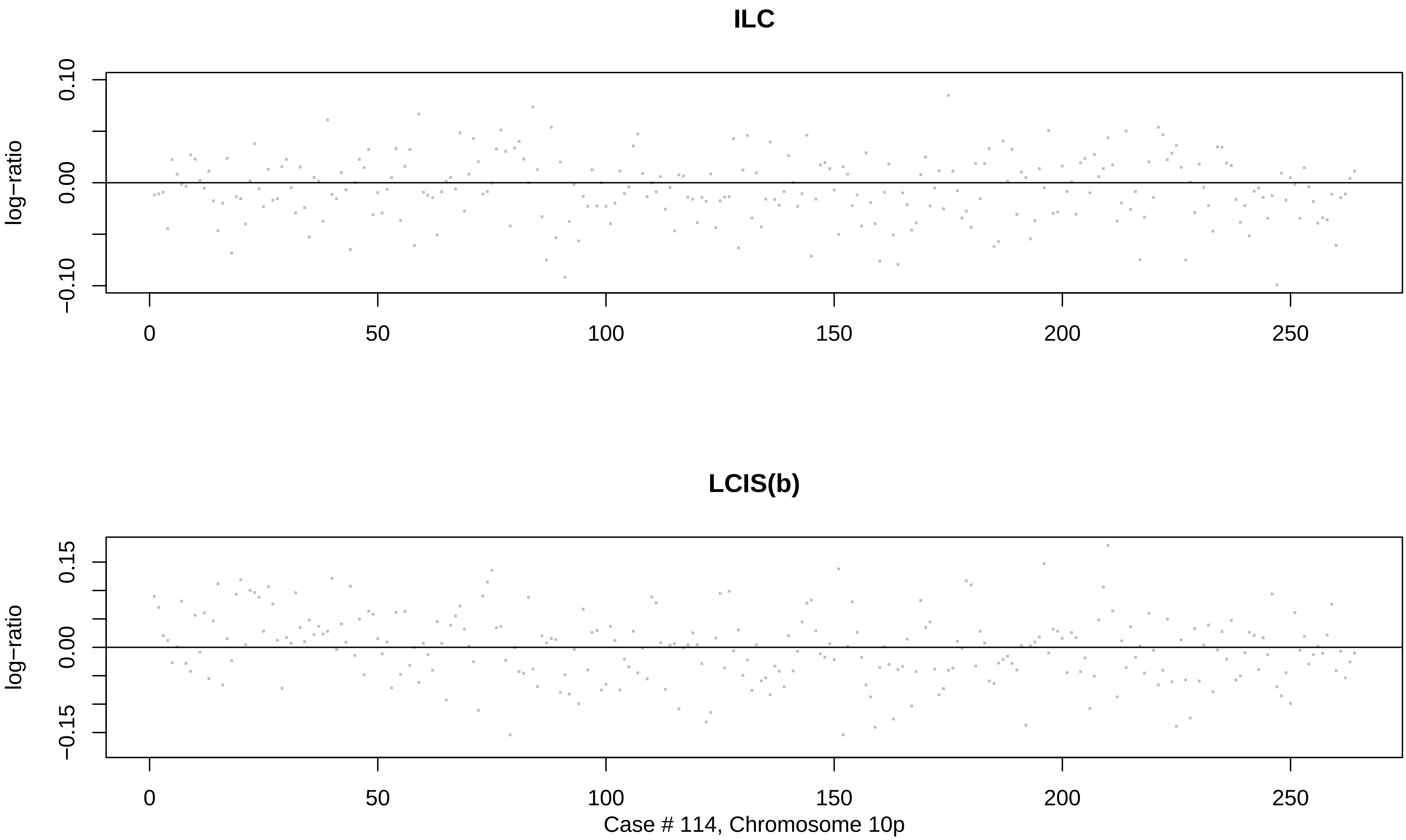

ILC
log−ratio
0.10
0.00
−0.10
0
50
100
150
200
250
LCIS(b)
log−ratio
0.15
0.00
−0.15
0
50
100
150
200
250
Case # 114, Chromosome 10p

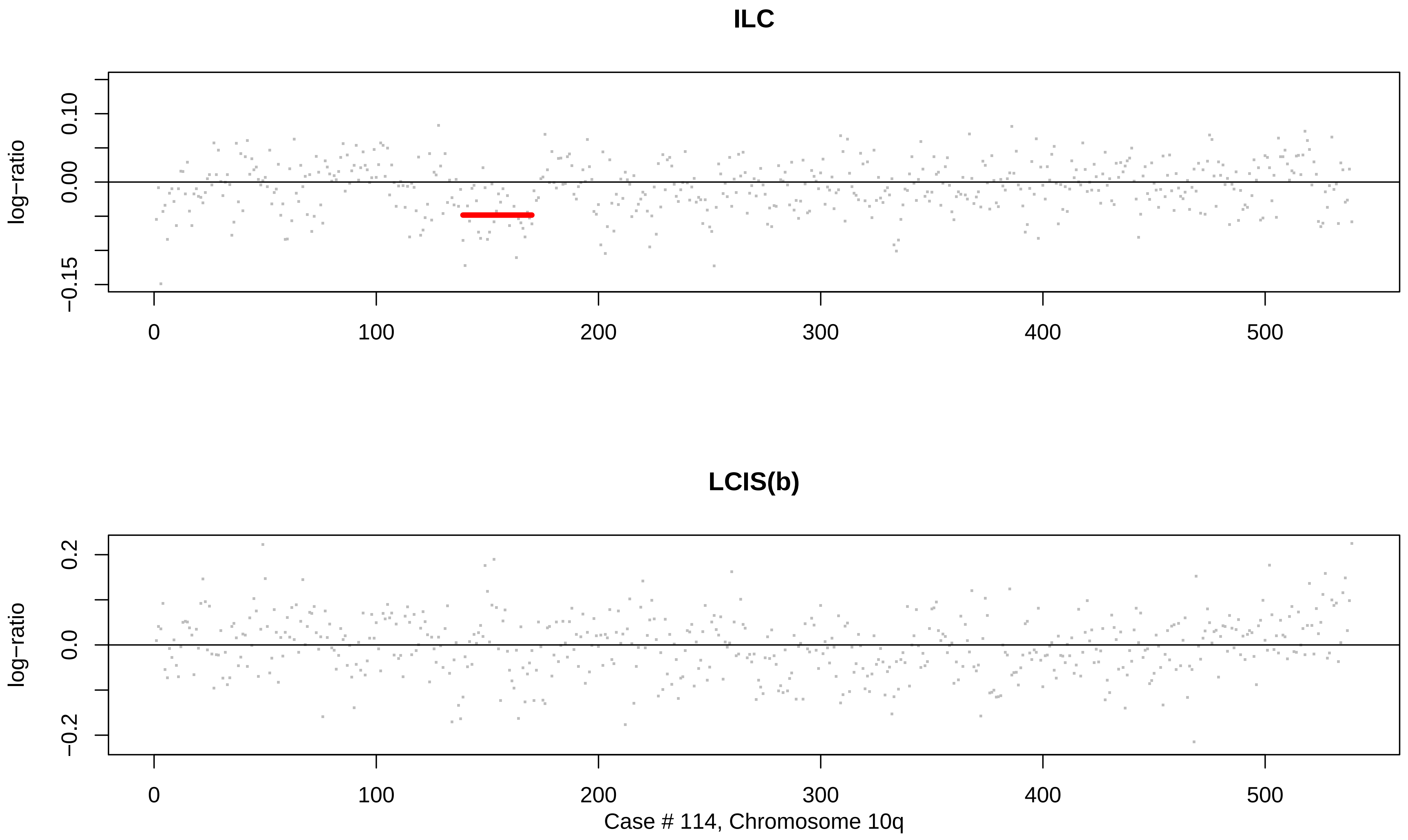
ILC
log−ratio
−0.15
0.00
0.10
0
100
200
300
400
500
LCIS(b)
log−ratio
−0.2
0.0
0.2
0
100
200
300
400
500
Case # 114, Chromosome 10q

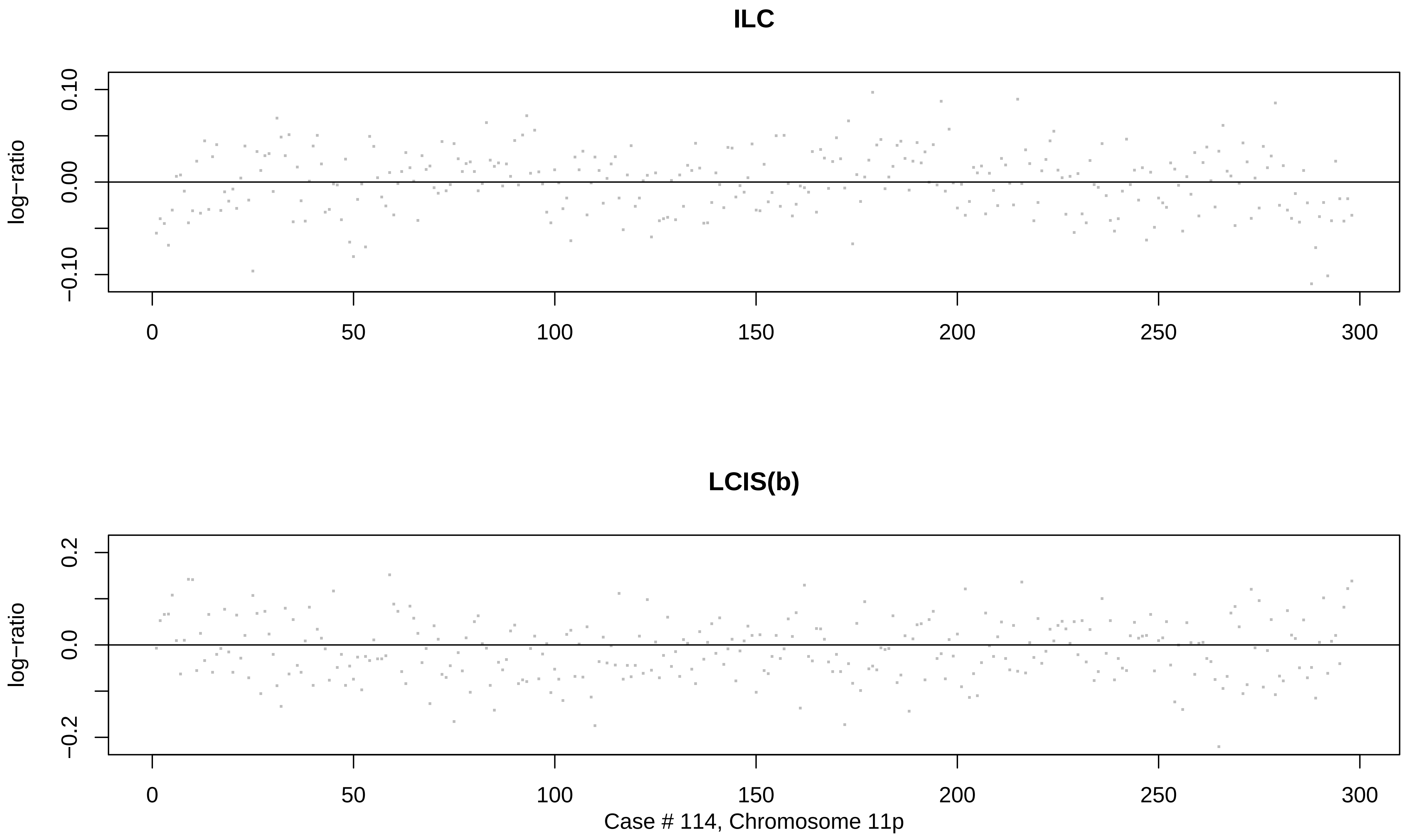
ILC
log−ratio
0.10
0.00
−0.10
0
50
100
150
200
250
300
LCIS(b)
log−ratio
0.2
0.0
−0.2
0
50
100
150
200
250
300
Case # 114, Chromosome 11p

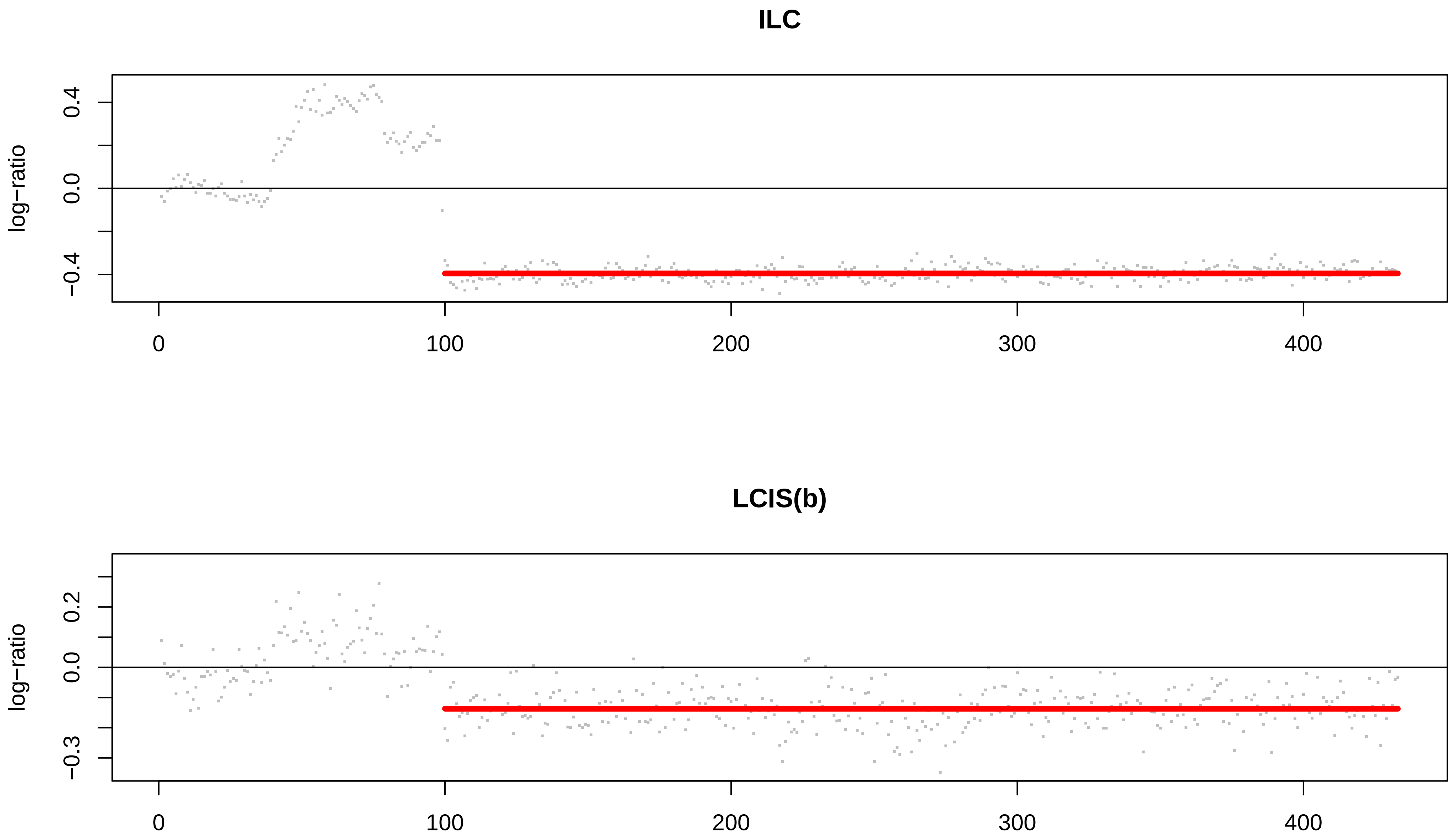

Case # 114, Chromosome 11q
Odds in favor of clonality = 4.5e+02

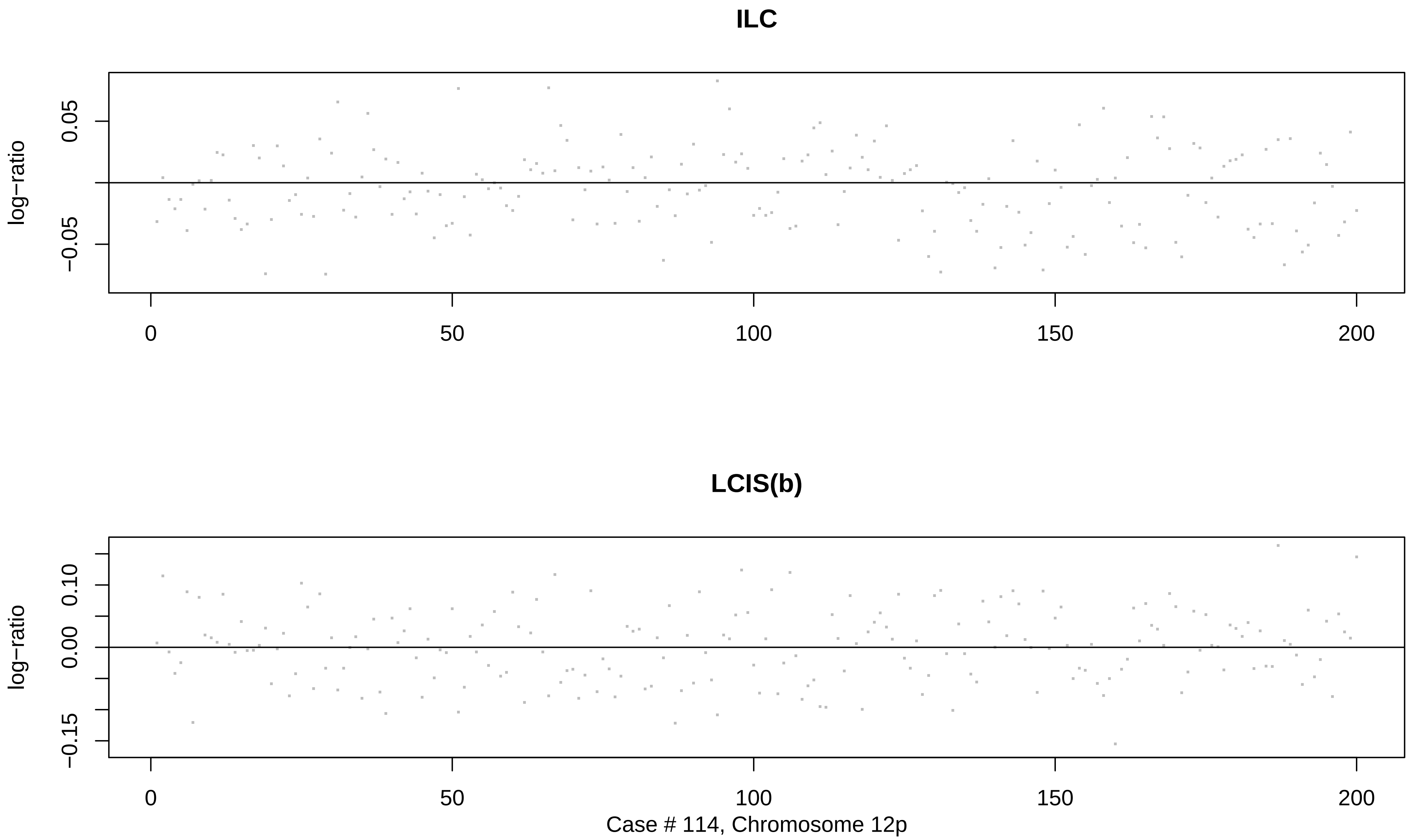
ILC
log−ratio
0.05
−0.05
0
50
100
150
200
LCIS(b)
log−ratio
0.10
0.00
−0.15
0
50
100
150
200
Case # 114, Chromosome 12p

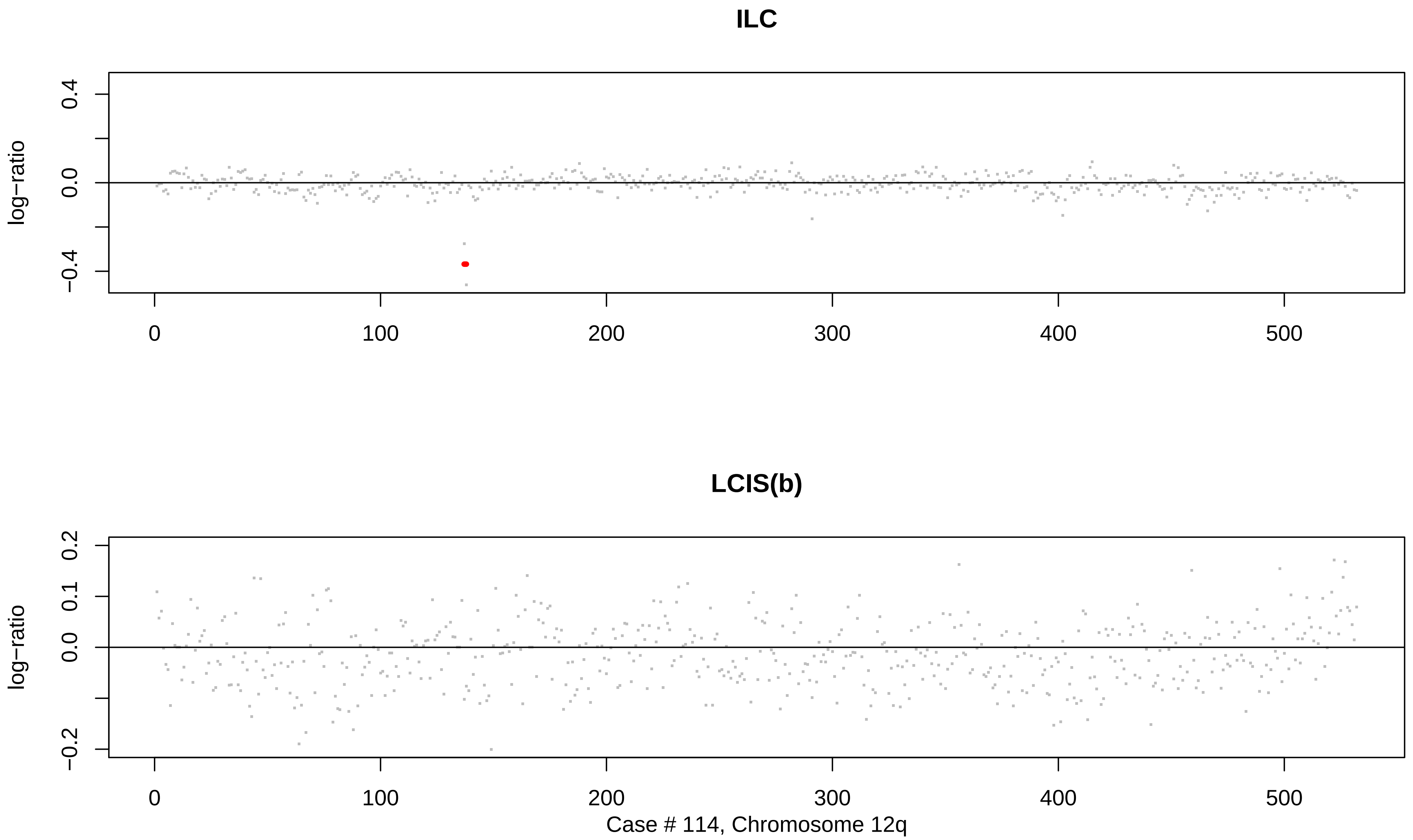
ILC
log−ratio
0.4
0.0
−0.4
0
100
200
300
400
500
LCIS(b)
log−ratio
0.2
0.1
0.0
−0.2
0
100
200
300
400
500
Case # 114, Chromosome 12q

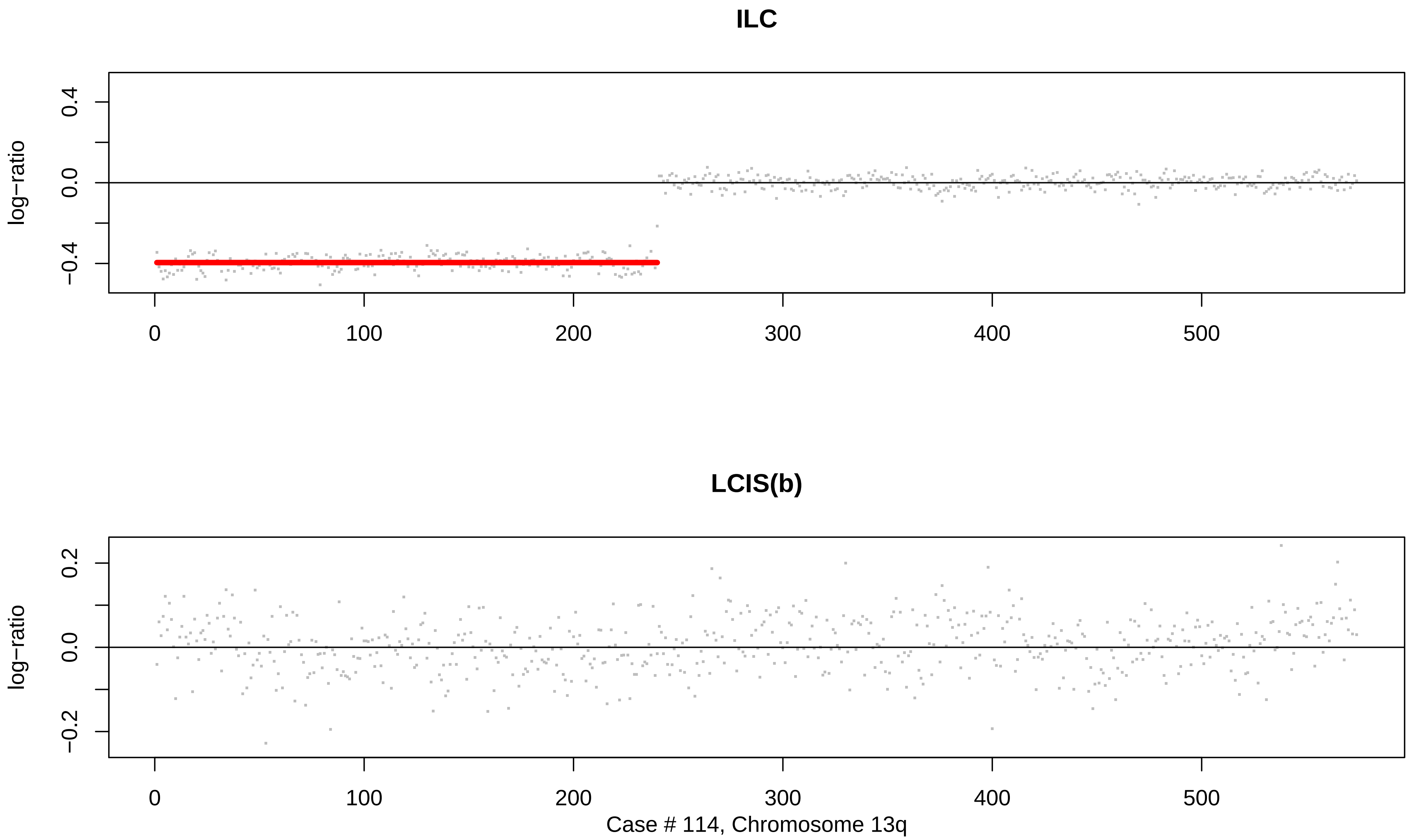
ILC
log−ratio
0.4
0.0
−0.4
0
100
200
300
400
500
LCIS(b)
log−ratio
0.2
0.0
−0.2
0
100
200
300
400
500
Case # 114, Chromosome 13q

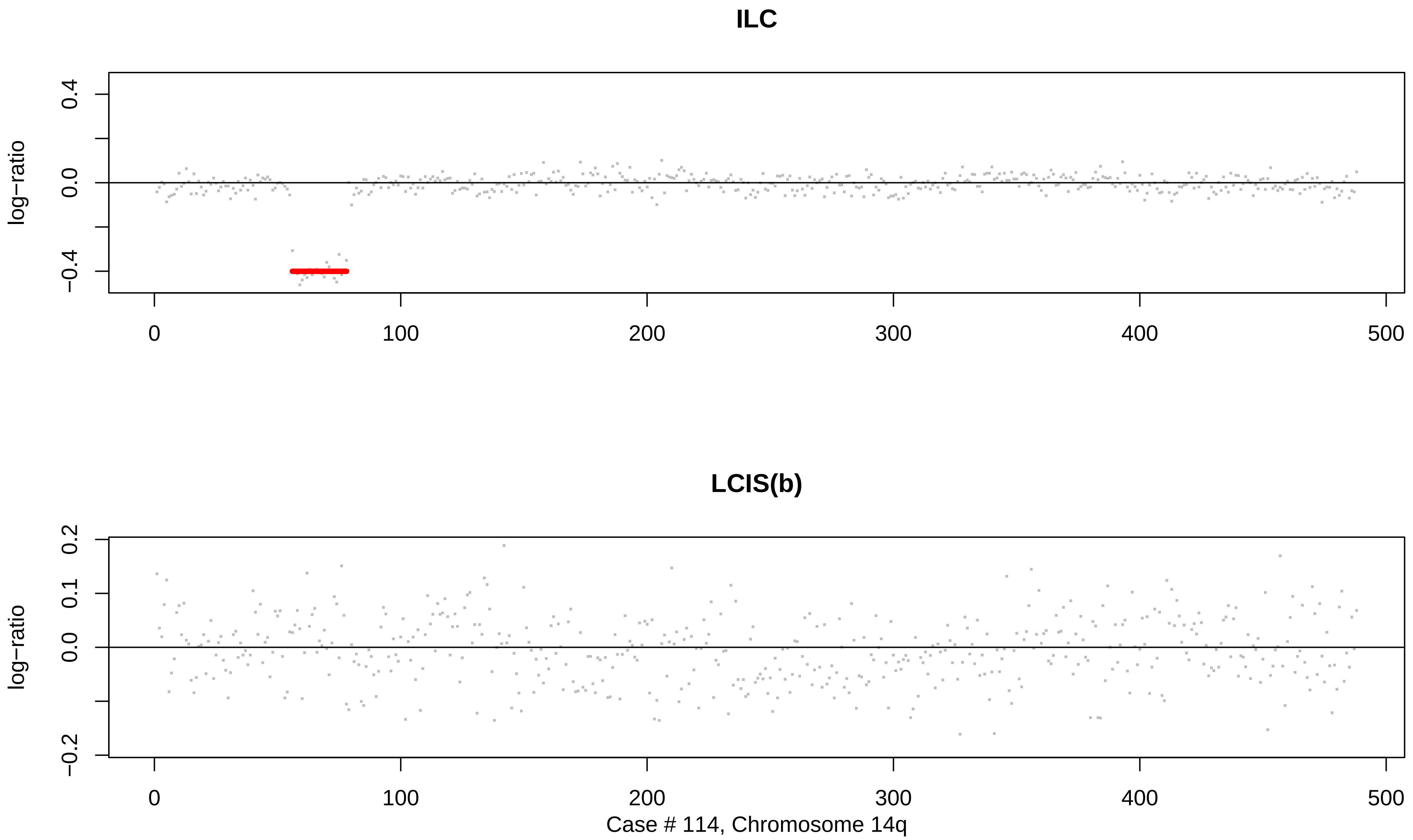
ILC
log−ratio
0.4
0.0
−0.4
0
100
200
300
400
500
LCIS(b)
log−ratio
0.2
0.1
0.0
−0.2
0
100
200
300
400
500
Case # 114, Chromosome 14q

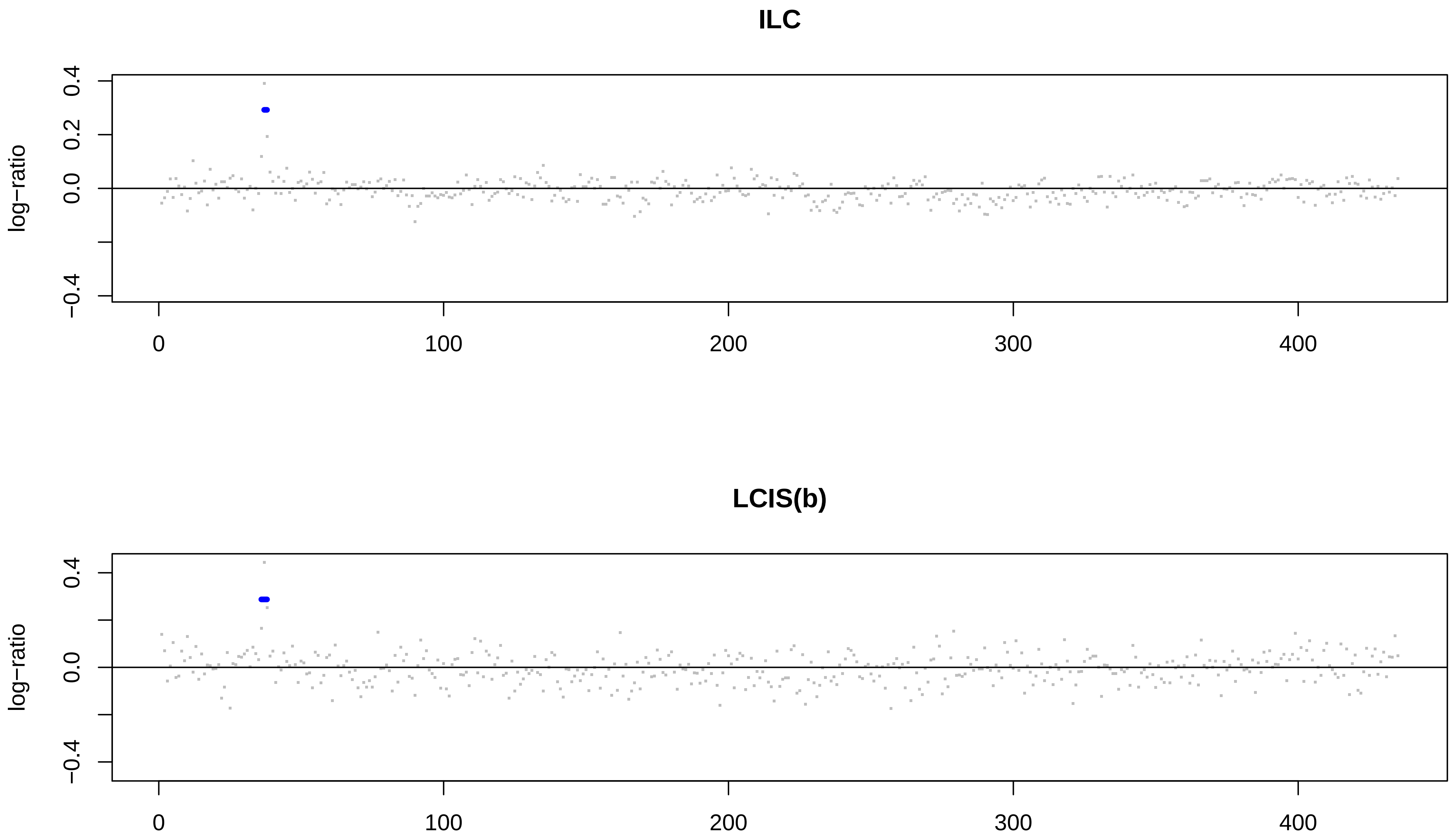

Case # 114, Chromosome 15q
Odds in favor of clonality = 6.8e+02

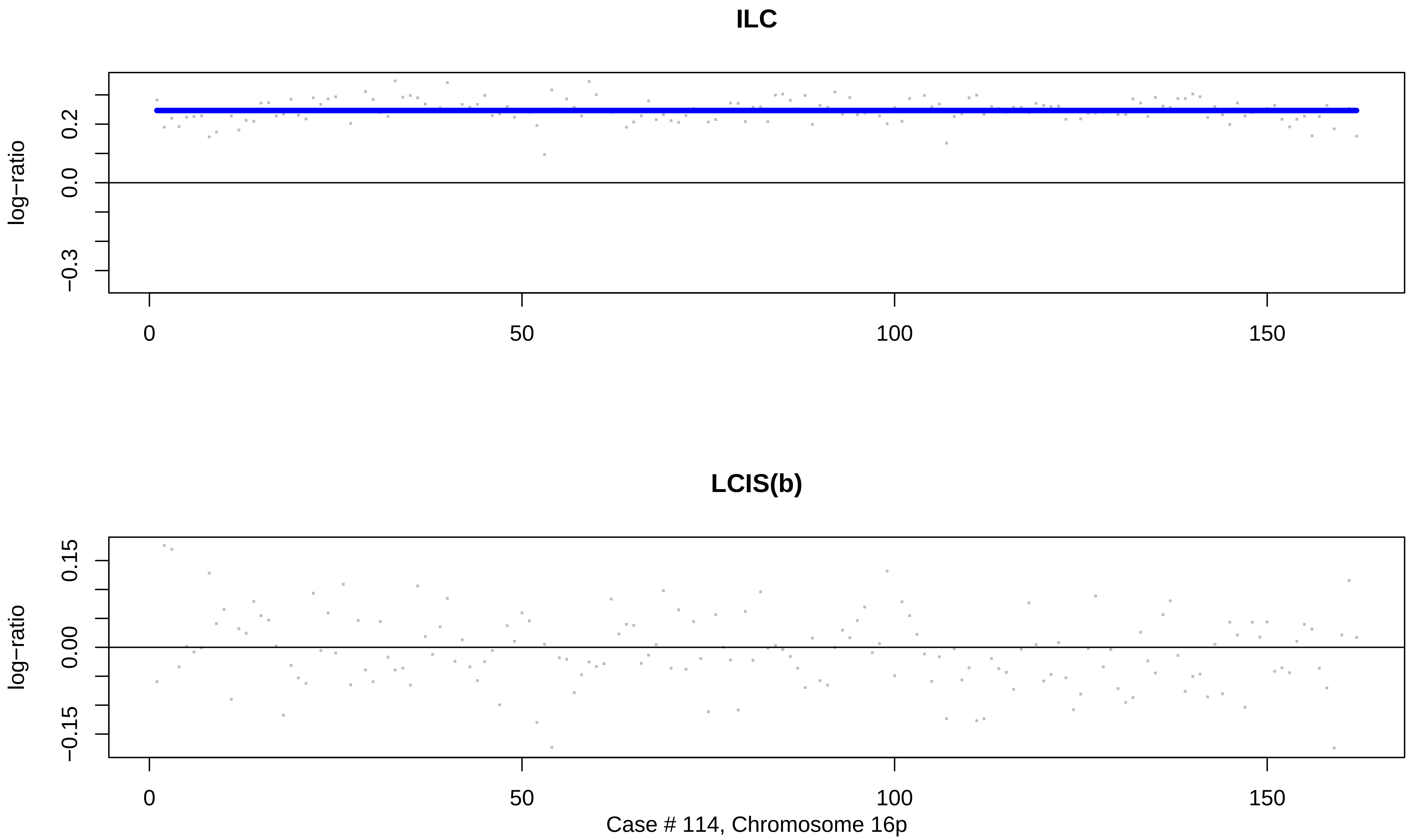
ILC
log−ratio
−0.3
0.0
0.2
0
50
100
150
LCIS(b)
log−ratio
−0.15
0.00
0.15
0
50
100
150
Case # 114, Chromosome 16p

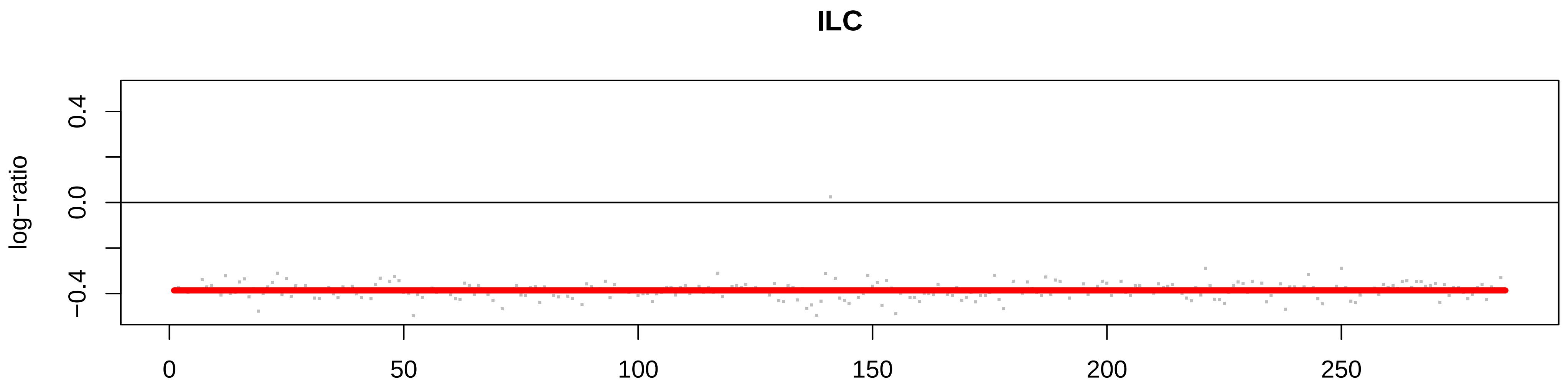
ILC
log−ratio
0.4
0.0
−0.4
0
50
100
150
200
250

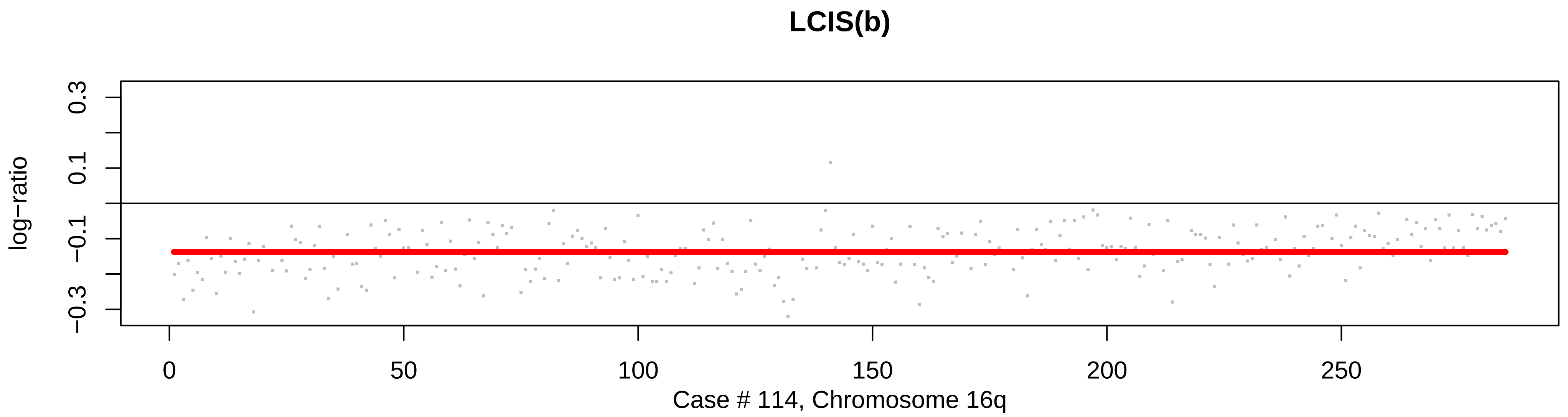
LCIS(b)
log−ratio
0.3
0.1
−0.1
−0.3
0
50
100
150
200
250
Case # 114, Chromosome 16q

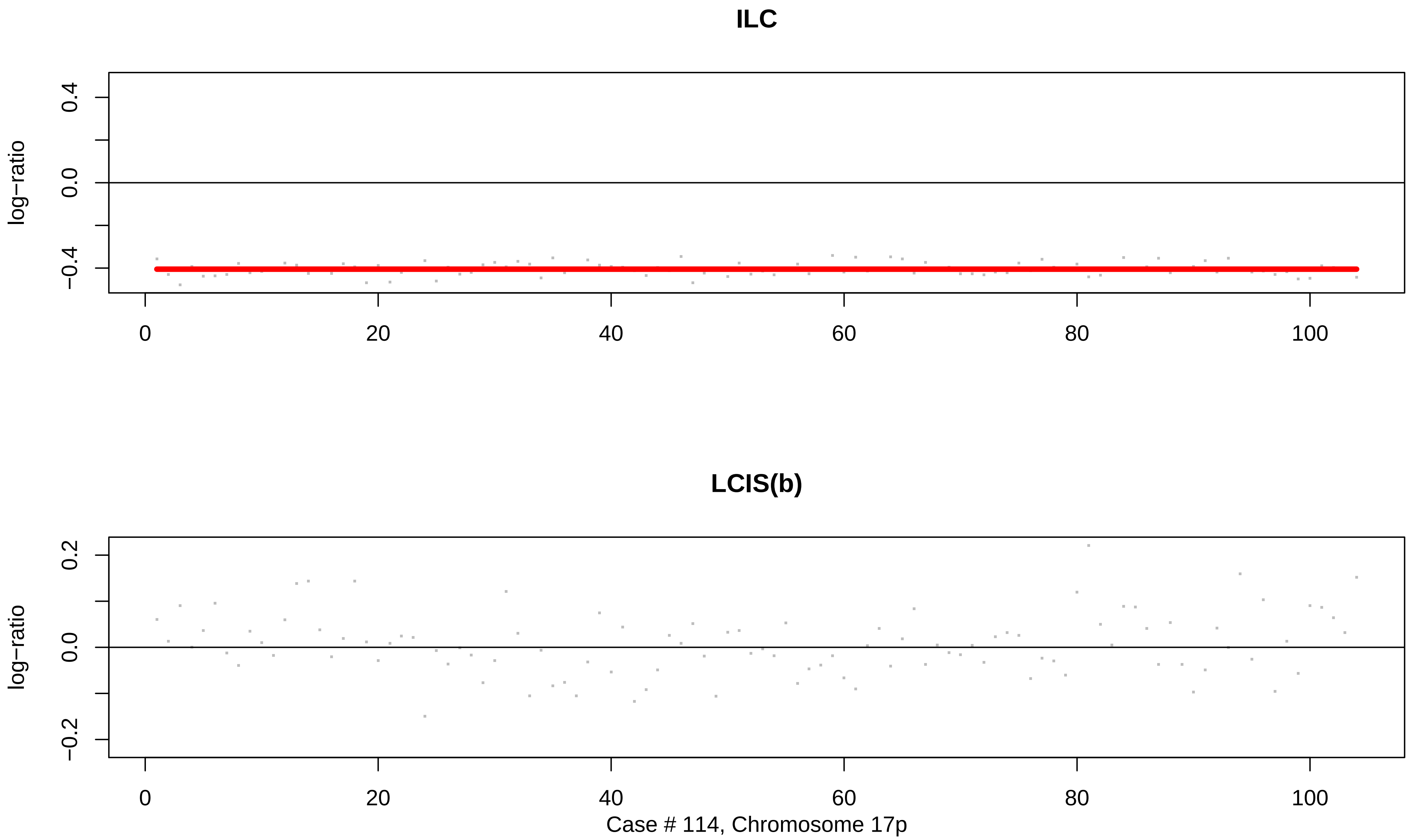

ILC
log−ratio
0.4
0.0
−0.4
0
20
40
60
80
100
LCIS(b)
log−ratio
0.2
0.0
−0.2
0
20
40
60
80
100
Case # 114, Chromosome 17p

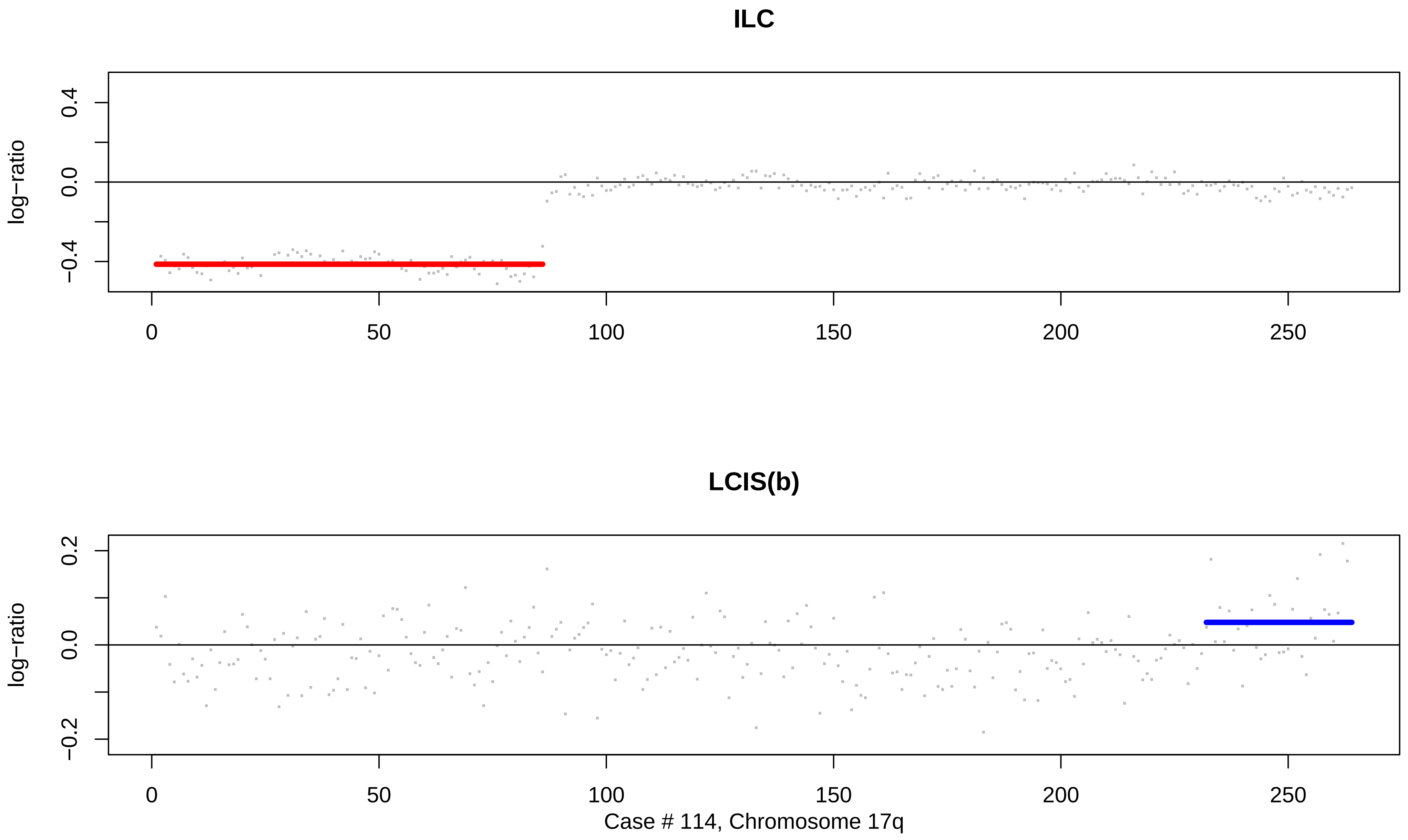
ILC
log−ratio
0.4
0.0
−0.4
0
50
100
150
200
250
LCIS(b)
log−ratio
0.2
0.0
−0.2
0
50
100
150
200
250
Case # 114, Chromosome 17q

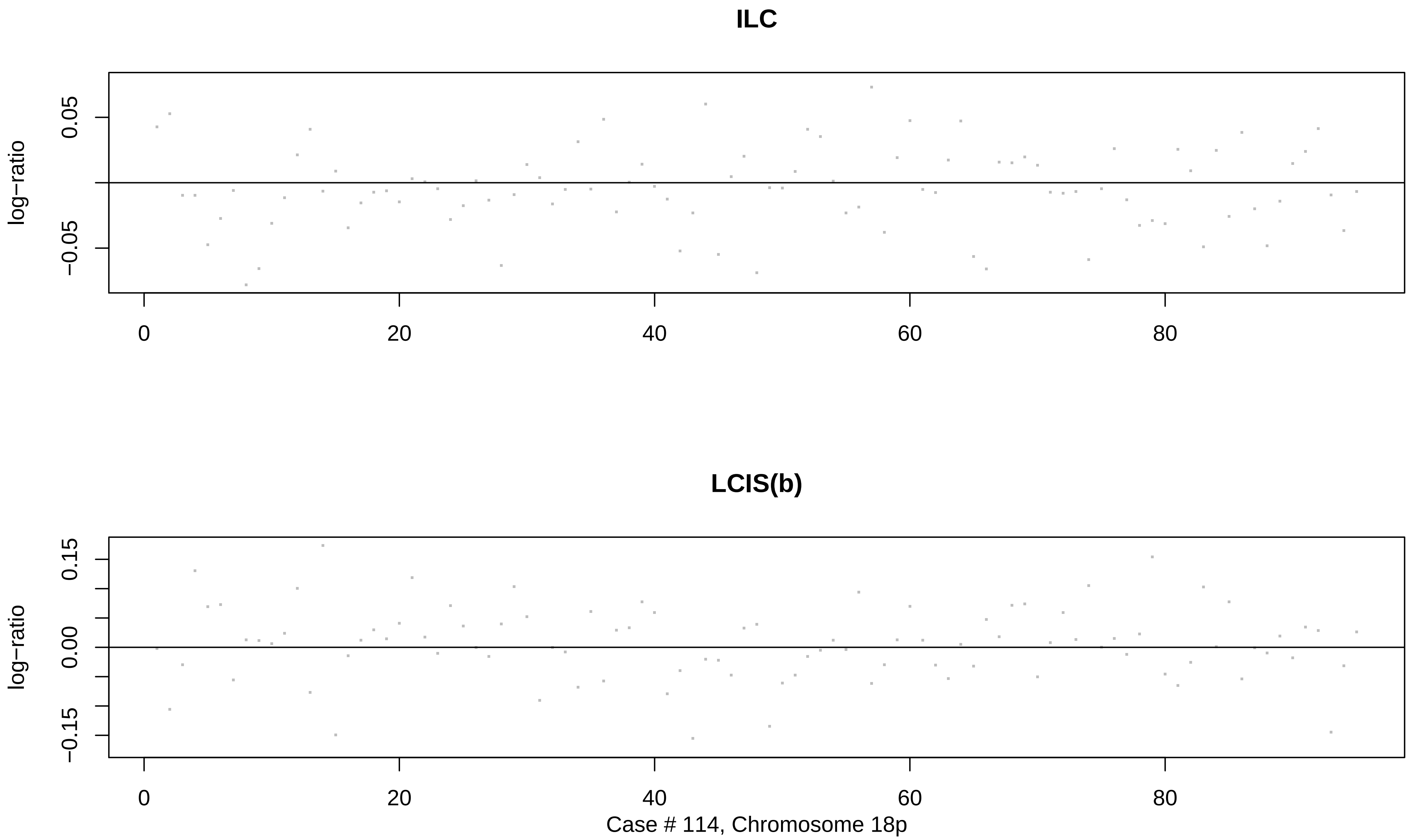

ILC
log−ratio
0.05
−0.05
0
20
40
60
80
LCIS(b)
log−ratio
0.15
0.00
−0.15
0
20
40
60
80
Case # 114, Chromosome 18p

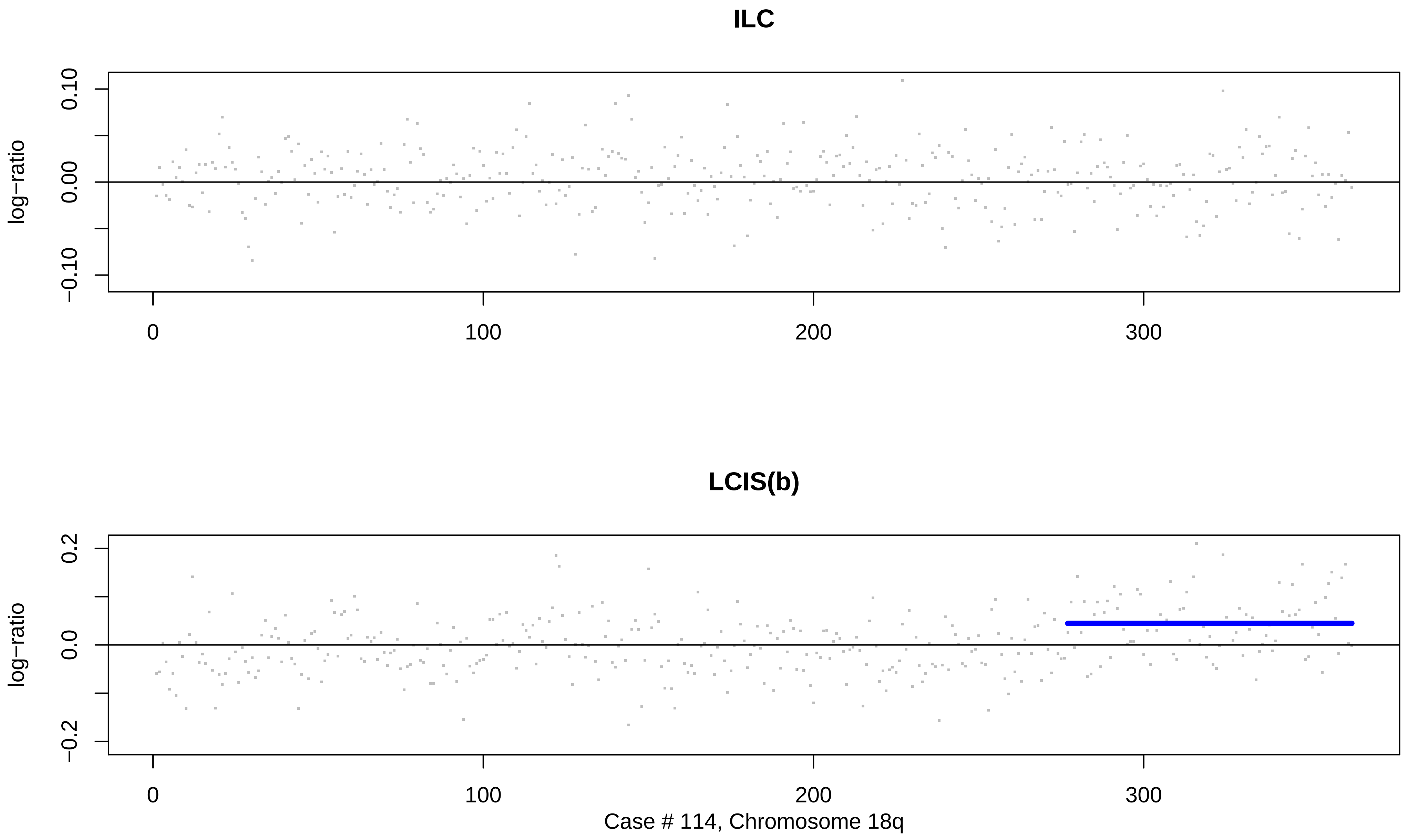
ILC
log−ratio
0.10
0.00
−0.10
0
100
200
300
LCIS(b)
log−ratio
0.2
0.0
−0.2
0
100
200
300
Case # 114, Chromosome 18q

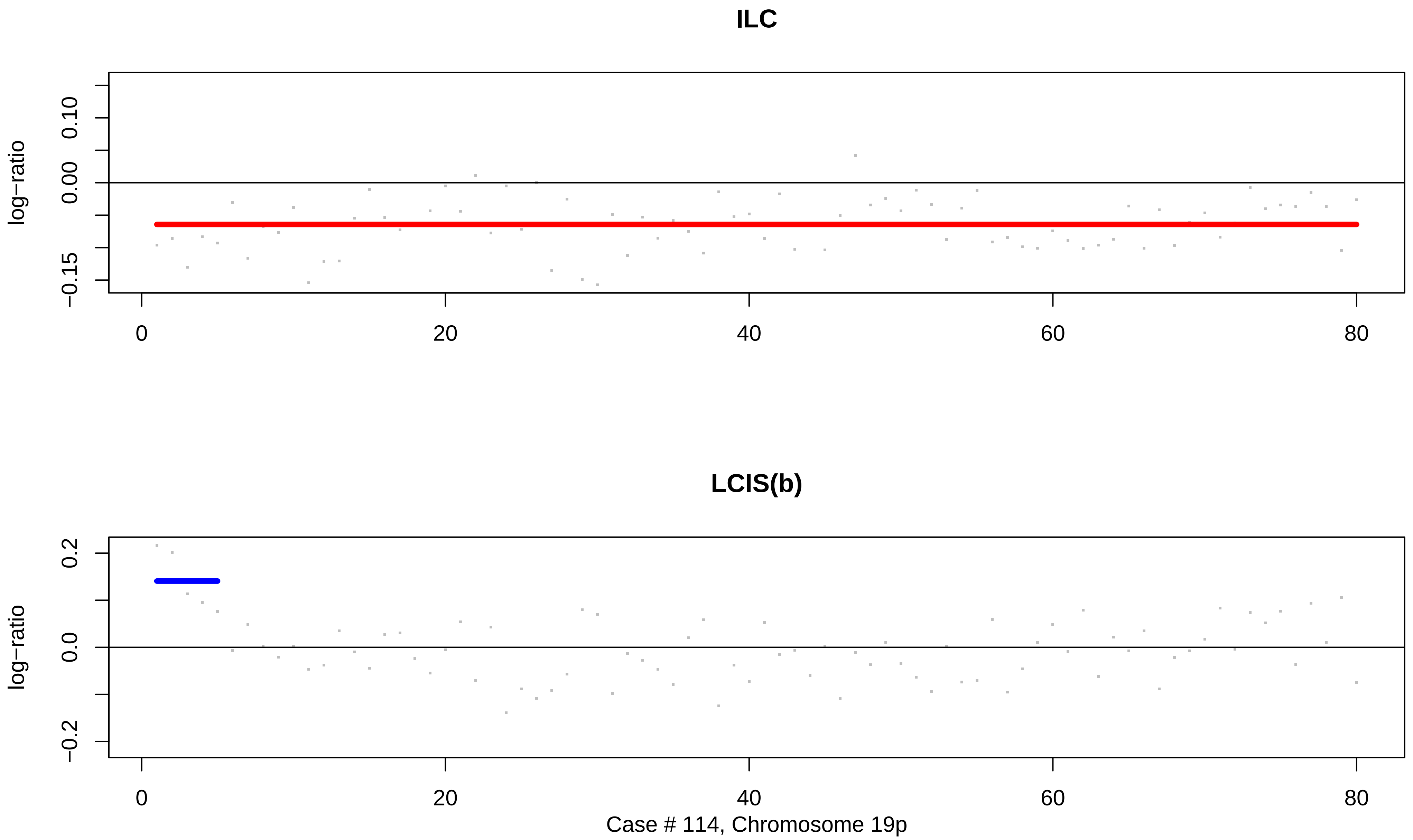
ILC
log−ratio
−0.15
0.00
0.10
0
20
40
60
80
LCIS(b)
log−ratio
−0.2
0.0
0.2
0
20
40
60
80
Case # 114, Chromosome 19p

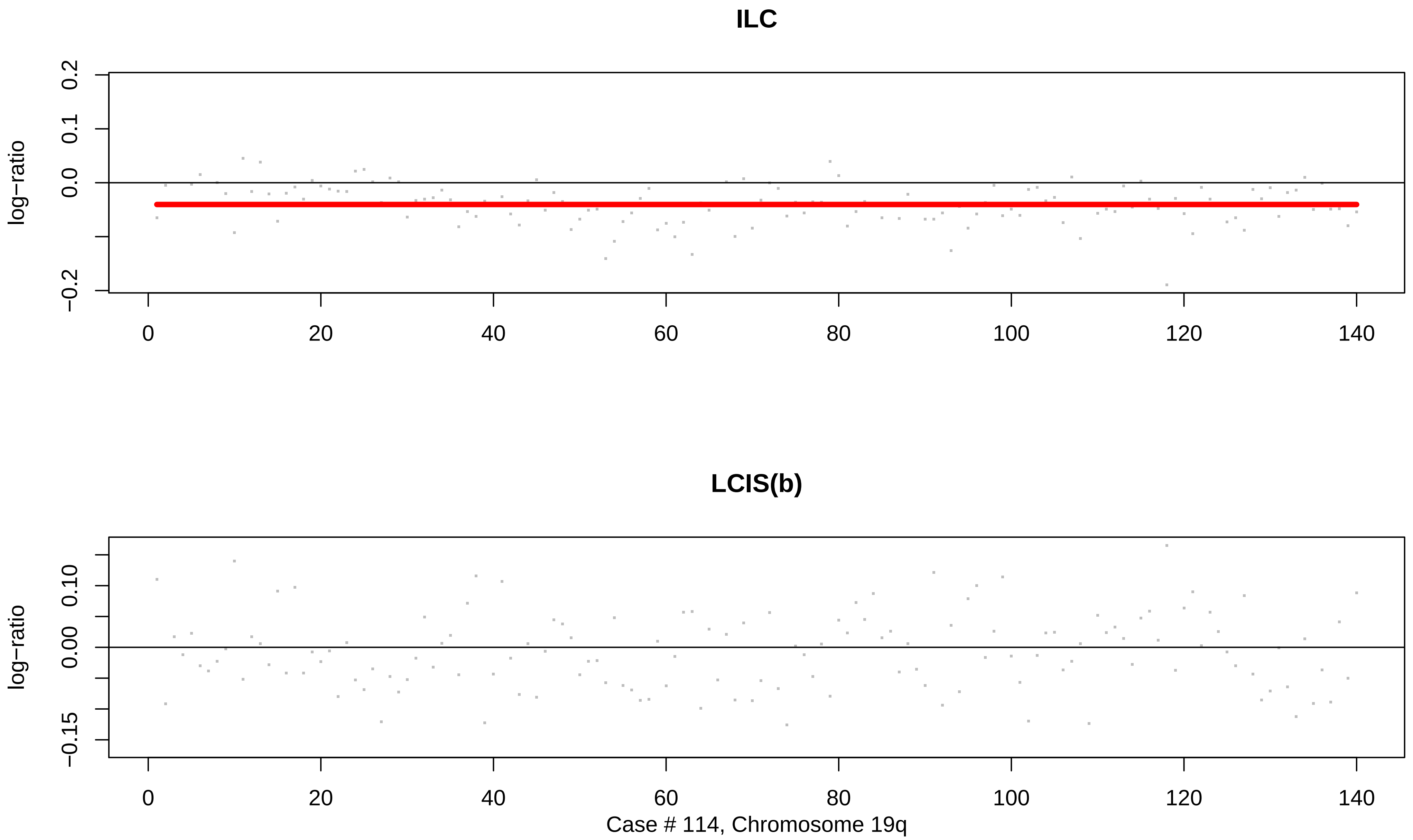
ILC
log−ratio
0.2
0.1
0.0
−0.2
0 20 40 60 80 100 120 140
LCIS(b)
log−ratio
0.10
0.00
−0.15
0 20 40 60 80 100 120 140
Case # 114, Chromosome 19q

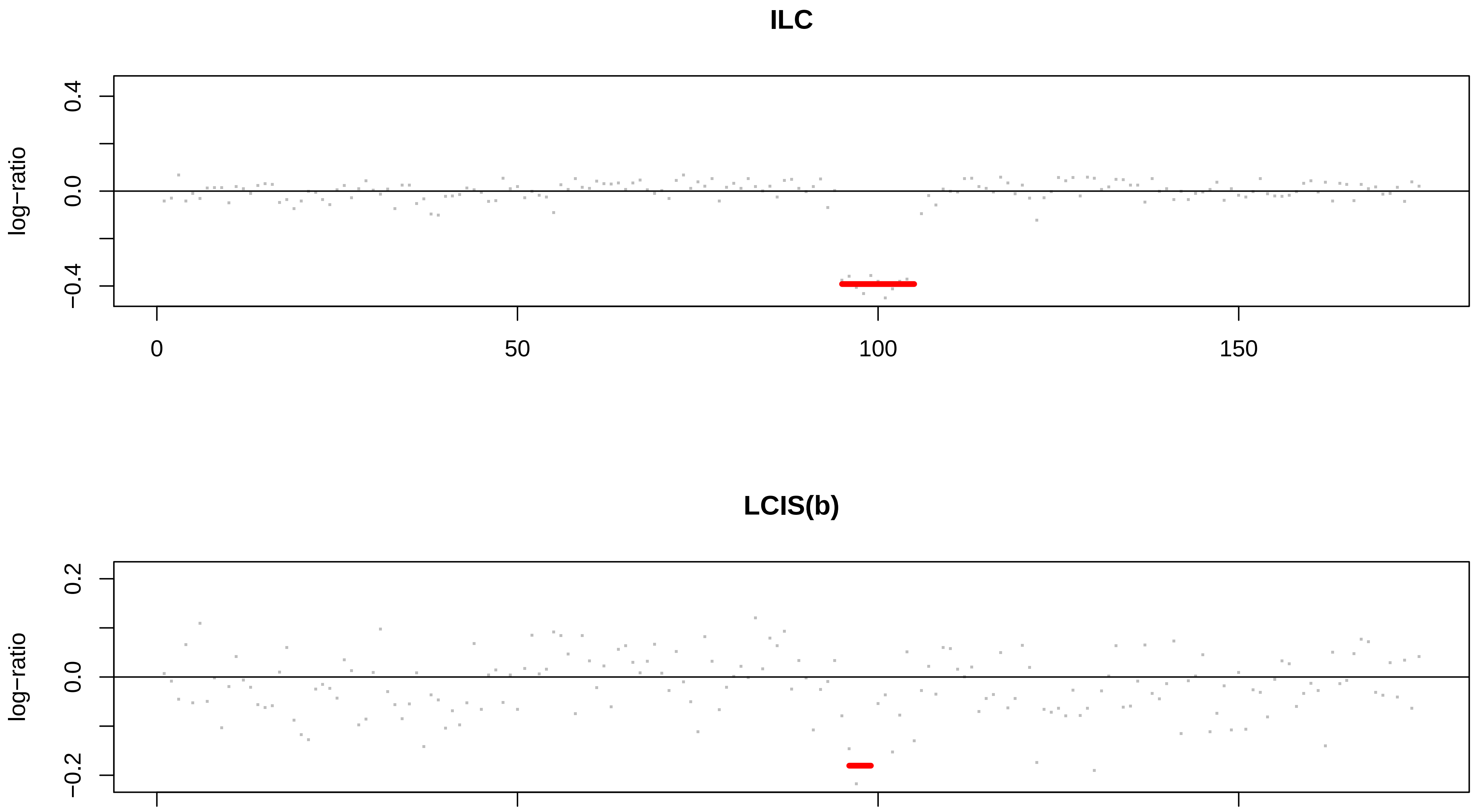

Case # 114, Chromosome 20p
Odds in favor of independence = 6.7

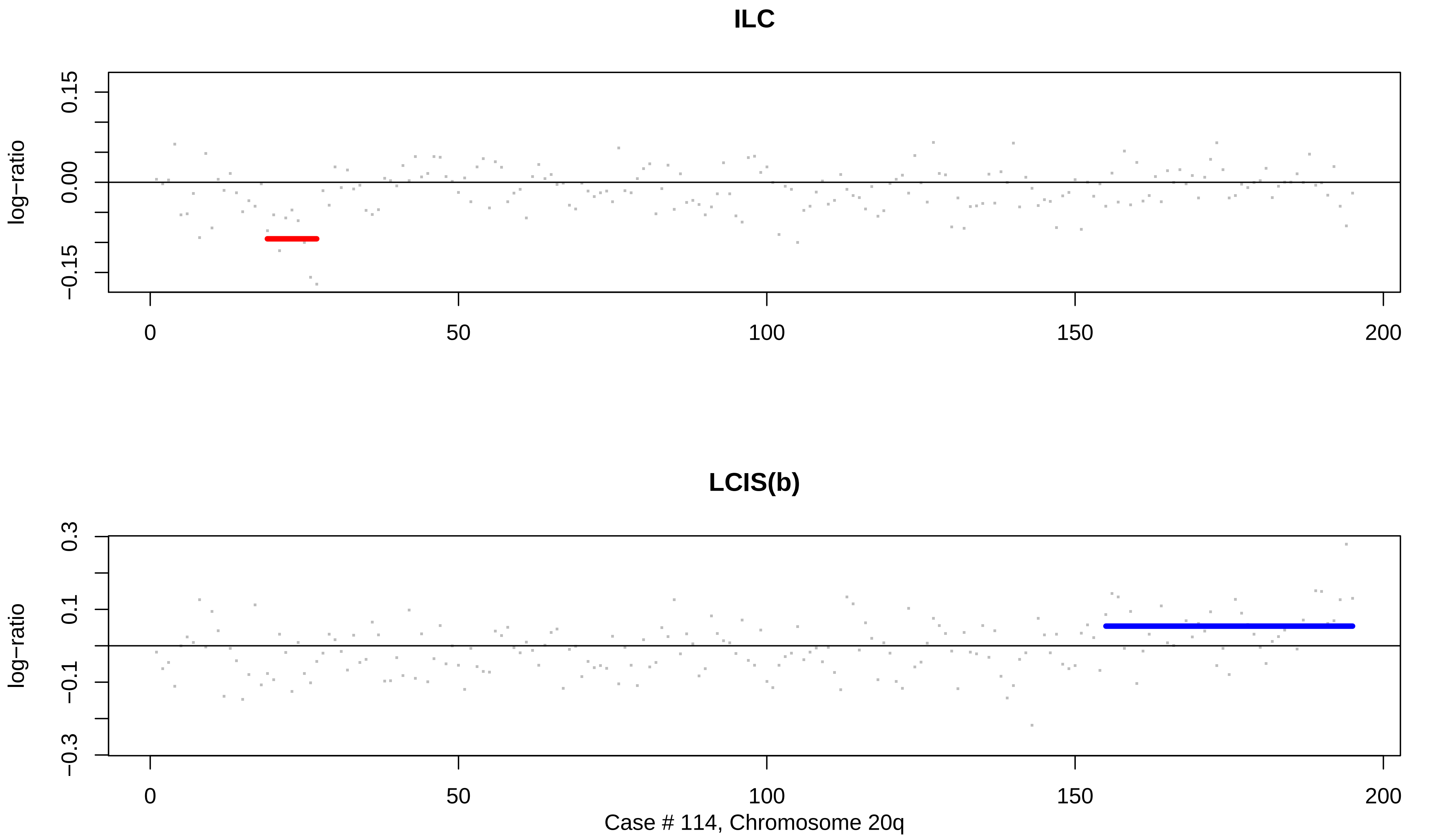
ILC
log−ratio
0.15
0.00
−0.15
0
50
100
150
200
LCIS(b)
log−ratio
0.3
0.1
−0.1
−0.3
0
50
100
150
200
Case # 114, Chromosome 20q

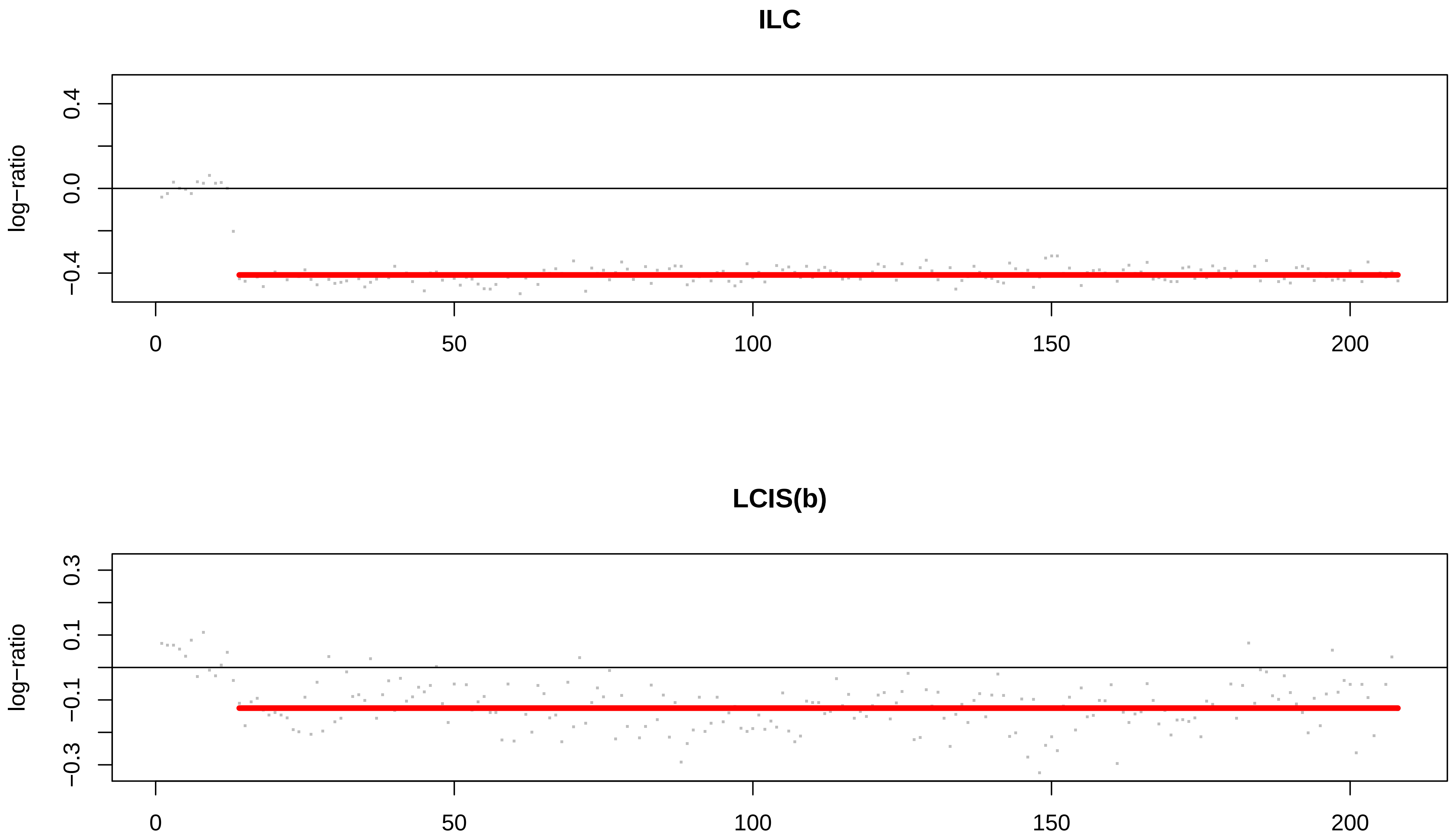

Case # 114, Chromosome 21q
Odds in favor of clonality = 2.4e+02

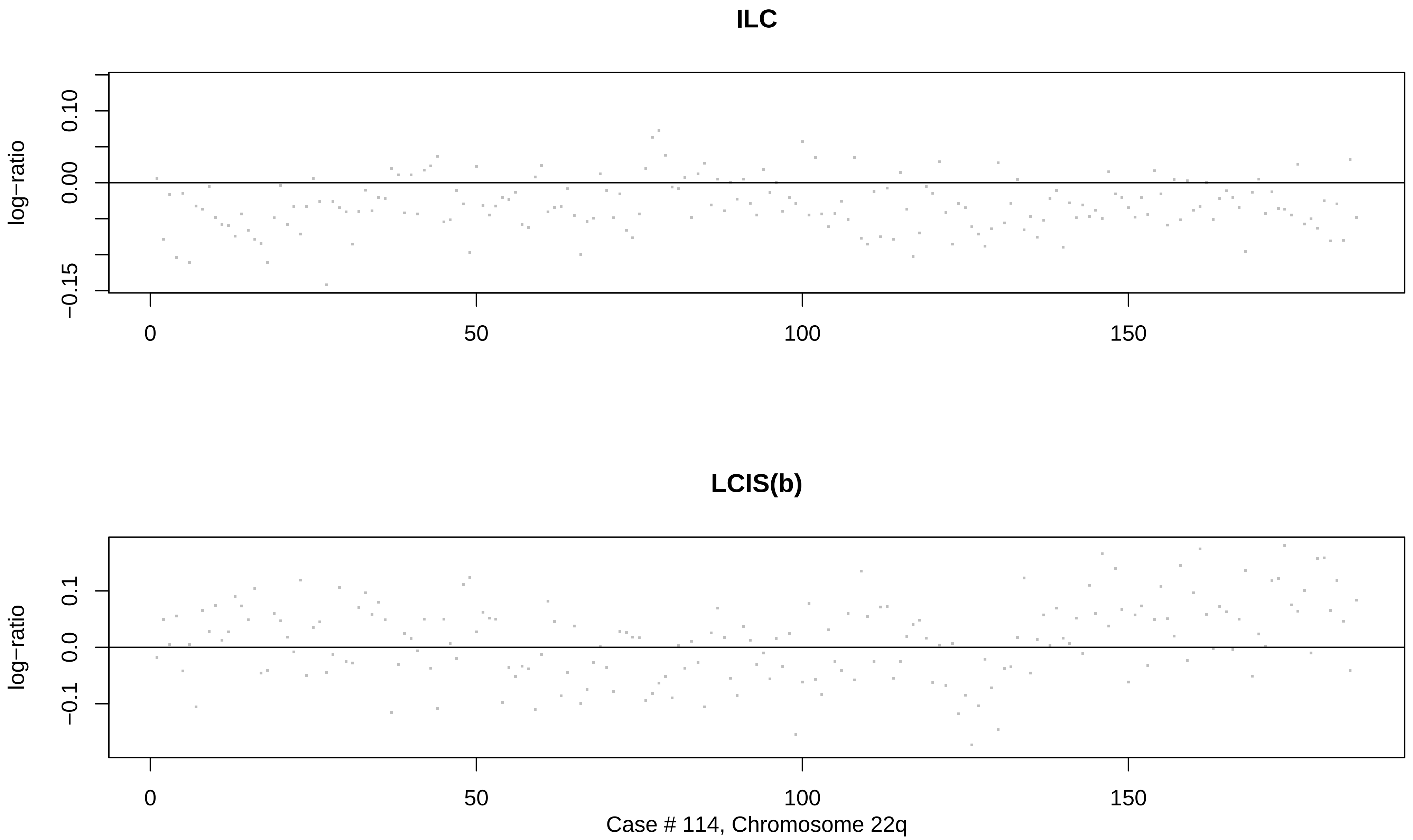

ILC
log−ratio
0.10
0.00
−0.15
0
50
100
150
LCIS(b)
log−ratio
0.1
0.0
−0.1
0
50
100
150
Case # 114, Chromosome 22q
