# Supplementary material for: Clonal relatedness between lobular carcinoma in situ and synchronous malignant lesions
Source: Breast Cancer Res. 2012 Jul 9;14(4):R103. doi: 10.1186/bcr3222 (PMC3680923; doi:10.1186/bcr3222)

Case #122: LCIS and paired invasive lobular carcinoma of pleomorphic morphology.

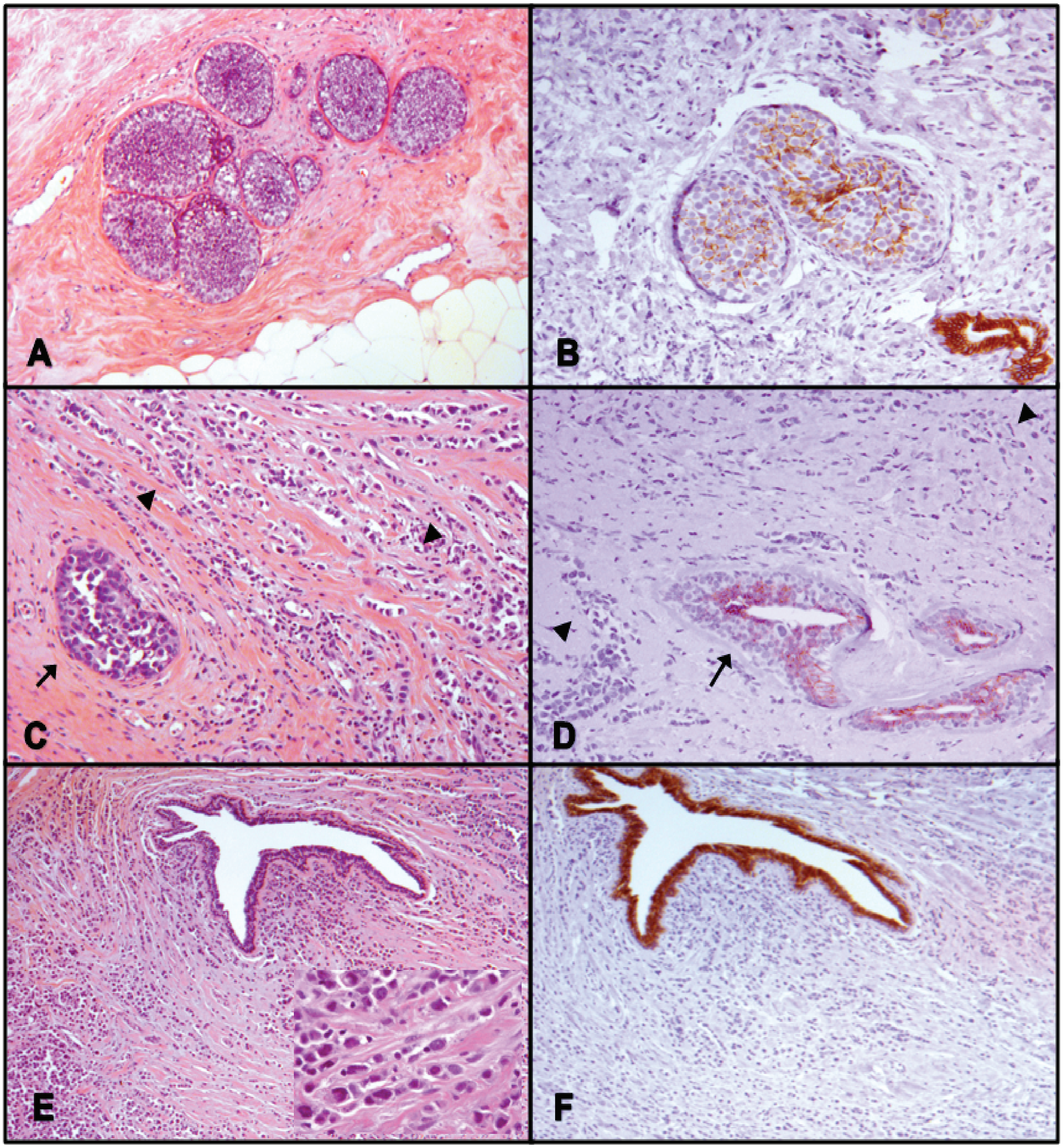

Case #93: LCIS and associated invasive lobular carcinoma with no immunoreactivity for E-Cadherin.

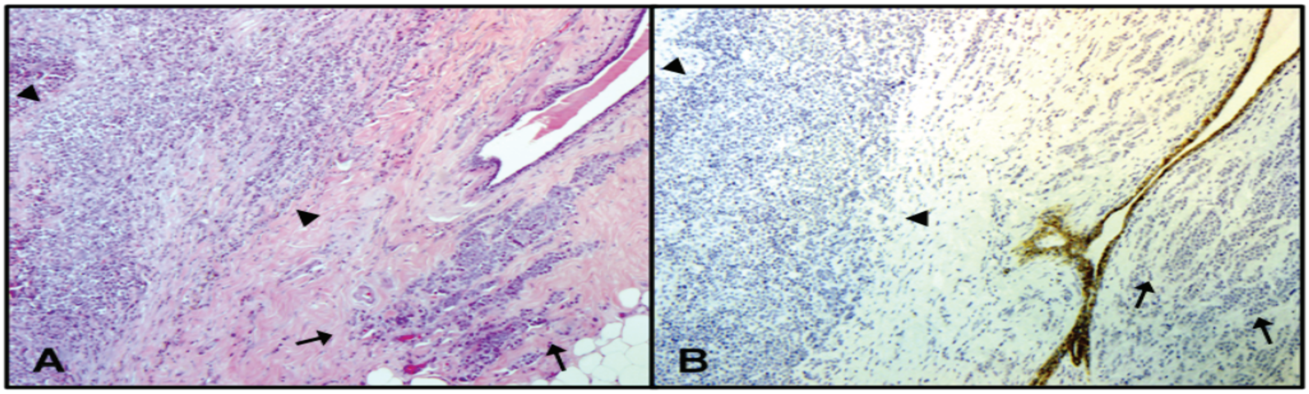

Supplement: Additional file 5 — Representative photomicrographs of Case #122 and Case #93. Case #122: LCIS and paired invasive lobular carcinoma of pleomorphic morphology. (A), (B) LCIS of pleomorphic morphology with no E-cadherin immunoreactivity. Residual luminal cells show membrane staining: (A) H & E, 40×; (B) E-cadherin, 100×. (C), (D) Focus of LCIS (arrows) and associated invasive lobular carcinoma (ILC, arrowheads) both of pleomorphic morphology showing lack of membranous reactivity for E-cadherin: (C) H & E, 40×; (D) E-cadherin, 100×. (E), (F) ILC pleomorphic variant with no positivity with E-cadherin staining as opposed to a normal duct. The inset image at 400× magnification illustrates tumor cells with abundant eosinophilic cytoplasm and high nuclear grade in a single-file infiltrating pattern: (E) H & E, 40×; (F) E-cadherin, 40×. Case #93: LCIS and associated invasive lobular carcinoma with no immunoreactivity for E-cadherin. (A) Classic LCIS and invasive lobular carcinoma (ILC) surrounding a normal duct (H & E, 40×). (B) E-cadherin staining shows lack of membranous reactivity in LCIS and ILC as opposed to the adjacent normal terminal duct (E-cadherin, 40×). [file bcr3222-S5.PDF]
